# Supplementary material for: Organocatalytic diastereo- and atroposelective construction of N–N axially chiral pyrroles and indoles
Source: Nat Commun. 2024 Jan 15;15:518. doi: 10.1038/s41467-024-44743-z (PMC10789812; doi:10.1038/s41467-024-44743-z)
Supplement: Supplementary file 1 — Supplementary Information [file 41467_2024_44743_MOESM1_ESM.pdf]

Supplementary Information

Organocatalytic Diastereo- and Atroposelective Construction of N–N Axially Chiral Pyrroles and Indoles

Shao-Jie Wang,[1] Xia Wang,[1] Xiaolan, Xin,[1] Shulei Zhang,[1] Hui Yang,[2] Ming Wah Wong,[2]* and Shenci Lu[1]*

[1]Frontiers Science Center for Flexible Electronics (FSCFE), Shaanxi Institute of Flexible Electronics (SIFE) & Shaanxi Institute of Biomedical Materials and Engineering (SIBME), Northwestern Polytechnical University (NPU), 127 West Youyi Road, Xi'an 710072, China

[2]Department of Chemistry, National University of Singapore, 3 Science Drive 3, Republic of Singapore, 117543

email: iamsclu@nwpu.edu.cn; chmwmw@nus.edu.sg

Table of Content

1. General information S3

2. General procedure for the synthesis of isothioureas and thioureas S4

3. General procedure for the synthesis of N–N axially chiral pyrroles and indoles 3 ... S16

4. Supplementary Table 1. Optimization of the Reaction Conditions............................. S17

5. General procedure for the synthesis of N–N axially chiral pyrroles and indoles 7 ... S19

6. Synthetic Transformations of Product.. S21

7. Non-linear effects.. S24

8. Enantiomerization barrier determination for 9c.. S28

9. Characterizations of N–N axially chiral pyrroles and indoles S30

10. X-ray Crystallographic Data S63

11. NMR spectra and Chiral HPLC chromatograms.. S68

12. Computational Methods S250

13. References S253

1. General information

^{1}H and **^{13}C NMR** spectra were recorded on a Bruker AMX500 (500 MHz) spectrometer. Chemical shifts were reported in parts per million (ppm), and the residual solvent peak was used as an internal reference: ^{1}H (chloroform δ 7.26), ^{13}C (chloroform δ 77.0). Data are reported as follows: chemical shift, multiplicity (s = singlet, d = doublet, t = triplet, q = quartet, m = multiplet, br = broad), coupling constants (Hz) and integration. Melting point (**MP**) was obtained on Buchi B-540. For thin layer chromatography (**TLC**), Merck pre-coated TLC plates (Merck 60 F254) were used, and compounds were visualized with a UV light at 254 nm. High resolution mass spectra (**HRMS**) were obtained on a Finnigan/MAT 95XL-T spectrometer. **Optical rotations** were measured using an Anton Paar MCP-150 digital polarimeter using a 1 cm glass cell. **Enantiomeric excesses** (ee) were determined by HPLC analysis on ThermoFisher HPLC units, including the following instruments: pump, LPG- 3400SDN; detector, VWD-3400RS; column, Chiralcel OD-H, Chiralpak AD-H, Chiralpak IA, Chiralpak IC. The NHC precursors were synthesized following the reported procedure.[1,2]

2. General procedure for the synthesis of isothioureas and thioureas

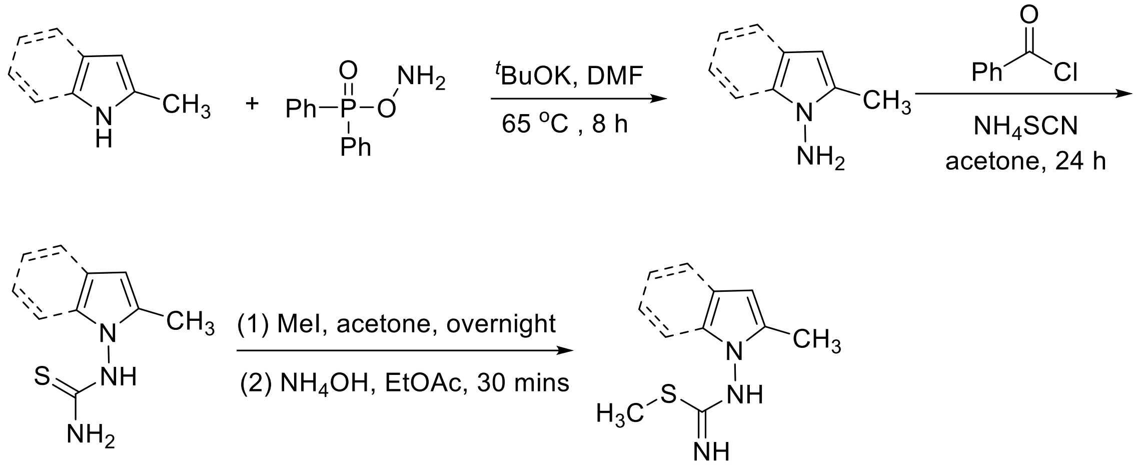

To a solution of either indole or carbazole (20 mmol, 1.0 equiv.) and ^{t}BuOK (30 mmol, 1.5 equiv.) in dimethylformamide (75 mL), was added a solution of ODPH (32 mmol, 1.6 equiv.) in dimethylformamide (75 mL). The reaction mixture was stirred at 65 °C for 8 h, then water (100 mL) was added and the solution extracted with EtOAc (3x50 mL). The extracts were dried over magnesium sulfate and filtered. The residue was purified by silica gel column chromatography (dichloromethane/petroleum ether, v/v = 1:3). The desired products were obtained in 33-45% yield.

Benzoyl chloride (5 mmol, 1.0 equiv.) was dissolved in acetone (2 mL), and the solution was stirred at room temperature. NH_4SCN (6 mmol, 1.2 equiv.) was dissolved in acetone (1 mL) and then added dropwise to the reaction mixture, which was then stirred for 15 min. The precipitate was removed by filtration, and then the filtrate was dissolved in ethyl acetate (2 mL). The amine compound (5 mmol, 1.0 equiv.) was dissolved in ethyl acetate (1 mL) and then added dropwise to the reaction mixture at room temperature, which was stirred until the reaction was complete as monitored by TLC. The white precipitate was collected by filtration and dissolved in C_2H_5OH (3 mL). NaOH solution (10 mmol, 2.0 equiv.) was added dropwise to the reaction mixture, which was then stirred at room temperature until the reaction was complete as monitored by TLC. The reaction mixture was adjusted pH to 7 with HCl, stirred in an ice bath, and filtered to afford the products (yield: 80−85%).

Iodomethane (5 mmol, 1.0 equiv.) was added to a solution of thiourea derivative (5 mmol, 1.0 equiv.) in acetone and the mixture was continued to stir overnight at room temperature. After that, the mixture was concentrated under vacuum and ethyl acetate (25 mL) was added. Later, H_2O (50 mL) was added and cooled to 0 ^{o}C. Next, conc. NH_4OH was mixed dropwise until all the solid disappeared and then, stirred for another 30 mins. Organic layer was separated, washed with brine, and concentrated. The product was used directly without further purification (yield: 70−75%).

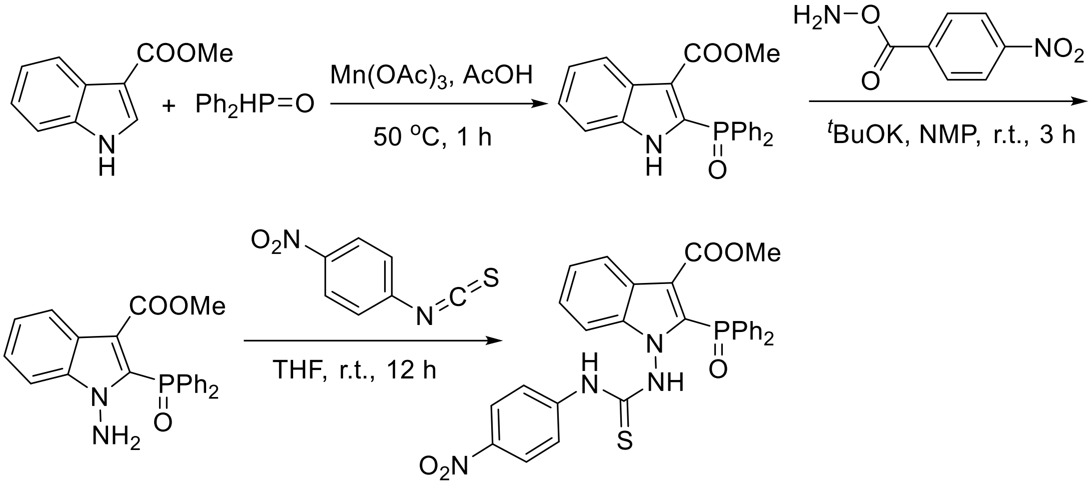

To a solution of the indole (1 mmol, 1.0 equiv.) in AcOH (3 mL), diphenylphosphine oxide (2 mmol, 2.0 equiv.) were added. The reaction mixture were heated in oil bath to 50 ℃, and manganese acetate (3 mmol, 3.0 equiv.) was added in batches within 0.5 h. After that, the reaction mixture was stirred at 50 °C for 0.5 h. After the reaction was complete as monitored by TLC, the mixture was concentrated under vacuum and H_2O (20 mL) was added and extracted with EtOAc (15 mL × 3). The combined extracts were washed with brine (15 mL), dried with Na_2SO_4, filtered, and concentrated under reduced pressure. The residue was purified by silica gel column chromatography (ethyl acetate/petroleum ether, v/v = 1:2) to give the desired product in 37% yield.

To a solution of (1H-indol-2-yl)diphenylphosphine oxide (0.37 mmol, 1.0 equiv.) in 3.7 mL NMP was added *ᵗ*BuOK (0.44 mmol, 1.2 equiv.) and the reaction mixture was stirred at r.t. for 0.5 h. Then, a solution of O-(4-nitrobenzoyl)hydroxylamine (0.44 mmol, 1.2 equiv.) in 2 mL NMP was added to the mixture, which was stirred at r.t. for 2 h. After the completion of the reaction which was indicated by TLC, the reaction mixture was quenched with H_2O and the aqueous layer was extracted with EtOAc (3×10 mL). The combined organic layers were dried over anhydrous Na_2SO_4 and then concentrated under reduced pressure. The residue was purified through flash column chromatography on silica gel (ethyl acetate/petroleum ether/dichloromethane, v/v/v = 1:1:1) to afford pure product in 50% yield as a white solid.

To a THF solution (2 mL) of the above product (0.18 mmol, 1.0 equiv.) in round-bottom flask was added isothiocyanate (0.36 mmol, 2.0 equiv.). The reaction mixture was stirred at room temperature for 12 h (complete consumption of starting material as shown by TLC). The precipitate was collected by filtration and washed with THF to afford pure product in 97% yield as a white solid.

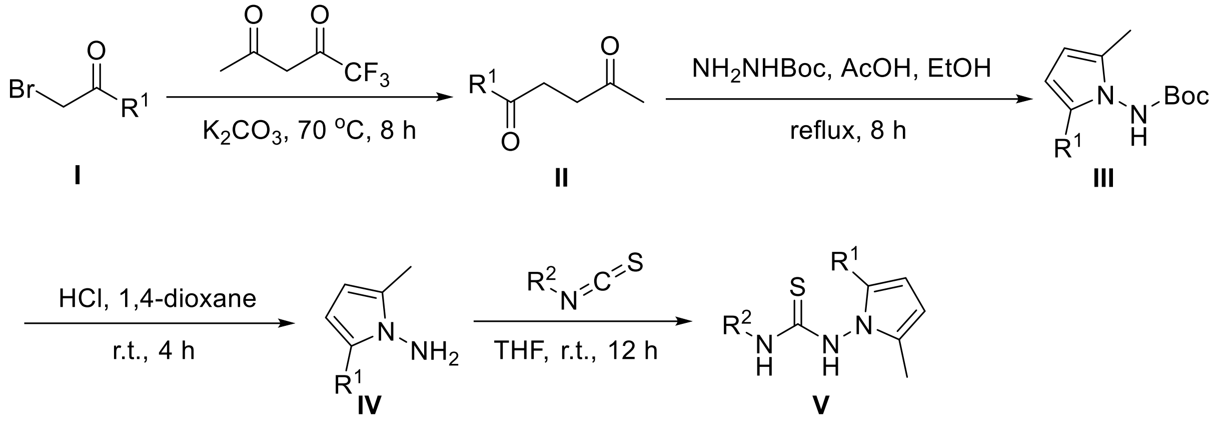

To a mixture of **I** (10.0 mmol, 2.0 equiv.), K_2CO_3 (25.0 mmol, 2.5 equiv.), 1,1,1-trifluoropentane-2,4-dione (10.0 mmol, 1.0 equiv.) in a Schlenk tube was added EtOAc (36.0 mL) and H_2O (4.0 mL). The mixture was stirred at 60 °C for 8 h. After cooling to room temperature, the reaction mixture was diluted with HCl aqueous solution (3 %, 20 mL) and extracted with EtOAc (15 mL × 3). The combined extracts were washed with brine (15 mL), dried with Na_2SO_4, filtered, and concentrated under reduced pressure. The residue was purified by silica gel column chromatography (ethyl acetate/petroleum ether, v/v = 1:20 to v/v = 1:5) to give product **II** in 75-80% yield.

A 100 mL oven-dried round-bottomed flask was charged with NH_2NHBoc (9.6 mmol, 1.2 equiv.), **II** (8 mmol, 1.0 equiv.) and EtOH-AcOH mixture (10:1). The resulting mixture was refluxed for 8 h. The reaction mixture was concentrated in *vacuo*, followed diluted by addition of 50 mL of water, then extracted with EtOAc (3 x 40 mL). The combined organic layers were washed with brine, then dried over Na_2SO_4 and evaporated in *vacuo*. The residue was purified by silica gel column chromatography (ethyl acetate/petroleum ether, v/v = 1:10 to v/v = 1:5). The desired **III** was obtained in 55-65% yield.

A solution of **III** (5.2 mmol) in 1,4-dioxane (10.4 mL) was cooled to 0 °C before the addition of 1.8 mL of conc. HCl. The reaction was stirred at room temperature for 4 h (complete consumption of starting material as shown by TLC). The crude was cooled down to 0 °C and a saturated solution $NaHCO_3$ was added until basification. The solution was extracted with dichloromethane and the organic phase washed with brine, dried over Na_2SO_4 and evaporated under reduced pressure. The residue was purified by silica gel column chromatography (ethyl acetate/petroleum ether, v/v = 1:10). The desired **IV** was obtained in 80-90% yield.

To a THF solution (30 mL) of **IV** (4.7 mmol, 1.0 equiv.) in round-bottom flask was added isothiocyanate (4.7 mmol, 1.0 equiv.). The reaction mixture was stirred at room temperature for 12 h (complete consumption of starting material as shown by TLC). The reaction mixture was concentrated in *vacuo*, The residue was purified by silica gel column chromatography (ethyl acetate/petroleum ether, v/v = 1:5). The desired **V** was obtained in 63-78% yield.

methyl (2-methyl-1H-indol-1-yl)carbamimidothioate (1a)

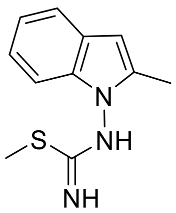

Yellow oil. **^{1}H NMR** (500 MHz, $CDCl_3$) δ 7.52 (d, J = 7.7 Hz, 1H), 7.15 – 7.02 (m, 3H), 6.27 (s, 1H), 4.66 (s, 2H), 2.61 (s, 3H), 2.32 (s, 3H). **^{13}C NMR** (126 MHz, $CDCl_3$) δ 165.2, 135.4, 133.2, 126.2, 120.2, 119.4, 119.05, 108.7, 97.6, 13.0, 11.6. **HRMS (ESI)** m/z calcd for $[C_{11}H_{13}N_3S, M+H]^+$: 220.0903; found: 220.0912.

methyl (5-fluoro-2-methyl-1H-indol-1-yl)carbamimidothioate (1n)

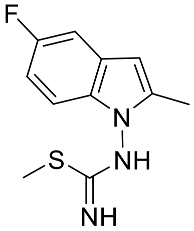

Yellow solid. **MP:** 103-105 °C. **^{1}H NMR** (500 MHz, $CDCl_3$) δ 7.15 (dd, J = 9.8, 2.5 Hz, 1H), 6.99 (dd, J = 8.8, 4.5 Hz, 1H), 6.83 (td, J = 9.1, 2.5 Hz, 1H), 6.20 (s, 1H), 4.87 (s, 2H), 2.62 (s, 3H), 2.27 (s, 3H).**^{13}C NMR** (126 MHz, $CDCl_3$) δ 165.5, 157.9 (d, J = 232.7 Hz), 137.4, 130.1, 126.7 (d, J = 10.2 Hz), 109.4 (d, J = 9.7 Hz), 108.5 (d, J = 25.9 Hz), 104.5 (d, J = 23.6 Hz), 98.0 (d, J = 3.8 Hz), 13.3, 11.9. **^{19}F NMR** (471 MHz, $CDCl_3$) δ -125.44. **HRMS (ESI)** m/z calcd for $[C_{11}H_{12}FN_3S, M+H]^+$: 238.0809; found: 238.0815.

methyl (5-chloro-2-methyl-1H-indol-1-yl)carbamimidothioate (1o)

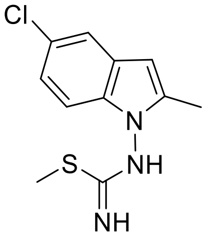

Yellow oil. **^{1}H NMR** (500 MHz, $CDCl_3$) δ 7.46 (d, J = 2.0 Hz, 1H), 7.09 – 6.94 (m, 2H), 6.18 (s, 1H), 4.84 (s, 2H), 2.62 (s, 3H), 2.27 (s, 3H). **^{13}C NMR** (126 MHz, $CDCl_3$) δ 165.5, 137.1, 131.8, 1274, 124.8, 120.6, 119.0, 109.9, 97.6, 13.3, 11.8. **HRMS (ESI)** m/z calcd for $[C_{11}H_{12}ClN_3S, M+H]^+$: 254.0513; found: 254.0521.

methyl (2,5-dimethyl-1H-indol-1-yl)carbamimidothioate (1p)

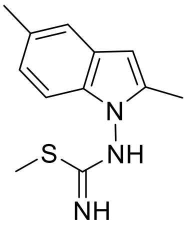

Yellow oil. **¹H NMR** (500 MHz, $CDCl_3$) δ 7.32 (s, 1H), 6.99 (d, J = 8.2 Hz, 1H), 6.94 (d, J = 9.1 Hz, 1H), 6.19 (s, 1H), 4.76 (s, 2H), 2.65 (s, 3H), 2.47 (s, 3H), 2.30 (s, 3H). **^{13}C NMR** (126 MHz, $CDCl_3$) δ 164.7, 135.7, 132.1, 128.5, 126.9, 122.0, 119.5, 108.7, 97.5, 21.3, 13.3, 11.7. **HRMS (ESI)** m/z calcd for $[C_{12}H_{15}N_3S, M+H]^+$: 234.1060; found: 234.1068.

methyl (2,3-dimethyl-1H-indol-1-yl)carbamimidothioate (1q)

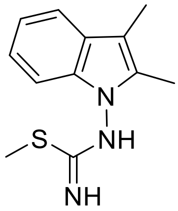

Yellow solid. **MP**: 121-123 °C. **¹H NMR** (500 MHz, $CDCl_3$) δ 7.53 (d, J = 7.6 Hz, 1H), 7.12 (dt, J = 20.3, 7.4 Hz, 3H), 4.88 (s, 2H), 2.64 (s, 3H), 2.32 (s, 3H), 2.25 (s, 3H).**^{13}C NMR** (126 MHz, $CDCl_3$) δ 164.7, 133.0, 131.9, 127.0, 120.4, 118.6, 117.7, 108.7, 105.1, 13.2, 9.4, 8.5. **HRMS (ESI)** m/z calcd for $[C_{12}H_{15}N_3S, M+H]^+$: 234.1060; found: 234.1066.

methyl (1,2,3,4-tetrahydro-9H-carbazol-9-yl)carbamimidothioate (1r)

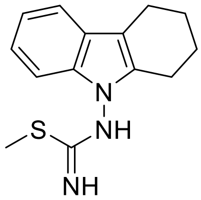

White solid. **MP**: 101-103 °C. **¹H NMR** (500 MHz, $CDCl_3$) δ 7.48 (d, J = 7.7 Hz, 1H), 7.15 – 7.01 (m, 3H), 4.83 (s, 2H), 2.77 (t, J = 5.7 Hz, 2H), 2.63 (t, J = 5.7 Hz, 5H), 1.91 (qd, J = 7.2, 3.1 Hz, 4H). **^{13}C NMR** (126 MHz, $CDCl_3$) δ 164.4, 135.3, 133.9, 126.2, 120.7, 118.9, 117.7, 109.0, 108.6, 23.4, 23.1, 21.8, 21.1, 13.3. **HRMS (ESI)** m/z calcd for $[C_{14}H_{17}N_3S, M+H]^+$: 260.1216; found: 260.1222.

methyl (2-methyl-5-phenyl-1H-pyrrol-1-yl)carbamimidothioate (1s)

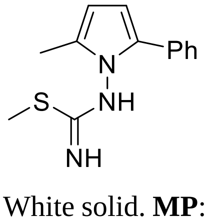

White solid. **MP**: 123-129 °C. **¹H NMR** (500 MHz, $CDCl_3$) δ 7.52 (dt, J = 8.2, 1.6 Hz, 2H), 7.27 (t, J = 7.8 Hz, 2H), 7.17 – 7.08 (m, 1H), 6.28 (d, J = 3.8 Hz, 1H), 5.95 (d, J = 3.8 Hz, 1H), 4.56 (s, 2H), 2.55 (s, 3H),

2.12 (s, 3H). **^{13}C NMR** (126 MHz, $CDCl_3$) δ 164.7, 133.1, 128.9, 128.1, 127.5, 126.2, 125.6, 105.7, 105.1, 13.2, 11.3. **HRMS (ESI)** m/z calcd for $[C_{13}H_{15}N_3S, M+H]^+$: 246.1060; found: 246.1064.

1-(2-methyl-5-phenyl-1H-pyrrol-1-yl)-3-(4-nitrophenyl)thiourea (5a)

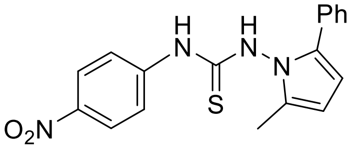

Yellow solid. **MP**: 175-177 °C. **^{1}H NMR** (500 MHz, $CDCl_3$) δ 8.93 (s, 1H), 8.25 – 7.94 (m, 2H), 7.59 – 7.51 (m, 2H), 7.48 – 7.43 (m, 2H), 7.43 – 7.36 (m, 2H), 7.35 – 7.19 (m, 2H), 6.40 (d, *J* = 3.9 Hz, 1H), 6.11 (dd, *J* = 3.9, 1.1 Hz, 1H), 2.30 (d, *J* = 1.0 Hz, 3H). **^{13}C NMR** (126 MHz, $CDCl_3$) δ 180.6, 145.1, 142.8, 132.9, 131.2, 130.3, 129.0, 127.7, 126.9, 124.4, 123.7, 108.2, 107.7, 11.4. **HRMS (ESI)** m/z calcd for $[C_{18}H_{16}N_4O_2S, M+H]^+$: 353.1067; found: 353.1066.

1-(2-methyl-5-phenyl-1H-pyrrol-1-yl)-3-(4-(trifluoromethyl)phenyl)thiourea (5b)

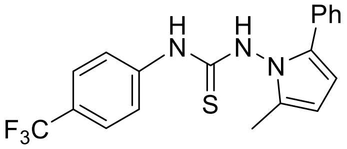

Yellow solid. **MP**: 175-177 °C. **^{1}H NMR** (500 MHz, $CDCl_3$) δ 9.40 (s, 1H), 7.51 (dd, J = 21.3, 8.1 Hz, 4H), 7.43 – 7.34 (m, 4H), 7.29 (t, J = 7.4 Hz, 1H), 7.14 (s, 1H), 6.37 (d, J = 3.9 Hz, 1H), 6.08 (d, J = 3.9 Hz, 1H), 2.29 (s, 3H).**^{13}C NMR** (126 MHz, $CDCl_3$) δ 181.0, 140.0, 132.7, 131.1, 130.5, 128.9, 128.5 (d, J = 33.0 Hz), 127.5, 126.9, 126.0 – 125.8 (m), 123.7 (d, J = 272.1 Hz), 124.6, 108.0, 107.4, 11.39. **^{19}F NMR** (471 MHz, $CDCl_3$) δ -62.45. **HRMS (ESI)** m/z calcd for $[C_{19}H_{16}F_3N_3S, M+Na]^+$: 398.0909; found: 398.0924.

1-(3,5-bis(trifluoromethyl)phenyl)-3-(2-methyl-5-phenyl-1H-pyrrol-1-yl)thiourea (5c)

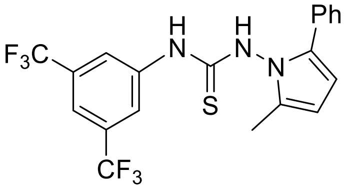

Yellow solid. **MP**: 170-172 °C. **^{1}H NMR** (500 MHz, $CDCl_3$) δ 9.34 (s, 1H), 7.69 (d, J = 8.5 Hz, 3H), 7.49 (ddd, J = 7.2, 3.7, 2.0 Hz, 2H), 7.40 (t, J = 7.7 Hz, 2H), 7.32 (t, J = 7.4 Hz, 1H), 7.20 (s, 1H), 6.40 (d, J = 3.9 Hz, 1H), 6.12 (dd, J = 3.9, 1.1 Hz, 1H), 2.32 (s, 3H). **^{13}C NMR** (126 MHz, $CDCl_3$) δ 181.4, 138.5, 132.6, 132.1 (d, J = 34.0 Hz), 132.1 (d, J = 101.9 Hz), 130.7 (d, J = 93.1 Hz), 129.0, 127.8, 126.9, 125.0, 124.9 (d, J = 272.8 Hz), 120.6 (d, J = 272.6 Hz). 120.2, 108.2, 107.6, 11.5. **^{19}F NMR** (471 MHz, $CDCl_3$) δ -62.98. **HRMS (ESI)** m/z calcd for $[C_{20}H_{15}F_6N_3S, M+Na]^+$: 466.0783; found: 466.0792.

1-(2-methyl-5-(p-tolyl)-1H-pyrrol-1-yl)-3-(4-nitrophenyl)thiourea (5d)

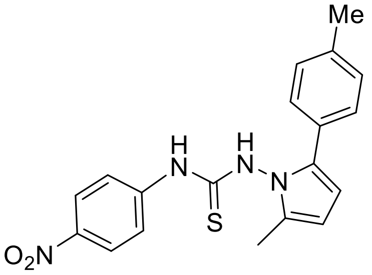

Syrup. **^{1}H NMR** (500 MHz, $CDCl_3$) δ 9.49 (s, 1H), 8.09 – 8.05 (m, 2H), 7.57 – 7.50 (m, 2H), 7.44 – 7.38 (m, 1H), 7.38 – 7.32 (m, 2H), 7.15 (d, *J* = 8.0 Hz, 2H), 6.30 (d, *J* = 3.9 Hz, 1H), 6.07 – 6.02 (m, 1H), 2.31 (s, 3H), 2.26 (s, 3H). **^{13}C NMR** (126 MHz, $CDCl_3$) δ 180.5, 144.8, 142.8, 137.2, 132.8, 130.7, 129.5, 127.4, 126.6, 124.1, 123.7, 107.5, 107.2, 21.0, 11.2. **HRMS (ESI)** m/z calcd for $[C_{19}H_{18}N_4O_2S, M+Na]^+$: 389.1042; found: 389.1055.

1-(2-(4-methoxyphenyl)-5-methyl-1H-pyrrol-1-yl)-3-(4-nitrophenyl)thiourea (5e)

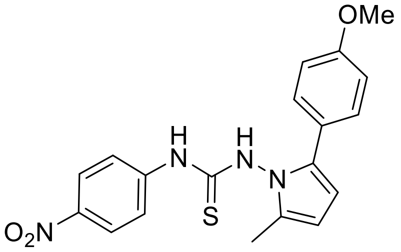

Yellow solid. **MP**: 175-177 °C. **^{1}H NMR** (500 MHz, $CDCl_3$) δ 8.74 (s, 1H), 8.20 – 8.08 (m, 2H), 7.63 – 7.54 (m, 2H), 7.41 – 7.33 (m, 2H), 7.26 (s, 1H), 6.98 – 6.89 (m, 2H), 6.30 (d, J = 3.9 Hz, 1H), 6.09 (dd, J = 3.9, 1.1 Hz, 1H), 3.81 (s, 3H), 2.28 (d, J = 1.1 Hz, 3H). **^{13}C NMR** (126 MHz, $CDCl_3$) δ 180.6, 159.2, 145.0, 142.8, 132.8, 130.4, 128.3, 124.4, 123.6, 122.8, 114.4, 107.5, 107.3, 55.3, 11.4. **HRMS (ESI)** m/z calcd for $[C_{19}H_{18}N_4O_3S, M+Na]^+$: 405.0992; found: 405.1008.

1-(2-(4-fluorophenyl)-5-methyl-1H-pyrrol-1-yl)-3-(4-nitrophenyl)thiourea (5f)

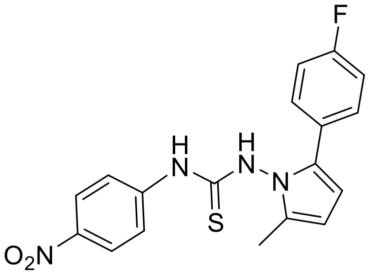

Yellow solid. **MP**: 175-177 °C. **^{1}H NMR** (500 MHz, $CDCl_3$) δ 9.13 (s, 1H), 8.18 – 8.14 (m, 2H), 7.61 – 7.55 (m, 2H), 7.46 – 7.39 (m, 2H), 7.28 (s, 1H), 7.07 (t, *J* = 8.6 Hz, 2H), 6.34 (d, *J* = 3.9 Hz, 1H), 6.10 (d, *J* = 3.9 Hz, 1H), 2.29 (s, 3H). **^{13}C NMR** (126 MHz, $CDCl_3$) δ 180.6, 162.2 (d, J = 248.2 Hz), 145.1, 142.7, 132.0, 131.1, 128.7 (d, J = 8.2 Hz), 126.5, 124.4, 123.7, 116.0 (d, J = 21.9 Hz), 108.1, 107.6, 11.4. **^{19}F NMR** (471 MHz, $CDCl_3$) δ -113.53. **HRMS (ESI)** m/z calcd for $[C_{18}H_{15}FN_4O_2S, M+Na]^+$: 393.0792; found: 393.0798.

1-(2-(4-chlorophenyl)-5-methyl-1H-pyrrol-1-yl)-3-(4-nitrophenyl)thiourea (5g)

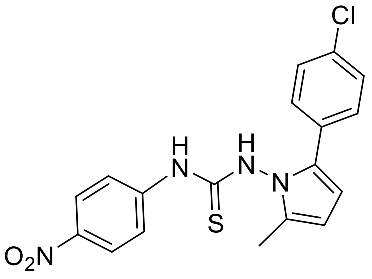

Yellow solid. **MP**: 175-177 °C. **^{1}H NMR** (500 MHz, $CDCl_3$) δ 8.80 (s, 1H), 8.20 – 8.13 (m, 2H), 7.60 (d, *J* = 9.1 Hz, 2H), 7.42 – 7.34 (m, 4H), 7.26 (s, 1H), 6.40 (d, *J* = 4.0 Hz, 1H), 6.13 (dd, *J* = 3.9, 1.1 Hz, 1H), 2.30 (d, *J* = 1.0 Hz, 3H). **^{13}C NMR** (126 MHz, $CDCl_3$) δ 180.5, 145.2, 142.7, 133.6, 131.8, 131.6, 129.2, 128.7, 127.9, 124.5, 123.6, 108.6, 107.9, 11.4. **HRMS (ESI)** m/z calcd for [$C_{18}H_{15}ClN_4O_2S$, M+Na]$^+$: 409.0496; found: 409.0507.

1-(2-(4-bromophenyl)-5-methyl-1H-pyrrol-1-yl)-3-(4-nitrophenyl)thiourea (5h)

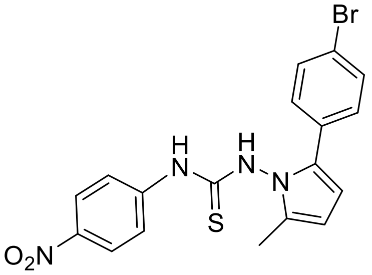

Yellow solid. **MP**: 175-177 °C. **^{1}H NMR** (500 MHz, $CDCl_3$) δ 9.32 (s, 1H), 8.14 (d, *J* = 8.7 Hz, 2H), 7.58 (d, *J* = 8.8 Hz, 2H), 7.46 (d, *J* = 2.1 Hz, 1H), 7.39 – 7.16 (m, 4H), 6.40 (d, *J* = 4.0 Hz, 1H), 6.11 (d, *J* = 3.9 Hz, 1H), 2.29 (s, 3H). **^{13}C NMR** (126 MHz, $CDCl_3$) δ 180.6, 145.1, 142.7, 134.7, 132.0, 131.4, 130.2, 127.5, 126.9, 124.4, 123.9, 109.0, 107.8, 11.4. **HRMS (ESI)** m/z calcd for [$C_{18}H_{15}BrN_4O_2S$, M+Na]$^+$: 452.9991; found: 452.9987.

1-(2-methyl-5-(4-(trifluoromethyl)phenyl)-1H-pyrrol-1-yl)-3-(4-nitrophenyl)thiourea (5i)

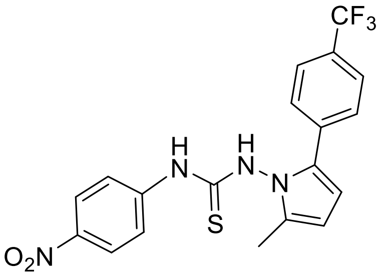

Yellow solid. **MP**: 175-177 °C. **^{1}H NMR** (500 MHz, $CDCl_3$) δ 9.38 (s, 1H), 8.24 – 8.04 (m, 2H), 7.73 – 7.55 (m, 6H), 7.27 (d, *J* = 11.0 Hz, 1H), 6.50 (d, *J* = 4.0 Hz, 1H), 6.15 (d, *J* = 3.9 Hz, 1H), 2.31 (s, 3H). **^{13}C NMR** (126 MHz, $CDCl_3$) δ 180.6, 145.2, 142.5, 133.7, 132.5, 131.3, 129.2 (d, J = 33.0 Hz). 126.5, 126.0 – 125.8 (m), 124.1 (d, J = 75.9 Hz), 124.0 (d, J = 272.1 Hz), 109.7, 108.2, 11.4. **^{19}F NMR** (471 MHz, $CDCl_3$) δ -62.57. **HRMS (ESI)** m/z calcd for [$C_{19}H_{15}F_3N_4O_2S$, M+H]$^+$: 421.0941; found: 421.0948.

1-(2-(4-cyanophenyl)-5-methyl-1H-pyrrol-1-yl)-3-(4-nitrophenyl)thiourea (5j)

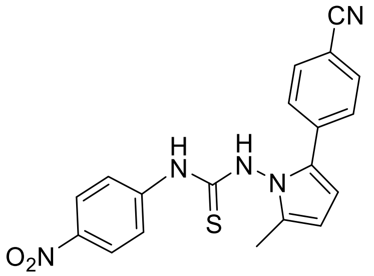

Syrup. **^{1}H NMR** (500 MHz, $CDCl_3$) δ 9.88 – 9.36 (m, 1H), 8.19 – 7.86 (m, 2H), 7.59 (t, *J* = 8.5 Hz, 7H), 6.52 (s, 1H), 6.14 (s, 1H), 2.32 (s, 3H). **^{13}C NMR** (126 MHz, $CDCl_3$) δ 180.4, 144.8, 142.6, 134.7, 133.5, 133.4, 132.5, 130.5, 126.2, 124.1, 118.6, 110.4, 109.5, 108.3, 11.2. **HRMS (ESI)** m/z calcd for $[C_{19}H_{15}N_5O_2S, M+Na]^+$: 400.0838; found: 400.0838.

1-(2-(3-methoxyphenyl)-5-methyl-1H-pyrrol-1-yl)-3-(4-nitrophenyl)thiourea (5k)

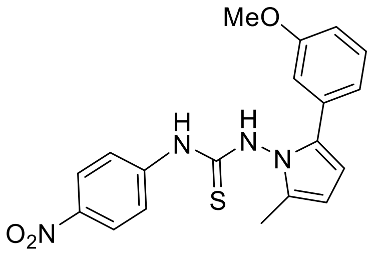

Yellow solid. **MP**: 155-157 °C. **^{1}H NMR** (500 MHz, $CDCl_3$) δ 9.45 (s, 1H), 8.09 (dt, *J* = 9.1, 2.0 Hz, 2H), 7.59 – 7.50 (m, 2H), 7.47 – 7.36 (m, 1H), 7.31 – 7.17 (m, 1H), 7.09 – 6.97 (m, 2H), 6.81 (dd, *J* = 8.3, 2.5 Hz, 1H), 6.37 (d, *J* = 3.9 Hz, 1H), 6.11 – 6.02 (m, 1H), 3.76 (s, 3H), 2.27 (d, *J* = 1.1 Hz, 3H). **^{13}C NMR** (126 MHz, $CDCl_3$) δ 180.5, 159.7, 144.9, 142.8, 132.5, 131.5, 131.3, 129.8, 124.2, 123.8, 119.2, 113.2, 112.1, 108.3, 107.4, 55.3, 11.3. **HRMS (ESI)** m/z calcd for $[C_{19}H_{18}N_4O_3S, M+Na]^+$: 405.0992; found: 405.1010.

1-(2-(3-chlorophenyl)-5-methyl-1H-pyrrol-1-yl)-3-(4-nitrophenyl)thiourea (5l)

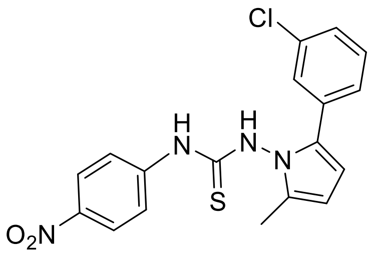

Yellow solid. **MP**: 155-157 °C. **^{1}H NMR** (500 MHz, $CDCl_3$) δ 9.42 (s, 1H), 8.13 (d, J = 8.8 Hz, 2H), 7.57 (d, J = 8.8 Hz, 2H), 7.48 (d, J = 8.2 Hz, 2H), 7.34 (d, J = 8.3 Hz, 2H), 7.26 (s, 1H), 6.38 (d, J = 3.9 Hz, 1H), 6.10 (d, J = 3.8 Hz, 1H), 2.29 (s, 3H). **^{13}C NMR** (126 MHz, $CDCl_3$) δ 180.5, 145.1, 142.6, 132.0, 131.7, 131.6, 129.2, 128.1, 124.4, 123.8, 121.5, 108.5, 107.8, 11.4. **HRMS (ESI)** m/z calcd for $[C_{18}H_{15}ClN_4O_2S, M+Na]^+$: 409.0496; found: 409.0498.

1-(2-(3-bromophenyl)-5-methyl-1H-pyrrol-1-yl)-3-(4-nitrophenyl)thiourea (5m)

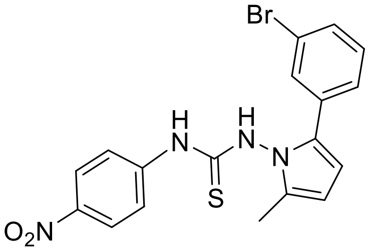

Yellow solid. **MP**: 166-168 °C. **^{1}H NMR** (500 MHz, $CDCl_3$) δ 9.06 (d, J = 26.9 Hz, 1H), 8.20 – 8.12 (m, 2H), 7.66 – 7.53 (m, 3H), 7.40 (t, J = 7.9 Hz, 2H), 7.30 – 7.21 (m, 2H), 6.41 (d, J = 3.9 Hz, 1H), 6.12 (d, J = 3.9 Hz, 1H), 2.30 (s, 3H). **^{13}C NMR** (126 MHz, $CDCl_3$) δ 180.7, 145.2, 142.7, 132.3, 132.3, 132.0, 131.4, 130.5, 129.9, 125.0, 124.5, 123.8, 123.0, 109.1, 107.9, 11.4. **HRMS (ESI)** m/z calcd for [$C_{18}H_{15}BrN_4O_2S$, M+H]$^+$: 431.0172; found: 431.0170.

1-(2-(2-fluorophenyl)-5-methyl-1H-pyrrol-1-yl)-3-(4-nitrophenyl)thiourea (5n)

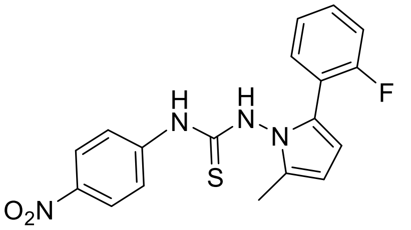

Yellow solid. **MP**: 152-154 °C. **^{1}H NMR** (500 MHz, $CDCl_3$) δ 8.97 (s, 1H), 8.16 – 8.10 (m, 2H), 7.62 – 7.56 (m, 2H), 7.39 – 7.29 (m, 2H), 7.28 – 7.22 (m, 1H), 7.19 – 7.11 (m, 2H), 6.35 (d, *J* = 3.9 Hz, 1H), 6.12 (d, *J* = 3.9 Hz, 1H), 2.28 (s, 3H). **^{13}C NMR** (126 MHz, $CDCl_3$) δ 180.7, 159.6 (d, *J* = 246.3 Hz), 144.9, 142.9, 131.4, 131.0, 130.0 (d, *J* = 8.5 Hz), 127.0, 124.5 (d, *J* = 3.1 Hz), 124.3, 123.5, 118.4 (d, *J* = 13.9 Hz), 116.0 (d, *J* = 22.7 Hz), 110.1, 107.6, 11.3. **^{19}F NMR** (471 MHz, $CDCl_3$) δ -114.68. **HRMS (ESI)** m/z calcd for [$C_{18}H_{15}FN_4O_2S$, M+Na]$^+$: 393.0792; found: 393.0797.

1-(2-methyl-5-(naphthalen-2-yl)-1H-pyrrol-1-yl)-3-(4-nitrophenyl)thiourea (5o)

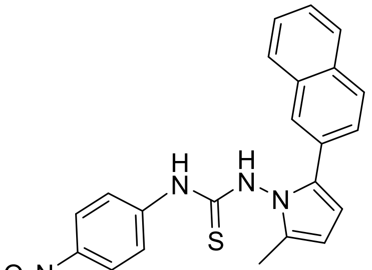

Yellow solid. **MP**: 152-154 °C. **^{1}H NMR** (500 MHz, $CDCl_3$) δ 9.32 (d, J = 10.3 Hz, 1H), 8.05 (d, J = 8.7 Hz, 2H), 7.92 (s, 1H), 7.82 – 7.68 (m, 3H), 7.57 (dd, J = 8.6, 1.9 Hz, 1H), 7.51 (d, J = 8.7 Hz, 2H), 7.47 – 7.38 (m, 3H), 6.49 (d, J = 4.0 Hz, 1H), 6.12 (d, J = 3.9 Hz, 1H), 2.28 (s, 3H). **^{13}C NMR** (126 MHz, $CDCl_3$) δ 180.7, 144.9, 142.8, 133.4, 132.8, 132.3, 131.6, 128.6, 128.1, 127.5, 126.5, 126.2, 125.0, 124.8, 124.3, 123.8, 108.7, 107.7, 11.4. **HRMS (ESI)** m/z calcd for [$C_{22}H_{18}N_4O_2S$, M+Na]$^+$: 425.1042; found: 425.1038.

1-(2-methyl-1H-indol-1-yl)-3-(4-nitrophenyl)thiourea (5ae)

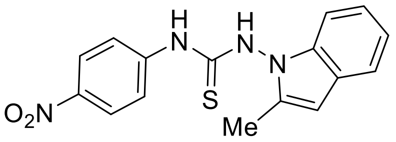

Yellow solid. **MP**: 176-178 °C. **¹H NMR** (500 MHz, $CDCl_3$) δ 8.45 (s, 1H), 8.19 – 8.10 (m, 2H), 7.71 – 7.64 (m, 3H), 7.55 (d, *J* = 7.6 Hz, 1H), 7.35 (d, *J* = 7.9 Hz, 1H), 7.27 – 7.13 (m, 2H), 6.40 (s, 1H), 2.42 (s, 3H). **¹³C NMR** (126 MHz, $CDCl_3$) δ 181.2, 145.1, 142.8, 136.8, 136.3, 126.7, 124.4, 123.8, 123.2, 122.4, 120.7, 108.7, 102.1, 11.6. **HRMS (ESI)** m/z calcd for [$C_{16}H_{14}N_4O_2S$, M+H]$^+$: 327.0910; found: 327.0904.

1-(4-nitrophenyl)-3-(2-phenyl-1H-indol-1-yl)thiourea (5af)

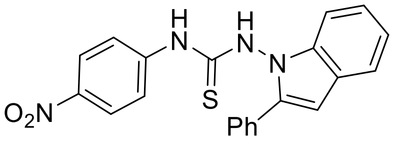

Yellow solid. **MP**: 170-172 °C. **¹H NMR** (500 MHz, $CDCl_3$) δ 8.57 (s, 1H), 8.11 (d, *J* = 9.1 Hz, 2H), 7.85 (s, 1H), 7.65 (d, *J* = 7.6 Hz, 1H), 7.60 – 7.55 (m, 4H), 7.47 (dd, *J* = 8.3, 6.4 Hz, 2H), 7.42 (dd, *J* = 7.7, 1.5 Hz, 2H), 7.36 – 7.32 (m, 1H), 7.30 – 7.25 (m, 1H), 6.81 (s, 1H). **¹³C NMR** (126 MHz, $CDCl_3$) δ 180.8, 145.1, 142.8, 140.3, 137.7, 129.5, 129.2, 129.0, 127.8, 126.6, 124.4, 124.3, 123.9, 123.0, 121.5, 109.5, 103.7. **HRMS (ESI)** m/z calcd for [$C_{21}H_{16}N_4O_2S$, M+H]$^+$: 389.1067; found: 389.1038.

methyl 2-(diphenylphosphoryl)-1-(3-(4-nitrophenyl)thioureido)-1H-indole-3-carboxylate (5ag)

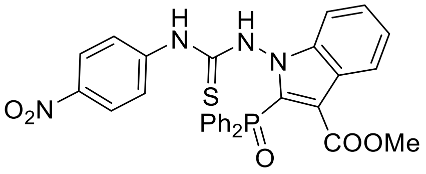

White solid. **MP**: 213-214 °C. **¹H NMR** (500 MHz, DMSO-*d*₆) δ 11.06 (s, 1H), 8.31 (s, 1H), 8.19 (d, *J* = 8.7 Hz, 2H), 7.98 (d, *J* = 8.1 Hz, 1H), 7.89 – 7.71 (m, 5H), 7.65 – 7.30 (m, 10H), 3.21 (s, 3H). **¹³C NMR** (126 MHz, DMSO-d_6) δ 187.0, 168.5, 150.4, 148.6, 143.8, 139.5, 138.7, 137.1, 137.0, 136.4, 136.3, 133.59, 133.5, 133.4, 133.3, 131.0, 129.2, 128.2, 126.7, 116.0, 56.1. **³¹P NMR** (202 MHz, DMSO-d_6) δ 25.78. **HRMS (ESI)** m/z calcd for $C_{29}H_{23}N_4O_5PS$, M+H]$^+$: 571.1200; found: 571.1208.

1-(2-methyl-5-phenyl-1H-pyrrol-1-yl)-3-(2-(trifluoromethyl)phenyl)thiourea (5ah)

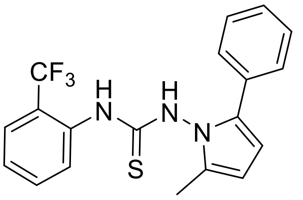

Yellow solid. **MP**: 175-177 °C. **¹H NMR** (500 MHz, $CDCl_3$) δ 9.35 (s, 1H), 7.59 (d, J = 7.8 Hz, 2H), 7.53 – 7.47 (m, 3H), 7.39 (dd, J = 8.5, 7.0 Hz, 2H), 7.31 (dq, J = 9.4, 7.6 Hz, 2H), 7.19 – 7.07 (m, 1H), 6.34 (d, J = 3.9 Hz, 1H), 6.06 (d, J = 3.8 Hz, 1H), 2.32 (s, 3H). **¹³C NMR** (126 MHz, $CDCl_3$) δ 182.3, 135.1, 132.5, 131.6 (d, J = 151.7 Hz), 130.6, 129.9, 128.7, 127.2, 127.0, 126.4, 126.2, 125.3 (d, J = 29.1 Hz), 124.2,

122.1, 107.9, 107.2, 11.1. **^{19}F NMR** (471 MHz, $CDCl_3$) δ -61.13. **HRMS (ESI)** m/z calcd for $[C_{19}H_{16}F_3N_3S, M+Na]^+$: 398.0909; found: 398.0915.

1-(2-(4-bromophenyl)-5-methyl-1H-pyrrol-1-yl)-3-(2-(trifluoromethyl)phenyl)thiourea (5ai)

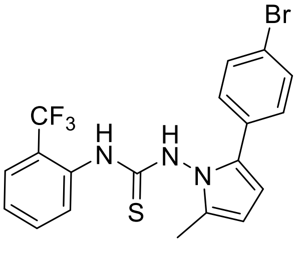

White solid. **MP**: 182-184 °C. **^{1}H NMR** (500 MHz, $CDCl_3$) δ 9.55 (s, 1H), 7.61 (dd, *J* = 14.9, 8.0 Hz, 2H), 7.51 (dd, *J* = 8.0, 5.9 Hz, 3H), 7.43 – 7.32 (m, 3H), 7.11 (d, *J* = 3.9 Hz, 1H), 6.35 (d, *J* = 4.0 Hz, 1H), 6.07 (d, *J* = 3.9 Hz, 1H), 2.31 (s, 3H). **^{13}C NMR** (126 MHz, $CDCl_3$) δ 182.1, 134.9, 132.3, 131.8, 131.42 (d, J = 21.2 Hz). 129.9, 129.5, 128.3, 127.4, 126.4 – 126.1 (m), 125.2 (d, J = 30.3 Hz), 124.2, 122.1, 121.2, 108.3, 107.5, 11.1. **^{19}F NMR** (471 MHz, $CDCl_3$) δ -61.09. **HRMS (ESI)** m/z calcd for $C_{19}H_{15}BrF_3N_3S$, $M+H]^+$: 454.0195; found: 454.0211.

1-(2,5-dimethyl-1H-pyrrol-1-yl)-3-(2-(trifluoromethyl)phenyl)thiourea (5aj)

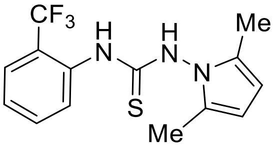

White solid. **MP**: 183-184 °C. **^{1}H NMR** (500 MHz, $CDCl_3$) δ 8.69 (d, *J* = 19.9 Hz, 1H), 7.92 (d, *J* = 8.2 Hz, 1H), 7.67 – 7.53 (m, 2H), 7.38 (t, *J* = 7.8 Hz, 1H), 7.20 (s, 1H), 5.87 (s, 2H), 2.22 (s, 6H). **^{13}C NMR** (126 MHz, $CDCl_3$) δ 182.7, 135.2, 132.3, 130.1, 127.9, 127.3, 126.3 (d, *J* = 5.1 Hz), 125.1 (d, *J* = 29.6 Hz), 123.3 (d, *J* = 273.8 Hz), 105.9, 11.0. **^{19}F NMR** (471 MHz, $CDCl_3$) δ -61.12. **HRMS (ESI)** m/z calcd for $[C_{14}H_{14}F_3N_3S, M+H]^+$: 314.0934; found: 314.0928.

3. General procedure for the synthesis of N–N axially chiral pyrrole/indole-based 5,6-dihydropyrimidin-4-ones

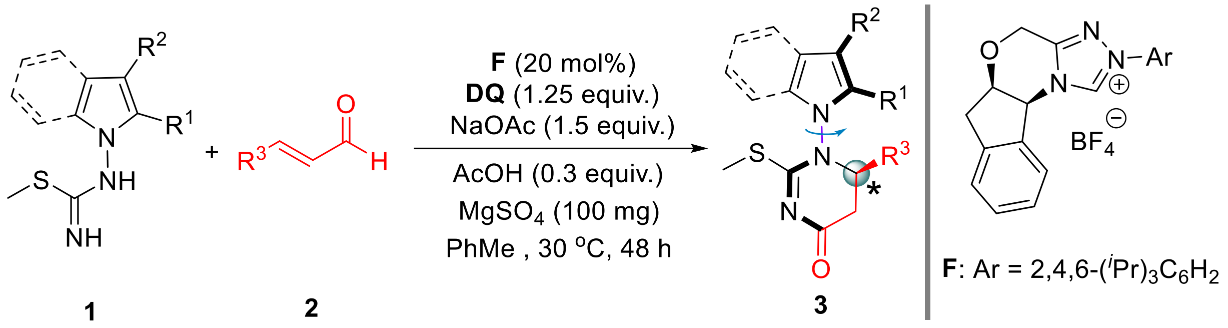

A dry 4 mL vial with a stir bar was charged with isothioureas **1** (0.1 mmol,1.0 equiv.), PreNHC **F** (20 mol%), NaOAc (0.15 mmol, 1.5 equiv.), 3,3',5,5'-Tetra-tert-butyl-4,4'-dibenzoquinone **(DQ)** (0.125 mmol, 1.25 equiv.) and $MgSO_4$ (100 mg). The mixture was taken into the glovebox, where AcOH (0.03 mmol, 0.3 equiv.), enals **2** (0.18 mmol, 1.8 equiv.) and toluene (2.0 mL) were added. The reaction mixture was taken outside the glovebox. The vial was then sealed and the mixture was allowed to stir in the fume hood at 30 ^{o}C for 48 h. When the substrate was consumed completely, the mixture was concentrated under vacuum and purified by column chromatography on silica gel (hexane/ethyl acetate = 2:1) to afford the pure products **3**.

4. Supplementary Table 1. Optimization of the Reaction Conditions.[a]

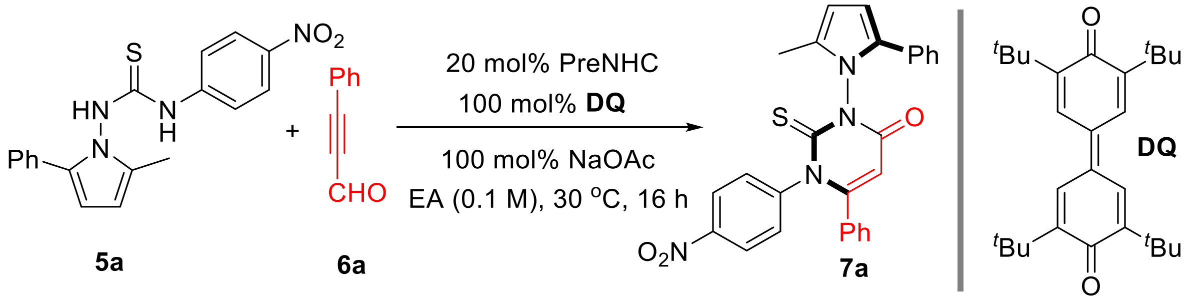

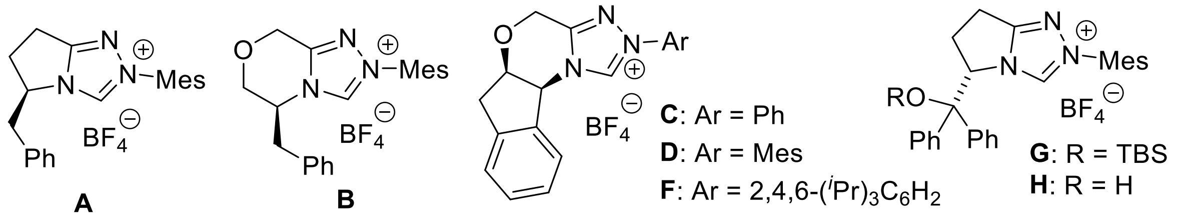

Entry	Deviation from standard conditions	**7a** Yield (%)[b]	**7a** ee (%)[c]
1	**none**	55(51)[d]	97
2	**A** instead of **H**	28	-56
3	**B** instead of **H**	33	-70
4	**C** instead of **H**	34	-54
5	**D** instead of **H**	40(36)[d]	-77
6	**F** instead of **H**	42	-16
7	**G** instead of **H**	trace	/
8	DBU instead of NaOAc	trace	n.d.
9	DIPEA instead of NaOAc	33	85
10	KO^{t}Bu instead of NaOAc	39	84
11	CsOAc instead of NaOAc	32	94
12	KOAc instead of NaOAc	43	94
13	toluene instead of EA	complex	n.d.
14	DCM instead of EA	43	74
15	THF instead of EA	36	80
16	10 mol% instead of 20 mol% **H**	5	81

[a]Unless other specified, the reactions were performed with **5a** (0.05 mmol, 1.0 equiv.), **6a** (0.06 mmol, 1.2 equiv.), PreNHC (20 mol%), **DQ** (100 mol%) and base (100 mol%) in solvent (0.5 mL) at 30 °C for 16 h. [b]Determined by ^{1}H NMR using 1,1,2,2-tetrachloroethane as the internal standard. [c]Determined by chiral HPLC analysis. [d]Isolated yield. Mes = 2,4,6-trimethylphenyl, TBS = *tert*-butyldimethylsilyl, DQ = 3,3',5,5'-Tetra-tert-butyl-4,4'-dibenzoquinone.

After a brief survey of NHC precatalysts, bases, and solvents, we were delighted to find that the reaction of **5a** with **6a** in the presence of the oxidant **DQ**, and the PreNHC **H** using NaOAc as the base at 30 °C in ethyl acetate resulted in the enantioselective synthesis of the N–N axially chiral pyrrole-based 2,3-dihydropyrimidin-4-ones **7a** in 51% isolated yield and excellent 97% ee (Supplementary Table 1, entry 1). The PreNHC optimization studies revealed that **A**, **B**, **C**, **D**, and **F** could give the desired products (**7a**) in lower yields with worse enantioselectivities (Supplementary Table 1, entries 2-6). No target product could be observed when using the PreNHC **G** with OTBS group (Supplementary Table 1, entry 7). The base optimization studies revealed that base such as DBU could not furnish the desired product (Supplementary Table 1, entry 8), whereas DIPEA, KO^{t}Bu CsOAc and KOAc could afford the desired product in reduced selectivity and yield as compared to NaOAc (Table S1, entries 9−12). An extensive solvent screening revealed that solvent toluene resulted in complex reaction in this catalytic system (Supplementary Table 1, entry 13), whereas DCM and THF resulted in reduced selectivity and yields (Supplementary Table 1, entries 14 and 15). At last, the amount of the PreNHC **H** was tested, and the result showed that using 10 mol% **H** afford product **7a** with 5% yield (Supplementary Table 1, entry 16).

5. General procedure for the synthesis of N–N axially chiral pyrrole-based 2,3-dihydropyrimidin-4-ones

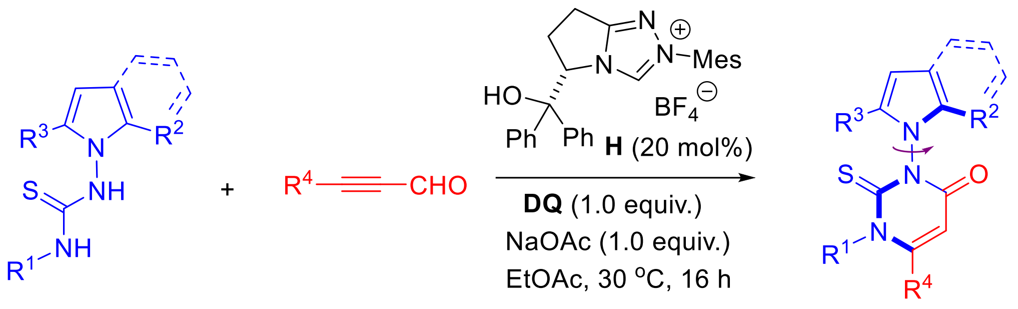

5 **6** **7**

To a 4 mL vial was added the thioureas **5** (0.2 mmol, 1.0 equiv.), PreNHC **H** (0.04 mmol, 20 mol%), 3,3',5,5'-Tetra-tert-butyl-4,4'-dibenzoquinone (**DQ**) (0.2 mmol, 1.0 equiv.) and NaOAc (0.2 mmol, 1.0 equiv.). The mixture was taken into the glovebox, where ynals **6** (0.24 mmol, 1.2 equiv.) and EtOAc (2.0 mL) were added. The reaction mixture was taken outside the glovebox. The vial was then sealed and the mixture was allowed to stir in the fume hood at 30 °C for 16 h. The crude reaction mixture was directly purified by silica gel column chromatography with hexanes/ethyl acetate (5:1 v/v) as eluent to afford the pure products **7**.

The proposed reaction mechanism is depicted in the Supplementary Figure 1. The free NHC catalyst is generated in situ from the pre-catalyst **H** under basic conditions and reacts with the ynal **6a** to give an NHC-bounded Breslow intermediate, which then undergoes oxidation to generate the alkynyl acylazolium intermediate **I** and reacts with the deprotonated nucleophilic substrate **5a'** through aza-Michael addition with nitrogen nucleophile to form the allenolate intermediate **II**, which can lead to the new acylazolium intermediate **III** through a proton transfer process. Next a second N–C bond is formed to create **IV** and the NHC catalyst can be released and finally affords the N–N axially chiral pyrrole bearing a 2,3-dihydropyrimidin-4-one moiety **7a**.

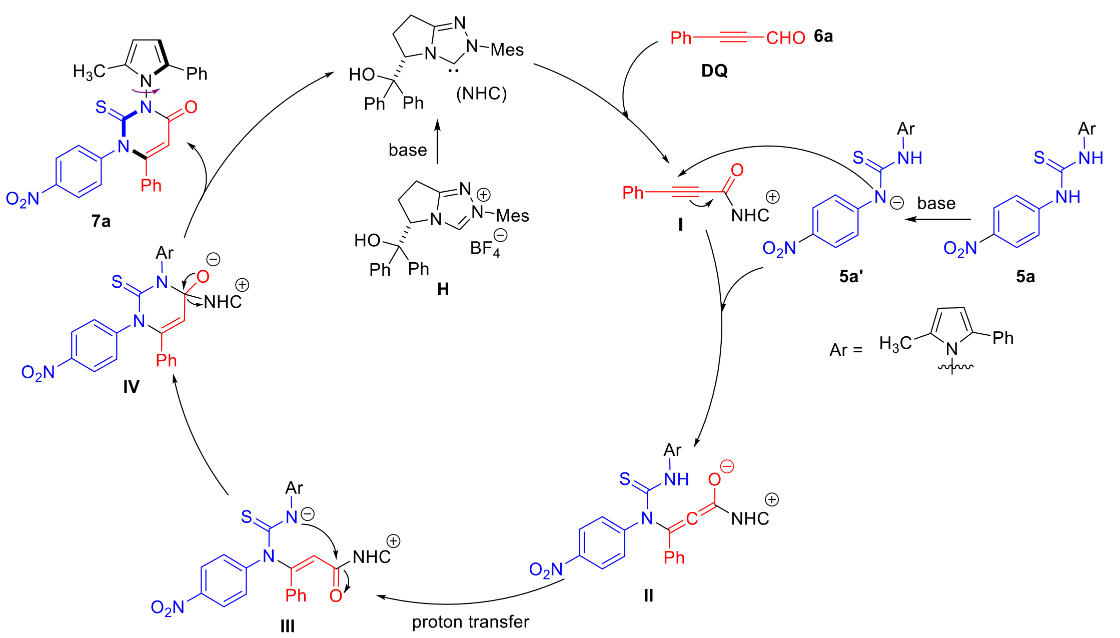

Supplementary Figure 1. Proposed reaction mechanism for the N–N axially chiral thiazine **7a** formation.

6. Synthetic Transformations of Product

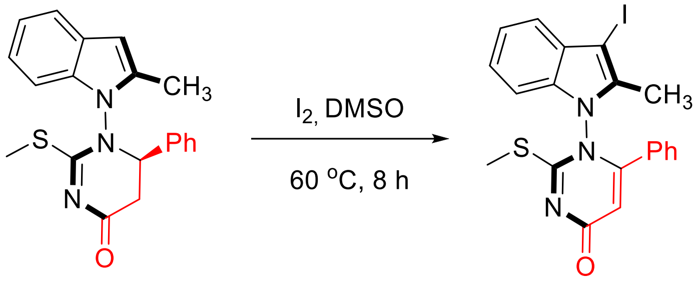

3a, 91% ee　　**4**, 95%, 90% ee

A mixture of **3a** (0.2 mmol, 1.0 equiv.) and iodine (0.4 mmol, 2.0 equiv.) in DMSO (2 mL) was warmed at 60 °C in an oil bath for 8 h. On completion of the reaction, the reaction mixture was poured into a saturated solution of sodium thiosulfate. The precipitated solid was collected and the desired product was purified by column chromatography using silica gel with increasing percentage of ethyl acetate in hexane as eluting solvent. The desired product **4** were obtained in 95% yield, 90% ee.

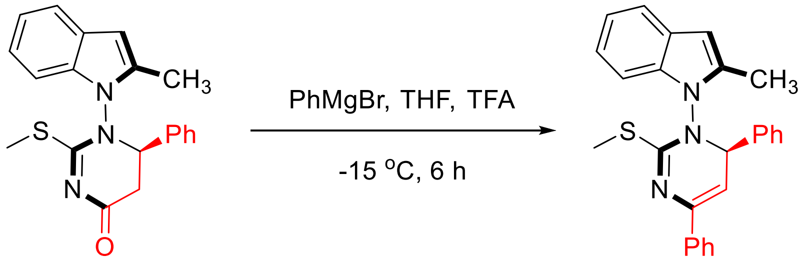

3a, 91% ee　　**10**, 91%, 92% ee

The product **3a** (0.1 mmol, 1.0 equiv.) was suspended in dry THF and then cooled -15 °C. Grignard reagent (PhMgBr, 0.12 mmol, 1.2 equiv.) was then added dropwise, and the reaction mixture was stirred at -15 °C for 5 h. Trifluoroacetic acid (0.3 mmol, 3.0 equiv.) was then carefully added, and the reaction was stirred at -15 °C for another 30 minutes. The mixture was concentrated under reduced pressure, diluted with water, and extracted with EtOAc. The combined organic layers were dried over $MgSO_4$, filtered, and concentrated under reduced pressure. The residue was purified by column chromatography (hexane/ethyl acetate =100:1) to obtain **10** (91%, 92% ee).

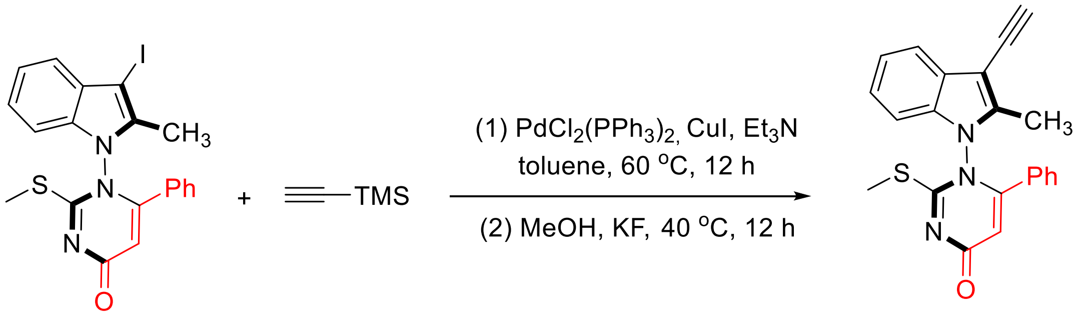

4, 90% ee　　**11**, 95%, 91% ee

Palladium(II)bis(triphenylphosphine) dichloride (10 mol%), **4** (0.2 mmol, 1 equiv.), and Cuprous iodide (0.24 mmol, 1.2 equiv.) were added to an oven-dried Schlenk tube equipped with a stir bar. The tube was then sealed, evacuated, and backfilled with nitrogen three times using standard Schlenk techniques. Et_3N (2 mL), toluene (2 mL) and Trimethylsilylacetylene (0.6 mmol, 3 equiv.) were sequentially added by syringe at ambient temperature. The resulting mixture was vigorously stirred and heated at 60 °C (oil bath) for 12 h. After the mixture was cooled to room temperature, water (10 mL) was added. The resulting mixture was extracted with ethyl acetate (5 mL × 3). The combined organic layers were then washed with brine, dried over Na_2SO_4, and concentrated in vacuum. The residue was used without further purification.

To a mixture of above product (0.2 mmol, 1 equiv.) and MeOH (2 mL) was added KF (1.0 mmol, 5 equiv.), and the resulting mixture was stirred at 40 °C until TLC indicated the reaction was complete. The reaction mixture was diluted with water (5 mL) and extracted with ethyl acetate (5 mL × 3). The combined organic phase washed with brine, dried over Na_2SO_4, and concentrated in vacuum. The residue was purified by column chromatography to give product **11** (95% yield, 91% ee).

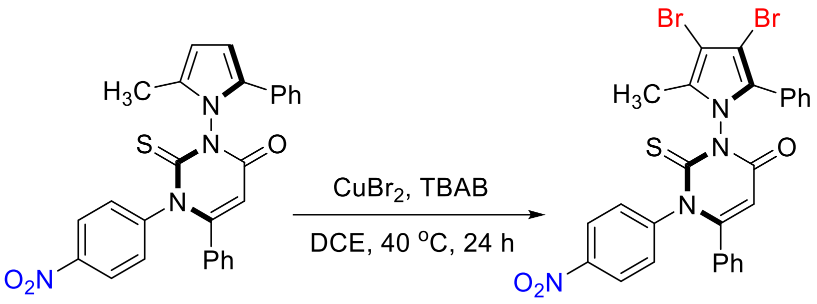

7a, 94% ee   **12**, 67%, 94% ee

To a solution of **7a** (0.2 mmol, 1 equiv.) in dichloroethane (4 mL) was added $CuBr_2$ (0.8 mmol, 4 equiv.) and TBAB (0.02 mmol, 0.1 equiv.). The reaction mixture was stirred at 40 °C for 24 h. The residue was purified directly by column chromatography to afford the desired product as a white solid (67% yield, 94% ee).

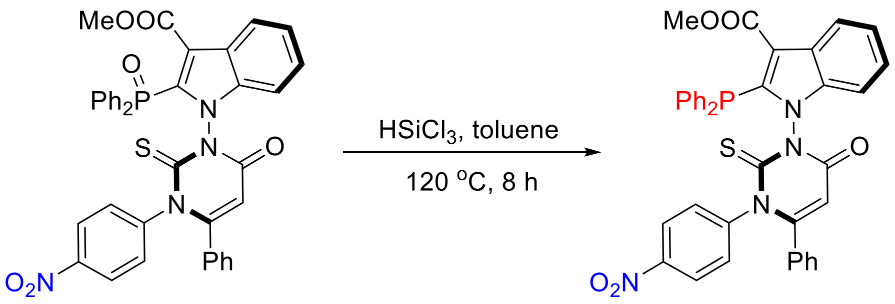

7aj, 86% ee   **13**, 84%, 85% ee

A dried pressure tube charged with **7aj** (0.05 mmol, 1 equiv.) and $HSiCl_3$ (0.15 mmol, 3.0 equiv.) in 0.5 mL toluene was stirred at 120 °C for 8 h under N_2. After removal of the solvent under reduced pressure,

the residue was used without further purification (This phosphine ligand was easily oxidized into phosphorus oxide compounds in air, 84% yield, 85% ee).

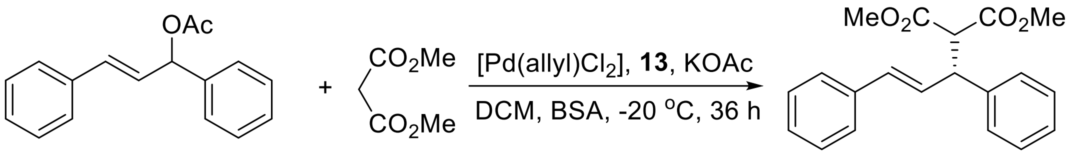

In a Schlenk tube, a solution of 1,3-diphenylprop-2-en-1-yl acetate **14** (0.1mmol, 1.0 equiv.), $[Pd(allyl)Cl]_2$ (0.005 mmol, 0.05 equiv.), and **13** (0.01mmol, 0.1 equiv.) in DCM (1 mL) was stirred at room temperature for 40 min. Dimethyl malonate **15** (0.3mmol, 3 equiv.), KOAc (0.04 mmol, 0.4 equiv.), and BSA (0.3 mmol, 3.0 equiv.) were then added. The reaction mixture was stirred at -20 ^{o}C for 36 h and then quenched with saturated aqueous NH_4Cl and extracted with CH_2Cl_2 (3 × 10 mL). The organic phase was washed with saturated aqueous $NaHCO_3$ and brine, dried over Na_2SO_4, and concentrated under a reduced pressure. The residue was purified by flash chromatography on silica gel with hexane–EtOAc (15:1) as eluent to produce **16** (81% yield, 40% ee).

7. Non-linear effects.

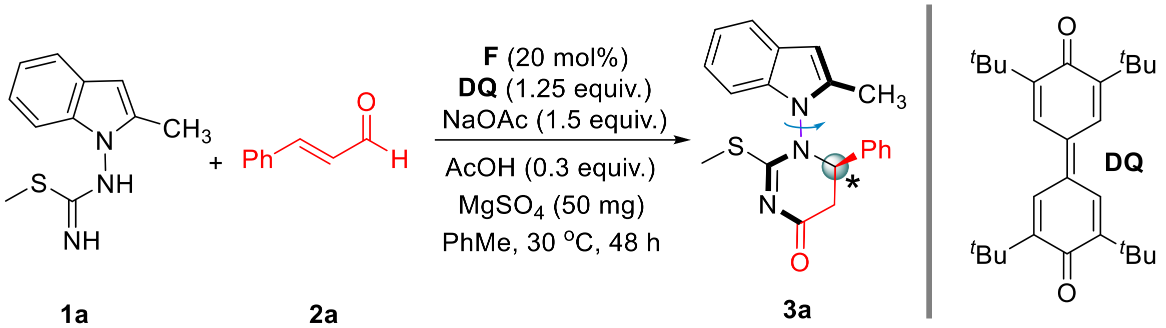

Entry [a]	ee of **F** (%)	ee of **3a** (%)[b]
1	0	-1.3
2	20	-0.6
3	40	-0.9
4	60	0.8
5	70	5.0
6	80	24.3
7	90	41.8
8	100	93.5

[a]Reaction conditions for the synthesis of **3a**: **1a** (0.05 mmol), cinnamaldehyde **2a** (0.09 mmol, 1.8 equiv.), **F** (20 mol%), **DQ** (125 mol%), AcOH (30 mol%), $MgSO_4$ (50 mg) and NaOAc (150 mol%) in PhMe (1 mL) under N_2 atmosphere at 30 °C for 48 h. [b]Determined by chiral HPLC analysis.

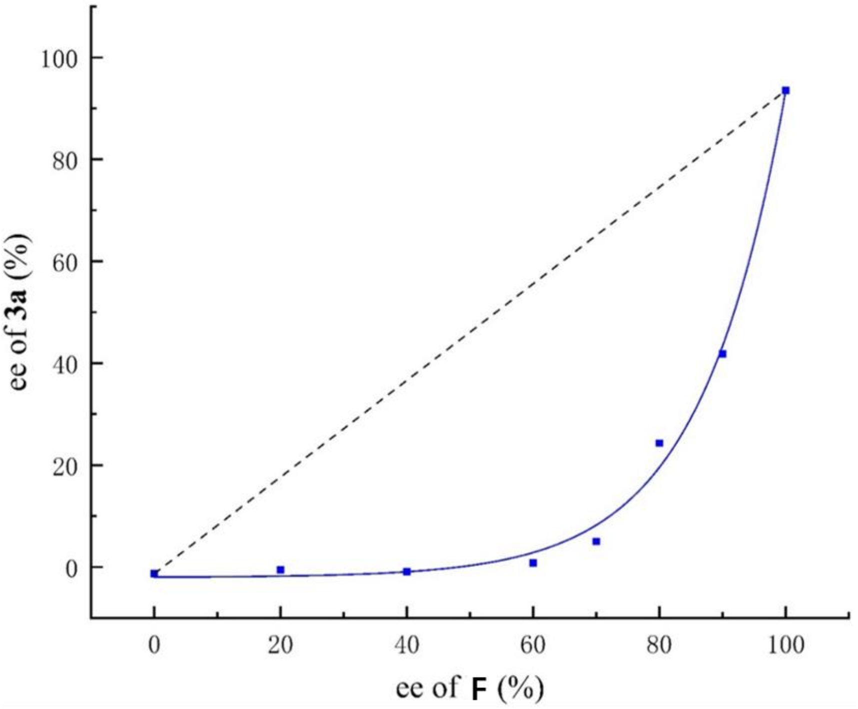

Supplementary Figure 2. Non-linear effects of the ee values of the NHC catalyst **F** and the product **3a.**

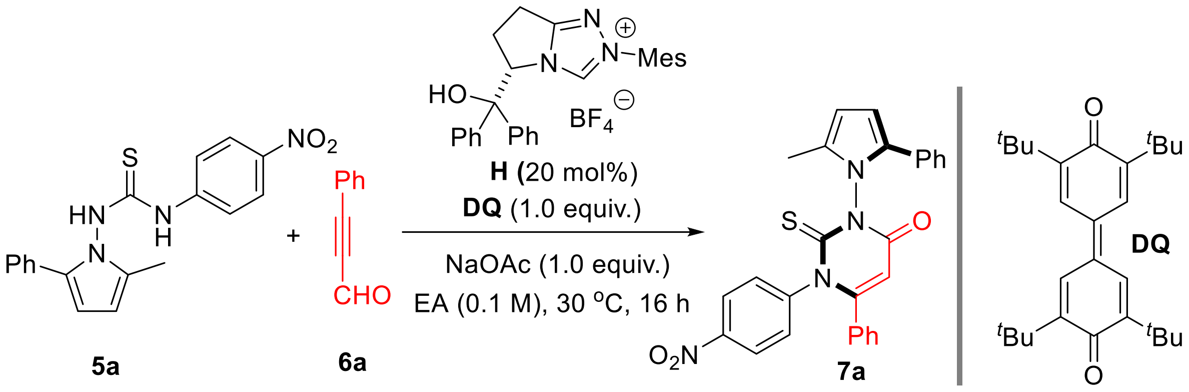

Entry[a]	ee of **H** (%)	ee of **7a** (%)[b]
1	0	-2.1
2	20	17.6
3	40	41.5
4	60	60.7
5	80	78.9
6	100	96.8

[a]Reaction conditions for the synthesis of **7a**: unless other specified, the reactions were performed with **5a** (0.05 mmol, 1.0 equiv.), **6a** (0.06 mmol, 1.2 equiv.), **H** (20 mol%), **DQ** (100 mol%) and base (100 mol%) in solvent (0.5 mL) at 30 °C for 16 h. [b]Determined by chiral HPLC analysis.

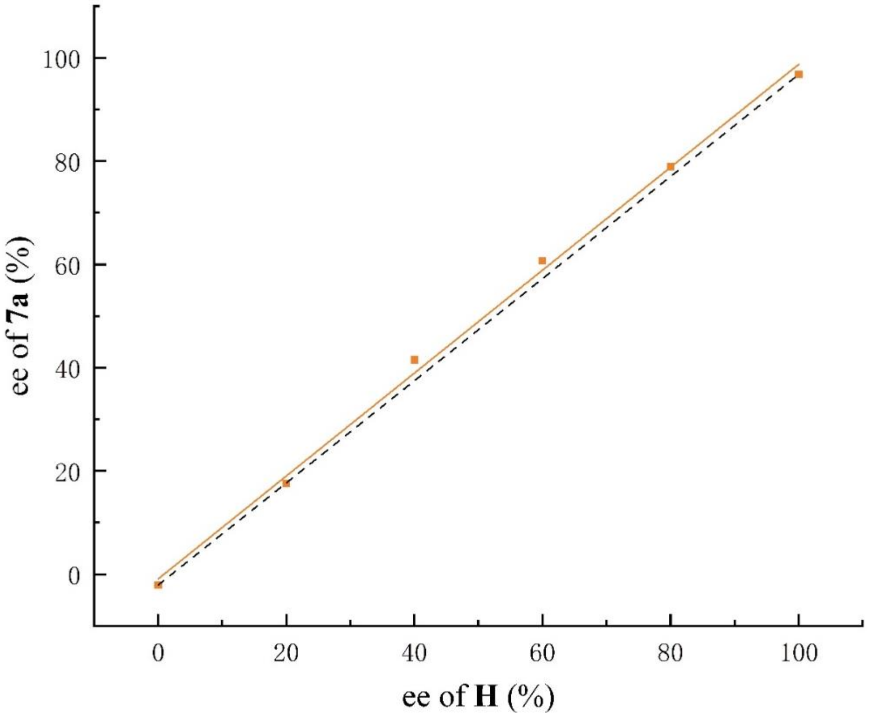

Supplementary Figure 3. Non-linear effects of the ee values of the NHC catalyst **H** and the product **7a.**

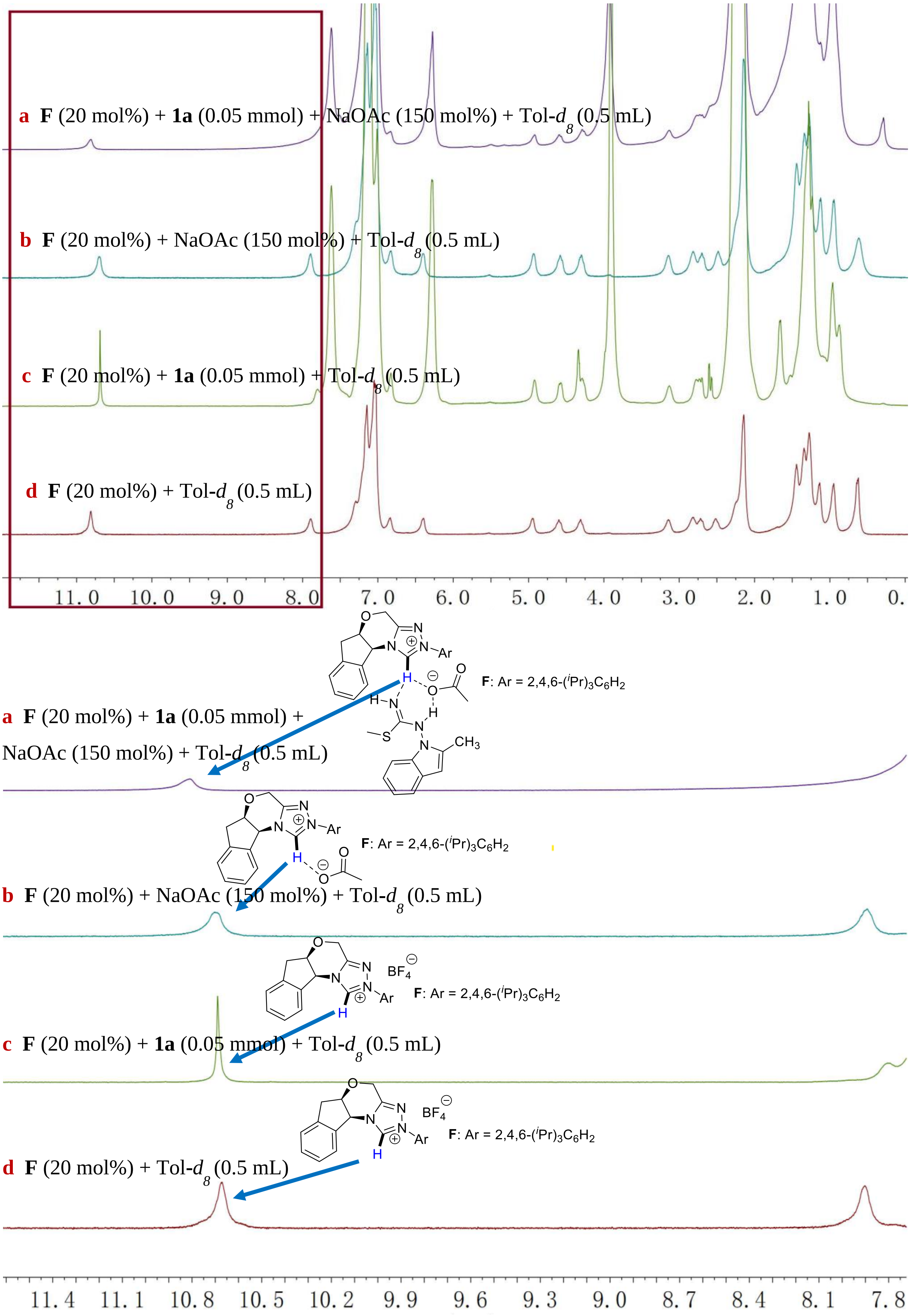

Supplementary Figure 4. ^{1}**H NMR** analysis of the catalytic system

Experimental Procedure: The azolium salt **F** (5.0 mg, 0.01 mmol, 0.2 equiv.) was placed to a dry NMR tube inside a glove box. The tube was sealed properly with a septum. 0.5 mL of d_8-toluene (dried over activated 4 Å MS) was added to the NMR tube under argon atmosphere and resulting a heterogeneous solution. **^{1}H NMR** spectrum of the heterogeneous solution was recorded (Supplementary Figure 4d). Then isothiourea **1a** (11 mg, 0.05 mmol, 1.0 equiv.) was added to the solution and **^{1}H NMR** spectrum was recorded (Supplementary Figure 4c). To a flame dried schlenk tube equipped with a magnetic stir bar, NHC precursor **F** (5.0 mg, 0.01 mmol, 0.2 equiv.), toluene (0.5 mL, stored on activated 4 Å MS) and NaOAc (6.15 mg, 0.075 mmol, 1.5 equiv.) were placed sequentially under argon atmosphere and the resulting mixture was stirred at room temperature for 20 min. The heterogeneous solution was cannulated under positive pressure of argon into another flame dried schlenk tube to get a homogeneous solution. The volatiles (toluene) were removed under vacuum pump and the residue was kept under vacuum for 1 h. 0.5 mL of d_8-toluene (dried over activated 4 Å MS) was added to the residue and the cleared solution was transferred into a NMR tube inside a glove box. The tube was sealed properly with a septum and **^{1}H NMR** spectrum of the solution was recorded (Supplementary Figure 4b). Then isothiourea **1a** (11 mg, 0.05 mmol, 1.0 equiv.) was added to the preformed NHC solution and the resulting mixture was shaked at room temperature for 5 min. And **^{1}H NMR** spectrum was recorded (Supplementary Figure 4a).

8. Enantiomerization barrier determination for 9c.

The enantiomerization barrier, corresponding to barrier to rotation for the following atropisomers, was obtained by kinetic of racemization of an enantiomer. The slope of the first-order kinetic line gives the racemization constant ($k_{racemization} = 2 \times k_{enantiomerization}$). Eyring equation gives the enantiomerization barrier ($\Delta G^{\neq}_{enantiomerization}$) from enantiomerization constant ($k_{enantiomerization}$), R = 8.31454 J·K^{-1} mol^{-1}, h = 6.62608 10^{-34} J·s^{-1} and k_B =1.38066 10^{-23} J·K^{-1}.

About 5 mg of enantio-enriched **9c** was refluxed in 0.5 mL of toluene. Samples of 10 µL of this solution were injected on Daicel Chiralpak IA (*i*-PrOH/hexane = 10/90, flow rate 1.0 mL/min, λ = 254 nm) to monitor the percentage decrease of the second eluted enantiomer over time.

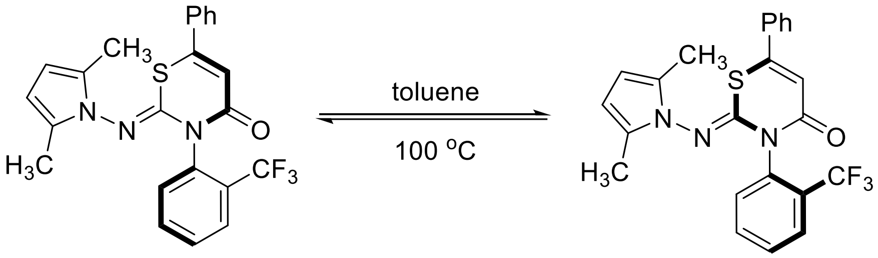

(*R*)-**9c** (*S*)-**9c**

Supplementary Table 2: ee (**9c**) vs time

Time (seconds)	Enantiomeric Excess (ee)	First Order Racemization (In[ee$_0$/ee])
0	59.38	0
3600	51.78	0.137
10800	31.72	0.627
21600	13.64	1.471
39600	3.46	2.842

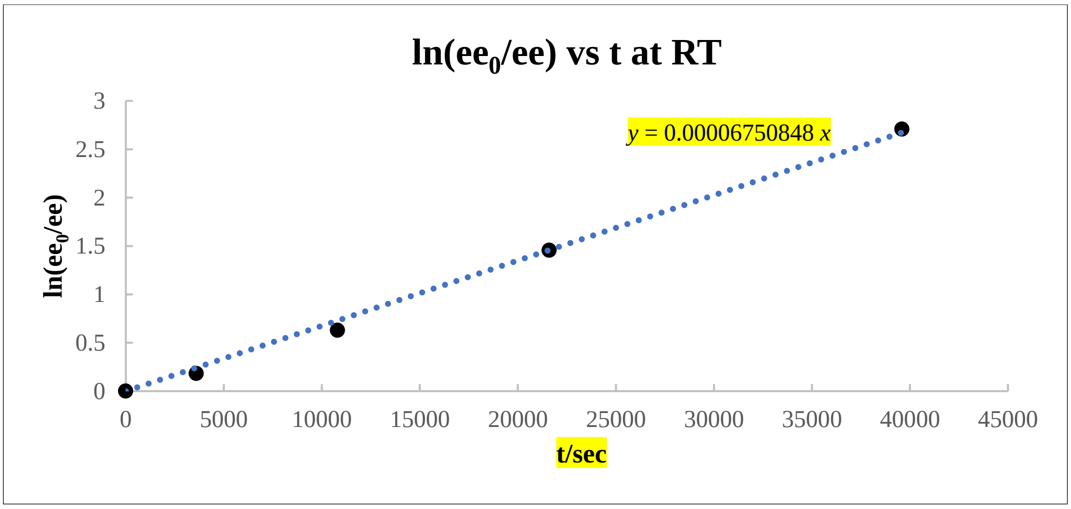

Supplementary Figure 5. ln (ee$_0$/ee) (**9c**) vs time.

$k_{racemization}$ (100 °C) = 7.003659×10^{-5} s^{-1}

$k_{enantiomerization}$ (100 °C) = 3.501829×10^{-5} s^{-1}

$\Delta G^{\neq}_{enantiomerization}$ = 123.870 kJ·mol^{-1} = 29.6 kcal·mol^{-1}

$k_{racemization}$ (25 °C) = 2.474068×10^{-9} s^{-1}

$t_{1/2}$(25 °C) = 3242.7 days

The proposed reaction mechanism is depicted in the Supplementary Figure 6. The free NHC catalyst is generated in situ from the pre-catalyst **H** under basic conditions and reacts with the ynal **6a** to give an NHC-bounded Breslow intermediate, which then undergoes oxidation to generate the alkynyl acylazolium intermediate **I** and reacts with the deprotonated nucleophilic substrate **5ah'** through the thia-Michael addition process to form the allenolate intermediate **II**, which can lead to the new acylazolium intermediate **III** through a proton transfer process. Next N–C bond is formed to create **IV** and the NHC catalyst can be released and finally affords the C–N axially chiral thiazine **9a**.

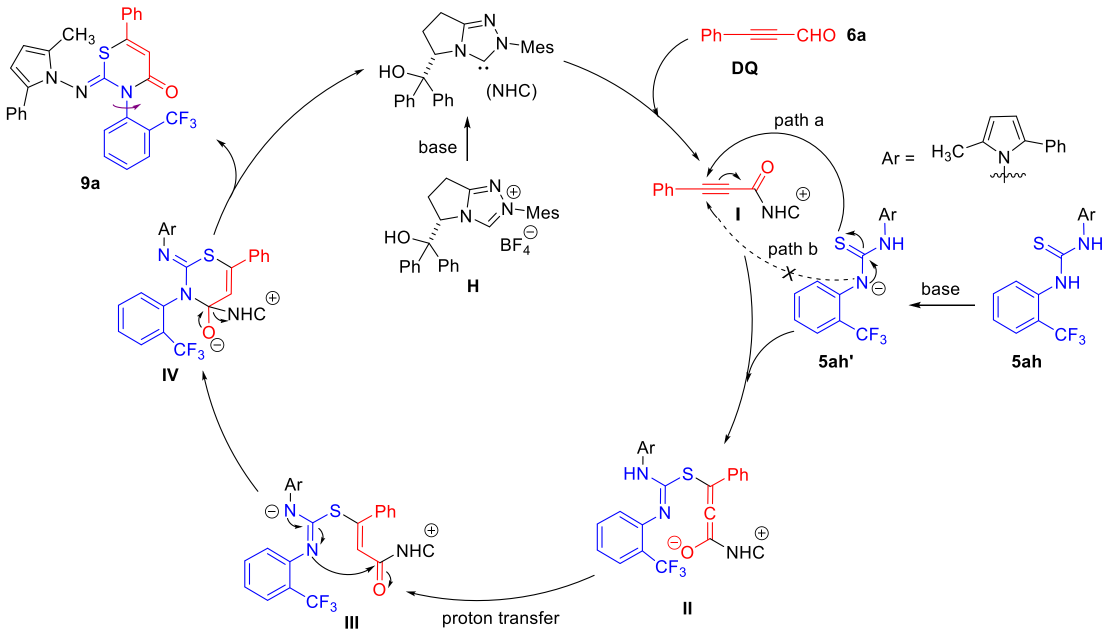

Supplementary Figure 6. Proposed reaction mechanism for the C–N axially chiral thiazine **9a** formation.

9. Characterizations of N–N axially chiral pyrroles and indoles.

(*R*$_a$,*R*)-1-(2-methyl-1H-indol-1-yl)-2-(methylthio)-6-phenyl-5,6-dihydropyrimidin-4(1H)-one (3a)

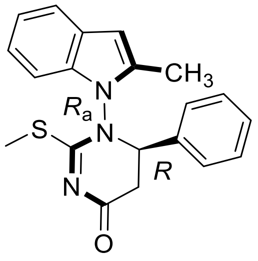

White solid. **MP**: 103-105 °C. **^{1}H NMR** (500 MHz, $CDCl_3$) δ 7.50 (d, J = 7.8 Hz, 1H), 7.36 – 7.16 (m, 6H), 7.10 – 6.98 (m, 2H), 6.07 (s, 1H), 5.15 – 4.88 (m, 1H), 3.34 (dd, J = 15.6, 7.5 Hz, 1H), 3.21 (dd, J = 15.6, 5.9 Hz, 1H), 2.39 (s, 3H), 1.66 (s, 3H). **^{13}C NMR** (126 MHz, $CDCl_3$) δ 176.1, 172.7, 137.2, 135.9, 133.7, 129.5, 128.8, 127.7, 126.9, 122.4, 121.7, 120.7, 108.2, 100.8, 62.1, 37.4, 14.2, 11.2. **HRMS (ESI)** m/z calcd for $[C_{20}H_{19}N_3OS, M+Na]^+$: 372.1141; found: 372.1153.

Specific Rotation: $[\alpha]^{25}_D$ = +123.7 (*c* = 1.0, $CHCl_3$). 94% ee (HPLC condition: Chiralcel AD- H column, *n*-Hexane/*i*-PrOH = 70:30, flow rate = 1.0 mL/min, wavelength = 254 nm, t_R = 5.723 min for major isomer, t_R = 7.237 min for minor isomer).

(*S*$_a$,*R*)-1-(2-methyl-1H-indol-1-yl)-2-(methylthio)-6-phenyl-5,6-dihydropyrimidin-4(1H)-one (3a')

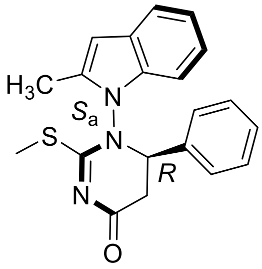

White solid. **MP**: 103-105 °C. **^{1}H NMR** (500 MHz, $CDCl_3$) δ 7.35 (d, J = 7.8 Hz, 1H), 7.23 – 7.19 (m, 1H), 7.15 (d, J = 4.4 Hz, 4H), 6.96 (t, J = 7.5 Hz, 1H), 6.85 (t, J = 7.7 Hz, 1H), 6.47 (d, J = 8.2 Hz, 1H), 6.31 (s, 1H), 4.87 (dd, J = 7.2, 5.7 Hz, 1H), 3.30 (dd, J = 15.6, 7.2 Hz, 1H), 3.18 (dd, J = 15.6, 5.7 Hz, 1H), 2.47 (s, 3H), 2.38 (s, 3H). **^{13}C NMR** (126 MHz, $CDCl_3$) δ 176.0, 172.6, 136.1, 135.4, 134.1, 129.4, 128.8, 127.5, 125.5, 121.9, 121.0, 119.8, 109.4, 102.0, 64.4, 37.9, 14.1, 11.9. **HRMS (ESI)** m/z calcd for $[C_{20}H_{19}N_3OS, M+Na]^+$: 372.1141; found: 372.1150.

Specific Rotation: $[\alpha]^{25}_D$ = -107.7 (*c* = 1.0, $CHCl_3$). 91% ee (HPLC condition: Chiralcel AD- H column, *n*-Hexane/*i*-PrOH = 70:30, flow rate = 1.0 mL/min, wavelength = 254 nm, t_R = 5.758 min for minor isomer, t_R = 6.827 min for major isomer).

(*R*$_a$,*R*)-1-(2-methyl-1H-indol-1-yl)-2-(methylthio)-6-(p-tolyl)-5,6-dihydropyrimidin-4(1H)-one (3b)

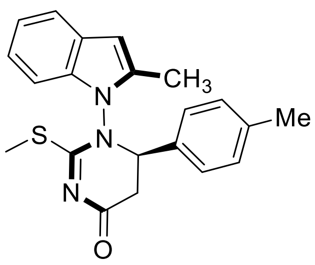

Yellow solid. **MP**: 103-105 °C. **^{1}H NMR** (500 MHz, $CDCl_3$) δ 7.51 (d, J = 7.7 Hz, 1H), 7.35 – 7.18 (m, 3H), 7.03 (d, J = 7.8 Hz, 2H), 6.90 (d, J = 7.8 Hz, 2H), 6.07 (s, 1H), 5.01 (t, J = 6.7 Hz, 1H), 3.32 (dd, J = 15.5, 7.4 Hz, 1H), 3.20 (dd, J = 15.5, 6.0 Hz, 1H), 2.40 (s, 3H), 2.29 (s, 3H), 1.67 (s, 3H). **^{13}C NMR** (126 MHz, $CDCl_3$) δ 176.1, 172.9, 139.5, 137.3, 133.8, 132.9, 129.5, 127.7, 126.9, 122.4, 121.7, 120.7, 108.2, 100.8, 61.9, 37.6, 21.1, 14.2, 11.3. **HRMS (ESI)** m/z calcd for $[C_{21}H_{21}N_3OS, M+Na]^+$: 386.1297; found: 386.1306.

Specific Rotation: $[\alpha]^{25}_{D}$ = +142.3 (*c* = 1.0, $CHCl_3$). 91% ee (HPLC condition: Chiralcel IA column, *n*-Hexane/*i*-PrOH = 70:30, flow rate = 1.0 mL/min, wavelength = 254 nm, t_R = 5.867 min for major isomer, t_R = 6.660 min for minor isomer).

(*R*a,*R*)-6-([1,1'-biphenyl]-4-yl)-1-(2-methyl-1H-indol-1-yl)-2-(methylthio)-5,6-dihydropyrimidin-4(1H)-one (3c)

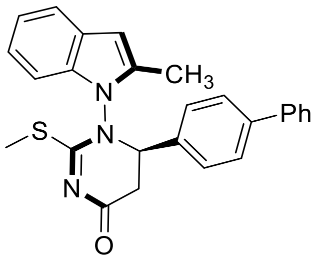

Yellow solid. **MP**: 121-123 °C. **^{1}H NMR** (500 MHz, $CDCl_3$) δ 7.55 – 7.50 (m, 3H), 7.49 – 7.40 (m, 4H), 7.38 – 7.25 (m, 4H), 7.10 (d, J = 8.0 Hz, 2H), 6.09 (t, J = 1.1 Hz, 1H), 5.17 – 4.97 (m, 1H), 3.38 (dd, J = 15.5, 7.5 Hz, 1H), 3.24 (dd, J = 15.6, 5.8 Hz, 1H), 2.42 (s, 3H), 1.71 (d, J = 1.1 Hz, 3H). **^{13}C NMR** (126 MHz, $CDCl_3$) δ 176.2, 172.7, 142.3, 139.8, 137.2, 134.8, 133.8, 128.8, 128.2, 127.8, 127.4, 126.9, 122.5, 121.8, 120.8, 108.3, 101.0, 61.8, 37.5, 14.3, 11.4. **HRMS (ESI)** m/z calcd for $[C_{26}H_{23}N_3OS, M+Na]^+$: 448.1454; found: 448.1460.

Specific Rotation: $[\alpha]^{25}_{D}$ = +154.3 (*c* = 1.0, $CHCl_3$). 93% ee (HPLC condition: Chiralcel IA column, *n*-Hexane/*i*-PrOH = 70:30, flow rate = 1.0 mL/min, wavelength = 254 nm, t_R = 7.542 min for major isomer, t_R =8.353 min for minor isomer).

(*R*a,*R*)-6-(4-methoxyphenyl)-1-(2-methyl-1H-indol-1-yl)-2-(methylthio)-5,6-dihydropyrimidin-4(1H)-one (3d)

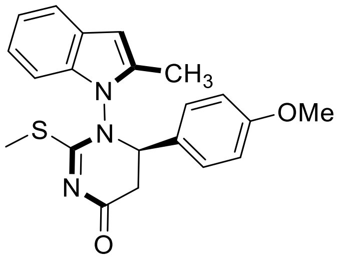

Yellow solid. **MP**: 112-114 °C. **^{1}H NMR** (500 MHz, $CDCl_3$) δ 7.50 (d, *J* = 7.6 Hz, 1H), 7.33 – 7.15 (m, 3H), 6.92 (d, *J* = 8.2 Hz, 2H), 6.73 (d, *J* = 8.4 Hz, 2H), 6.07 (s, 1H), 5.02 (t, *J* = 6.8 Hz, 1H), 3.75 (s, 3H), 3.30 (dd, *J* = 15.5, 7.2 Hz, 1H), 3.21 (dd, *J* = 15.5, 6.5 Hz, 1H), 2.40 (s, 3H), 1.70 (s, 3H). **^{13}C NMR** (126 MHz, $CDCl_3$) δ 176.1, 173.0, 160.4, 137.3, 133.8, 129.2, 127.8, 126.9, 122.4, 121.7, 120.7, 114.1, 108.3, 100.8, 61.5, 55.2, 37.7, 14.3, 11.3. **HRMS (ESI)** m/z calcd for $[C_{21}H_{21}N_3O_2S, M+Na]^+$: 402.1246; found: 402.1256.

Specific Rotation: $[\alpha]^{25}_D$ = +78.6 (*c* = 1.0, $CHCl_3$). 90% ee (HPLC condition: Chiralcel AD-H column, *n*-Hexane/*i*-PrOH = 80:20, flow rate = 1.0 mL/min, wavelength = 254 nm, t_R = 8.928 min for major isomer, t_R = 14.558 min for minor isomer).

(*R*a,*R*)-6-(4-(dimethylamino)phenyl)-1-(2-methyl-1H-indol-1-yl)-2-(methylthio)-5,6-dihydropyrimidin-4(1H)-one (3e)

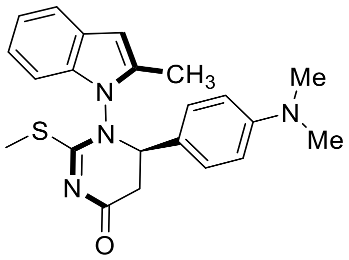

Yellow solid. **MP**: 114-117 °C. **^{1}H NMR** (500 MHz, $CDCl_3$) δ 7.50 (d, J = 7.7 Hz, 1H), 7.28 (d, J = 7.7 Hz, 1H), 7.24 (d, J = 7.8 Hz, 1H), 7.22 – 7.17 (m, 1H), 6.83 (d, J = 8.4 Hz, 2H), 6.49 (d, J = 8.4 Hz, 2H), 6.08 (s, 1H), 4.97 (t, J = 6.9 Hz, 1H), 3.26 (qd, J = 15.5, 7.0 Hz, 2H), 2.90 (s, 6H), 2.39 (s, 3H), 1.72 (s, 3H). **^{13}C NMR** (126 MHz, $CDCl_3$) δ 175.9, 173.5, 151.0, 137.5, 133.9, 128.8, 126.9, 122.7, 122.3, 121.6, 120.6, 111.9, 108.3, 100.5, 61.8, 40.1, 37.7, 14.2, 11.3. **HRMS (ESI)** m/z calcd for $[C_{22}H_{24}N_4OS, M+Na]^+$: 415.1563; found: 415.1576.

Specific Rotation: $[\alpha]^{25}_D$ = +115.0 (*c* = 1.0, $CHCl_3$). 92% ee (HPLC condition: Chiralcel IA column, *n*-Hexane/*i*-PrOH = 70:30, flow rate = 1.0 mL/min, wavelength = 254 nm, t_R = 6.968 min for major isomer, t_R = 7.973 min for minor isomer).

(*R*a,*R*)-6-(4-fluorophenyl)-1-(2-methyl-1H-indol-1-yl)-2-(methylthio)-5,6-dihydropyrimidin-4(1H)-one (3f)

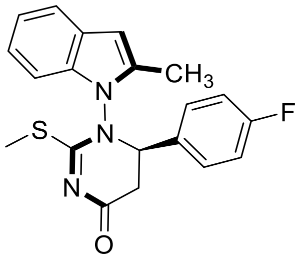

White solid. **MP**: 112-114 °C. **^{1}H NMR** (500 MHz, $CDCl_3$) δ 7.51 (d, J = 7.7 Hz, 1H), 7.34 – 7.18 (m, 3H), 7.04 – 6.97 (m, 2H), 6.92 (t, J = 8.6 Hz, 2H), 6.09 (s, 1H), 5.06 (t, J = 6.8 Hz, 1H), 3.32 (dd, J = 15.5, 7.3 Hz, 1H), 3.19 (dd, J = 15.5, 6.4 Hz, 1H), 2.40 (s, 3H), 1.71 (s, 3H). **^{13}C NMR** (126 MHz, $CDCl_3$) δ 176.3, 172.5, 163.2 (d, J = 250.0 Hz), 137.0, 133.7, 131.7 (d, J = 3.4 Hz), 129.7 (d, J = 8.7 Hz), 126.9, 122.6, 121.9, 120.8, 115.9 (d, J = 21.8 Hz), 108.3, 101.1, 61.3, 37.6, 14.3, 11.3. **^{19}F NMR** (471 MHz, $CDCl_3$) δ -110.87. **HRMS (ESI)** m/z calcd for calcd for [$C_{20}H_{18}FN_3OS$, M+Na]$^+$: 390.1047; found: 390.1055.

Specific Rotation: $[\alpha]^{25}_{D}$ = +98.8 (*c* = 1.0, $CHCl_3$). 90% ee (HPLC condition: Chiralcel AD-H column, *n*-Hexane/*i*-PrOH = 70:30, flow rate = 1.0 mL/min, wavelength = 254 nm, t_R = 5.568 min for major isomer, t_R = 7.088 min for minor isomer).

(R_a,*R*)-6-(4-chlorophenyl)-1-(2-methyl-1H-indol-1-yl)-2-(methylthio)-5,6-dihydropyrimidin-4(1H)-one (3g)

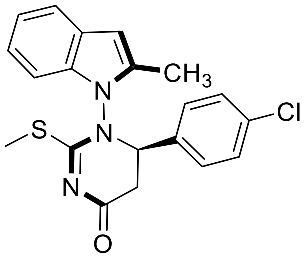

White solid. **MP**: 112-114 °C. **^{1}H NMR** (500 MHz, $CDCl_3$) δ 7.51 (d, J = 7.7 Hz, 1H), 7.35 – 7.17 (m, 5H), 6.96 (d, J = 8.1 Hz, 2H), 6.09 (s, 1H), 5.04 (t, J = 6.7 Hz, 1H), 3.31 (dd, J = 15.5, 7.3 Hz, 1H), 3.16 (dd, J = 15.5, 6.1 Hz, 1H), 2.40 (s, 3H), 1.72 (s, 3H). **^{13}C NMR** (126 MHz, $CDCl_3$) δ 176.3, 172.3, 136.9, 135.6, 134.4, 133.7, 129.2, 129.1, 127.0, 122.6, 121.9, 120.8, 108.2, 101.2, 61.4, 37.5, 14.3, 11.4. **HRMS (ESI)** m/z calcd for [$C_{20}H_{18}ClN_3OS$, M+Na]$^+$: 406.0751; found: 406.0757.

Specific Rotation: $[\alpha]^{25}_{D}$ = +134.4 (*c* = 1.0, $CHCl_3$). 90% ee (HPLC condition: Chiralcel AD column, *n*-Hexane/*i*-PrOH = 70:30, flow rate = 1.0 mL/min, wavelength = 254 nm, t_R = 6.135 min for major isomer, t_R = 7.268 min for minor isomer).

(R_a,*R*)-6-(4-bromophenyl)-1-(2-methyl-1H-indol-1-yl)-2-(methylthio)-5,6-dihydropyrimidin-4(1H)-one (3h)

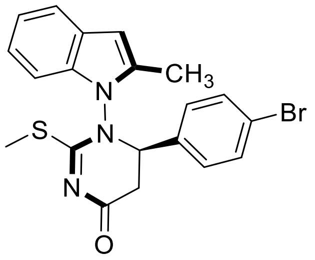

White solid. **MP**: 115-117 °C. **^{1}H NMR** (500 MHz, $CDCl_3$) δ 7.52 (d, J = 7.7 Hz, 1H), 7.37 (d, J = 8.2 Hz, 2H), 7.33 – 7.26 (m, 1H), 7.24 – 7.17 (m, 2H), 6.90 (d, J = 8.1 Hz, 2H), 6.16 – 5.93 (m, 1H), 5.02 (t, J = 6.7 Hz, 1H), 3.32 (dd, J = 15.5, 7.3 Hz, 1H), 3.16 (dd, J = 15.5, 6.0 Hz, 1H), 2.40 (s, 3H), 1.73 (s, 3H).**^{13}C NMR** (126 MHz, $CDCl_3$) δ 176.4, 172.3, 137.0, 134.9, 133.7, 132.1, 129.5, 127.0, 123.8, 122.6, 122.0, 120.9, 108.3, 101.2, 61.5, 37.5, 14.3, 11.4. **HRMS (ESI)** m/z calcd for $[C_{20}H_{18}BrN_3OS, M+Na]^+$: 450.0246; found: 450.0246.

Specific Rotation: $[\alpha]^{25}_{D}$ = +87.1 (*c* = 1.0, $CHCl_3$). 91% ee (HPLC condition: Chiralcel AD-H column, *n*-Hexane/*i*-PrOH = 80:20, flow rate = 1.0 mL/min, wavelength = 254 nm, t_R = 8.897 min for major isomer, t_R = 10.442 min for minor isomer).

(*R*a,*R*)-1-(2-methyl-1H-indol-1-yl)-2-(methylthio)-6-(m-tolyl)-5,6-dihydropyrimidin-4(1H)-one (3i)

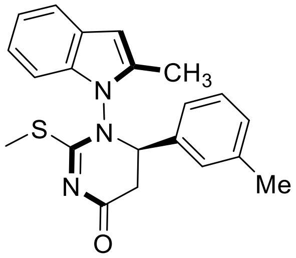

White solid. **MP**: 105-107 °C. **^{1}H NMR** (500 MHz, $CDCl_3$) δ 7.49 (d, J = 7.7 Hz, 1H), 7.27 – 7.19 (m, 3H), 7.10 (d, J = 4.8 Hz, 2H), 6.87 – 6.72 (m, 2H), 6.06 (s, 1H), 4.99 (dd, J = 7.5, 6.0 Hz, 1H), 3.31 (dd, J = 15.5, 7.5 Hz, 1H), 3.19 (dd, J = 15.6, 6.0 Hz, 1H), 2.40 (s, 3H), 2.20 (s, 3H), 1.67 (d, J = 1.1 Hz, 3H). **^{13}C NMR** (126 MHz, $CDCl_3$) δ 176.2, 172.8, 138.7, 137.4, 135.9, 133.9, 130.2, 128.8, 128.5, 127.0, 124.9, 122.5, 121.8, 120.7, 108.3, 100.9, 62.1, 37.5, 21.2, 14.3, 11.3. **HRMS (ESI)** m/z calcd for $[C_{21}H_{21}N_3OS, M+Na]^+$: 386.1297; found: 386.1308.

Specific Rotation: $[\alpha]^{25}_{D}$ = +89.4 (*c* = 1.0, $CHCl_3$). 90% ee (HPLC condition: Chiralcel AD-H column, *n*-Hexane/*i*-PrOH = 80:20, flow rate = 1.0 mL/min, wavelength = 254 nm, t_R = 6.178 min for major isomer, t_R = 8.465 min for minor isomer).

(*R*a,*R*)-6-(3-methoxyphenyl)-1-(2-methyl-1H-indol-1-yl)-2-(methylthio)-5,6-dihydropyrimidin-4(1H)-one (3j)

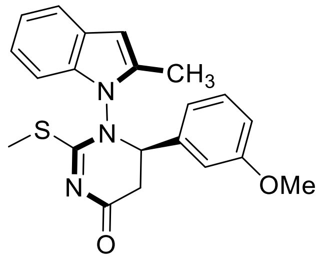

White solid. **MP**: 100-102 °C. **^{1}H NMR** (500 MHz, $CDCl_3$) δ 7.50 (d, J = 7.8 Hz, 1H), 7.33 – 7.11 (m, 4H), 6.83 (d, J = 8.4 Hz, 1H), 6.69 (d, J = 7.7 Hz, 1H), 6.42 (s, 1H), 6.07 (s, 1H), 5.02 (t, J = 6.9 Hz, 1H), 3.56 (s, 3H), 3.31 (dd, J = 15.5, 7.2 Hz, 1H), 3.22 (dd, J = 15.6, 6.5 Hz, 1H), 2.39 (s, 3H), 1.73 (s, 3H).**^{13}C NMR** (126 MHz, $CDCl_3$) δ 176.2, 172.7, 159.6, 137.3, 136.9, 133.8, 129.9, 126.9, 122.4, 121.7, 120.7, 119.6, 115.4, 113.2, 108.3, 100.8, 61.9, 55.0, 37.4, 14.2, 11.2. **HRMS (ESI)** m/z calcd for [$C_{21}H_{21}N_3O_2S$, M+Na]$^+$: 402.1246; found: 402.1260.

Specific Rotation: $[\alpha]^{25}_D$ = +214.1 (*c* = 1.0, $CHCl_3$). 90% ee (HPLC condition: Chiralcel AD-H column, *n*-Hexane/*i*-PrOH = 80:20, flow rate = 1.0 mL/min, wavelength = 254 nm, t_R = 7.860 min for major isomer, t_R = 11.482 min for minor isomer).

(*R*a,*R*)-6-(furan-2-yl)-1-(2-methyl-1H-indol-1-yl)-2-(methylthio)-5,6-dihydropyrimidin-4(1H)-one (3k)

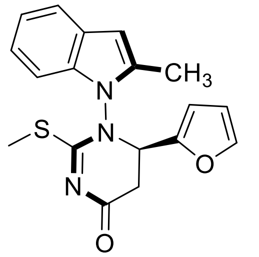

Yellow solid. **MP**: 101-103 °C. **^{1}H NMR** (500 MHz, $CDCl_3$) δ 7.51 (d, J = 7.8 Hz, 1H), 7.38 – 7.34 (m, 1H), 7.26 (d, J = 5.7 Hz, 1H), 7.20 (d, J = 7.2 Hz, 2H), 6.21 (t, J = 2.5 Hz, 1H), 6.14 (s, 1H), 5.88 (d, J = 3.3 Hz, 1H), 5.16 (t, J = 7.2 Hz, 1H), 3.33 (dd, J = 15.6, 7.7 Hz, 1H), 3.24 (dd, J = 15.6, 6.7 Hz, 1H), 2.40 (s, 3H), 1.85 (s, 3H). **^{13}C NMR** (126 MHz, $CDCl_3$) δ 176.0, 172.2, 147.9, 143.4, 137.3, 134.0, 127.0, 122.5, 121.8, 120.7, 111.0, 110.8, 108.2, 100.9, 55.1, 35.9, 14.3, 10.4. **HRMS (ESI)** m/z calcd for [$C_{18}H_{17}N_3O_2S$, M+Na]$^+$: 362.0933; found: 362.0935.

Specific Rotation: $[\alpha]^{25}_D$ = +71.5 (*c* = 1.0, $CHCl_3$). 90% ee (HPLC condition: Chiralcel IA column, *n*-Hexane/*i*-PrOH = 80:20, flow rate = 1.0 mL/min, wavelength = 254 nm, t_R = 7.483 min for major isomer, t_R = 9.278 min for minor isomer).

(*R*a,*R*)-1-(2-methyl-1H-indol-1-yl)-2-(methylthio)-6-(3,3,3-trifluoroprop-1-en-1-yl)-5,6-dihydropyrimidin-4(1H)-one (3l)

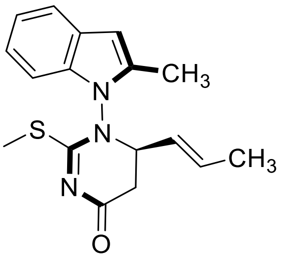

Yellow solid. **MP**: 103-105 °C. **^{1}H NMR** (500 MHz, $CDCl_3$) δ 7.52 (d, J = 7.6 Hz, 1H), 7.25 – 7.21 (m, 1H), 7.18 (d, J = 7.0 Hz, 1H), 7.13 (d, J = 8.0 Hz, 1H), 6.27 (s, 1H), 5.26 (d, J = 4.3 Hz, 2H), 4.60 (ddd, J = 9.8, 5.0, 2.6 Hz, 1H), 3.01 (dd, J = 15.4, 5.5 Hz, 1H), 2.88 (dd, J = 15.4, 9.7 Hz, 1H), 2.36 (d, J = 14.9 Hz, 6H), 1.45 (d, J = 4.2 Hz, 3H). **^{13}C NMR** (126 MHz, $CDCl_3$) δ 176.4, 173.0, 137.1, 134.4, 134.0, 126.7, 124.3, 122.4, 121.6, 120.6, 108.5, 101.0, 61.2, 38.2, 17.5, 14.3, 12.0. **HRMS (ESI)** m/z calcd for $[C_{17}H_{19}N_3OS, M+Na]^+$: 336.1141; found: 336.1149.

Specific Rotation: $[\alpha]^{25}_D$ = +118.4 (*c* = 1.0, $CHCl_3$). 78% ee (HPLC condition: Chiralcel AD-H column, *n*-Hexane/*i*-PrOH = 70:30, flow rate = 1.0 mL/min, wavelength = 254 nm, t_R = 8.997 min for minor isomer, t_R = 13.858 min for major isomer).

(*R*$_a$,*S*)-1-(2-methyl-1H-indol-1-yl)-2-(methylthio)-6-propyl-5,6-dihydropyrimidin-4(1H)-one (3m)

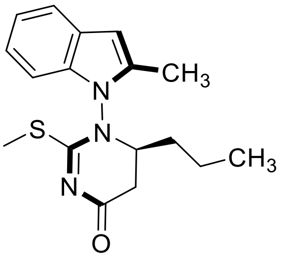

White solid. **MP**: 103-105 °C. **^{1}H NMR** (500 MHz, $CDCl_3$) δ 7.60 – 7.52 (m, 1H), 7.21 (ddd, J = 13.7, 7.5, 1.3 Hz, 2H), 7.13 – 7.06 (m, 1H), 6.34 (t, J = 1.1 Hz, 1H), 4.27 – 4.13 (m, 1H), 3.04 (dd, J = 15.5, 5.3 Hz, 1H), 2.76 (dd, J = 15.5, 10.0 Hz, 1H), 2.40 (d, J = 1.1 Hz, 3H), 2.35 (s, 3H), 1.40 (ddt, J = 9.6, 4.8, 2.5 Hz, 1H), 1.31 (dddd, J = 12.8, 10.3, 7.1, 3.7 Hz, 1H), 1.11 (ddd, J = 14.1, 7.3, 4.1 Hz, 2H), 0.75 (t, J = 7.2 Hz, 3H). **^{13}C NMR** (126 MHz, $CDCl_3$) δ 177.1, 173.3, 136.7, 133.9, 126.6, 122.5, 121.7, 120.7, 108.3, 101.1, 58.7, 37.1, 33.2, 17.8, 14.2, 13.5, 11.8. **HRMS (ESI)** m/z calcd for $[C_{17}H_{21}N_3OS, M+Na]^+$: 338.1297; found: 338.1300.

Specific Rotation: $[\alpha]^{25}_D$ = +156.3 (*c* = 1.0, $CHCl_3$). 73% ee (HPLC condition: Chiralcel OD-H column, *n*-Hexane/*i*-PrOH = 90:10, flow rate = 1.0 mL/min, wavelength = 254 nm, t_R = 12.648 min for major isomer, t_R = 15.300 min for minor isomer).

(*S*$_a$,*S*)-1-(2-methyl-1H-indol-1-yl)-2-(methylthio)-6-propyl-5,6-dihydropyrimidin-4(1H)-one (3m')

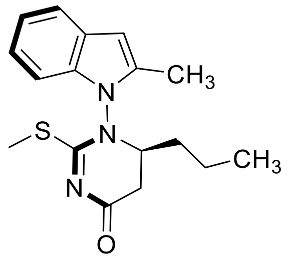

Yellow solid. **MP**: 103-105 °C. **^{1}H NMR** (500 MHz, $CDCl_3$) δ 7.52 (d, J = 7.7 Hz, 1H), 7.23 (d, J = 4.1 Hz, 2H), 7.20 – 7.13 (m, 1H), 6.38 (s, 1H), 4.02 – 3.88 (m, 1H), 2.99 (dd, J = 15.3, 5.8 Hz, 1H), 2.79 (dd, J = 15.3, 8.0 Hz, 1H), 2.34 (d, J = 3.5 Hz, 6H), 1.57 (ddt, J = 15.6, 9.7, 5.3 Hz, 1H), 1.39 (ddt, J = 19.3, 15.1, 4.8 Hz, 2H), 1.13 (dt, J = 14.2, 5.5 Hz, 1H), 0.77 (d, J = 7.3 Hz, 3H). **^{13}C NMR** (126 MHz, $CDCl_3$) δ 176.0, 173.1, 136.0, 134.8, 125.9, 122.6, 121.5, 120.4, 109.0, 101.8, 60.7, 36.6, 33.5, 18.0, 14.1, 13.5, 11.7. **HRMS (ESI)** m/z calcd for $[C_{17}H_{21}N_3OS, M+Na]^+$: 338.1297; found: 338.1302.

Specific Rotation: $[\alpha]^{25}_{D}$ = -117.2 (*c* = 1.0, $CHCl_3$). 80% ee (HPLC condition: Chiralcel OD-H column, *n*-Hexane/*i*-PrOH = 90:10, flow rate = 1.0 mL/min, wavelength = 254 nm, t_R = 13.642 min for minor isomer, t_R =16.073 min for major isomer).

(*R*a,*R*)-1-(5-fluoro-2-methyl-1H-indol-1-yl)-2-(methylthio)-6-phenyl-5,6-dihydropyrimidin-4(1H)-one (3n)

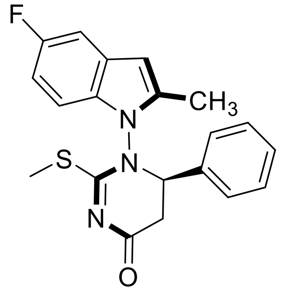

Yellow solid. **MP**: 112-114°C. **^{1}H NMR** (500 MHz, $CDCl_3$) δ 7.37 – 7.30 (m, 1H), 7.24 (dd, J = 14.8, 7.2 Hz, 2H), 7.16 (ddd, J = 9.3, 6.0, 3.3 Hz, 2H), 7.02 (dd, J = 8.5, 2.8 Hz, 3H), 6.02 (s, 1H), 5.03 (t, J = 6.9 Hz, 1H), 3.31 (dd, J = 15.6, 7.2 Hz, 1H), 3.24 (dd, J = 15.6, 6.5 Hz, 1H), 2.41 (s, 3H), 1.67 (s, 3H). **^{13}C NMR** (126 MHz, $CDCl_3$) δ 176.1, 172.6, 159.0 (d, J = 237.2 Hz). 139.1, 135.5, 130.2, 129.7, 128.9, 127.8, 110.6, 110.4, 108.9 (d, J = 9.9 Hz), 106.2 (d, J = 24.0 Hz), 100.8 (d, J = 3.8 Hz), 62.2, 37.5, 14.3, 11.3. **^{19}F NMR** (471 MHz, $CDCl_3$) δ -122.19. **HRMS (ESI)** m/z calcd for $[C_{20}H_{18}FN_3OS, M+Na]^+$: 390.1047; found: 390.1056.

Specific Rotation: $[\alpha]^{25}_{D}$ = +89.9 (*c* = 1.0, $CHCl_3$). 90% ee (HPLC condition: Chiralcel AD-H column, *n*-Hexane/*i*-PrOH = 80:20, flow rate = 1.0 mL/min, wavelength = 254 nm, t_R = 8.185 min for major isomer, t_R =12.037 min for minor isomer).

(*R*a,*R*)-1-(5-chloro-2-methyl-1H-indol-1-yl)-2-(methylthio)-6-phenyl-5,6-dihydropyrimidin-4(1H)-one (3o)

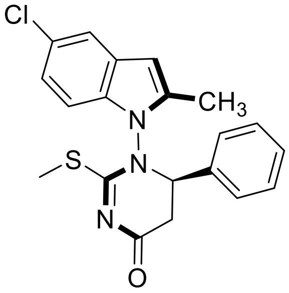

Yellow solid. **MP**: 125-127 °C. **^{1}H NMR** (500 MHz, $CDCl_3$) δ 7.47 (d, J = 1.9 Hz, 1H), 7.32 (t, J = 7.3 Hz, 1H), 7.29 – 7.22 (m, 3H), 7.18 (d, J = 8.5 Hz, 1H), 7.01 (d, J = 7.6 Hz, 2H), 6.01 (s, 1H), 5.04 (t, J = 6.9 Hz, 1H), 3.28 (qd, J = 15.6, 7.0 Hz, 2H), 2.41 (s, 3H), 1.67 (s, 3H). **^{13}C NMR** (126 MHz, $CDCl_3$) δ 176.0, 172.6, 138.8, 135.4, 132.2, 129.7, 128.9, 127.9, 127.8, 127.4, 122.7, 120.3, 109.3, 100.4, 37.5, 14.3, 11.3. **HRMS (ESI)** m/z calcd for $[C_{20}H_{18}ClN_3OS, M+Na]^+$: 406.0751; found: 406.0749.

Specific Rotation: $[\alpha]^{25}_{D}$ = +78.5 (*c* = 1.0, $CHCl_3$). 91% ee (HPLC condition: Chiralcel IA column, *n*-Hexane/*i*-PrOH = 70:30, flow rate = 1.0 mL/min, wavelength = 254 nm, t_R = 6.645 min for major isomer, t_R = 8.322 min for minor isomer).

(*R*$_a$,*R*)-1-(2,5-dimethyl-1H-indol-1-yl)-2-(methylthio)-6-phenyl-5,6-dihydropyrimidin-4(1H)-one (3p)

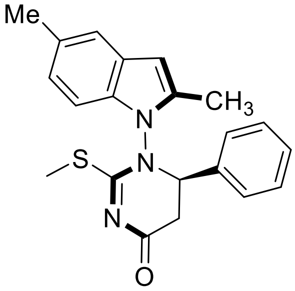

White solid. **MP**: 115-117 °C. **^{1}H NMR** (500 MHz, $CDCl_3$) δ 7.30 (d, J = 7.5 Hz, 2H), 7.23 (t, J = 7.5 Hz, 2H), 7.12 (q, J = 8.2 Hz, 2H), 7.03 (d, J = 7.4 Hz, 2H), 5.99 (s, 1H), 5.20 – 4.92 (m, 1H), 3.33 (dd, J = 15.5, 7.4 Hz, 1H), 3.21 (dd, J = 15.5, 5.9 Hz, 1H), 2.46 (s, 3H), 2.39 (s, 3H), 1.63 (s, 3H). **^{13}C NMR** (126 MHz, $CDCl_3$) δ 176.6, 173.0, 137.5, 136.3, 132.3, 131.5, 129.8, 129.1, 128.1, 127.5, 124.1, 120.9, 108.2, 100.7, 62.3, 37.7, 21.6, 14.5, 11.5. **HRMS (ESI)** m/z calcd for $[C_{21}H_{21}N_3OS, M+Na]^+$: 386.1297; found: 386.1302.

Specific Rotation: $[\alpha]^{25}_{D}$ = +113.7 (*c* = 1.0, $CHCl_3$). 90% ee (HPLC condition: Chiralcel AD-H column, *n*-Hexane/*i*-PrOH = 80:20, flow rate = 1.0 mL/min, wavelength = 254 nm, t_R = 6.467 min for major isomer, t_R = 8.330 min for minor isomer).

(*R*$_a$,*R*)-1-(2,3-dimethyl-1H-indol-1-yl)-2-(methylthio)-6-phenyl-5,6-dihydropyrimidin-4(1H)-one (3q)

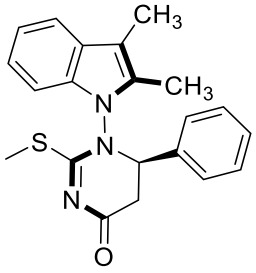

White solid. **MP**: 112-114 °C. **^{1}H NMR** (500 MHz, $CDCl_3$) δ 7.62 – 7.42 (m, 1H), 7.32 – 7.28 (m, 2H), 7.24 – 7.19 (m, 4H), 7.00 (d, J = 7.5 Hz, 2H), 4.95 (dd, J = 7.6, 5.4 Hz, 1H), 3.33 (dd, J = 15.5, 7.6 Hz, 1H), 3.16 (dd, J = 15.5, 5.3 Hz, 1H), 2.39 (s, 3H), 2.05 (s, 3H), 1.56 (s, 3H). **^{13}C NMR** (126 MHz, $CDCl_3$) δ 176.3, 172.7, 136.1, 133.4, 132.9, 129.4, 128.7, 128.0, 127.8, 122.5, 121.4, 118.8, 108.1, 108.1, 62.0, 37.6, 14.2, 8.9, 8.5. **HRMS (ESI)** m/z calcd for $[C_{21}H_{21}N_3OS, M+Na]^+$: 386.1297; found: 386.1306.

Specific Rotation: $[\alpha]^{25}_{D}$ = +135.9 (*c* = 1.0, $CHCl_3$). 91% ee (HPLC condition: Chiralcel AD-H column, *n*-Hexane/*i*-PrOH = 80:20, flow rate = 1.0 mL/min, wavelength = 254 nm, *t*R = 6.250 min for major isomer, *t*R = 7.362 min for minor isomer).

(*R*a,*R*)-2-(methylthio)-6-phenyl-1-(1,2,3,4-tetrahydro-9H-carbazol-9-yl)-5,6-dihydropyrimidin-4(1H)-one (3r)

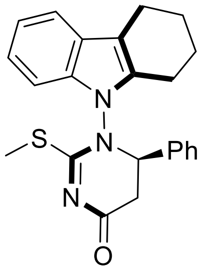

Yellow solid. **MP**: 121-123 °C. **^{1}H NMR** (500 MHz, $CDCl_3$) δ 7.45 (d, J = 7.6 Hz, 1H), 7.33 – 7.26 (m, 3H), 7.21 (t, J = 7.4 Hz, 3H), 7.01 – 6.91 (m, 2H), 4.95 (dd, J = 7.6, 5.5 Hz, 1H), 3.32 (dd, J = 15.6, 7.6 Hz, 1H), 3.16 (dd, J = 15.6, 5.6 Hz, 1H), 2.56 (q, J = 7.5, 5.5 Hz, 1H), 2.51 – 2.44 (m, 1H), 2.39 (s, 3H), 2.27 – 2.20 (m, 1H), 1.70 – 1.52 (m, 3H), 1.46 – 1.30 (m, 2H). **^{13}C NMR** (126 MHz, $CDCl_3$) δ 176.1, 172.8, 136.2, 136.1, 133.8, 129.3, 128.7, 127.7, 127.0, 122.4, 121.4, 118.6, 110.9, 108.3, 61.8, 37.5, 22.4, 22.3, 21.1, 20.5, 14.2. **HRMS (ESI)** m/z calcd for $[C_{23}H_{23}N_3OS, M+Na]^+$: 412.1454; found: 412.1463.

Specific Rotation: $[\alpha]^{25}_{D}$ = +154.1 (*c* = 1.0, $CHCl_3$). 91% ee (HPLC condition: Chiralcel IA column, *n*-Hexane/*i*-PrOH = 80:20, flow rate = 1.0 mL/min, wavelength = 254 nm, t_R = 7.148 min for major isomer, t_R = 8.142 min for minor isomer).

(*R*a,*R*)-1-(2-methyl-5-phenyl-1H-pyrrol-1-yl)-2-(methylthio)-6-phenyl-5,6-dihydropyrimidin-4(1H)-one (3s)

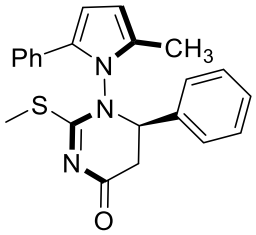

Yellow solid. **MP**: 112-114 °C. **^{1}H NMR** (500 MHz, $CDCl_3$) δ 7.21 – 7.18 (m, 3H), 7.11 (t, J = 7.4 Hz, 1H), 7.02 (dd, J = 6.7, 3.0 Hz, 2H), 6.90 (t, J = 7.6 Hz, 2H), 6.55 (d, J = 7.6 Hz, 2H), 5.96 – 5.87 (m, 2H), 4.88 (dd, J = 11.8, 5.1 Hz, 1H), 3.07 (dd, J = 15.5, 11.7 Hz, 1H), 2.88 (dd, J = 15.5, 5.1 Hz, 1H), 2.58 (s, 3H), 2.38 (s, 3H). **^{13}C NMR** (126 MHz, $CDCl_3$) δ 177.2, 173.3, 133.6, 132.3, 131.3, 129.3, 129.0, 128.1, 128.1, 127.9, 127.1, 126.8, 107.9, 107.3, 64.4, 37.8, 14.7, 12.1. **HRMS (ESI)** m/z calcd for [$C_{22}H_{21}N_3OS$, M+Na]$^+$: 398.1297; found: 398.1306.

Specific Rotation: $[\alpha]^{25}_{D}$ = +122.9 (*c* = 1.0, $CHCl_3$). 88% ee (HPLC condition: Chiralcel AD-H column, *n*-Hexane/*i*-PrOH = 90:10, flow rate = 1.0 mL/min, wavelength = 254 nm, t_R = 9.597 min for minor isomer, t_R = 12.793 min for major isomer).

(*R*$_a$,*R*)-6-(4-chlorophenyl)-1-(2-methyl-5-phenyl-1H-pyrrol-1-yl)-2-(methylthio)-5,6-dihydropyrimidin-4(1H)-one (3t)

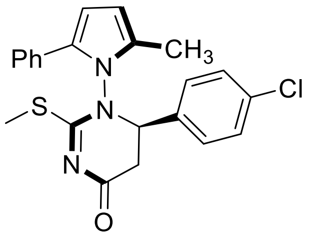

Yellow solid. **MP**: 115-117 °C. **^{1}H NMR** (500 MHz, $CDCl_3$) δ 7.23 – 7.16 (m, 3H), 7.03 (dt, J = 7.4, 3.6 Hz, 2H), 6.83 (d, J = 8.4 Hz, 2H), 6.48 (d, J = 8.4 Hz, 2H), 6.02 – 5.90 (m, 2H), 4.78 (dd, J = 9.9, 6.0 Hz, 1H), 2.95 (qd, J = 15.4, 8.0 Hz, 2H), 2.58 (s, 3H), 2.36 (s, 3H). **^{13}C NMR** (126 MHz, $CDCl_3$) δ 176.8, 172.6, 135.0, 133.7, 131.5, 131.2, 129.2, 129.0, 128.3, 128.1, 127.1, 126.9, 108.1, 107.7, 63.6, 37.6, 14.7, 12.0. **HRMS (ESI)** m/z calcd for [$C_{22}H_{20}ClN_3OS$, M+Na]$^+$: 432.0908; found: 432.0914.

Specific Rotation: $[\alpha]^{25}_{D}$ = +111.6 (*c* = 1.0, $CHCl_3$). 90% ee (HPLC condition: Chiralcel IA column, *n*-Hexane/*i*-PrOH = 90:10, flow rate = 1.0 mL/min, wavelength = 254 nm, t_R = 12.342 min for minor isomer, t_R = 15.575 min for major isomer).

(*R*$_a$,*R*)-1-(2-methyl-5-phenyl-1H-pyrrol-1-yl)-2-(methylthio)-6-(p-tolyl)-5,6-dihydropyrimidin-4(1H)-one (3u)

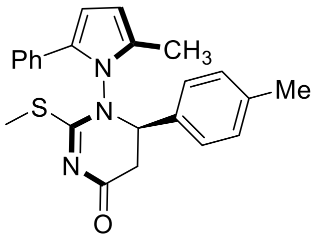

Yellow solid. **MP**: 122-124 °C. **^{1}H NMR** (500 MHz, $CDCl_3$) δ 7.23 – 7.10 (m, 3H), 7.02 (ddd, J = 5.5, 2.9, 1.6 Hz, 2H), 6.68 (d, J = 7.7 Hz, 2H), 6.49 – 6.41 (m, 2H), 5.96 – 5.88 (m, 2H), 4.83 (dd, J = 11.4, 5.2 Hz, 1H), 3.05 (dd, J = 15.4, 11.4 Hz, 1H), 2.88 (dd, J = 15.5, 5.3 Hz, 1H), 2.57 (s, 3H), 2.36 (s, 3H), 2.20 (s, 3H).**^{13}C NMR** (126 MHz, $CDCl_3$) δ 177.0, 173.4, 138.9, 133.6, 131.4, 129.5, 129.3, 128.8, 127.9, 127.8, 127.1, 126.6, 107.8, 107.3, 64.1, 37.9, 21.0, 14.7, 12.0. **HRMS (ESI)** m/z calcd for $[C_{23}H_{23}N_3OS, M+Na]^+$: 412.1454; found: 412.1465.

Specific Rotation: $[\alpha]^{25}_{D}$ = +134.6 (*c* = 1.0, $CHCl_3$). 90% ee (HPLC condition: Chiralcel IA column, *n*-Hexane/*i*-PrOH = 90:10, flow rate = 1.0 mL/min, wavelength = 254 nm, t_R = 11.908 min for minor isomer, t_R = 14.113 min for major isomer).

(*R*a,*R*)-6-(furan-2-yl)-1-(2-methyl-5-phenyl-1H-pyrrol-1-yl)-2-(methylthio)-5,6-dihydropyrimidin-4(1H)-one (3v)

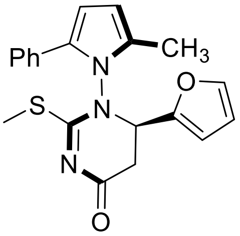

Yellow solid. **MP**: 122-124 °C. **^{1}H NMR** (500 MHz, $CDCl_3$) δ 7.52 – 7.48 (m, 2H), 7.43 (t, J = 7.6 Hz, 2H), 7.35 (q, J = 2.7, 2.2 Hz, 2H), 6.27 (d, J = 3.9 Hz, 1H), 6.25 (dd, J = 3.4, 1.8 Hz, 1H), 6.05 (d, J = 3.3 Hz, 1H), 5.92 – 5.78 (m, 1H), 4.39 (t, J = 6.7 Hz, 1H), 2.91 (dd, J = 15.5, 6.2 Hz, 1H), 2.68 (dd, J = 15.5, 7.2 Hz, 1H), 2.55 (s, 3H), 1.74 (s, 3H). **^{13}C NMR** (126 MHz, $CDCl_3$) δ 174.8, 172.1, 148.2, 143.3, 132.2, 131.3, 131.1, 129.0, 127.7, 127.4, 110.7, 110.6, 108.7, 106.5, 56.5, 35.2, 14.3, 10.3. **HRMS (ESI)** m/z calcd for $[C_{20}H_{19}N_3O_2S, M+Na]^+$: 388.1090; found: 388.1098.

Specific Rotation: $[\alpha]^{25}_{D}$ = +214.7 (*c* = 1.0, $CHCl_3$). 85% ee (HPLC condition: Chiralcel AD-H column, *n*-Hexane/*i*-PrOH = 90:10, flow rate = 1.0 mL/min, wavelength = 254 nm, t_R = 12.528 min for minor isomer, t_R = 14.680 min for major isomer)

(*S*)-3-(2-methyl-5-phenyl-1H-pyrrol-1-yl)-1-(4-nitrophenyl)-6-phenyl-2-thioxo-2,3-dihydropyrimidin-4(1H)-one (7a)

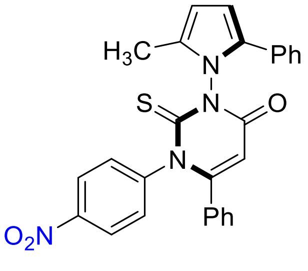

Yellow solid. **MP**: 180-182 °C. **^{1}H NMR** (500 MHz, $CDCl_3$) δ 8.05 (d, *J* = 9.3 Hz, 2H), 7.44 – 7.30 (m, 5H), 7.28 – 7.24 (m, 1H), 7.21 (dd, *J* = 8.3, 6.6 Hz, 3H), 7.06 – 6.98 (m, 2H), 6.84 (s, 1H), 6.36 (d, *J* = 3.8 Hz, 1H), 6.17 (dd, *J* = 3.7, 1.2 Hz, 1H), 6.13 (s, 1H), 2.25 (d, *J* = 1.0 Hz, 3H). **^{13}C NMR** (126 MHz, $CDCl_3$)

δ 178.5, 157.9, 155.1, 147.0, 146.0, 132.1, 131.8, 131.7, 130.7, 130.3, 129.2, 128.5, 128.3, 128.1, 128.1, 127.2, 124.3, 107.8, 106.7, 106.0, 11.3. **HRMS (ESI)** m/z calcd for $[C_{27}H_{20}N_4O_3S, M+Na]^+$: 503.1148; found: 503.1147.

Specific Rotation: $[\alpha]^{25}_{D}$ = +47.4 (*c* = 1.0, $CHCl_3$). 97% ee (HPLC condition: Chiralcel OD- H column, *n*-Hexane/*i*-PrOH = 80:20, flow rate = 1.0 mL/min, wavelength = 254 nm, t_R = 23.703 min for major isomer, t_R = 36.253 min for minor isomer).

(*S*)-3-(2-methyl-5-phenyl-1H-pyrrol-1-yl)-6-phenyl-2-thioxo-1-(4-(trifluoromethyl)phenyl)-2,3-dihydropyrimidin-4(1H)-one (7b)

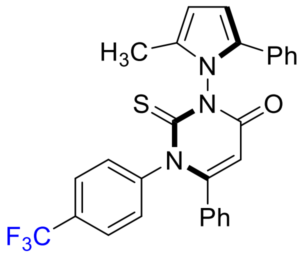

Yellow solid. **MP**: 180-182 °C. **^{1}H NMR** (500 MHz, $CDCl_3$) δ 7.47 – 7.41 (m, 4H), 7.40 – 7.30 (m, 3H), 7.24 – 7.10 (m, 4H), 7.03 – 6.95 (m, 2H), 6.80 (s, 1H), 6.36 (d, *J* = 3.7 Hz, 1H), 6.17 (d, *J* = 3.7 Hz, 1H), 6.11 (s, 1H), 2.26 (s, 3H). **^{13}C NMR** (126 MHz, $CDCl_3$) δ 178.8, 158.1, 155.5, 143.8, 132.5, 131.8, 130.7 (d, *J* = 33.1 Hz), 130.0, 130.0, 129.2, 128.4, 128.3, 128.2, 128.1, 127.2, 126.2 (d, *J* = 15.4 Hz), 124.3, 122.1, 107.8, 106.6, 105.8, 11.4. **^{19}F NMR** (471 MHz, $CDCl_3$) δ -62.86. **HRMS (ESI)** m/z calcd for $[C_{28}H_{20}F_3N_3OS, M+Na]^+$: 526.1171; found: 526.1182.

Specific Rotation: $[\alpha]^{25}_{D}$ = +56.3 (*c* = 1.0, $CHCl_3$). 90% ee (HPLC condition: Chiralcel IC column, *n*-Hexane/*i*-PrOH = 80:20, flow rate = 1.0 mL/min, wavelength = 254 nm, t_R = 8.692 min for minor isomer, t_R = 10.245 min for major isomer).

(*S*)-1-(3,5-bis(trifluoromethyl)phenyl)-3-(2-methyl-5-phenyl-1H-pyrrol-1-yl)-6-phenyl-2-thioxo-2,3-dihydropyrimidin-4(1H)-one (7c)

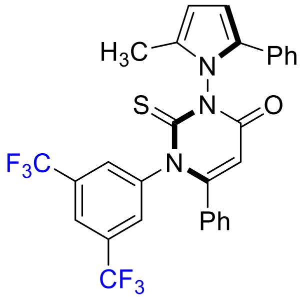

Yellow solid. **MP**: 180-182 °C. **^{1}H NMR** (500 MHz, $CDCl_3$) δ 7.63 (s, 1H), 7.50 – 7.30 (m, 6H), 7.27 – 7.10 (m, 3H), 7.01 – 6.84 (m, 3H), 6.36 (d, *J* = 3.8 Hz, 1H), 6.17 (d, *J* = 8.7 Hz, 2H), 2.27 (s, 3H). **^{13}C NMR** (126 MHz, $CDCl_3$) δ 178.7, 158.0, 155.1, 142.2, 132.5 (d, *J* = 60.2 Hz), 132.5 (d, *J* = 7.9 Hz), 132.1, 131.9, 131.8, 130.2 (d, *J* = 21.2 Hz), 129.4, 128.7, 128.5, 128.3, 128.1, 127.4, 122.5 – 122.2 (m), 122.3 (d,

J = 273.1 Hz), 107.9, 106.7, 106.0, 11.4. **^{19}F NMR** (471 MHz, $CDCl_3$) δ -63.14, -63.20, -63.24. **HRMS (ESI)** m/z calcd for [$C_{29}H_{19}F_6N_3OS$, M+Na]$^+$: 594.1045; found: 594.1055.

Specific Rotation: $[\alpha]^{25}_{D}$ = +77.3 (*c* = 1.0, $CHCl_3$). 94% ee (HPLC condition: Chiralcel IA column, *n*-Hexane/*i*-PrOH = 90:10, flow rate = 1.0 mL/min, wavelength = 254 nm, t_R = 5.608 min for minor isomer, t_R = 6.433 min for major isomer).

(*S*)-3-(2-methyl-5-(p-tolyl)-1H-pyrrol-1-yl)-1-(4-nitrophenyl)-6-phenyl-2-thioxo-2,3-dihydropyrimidin-4(1H)-one (7d)

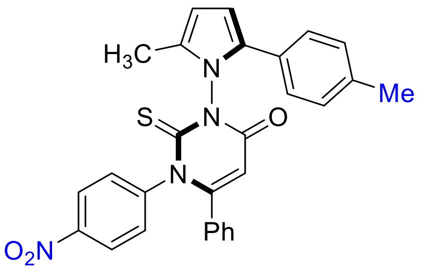

Yellow solid. **MP**: 172-174 °C. **^{1}H NMR** (500 MHz, $CDCl_3$) δ 8.08 – 8.03 (m, 2H), 7.33 – 7.24 (m, 4H), 7.23 – 7.17 (m, 4H), 7.07 – 6.99 (m, 2H), 6.92 (s, 1H), 6.31 (d, *J* = 3.8 Hz, 1H), 6.15 (d, *J* = 3.7 Hz, 1H), 6.12 (s, 1H), 2.39 (s, 3H), 2.24 (s, 3H). **^{13}C NMR** (126 MHz, $CDCl_3$) δ 178.6, 157.8, 155.0, 147.1, 146.1, 137.0, 132.2, 131.8, 130.8, 130.3, 129.0, 128.9, 128.8, 128.6, 128.2, 127.9, 124.3, 107.5, 106.6, 106.1, 21.2, 11.3. **HRMS (ESI)** m/z calcd for [$C_{28}H_{22}N_4O_3S$, M+Na]$^+$: 517.1305; found: 517.1305.

Specific Rotation: $[\alpha]^{25}_{D}$ = +126.8 (*c* = 1.0, $CHCl_3$). 92% ee (HPLC condition: Chiralcel AD-H column, *n*-Hexane/*i*-PrOH = 70:30, flow rate = 1.0 mL/min, wavelength = 254 nm, t_R = 11.867 min for major isomer, t_R = 14.192 min for minor isomer).

(*S*)-3-(2-(4-methoxyphenyl)-5-methyl-1H-pyrrol-1-yl)-1-(4-nitrophenyl)-6-phenyl-2-thioxo-2,3-dihydropyrimidin-4(1H)-one (7e)

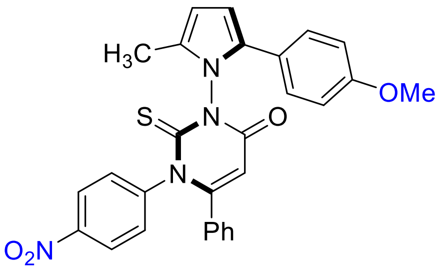

Yellow solid. **MP**: 172-174 °C. **^{1}H NMR** (500 MHz, $CDCl_3$) δ 8.08 – 8.03 (m, 2H), 7.37 – 7.33 (m, 2H), 7.26 (d, *J* = 5.4 Hz, 2H), 7.21 (t, *J* = 7.6 Hz, 3H), 7.04 (d, *J* = 7.3 Hz, 2H), 6.92 (d, *J* = 8.5 Hz, 2H), 6.27 (d, *J* = 3.7 Hz, 1H), 6.15 (d, *J* = 3.7 Hz, 1H), 6.12 (s, 1H), 3.85 (s, 3H), 2.24 (s, 3H). **^{13}C NMR** (126 MHz, $CDCl_3$) δ 178.6, 159.0, 157.9, 155.0, 147.1, 146.0, 132.2, 131.6, 130.8, 130.3, 129.7, 128.6, 128.4, 128.2, 124.4, 124.3, 113.7, 107.2, 106.5, 106.1, 55.3, 11.4. **HRMS (ESI)** m/z calcd for [$C_{28}H_{22}N_4O_4S$, M+Na]$^+$: 533.1254; found: 533.1260.

Specific Rotation: $[\alpha]^{25}_{D}$ = +111.9 (*c* = 1.0, $CHCl_3$). 93% ee (HPLC condition: Chiralcel IA column, *n*-Hexane/*i*-PrOH = 70:30, flow rate = 1.0 mL/min, wavelength = 254 nm, t_R = 12.733 min for minor isomer, t_R = 14.517 min for major isomer).

(*S*)-3-(2-(4-fluorophenyl)-5-methyl-1H-pyrrol-1-yl)-1-(4-nitrophenyl)-6-phenyl-2-thioxo-2,3-dihydropyrimidin-4(1H)-one (7f)

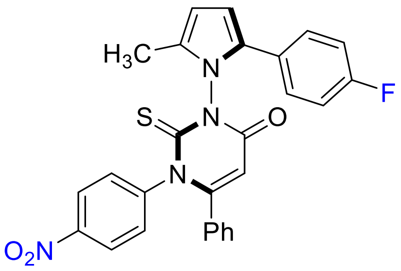

Yellow solid. **MP**: 177-179 °C. **^{1}H NMR** (500 MHz, $CDCl_3$) δ 8.07 (dt, *J* = 8.8, 1.2 Hz, 2H), 7.41 – 7.37 (m, 2H), 7.25 – 7.19 (m, 4H), 7.10 – 7.02 (m, 4H), 6.92 (d, *J* = 8.1 Hz, 1H), 6.30 (d, *J* = 3.7 Hz, 1H), 6.16 (dd, *J* = 3.8, 1.2 Hz, 1H), 6.13 (s, 1H), 2.23 (s, 3H). **^{13}C NMR (126 MHz, $CDCl_3$)** δ 178.5, 162.2 (d, *J* = 246.4 Hz), 157.8, 155.2, 146.5 (d, *J* = 148.5 Hz), 132.1, 130.9, 130.7, 130.4, 130.1 (d, *J* = 8.2 Hz), 128.9, 128.6, 128.1, 127.9 (d, *J* = 2.9 Hz), 124.4, 115.3, 115.1, 107.9, 106.7, 106.0, 11.3. **^{19}F NMR** (471 MHz, $CDCl_3$) δ -114.30. **HRMS (ESI)** m/z calcd for $[C_{27}H_{19}FN_4O_3S, M+Na]^+$: 521.1054; found: 521.1064.

Specific Rotation: $[\alpha]^{25}_{D}$ = +134.6 (*c* = 1.0, $CHCl_3$). 92% ee (HPLC condition: Chiralcel OD-H column, *n*-Hexane/*i*-PrOH = 70:30, flow rate = 1.0 mL/min, wavelength = 254 nm, t_R = 14.037 min for minor isomer, t_R = 16.818 min for major isomer).

(*S*)-3-(2-(4-chlorophenyl)-5-methyl-1H-pyrrol-1-yl)-1-(4-nitrophenyl)-6-phenyl-2-thioxo-2,3-dihydropyrimidin-4(1H)-one (7g)

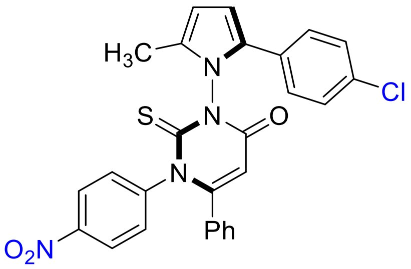

Yellow solid. **MP**: 177-179 °C. **^{1}H NMR** (500 MHz, $CDCl_3$) δ 8.07 (dt, *J* = 8.7, 1.1 Hz, 2H), 7.35 (s, 4H), 7.28 – 7.17 (m, 4H), 7.10 – 7.03 (m, 2H), 6.97 (s, 1H), 6.34 (d, *J* = 3.7 Hz, 1H), 6.17 (dd, *J* = 3.7, 1.1 Hz, 1H), 6.14 (s, 1H), 2.23 (s, 3H). **^{13}C NMR** (126 MHz, $CDCl_3$) δ 178.4, 157.8, 155.3, 147.1, 145.9, 133.1, 132.0, 130.8, 130.4, 130.2, 129.4, 129.2, 128.6, 128.5, 128.2, 124.4, 108.3, 107.0, 106.0, 11.3. **HRMS (ESI)** m/z calcd for $[C_{27}H_{19}ClN_4O_3S, M+H]^+$: 515.0939; found: 515.0950.

Specific Rotation: $[\alpha]^{25}_{D}$ = +189.7 (*c* = 1.0, $CHCl_3$). 92% ee (HPLC condition: Chiralcel IA column, *n*-Hexane/*i*-PrOH = 70:30, flow rate = 1.0 mL/min, wavelength = 254 nm, t_R = 9.928 min for minor isomer, t_R = 11.235 min for major isomer).

(*S*)-3-(2-(4-bromophenyl)-5-methyl-1H-pyrrol-1-yl)-1-(4-nitrophenyl)-6-phenyl-2-thioxo-2,3-dihydropyrimidin-4(1H)-one (7h)

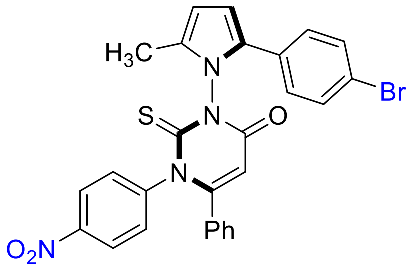

Yellow solid. **MP**: 177-179 °C. **^{1}H NMR** (500 MHz, $CDCl_3$) δ 8.08 (d, *J* = 8.5 Hz, 2H), 7.50 (d, *J* = 8.0 Hz, 2H), 7.33 – 7.18 (m, 6H), 7.07 (d, *J* = 7.5 Hz, 2H), 6.98 (s, 1H), 6.34 (d, *J* = 3.8 Hz, 1H), 6.19 – 6.09 (m, 2H), 2.23 (s, 3H). **^{13}C NMR** (126 MHz, $CDCl_3$) δ 178.4, 157.8, 155.3, 147.1, 145.9, 132.0, 131.5, 130.8, 130.8, 130.7, 130.4, 129.5, 128.6, 128.2, 124.4, 121.3, 108.3, 107.0, 106.0, 11.3. **HRMS (ESI)** m/z calcd for [$C_{27}H_{19}BrN_4O_3S$, M+Na]$^+$: 581.0253; found: 581.0251.

Specific Rotation: $[\alpha]^{25}_{D}$ = +113.7 (*c* = 1.0, $CHCl_3$). 92% ee (HPLC condition: Chiralcel OD-H column, *n*-Hexane/*i*-PrOH = 70:30, flow rate = 1.0 mL/min, wavelength = 254 nm, t_R = 16.092 min for minor isomer, t_R = 19.898 min for major isomer).

(*S*)-3-(2-methyl-5-(4-(trifluoromethyl)phenyl)-1H-pyrrol-1-yl)-1-(4-nitrophenyl)-6-phenyl-2-thioxo-2,3-dihydropyrimidin-4(1H)-one (7i)

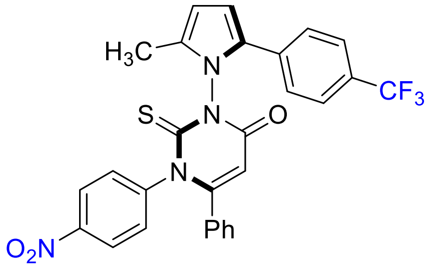

Yellow solid. **MP**: 177-179 °C. **^{1}H NMR** (500 MHz, $CDCl_3$) δ 8.07 (d, *J* = 8.6 Hz, 2H), 7.64 (d, *J* = 8.1 Hz, 2H), 7.53 (d, *J* = 8.0 Hz, 2H), 7.24 (dt, *J* = 15.0, 6.7 Hz, 4H), 7.10 – 7.04 (m, 2H), 6.94 (s, 1H), 6.44 (d, *J* = 3.9 Hz, 1H), 6.20 (d, *J* = 3.8 Hz, 1H), 6.16 (s, 1H), 2.25 (s, 3H). **^{13}C NMR** (126 MHz, $CDCl_3$) δ 178.3, 157.7, 155.4, 147.1, 145.9, 135.2, 132.0, 130.7, 130.6, 130.4, 130.3, 128.6, 128.1, 127.7, 125.4 (q, *J* = 3.7 Hz), 124.4, 109.2, 107.3, 106.0, 11.3. **^{19}F NMR** (471 MHz, $CDCl_3$) δ -62.32. **HRMS (ESI)** m/z calcd for [$C_{28}H_{19}F_3N_4O_3S$, M+Na]$^+$: 571.1022; found: 571.1026.

Specific Rotation: $[\alpha]^{25}_{D}$ = +157.8 (*c* = 1.0, $CHCl_3$). 88% ee (HPLC condition: Chiralcel IA column, *n*-Hexane/*i*-PrOH = 70:30, flow rate = 1.0 mL/min, wavelength = 254 nm, t_R = 7.935 min for minor isomer, t_R = 9.427 min for major isomer).

(*S*)-4-(5-methyl-1-(3-(4-nitrophenyl)-6-oxo-4-phenyl-2-thioxo-3,6-dihydropyrimidin-1(2H)-yl)-1H-pyrrol-2-yl)benzonitrile (7j)

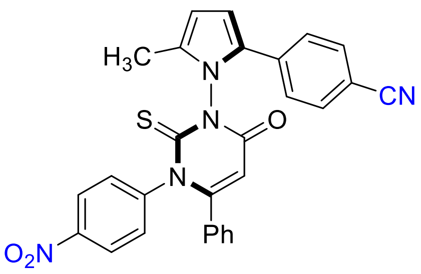

Yellow solid. **MP**: 177-179 °C. **^{1}H NMR** (500 MHz, $CDCl_3$) δ 8.13 – 8.06 (m, 2H), 7.66 (d, *J* = 8.1 Hz, 2H), 7.50 (d, *J* = 8.2 Hz, 2H), 7.28 – 7.23 (m, 4H), 7.09 (d, *J* = 7.1 Hz, 2H), 7.02 (s, 1H), 6.47 (d, *J* = 3.9 Hz, 1H), 6.22 (d, *J* = 3.9 Hz, 1H), 6.18 (s, 1H), 2.24 (s, 3H). **^{13}C NMR** (126 MHz, $CDCl_3$) δ 178.2, 157.7, 155.6, 147.2, 145.8, 136.1, 132.3, 131.9, 131.2, 130.7, 130.5, 130.3, 128.7, 128.2, 127.4, 124.5, 118.9, 110.0, 107.8, 106.0, 28.1, 11.4. **HRMS (ESI)** m/z calcd for $[C_{28}H_{19}N_5O_3S, M+Na]^+$: 528.1101; found: 528.1100.

Specific Rotation: $[\alpha]^{25}_{D}$ = +155.9 (*c* = 1.0, $CHCl_3$). 85% ee (HPLC condition: Chiralcel OD-H column, *n*-Hexane/*i*-PrOH = 70:30, flow rate = 1.0 mL/min, wavelength = 254 nm, t_R = 24.730 min for minor isomer, t_R = 34.615 min for major isomer).

(*S*)-3-(2-(3-methoxyphenyl)-5-methyl-1H-pyrrol-1-yl)-1-(4-nitrophenyl)-6-phenyl-2-thioxo-2,3-dihydropyrimidin-4(1H)-one (7k)

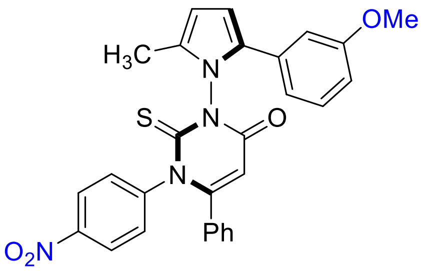

Yellow solid. **MP**: 177-179 °C. **^{1}H NMR** (500 MHz, $CDCl_3$) δ 8.07 (dt, *J* = 8.8, 1.2 Hz, 2H), 7.33 – 7.17 (m, 5H), 7.08 – 6.97 (m, 5H), 6.87 (ddd, *J* = 8.3, 2.6, 1.0 Hz, 1H), 6.38 (d, *J* = 3.8 Hz, 1H), 6.16 (dd, *J* = 3.8, 1.1 Hz, 1H), 6.13 (s, 1H), 3.81 (s, 3H), 2.25 (d, *J* = 1.0 Hz, 3H). **^{13}C NMR** (126 MHz, $CDCl_3$) δ 178.6, 159.5, 157.8, 155.1, 147.1, 146.0, 132.9, 132.2, 131.5, 130.8, 130.3, 129.5, 129.3, 128.6, 128.1, 124.4, 120.4, 113.1, 113.0, 107.9, 106.7, 106.1, 55.2, 11.3. **HRMS (ESI)** m/z calcd for $[C_{28}H_{22}N_4O_4S, M+Na]^+$: 533.1254; found: 533.1265.

Specific Rotation: $[\alpha]^{25}_{D}$ = -85.6 (*c* = 1.0, $CHCl_3$). 93% ee (HPLC condition: Chiralcel IC column, *n*-Hexane/*i*-PrOH = 70:30, flow rate = 1.0 mL/min, wavelength = 254 nm, t_R = 44.290 min for major isomer, t_R = 53.127 min for minor isomer).

(*S*)-3-(2-(3-chlorophenyl)-5-methyl-1H-pyrrol-1-yl)-1-(4-nitrophenyl)-6-phenyl-2-thioxo-2,3-dihydropyrimidin-4(1H)-one (7l)

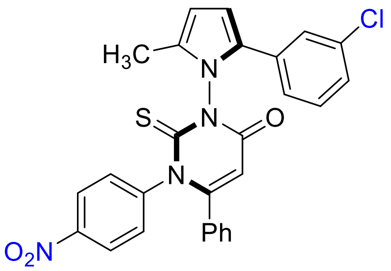

Yellow solid. **MP**: 177-179 °C. **^{1}H NMR** (500 MHz, $CDCl_3$) δ 8.09 (d, *J* = 8.5 Hz, 2H), 7.42 (d, *J* = 1.8 Hz, 1H), 7.33 – 7.21 (m, 7H), 7.14 – 7.01 (m, 3H), 6.43 (d, *J* = 3.8 Hz, 1H), 6.17 (d, *J* = 3.8 Hz, 1H), 6.12 (s, 1H), 2.26 (s, 3H). **^{13}C NMR** (126 MHz, $CDCl_3$) δ 178.6, 157.5, 155.3, 147.1, 145.9, 134.0, 133.3, 132.1, 130.8, 130.3, 130.2, 130.0, 129.9, 128.6, 128.2, 126.9, 126.5, 126.3, 124.6, 108.5, 106.9, 106.1, 11.3. **HRMS (ESI)** m/z calcd for $[C_{27}H_{19}ClN_4O_3S, M+Na]^+$: 537.0758; found: 537.0770.

Specific Rotation: $[\alpha]^{25}_{D}$ = +177.2 (*c* = 1.0, $CHCl_3$). 92% ee (HPLC condition: Chiralcel AD-H column, *n*-Hexane/*i*-PrOH = 70:30, flow rate = 1.0 mL/min, wavelength = 254 nm, t_R = 10.678 min for minor isomer, t_R = 11.733 min for major isomer).

(*S*)-3-(2-(3-bromophenyl)-5-methyl-1H-pyrrol-1-yl)-1-(4-nitrophenyl)-6-phenyl-2-thioxo-2,3-dihydropyrimidin-4(1H)-one (7m)

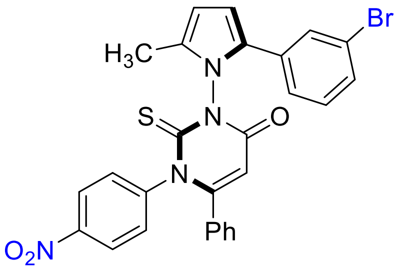

Yellow solid. **MP**: 177-179 °C. **^{1}H NMR** (500 MHz, $CDCl_3$) δ 8.10 (d, *J* = 8.4 Hz, 2H), 7.58 (t, *J* = 1.9 Hz, 1H), 7.45 – 7.41 (m, 1H), 7.38 (dt, *J* = 7.8, 1.3 Hz, 1H), 7.29 – 7.21 (m, 5H), 7.15 (d, *J* = 8.1 Hz, 1H), 7.13 – 7.09 (m, 2H), 6.43 (d, *J* = 3.8 Hz, 1H), 6.17 (d, *J* = 3.8 Hz, 1H), 6.12 (s, 1H), 2.26 (s, 3H). **^{13}C NMR** (126 MHz, $CDCl_3$) δ 178.6, 157.5, 155.3, 147.1, 145.9, 133.6, 132.1, 131.0, 130.8, 130.3, 130.2, 129.8, 129.2, 128.6, 128.2, 126.9, 124.6, 124.3, 122.2, 108.6, 106.9, 106.0, 11.3. **HRMS (ESI)** m/z calcd for $[C_{27}H_{19}BrN_4O_3S, M+H]^+$: 559.0434; found: 559.0444.

Specific Rotation: $[\alpha]^{25}_{D}$ = +47.7 (*c* = 1.0, $CHCl_3$). 90% ee (HPLC condition: Chiralcel IA column, *n*-Hexane/*i*-PrOH = 70:30, flow rate = 1.0 mL/min, wavelength = 254 nm, t_R = 9.610 min for minor isomer, t_R = 11.738 min for major isomer).

(*S*)-3-(2-(2-fluorophenyl)-5-methyl-1H-pyrrol-1-yl)-1-(4-nitrophenyl)-6-phenyl-2-thioxo-2,3-dihydropyrimidin-4(1H)-one (7n)

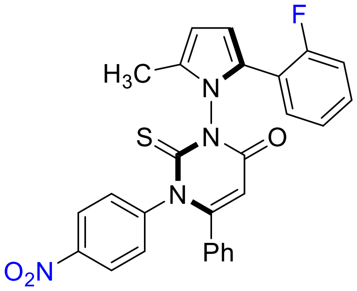

Yellow solid. **MP**: 177-179 °C. **^{1}H NMR** (500 MHz, $CDCl_3$) δ 8.12 – 7.97 (m, 2H), 7.41 – 7.30 (m, 2H), 7.26 – 7.09 (m, 6H), 7.06 – 6.98 (m, 2H), 6.81 (s, 1H), 6.47 – 6.45 (m, 1H), 6.22 (d, *J* = 3.7 Hz, 1H), 6.15 (s, 1H), 2.26 (s, 3H). **^{13}C NMR** (126 MHz, $CDCl_3$) δ 178.5, 160.9, 159.0, 157.7, 154.9, 147.0, 146.0, 132.2, 130.7, 130.3, 129.8, 129.2 (d, *J* = 8.1 Hz), 128.6, 128.1, 124.4, 124.3, 123.7 (d, *J* = 4.1 Hz), 119.6 (d, *J* = 14.0 Hz), 116.0 (d, J = 22.6 Hz), 110.4 (d, *J* = 2.8 Hz), 106.8, 106.1, 11.4. **^{19}F NMR** (471 MHz, $CDCl_3$) δ -112.69. **HRMS (ESI)** m/z calcd for $[C_{27}H_{19}FN_4O_3S, M+Na]^+$: 521.1054; found: 521.1064.

Specific Rotation: $[\alpha]^{25}_{D}$ = +76.9 (*c* = 1.0, $CHCl_3$). 99% ee (HPLC condition: Chiralcel AD-H column, *n*-Hexane/*i*-PrOH = 70:30, flow rate = 1.0 mL/min, wavelength = 254 nm, t_R = 9.115 min for major isomer, t_R = 9.838 min for minor isomer).

(*S*)-3-(2-methyl-5-(naphthalen-2-yl)-1H-pyrrol-1-yl)-1-(4-nitrophenyl)-6-phenyl-2-thioxo-2,3-dihydropyrimidin-4(1H)-one (7o)

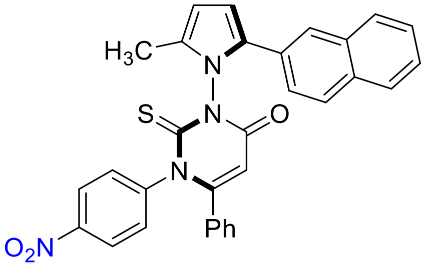

Yellow solid. **MP**: 177-179 °C. **^{1}H NMR** (500 MHz, $CDCl_3$) δ 7.98 (d, *J* = 37.4 Hz, 2H), 7.89 – 7.78 (m, 4H), 7.58 (dd, *J* = 8.5, 1.7 Hz, 1H), 7.51 (td, *J* = 5.9, 5.3, 3.2 Hz, 2H), 7.26 – 7.11 (m, 4H), 7.02 – 6.94 (m, 2H), 6.65 (s, 1H), 6.48 (d, *J* = 3.8 Hz, 1H), 6.25 – 6.19 (m, 1H), 6.15 (s, 1H), 2.29 (s, 3H). **^{13}C NMR** (126 MHz, $CDCl_3$) δ 178.5, 157.9, 155.2, 147.0, 145.9, 133.4, 132.4, 132.1, 131.7, 130.7, 130.3, 129.6, 129.2, 128.5, 128.1, 127.9, 127.9, 127.6, 126.6, 126.3, 126.1, 126.0, 124.3, 108.4, 106.9, 106.0, 11.4. **HRMS (ESI)** m/z calcd for $[C_{31}H_{22}N_4O_3S, M+Na]^+$: 553.1305; found: 553.1313.

Specific Rotation: $[\alpha]^{25}_{D}$ = +125.2 (*c* = 1.0, $CHCl_3$). 90% ee (HPLC condition: Chiralcel OD-H column, *n*-Hexane/*i*-PrOH = 70:30, flow rate = 1.0 mL/min, wavelength = 254 nm, t_R = 17.542 min for minor isomer, t_R = 21.837 min for major isomer).

(*S*)-3-(2-methyl-5-phenyl-1H-pyrrol-1-yl)-1-(4-nitrophenyl)-2-thioxo-6-(p-tolyl)-2,3-dihydropyrimidin-4(1H)-one (7p)

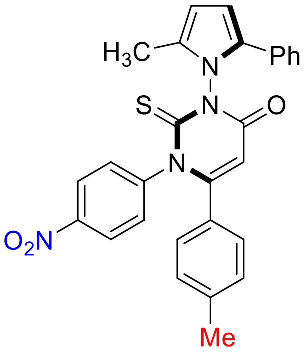

Yellow solid. **MP**: 174-176 °C. **^{1}H NMR** (500 MHz, $CDCl_3$) δ 8.09 – 8.00 (m, 2H), 7.46 – 7.30 (m, 5H), 7.23 – 7.13 (m, 1H), 6.99 (d, *J* = 7.9 Hz, 2H), 6.90 (d, *J* = 8.2 Hz, 3H), 6.35 (d, *J* = 3.7 Hz, 1H), 6.16 (dd, *J* = 3.7, 1.1 Hz, 1H), 6.11 (s, 1H), 2.25 (s, 6H). **^{13}C NMR** (126 MHz, $CDCl_3$) δ 178.6, 157.9, 155.3, 147.0, 146.1, 140.7, 131.8, 131.7, 130.7, 129.2, 129.2, 128.2, 128.0, 127.2, 124.3, 107.8, 106.6, 105.9, 21.2, 11.3. **HRMS (ESI)** m/z calcd for $[C_{28}H_{22}N_4O_3S, M+Na]^+$: 517.1305; found: 517.1309.

Specific Rotation: $[\alpha]^{25}_{D}$ = +110.8 (*c* = 1.0, $CHCl_3$). 97% ee (HPLC condition: Chiralcel IA column, *n*-Hexane/*i*-PrOH = 70:30, flow rate = 1.0 mL/min, wavelength = 254 nm, t_R = 10.187 min for major isomer, t_R = 11.550 min for minor isomer).

(*S*)-6-(4-ethylphenyl)-3-(2-methyl-5-phenyl-1H-pyrrol-1-yl)-1-(4-nitrophenyl)-2-thioxo-2,3-dihydropyrimidin-4(1H)-one (7q)

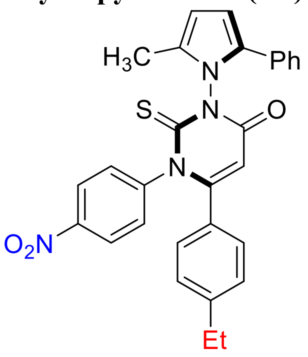

Yellow solid. **MP**: 171-173 °C. **^{1}H NMR** (500 MHz, $CDCl_3$) δ 8.10 – 8.02 (m, 2H), 7.47 – 7.34 (m, 5H), 7.21 (s, 1H), 7.01 (d, *J* = 7.9 Hz, 2H), 6.93 (d, *J* = 8.0 Hz, 3H), 6.35 (d, *J* = 3.7 Hz, 1H), 6.16 (d, *J* = 3.7 Hz, 1H), 6.11 (s, 1H), 2.54 (q, *J* = 7.6 Hz, 2H), 2.25 (s, 3H), 1.13 (t, *J* = 7.6 Hz, 3H). **^{13}C NMR** (126 MHz, $CDCl_3$) δ 178.6, 158.0, 155.3, 147.0, 146.9, 146.2, 131.8, 131.7, 130.8, 129.4, 129.2, 128.3, 128.1, 128.1, 128.0, 127.2, 124.3, 107.8, 106.6, 106.0, 28.3, 14.8, 11.3. **HRMS (ESI)** m/z calcd for $[C_{29}H_{24}N_4O_3S, M+Na]^+$: 531.1461; found: 531.1475.

Specific Rotation: $[\alpha]^{25}_{D}$ = +132.8 (*c* = 1.0, $CHCl_3$). 97% ee (HPLC condition: Chiralcel AD-H column, *n*-Hexane/*i*-PrOH = 70:30, flow rate = 1.0 mL/min, wavelength = 254 nm, t_R = 9.307 min for major isomer, t_R = 12.030 min for minor isomer).

(*S*)-6-(4-(tert-butyl)phenyl)-3-(2-methyl-5-phenyl-1H-pyrrol-1-yl)-1-(4-nitrophenyl)-2-thioxo-2,3-dihydropyrimidin-4(1H)-one (7r)

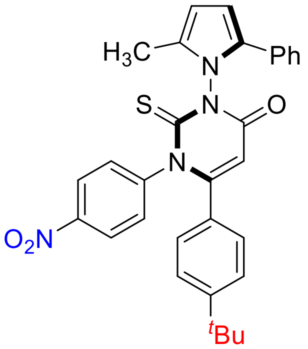

Yellow solid. **MP**: 168-170 °C. **^{1}H NMR** (500 MHz, $CDCl_3$) δ 8.08 – 8.04 (m, 2H), 7.43 – 7.29 (m, 5H), 7.23 – 7.12 (m, 3H), 6.94 (d, *J* = 8.4 Hz, 2H), 6.85 (s, 1H), 6.35 (d, *J* = 3.8 Hz, 1H), 6.17 (dd, *J* = 3.8, 1.1 Hz, 1H), 6.12 (s, 1H), 2.25 (s, 3H), 1.20 (s, 9H). **^{13}C NMR** (126 MHz, $CDCl_3$) δ 178.6, 158.0, 155.3, 153.9, 147.0, 146.2, 131.8, 131.8, 130.8, 129.2, 129.2, 128.3, 128.1, 128.0, 127.2, 125.5, 124.3, 107.8, 106.6, 106.0, 34.7, 30.9, 11.4. **HRMS (ESI)** m/z calcd for $[C_{31}H_{28}N_4O_3S, M+Na]^+$: 559.1774; found: 559.1786.

Specific Rotation: $[\alpha]^{25}_{D}$ = +39.1 (*c* = 1.0, $CHCl_3$). 95% ee (HPLC condition: Chiralcel IC column, *n*-Hexane/*i*-PrOH = 70:30, flow rate = 1.0 mL/min, wavelength = 254 nm, t_R = 15.408 min for minor isomer, t_R = 22.920 min for major isomer).

(*S*)-6-([1,1'-biphenyl]-4-yl)-3-(2-methyl-5-phenyl-1H-pyrrol-1-yl)-1-phenyl-2-thioxo-2,3-dihydropyrimidin-4(1H)-one (7s)

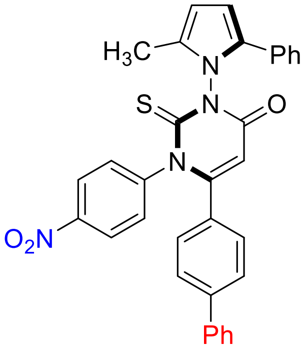

Yellow solid. **MP**: 167-169 °C. **^{1}H NMR** (500 MHz, $CDCl_3$) δ 8.11 – 8.02 (m, 2H), 7.51 – 7.32 (m, 13H), 7.15 – 7.07 (m, 2H), 6.88 (s, 1H), 6.36 (d, *J* = 3.7 Hz, 1H), 6.18 (d, *J* = 2.8 Hz, 2H), 2.26 (s, 3H). **^{13}C NMR** (126 MHz, $CDCl_3$) δ 178.6, 157.9, 154.9, 147.1, 146.1, 143.1, 138.8, 131.8, 131.8, 130.8, 130.8, 129.2, 128.9, 128.9, 128.7, 128.3, 128.1, 127.2, 127.1, 126.9, 124.5, 107.9, 106.7, 106.1, 11.4. **HRMS (ESI)** m/z calcd for $[C_{33}H_{24}N_4O_3S, M+Na]^+$: 579.1461; found: 579.1465.

Specific Rotation: $[\alpha]^{25}_{D}$ = +51.5 (*c* = 1.0, $CHCl_3$). 96% ee (HPLC condition: Chiralcel IC column, *n*-Hexane/*i*-PrOH = 70:30, flow rate = 1.0 mL/min, wavelength = 254 nm, t_R = 27.667 min for minor isomer, t_R = 39.252 min for major isomer).

(*S*)-6-(4-methoxyphenyl)-3-(2-methyl-5-phenyl-1H-pyrrol-1-yl)-1-(4-nitrophenyl)-2-thioxo-2,3-dihydropyrimidin-4(1H)-one (7t)

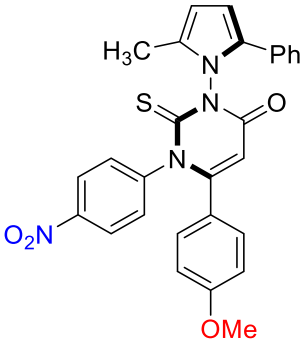

Yellow solid. **MP**: 165-167 °C. **^{1}H NMR** (500 MHz, $CDCl_3$) δ 8.08 (d, *J* = 8.6 Hz, 2H), 7.46 – 7.32 (m, 5H), 7.21 (s, 1H), 7.02 – 6.83 (m, 3H), 6.75 – 6.62 (m, 2H), 6.35 (d, *J* = 3.7 Hz, 1H), 6.17 (dd, *J* = 3.7, 1.2 Hz, 1H), 6.11 (s, 1H), 3.74 (s, 3H), 2.49 – 2.13 (m, 3H). **^{13}C NMR** (126 MHz, $CDCl_3$) δ 178.7, 160.8, 158.0, 155.1, 147.0, 146.3, 131.8, 131.8, 130.8, 129.8, 129.2, 128.3, 128.1, 127.2, 124.4, 124.2, 114.0, 107.8, 106.7, 105.9, 55.2, 11.4. **HRMS (ESI)** m/z calcd for $[C_{28}H_{22}N_4O_4S, M+Na]^+$: 533.1254; found: 533.1257.

Specific Rotation: $[\alpha]^{25}_{D}$ = +67.1 (*c* = 1.0, $CHCl_3$). 96% ee (HPLC condition: Chiralcel IA column, *n*-Hexane/*i*-PrOH = 80:20, flow rate = 1.0 mL/min, wavelength = 254 nm, t_R = 18.473 min for major isomer, t_R = 21.242 min for minor isomer).

(*S*)-6-(4-chlorophenyl)-3-(2-methyl-5-phenyl-1H-pyrrol-1-yl)-1-(4-nitrophenyl)-2-thioxo-2,3-dihydropyrimidin-4(1H)-one (7u)

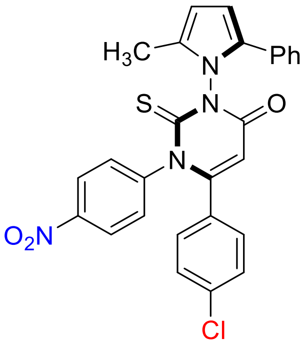

Yellow solid. **MP**: 157-159 °C. **^{1}H NMR** (500 MHz, $CDCl_3$) δ 8.09 (d, *J* = 8.5 Hz, 2H), 7.77 – 7.64 (m, 1H), 7.44 – 7.30 (m, 5H), 7.24 – 7.17 (m, 2H), 7.02 – 6.96 (m, 2H), 6.84 (s, 1H), 6.35 (d, *J* = 3.7 Hz, 1H), 6.17 (dd, *J* = 3.7, 1.2 Hz, 1H), 6.11 (s, 1H), 2.24 (s, 3H). **^{13}C NMR** (126 MHz, $CDCl_3$) δ 178.4, 157.7, 153.9, 147.2, 145.8, 136.8, 131.8, 131.7, 130.7, 130.5, 129.5, 129.1, 129.0, 128.3, 128.1, 127.3, 124.6, 107.9, 106.7, 106.2, 11.3. **HRMS (ESI)** m/z calcd for $[C_{27}H_{19}ClN_4O_3S, M+Na]^+$: 515.0939; found: 515.0951.

Specific Rotation: $[\alpha]^{25}_{D}$ = +105.0 (*c* = 1.0, $CHCl_3$). 96% ee (HPLC condition: Chiralcel IC column, *n*-Hexane/*i*-PrOH = 70:30, flow rate = 1.0 mL/min, wavelength = 254 nm, t_R = 22.287 min for minor isomer, t_R = 26.567 min for major isomer).

(*S*)-6-(4-bromophenyl)-3-(2-methyl-5-phenyl-1H-pyrrol-1-yl)-1-(4-nitrophenyl)-2-thioxo-2,3-dihydropyrimidin-4(1H)-one (7v)

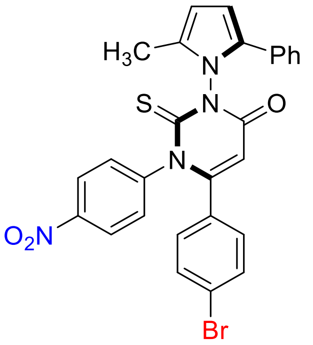

Yellow solid. **MP**: 157-159 °C. **^{1}H NMR** (500 MHz, $CDCl_3$) δ 8.09 (d, *J* = 8.6 Hz, 2H), 7.37 (dtd, *J* = 18.3, 7.6, 6.7, 3.9 Hz, 7H), 7.21 (s, 1H), 6.94 – 6.89 (m, 2H), 6.84 (s, 1H), 6.35 (d, *J* = 3.7 Hz, 1H), 6.17 (dd, *J* = 3.6, 1.2 Hz, 1H), 6.10 (s, 1H), 2.24 (s, 3H). **^{13}C NMR** (126 MHz, $CDCl_3$) δ 178.4, 157.7, 153.9, 147.2, 145.8, 132.0, 131.8, 131.7, 131.0, 130.7, 129.6, 129.1, 128.3, 128.1, 127.3, 125.1, 124.6, 107.9, 106.8, 106.2, 11.3. **HRMS (ESI)** m/z calcd for $[C_{27}H_{19}BrN_4O_3S, M+Na]^+$: 581.0253; found: 581.0264.

Specific Rotation: $[\alpha]^{25}_{D}$ = -64.8 (*c* = 1.0, $CHCl_3$). 95% ee (HPLC condition: Chiralcel IC column, *n*-Hexane/*i*-PrOH = 70:30, flow rate = 1.0 mL/min, wavelength = 254 nm, t_R = 23.828 min for minor isomer, t_R = 29.095 min for major isomer).

(*S*)-3-(2-methyl-5-phenyl-1H-pyrrol-1-yl)-1-phenyl-2-thioxo-6-(m-tolyl)-2,3-dihydropyrimidin-4(1H)-one (7w)

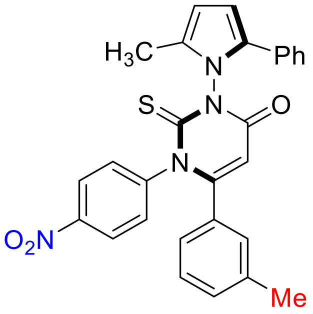

Yellow solid. **MP**: 157-159 °C. **^{1}H NMR** (500 MHz, $CDCl_3$) δ 8.11 – 7.98 (m, 2H), 7.44 – 7.30 (m, 5H), 7.25 (s, 1H), 7.10 – 7.00 (m, 2H), 6.86 (s, 2H), 6.82 – 6.74 (m, 1H), 6.35 (d, *J* = 3.7 Hz, 1H), 6.17 (dd, *J* = 3.8, 1.1 Hz, 1H), 6.12 (s, 1H), 2.24 (d, *J* = 1.0 Hz, 3H), 2.22 (s, 3H). **^{13}C NMR** (126 MHz, $CDCl_3$) δ 178.5, 157.9, 155.3, 147.0, 146.1, 138.6, 132.1, 131.8, 131.7, 131.0, 130.8, 129.1, 128.7, 128.4, 128.3, 128.1, 127.2, 125.3, 124.3, 107.8, 106.7, 105.9, 21.1, 11.3. **HRMS (ESI)** m/z calcd for $[C_{28}H_{22}N_4O_3S, M+Na]^+$: 517.1305; found: 517.1313.

Specific Rotation: $[\alpha]^{25}_{D}$ = +118.4 (*c* = 1.0, $CHCl_3$). 96% ee (HPLC condition: Chiralcel AD-H column, *n*-Hexane/*i*-PrOH = 70:30, flow rate = 1.0 mL/min, wavelength = 254 nm, t_R = 8.593 min for major isomer, t_R = 10.087 min for minor isomer).

(*S*)-6-(3-methoxyphenyl)-3-(2-methyl-5-phenyl-1H-pyrrol-1-yl)-1-(4-nitrophenyl)-2-thioxo-2,3-dihydropyrimidin-4(1H)-one (7x)

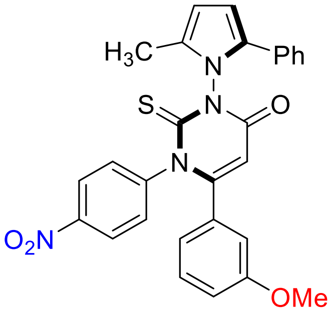

Yellow solid. **MP**: 157-159 °C. **^{1}H NMR** (500 MHz, $CDCl_3$) δ 8.09 – 8.03 (m, 2H), 7.45 – 7.30 (m, 5H), 7.22 (s, 1H), 7.10 (t, *J* = 8.0 Hz, 1H), 6.86 (s, 1H), 6.79 – 6.75 (m, 1H), 6.60 – 6.55 (m, 2H), 6.35 (d, *J* = 3.7 Hz, 1H), 6.18 – 6.15 (m, 1H), 6.13 (s, 1H), 3.68 (s, 3H), 2.24 (s, 3H). **^{13}C NMR** (126 MHz, $CDCl_3$) δ 178.5, 159.2, 157.9, 154.8, 147.0, 146.0, 133.2, 131.8, 131.7, 130.7, 129.8, 129.2, 128.3, 128.1, 127.2, 124.3, 120.4, 115.1, 114.4, 107.8, 106.6, 105.9, 55.3, 11.3. **HRMS (ESI)** m/z calcd for [$C_{28}H_{22}N_4O_4S$, M+Na]$^+$: 533.1254; found: 533.1260.

Specific Rotation: $[\alpha]^{25}_{D}$ = +58.9 (*c* = 1.0, $CHCl_3$). 95% ee (HPLC condition: Chiralcel IC column, *n*-Hexane/*i*-PrOH = 70:30, flow rate = 1.0 mL/min, wavelength = 254 nm, t_R = 34.632 min for minor isomer, t_R = 44.457 min for major isomer).

(*S*)-6-(3,5-dimethylphenyl)-3-(2-methyl-5-phenyl-1H-pyrrol-1-yl)-1-(4-nitrophenyl)-2-thioxo-2,3-dihydropyrimidin-4(1H)-one (7y)

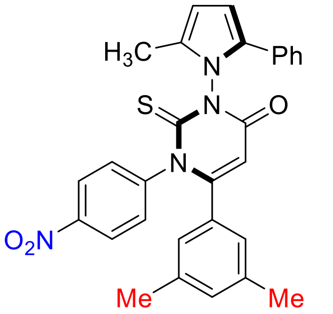

Yellow solid. **MP**: 157-159 °C. **^{1}H NMR** (500 MHz, $CDCl_3$) δ 8.09 – 8.04 (m, 2H), 7.45 – 7.34 (m, 5H), 7.23 (s, 1H), 6.86 (s, 2H), 6.63 (d, *J* = 1.5 Hz, 2H), 6.35 (d, *J* = 3.8 Hz, 1H), 6.16 (dd, *J* = 3.7, 1.1 Hz, 1H), 6.10 (s, 1H), 2.24 (d, *J* = 0.9 Hz, 3H), 2.15 (s, 6H). **^{13}C NMR** (126 MHz, $CDCl_3$) δ 178.6, 157.9, 155.5, 147.0, 146.2, 138.3, 132.0, 131.8, 131.8, 130.8, 129.1, 128.3, 128.0, 127.2, 125.9, 124.1, 107.8, 106.7, 105.8, 21.0, 11.3. **HRMS (ESI)** m/z calcd for [$C_{29}H_{24}N_4O_3S$, M+Na]$^+$: 531.1461; found: 531.1472.

Specific Rotation: $[\alpha]^{25}_{D}$ = +117.2 (*c* = 1.0, $CHCl_3$). 96% ee (HPLC condition: Chiralcel IA column, *n*-Hexane/*i*-PrOH = 70:30, flow rate = 1.0 mL/min, wavelength = 254 nm, t_R = 6.117 min for minor isomer, t_R =7.110 min for major isomer).

(*S*)-6-(3,5-dimethoxyphenyl)-3-(2-methyl-5-phenyl-1H-pyrrol-1-yl)-1-(4-nitrophenyl)-2-thioxo-2,3-dihydropyrimidin-4(1H)-one (7z)

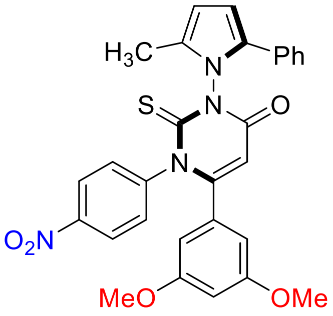

Yellow solid. **MP**: 157-159 °C. **^{1}H NMR** (500 MHz, $CDCl_3$) δ 8.08 (d, *J* = 8.7 Hz, 2H), 7.42 – 7.29 (m, 6H), 6.87 (s, 1H), 6.35 (d, *J* = 3.8 Hz, 1H), 6.29 (t, *J* = 2.3 Hz, 1H), 6.16 (dd, *J* = 3.8, 1.1 Hz, 1H), 6.15 – 6.12 (m, 3H), 3.66 (s, 6H), 2.24 (s, 3H). **^{13}C NMR** (126 MHz, $CDCl_3$) δ 178.5, 160.6, 157.9, 154.9, 147.1, 146.0, 133.6, 131.8, 131.7, 130.6, 129.2, 128.3, 128.1, 127.2, 124.3, 107.8, 106.8, 106.7, 105.8, 101.2, 55.4, 11.3. **HRMS (ESI)** m/z calcd for $[C_{29}H_{24}N_4O_5S, M+Na]^+$: 563.1359; found: 563.1368.

Specific Rotation: $[\alpha]^{25}_{D}$ = +83.7 (*c* = 1.0, $CHCl_3$). 96% ee (HPLC condition: Chiralcel AD-H column, *n*-Hexane/*i*-PrOH = 80:20, flow rate = 1.0 mL/min, wavelength = 254 nm, t_R = 13.118 min for minor isomer, t_R = 16.127 min for major isomer).

(*S*)-6-(3,4-dimethylphenyl)-3-(2-methyl-5-phenyl-1H-pyrrol-1-yl)-1-(4-nitrophenyl)-2-thioxo-2,3-dihydropyrimidin-4(1H)-one (7aa)

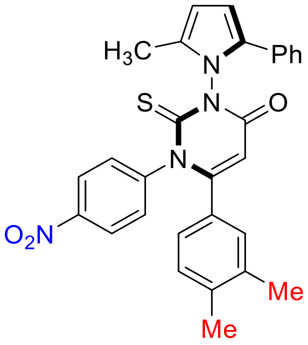

Yellow solid. **MP**: 157-159 °C. **^{1}H NMR** (500 MHz, $CDCl_3$) δ 8.08 – 8.02 (m, 2H), 7.44 – 7.34 (m, 5H), 7.22 (d, *J* = 4.4 Hz, 1H), 6.88 (d, *J* = 15.3 Hz, 2H), 6.82 (d, *J* = 1.9 Hz, 1H), 6.72 – 6.67 (m, 1H), 6.35 (d, *J* = 3.7 Hz, 1H), 6.16 (d, *J* = 3.7 Hz, 1H), 6.09 (s, 1H), 2.24 (s, 3H), 2.13 (d, *J* = 12.5 Hz, 6H). **^{13}C NMR** (126 MHz, $CDCl_3$) δ 178.6, 158.0, 155.4, 147.0, 146.2, 139.4, 137.1, 131.8, 131.7, 130.7, 129.6, 129.5, 129.1, 128.2, 128.0, 127.2, 125.6, 124.2, 107.7, 106.6, 105.8, 19.5, 19.5, 11.3. **HRMS (ESI)** m/z calcd for $[C_{29}H_{24}N_4O_3S, M+Na]^+$: 531.1461; found: 531.1469.

Specific Rotation: $[\alpha]^{25}_{D}$ = +93.1 (*c* = 1.0, $CHCl_3$). 96% ee (HPLC condition: Chiralcel AD-H column, *n*-Hexane/*i*-PrOH = 70:30, flow rate = 1.0 mL/min, wavelength = 254 nm, t_R = 8.558 min for major isomer, t_R = 10.782 min for minor isomer).

(*S*)-6-(3,4-dimethoxyphenyl)-3-(2-methyl-5-phenyl-1H-pyrrol-1-yl)-1-(4-nitrophenyl)-2-thioxo-2,3-dihydropyrimidin-4(1H)-one (7ab)

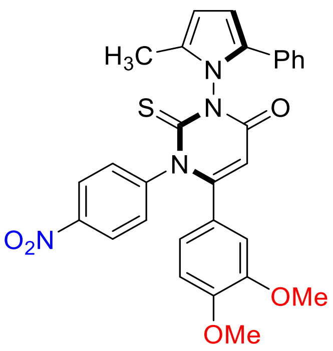

Yellow solid. **MP**: 157-159 °C. **^{1}H NMR** (500 MHz, $CDCl_3$) δ 8.11 – 8.04 (m, 2H), 7.44 – 7.28 (m, 6H), 6.92 (s, 1H), 6.68 – 6.61 (m, 2H), 6.46 (d, *J* = 2.0 Hz, 1H), 6.35 (d, *J* = 3.8 Hz, 1H), 6.16 (dd, *J* = 3.7, 1.0 Hz, 1H), 6.13 (s, 1H), 3.79 (s, 3H), 3.69 (s, 3H), 2.24 (s, 3H). **^{13}C NMR** (126 MHz, $CDCl_3$) δ 178.6, 158.0, 155.0, 150.4, 148.5, 147.0, 146.3, 131.8, 131.7, 130.7, 129.1, 128.2, 128.0, 127.1, 124.3, 124.2, 121.7, 111.2, 110.6, 107.8, 106.7, 105.9, 55.9, 55.8, 11.3. **HRMS (ESI)** m/z calcd for $[C_{29}H_{24}N_4O_5S, M+Na]^+$: 563.1359; found: 563.1362.

Specific Rotation: $[\alpha]^{25}_{D}$ = +78.5 (*c* = 1.0, $CHCl_3$). 96% ee (HPLC condition: Chiralcel AD-H column, *n*-Hexane/*i*-PrOH = 70:30, flow rate = 1.0 mL/min, wavelength = 254 nm, t_R = 13.053 min for major isomer, t_R = 18.332 min for minor isomer).

(*S*)-6-(benzo[d][1,3]dioxol-5-yl)-3-(2-methyl-5-phenyl-1H-pyrrol-1-yl)-1-(4-nitrophenyl)-2-thioxo-2,3-dihydropyrimidin-4(1H)-one (7ac)

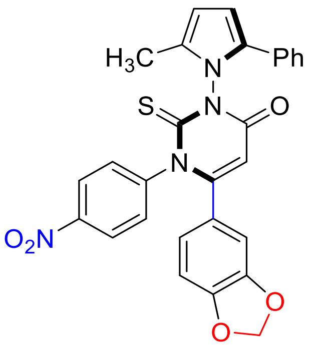

Yellow solid. **MP**: 144-146 °C. **^{1}H NMR** (500 MHz, $CDCl_3$) δ 8.09 (d, *J* = 8.6 Hz, 2H), 7.42 – 7.27 (m, 6H), 6.86 (s, 1H), 6.61 (d, *J* = 8.0 Hz, 1H), 6.53 (dd, *J* = 8.0, 1.8 Hz, 1H), 6.45 (d, *J* = 1.8 Hz, 1H), 6.34 (d, *J* = 3.8 Hz, 1H), 6.15 (d, *J* = 3.8 Hz, 1H), 6.09 (s, 1H), 5.91 (s, 2H), 2.23 (s, 3H). **^{13}C NMR** (126 MHz, $CDCl_3$) δ 178.5, 157.9, 154.7, 149.1, 147.7, 147.0, 146.1, 131.7, 131.7, 130.7, 129.1, 128.2, 128.0, 127.2, 125.5, 124.4, 122.9, 108.3, 108.3, 107.8, 106.6, 106.0, 101.8, 11.3. **HRMS (ESI)** m/z calcd for $[C_{28}H_{20}N_4O_5S, M+Na]^+$: 547.1046; found: 547.1055.

Specific Rotation: $[\alpha]^{25}_{D}$ = +65.9 (*c* = 1.0, $CHCl_3$). 98% ee (HPLC condition: Chiralcel AD-H column, *n*-Hexane/*i*-PrOH = 70:30, flow rate = 1.0 mL/min, wavelength = 254 nm, t_R = 11.840 min for major isomer, t_R = 14.610 min for minor isomer).

(*S*)-3-(2-methyl-5-phenyl-1H-pyrrol-1-yl)-1-(4-nitrophenyl)-6-(thiophen-3-yl)-2-thioxo-2,3-dihydropyrimidin-4(1H)-one (7ad)

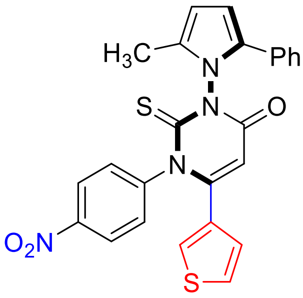

Yellow solid. **MP**: 154-156 °C. **^{1}H NMR** (500 MHz, $CDCl_3$) δ 8.25 – 8.02 (m, 2H), 7.43 – 7.25 (m, 6H), 7.19 (dd, *J* = 3.0, 1.4 Hz, 1H), 7.12 (dd, *J* = 5.1, 3.0 Hz, 1H), 6.93 (s, 1H), 6.55 (dd, *J* = 5.1, 1.4 Hz, 1H), 6.34 (d, *J* = 3.8 Hz, 1H), 6.19 (s, 1H), 6.16 (dd, *J* = 3.7, 1.1 Hz, 1H), 2.23 (s, 3H). **^{13}C NMR** (126 MHz, $CDCl_3$) δ 178.4, 157.8, 150.3, 147.3, 146.1, 132.1, 131.8, 131.7, 130.5, 129.1, 128.3, 128.1, 128.0, 127.2, 127.2, 126.7, 124.5, 107.8, 106.7, 105.7, 11.3. **HRMS (ESI)** m/z calcd for [$C_{25}H_{18}N_4O_3S_2$, M+Na]$^+$: 509.0712; found: 509.0716.

Specific Rotation: $[\alpha]^{25}_{D}$ = +59.1 (*c* = 1.0, $CHCl_3$). 94% ee (HPLC condition: Chiralcel IC column, *n*-Hexane/*i*-PrOH = 70:30, flow rate = 1.0 mL/min, wavelength = 254 nm, t_R = 26.462 min for minor isomer, t_R = 32.882 min for major isomer).

(*S*)-3-(2-methyl-1H-indol-1-yl)-1-(4-nitrophenyl)-6-phenyl-2-thioxo-2,3-dihydropyrimidin-4(1H)-one (7ae)

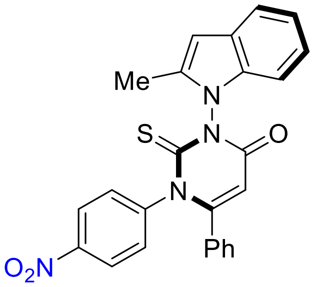

Yellow solid. **MP**: 185-187 °C. **^{1}H NMR** (500 MHz, $CDCl_3$) δ 8.11 (d, *J* = 9.3 Hz, 2H), 7.57 (d, *J* = 6.8 Hz, 1H), 7.37 (d, *J* = 7.8 Hz, 1H), 7.30 (d, *J* = 7.3 Hz, 2H), 7.26 (d, *J* = 7.6 Hz, 2H), 7.21 – 7.12 (m, 5H), 6.46 (t, *J* = 1.1 Hz, 1H), 6.24 (s, 1H), 2.36 (d, *J* = 1.1 Hz, 3H). **^{13}C NMR** (126 MHz, $CDCl_3$) δ 178.5, 157.4, 155.4, 147.2, 146.1, 135.2, 133.6, 132.3, 130.4, 128.7, 128.3, 126.8, 124.5, 122.1, 121.2, 120.7, 107.8, 106.4, 101.0, 11.6. **HRMS (ESI)** m/z calcd for [$C_{25}H_{18}N_4O_3S$, M+H]$^+$: 455.1173; found: 455.1196.

Specific Rotation: $[\alpha]^{25}_{D}$ = +71.1 (*c* = 1.0, $CHCl_3$). 80% ee (HPLC condition: Chiralcel OD-H column, *n*-Hexane/*i*-PrOH = 50:50, flow rate = 1.0 mL/min, wavelength = 254 nm, t_R = 11.920 min for minor isomer, t_R = 26.487 min for major isomer).

(*S*)-1-(4-nitrophenyl)-6-phenyl-3-(2-phenyl-1H-indol-1-yl)-2-thioxo-2,3-dihydropyrimidin-4(1H)-one (7af)

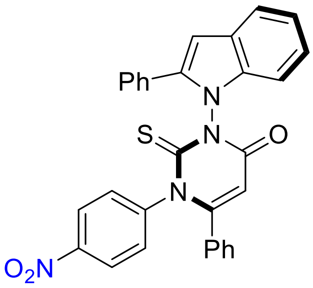

Yellow solid. **MP**: 178-179 °C. **^{1}H NMR** (500 MHz, $CDCl_3$) δ 8.03 (dt, *J* = 8.7, 1.2 Hz, 2H), 7.68 (dt, *J* = 7.7, 1.1 Hz, 1H), 7.62 – 7.58 (m, 2H), 7.48 – 7.41 (m, 3H), 7.29 – 7.24 (m, 5H), 7.19 (dd, *J* = 8.4, 6.8 Hz, 2H), 7.05 – 6.98 (m, 2H), 6.88 (s, 1H), 6.79 (s, 1H), 6.12 (s, 1H). **^{13}C NMR** (126 MHz, $CDCl_3$) δ 178.2, 157.9, 155.2, 147.1, 146.1, 140.1, 135.4, 132.2, 130.9, 130.3, 128.6, 128.5, 128.5, 128.2, 127.1, 124.4, 123.4, 121.9, 121.5, 109.1, 106.2, 103.3. **HRMS (ESI)** m/z calcd for $[C_{30}H_{20}N_4O_3S, M+H]^+$: 517.1329; found: 517.1285.

Specific Rotation: $[\alpha]^{25}_{D}$ = +126.3 (*c* = 1.0, $CHCl_3$). 92% ee (HPLC condition: Chiralcel IA column, *n*-Hexane/*i*-PrOH = 80:20, flow rate = 1.0 mL/min, wavelength = 254 nm, t_R = 26.340 min for minor isomer, t_R = 39.972 min for major isomer).

(*S*)-Methyl 2-(diphenylphosphoryl)-1-(3-(4-nitrophenyl)-6-oxo-4-phenyl-2-thioxo-3,6-dihydropyrimidin-1(2H)-yl)-1H-indole-3-carboxylate (7ag)

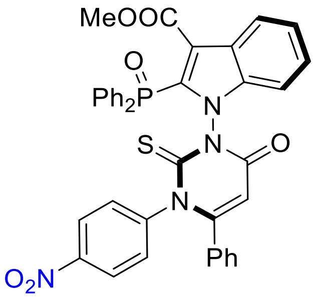

Syrup. **^{1}H NMR** (500 MHz, $CDCl_3$) δ 8.21 (d, *J* = 8.1 Hz, 1H), 8.03 (t, *J* = 7.9 Hz, 2H), 7.96 – 7.90 (m, 2H), 7.89 – 7.82 (m, 2H), 7.51 (dd, *J* = 7.3, 1.7 Hz, 2H), 7.47 – 7.38 (m, 8H), 7.33 – 7.29 (m, 1H), 7.25 – 7.16 (m, 5H), 6.22 (s, 1H), 3.45 (s, 3H). **^{13}C NMR** (126 MHz, $CDCl_3$) δ 179.7, 163.3, 158.2, 155.4, 147.2, 146.8, 137.5, 132.8, 132.7, 132.5, 132.4, 132.1, 132.0, 131.5, 131.0, 130.1, 128.6, 128.5, 128.2, 128.1, 128.0, 127.9, 126.5, 124.7, 123.9, 123.1, 109.2, 106.9, 51.2. **^{31}P NMR** (202 MHz, $CDCl_3$) δ 27.64. **HRMS (ESI)** m/z calcd for $[C_{38}H_{27}N_4O_6PS, M+Na]^+$: 721.1281; found: 721.1319.

Specific Rotation: $[\alpha]^{25}_{D}$ = +55.7 (*c* = 1.0, $CHCl_3$). 86% ee (HPLC condition: Chiralcel OD-H column, *n*-Hexane/*i*-PrOH = 70:30, flow rate = 1.0 mL/min, wavelength = 254 nm, t_R = 8.462 min for minor isomer, t_R = 13.068 min for major isomer).

2-((2-methyl-5-phenyl-1H-pyrrol-1-yl)imino)-6-phenyl-3-(2-(trifluoromethyl)phenyl)-2,3-dihydro-4H-1,3-thiazin-4-one (9a)

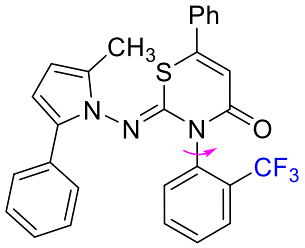

Yellow solid. **MP**: 180-182 °C. **^{1}H NMR** (500 MHz, $CDCl_3$) δ 7.88 (d, *J* = 7.9 Hz, 1H), 7.80 (s, 1H), 7.66 (s, 1H), 7.50 – 7.40 (m, 5H), 7.33 (dt, *J* = 23.8, 7.9 Hz, 5H), 7.24 (d, *J* = 13.9 Hz, 1H), 6.61 (s, 1H), 6.25 (d, *J* = 3.9 Hz, 1H), 5.98 (d, *J* = 3.8 Hz, 1H), 2.11 (s, 3H). **^{13}C NMR** (126 MHz, $CDCl_3$) δ 163.5, 161.8, 149.1, 134.7, 133.9, 133.2, 132.4, 131.8, 130.5, 129.8, 129.3, 128.1 (d, *J* = 10.7 Hz), 128.0, 127.9, 127.9, 127.2, 126.5, 126.4, 126.1, 113.8, 106.3, 105.8, 11.4. **^{19}F NMR** (471 MHz, $CDCl_3$) δ -61.07. **HRMS (ESI)** m/z calcd for $[C_{28}H_{20}F_3N_3OS, M+Na]^+$: 526.1171; found: 526.1183.

Specific Rotation: $[\alpha]^{25}_{D}$ = +48.6 (*c* = 1.0, $CHCl_3$). 77% ee (HPLC condition: Chiralcel IA column, *n*-Hexane/*i*-PrOH = 80:20, flow rate = 1.0 mL/min, wavelength = 254 nm, t_R = 6.645 min for minor isomer, t_R = 10.763 min for major isomer).

2-((2-(4-bromophenyl)-5-methyl-1H-pyrrol-1-yl)imino)-6-phenyl-3-(2-(trifluoromethyl)phenyl)-2,3-dihydro-4H-1,3-thiazin-4-one (9b)

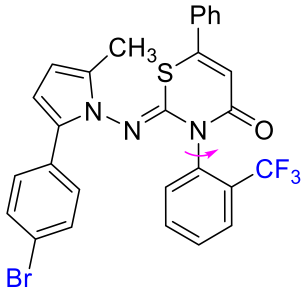

Yellow solid. **MP**: 189-190 °C. **^{1}H NMR** (500 MHz, $CDCl_3$) δ 7.89 (d, *J* = 7.9 Hz, 1H), 7.82 (t, *J* = 7.7 Hz, 1H), 7.66 (t, *J* = 7.7 Hz, 1H), 7.49 – 7.46 (m, 1H), 7.45 – 7.40 (m, 5H), 7.29 (d, *J* = 8.0 Hz, 1H), 7.23 (d, *J* = 2.6 Hz, 2H), 7.22 (s, 1H), 6.63 (s, 1H), 6.25 (d, *J* = 3.8 Hz, 1H), 5.98 (d, *J* = 3.9 Hz, 1H), 2.10 (s, 3H). **^{13}C NMR** (126 MHz, $CDCl_3$) δ 163.8, 161.6, 148.9, 134.6, 133.8, 133.2, 131.8, 131.2 (d, *J* = 7.7 Hz), 130.3, 129.9, 129.3, 128.5, 128.2 – 128.0 (m), 127.0, 126.8, 126.4, 123.13 (d, J = 273.9 Hz), 119.8, 113.9, 106.6, 106.0, 11.4. **^{19}F NMR** (471 MHz, $CDCl_3$) δ -60.99. **HRMS (ESI)** m/z calcd for $[C_{28}H_{19}BrF_3N_3OS, M+Na]^+$: 582.0457; found: 582.0490.

Specific Rotation: $[\alpha]^{25}_{D}$ = +37.0 (*c* = 1.0, $CHCl_3$). 54% ee (HPLC condition: Chiralcel IA column, *n*-Hexane/*i*-PrOH = 70:30, flow rate = 1.0 mL/min, wavelength = 254 nm, t_R = 6.068 min for minor isomer, t_R = 6.723 min for major isomer).

2-((2,5-dimethyl-1H-pyrrol-1-yl)imino)-6-phenyl-3-(2-(trifluoromethyl)phenyl)-2,3-dihydro-4H-1,3-thiazin-4-one (9c)

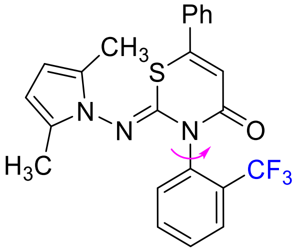

Yellow solid. **MP**: 190-192 °C. **^{1}H NMR** (500 MHz, $CDCl_3$) δ 7.85 (dd, *J* = 8.0, 1.4 Hz, 1H), 7.74 (td, *J* = 7.7, 1.4 Hz, 1H), 7.60 (t, *J* = 7.7 Hz, 1H), 7.56 – 7.53 (m, 2H), 7.51 – 7.49 (m, 1H), 7.47 – 7.43 (m, 2H), 7.40 (d, *J* = 7.9 Hz, 1H), 6.74 (s, 1H), 5.79 (s, 2H), 2.02 (s, 6H). **^{13}C NMR** (126 MHz, $CDCl_3$) δ 163.8, 161.9, 149.2, 134.9, 134.0, 133.5, 131.8, 130.5, 129.6, 129.3, 128.0 (d, *J* = 4.4 Hz), 126.5, 124.2, 124.0 (d, *J* = 116.1 Hz), 122.0, 113.9, 104.2, 11.3. **^{19}F NMR** (471 MHz, $CDCl_3$) δ -61.20. **HRMS (ESI)** m/z calcd for [$C_{23}H_{18}F_3N_3OS$, M+H]$^+$: 442.1196; found: 442.1203.

Specific Rotation: $[\alpha]^{25}_{D}$ = +34.7 (*c* = 1.0, $CHCl_3$). 60% ee (HPLC condition: Chiralcel IA column, *n*-Hexane/*i*-PrOH = =90:10, flow rate = 1.0 mL/min, wavelength = 254 nm, t_R = 8.982 min for major isomer, t_R = 10.717 min for minor isomer).

(*R*)-1-(3-iodo-2-methyl-1H-indol-1-yl)-2-(methylthio)-6-phenylpyrimidin-4(1H)-one (4)

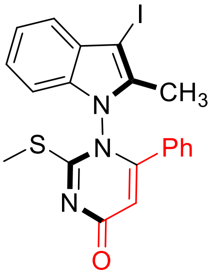

White solid. **MP**: 183-185 °C. **^{1}H NMR** (500 MHz, $CDCl_3$) δ 7.25 – 7.17 (m, 4H), 7.08 (d, *J* = 8.1 Hz, 1H), 7.02 (d, *J* = 7.8 Hz, 2H), 6.95 (dd, *J* = 8.2, 1.4 Hz, 2H), 6.12 (s, 1H), 2.39 (s, 3H), 2.15 (s, 3H). **^{13}C NMR** (126 MHz, $CDCl_3$) δ 167.7, 166.6, 155.1, 136.3, 135.8, 130.7, 129.1, 128.3, 128.2, 127.4, 124.5, 122.7, 121.5, 110.3, 108.9, 61.7, 14.5, 12.4. **HRMS (ESI)** m/z calcd for [$C_{20}H_{16}IN_3OS$, M+Na]$^+$: 495.9951; found: 495.9963.

Specific Rotation: $[\alpha]^{25}_{D}$ = +174.5 (*c* = 1.0, $CHCl_3$). 90% ee (HPLC condition: Chiralcel OD-H column, *n*-Hexane/*i*-PrOH = 80:20, flow rate = 1.0 mL/min, wavelength = 254 nm, t_R = 16.310 min for minor isomer, t_R = 28.493 min for major isomer).

(*S*)-1-(3-iodo-2-methyl-1H-indol-1-yl)-2-(methylthio)-6-phenylpyrimidin-4(1H)-one (*ent*-4)

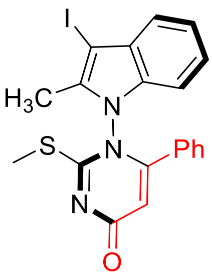

Specific Rotation: $[\alpha]^{25}_{D}$ = -172.8 (*c* = 1.0, $CHCl_3$). 90% ee (HPLC condition: Chiralcel OD-H column, *n*-Hexane/*i*-PrOH = 80:20, flow rate = 1.0 mL/min, wavelength = 254 nm, t_R = 16.363 min for minor isomer, t_R = 29.265 min for major isomer).

(*R*a,*R*)-2-methyl-1-(2-(methylthio)-4,6-diphenylpyrimidin-1(6H)-yl)-1H-indole (10)

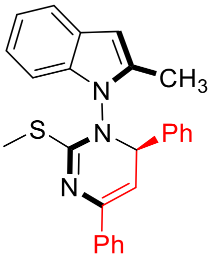

Yellow solid. **MP**: 183-185 °C. **^{1}H NMR** (500 MHz, $CDCl_3$) δ 7.95 (d, *J* = 7.6 Hz, 2H), 7.54 – 7.47 (m, 2H), 7.41 (t, *J* = 7.6 Hz, 2H), 7.36 (d, *J* = 7.0 Hz, 1H), 7.30 (d, *J* = 7.2 Hz, 1H), 7.25 (t, *J* = 7.2 Hz, 3H), 7.18 (d, *J* = 7.5 Hz, 1H), 7.08 (d, *J* = 7.4 Hz, 2H), 6.03 (s, 1H), 5.76 (d, *J* = 3.6 Hz, 1H), 5.68 (d, *J* = 3.6 Hz, 1H), 2.40 (s, 3H), 1.48 (s, 3H). **^{13}C NMR** (126 MHz, $CDCl_3$) δ 160.7, 140.9, 140.4, 138.3, 137.7, 132.9, 129.1, 128.9, 128.5, 128.2, 128.1, 127.1, 125.5, 121.8, 121.2, 120.4, 108.9, 103.0, 99.4, 63.8, 13.7, 10.9. **HRMS (ESI)** m/z calcd for $[C_{26}H_{23}N_3S, M+H]^+$: 410.1; found: 410.1.

Specific Rotation: $[\alpha]^{25}_{D}$ = +214.1 (*c* = 1.0, $CHCl_3$). 93% ee (HPLC condition: Chiralcel AD-H column, *n*-Hexane/*i*-PrOH = 97:3, flow rate = 0.3 mL/min, wavelength = 254 nm, t_R = 14.982 min for minor isomer, t_R = 18.963 min for major isomer).

(*R*)-1-(3-ethynyl-2-methyl-1H-indol-1-yl)-2-(methylthio)-6-phenylpyrimidin-4(1H)-one (11)

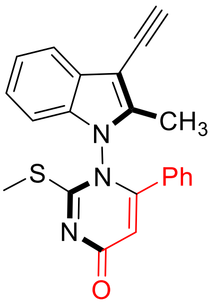

White solid. **MP**: 178-180 °C. **^{1}H NMR** (500 MHz, $CDCl_3$) δ 7.59 (d, *J* = 7.8 Hz, 1H), 7.33 (d, *J* = 7.8 Hz, 1H), 7.27 (d, *J* = 3.5 Hz, 2H), 7.20 (d, *J* = 8.1 Hz, 1H), 7.13 (t, *J* = 7.8 Hz, 2H), 7.05 (d, *J* = 7.8 Hz, 2H), 6.20 (s, 1H), 3.27 (s, 1H), 2.47 (s, 3H), 2.25 (s, 3H). **^{13}C NMR** (126 MHz, $CDCl_3$) δ 167.7, 166.5, 155.1,

140.0, 134.6, 130.8, 129.2, 128.5, 127.5, 126.2, 124.3, 122.8, 120.0, 110.3, 108.8, 97.2, 81.9, 75.4, 14.5, 10.6. **HRMS (ESI)** m/z calcd for $[C_{22}H_{17}N_3OS, M+Na]^+$: 394.0984; found: 394.0990.

Specific Rotation: $[\alpha]^{25}_D$ = +244.2 (*c* = 1.0, $CHCl_3$). 88% ee (HPLC condition: Chiralcel IC column, *n*-Hexane/*i*-PrOH = 70:30, flow rate = 1.0 mL/min, wavelength = 254 nm, t_R = 38.285 min for minor isomer, t_R = 46.190 min for major isomer).

(*S*)-3-(3,4-dibromo-2-methyl-5-phenyl-1H-pyrrol-1-yl)-1-(4-nitrophenyl)-6-phenyl-2-thioxo-2,3-dihydropyrimidin-4(1H)-one (12)

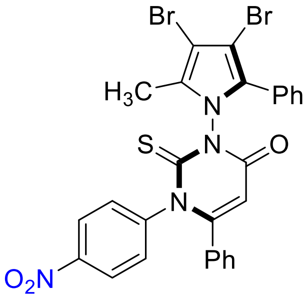

White solid. **MP**: 173-175 °C. **^{1}H NMR** (500 MHz, $CDCl_3$) δ 8.18 – 7.91 (m, 2H), 7.44 (s, 5H), 7.28 – 7.23 (m, 1H), 7.19 (t, *J* = 7.6 Hz, 3H), 7.00 – 6.91 (m, 2H), 6.70 (s, 1H), 6.07 (s, 1H), 2.26 (s, 3H). **^{13}C NMR** (126 MHz, $CDCl_3$) δ 177.7, 157.5, 155.3, 147.1, 145.8, 131.9, 130.6, 130.4, 130.1, 129.7, 129.0, 128.7, 128.6, 128.2, 128.0, 127.6, 124.4, 105.7, 99.3, 99.1, 10.9. **HRMS (ESI)** m/z calcd for $[C_{27}H_{18}Br_2N_4O_3S, M+Na]^+$: 658.9358; found: 658.9359.

Specific Rotation: $[\alpha]^{25}_D$ = +105.6 (*c* = 1.0, $CHCl_3$). 94% ee (HPLC condition: Chiralcel IA column, *n*-Hexane/*i*-PrOH = 70:30, flow rate = 1.0 mL/min, wavelength = 254 nm, t_R = 7.507 min for minor isomer, t_R = 8.935 min for major isomer).

(*S*)-methyl 2-(diphenylphosphaneyl)-1-(3-(4-nitrophenyl)-6-oxo-4-phenyl-2-thioxo-3,6-dihydropyrimidin-1(2H)-yl)-1H-indole-3-carboxylate (13)

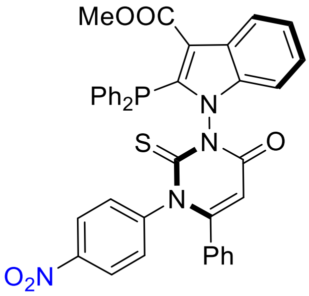

Yellow solid. **MP**: 193-195 °C. **^{1}H NMR** (500 MHz, $CDCl_3$) δ 8.22 (dd, *J* = 6.7, 2.6 Hz, 1H), 8.04 (d, *J* = 7.9 Hz, 2H), 7.66 – 7.57 (m, 4H), 7.32 (tt, *J* = 8.6, 3.4 Hz, 8H), 7.24 – 7.16 (m, 4H), 7.12 (s, 2H), 7.04 (d, *J* = 7.4 Hz, 2H), 5.89 (s, 1H), 3.53 (d, *J* = 1.7 Hz, 3H). **^{13}C NMR** (126 MHz, $CDCl_3$) δ 178.0, 164.2, 157.1, 154.5, 147.3, 146.0, 141.4, 136.1, 134.3, 134.2, 133.8, 133.6, 132.4, 130.3, 128.9, 128.8, 128.6, 128.3, 128.2, 128.1, 128.1, 124.6, 124.3, 123.2, 122.3, 114.8, 108.6, 106.2, 50.8. **^{31}P NMR** (202 MHz, $CDCl_3$) δ -16.33. **HRMS (ESI)** m/z calcd for $[C_{38}H_{27}N_4O_5PS, M+H]^+$: 683.1513; found: 683.1482.

Specific Rotation: $[\alpha]^{25}_{D}$ = +49.1 (c = 1.0, $CHCl_3$). 85% ee (HPLC condition: Chiralcel IA column, *n*-Hexane/*i*-PrOH = 70:30, flow rate = 1.0 mL/min, wavelength = 254 nm, t_R = 11.027 min for minor isomer, t_R = 19.310 min for major isomer).

(*R*,*E*)-Dimethyl -2-(1,3-diphenylallyl)malonate (16)

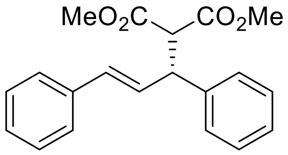

Yellow oil. **^{1}H NMR** (500 MHz, $CDCl_3$) δ 7.36 – 7.16 (m, 10H), 6.48 (d, J = 15.8 Hz, 1H), 6.33 (dd, J = 15.7, 8.6 Hz, 1H), 4.27 (dd, J = 10.9, 8.6 Hz, 1H), 3.96 (d, J = 10.8 Hz, 1H), 3.69 (s, 3H), 3.51 (s, 3H). **^{13}C NMR** (126 MHz, $CDCl_3$) δ 168.0, 167.6, 140.0, 136.6, 131.6, 128.9, 128.6, 128.3, 127.7, 127.4, 127.0, 126.2, 57.4, 52.4, 52.3, 49.0.

40% ee (HPLC condition: Chiralcel IA column, *n*-Hexane/*i*-PrOH = 80:20, flow rate = 1.0 mL/min, wavelength = 254 nm, t_R = 6.167 min for major isomer, t_R = 7.267 min for minor isomer).

10. X-ray Crystallographic Data

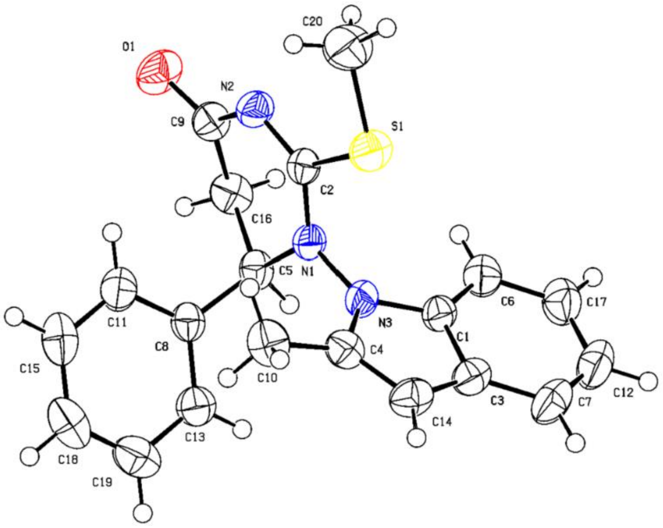

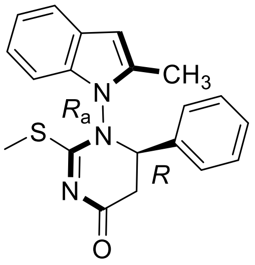

CCDC: 2206276

Supplementary Table 3. X-ray Crystallographic Data of Compound **3a**

Bond precision:	C-C = 0.0048 Å		Wavelength=1.54184
Cell:	a=7.8516(1)	b=12.3466(2)	c=18.4272(2)
	alpha=90	beta=90	gamma=90
Temperature:	278 K		
	Calculated		Reported
Volume	1786.34(4)		1786.34(4)
Space group	P 21 21 21		P 21 21 21
Hall group	P 2ac 2ab		P 2ac 2ab
Moiety formula	$C_{20} H_{19} N_3OS$		$C_{20} H_{19} N_3OS$
Sum formula	$C_{20} H_{19} N_3OS$		$C_{20} H_{19} N_3OS$
Mr	349.44		349.44
Dx,g cm^{-3}	1.299		1.299
Z	4		4
Mu (mm^{-1})	1.703		1.703
F000	736.0		736.0
F000’	739.22		
h,k,l_{max}	9,15,23		9,15,23
Nref	3796[2179]		3593
T_{min}, T_{max}	0.799,0.858		0.445,1.000
$T_{min'}$	0.775		

Correction method= # Reported T Limits: T_{min}=0.445 T_{max}=1.000
AbsCorr = MULTI-SCAN
Data completeness= 1.65/0.95 Theta(max)= 77.387
R(reflections)= 0.0541(3413) wR2(reflections)= 0.1493(3593)
S = 1.057 Npar= 228

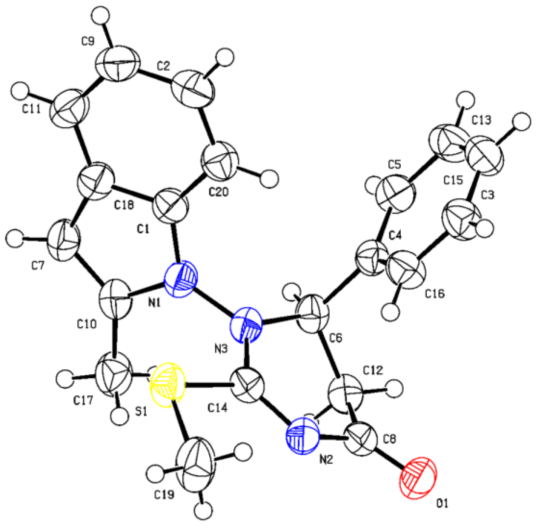

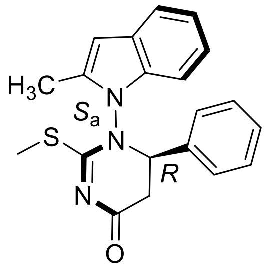

CCDC: 2218428

Supplementary Table 4. X-ray Crystallographic Data of Compound **3a’**

Bond precision:	C-C = 0.0039 Å	Wavelength=1.54178	
Cell:	a=28.2700(16)<br>alpha=90	b=7.7477(5)<br>beta=90	c=8.0164(5)<br>gamma=90
Temperature:	254 K		
	Calculated		Reported
Volume	1755.81(19)		1755.81(19)
Space group	P 21 21 21		P 21 21 21
Hall group	P 2ac 2ab		P 2ac 2ab
Moiety formula	$C_{20}\,H_{19}\,N_3OS$		$C_{20}\,H_{19}\,N_3OS$
Sum formula	$C_{20}\,H_{19}\,N_3OS$		$C_{20}\,H_{19}\,N_3OS$
Mr	349.44		349.44
Dx,g cm^{-3}	1.322		1.322
Z	4		4
Mu (mm^{-1})	1.732		1.732
F000	736.0		736.0
F000’	739.22		
h,k,l$_{max}$	34,9,9		34,9,9
Nref	3238[1897]		3230
T_{min},T_{max}	0.457,0.595		0.471,0.753
$T_{min'}$	0.401		

Correction method= # Reported T Limits: T_{min}=0.471 T_{max}=0.753
AbsCorr = MULTI-SCAN
Data completeness= 1.70/1.00 Theta(max)= 68.437
R(reflections)= 0.0371(3153) wR2(reflections)= 0.1010(3230)
S = 1.041 Npar= 228

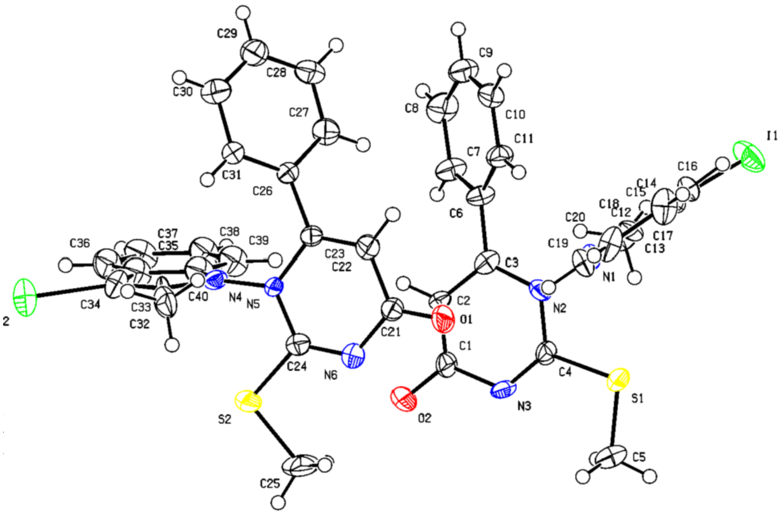

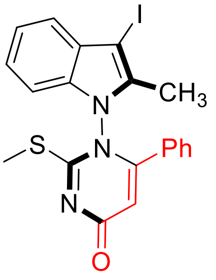

CCDC: 2221389

Supplementary Table 5. X-ray Crystallographic Data of Compound **4**

Bond precision:	C-C = 0.0167 Å	Wavelength=0.71000	
Cell:	a=7.3803(2)	b=9.3194(2)	c=55.0127(11)
	alpha=90	beta=90	gamma=90
Temperature:	293 K		
	Calculated		Reported
Volume	3783.77(15)		3783.77(15)
Space group	P 21 21 21		P 21 21 21
Hall group	P 2ac 2ab		P 2ac 2ab
Moiety formula	$C_{20} H_{16} I N_3OS$		$2(C_{20} H_{16} I N_3OS)$
Sum formula	$C_{20} H_{16} I N_3OS$		$C_{40} H_{32} I_2 N_6 O_2S_2$
Mr	473.32		946.63
Dx,g cm^{-3}	1.662		1.662
Z	8		4
Mu (mm^{-1})	1.817		1.817
F000	1872.0		1872.0
F000’	1869.76		
h,k,l_{max}	8,11,66		8,11,66
Nref	6911[3995]		6857
T_{min},T_{max}	0.790,0.834		0.374,0.753
$T_{min'}$	0.790		

Correction method= # Reported T Limits: T_{min}=0.374 T_{max}=0.753 AbsCorr = NONE
Data completeness= 1.72/0.99 Theta(max)= 25.321
R(reflections)= 0.0687(6324) wR2(reflections)= 0.1869(6857)
S = 1.049 Npar= 461

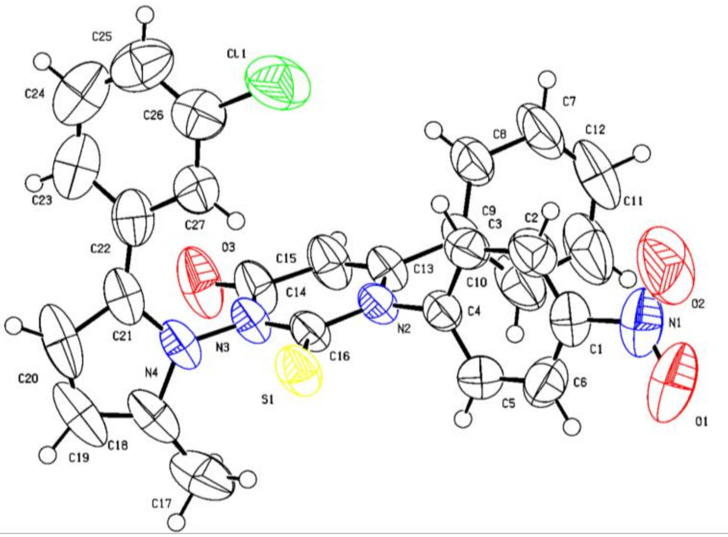

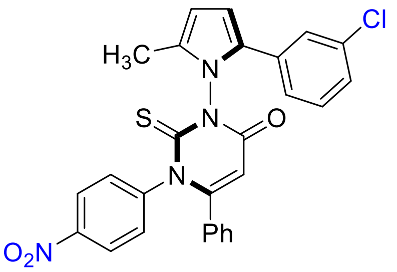

CCDC: 2202253

Supplementary Table 6. X-ray Crystallographic Data of Compound **7l**

Bond precision:	C-C = 0.0056 Å	Wavelength=1.34139	
Cell:	a=9.5184(11)	b=15.0735(17	c=17.613(2)
	alpha=90	beta=90	gamma=90
Temperature:	298 K		
	Calculated		Reported
Volume	2527.0(5)		2527.0(5)
Space group	P 21 21 21		P 21 21 21
Hall group	P 2ac 2ab		P 2ac 2ab
Moiety formula	$C_{27} H_{19} ClN_4O_3S$		$0.167(C_{27} H_{19} ClN_4O_3S)$
Sum formula	$C_{27} H_{19} ClN_4O_3S$		C4.50 H3.17 Cl0.17 N0.67 O0.50 S0.17
Mr	514.97		85.83
Dx,g cm^{-3}	1.354		1.354
Z	4		24
Mu (mm^{-1})	1.588		1.588
F000	1064.0		1064.0
F000’	1068.64		
h,k,l_{max}	12,20,23		12,18,23
Nref	6549[3674]		5988
T_{min},T_{max}			0.598,0.753
$T_{min'}$			

Correction method= # Reported T Limits: T_{min}=0.598 T_{max}=0.753

AbsCorr = NONE

Data completeness= 1.63/0.91 Theta(max)= 65.143

R(reflections)= 0.0447(4803) wR2(reflections)=0.1415(5988)

S = 1.095 Npar= 327

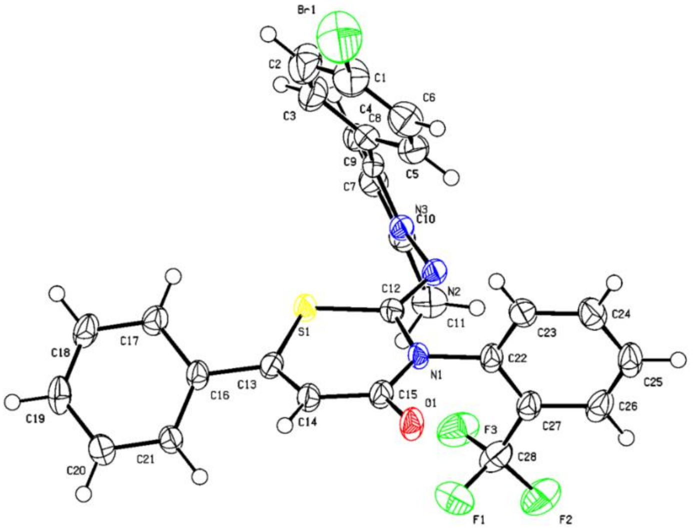

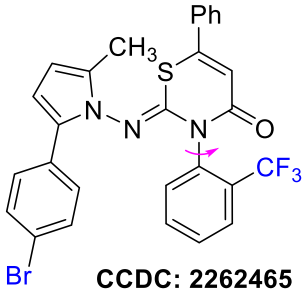

CCDC: 2262465

Supplementary Table 7. X-ray Crystallographic Data of Compound **9b**

Bond precision:	C-C = 0.0053 Å	Wavelength=0.71073	
Cell:	a=17 . 9734(8)	b=10 .2688(4)	c=17.8687(7)
	alpha=90	beta=115 .322(1)	gamma=90
Temperature:	100 K		
	Calculated		Reported
Volume	2981 .1(2)		2981 .1(2)
Space group	P 21/c		P 1 21/c 1
Hall group	-P 2ybc		-P 2ybc
Moiety formula	$C_{28}H_{19}BrF_3N_3OS$		$C_{28}H_{19}BrF_3N_3OS$
Sum formula	$C_{28}H_{19}BrF_3N_3OS$		$C_{28}H_{19}BrF_3N_3OS$
Mr	582.42		582.42
Dx,g cm^{-3}	1.298		1.298
Z	4		4
Mu (mm^{-1})	1.491		1.491
F000	1176.0		1176.0
F000’	1175.88		
h,k,l_{max}	22,12,22		22,12,22
Nref	6141		6120
T_{min},T_{max}	0.867,0.928		0.545,0.745
$T_{min'}$	0.800		

Correction method= # Reported T Limits: T_{min}=0.545 T_{max}=0.745

AbsCorr = NONE

Data completeness= 0.997 Theta(max)= 26.451

R(reflections)= 0.0554(3682) wR2(reflections)= 0.1569(6120)

S = 1.025 Npar= 335

11. NMR spectra and Chiral HPLC chromatograms

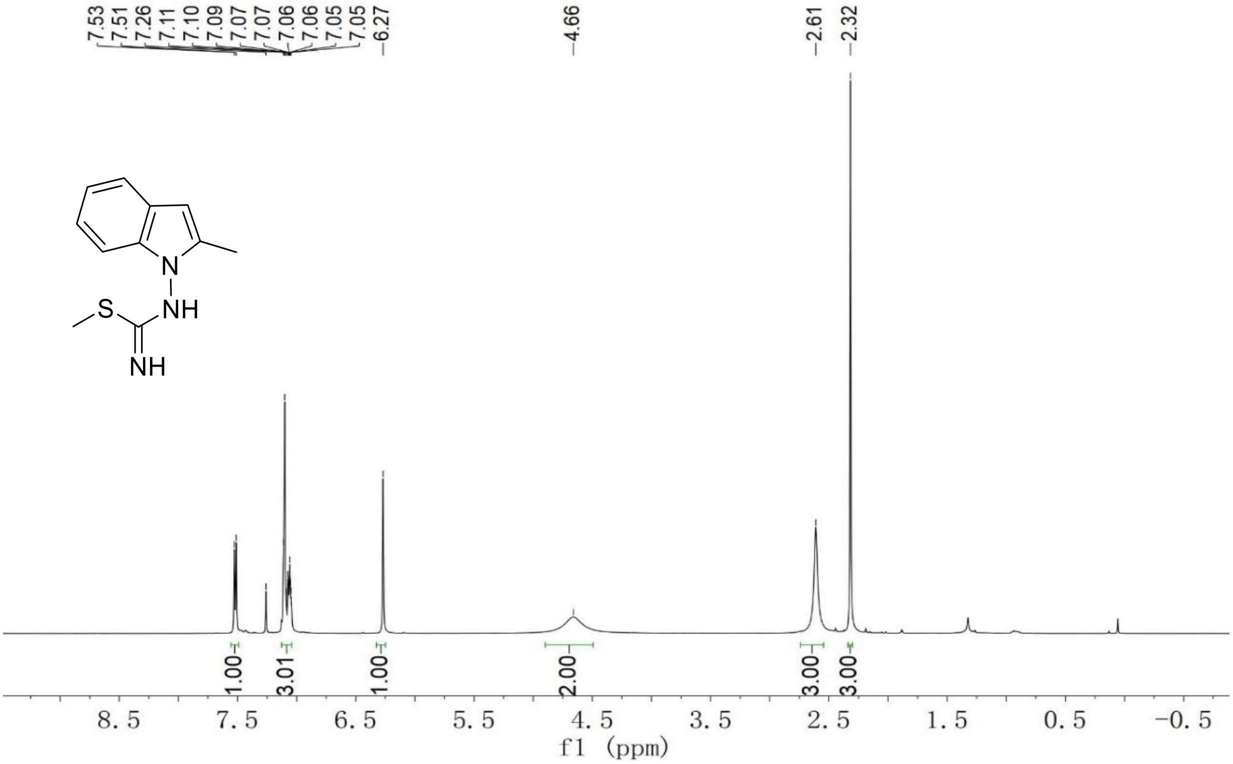

Supplementary Figure 7. ^{1}**H NMR spectrum of compound 1a (**$CDCl_3$**, 500 MHz, 298 K)**

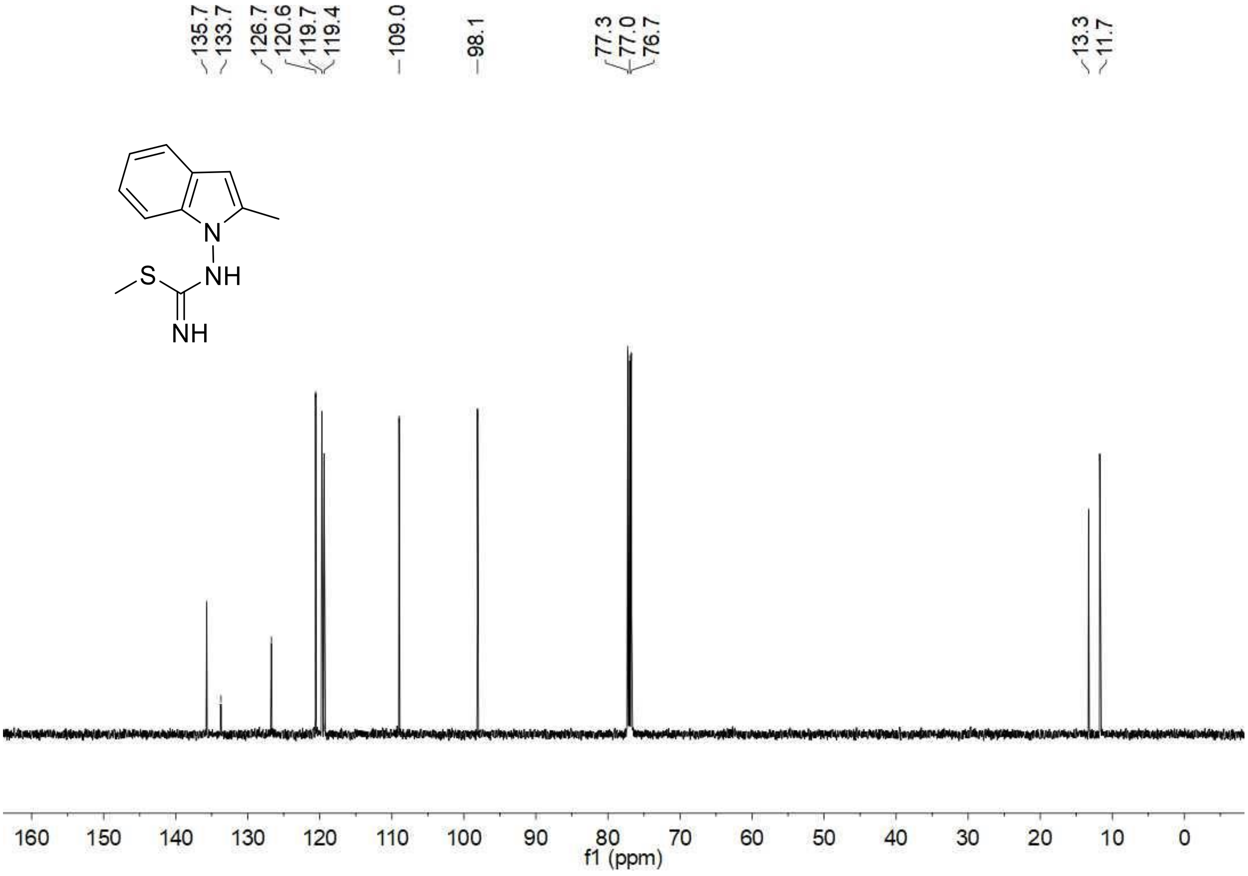

Supplementary Figure 8. ^{13}**C NMR spectrum of compound 1a (**$CDCl_3$**, 126 MHz, 298 K)**

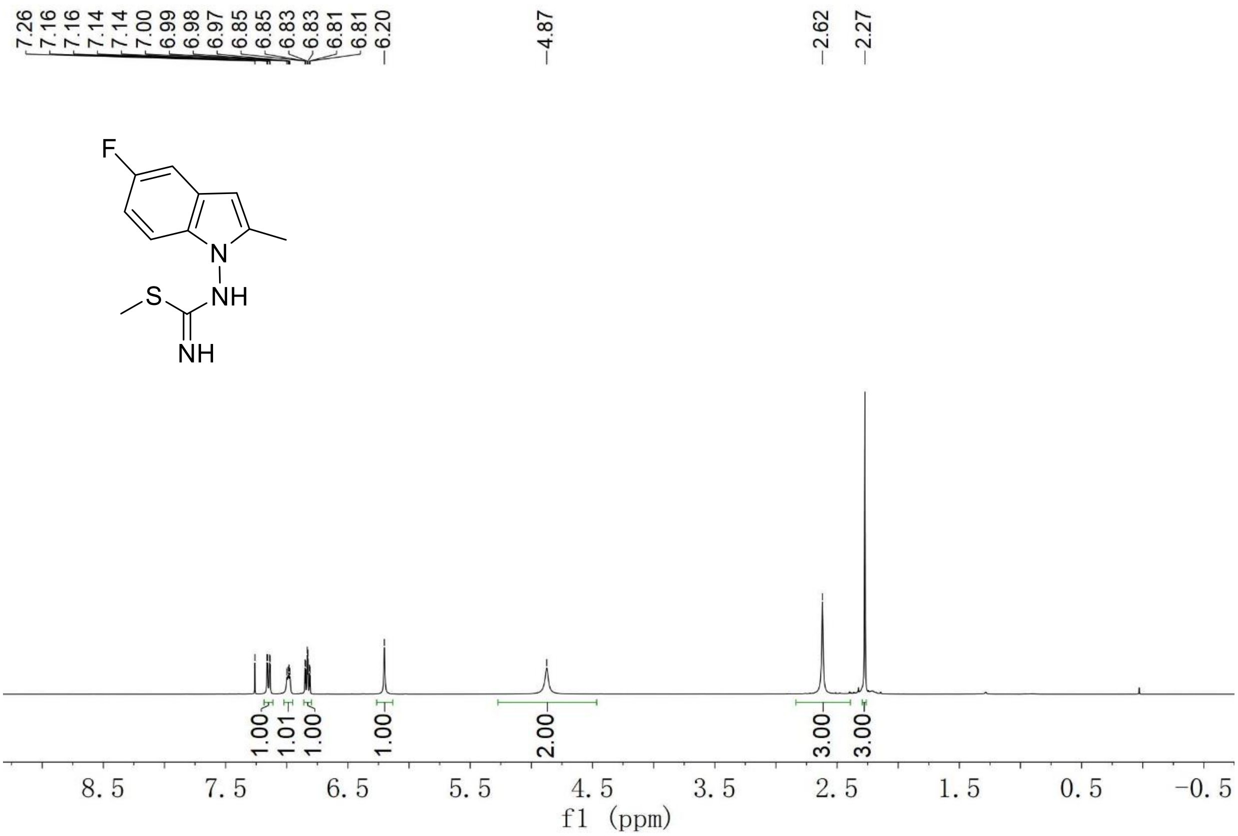

Supplementary Figure 9. ^{1}H NMR spectrum of compound 1n ($CDCl_3$, 500 MHz, 298 K)

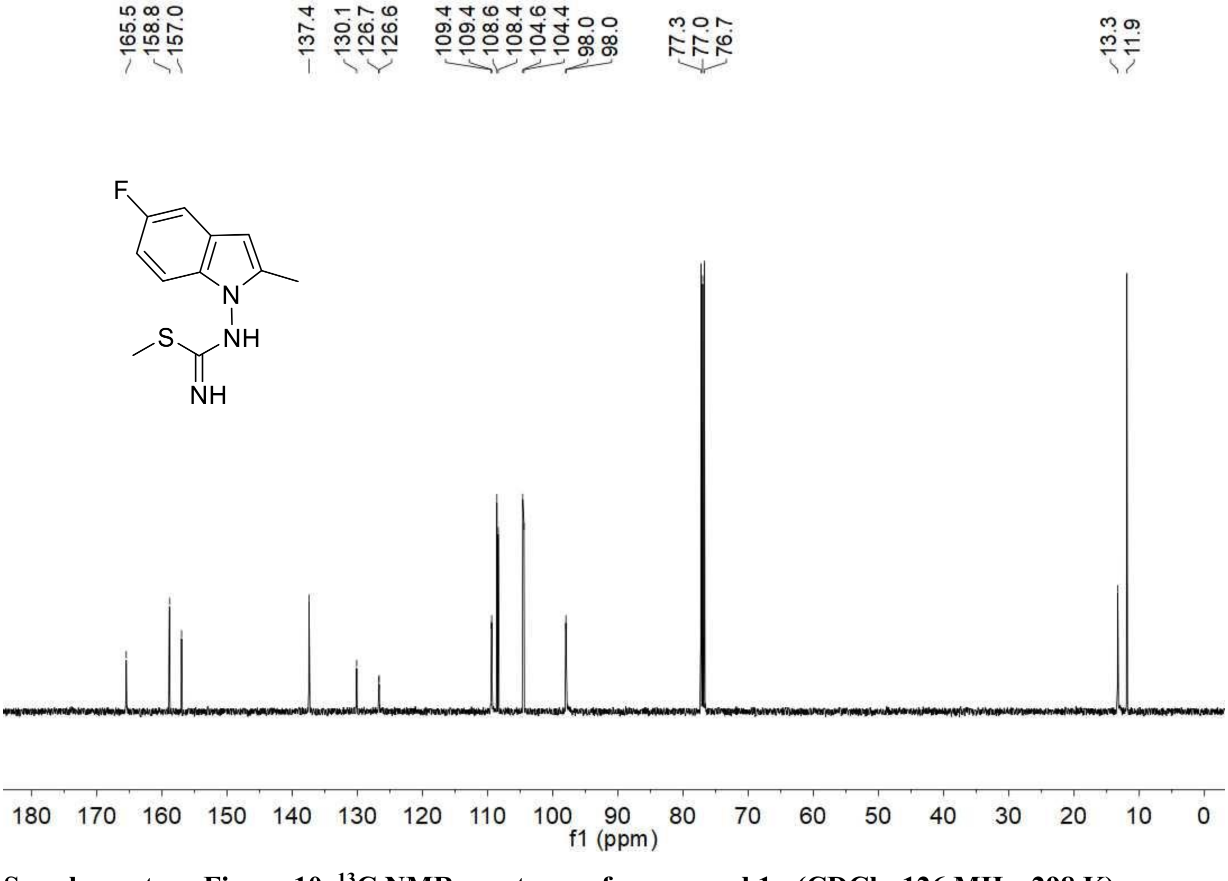

Supplementary Figure 10. ^{13}C NMR spectrum of compound 1n ($CDCl_3$, 126 MHz, 298 K)

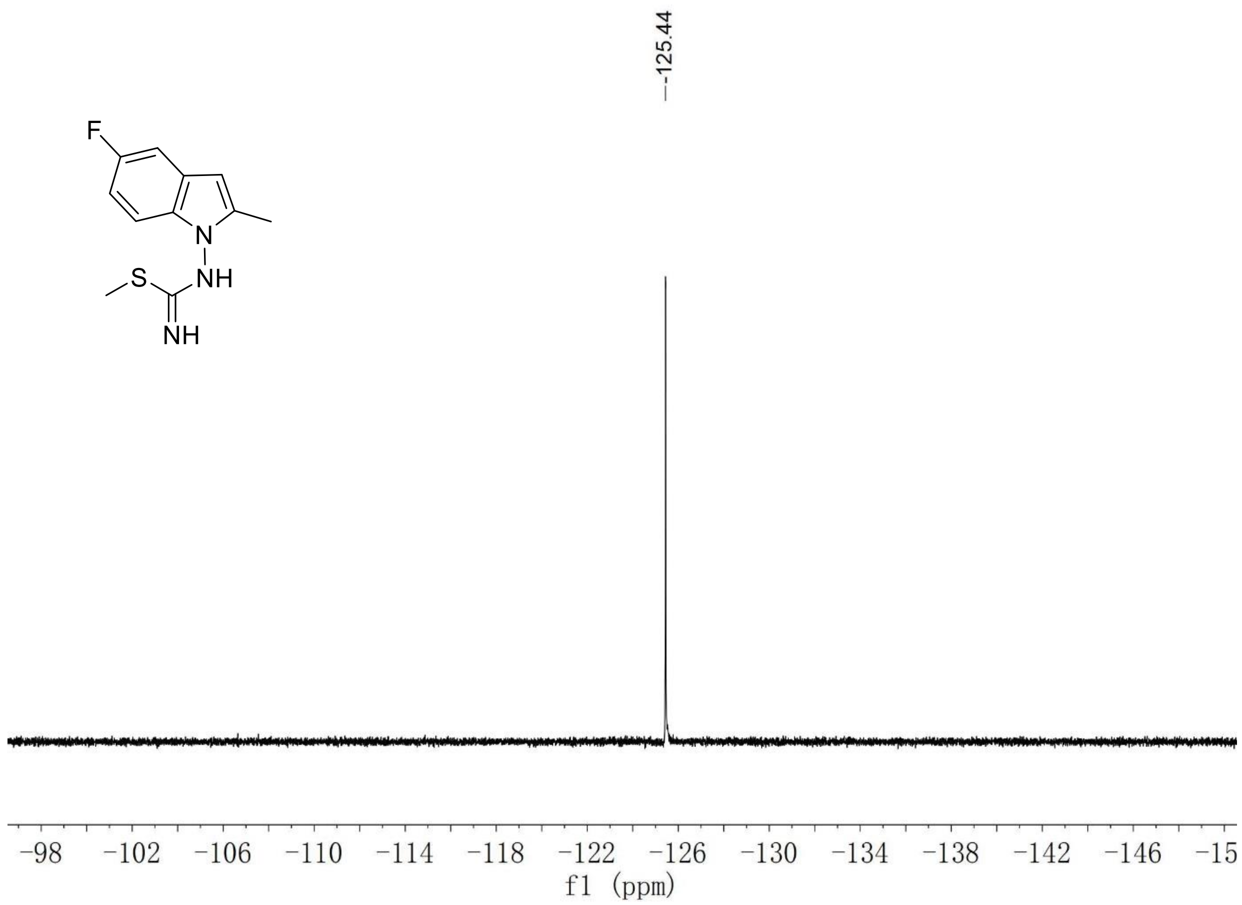

Supplementary Figure 11. ^{19}F NMR spectrum of compound 1n ($CDCl_3$, 471 MHz, 298 K)

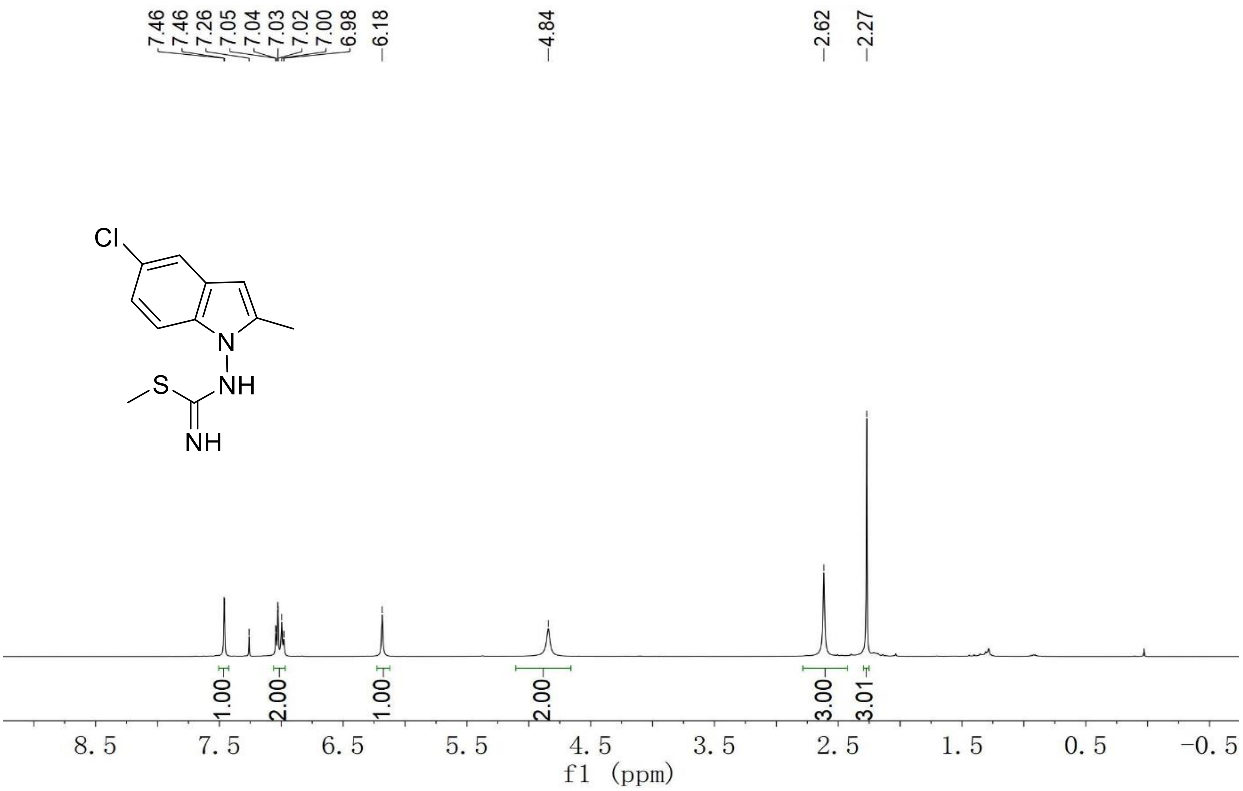

Supplementary Figure 12. ^{1}H NMR spectrum of compound 1o ($CDCl_3$, 500 MHz, 298 K)

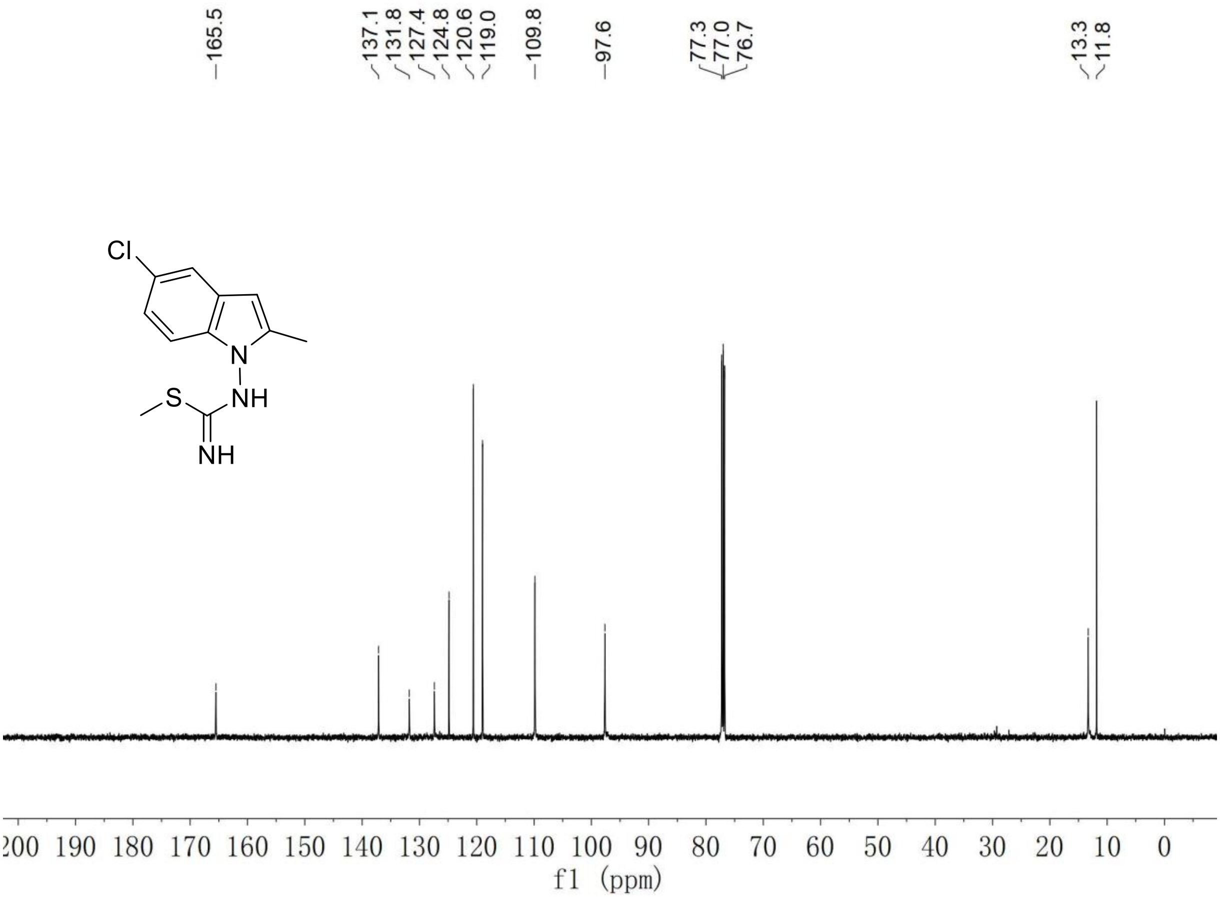

Supplementary Figure 13. ^{13}C NMR spectrum of compound 1o ($CDCl_3$, 126 MHz, 298 K)

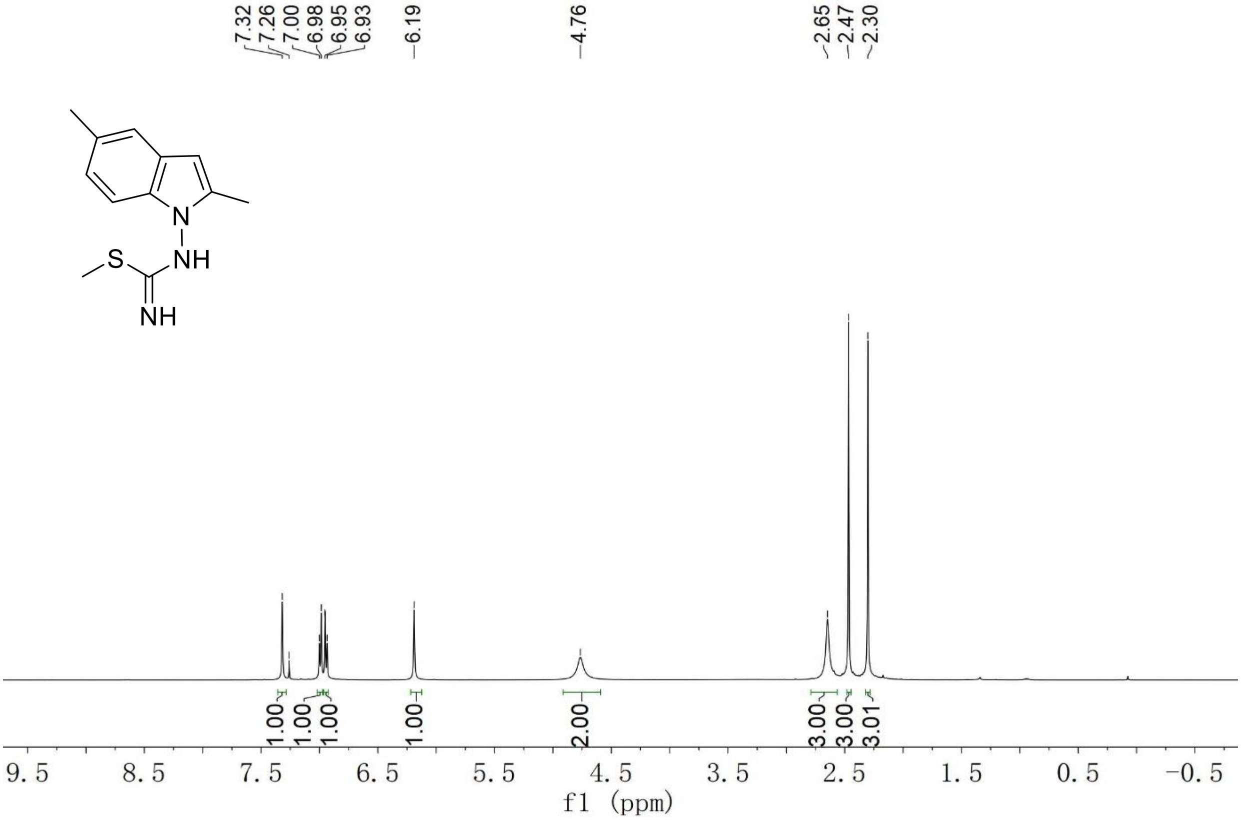

Supplementary Figure 14. ^{1}H NMR spectrum of compound 1p ($CDCl_3$, 500 MHz, 298 K)

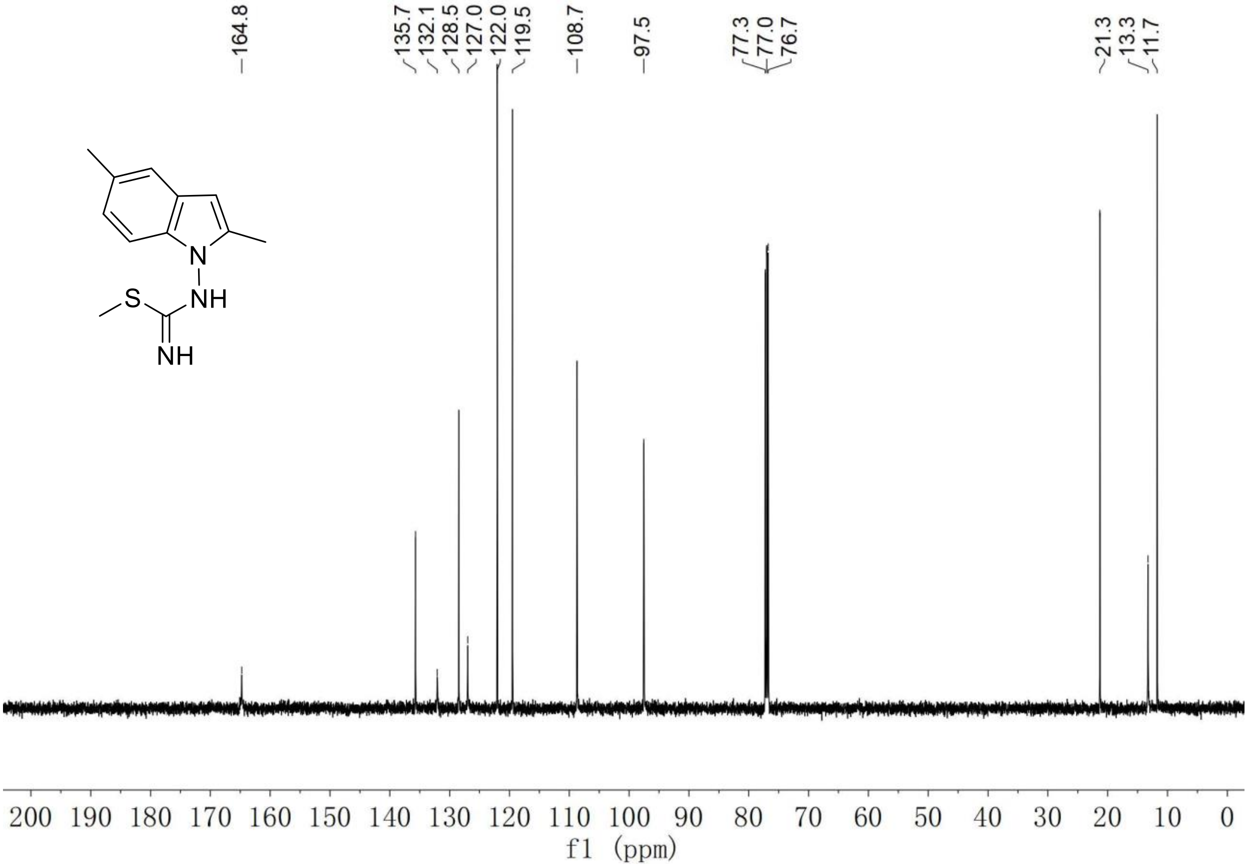

Supplementary Figure 15. ^{13}C NMR spectrum of compound 1p ($CDCl_3$, 126 MHz, 298 K)

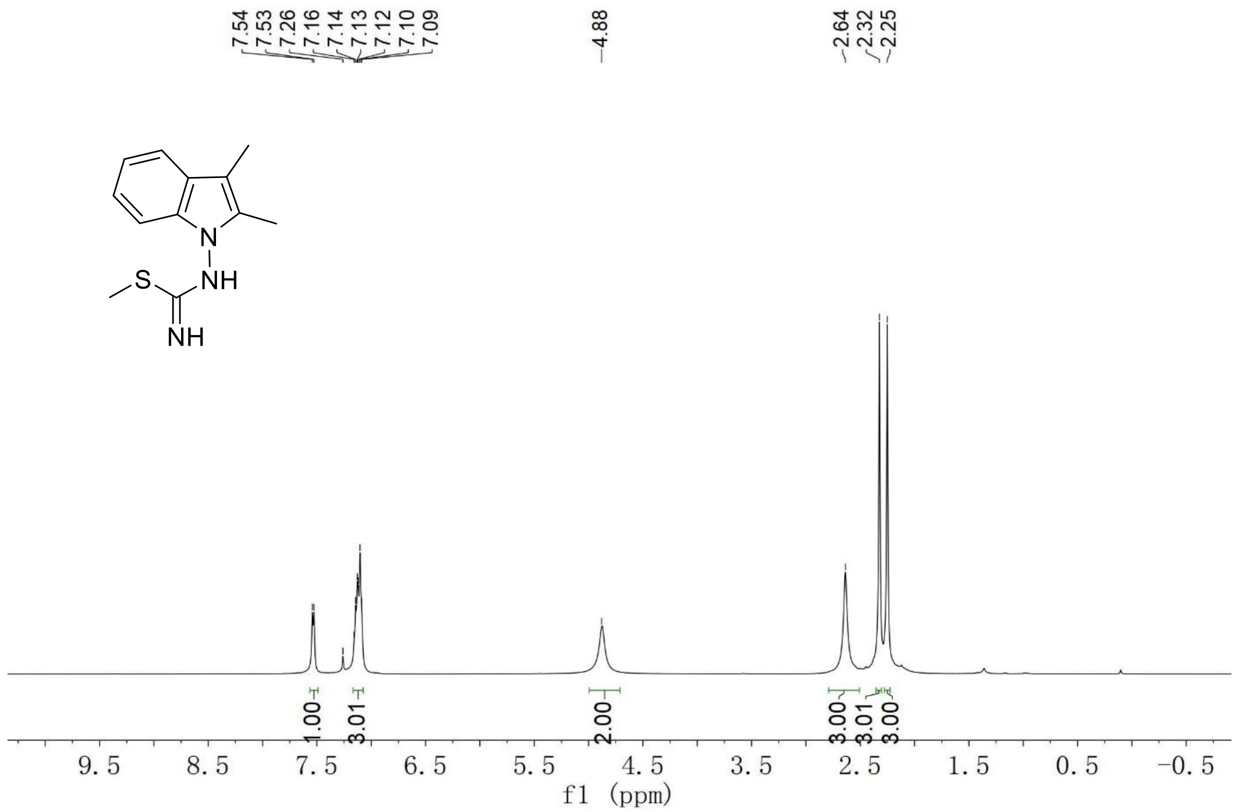

Supplementary Figure 16. ^{1}H NMR spectrum of compound 1q ($CDCl_3$, 500 MHz, 298 K)

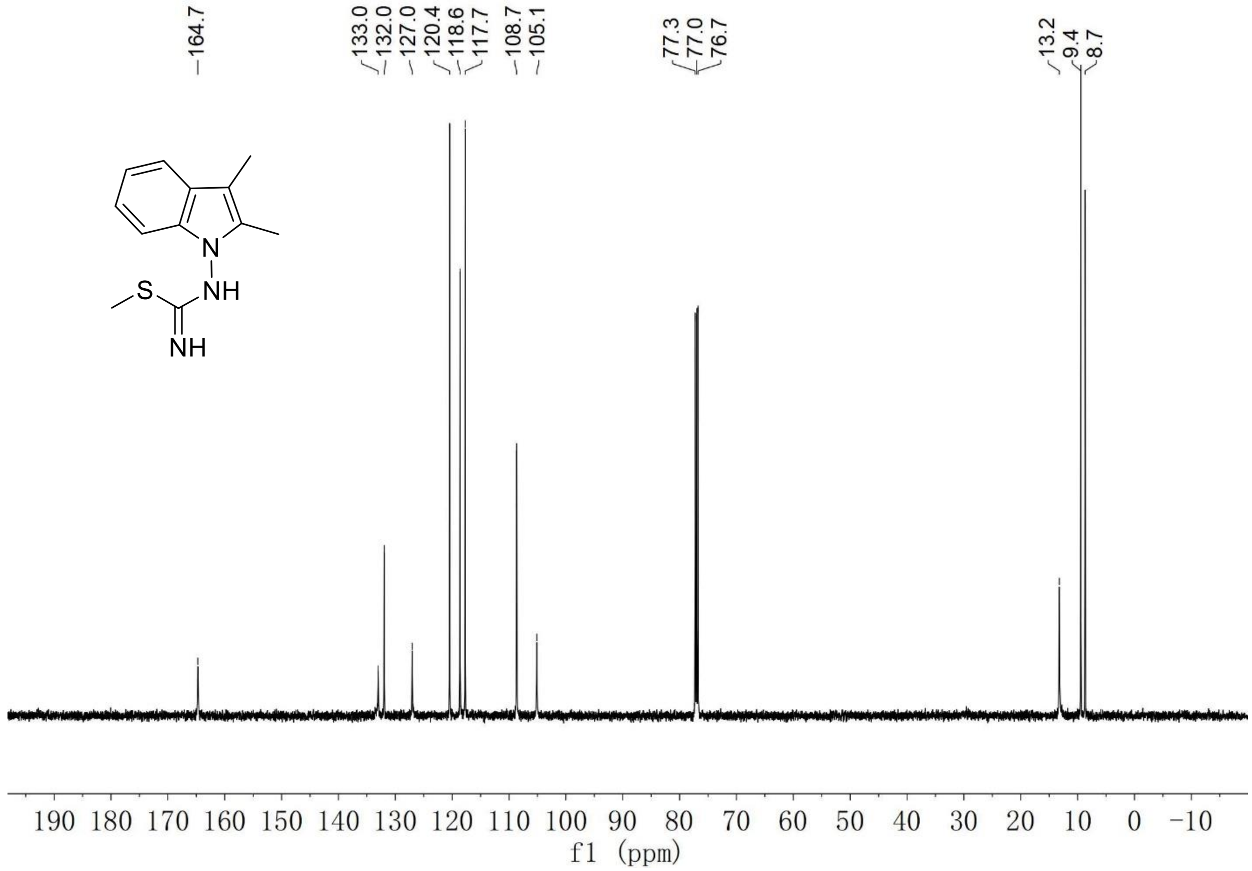

Supplementary Figure 17. ^{13}C NMR spectrum of compound 1q ($CDCl_3$, 126 MHz, 298 K)

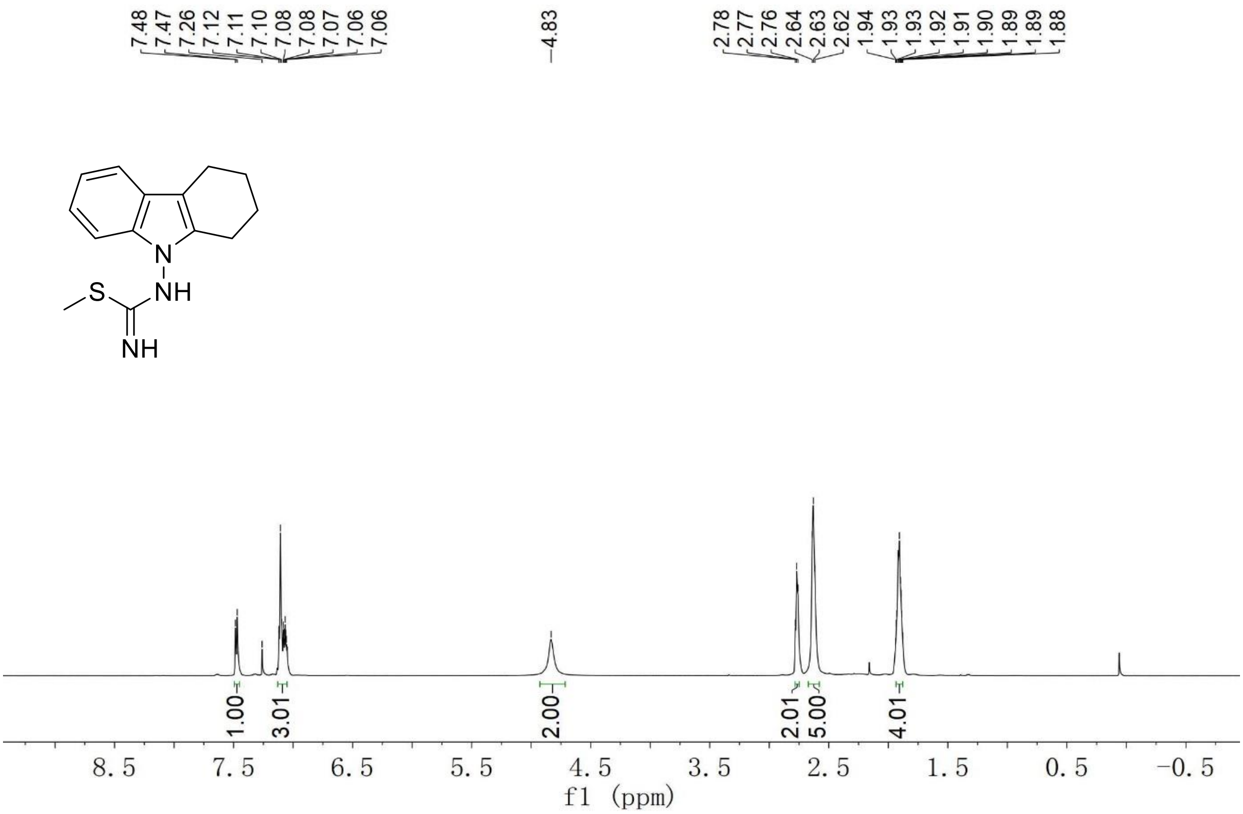

Supplementary Figure 18. ^{1}H NMR spectrum of compound 1r ($CDCl_3$, 500 MHz, 298 K)

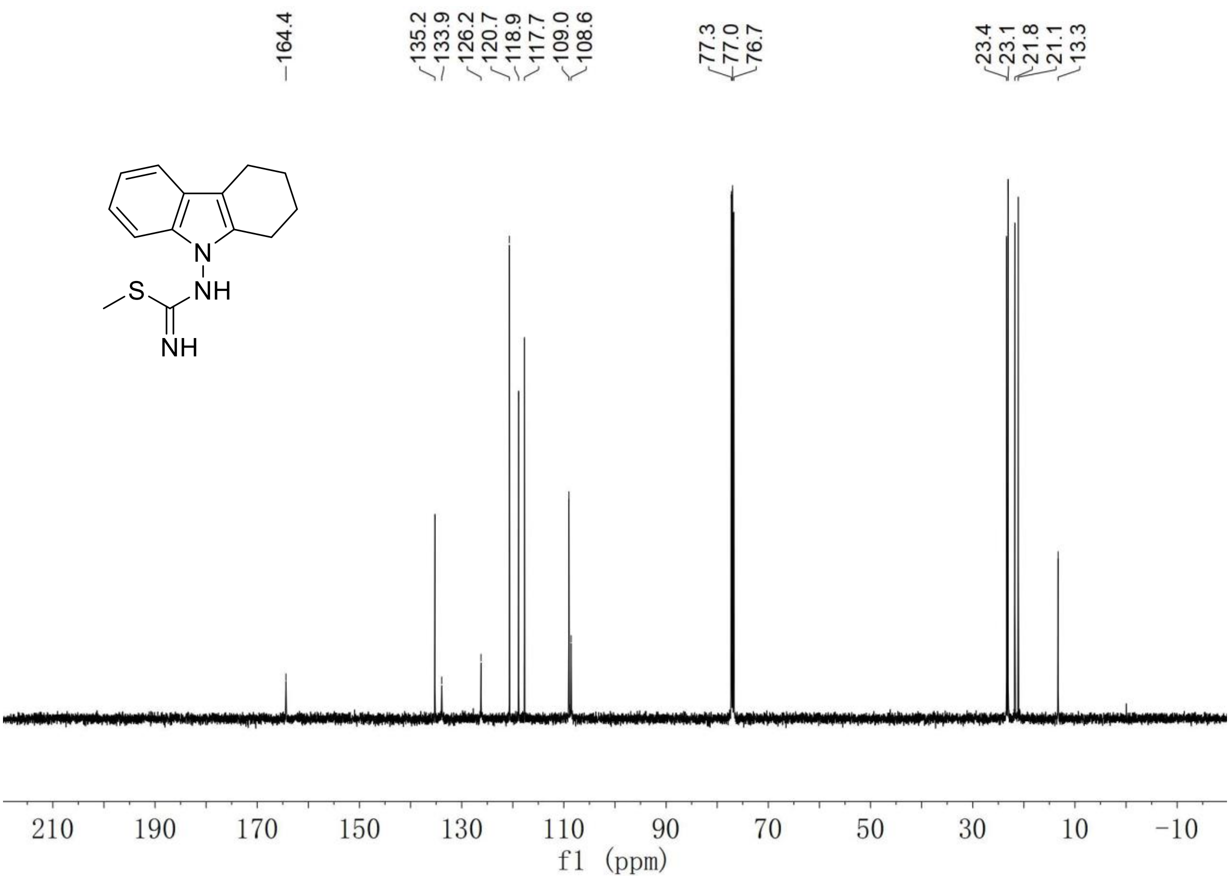

Supplementary Figure 19. ^{13}C NMR spectrum of compound 1r ($CDCl_3$, 126 MHz, 298 K)

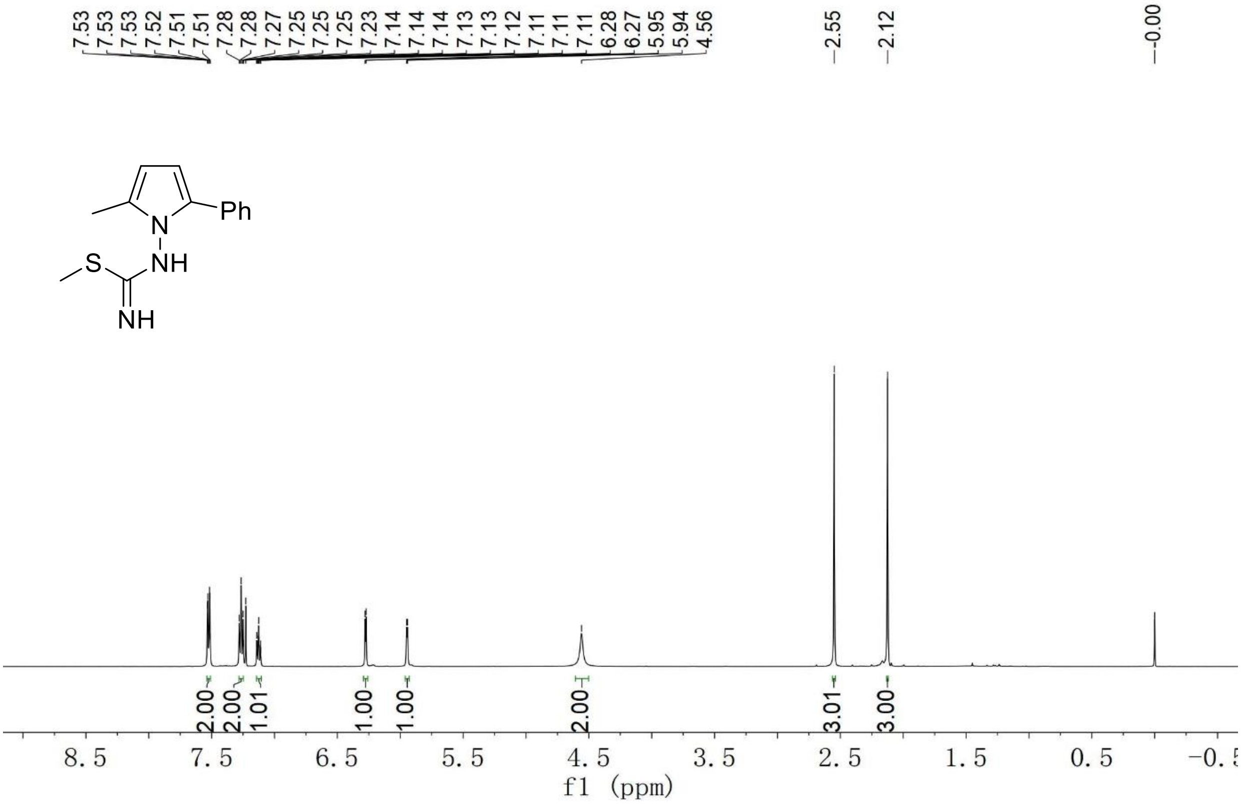

Supplementary Figure 20. ^{1}H NMR spectrum of compound 1s ($CDCl_3$, 500 MHz, 298 K)

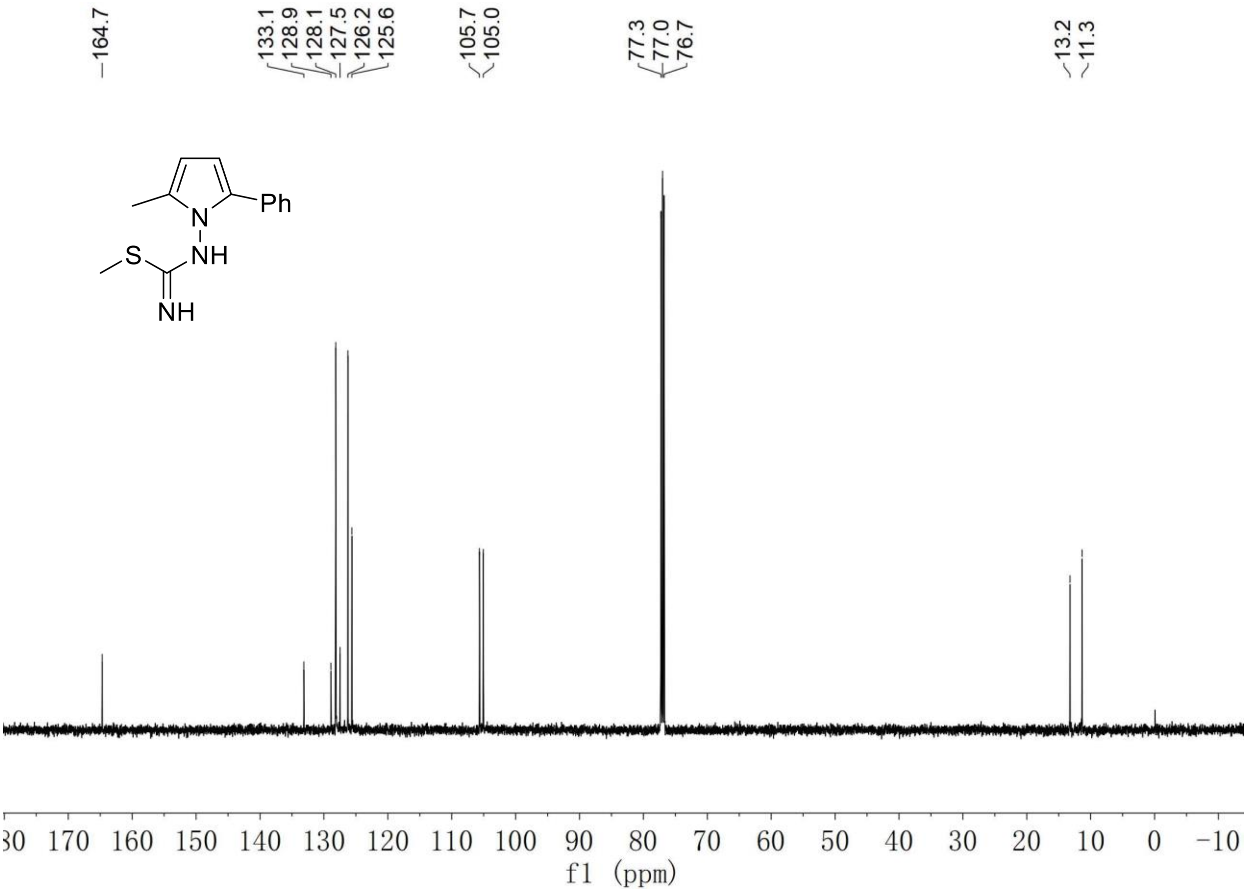

Supplementary Figure 21. ^{13}C NMR spectrum of compound 1s ($CDCl_3$, 126 MHz, 298 K)

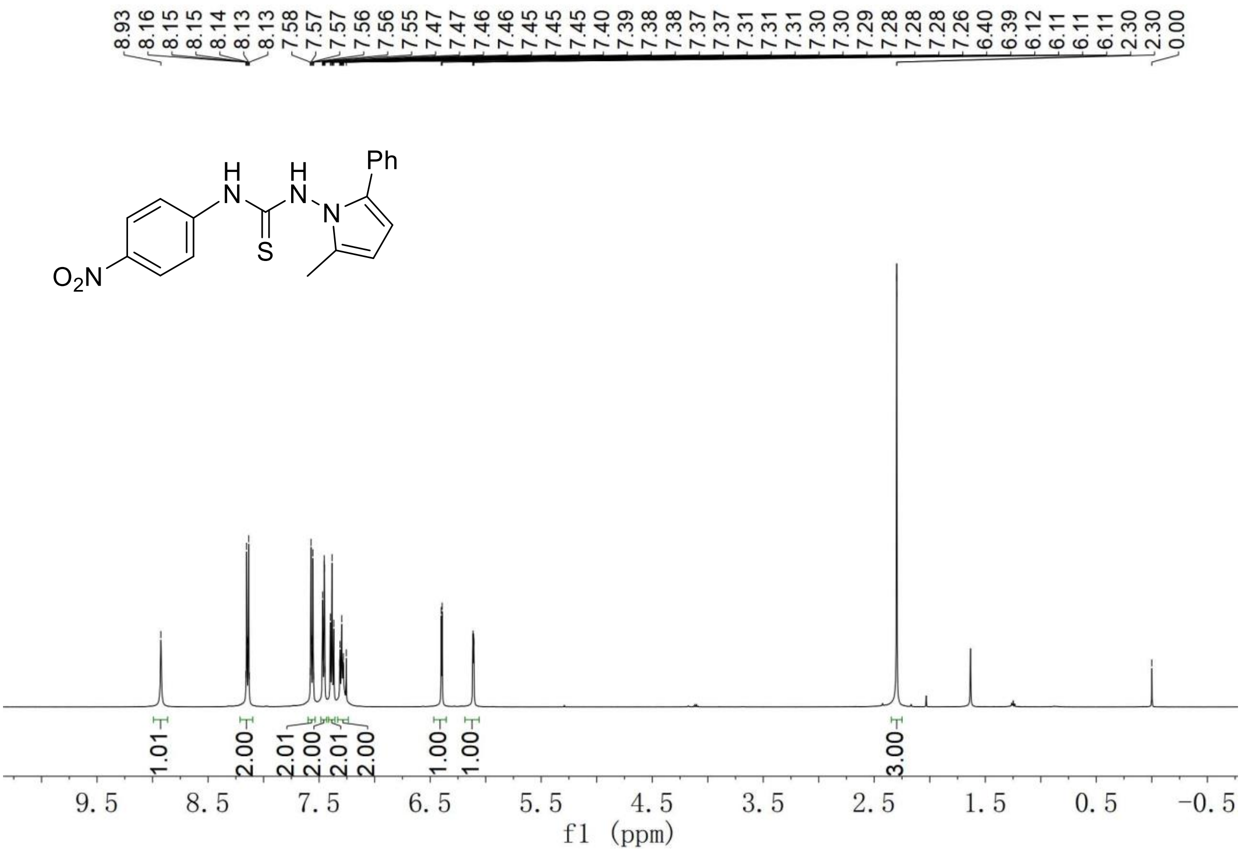

Supplementary Figure 22. **^{1}H NMR spectrum of compound 5a ($CDCl_3$, 500 MHz, 298 K)**

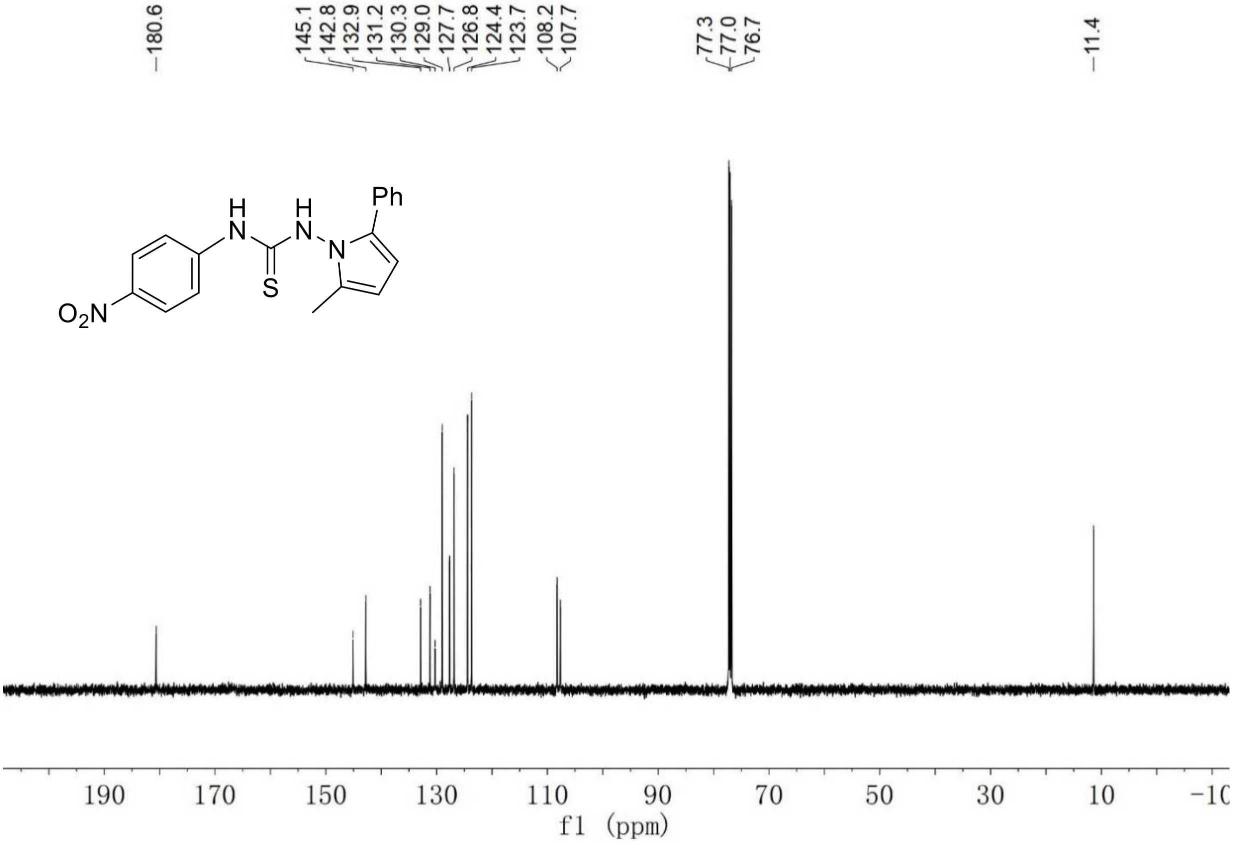

Supplementary Figure 23. **^{13}C NMR spectrum of compound 5a ($CDCl_3$, 126 MHz, 298 K)**

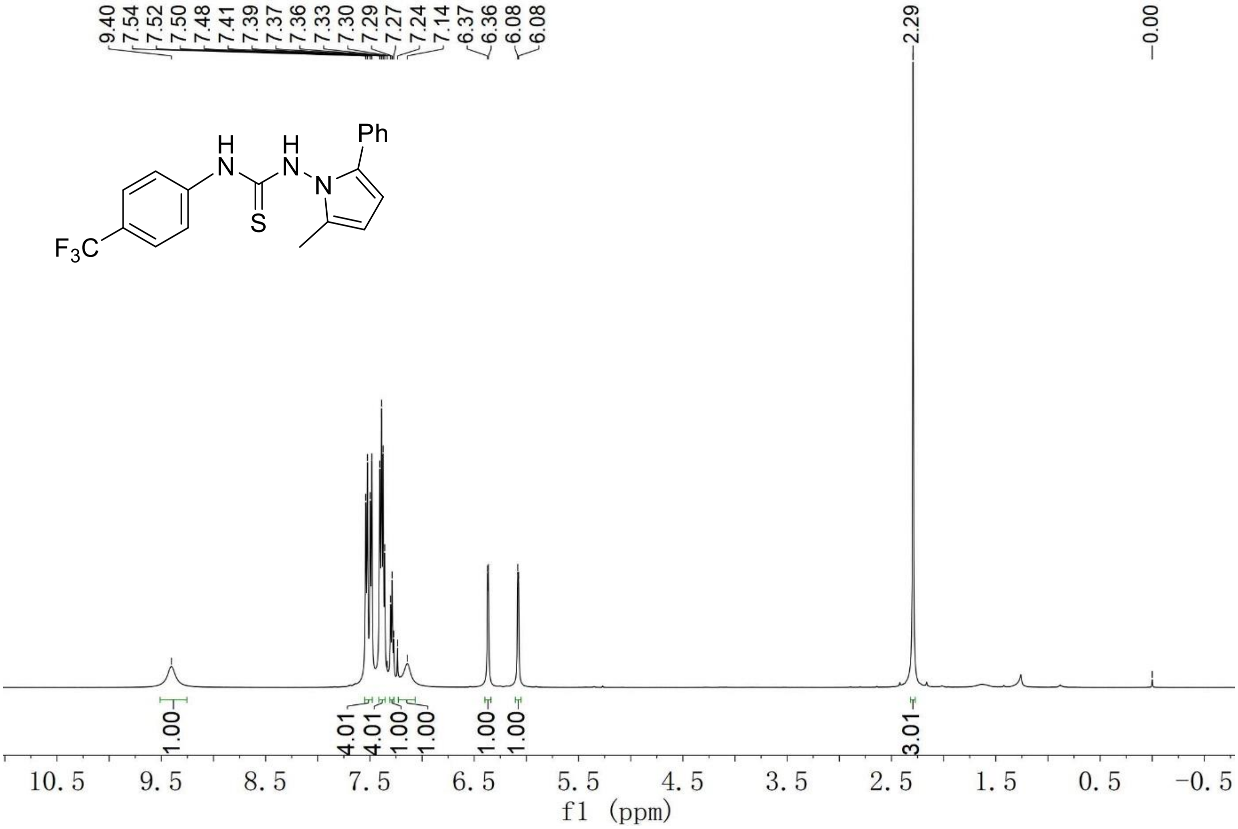

Supplementary Figure 24. ^{1}**H NMR spectrum of compound 5b (CDCl$_3$, 500 MHz, 298 K)**

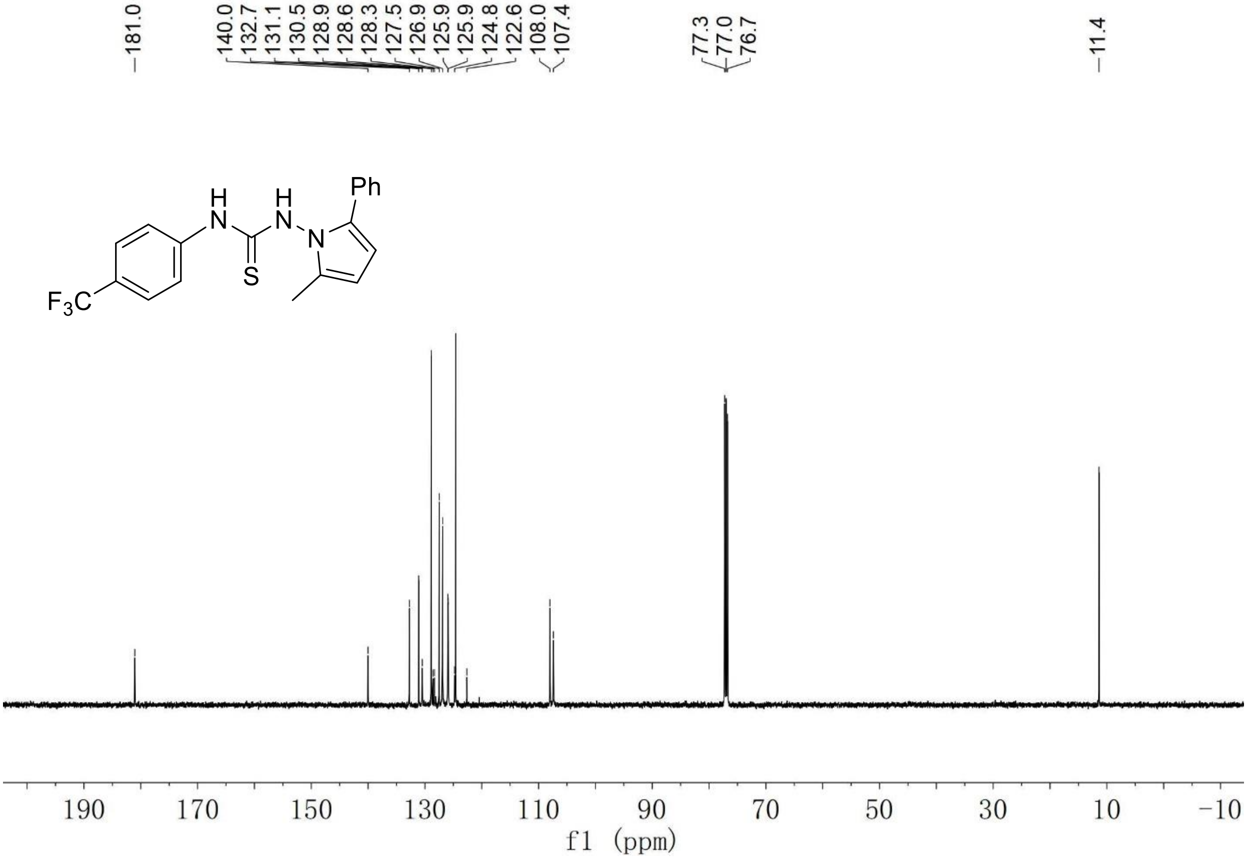

Supplementary Figure 25. ^{13}**C NMR spectrum of compound 5b (CDCl$_3$, 126 MHz, 298 K)**

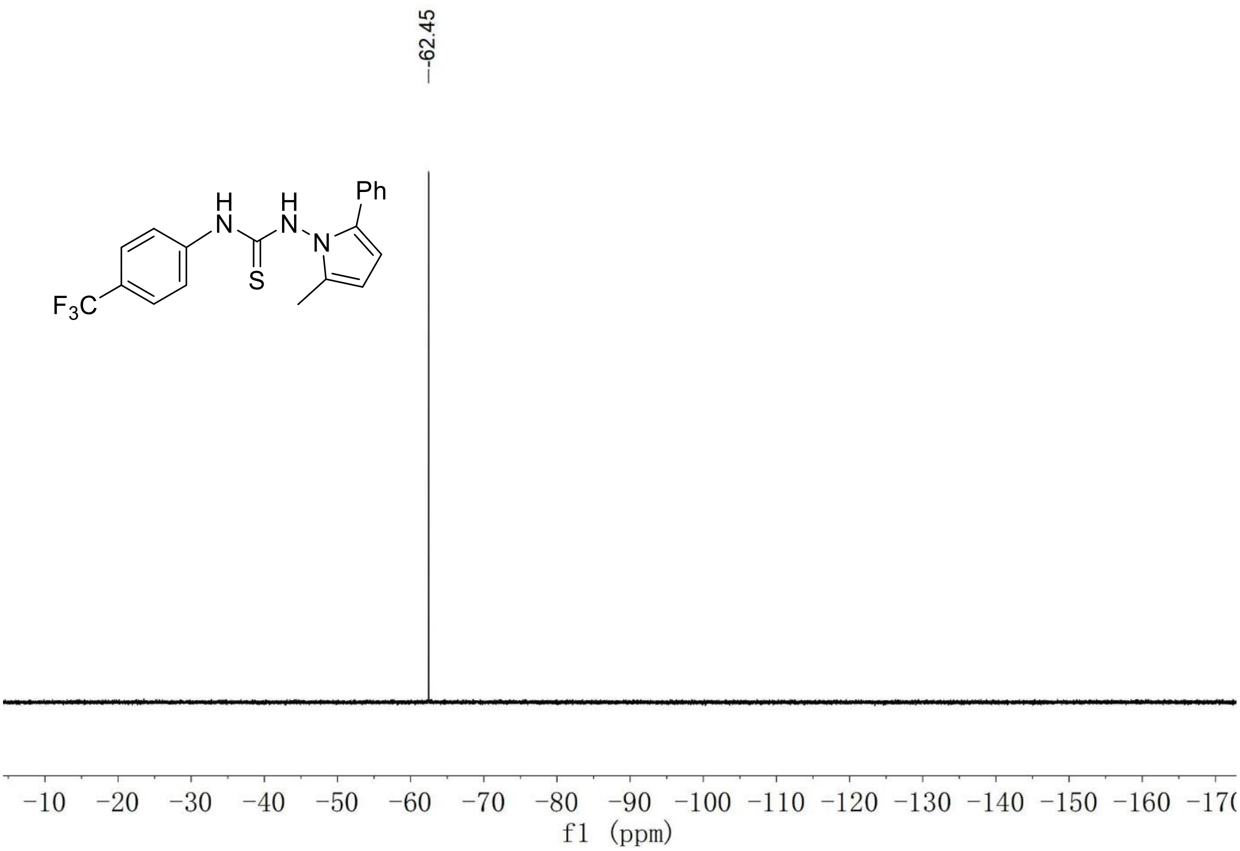

Supplementary Figure 26. ^{19}F NMR spectrum of compound 5b ($CDCl_3$, 471 MHz, 298 K)

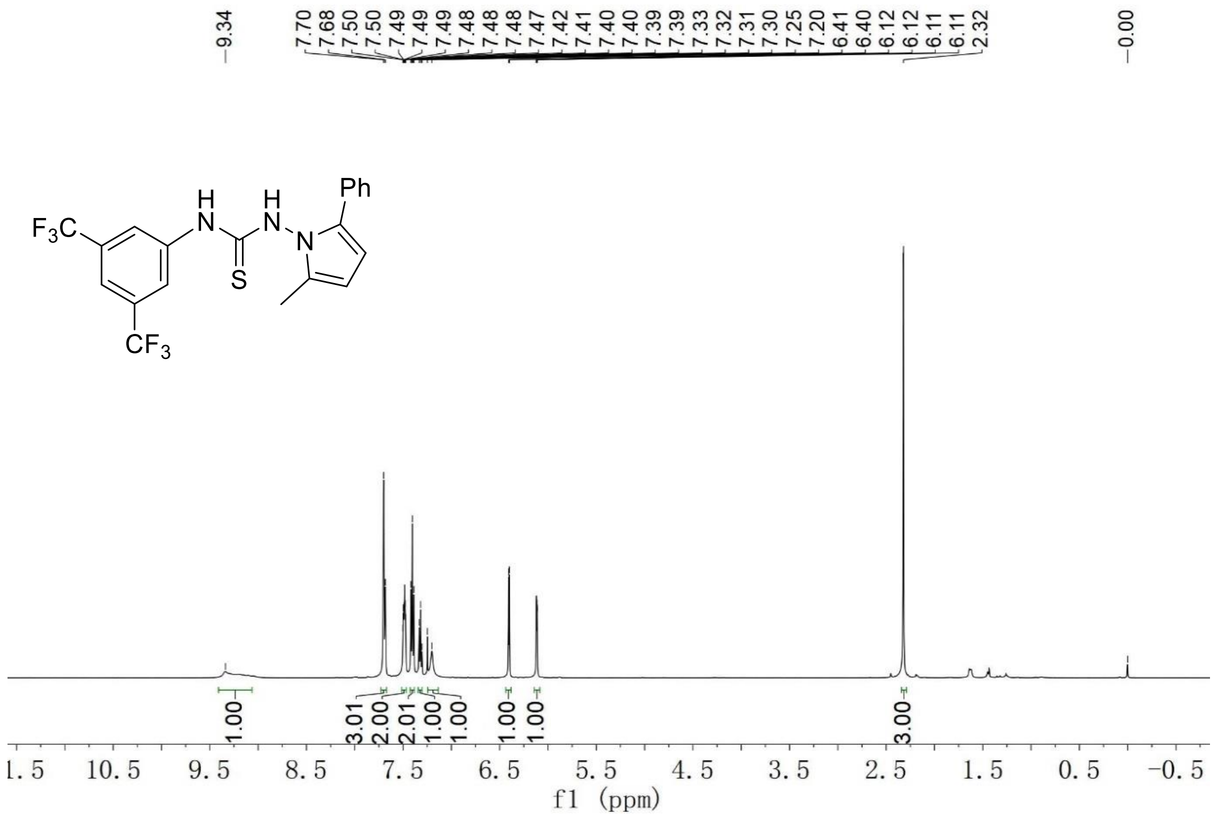

Supplementary Figure 27. ^{1}H NMR spectrum of compound 5c ($CDCl_3$, 500 MHz, 298 K)

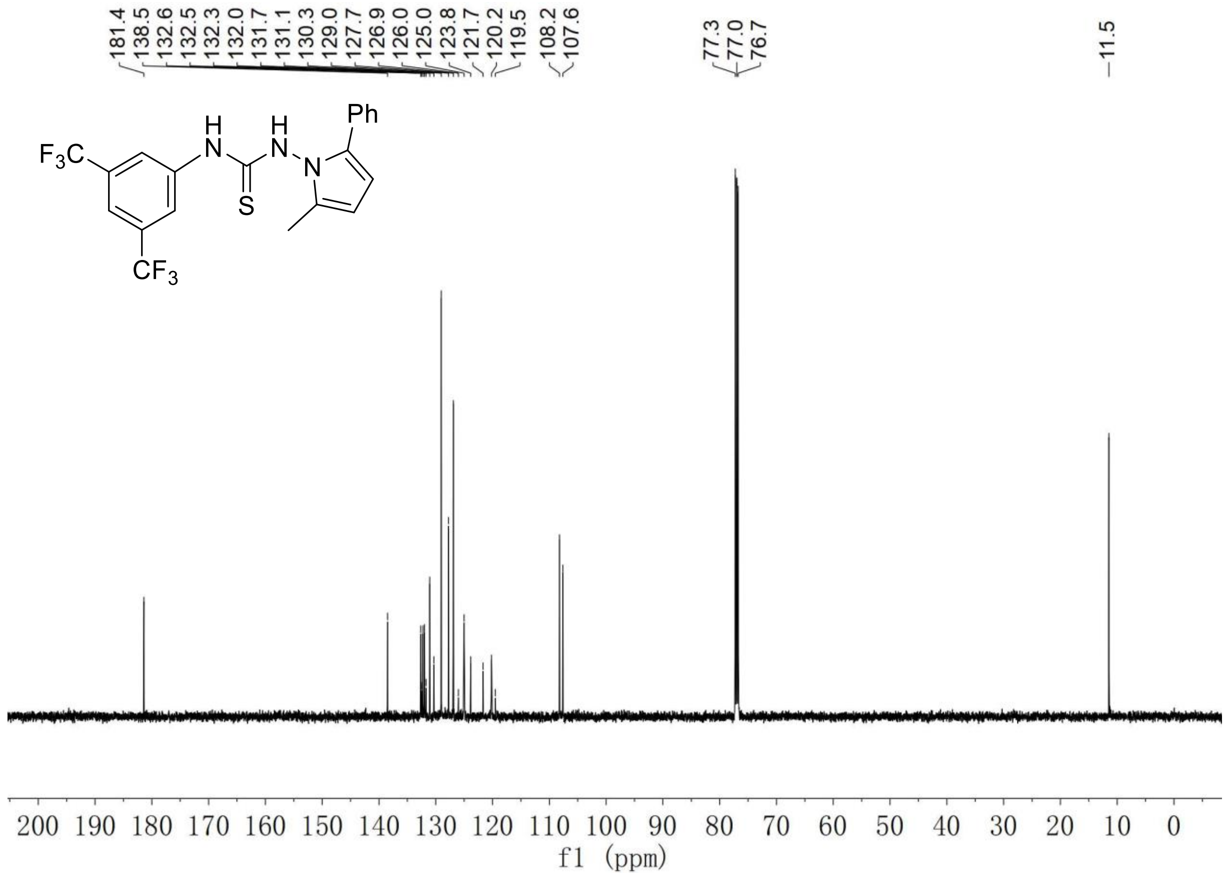

Supplementary Figure 28. ^{13}C NMR spectrum of compound 5c ($CDCl_3$, 126 MHz, 298 K)

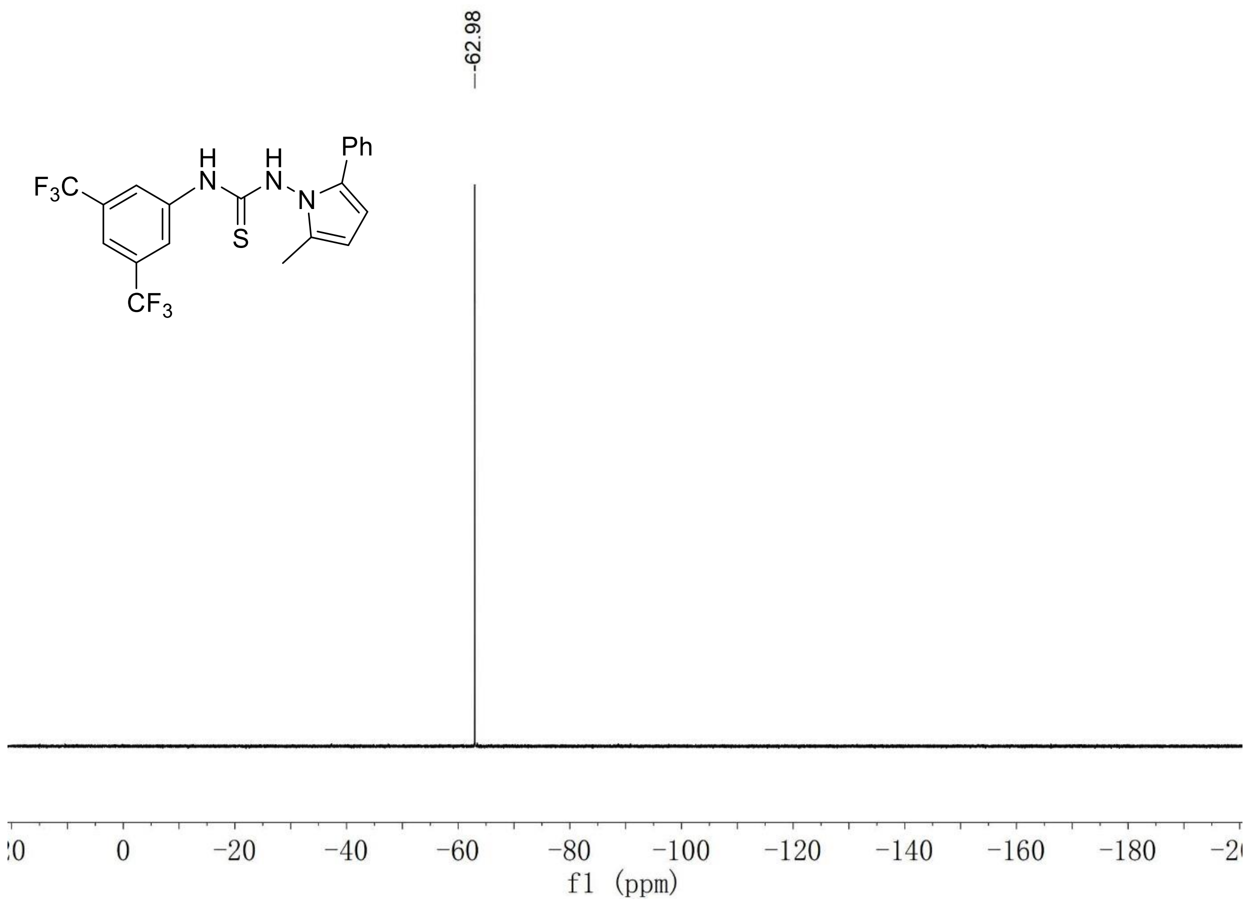

Supplementary Figure 29. ^{19}F NMR spectrum of compound 5c ($CDCl_3$, 471 MHz, 298 K)

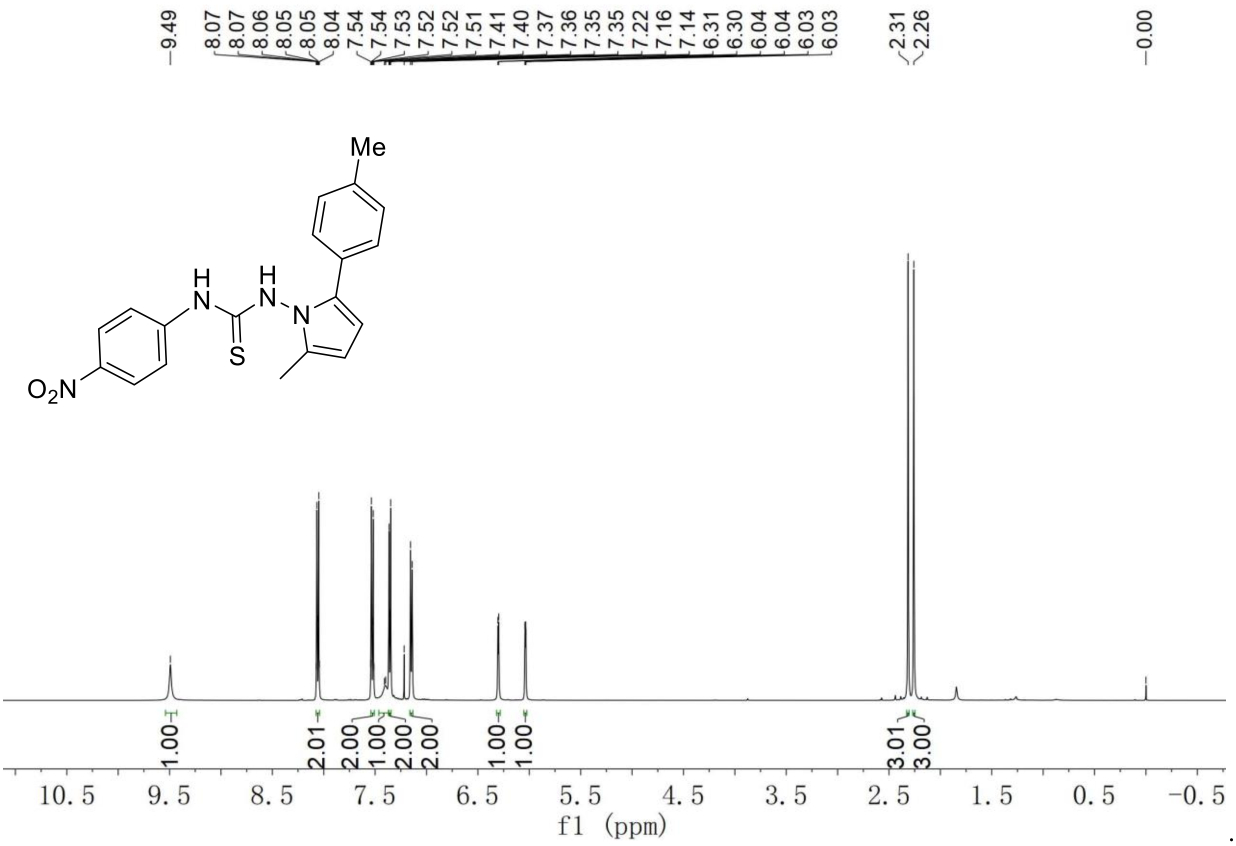

Supplementary Figure 30. ^{1}H NMR spectrum of compound 5d ($CDCl_3$, 500 MHz, 298 K)

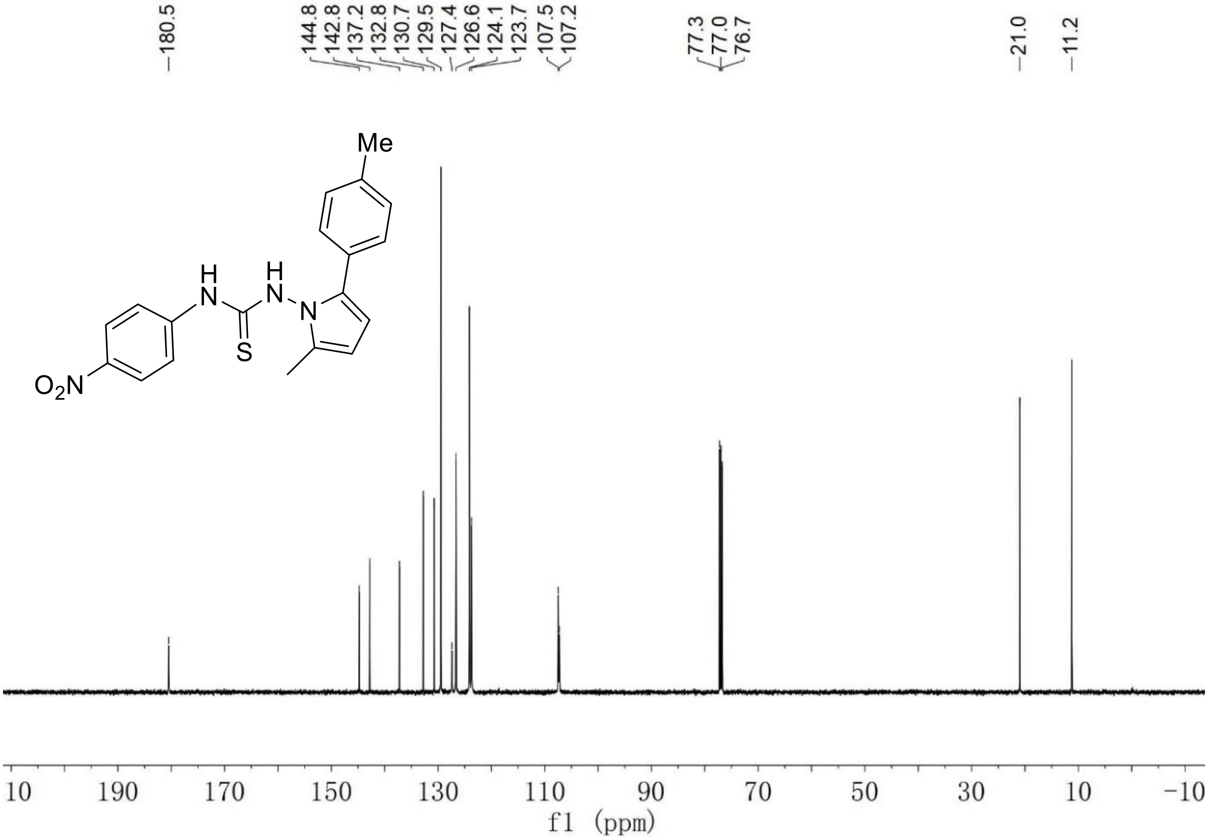

Supplementary Figure 31. ^{13}C NMR spectrum of compound 5d ($CDCl_3$, 126 MHz, 298 K)

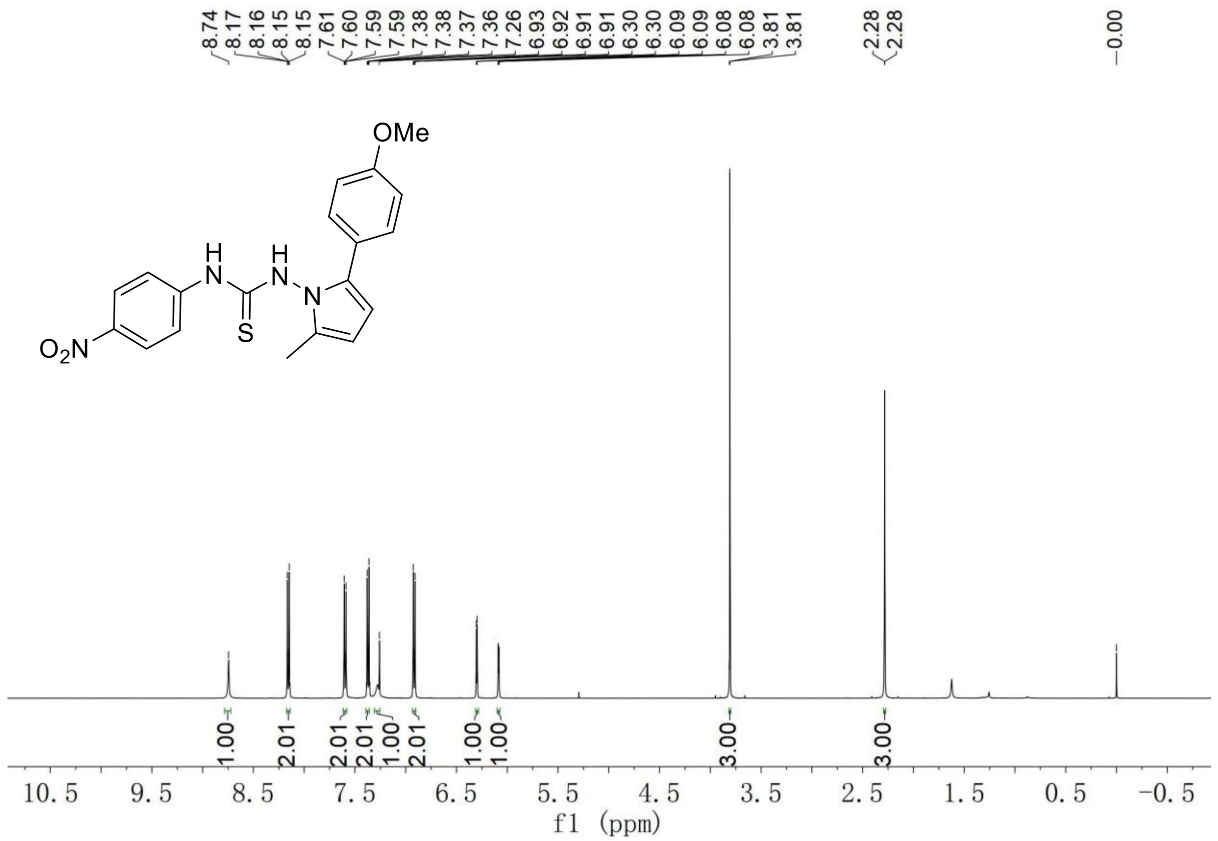

Supplementary Figure 32. ^{1}H NMR spectrum of compound 5e ($CDCl_3$, 500 MHz, 298 K)

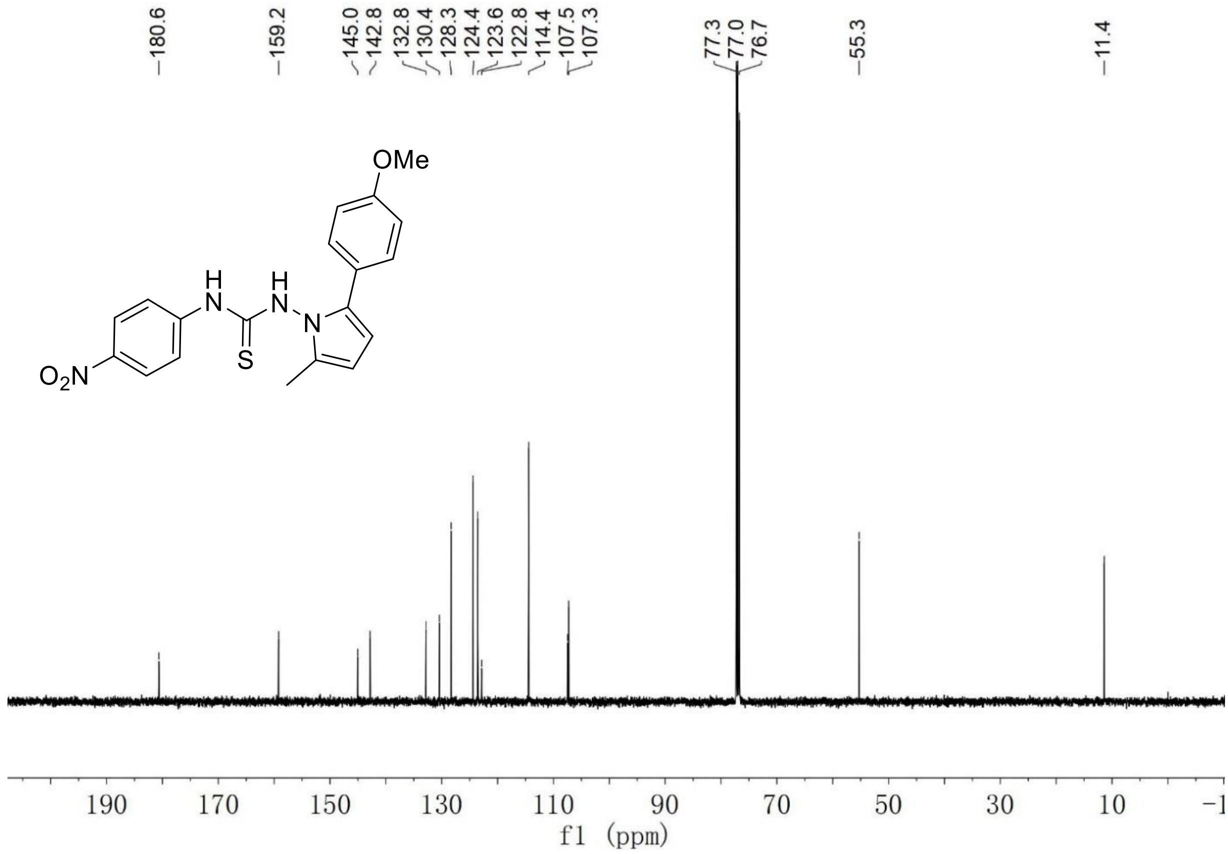

Supplementary Figure 33. ^{13}C NMR spectrum of compound 5e ($CDCl_3$, 126 MHz, 298 K)

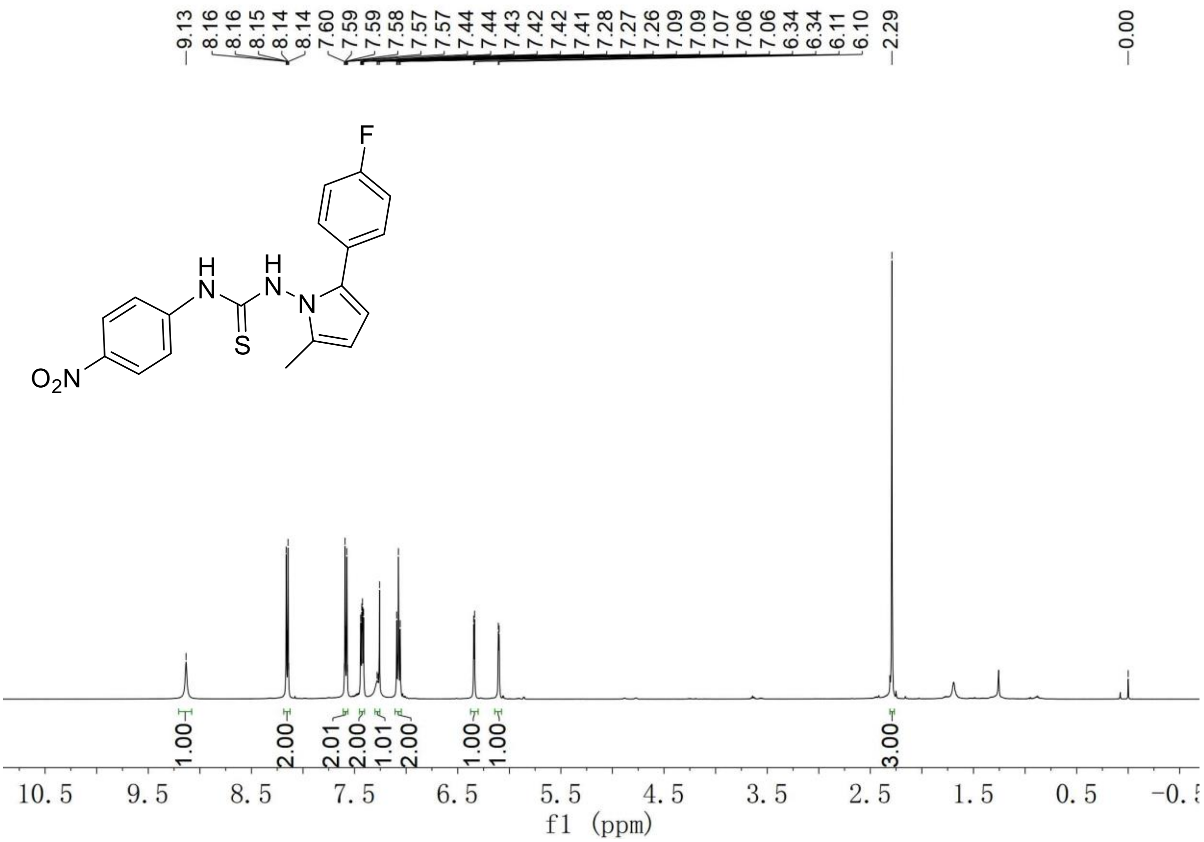

Supplementary Figure 34. ^{1}H NMR spectrum of compound 5f ($CDCl_3$, 500 MHz, 298 K)

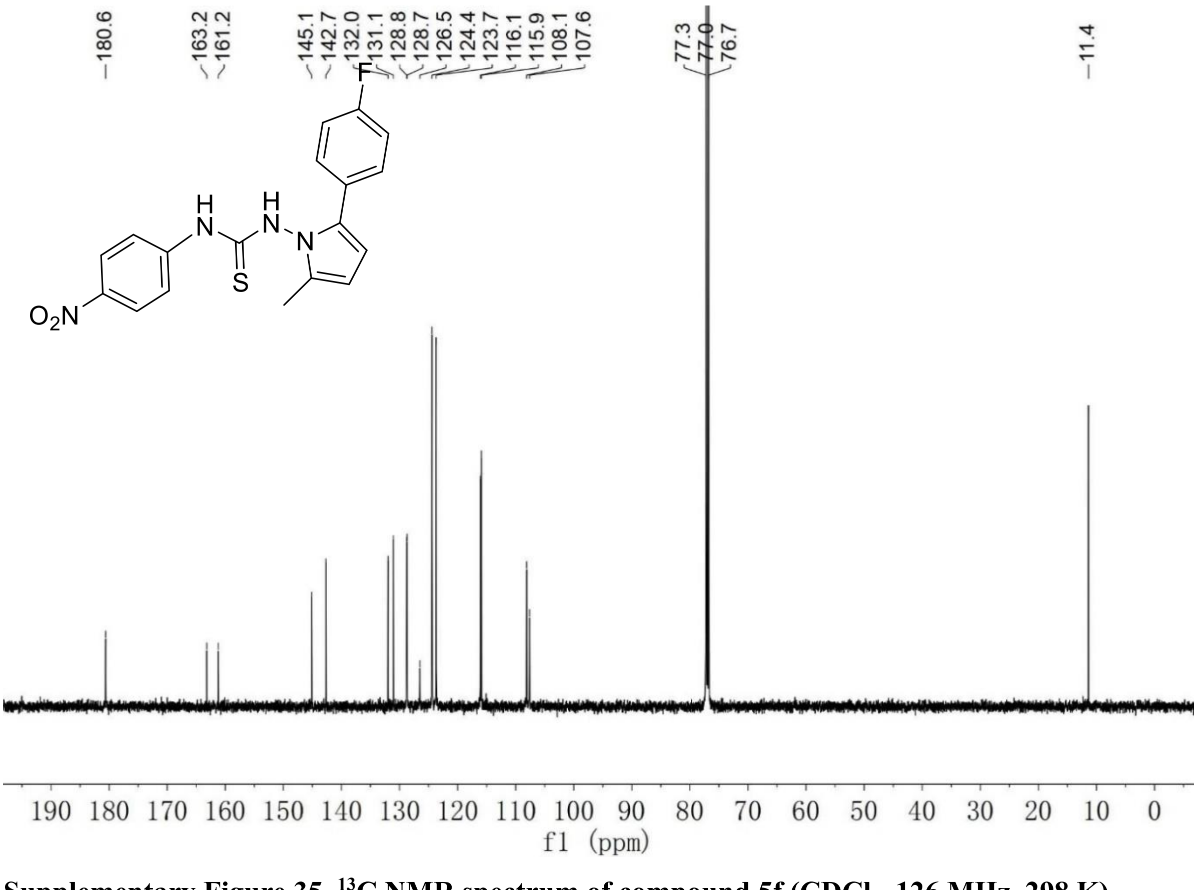

Supplementary Figure 35. ^{13}C NMR spectrum of compound 5f ($CDCl_3$, 126 MHz, 298 K)

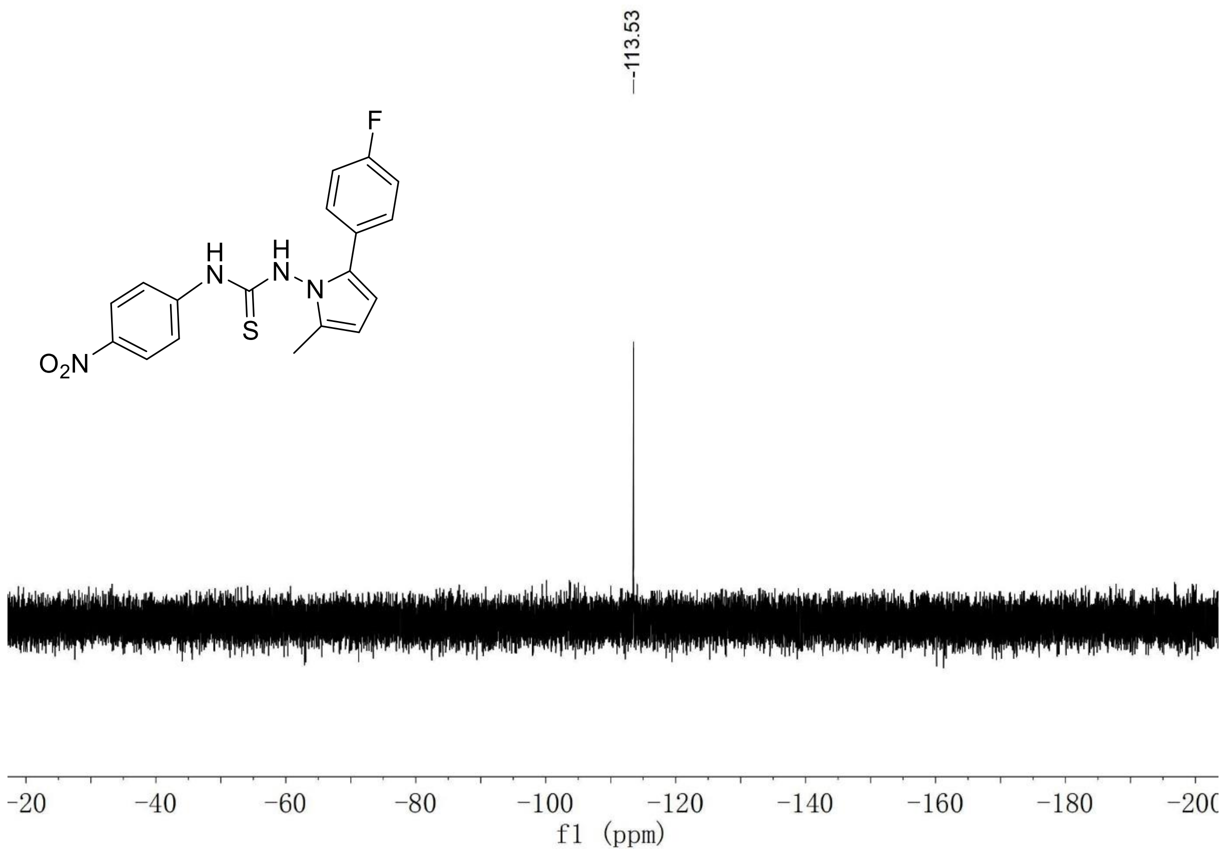

Supplementary Figure 36. ^{19}F NMR spectrum of compound 5f ($CDCl_3$, 471 MHz, 298 K)

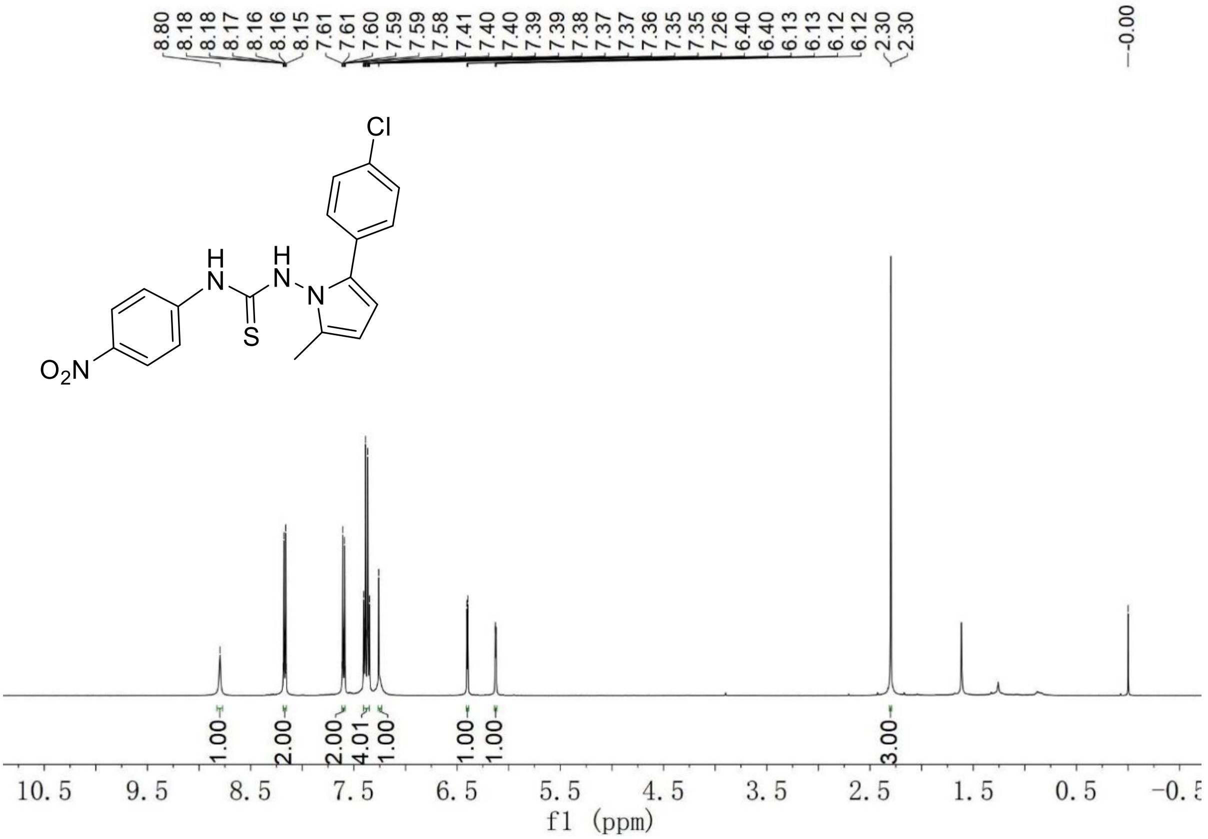

Supplementary Figure 37. ^{1}H NMR spectrum of compound 5g ($CDCl_3$, 500 MHz, 298 K)

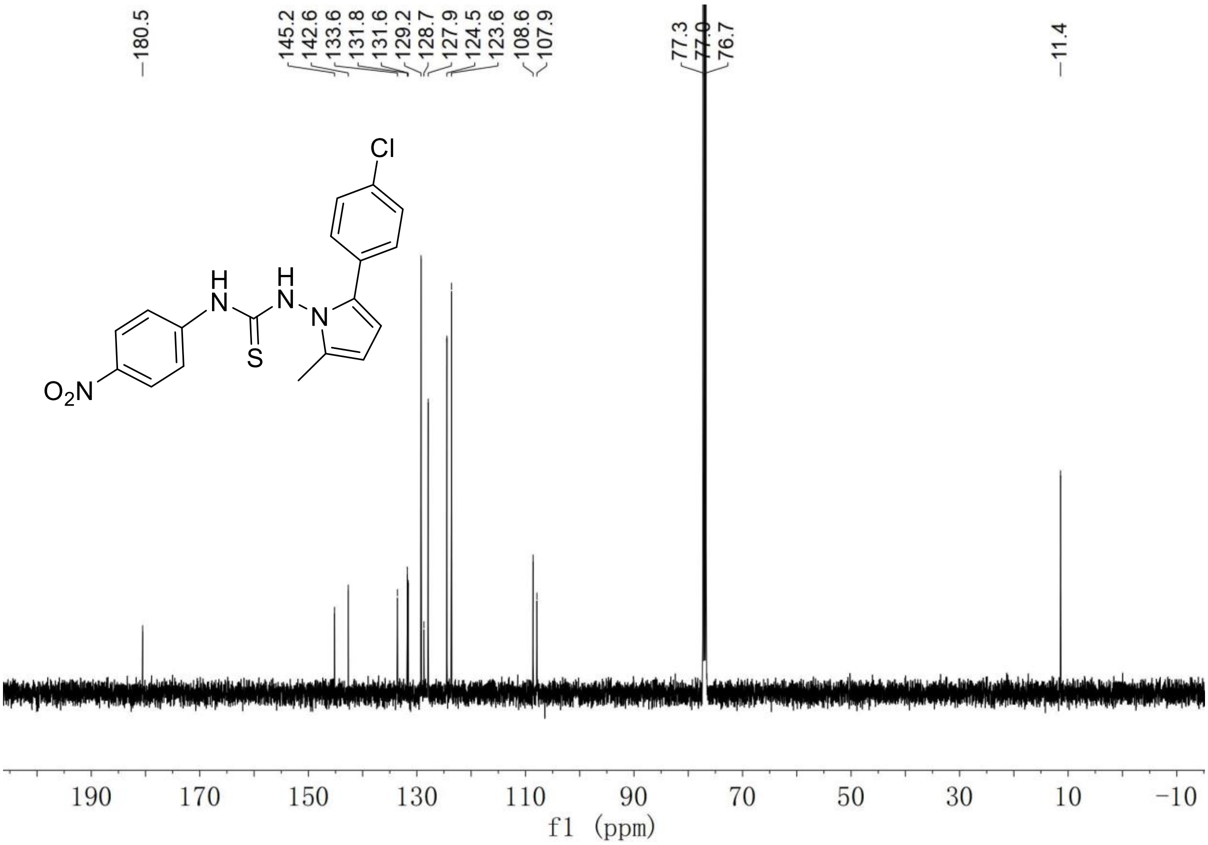

Supplementary Figure 38. ^{13}C NMR spectrum of compound 5g ($CDCl_3$, 126 MHz, 298 K)

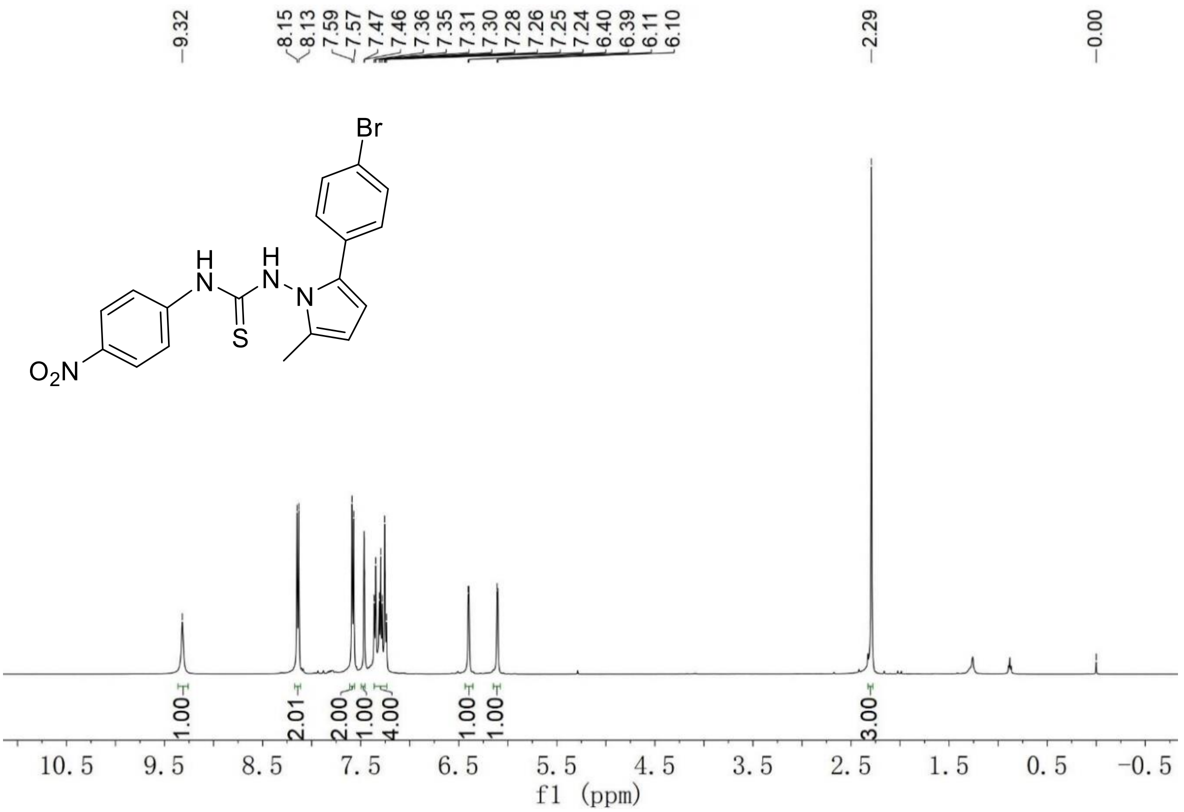

Supplementary Figure 39. ^{1}H NMR spectrum of compound 5h ($CDCl_3$, 500 MHz, 298 K)

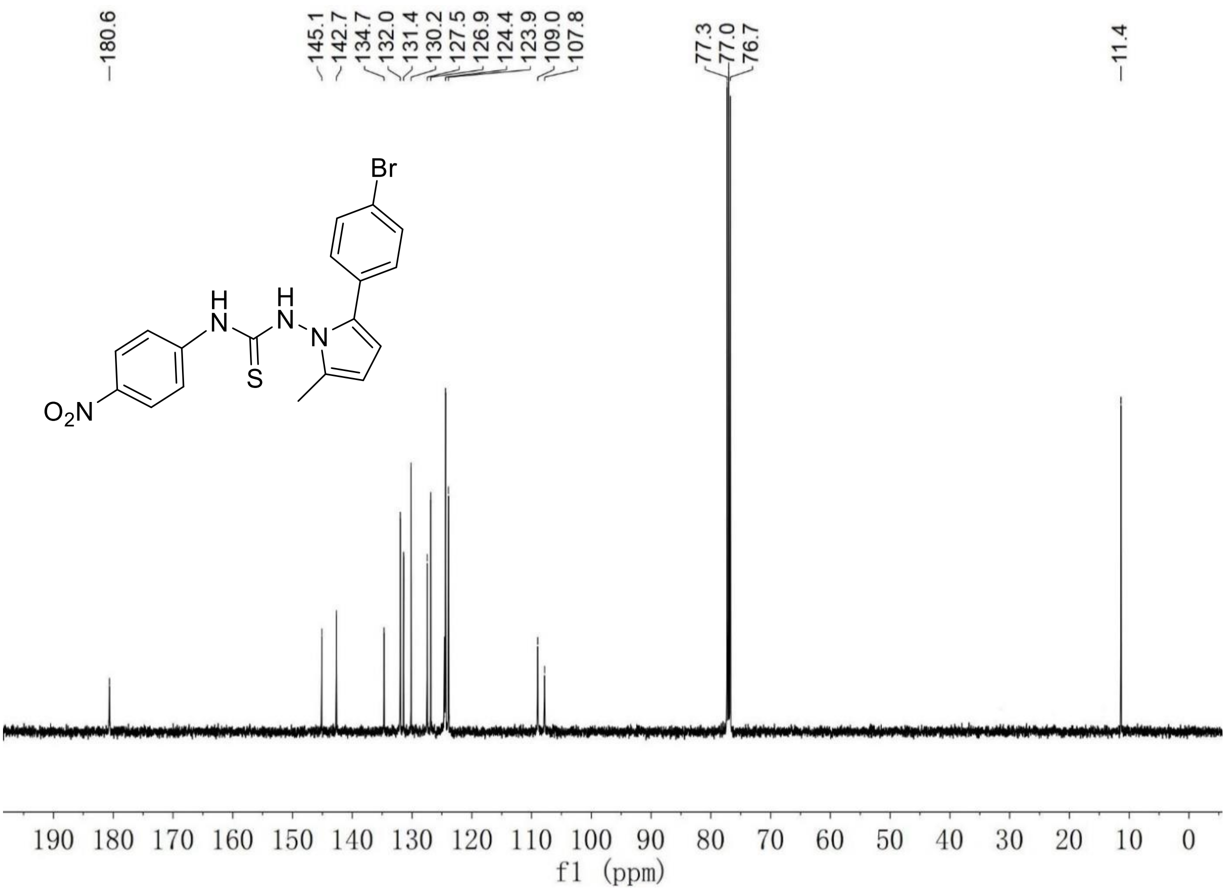

Supplementary Figure 40. ^{13}C NMR spectrum of compound 5h ($CDCl_3$, 126 MHz, 298 K)

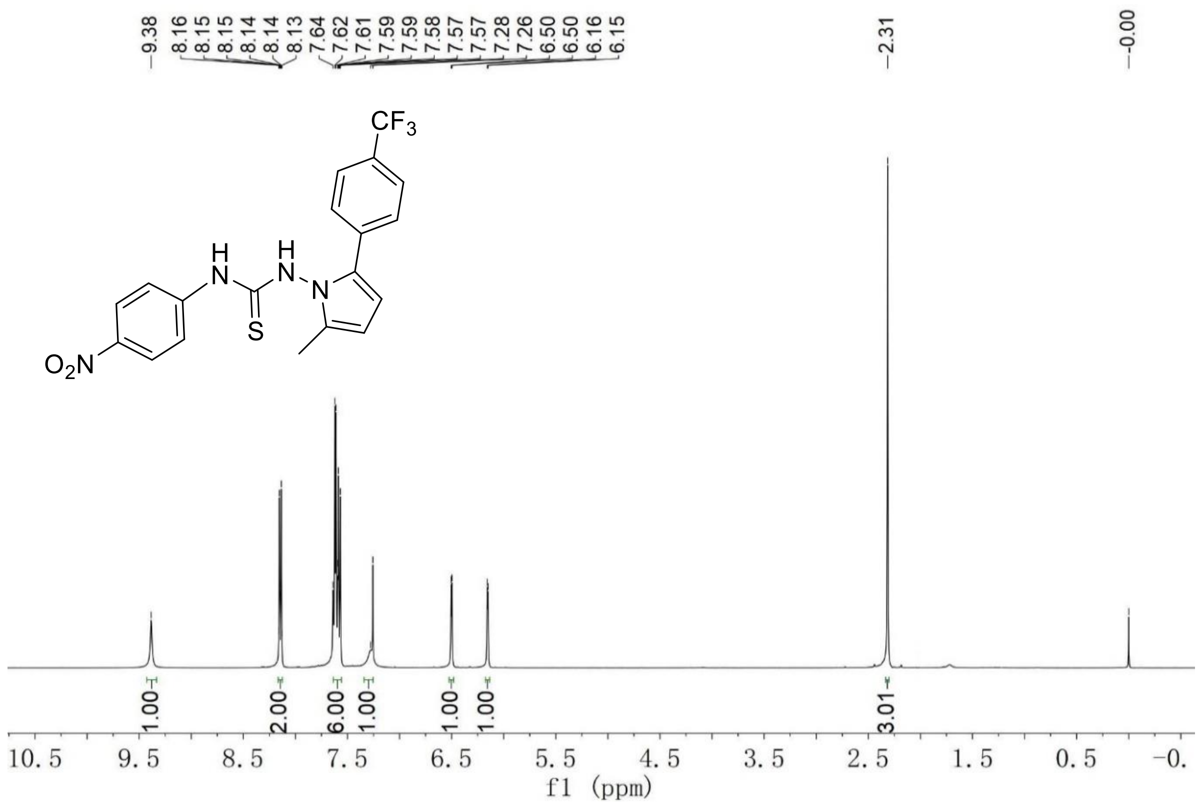

Supplementary Figure 41. ^{1}**H NMR spectrum of compound 5i (CDCl$_3$, 500 MHz, 298 K)**

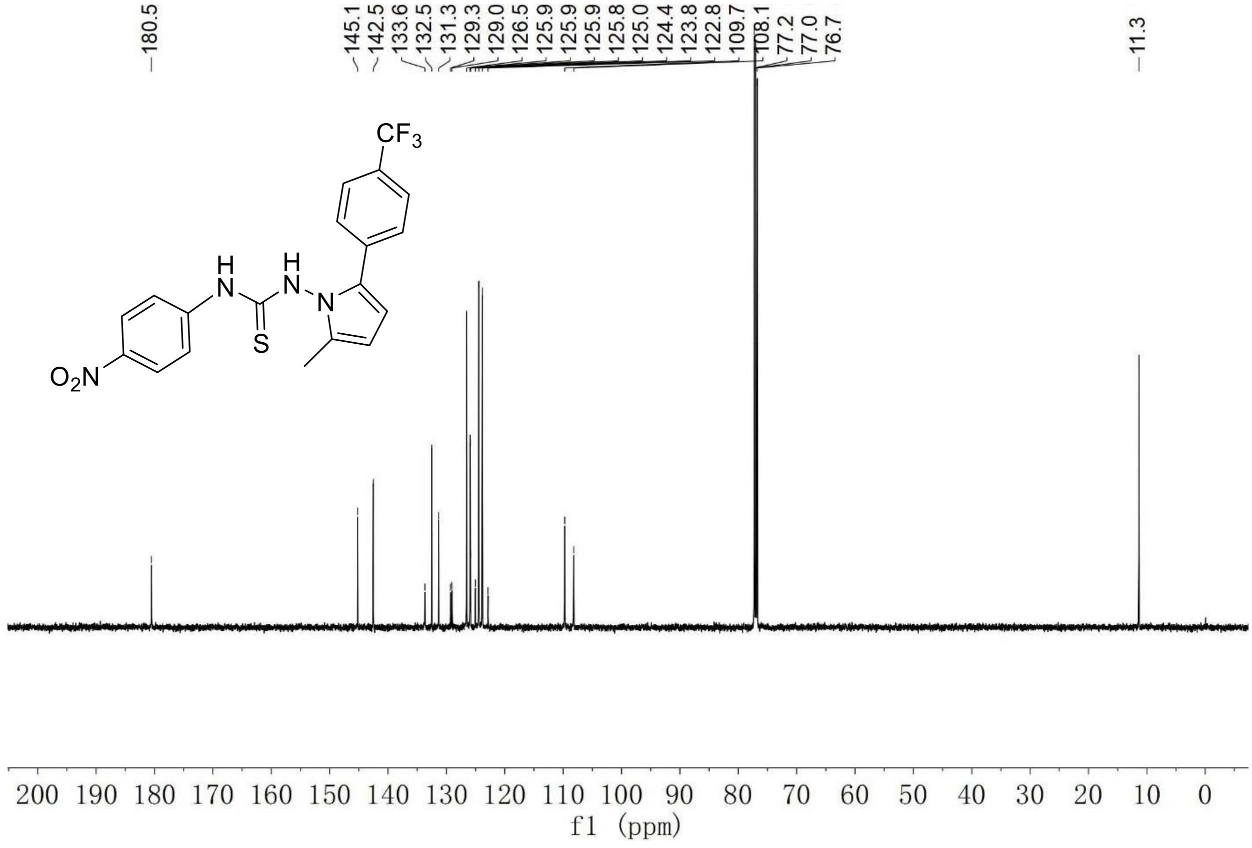

Supplementary Figure 42. ^{13}**C NMR spectrum of compound 5i (CDCl$_3$, 126 MHz, 298 K)**

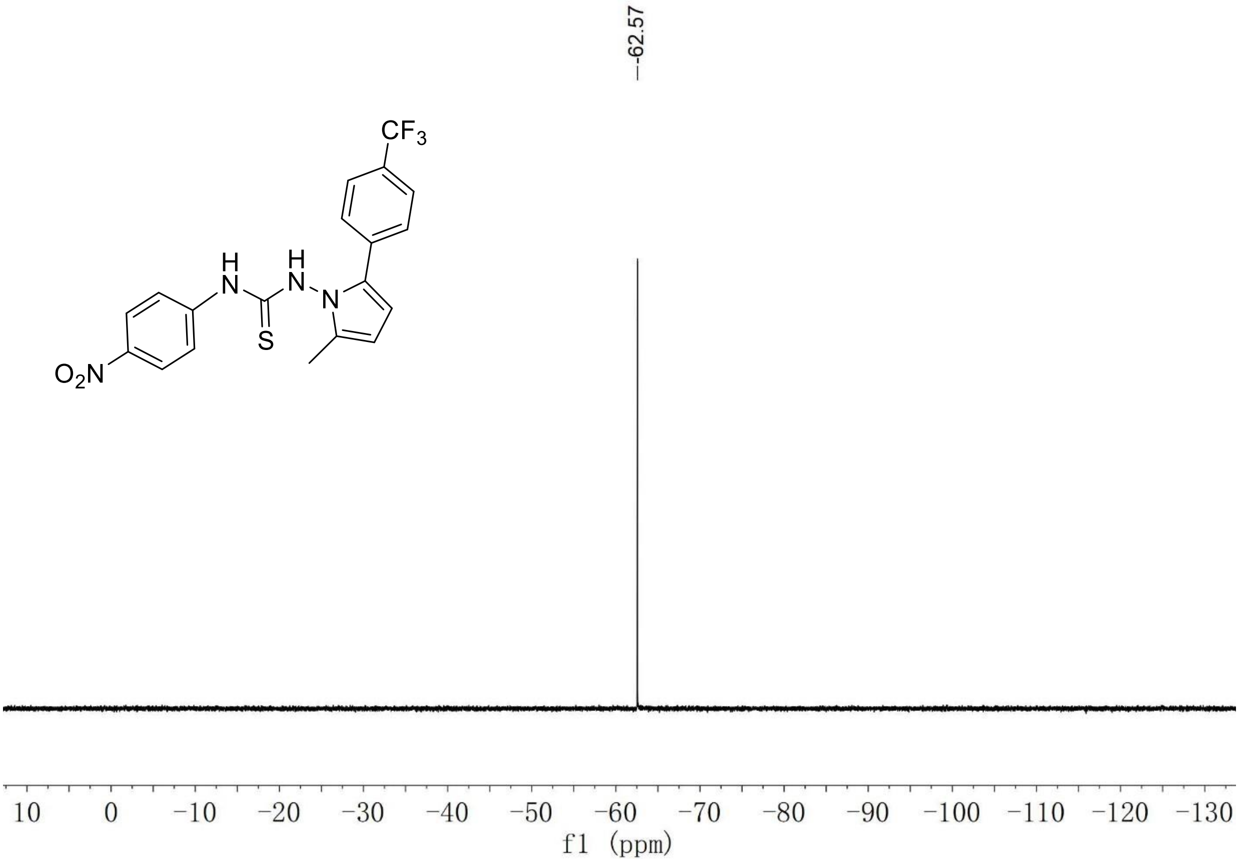

Supplementary Figure 43. ^{19}F NMR spectrum of compound 5i ($CDCl_3$, 471 MHz, 298 K)

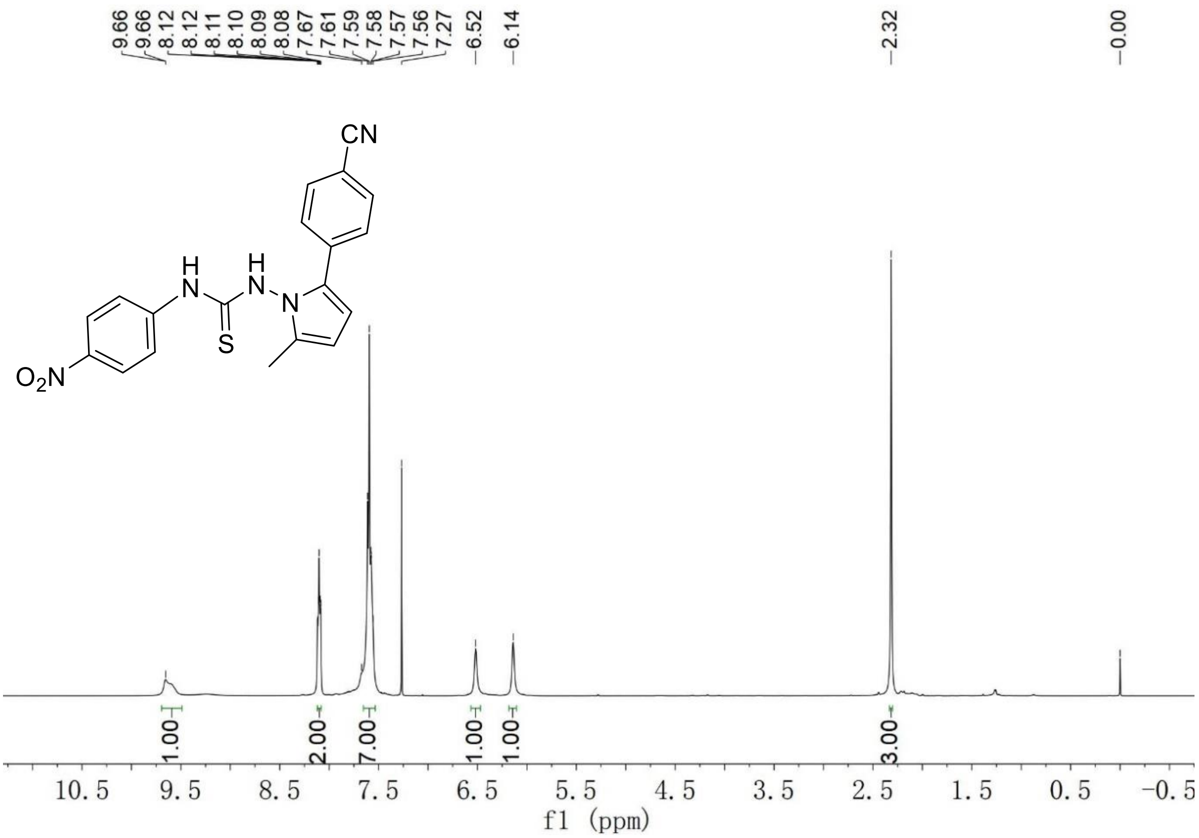

Supplementary Figure 44. ^{1}H NMR spectrum of compound 5j ($CDCl_3$, 500 MHz, 298 K)

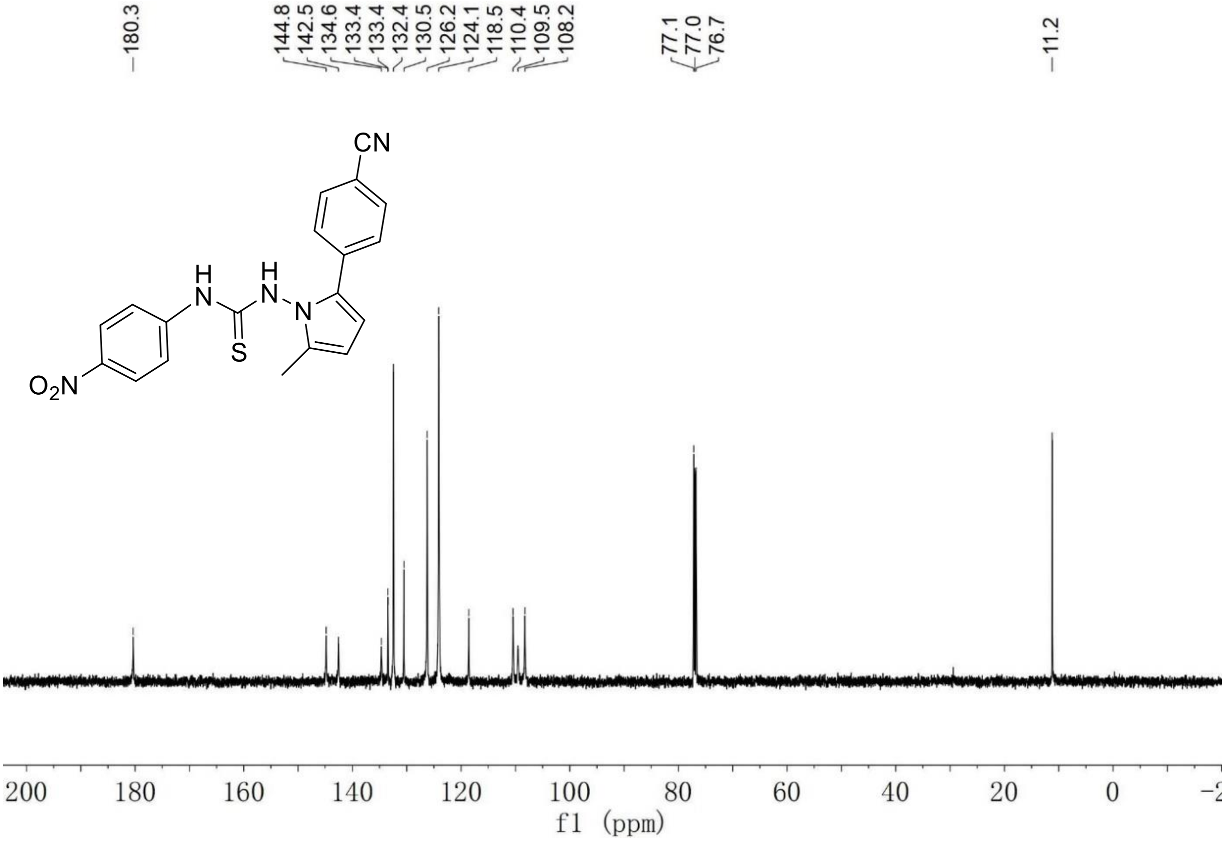

Supplementary Figure 45. ^{13}C NMR spectrum of compound 5j ($CDCl_3$, 126 MHz, 298 K)

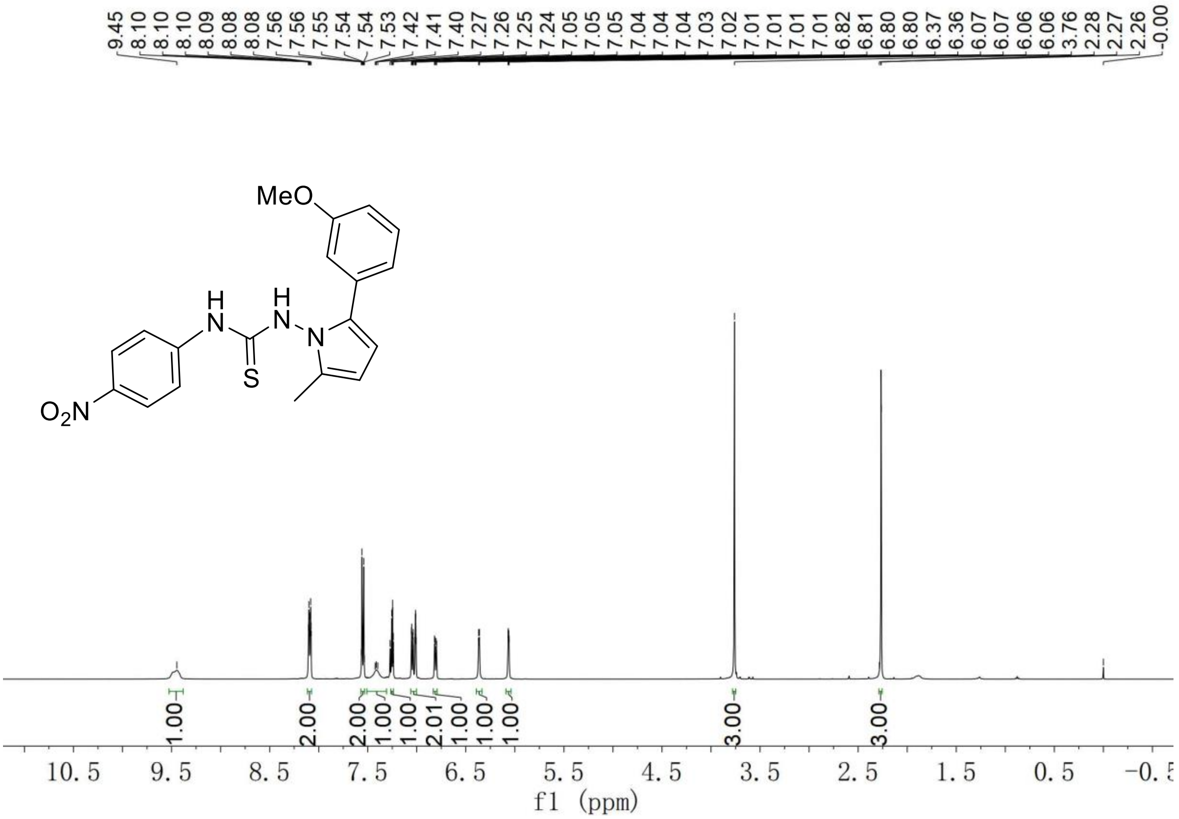

Supplementary Figure 46. ^{1}H NMR spectrum of compound 5k ($CDCl_3$, 500 MHz, 298 K)

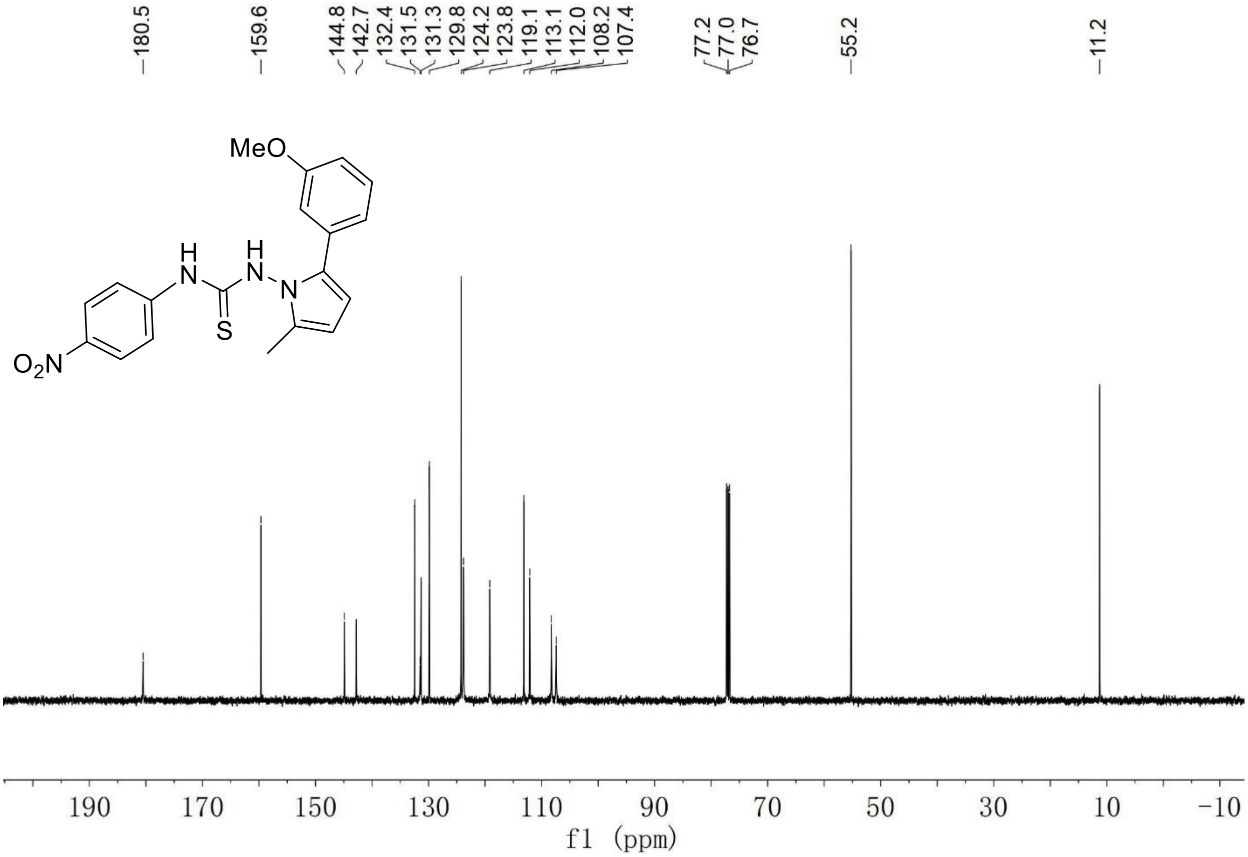

Supplementary Figure 47. ^{13}C NMR spectrum of compound 5k ($CDCl_3$, 126 MHz, 298 K)

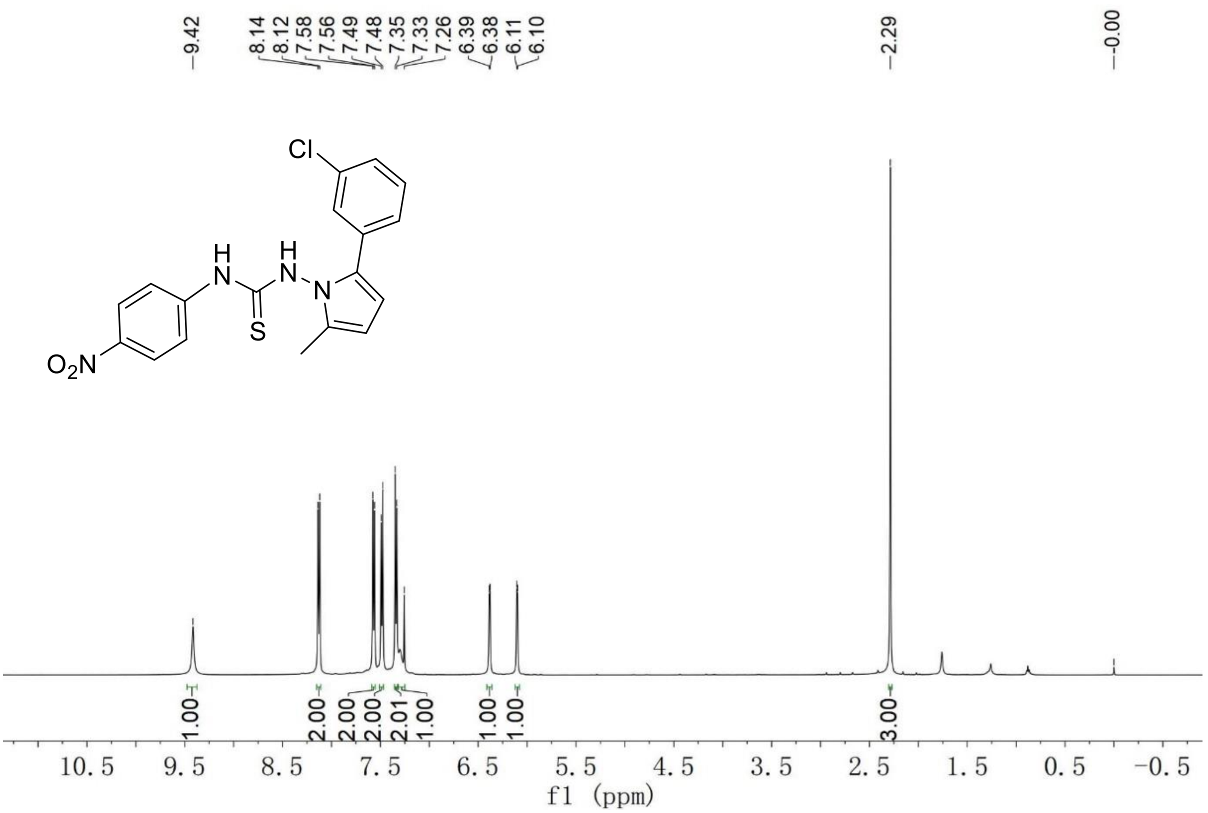

Supplementary Figure 48. ^{1}H NMR spectrum of compound 5l ($CDCl_3$, 500 MHz, 298 K)

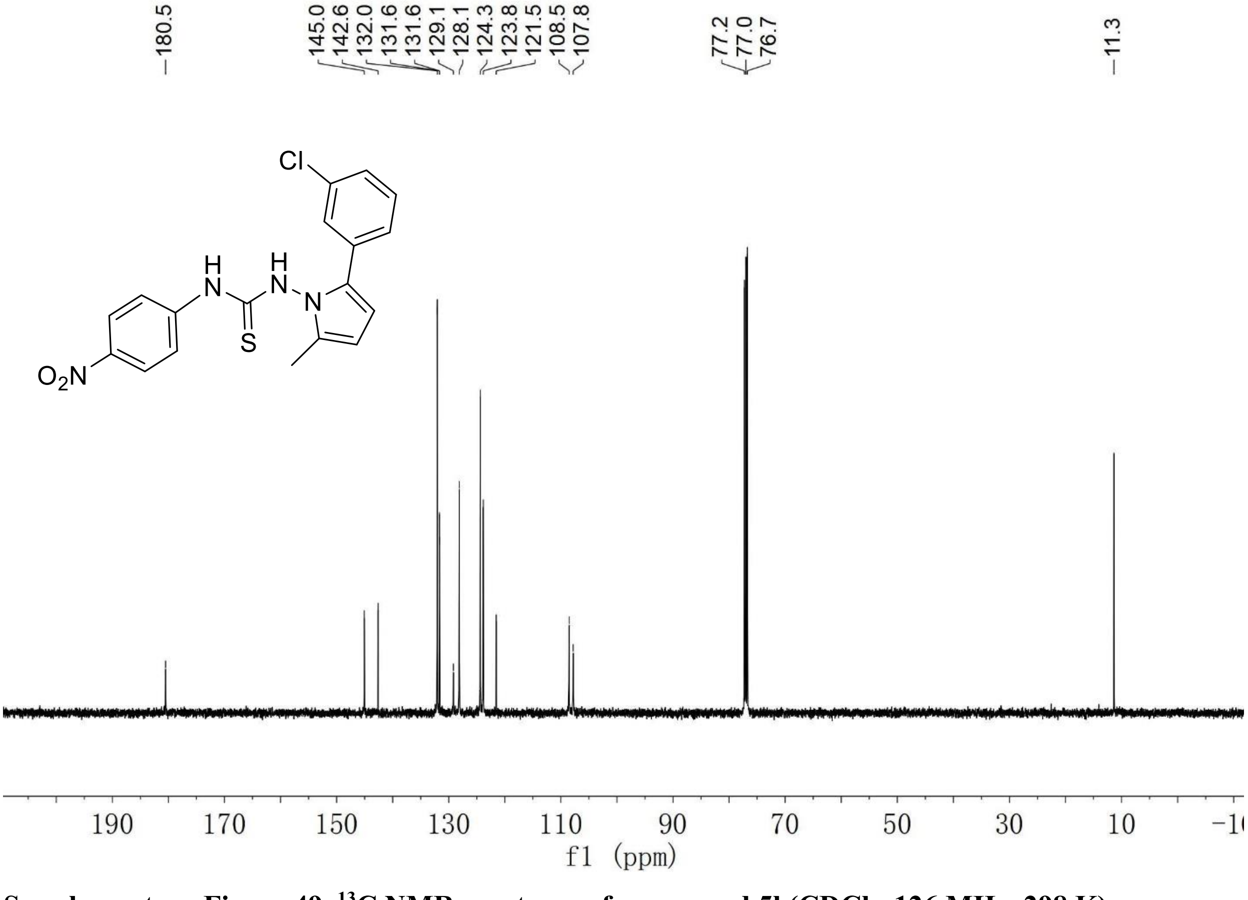

Supplementary Figure 49. ^{13}C NMR spectrum of compound 5l ($CDCl_3$, 126 MHz, 298 K)

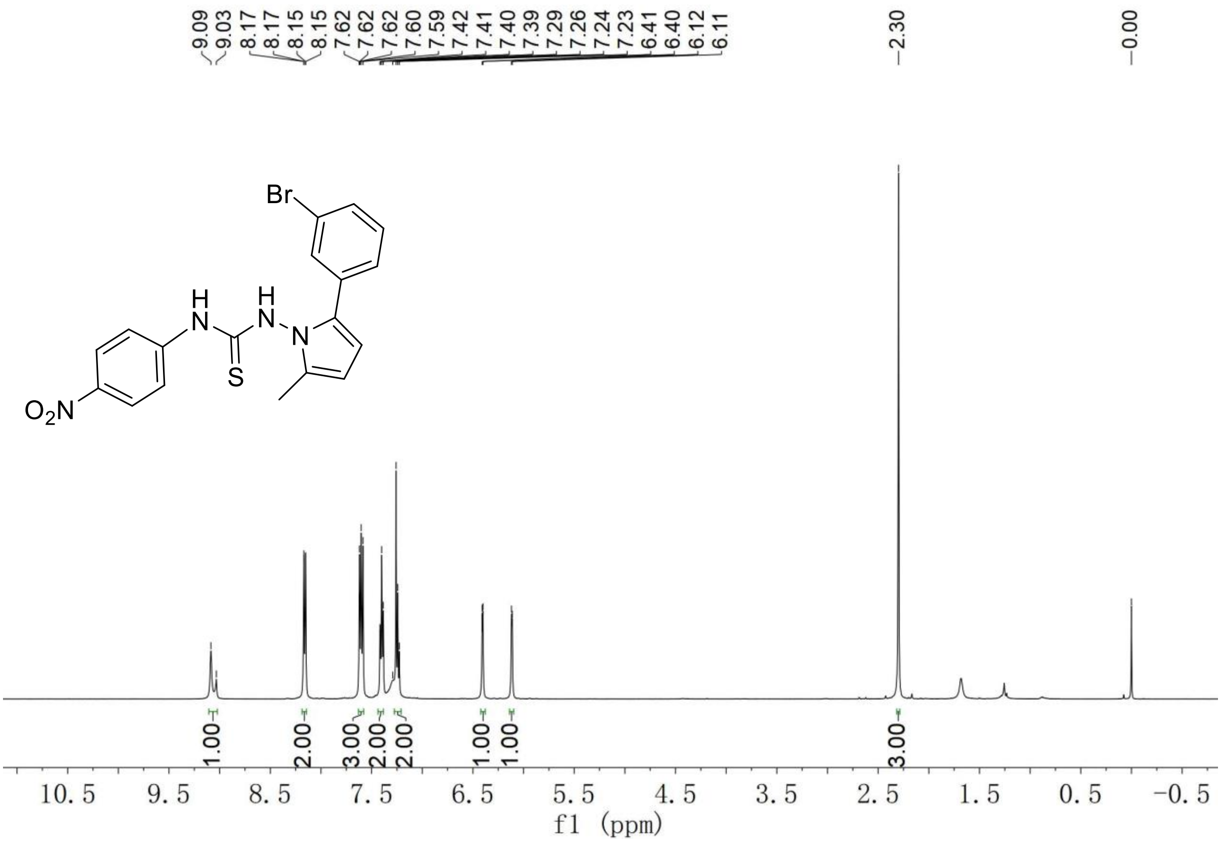

Supplementary Figure 50. ^{1}H NMR spectrum of compound 5m ($CDCl_3$, 500 MHz, 298 K)

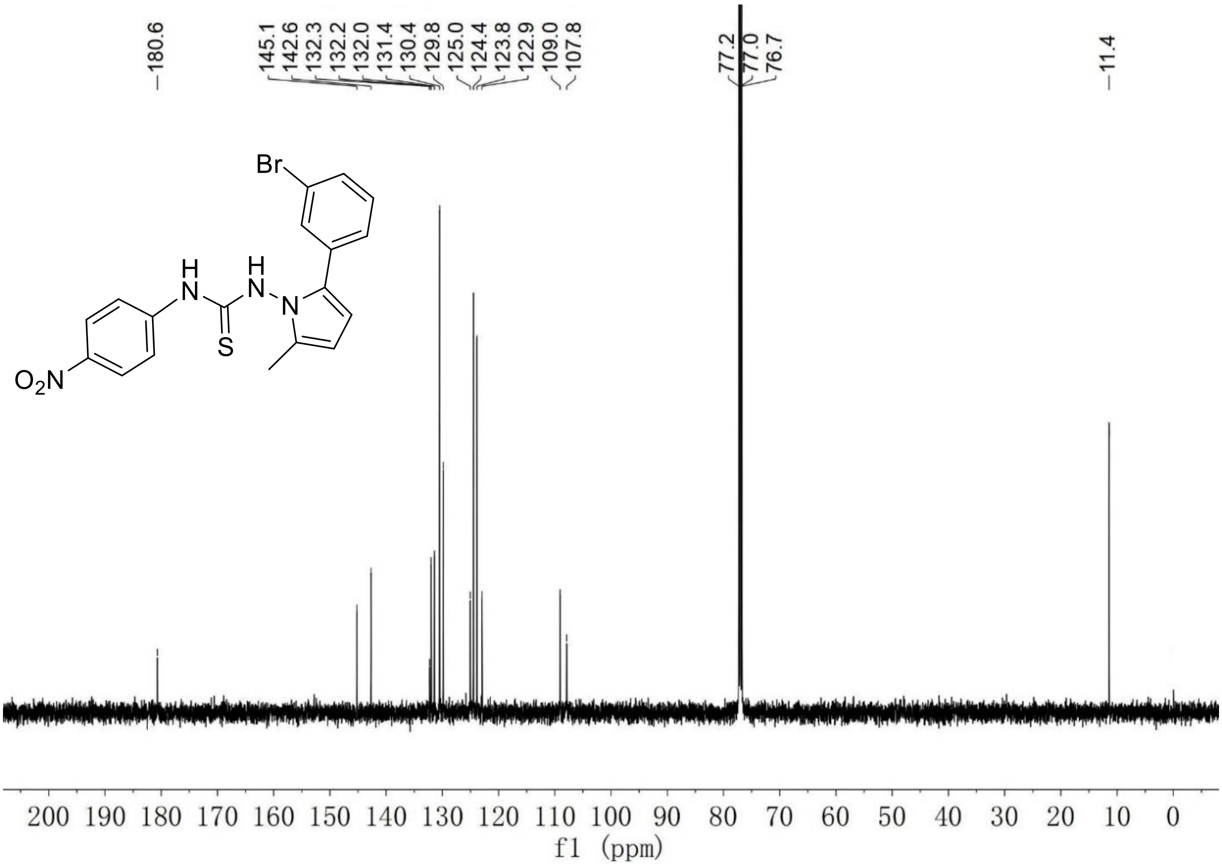

Supplementary Figure 51. ^{13}C NMR spectrum of compound 5m ($CDCl_3$, 126 MHz, 298 K)

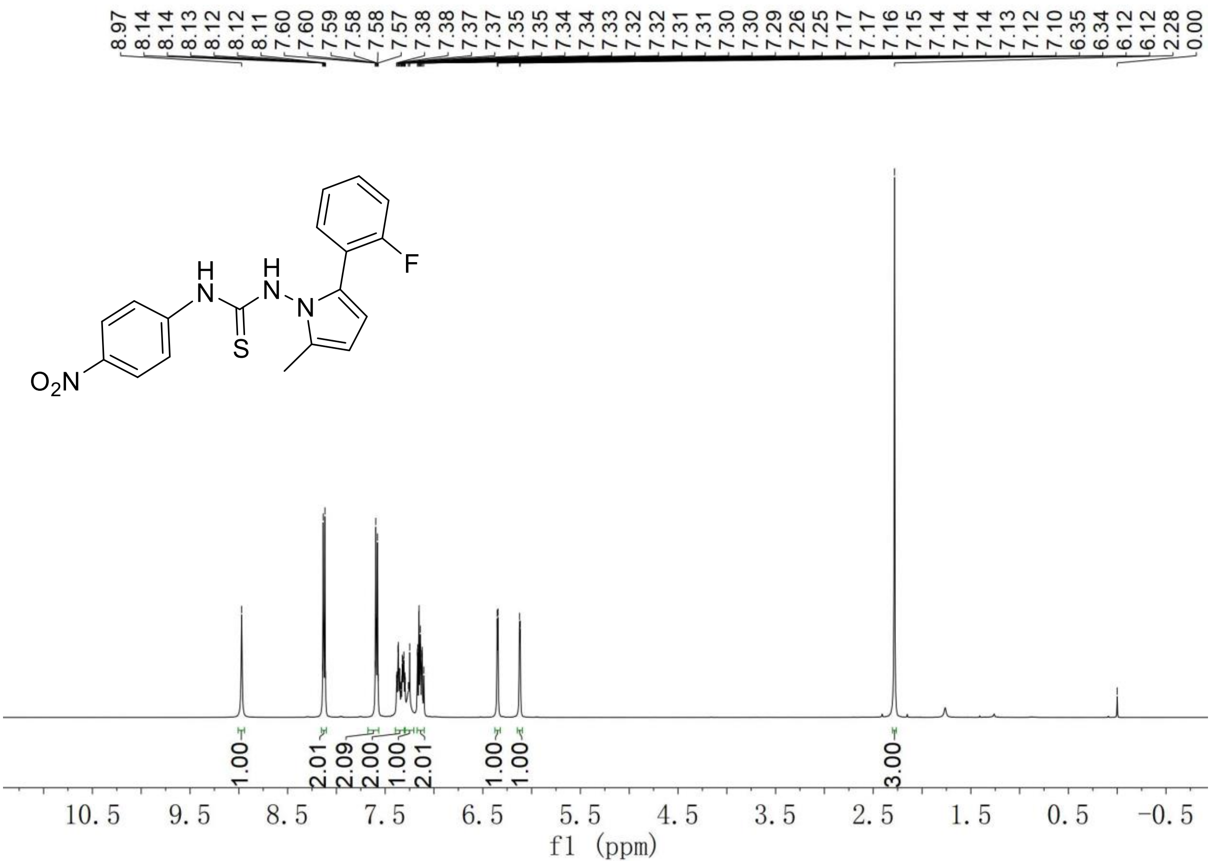

Supplementary Figure 52. ^{1}H NMR spectrum of compound 5n ($CDCl_3$, 500 MHz, 298 K)

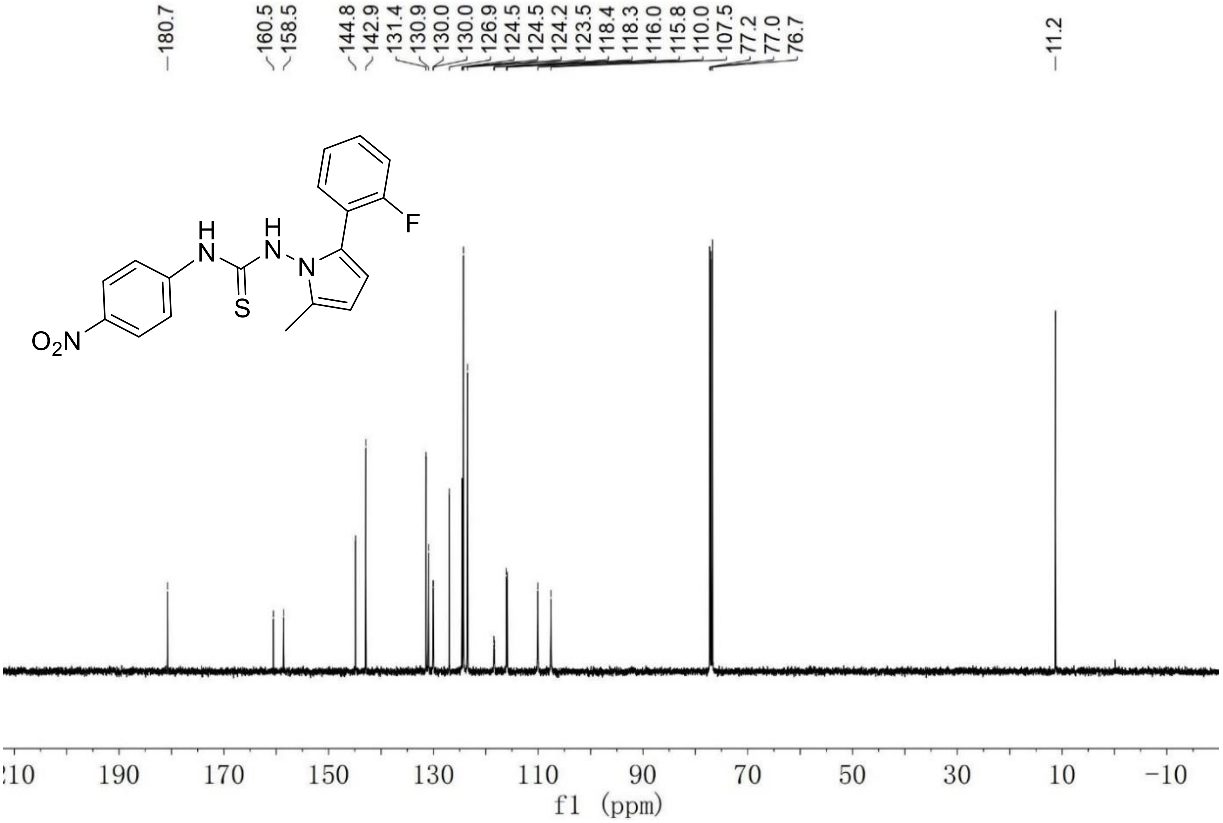

Supplementary Figure 53. ^{13}C NMR spectrum of compound 5n ($CDCl_3$, 126 MHz, 298 K)

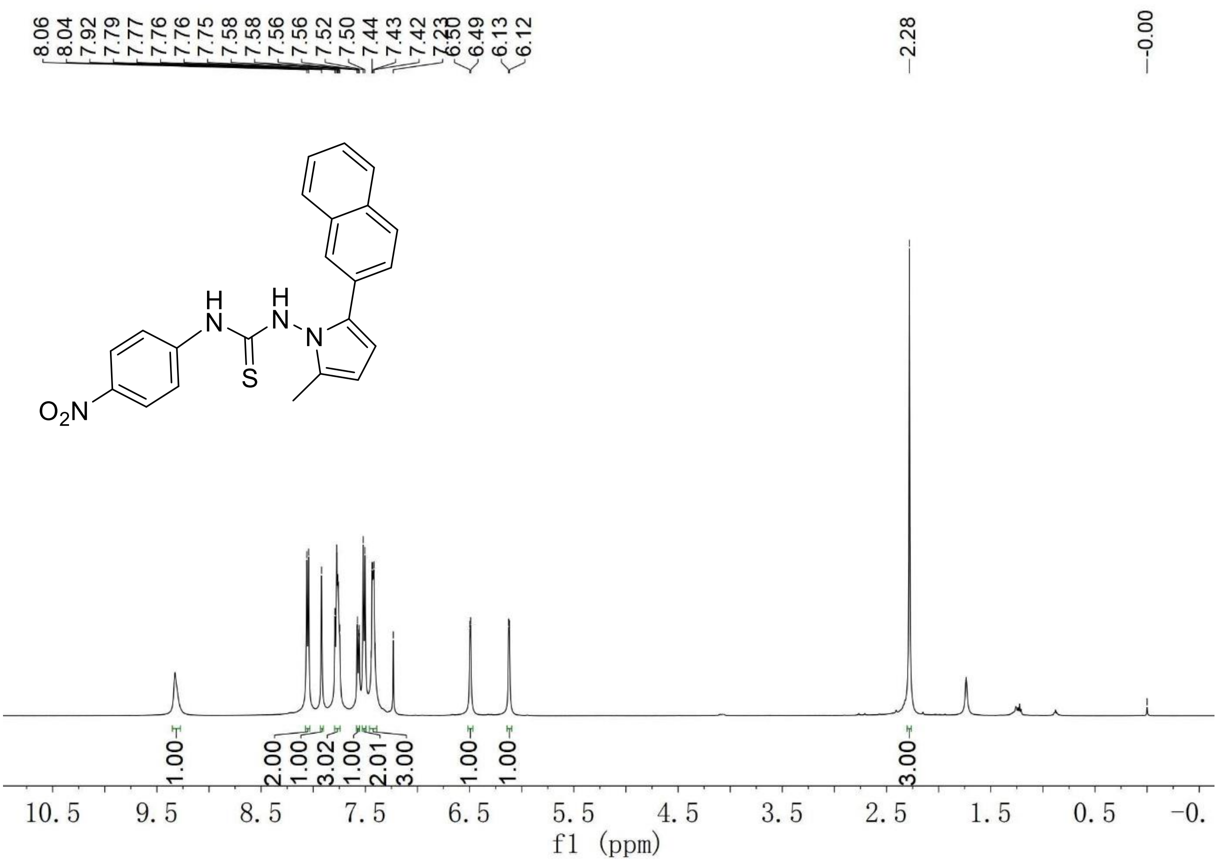

Supplementary Figure 54. ^{1}H NMR spectrum of compound 5o ($CDCl_3$, 500 MHz, 298 K)

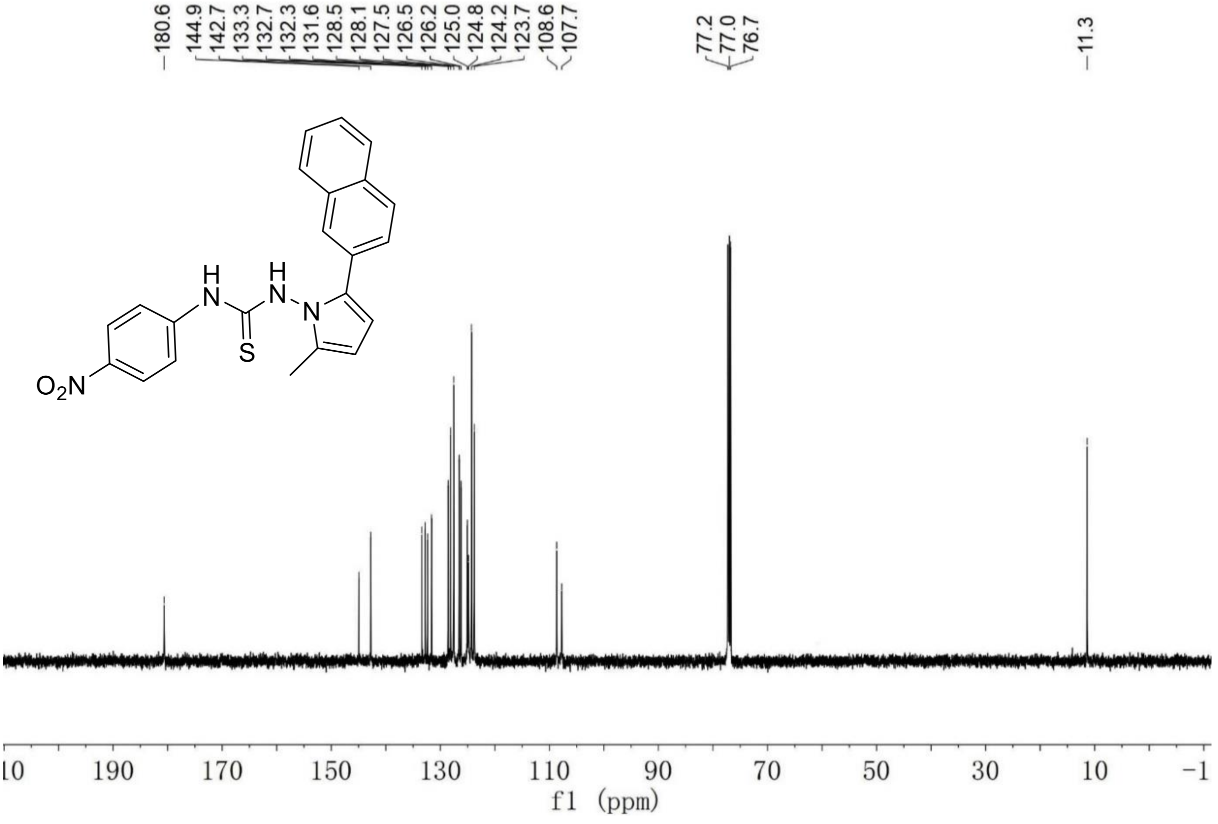

Supplementary Figure 55. ^{13}C NMR spectrum of compound 5o ($CDCl_3$, 126 MHz, 298 K)

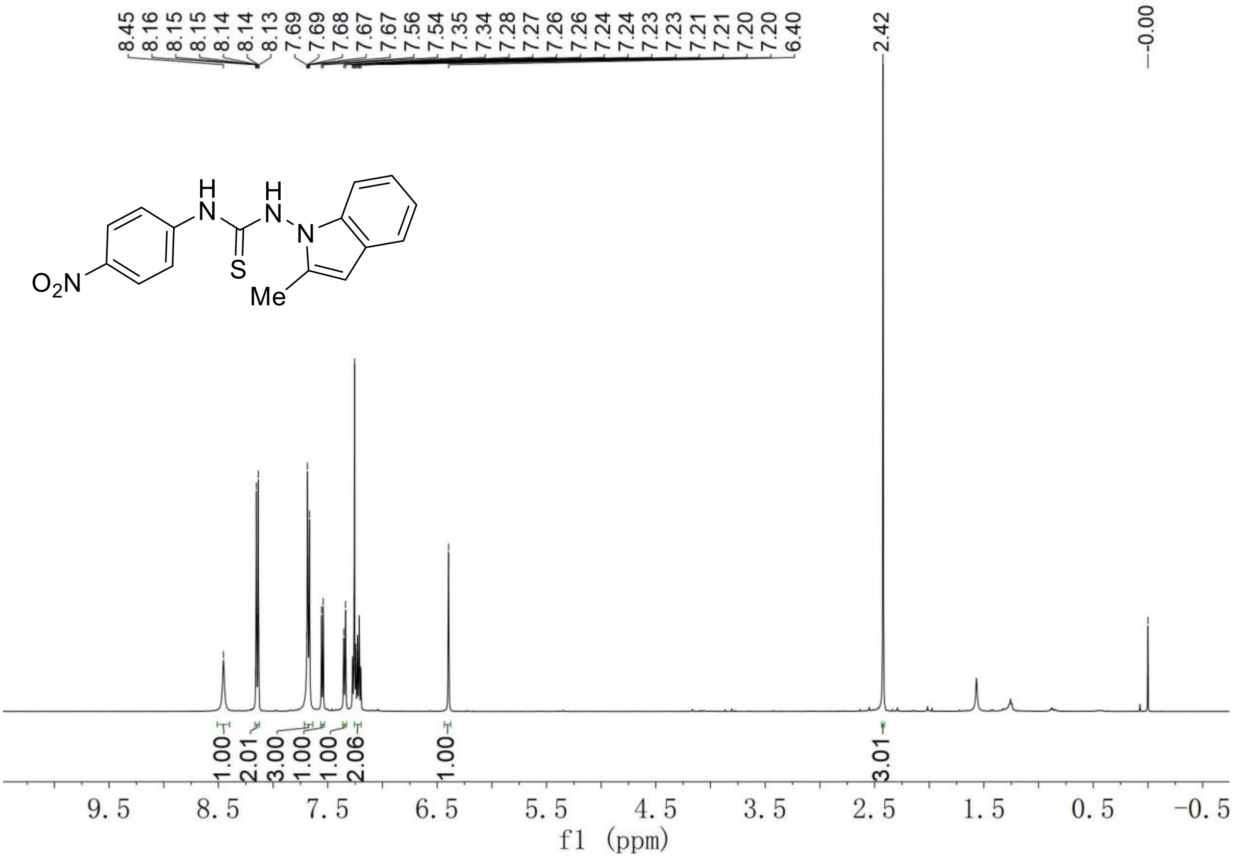

Supplementary Figure 56. ^{1}H NMR spectrum of compound 5ae ($CDCl_3$, 500 MHz, 298 K)

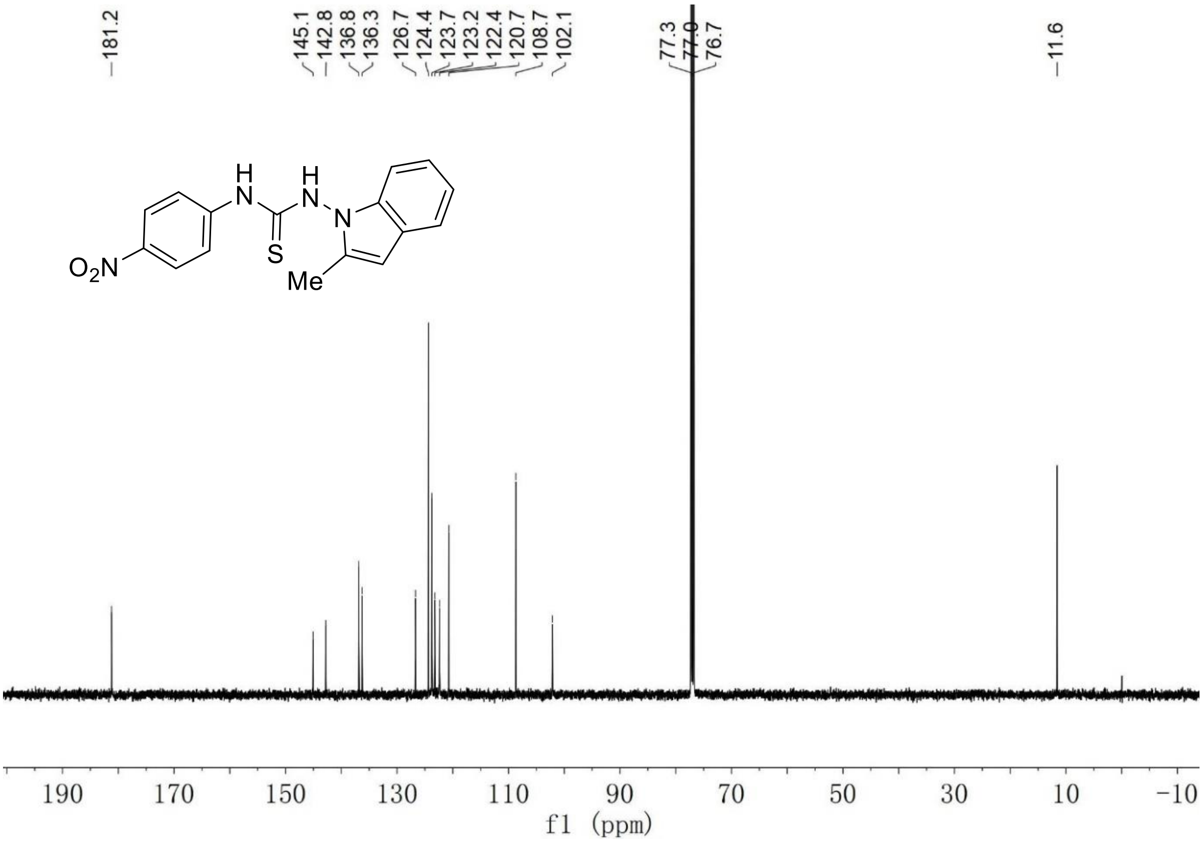

Supplementary Figure 57. ^{13}C NMR spectrum of compound 5ae ($CDCl_3$, 126 MHz, 298 K)

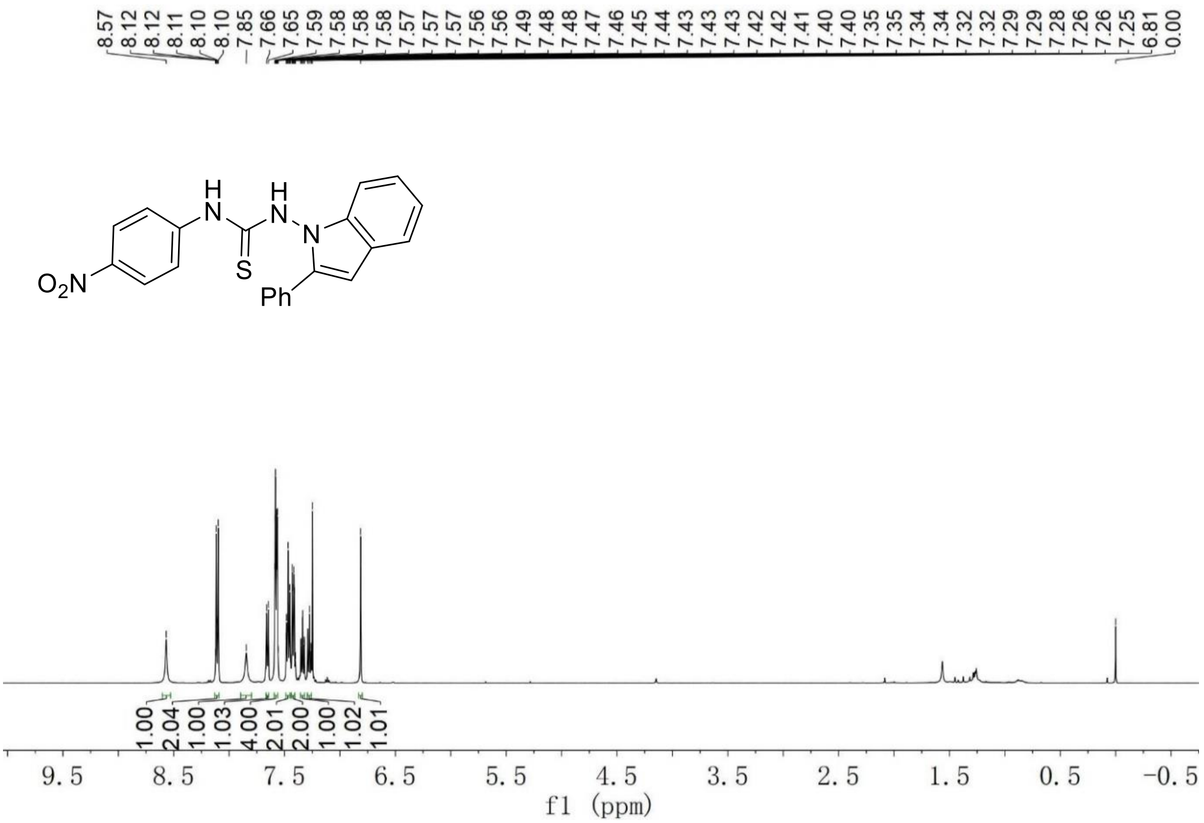

Supplementary Figure 58. ^{1}H NMR spectrum of compound 5af ($CDCl_3$, 500 MHz, 298 K)

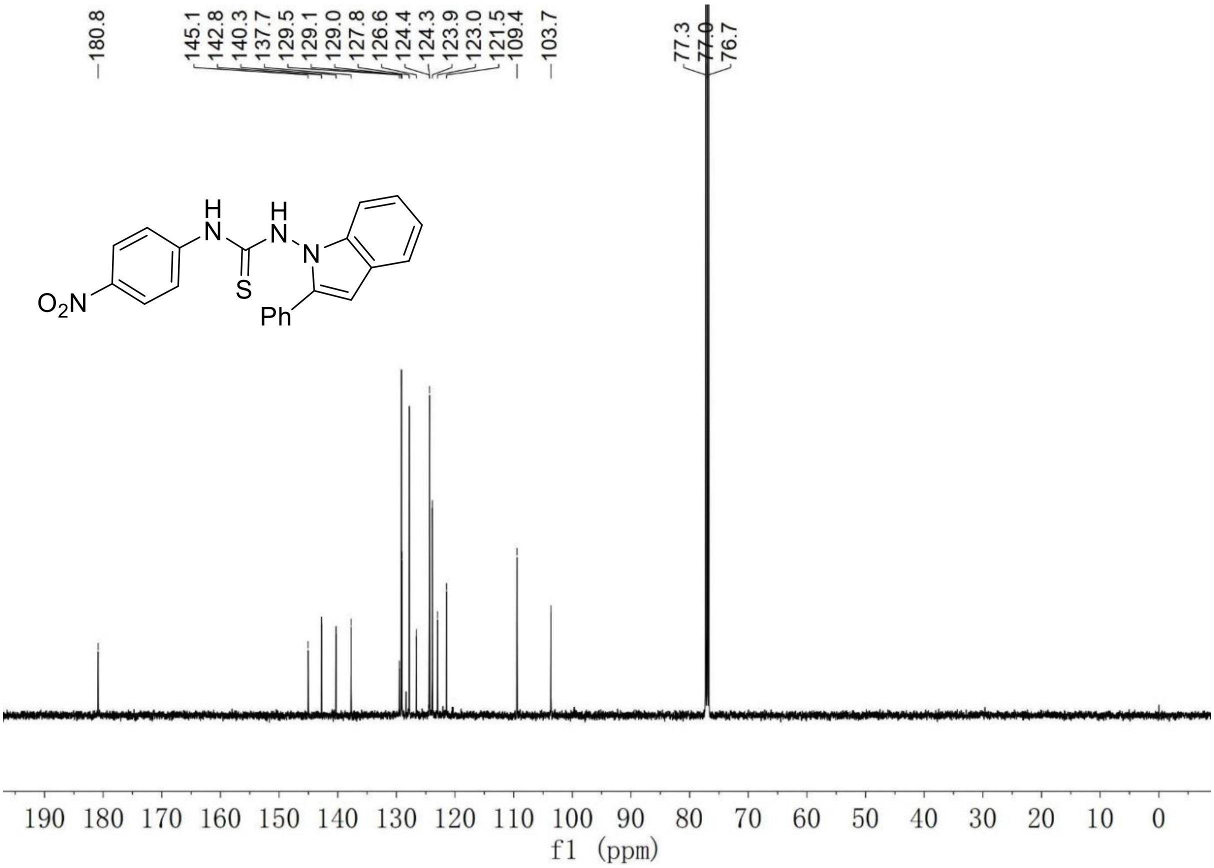

Supplementary Figure 59. ^{13}C NMR spectrum of compound 5af ($CDCl_3$, 126 MHz, 298 K)

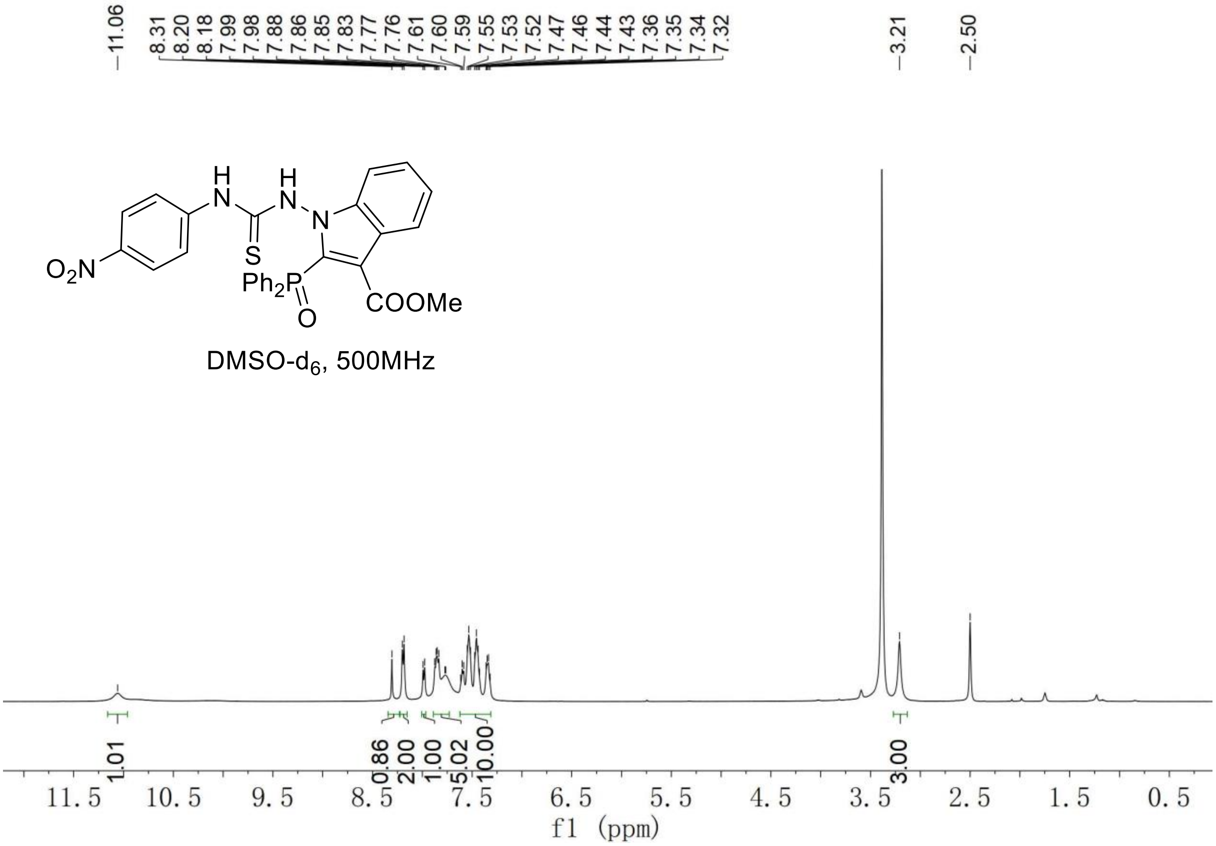

Supplementary Figure 60. ^{1}H NMR spectrum of compound 5ag (DMSO-d_6, 500 MHz, 298 K)

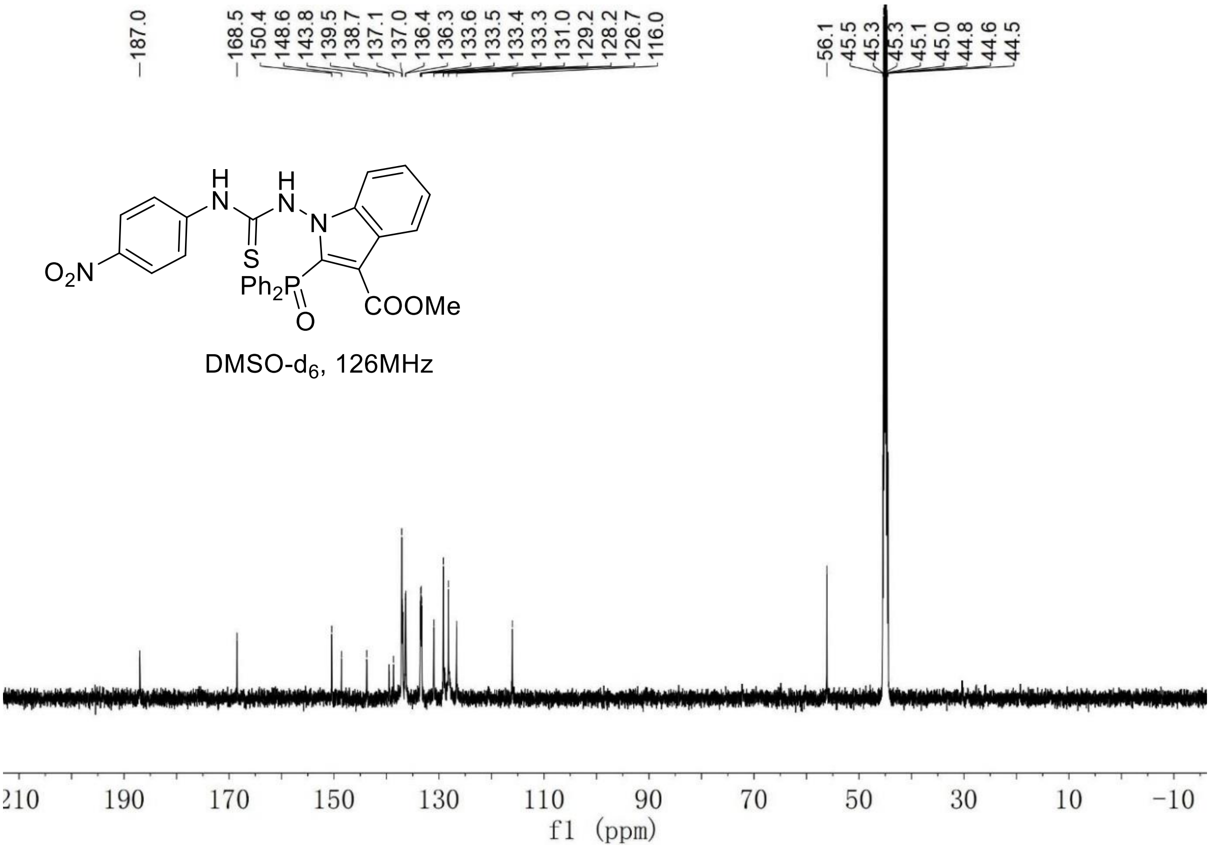

Supplementary Figure 61. ^{13}C NMR spectrum of compound 5ag (DMSO-d_6, 126 MHz, 298 K)

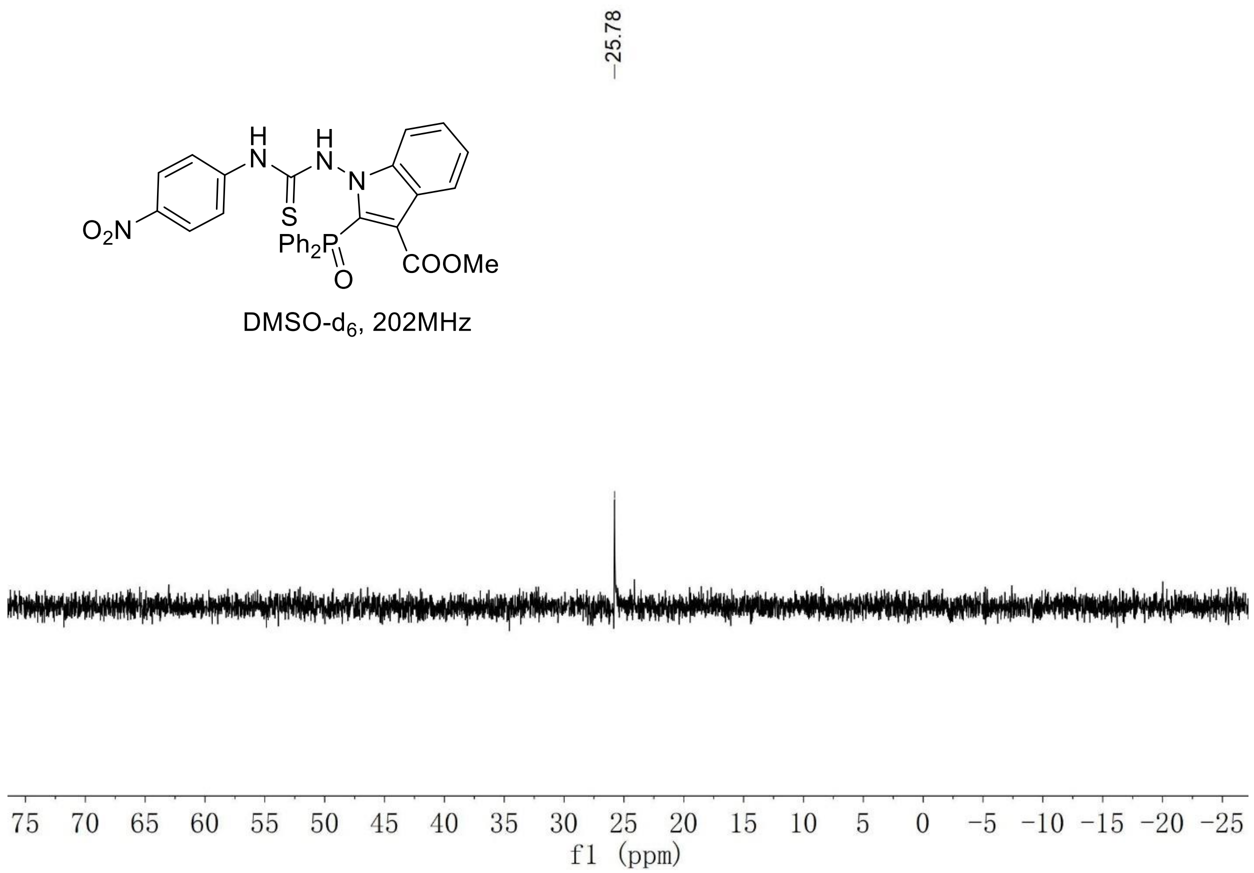

Supplementary Figure 62. ^{31}P NMR spectrum of compound 5ag (DMSO-*d*$_6$, 202 MHz, 298 K)

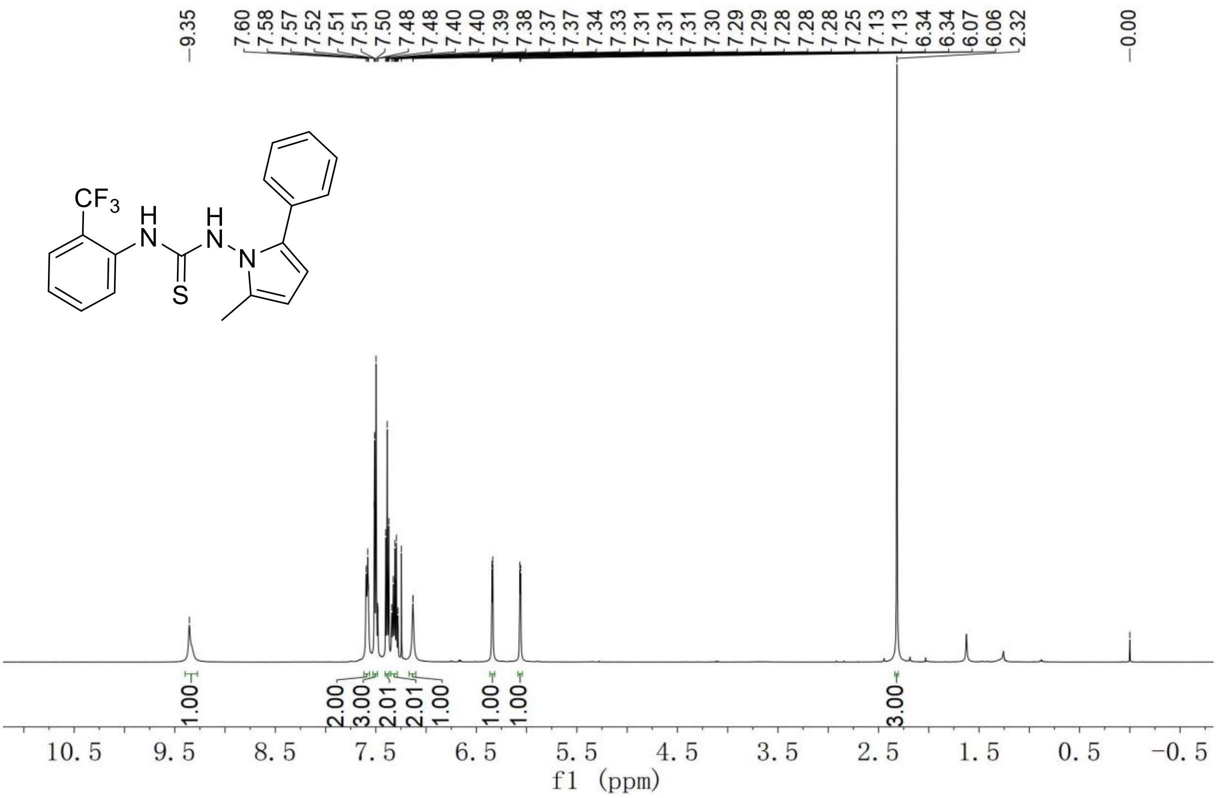

Supplementary Figure 63. ^{1}H NMR spectrum of compound 5ah ($CDCl_3$, 500 MHz, 298 K)

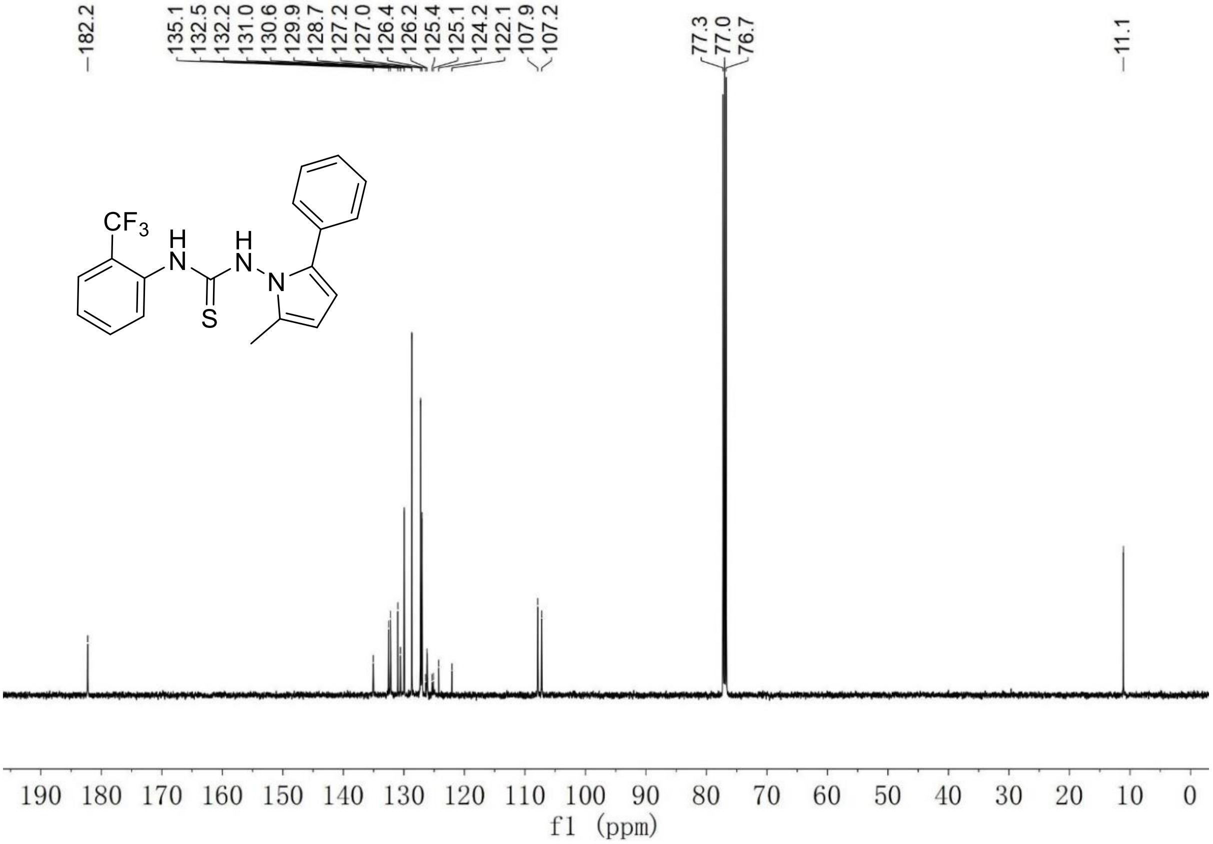

Supplementary Figure 64. ^{13}C NMR spectrum of compound 5ah ($CDCl_3$, 126 MHz, 298 K)

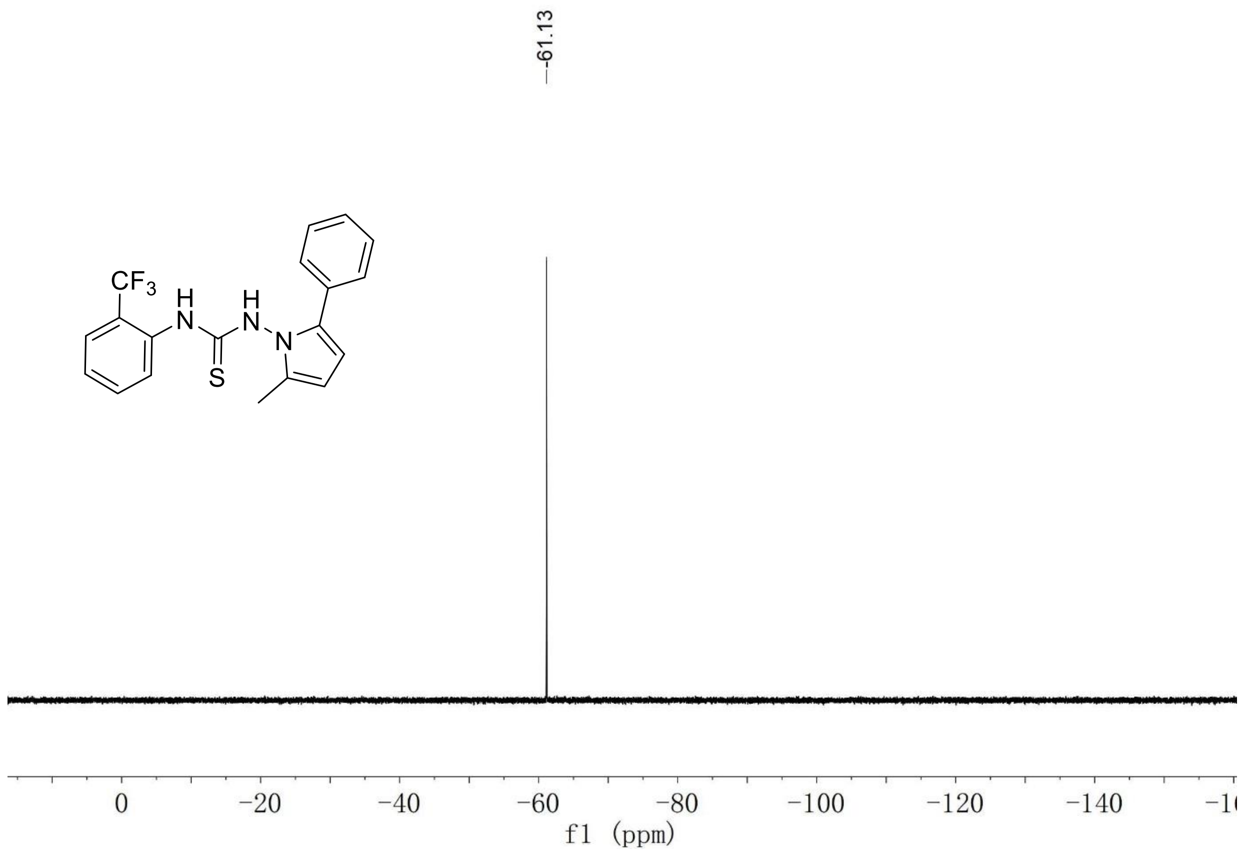

Supplementary Figure 65. ^{19}F NMR spectrum of compound 5ah ($CDCl_3$, 471 MHz, 298 K)

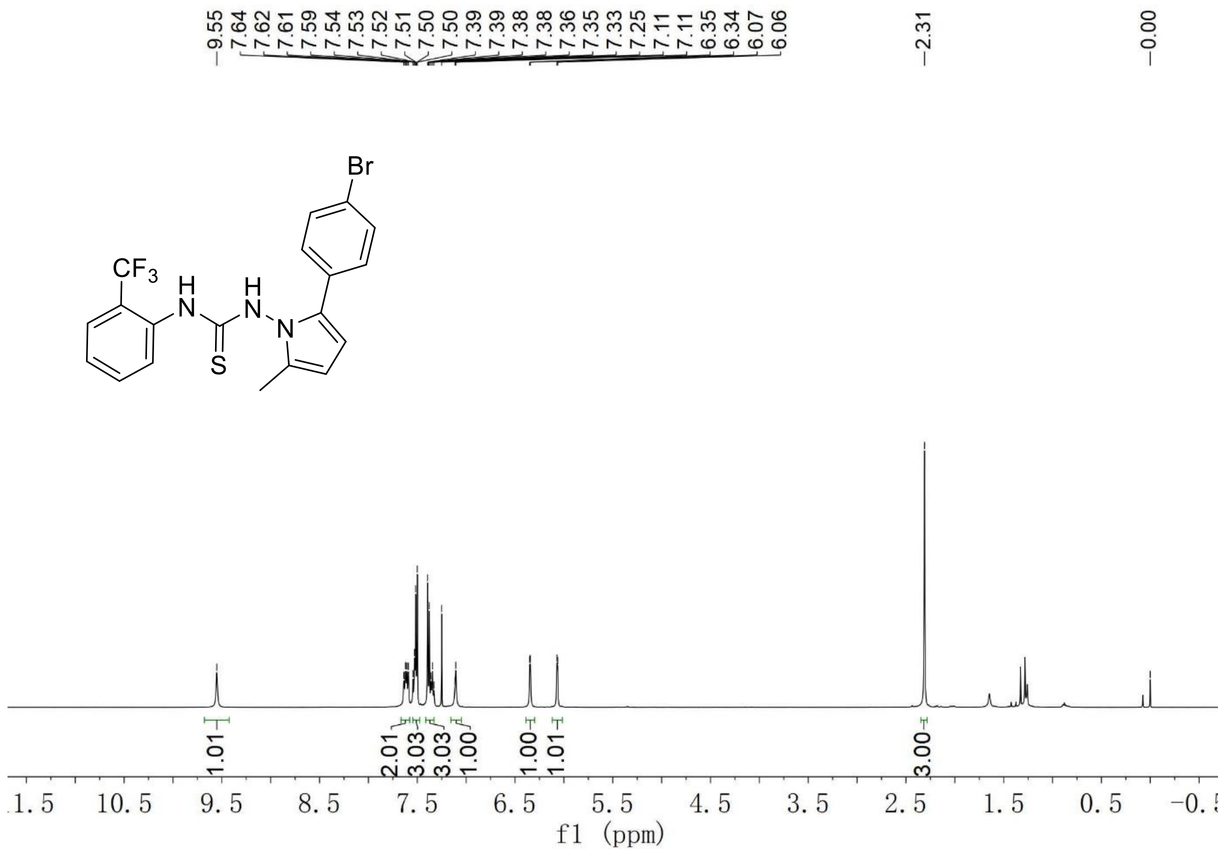

Supplementary Figure 66. ^{1}H NMR spectrum of compound 5ai ($CDCl_3$, 500 MHz, 298 K)

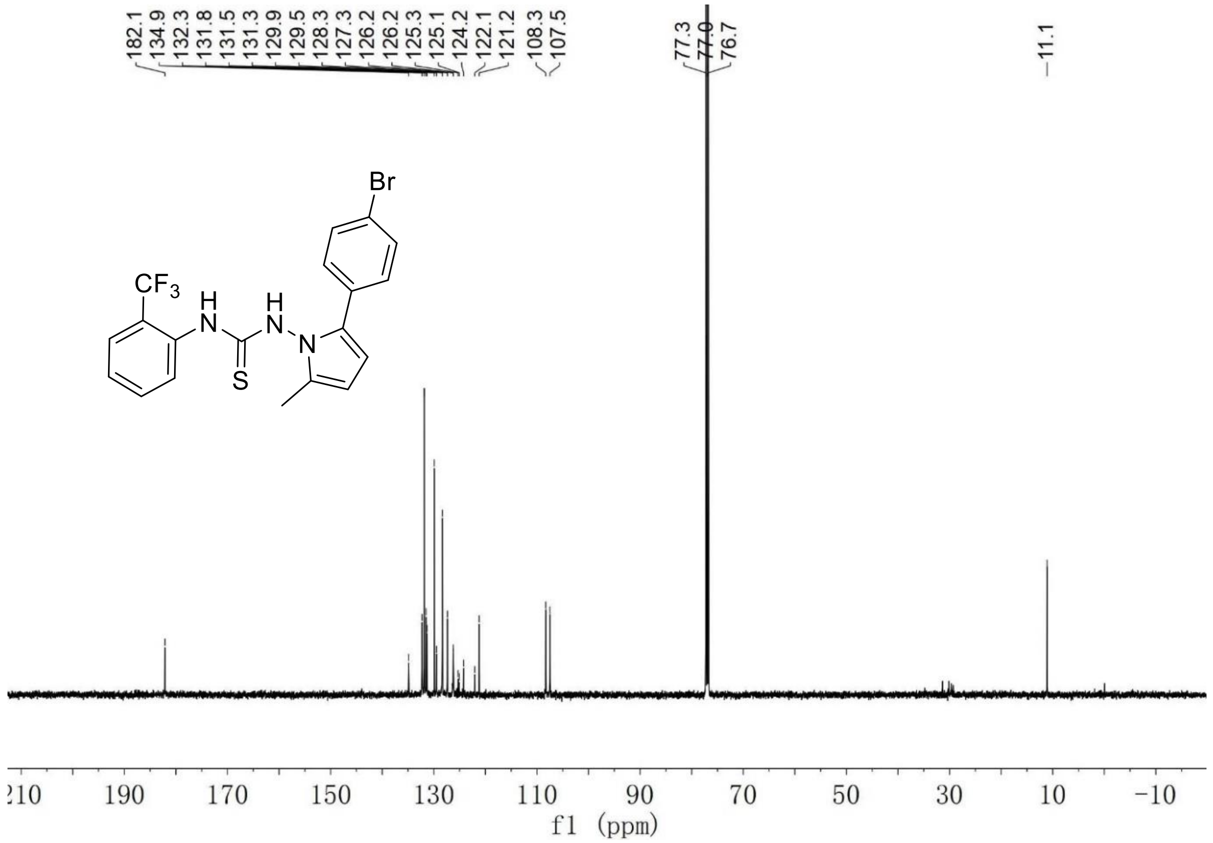

Supplementary Figure 67. ^{13}C NMR spectrum of compound 5ai ($CDCl_3$, 126 MHz, 298 K)

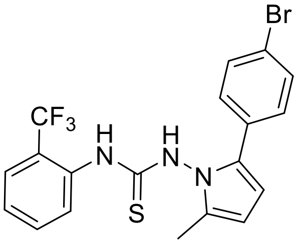

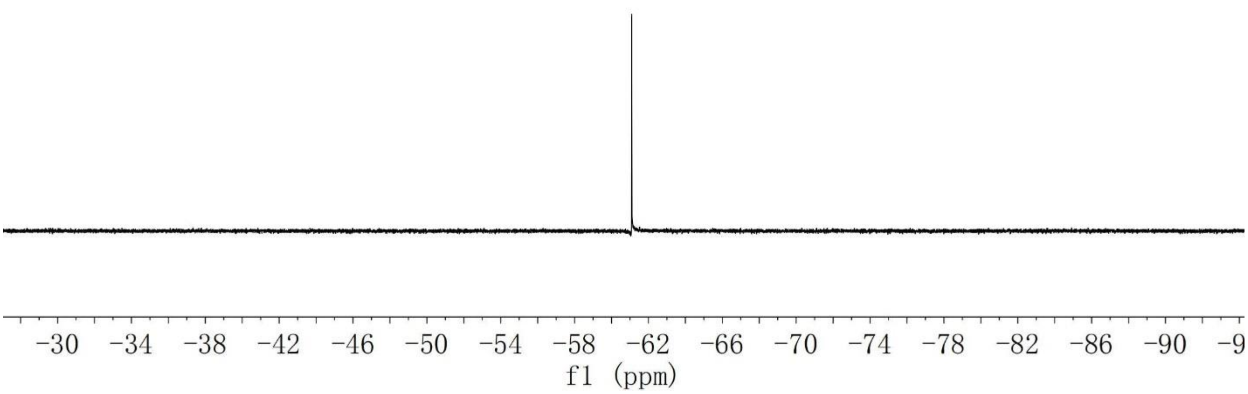

Supplementary Figure 68. ^{19}F NMR spectrum of compound 5ai ($CDCl_3$, 471 MHz, 298 K)

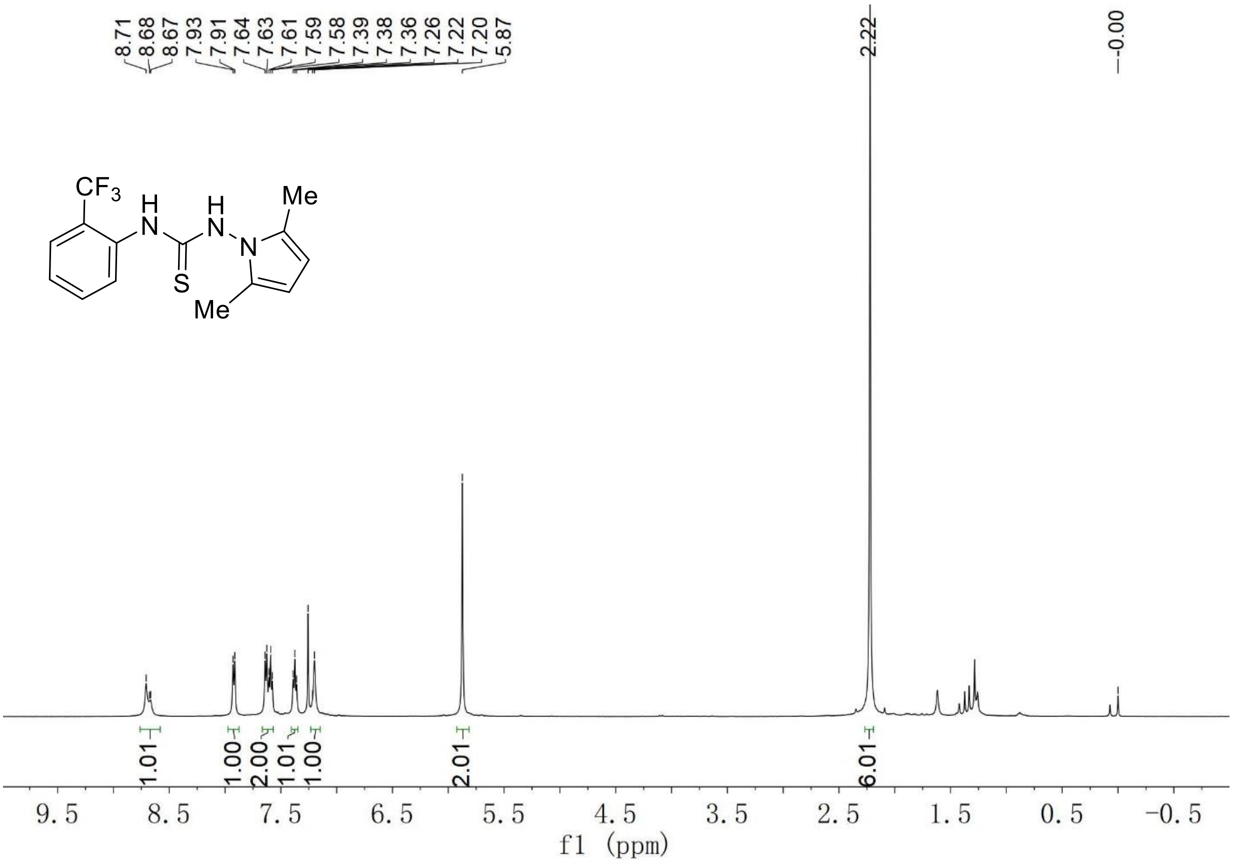

Supplementary Figure 69. ^{1}H NMR spectrum of compound 5aj ($CDCl_3$, 500 MHz, 298 K)

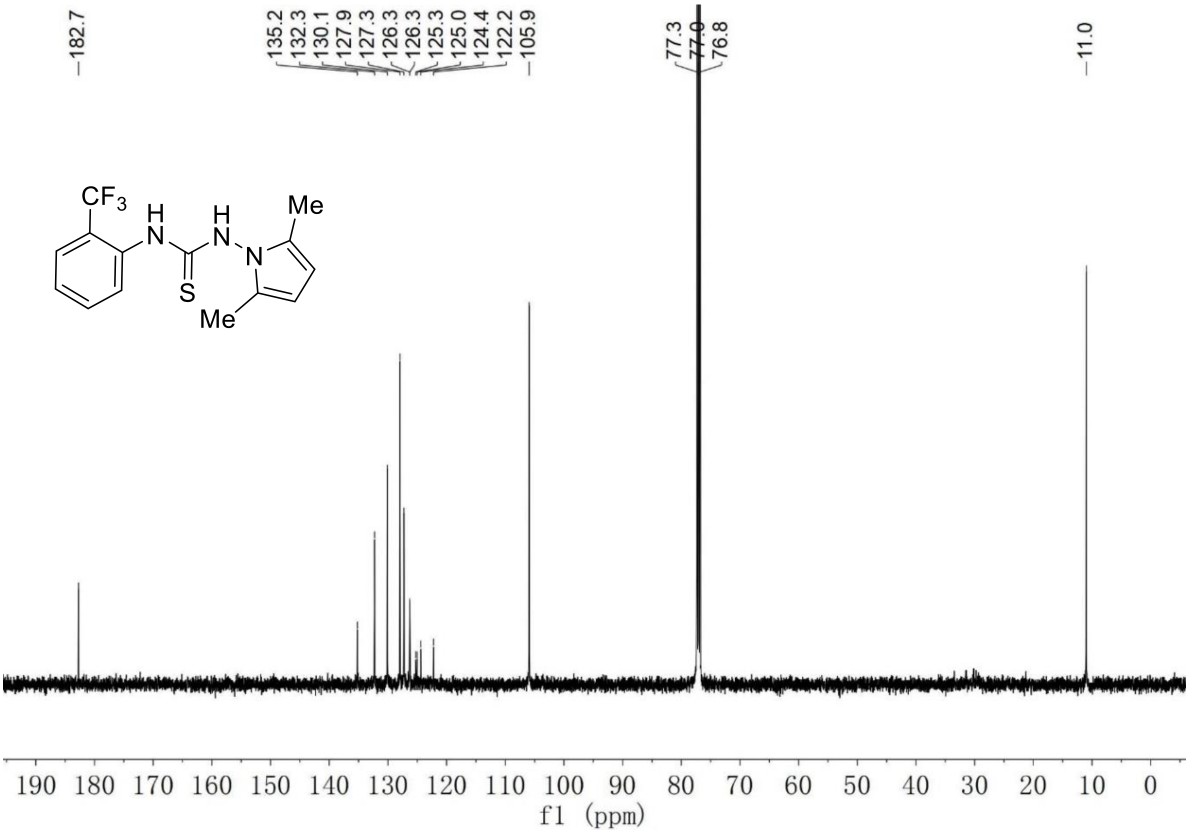

Supplementary Figure 70. ^{13}C NMR spectrum of compound 5aj ($CDCl_3$, 126 MHz, 298 K)

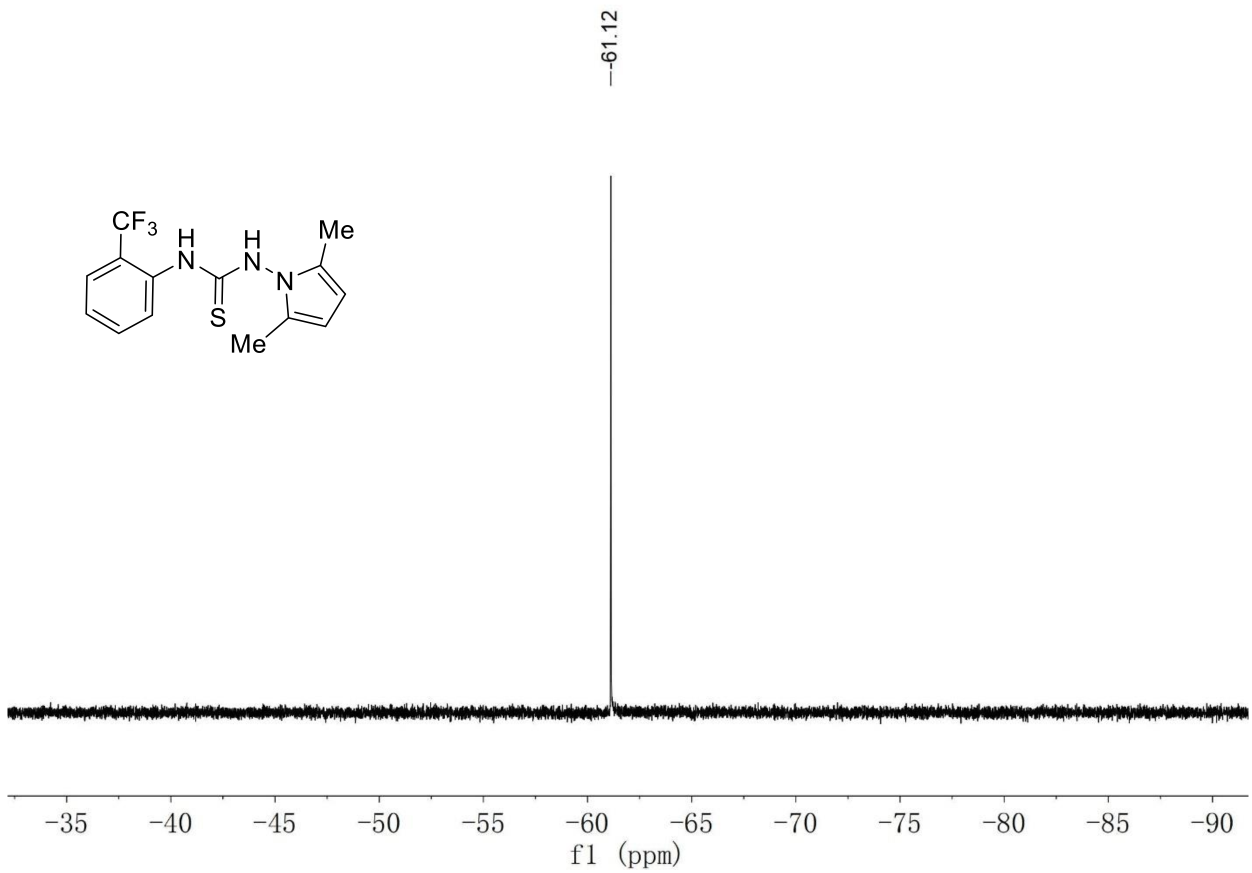

Supplementary Figure 71. ^{19}F NMR spectrum of compound 5aj ($CDCl_3$, 471 MHz, 298 K)

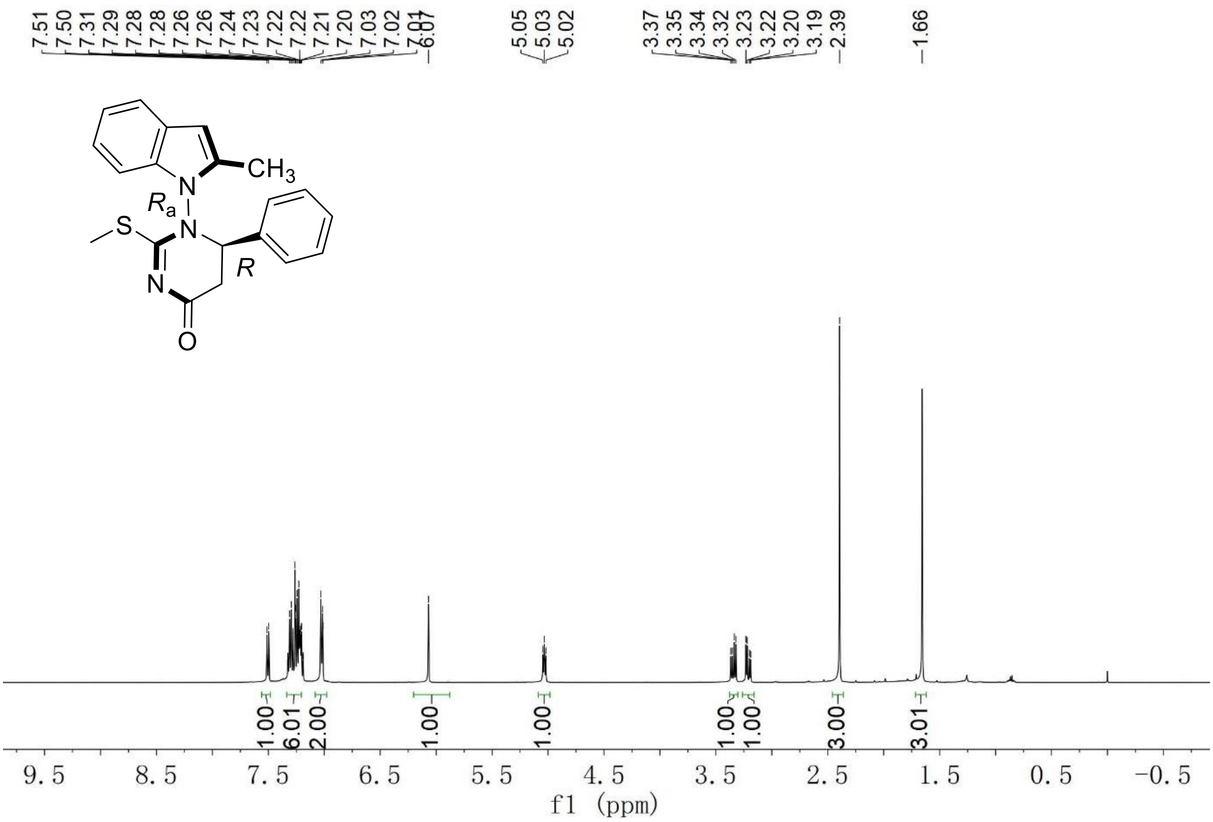

Supplementary Figure 72. ^{1}H NMR spectrum of compound 3a ($CDCl_3$, 500 MHz, 298 K)

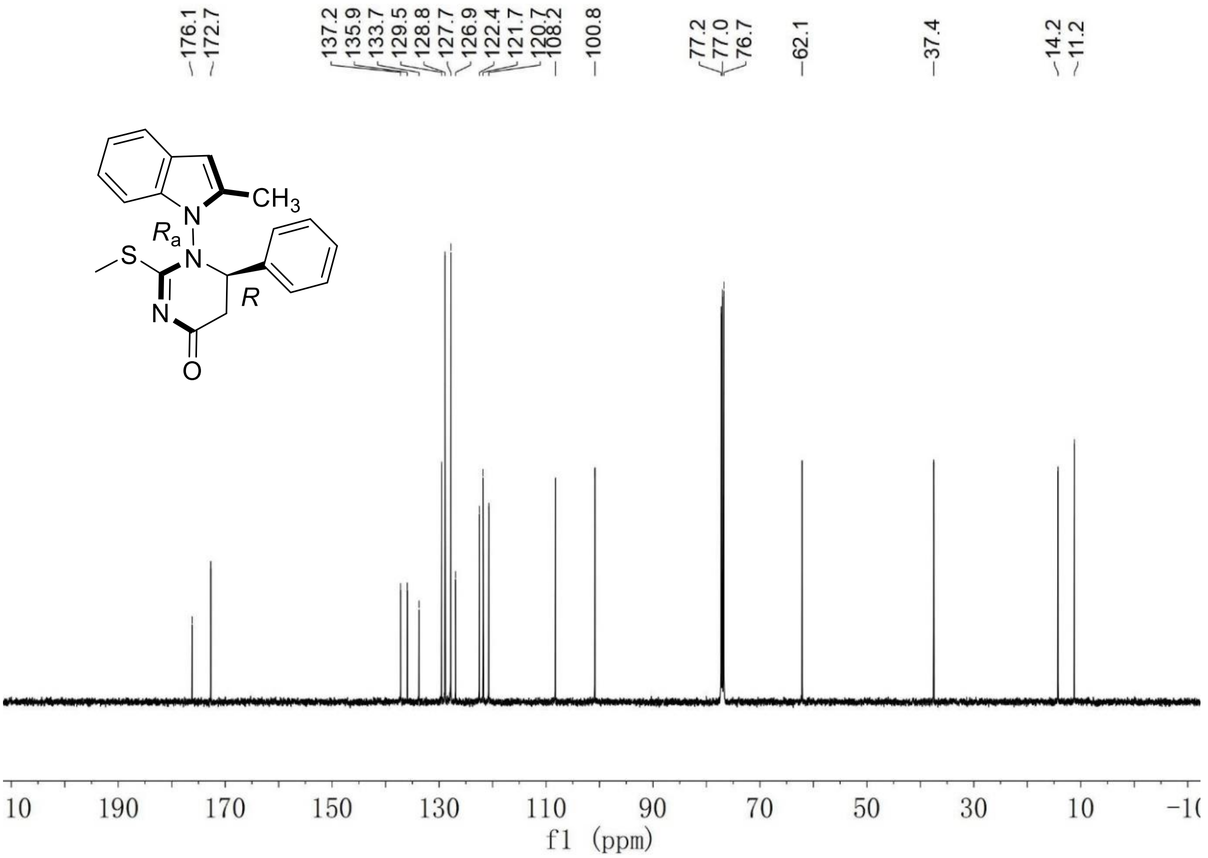

Supplementary Figure 73. ^{13}C NMR spectrum of compound 3a ($CDCl_3$, 126 MHz, 298 K)

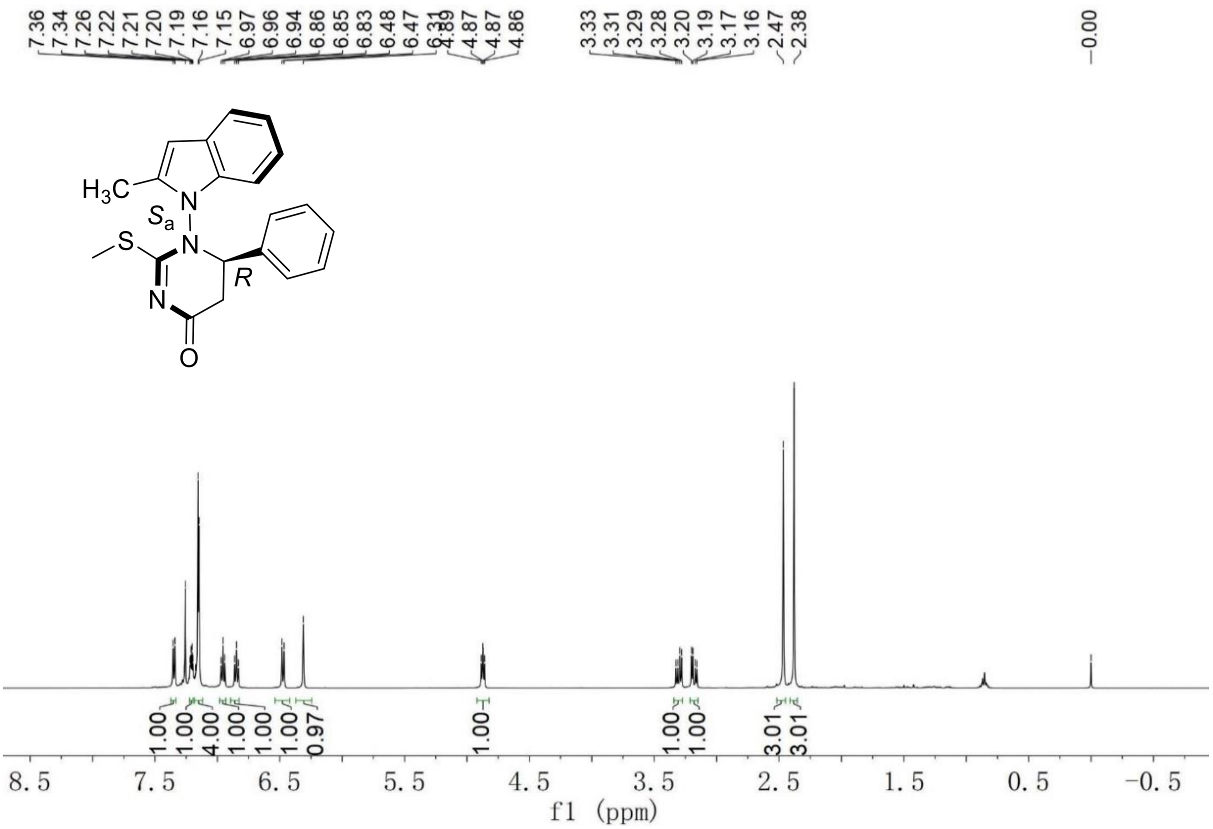

Supplementary Figure 74. ^{1}H NMR spectrum of compound 3a' ($CDCl_3$, 500 MHz, 298 K)

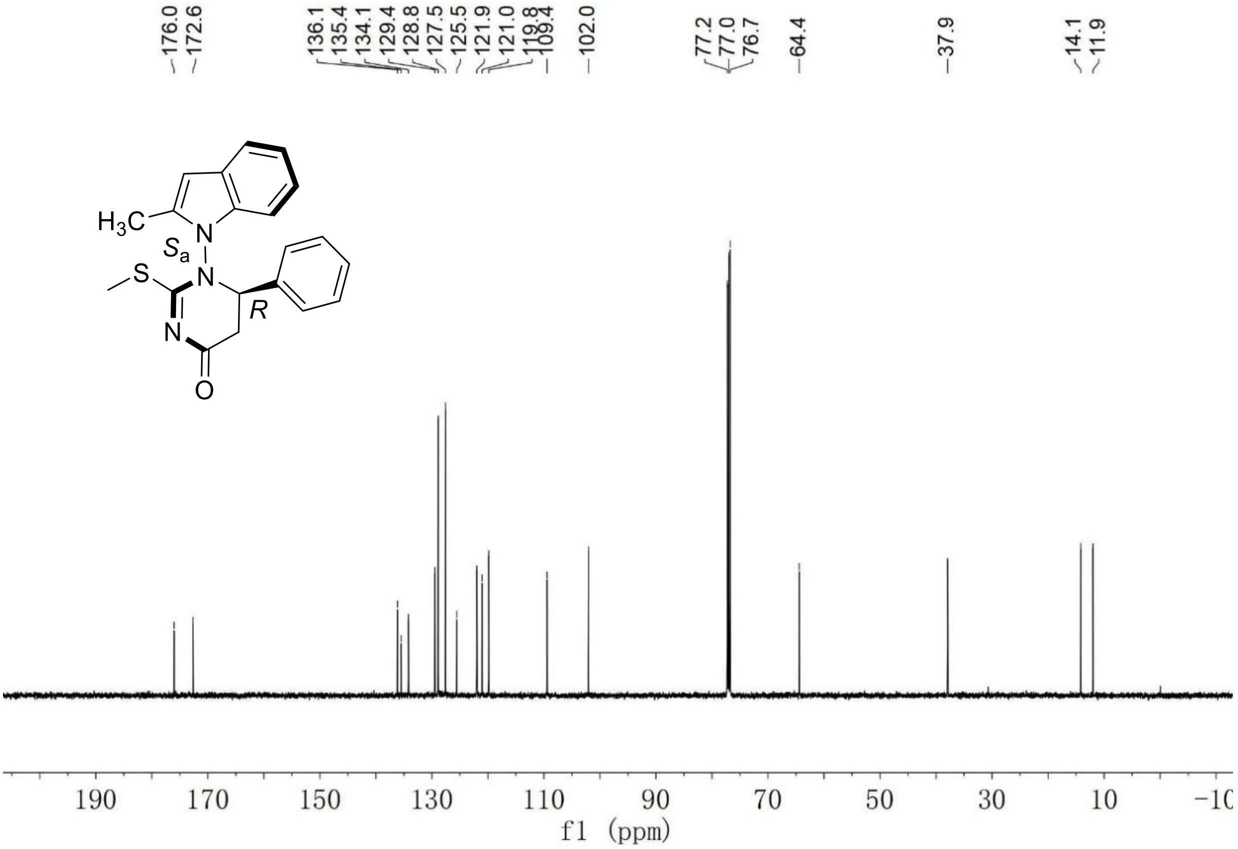

Supplementary Figure 75. ^{13}C NMR spectrum of compound 3a' ($CDCl_3$, 126 MHz, 298 K)

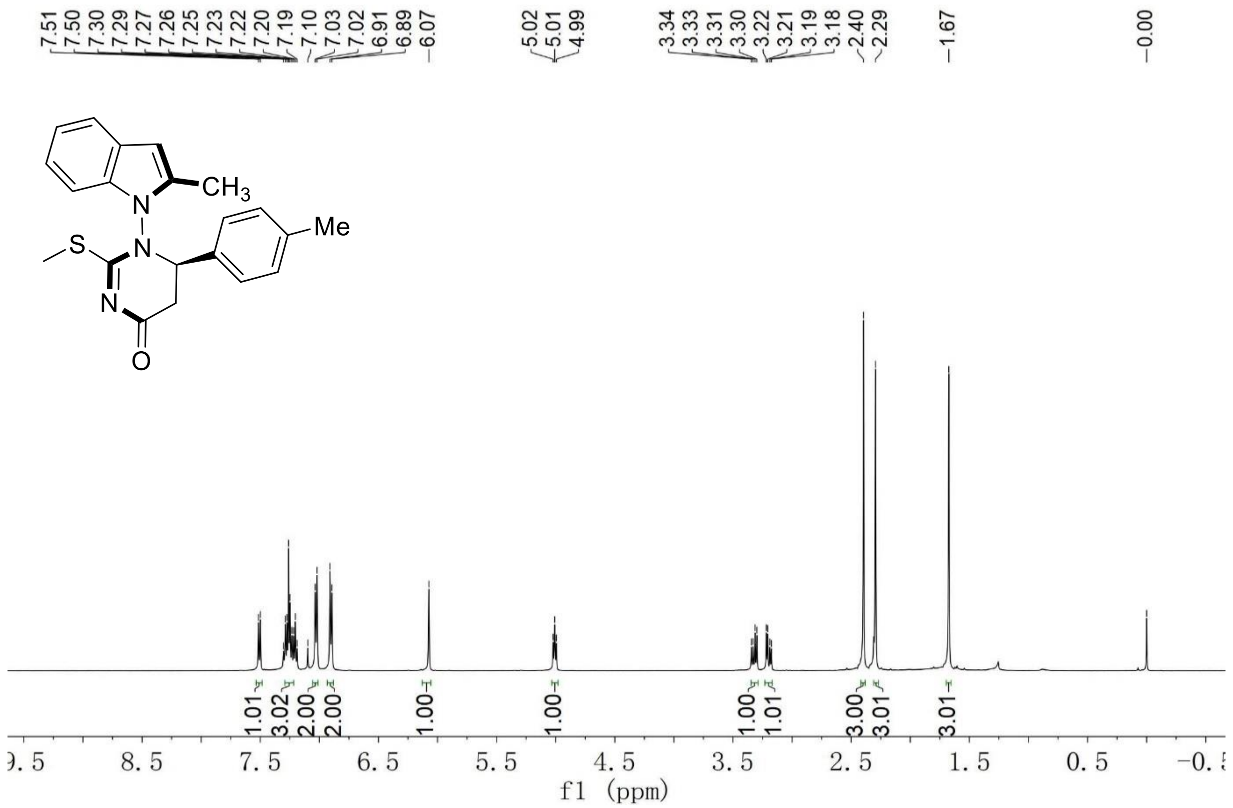

Supplementary Figure 76. ^{1}H NMR spectrum of compound 3b ($CDCl_3$, 500 MHz, 298 K)

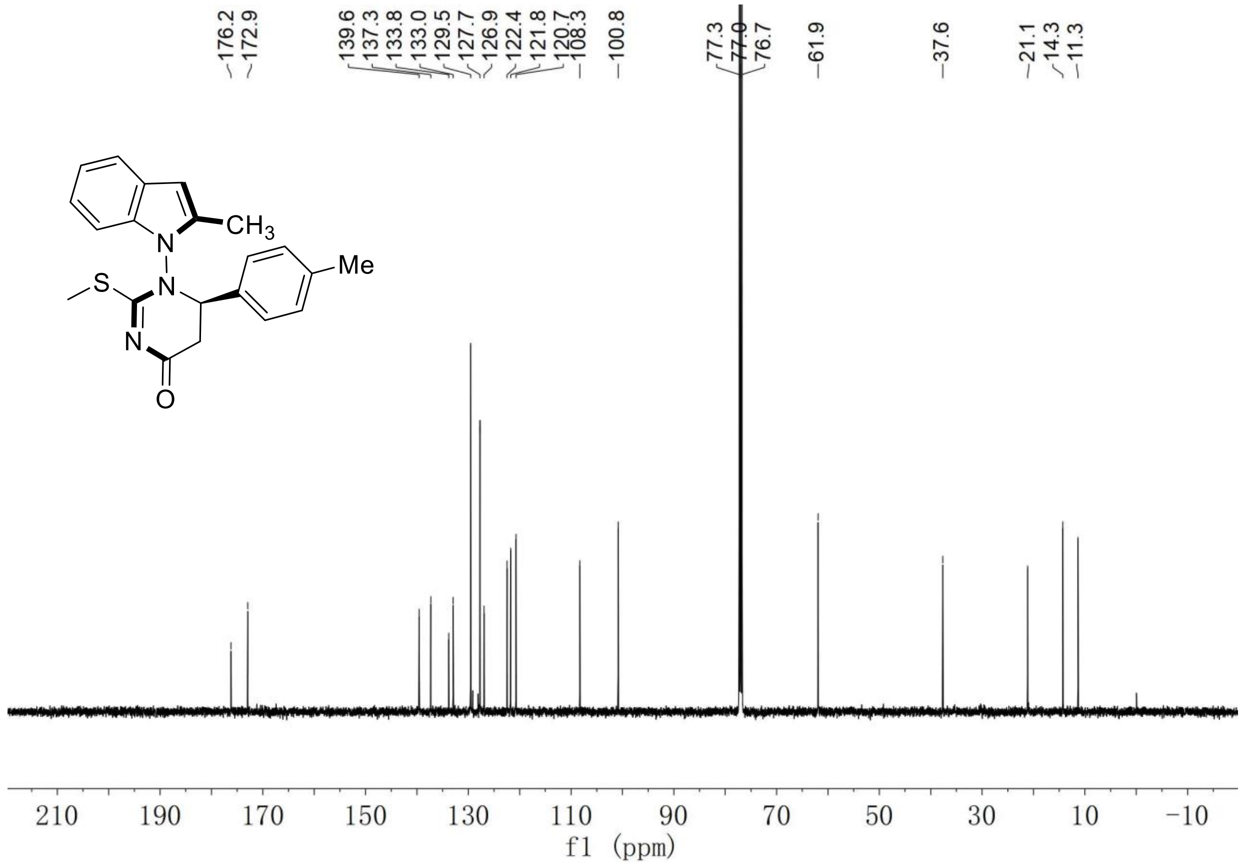

Supplementary Figure 77. ^{13}C NMR spectrum of compound 3b ($CDCl_3$, 126 MHz, 298 K)

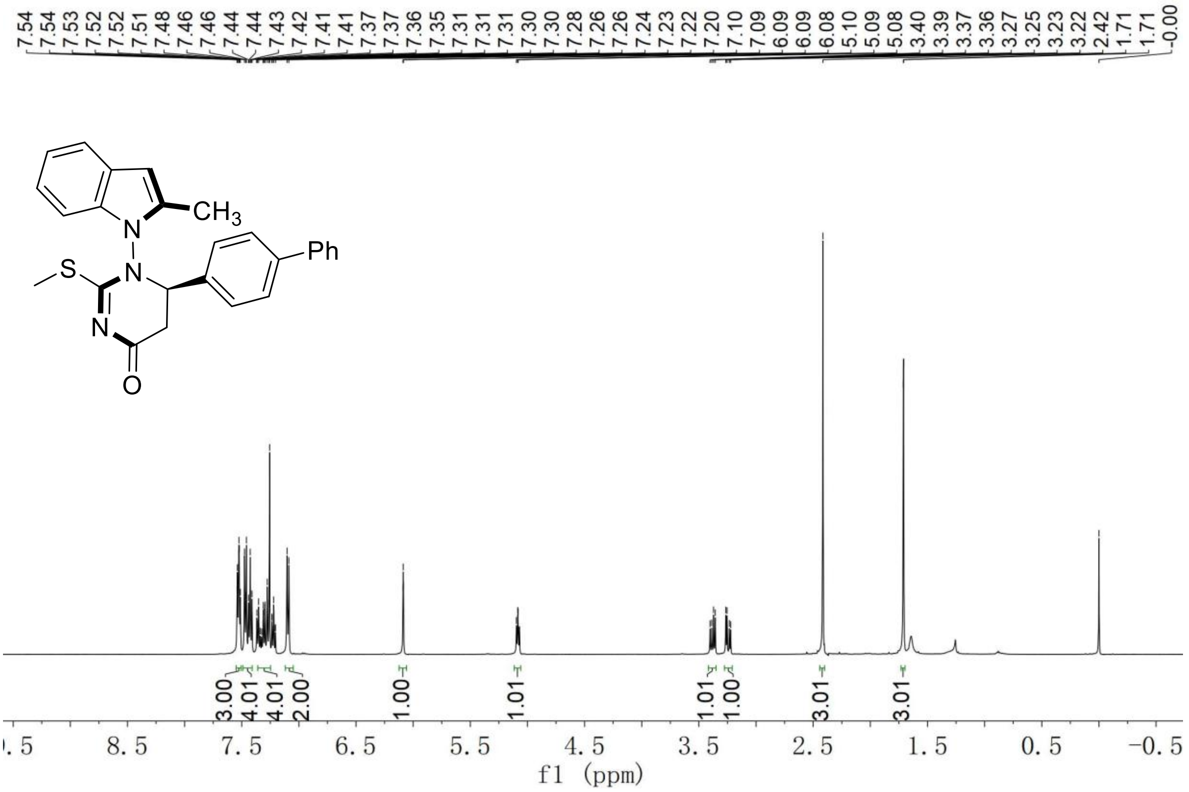

Supplementary Figure 78. ^{1}H NMR spectrum of compound 3c ($CDCl_3$, 500 MHz, 298 K)

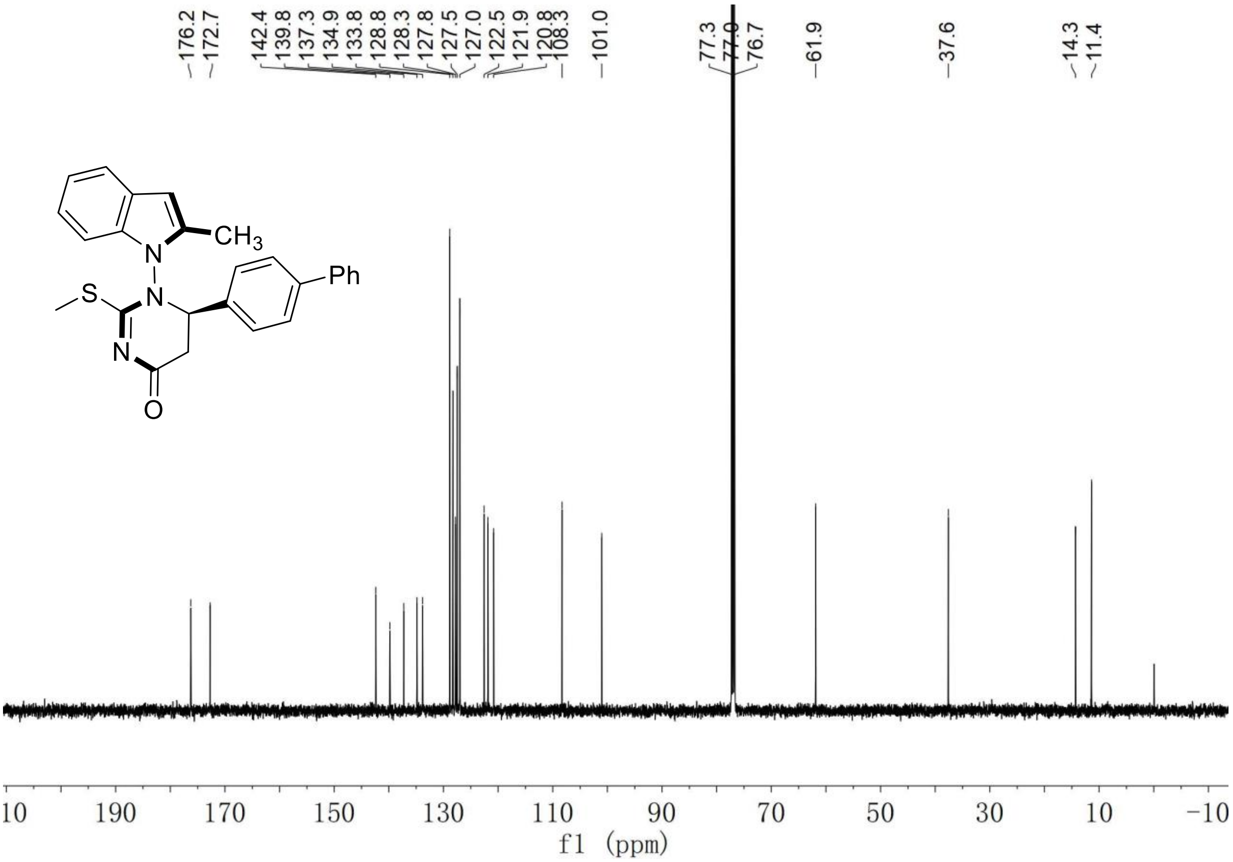

Supplementary Figure 79. ^{13}C NMR spectrum of compound 3c ($CDCl_3$, 126 MHz, 298 K)

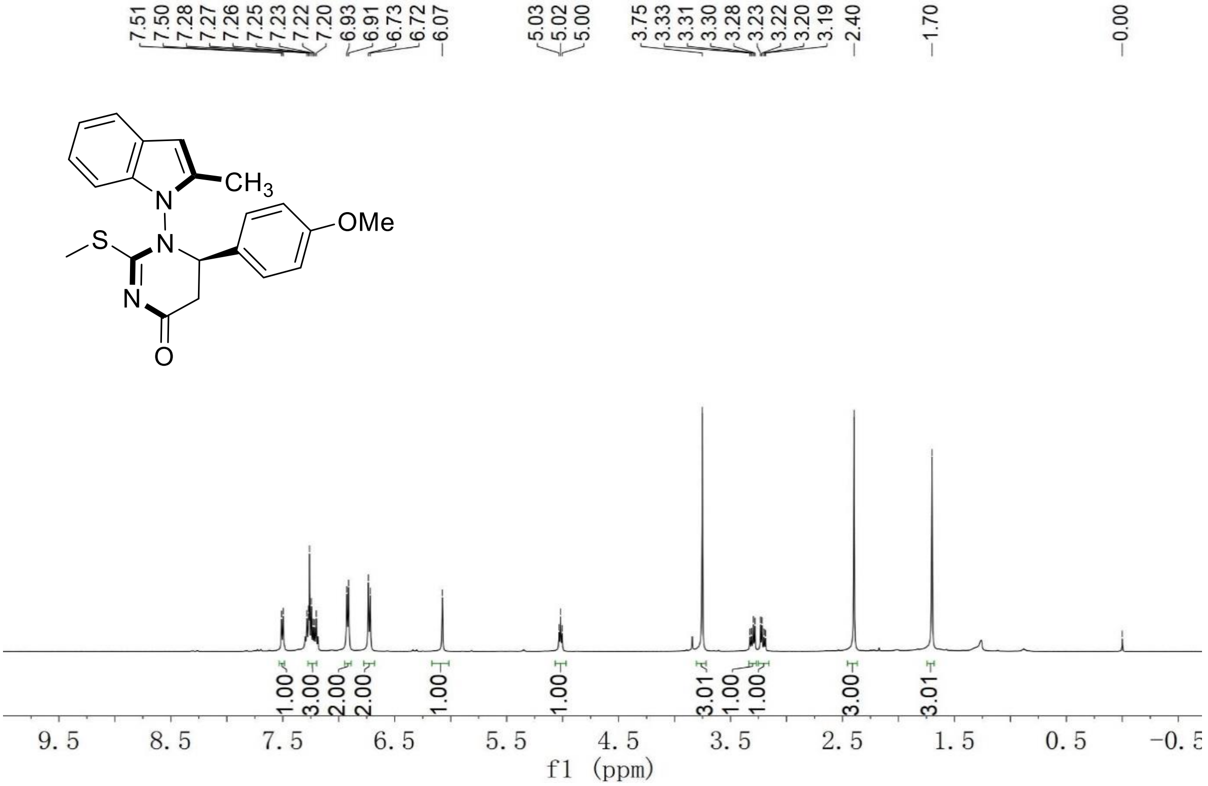

Supplementary Figure 80. ^{1}H NMR spectrum of compound 3d ($CDCl_3$, 500 MHz, 298 K)

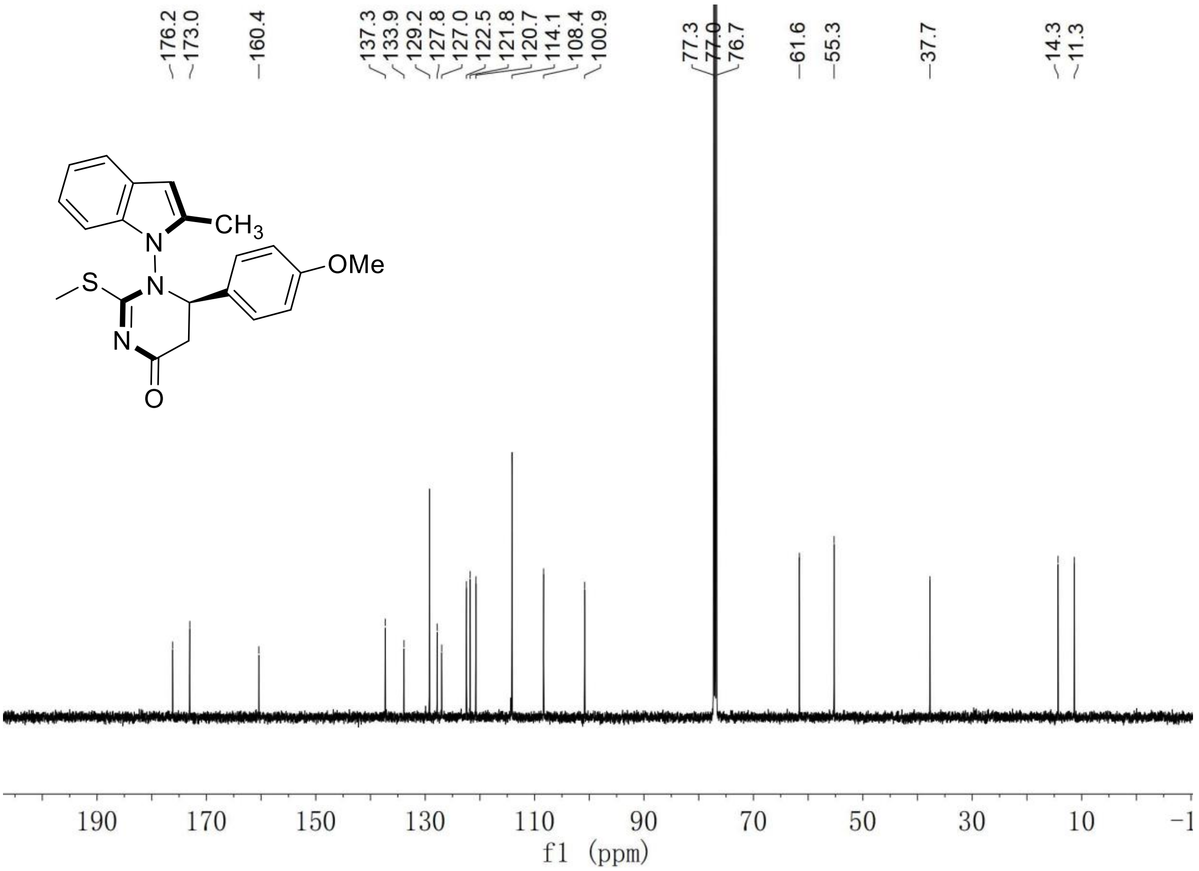

Supplementary Figure 81. ^{13}C NMR spectrum of compound 3d ($CDCl_3$, 126 MHz, 298 K)

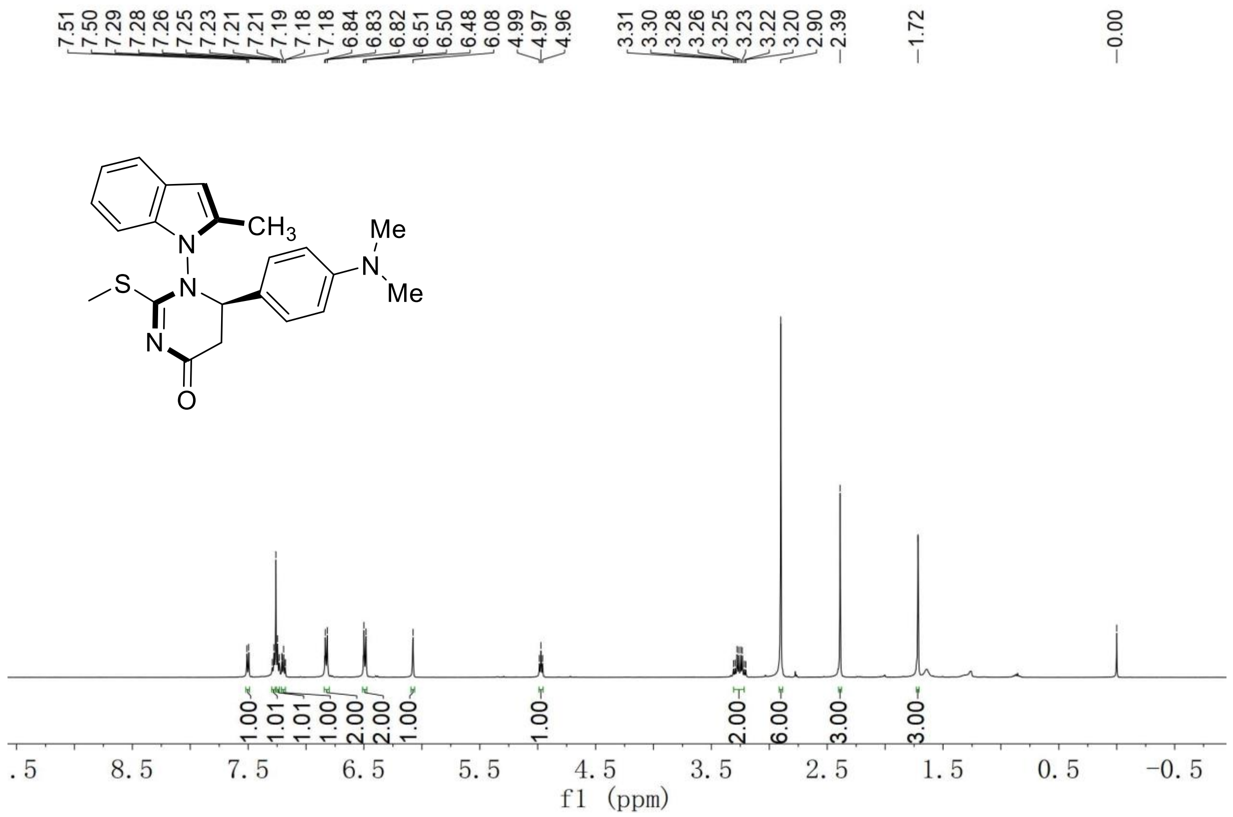

Supplementary Figure 82. ^{1}H NMR spectrum of compound 3e ($CDCl_3$, 500 MHz, 298 K)

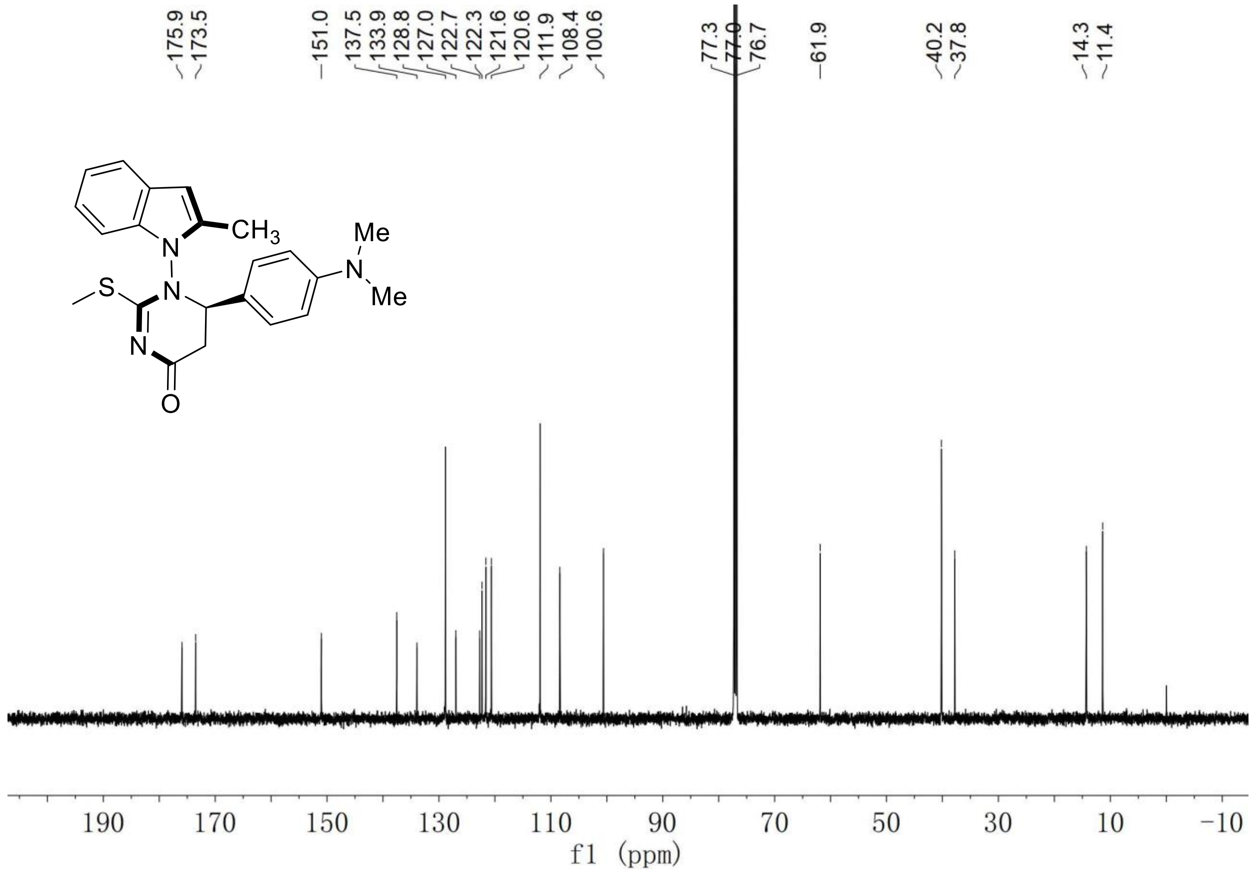

Supplementary Figure 83. ^{13}C NMR spectrum of compound 3e ($CDCl_3$, 126 MHz, 298 K)

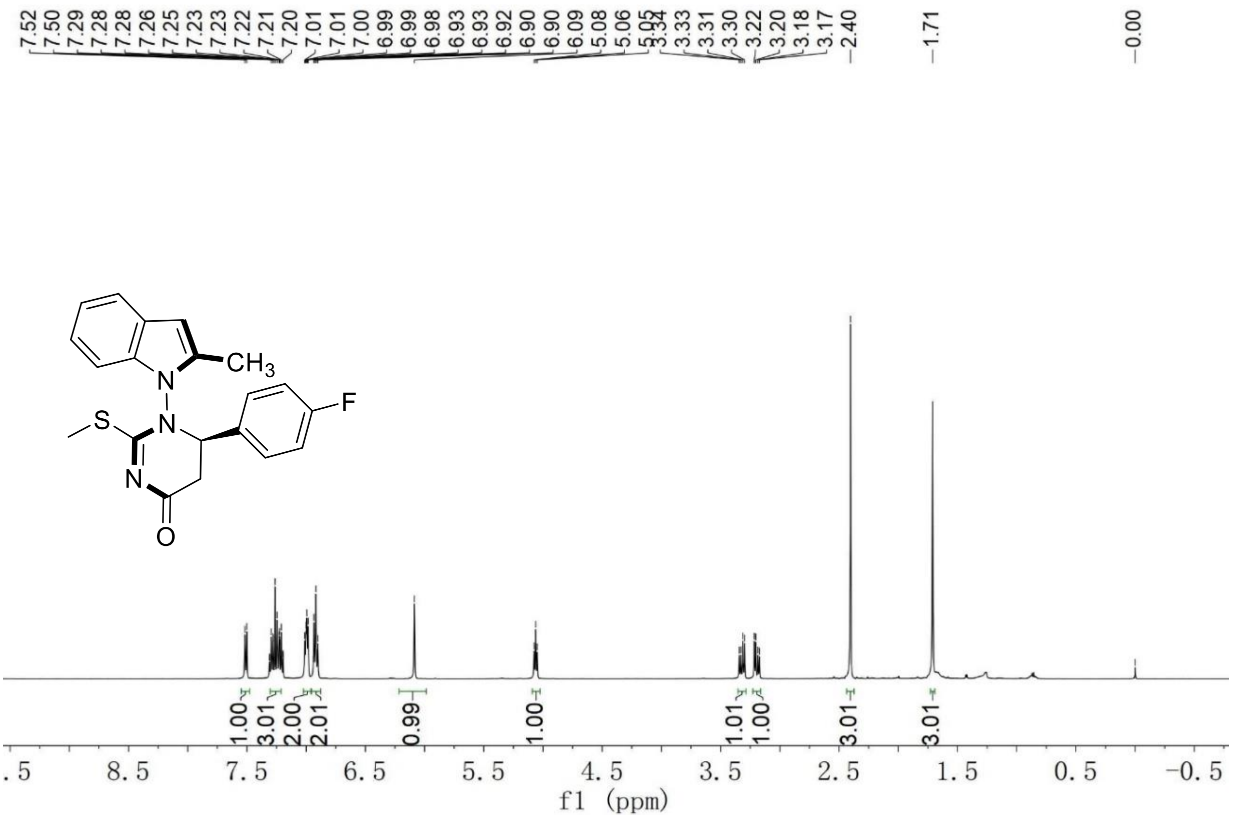

Supplementary Figure 84. ^{1}H NMR spectrum of compound 3f ($CDCl_3$, 500 MHz, 298 K)

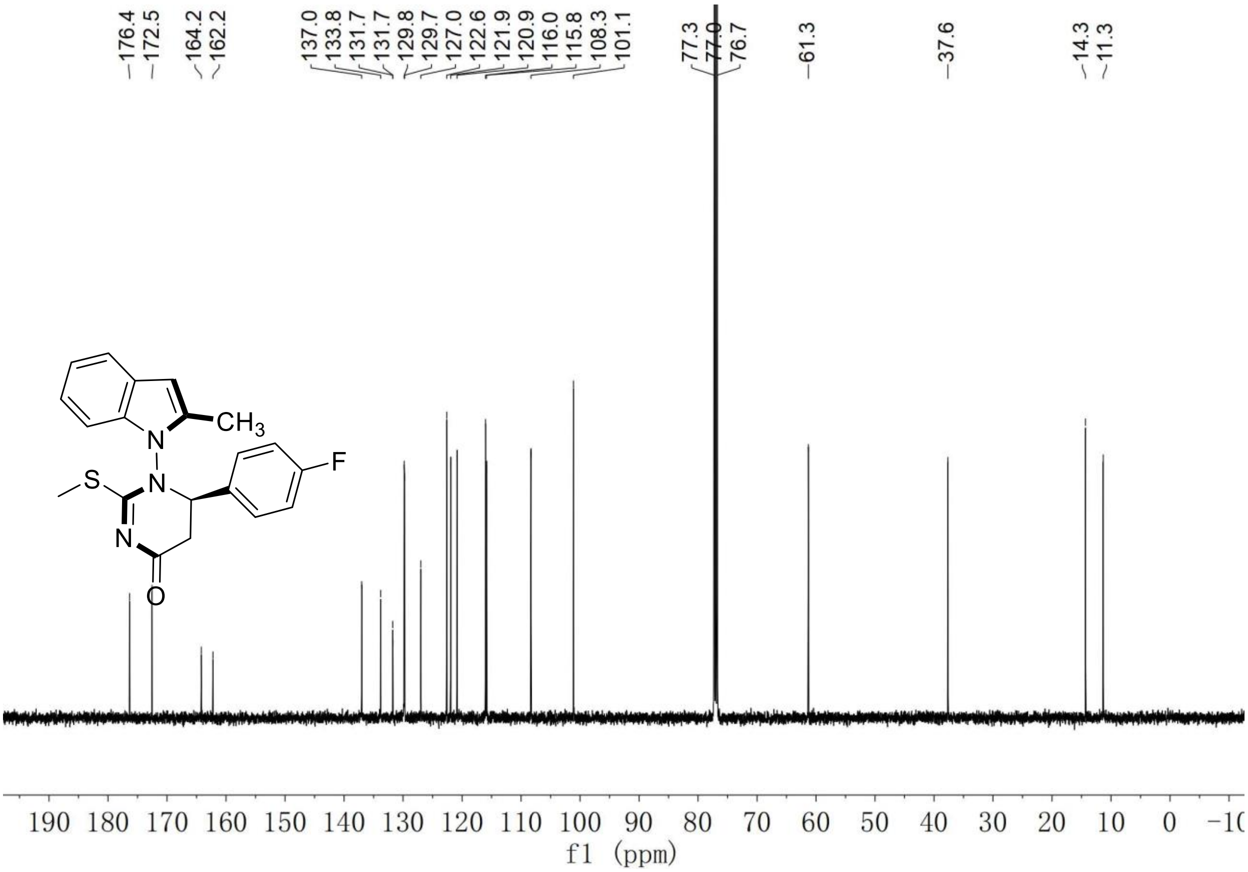

Supplementary Figure 85. ^{13}C NMR spectrum of compound 3f ($CDCl_3$, 126 MHz, 298 K)

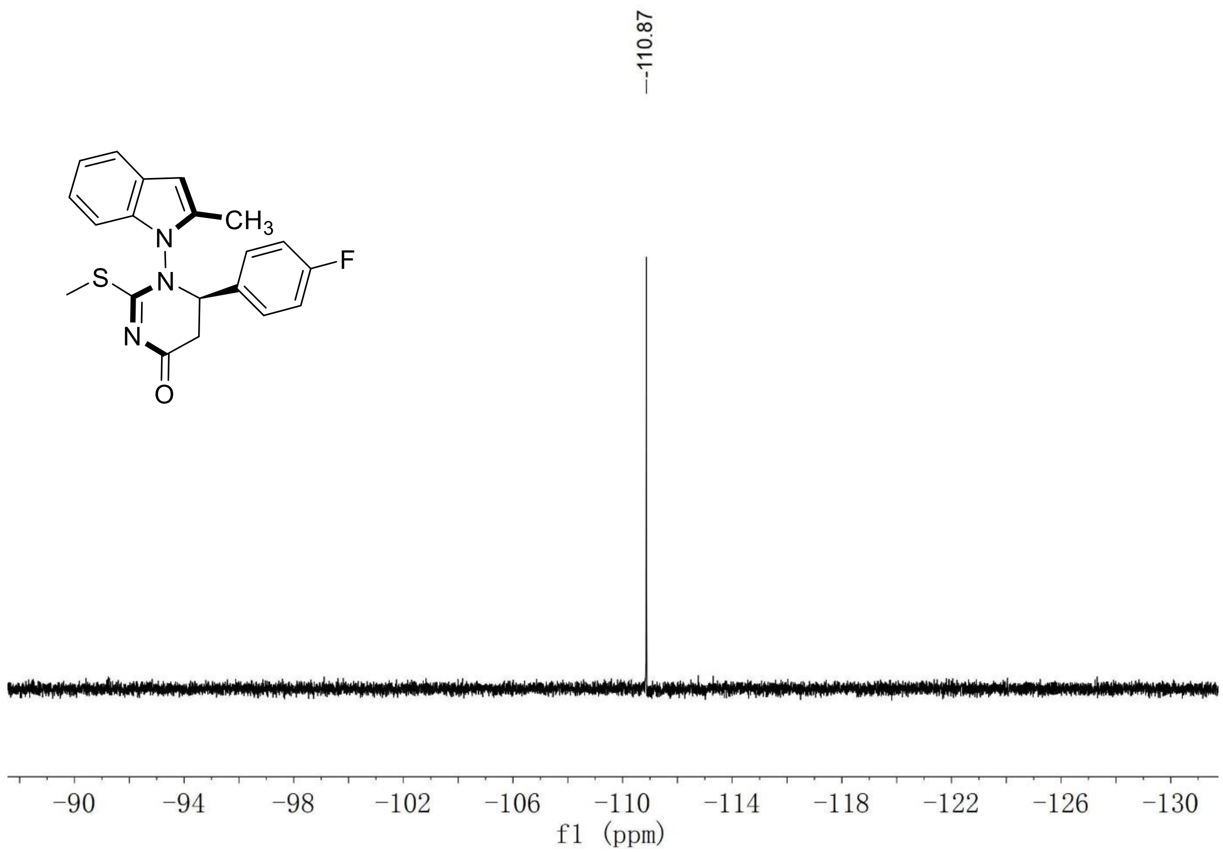

Supplementary Figure 86. ^{19}F NMR spectrum of compound 3f ($CDCl_3$, 471 MHz, 298 K)

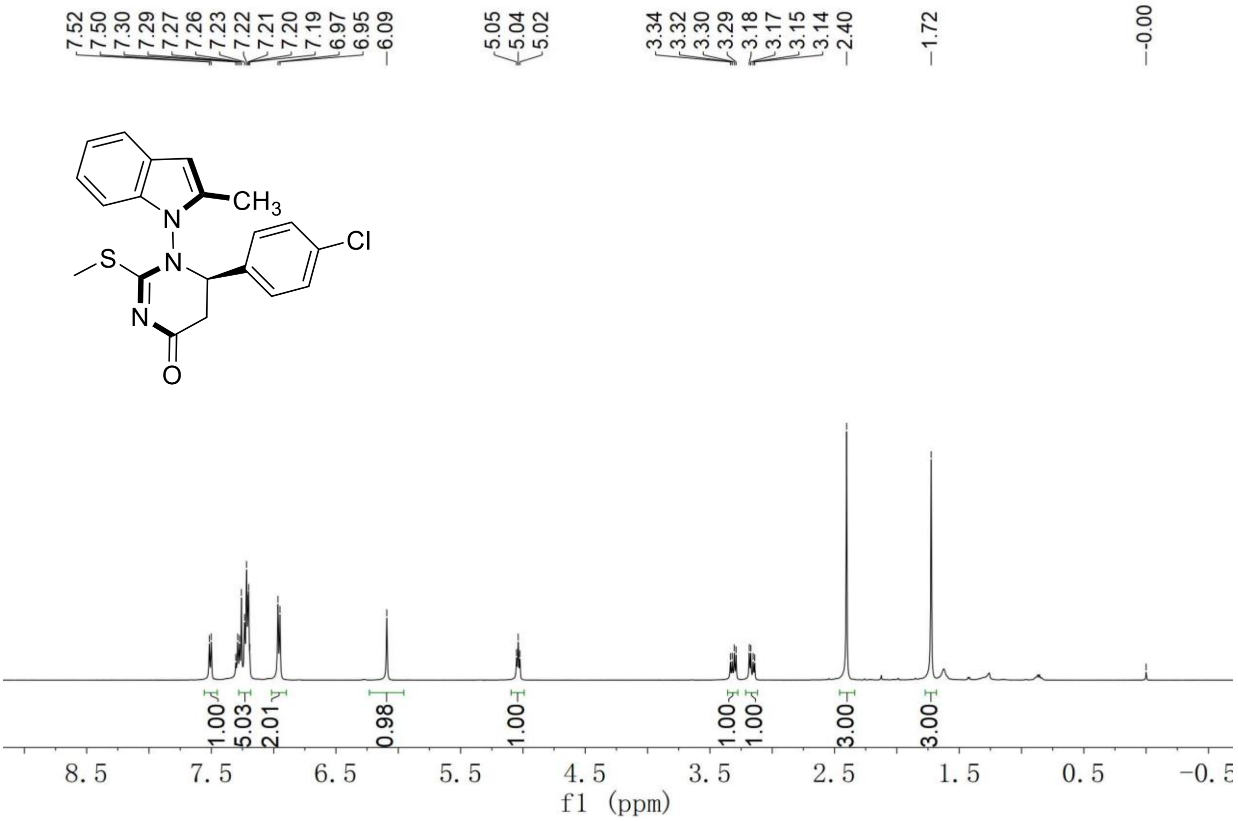

Supplementary Figure 87. ^{1}H NMR spectrum of compound 3g ($CDCl_3$, 500 MHz, 298 K)

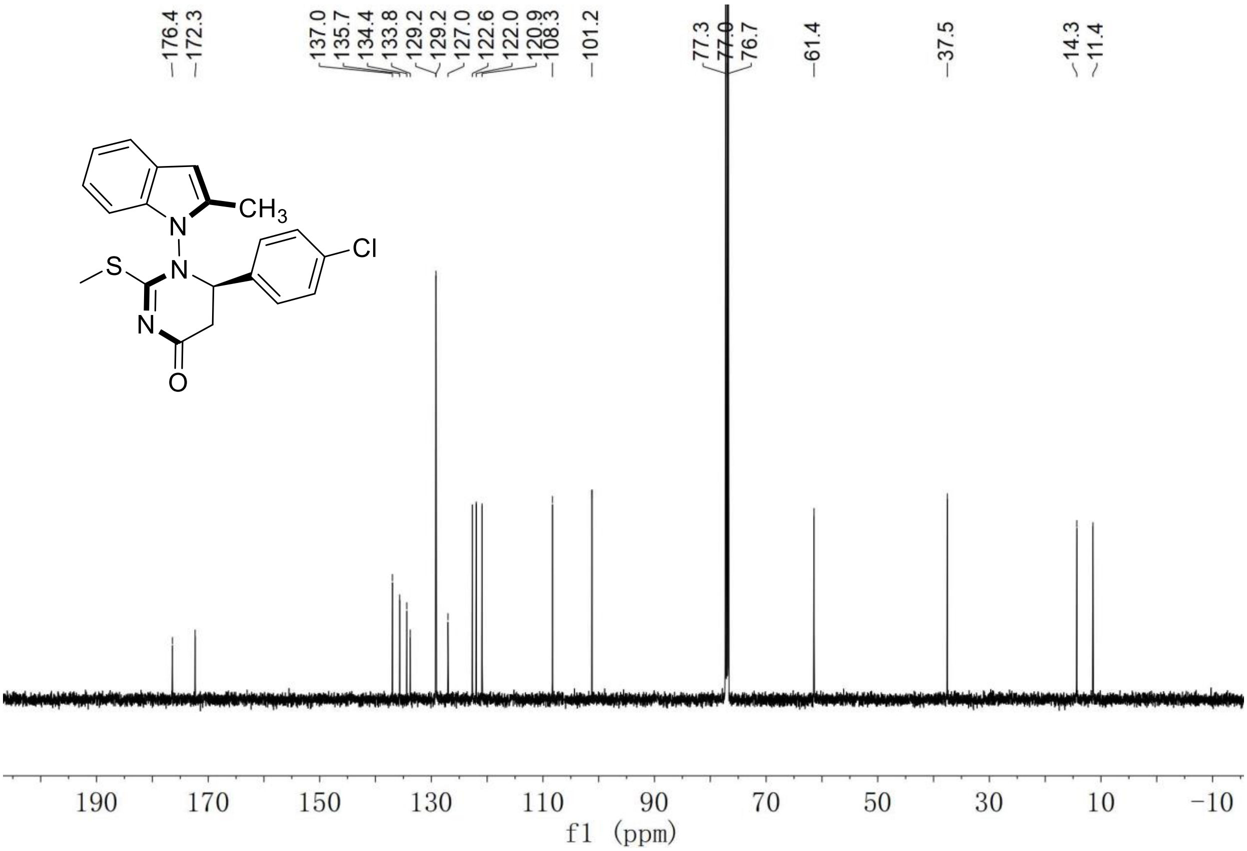

Supplementary Figure 88. ^{13}C NMR spectrum of compound 3g ($CDCl_3$, 126 MHz, 298 K)

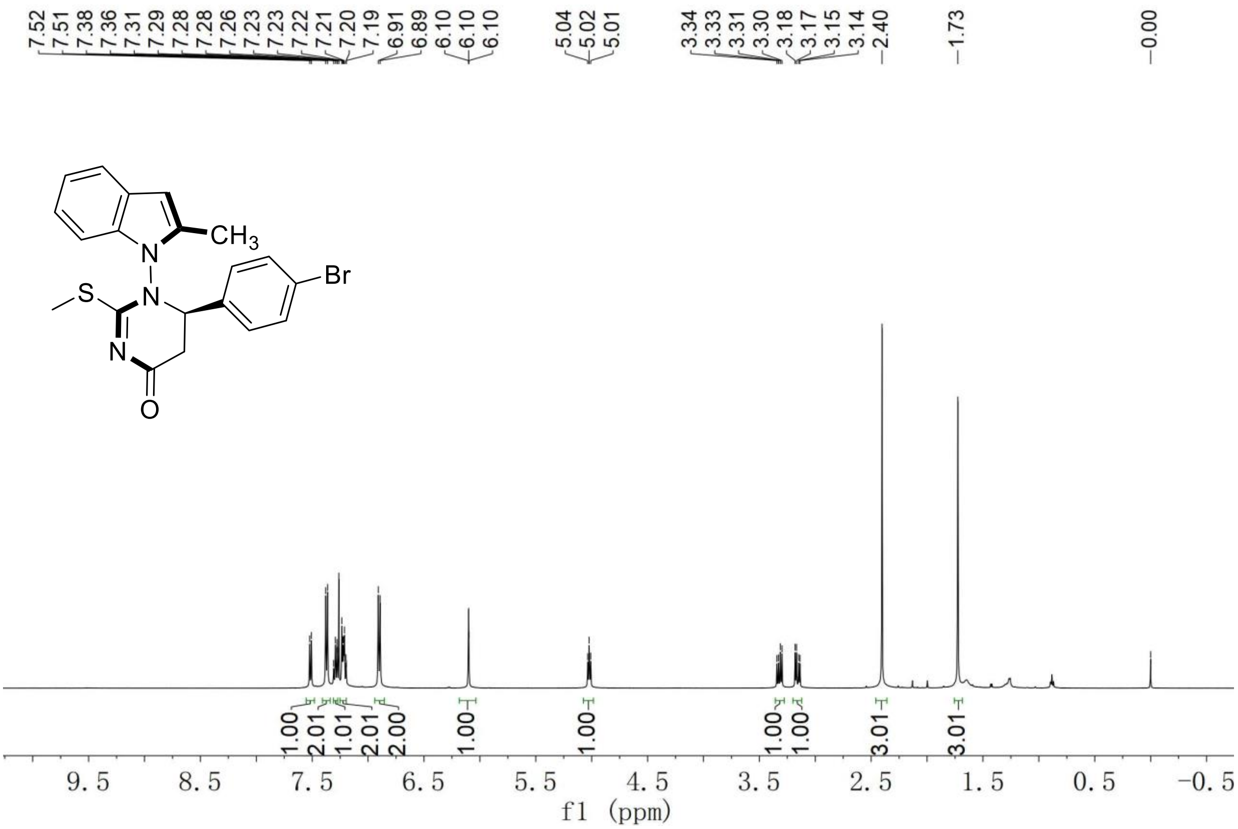

Supplementary Figure 89. ^{1}H NMR spectrum of compound 3h ($CDCl_3$, 500 MHz, 298 K)

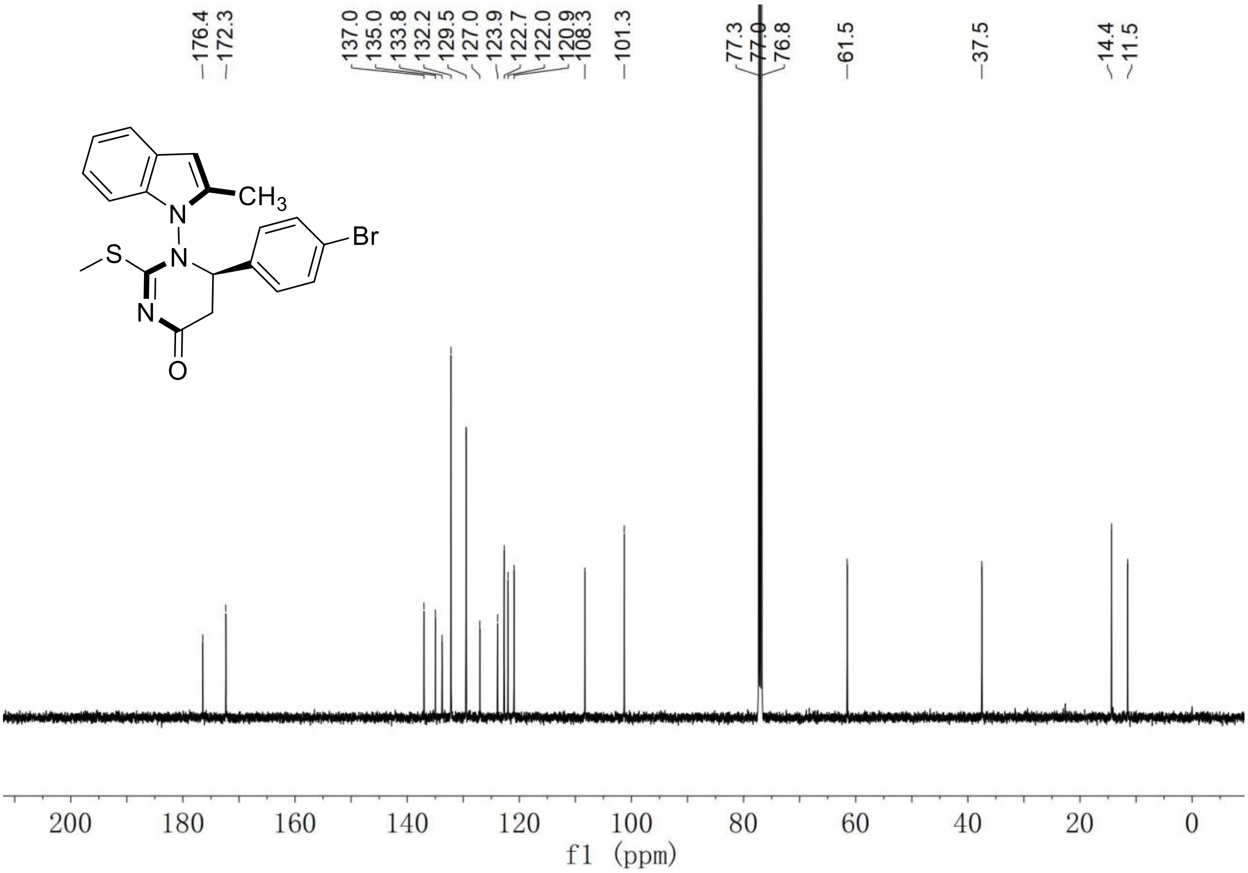

Supplementary Figure 90. ^{13}C NMR spectrum of compound 3h ($CDCl_3$, 126 MHz, 298 K)

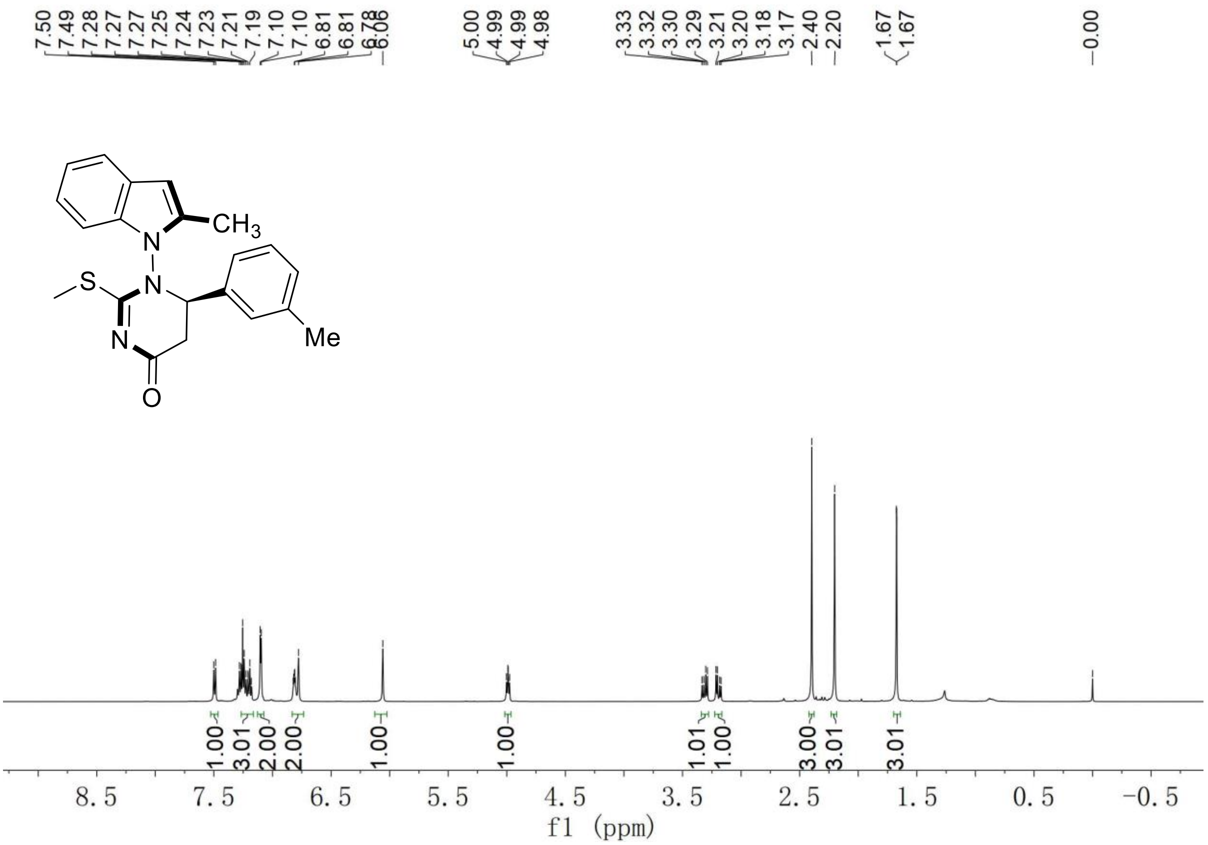

Supplementary Figure 91. ^{1}H NMR spectrum of compound 3i ($CDCl_3$, 500 MHz, 298 K)

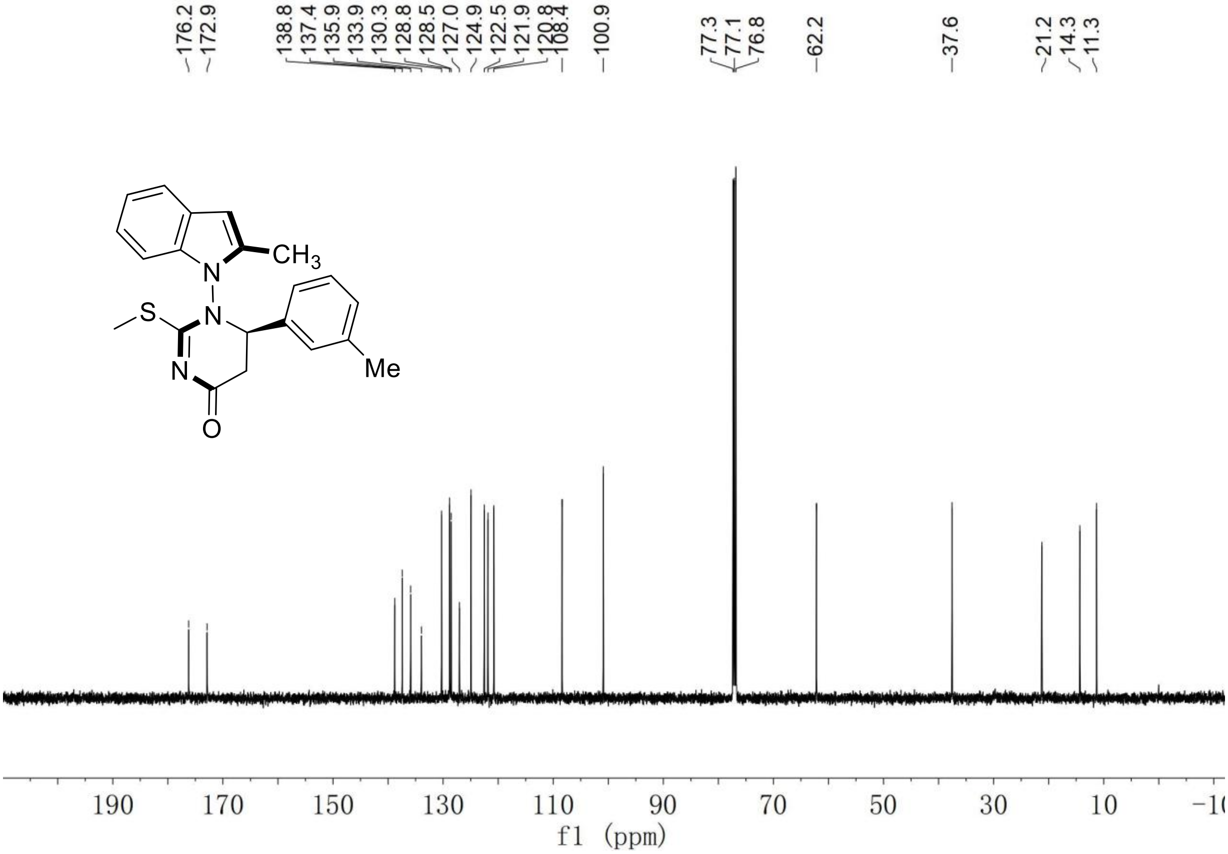

Supplementary Figure 92. ^{13}C NMR spectrum of compound 3i ($CDCl_3$, 126 MHz, 298 K)

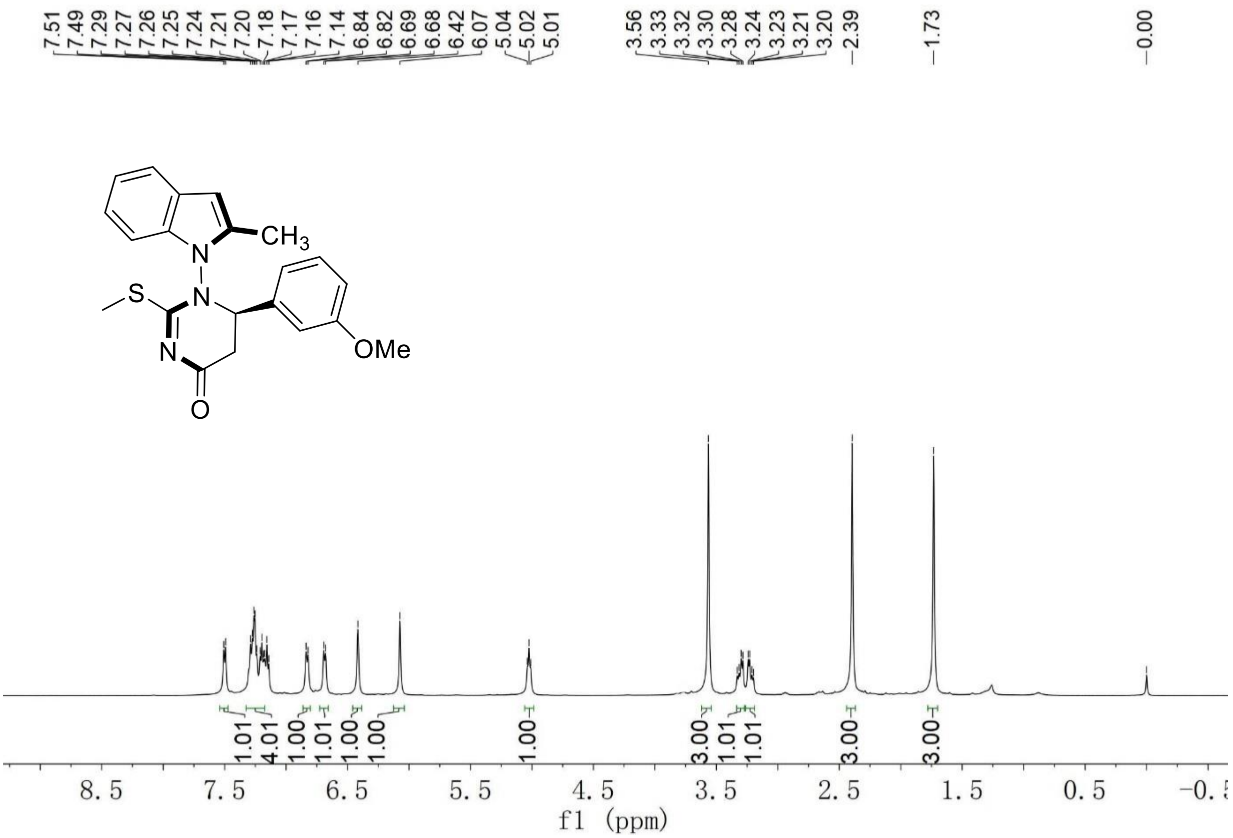

Supplementary Figure 93. ^{1}**H NMR spectrum of compound 3j (CDCl$_3$, 500 MHz, 298 K)**

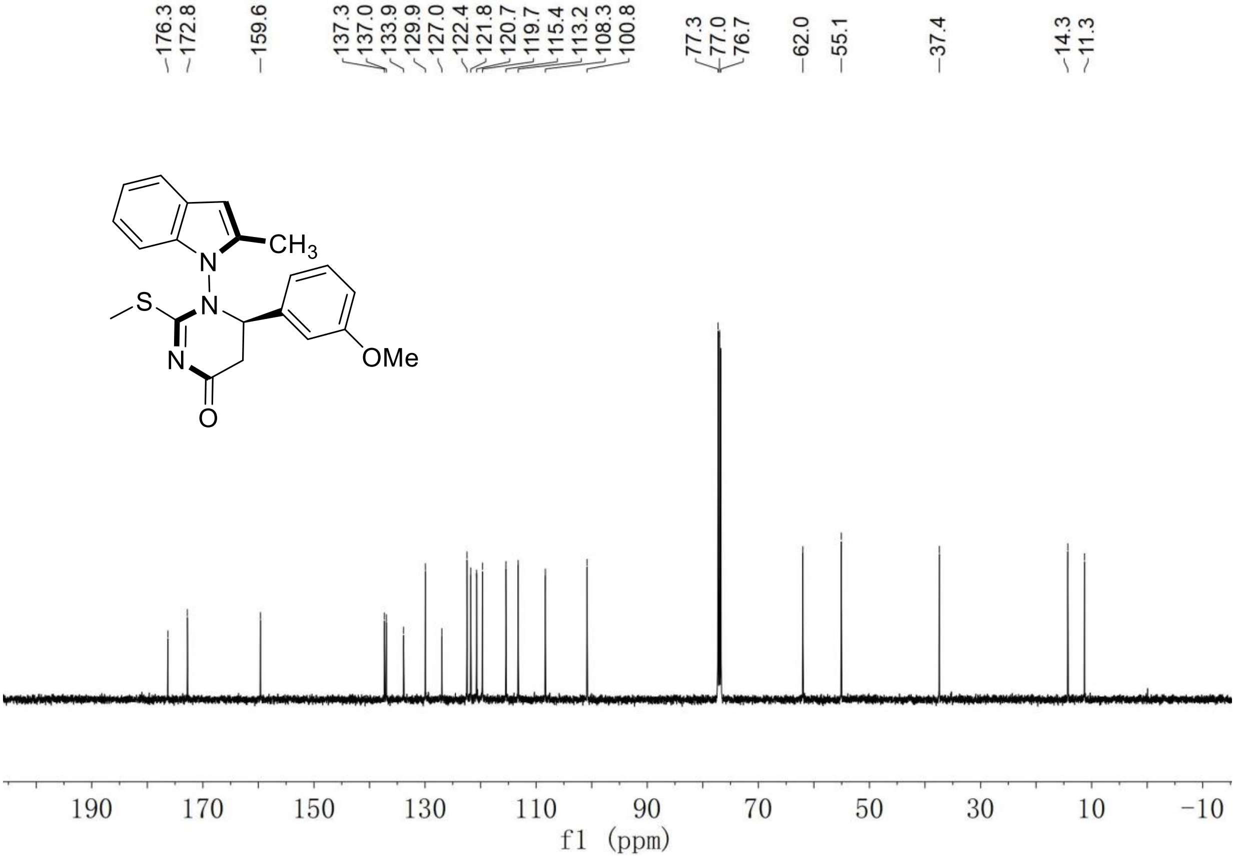

Supplementary Figure 94. ^{13}**C NMR spectrum of compound 3j (CDCl$_3$, 126 MHz, 298 K)**

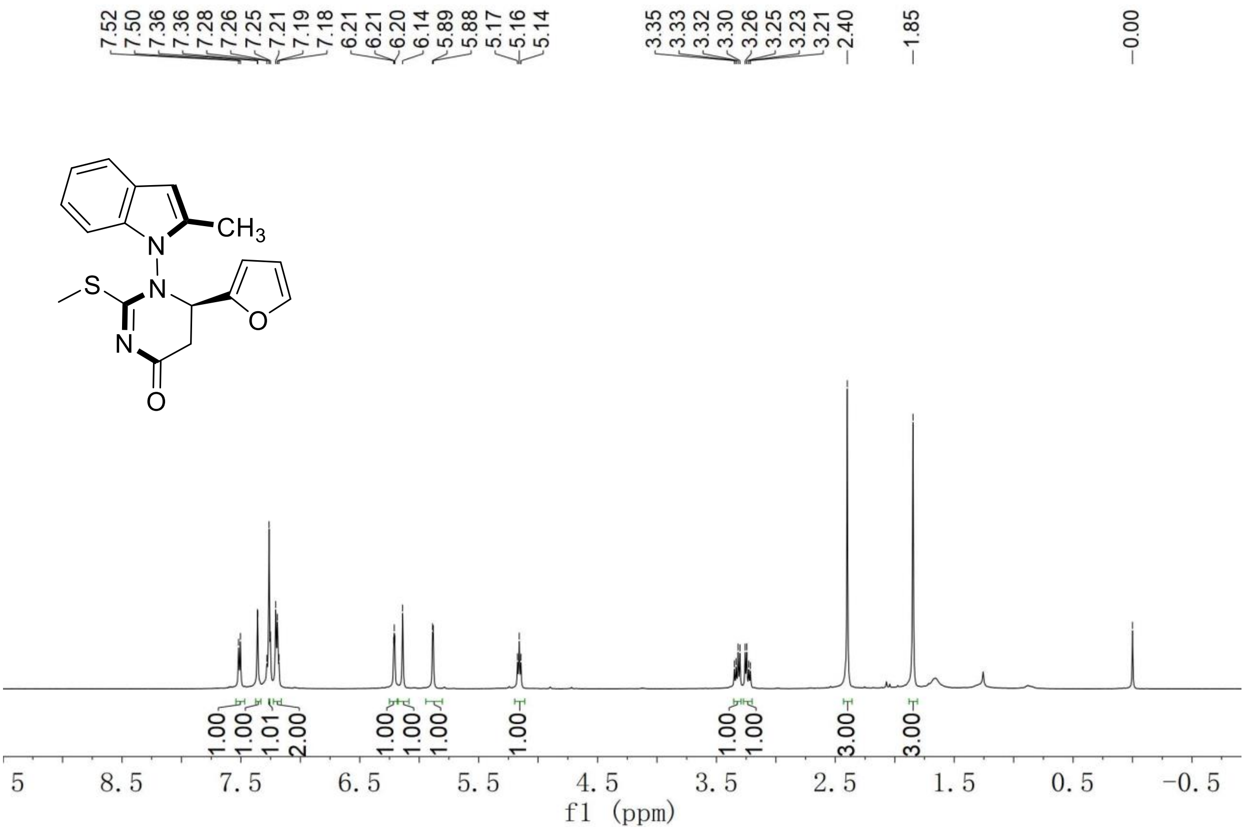

Supplementary Figure 95. ^{1}H NMR spectrum of compound 3k ($CDCl_3$, 500 MHz, 298 K)

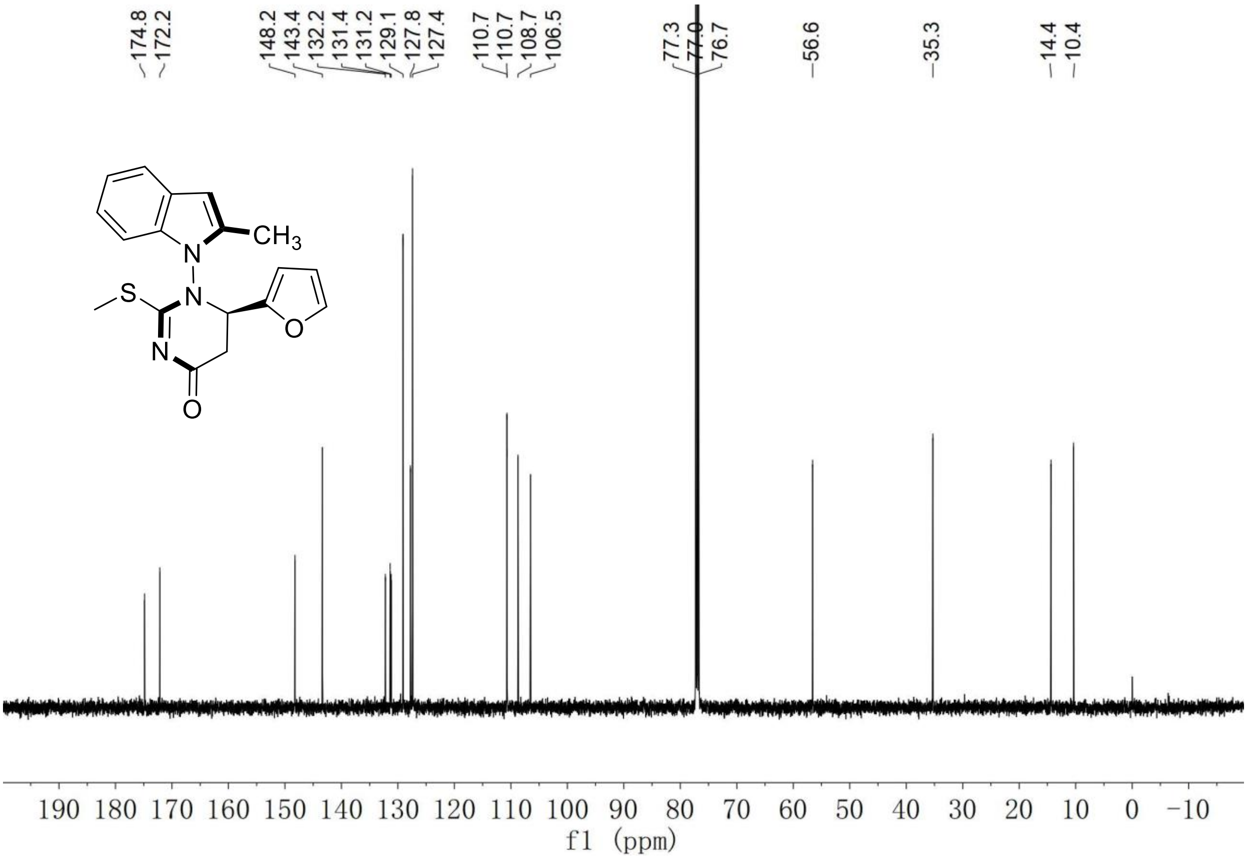

Supplementary Figure 96. ^{13}C NMR spectrum of compound 3k ($CDCl_3$, 126 MHz, 298 K)

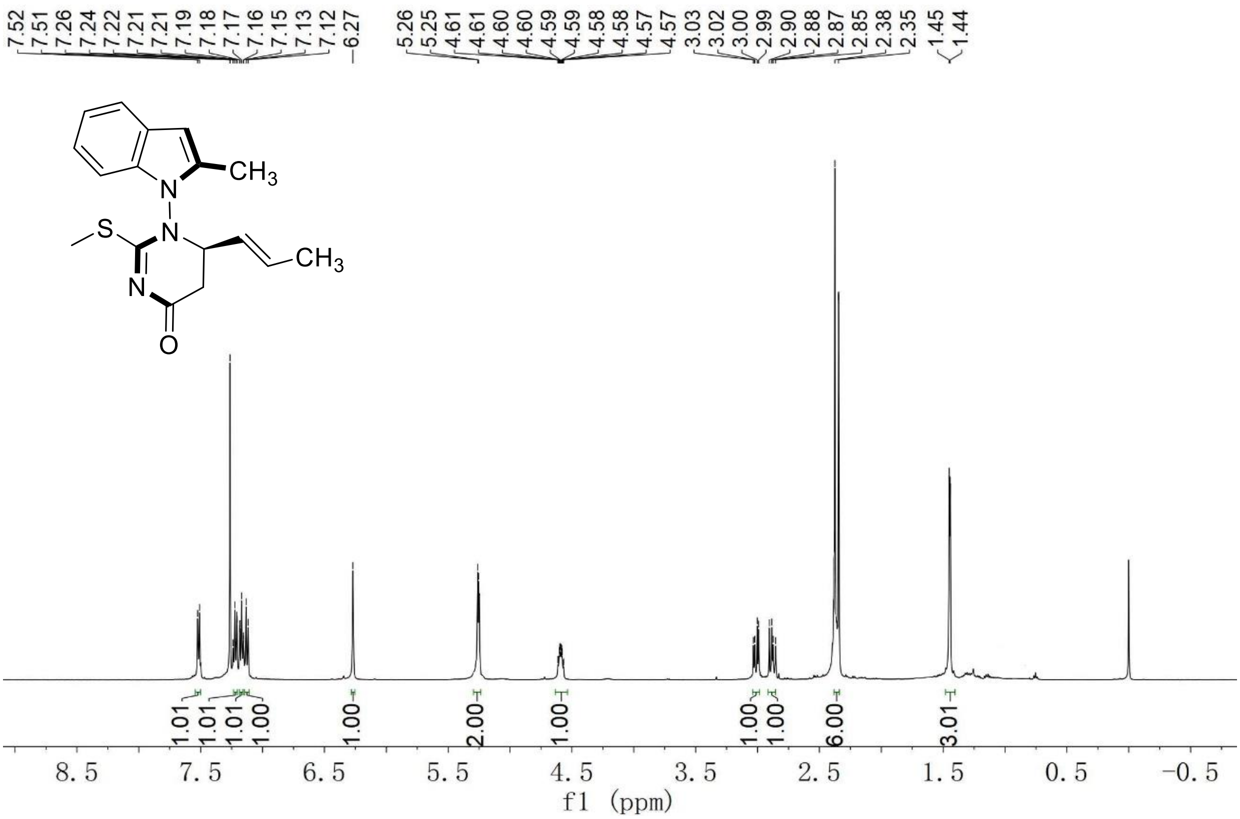

Supplementary Figure 97. ^{1}H NMR spectrum of compound 3l ($CDCl_3$, 500 MHz, 298 K)

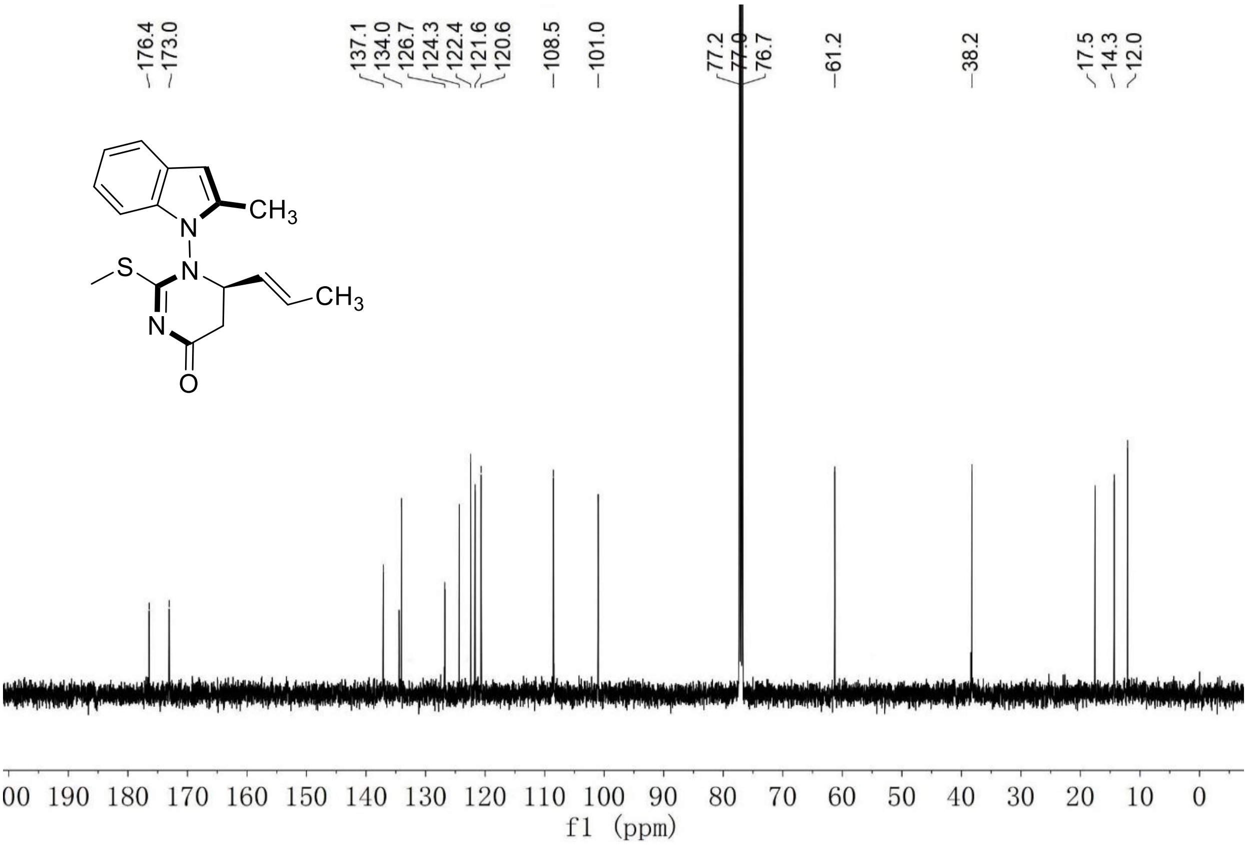

Supplementary Figure 98. ^{13}C NMR spectrum of compound 3l ($CDCl_3$, 126 MHz, 298 K)

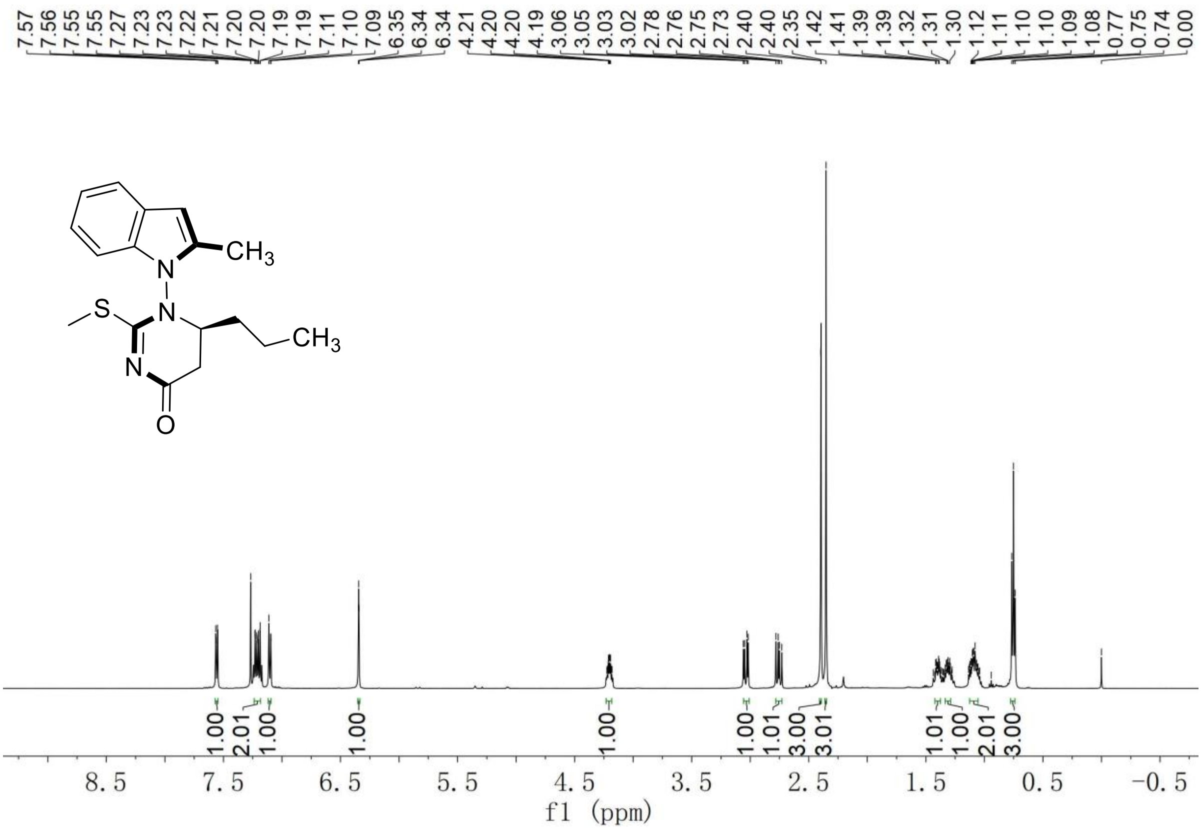

Supplementary Figure 99. ^{1}**H NMR spectrum of compound 3m (**$CDCl_3$**, 500 MHz, 298 K)**

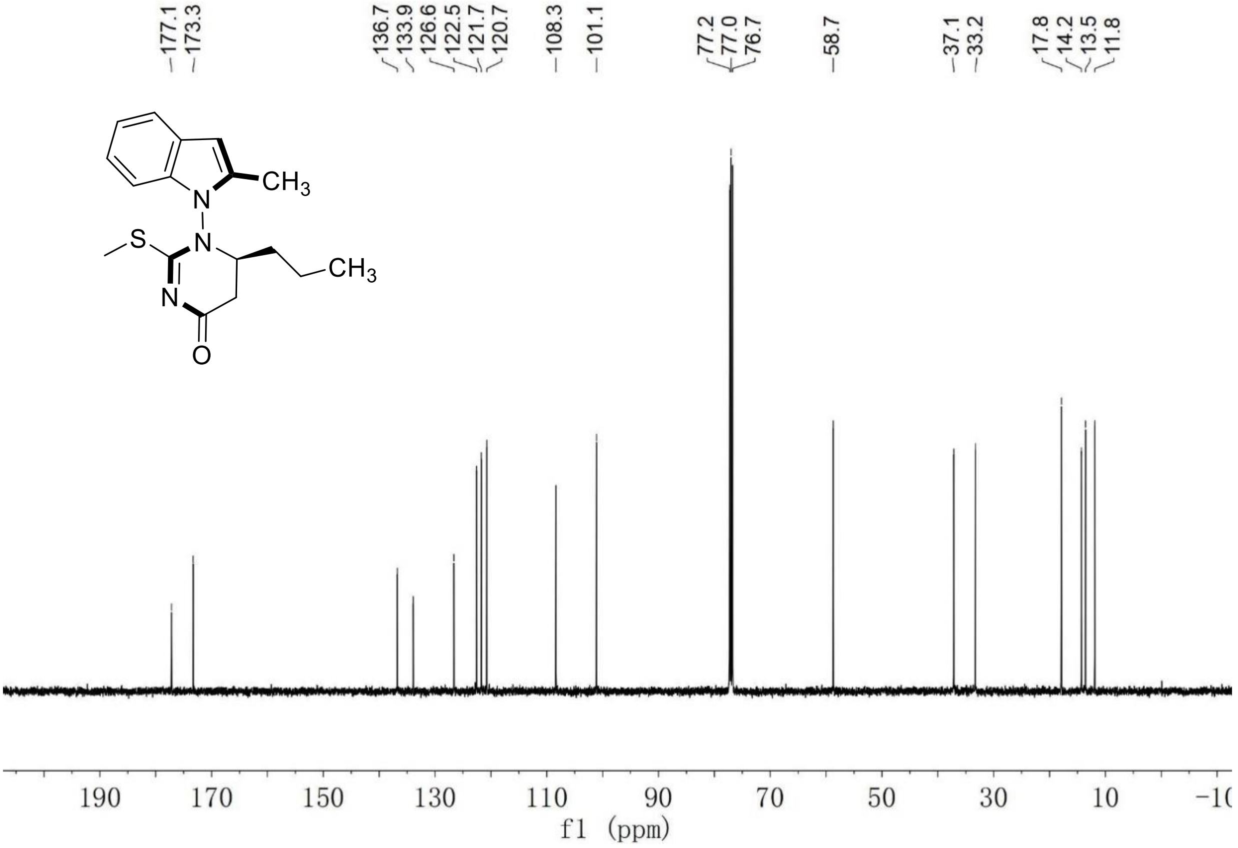

Supplementary Figure 100. ^{13}**C NMR spectrum of compound 3m (**$CDCl_3$**, 126 MHz, 298 K)**

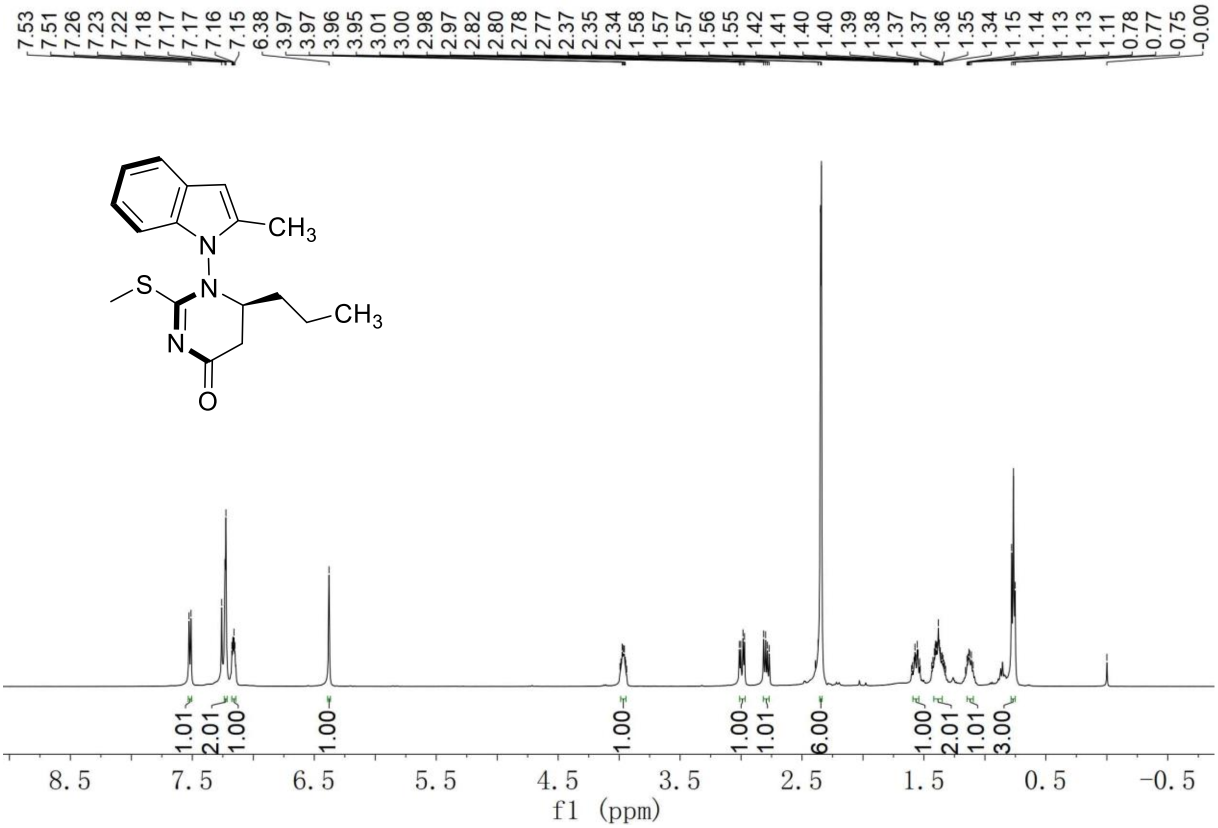

Supplementary Figure 101. ^{1}**H NMR spectrum of compound 3m' (CDCl$_3$, 500 MHz, 298 K)**

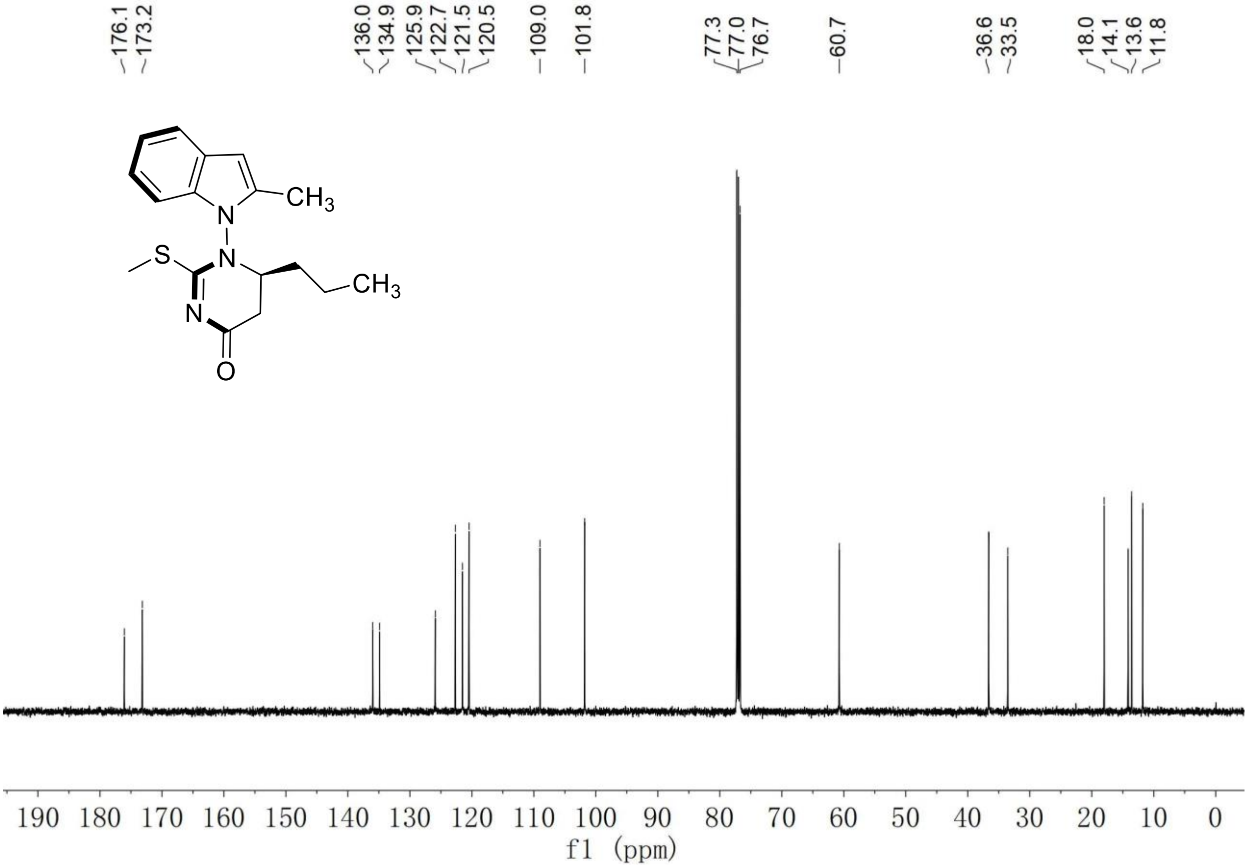

Supplementary Figure 102. ^{13}**C NMR spectrum of compound 3m' (CDCl$_3$, 126 MHz, 298 K)**

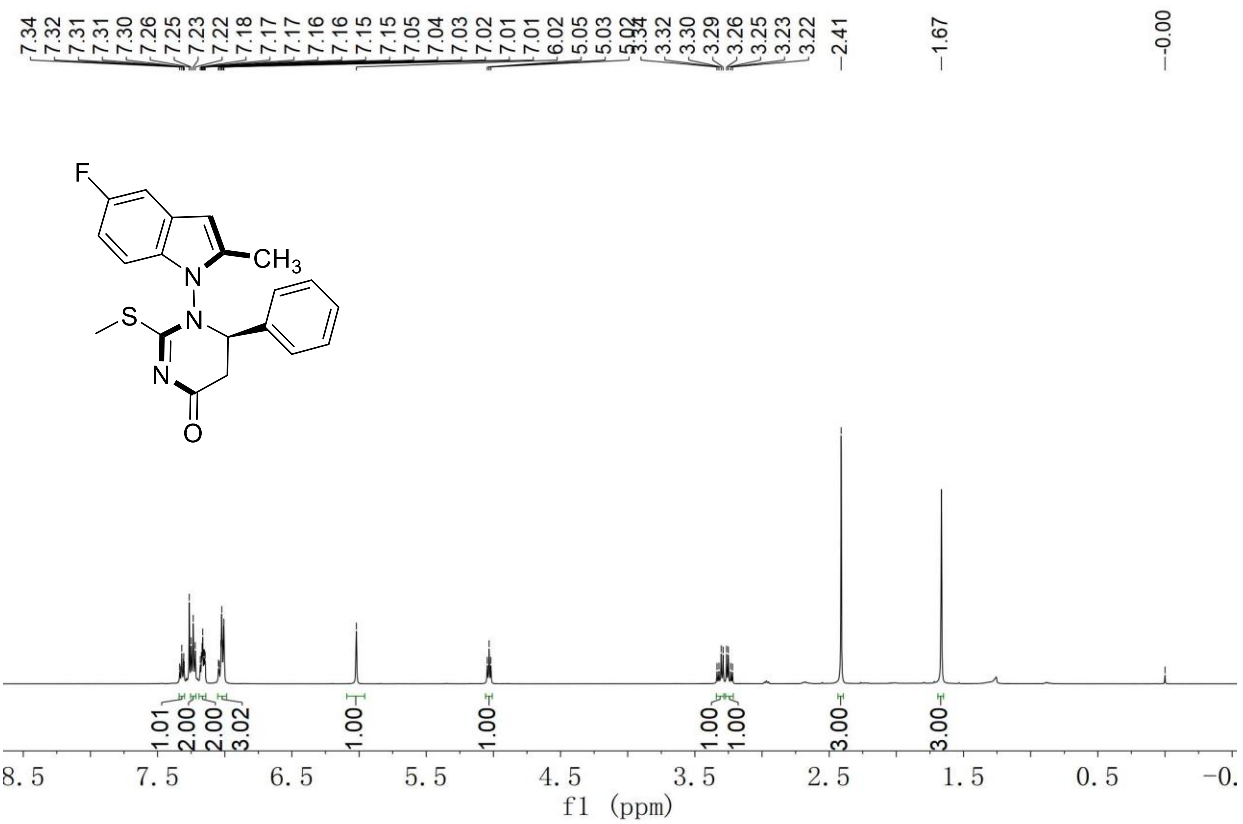

Supplementary Figure 103. ^{1}H NMR spectrum of compound 3n ($CDCl_3$, 500 MHz, 298 K)

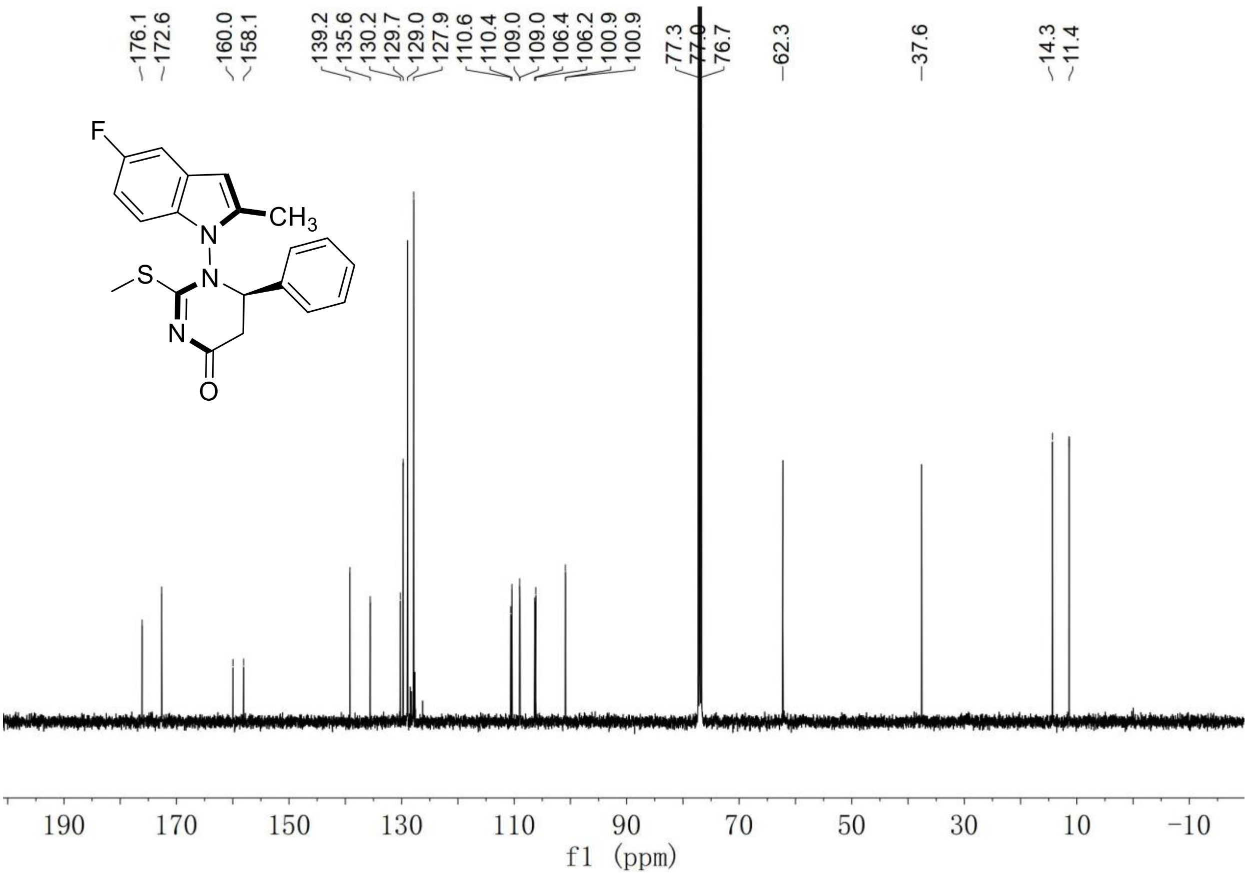

Supplementary Figure 104. ^{13}C NMR spectrum of compound 3n ($CDCl_3$, 126 MHz, 298 K)

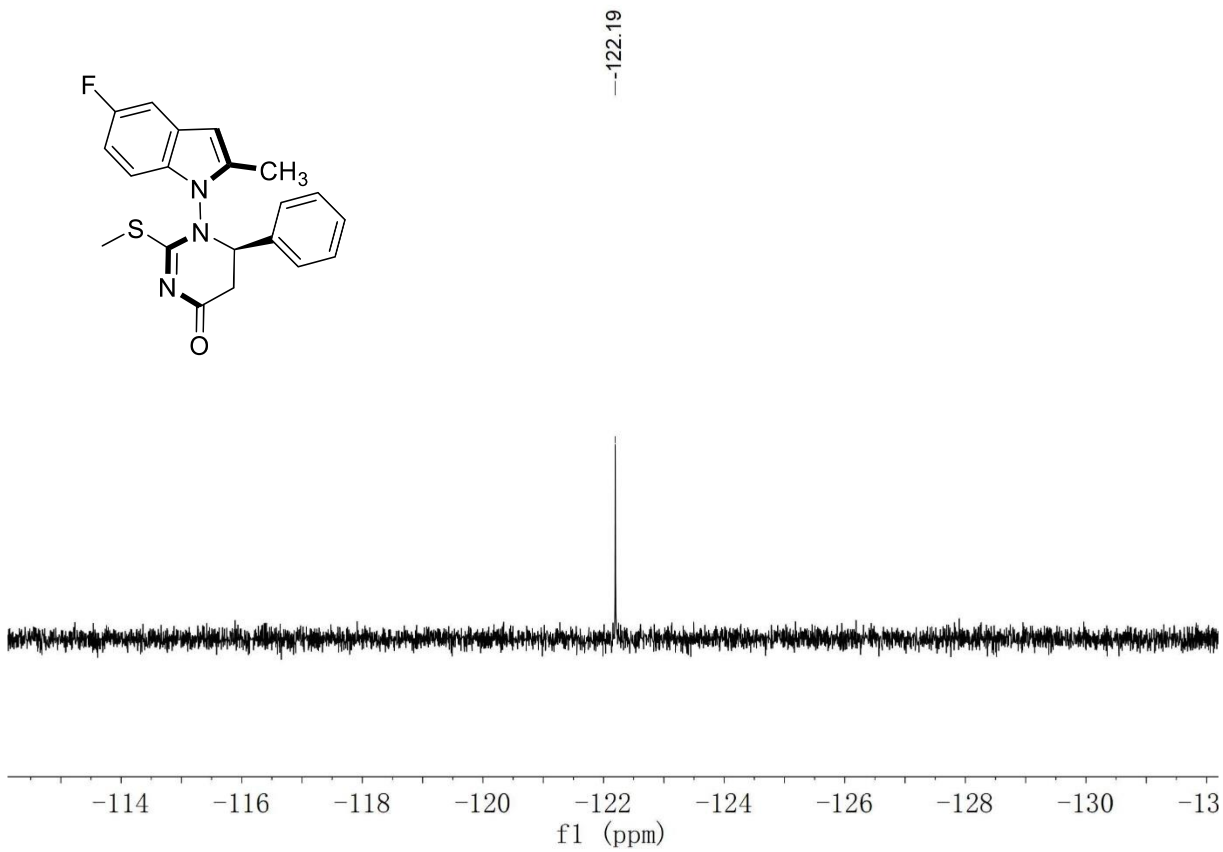

Supplementary Figure 105. ^{19}F NMR spectrum of compound 3n ($CDCl_3$, 471 MHz, 298 K)

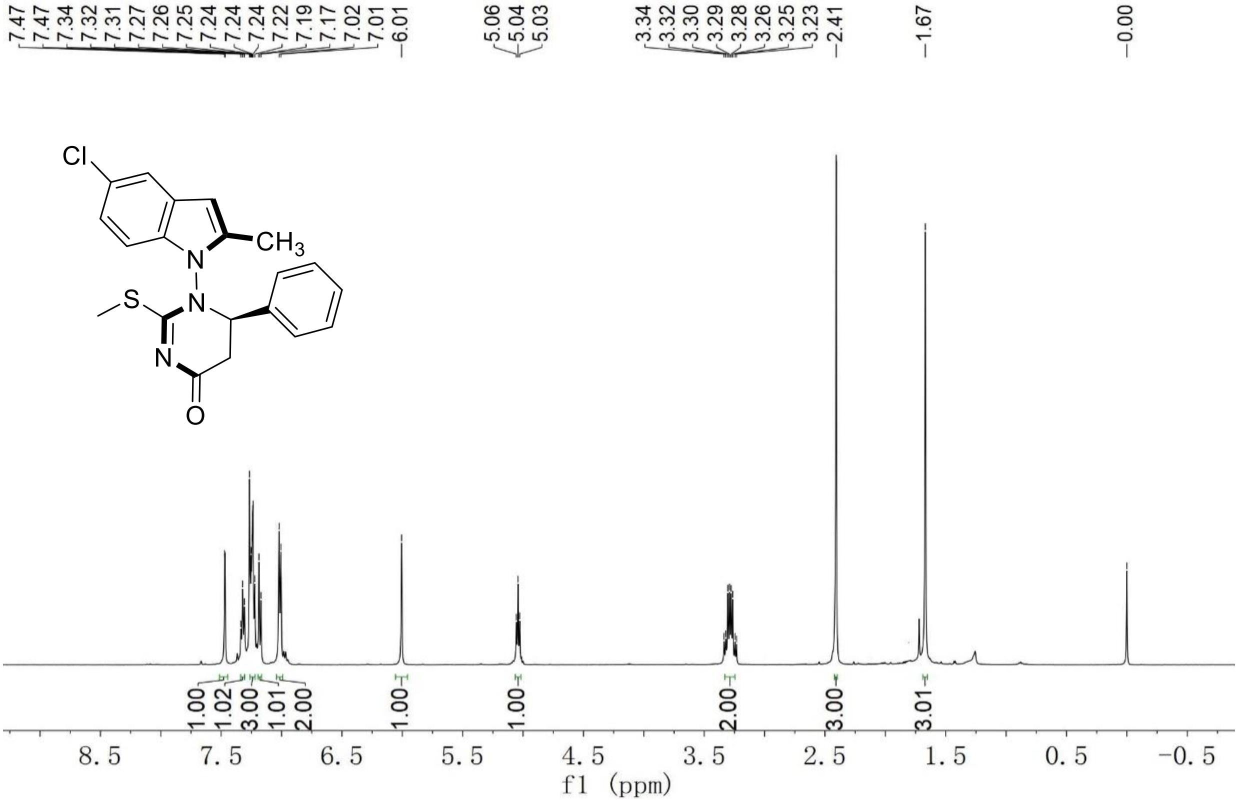

Supplementary Figure 106. **^{1}H NMR spectrum of compound 3o (CDCl$_3$, 500 MHz, 298 K)**

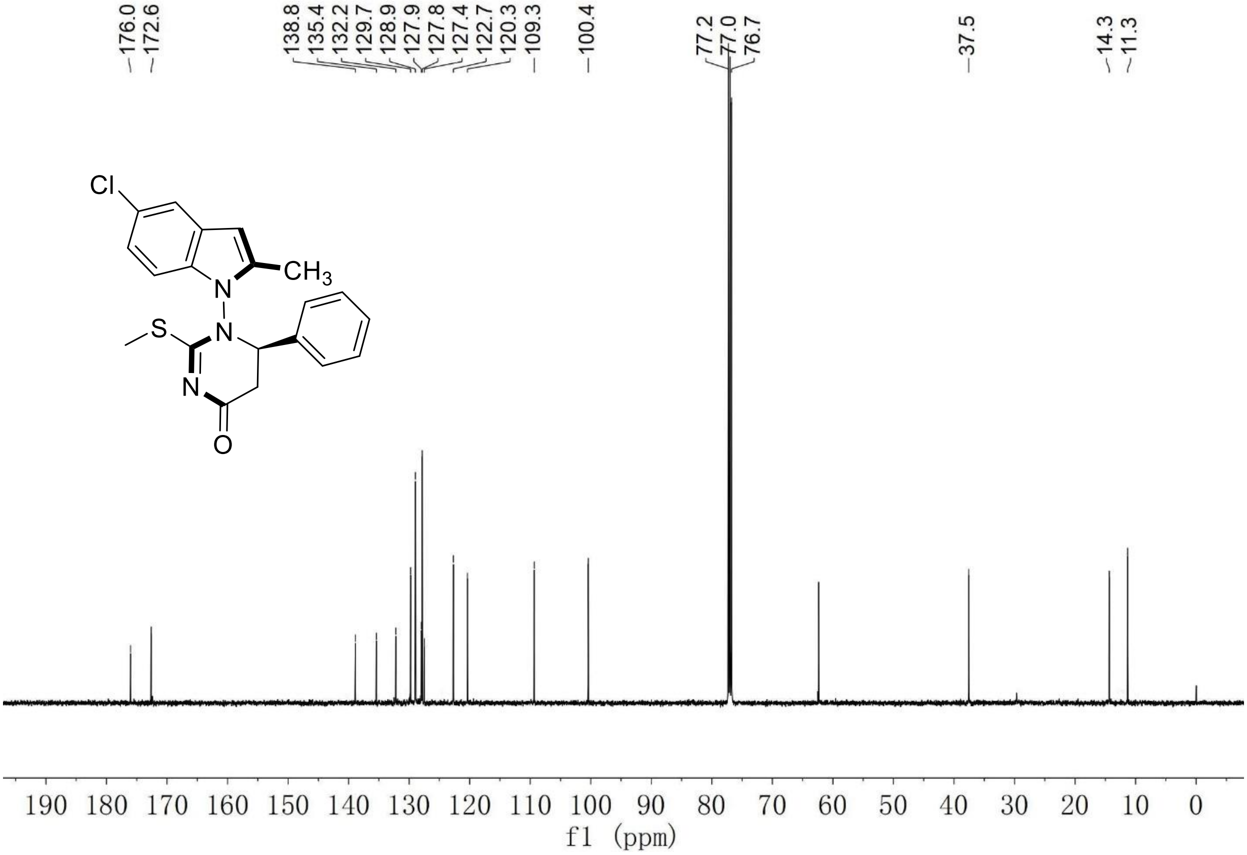

Supplementary Figure 107. **^{13}C NMR spectrum of compound 3o (CDCl$_3$, 126 MHz, 298 K)**

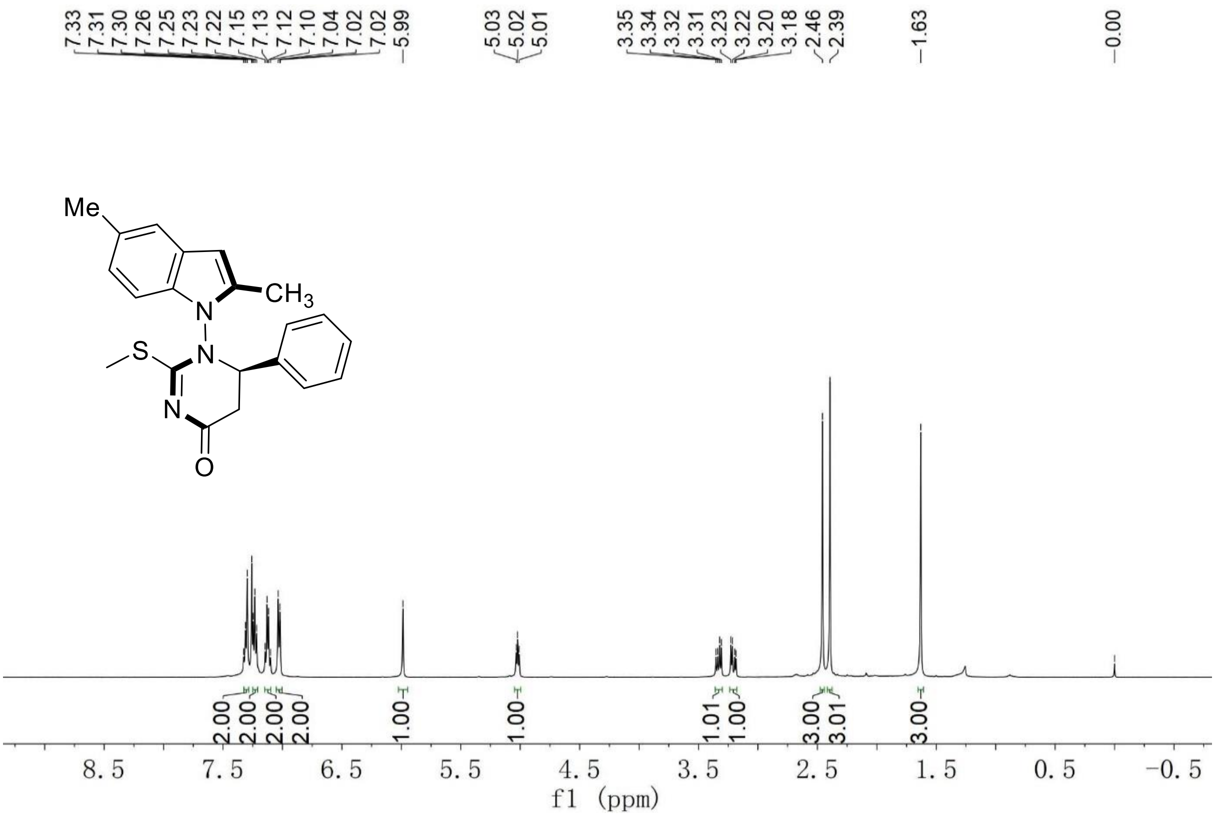

Supplementary Figure 108. ^{1}H NMR spectrum of compound 3p ($CDCl_3$, 500 MHz, 298 K)

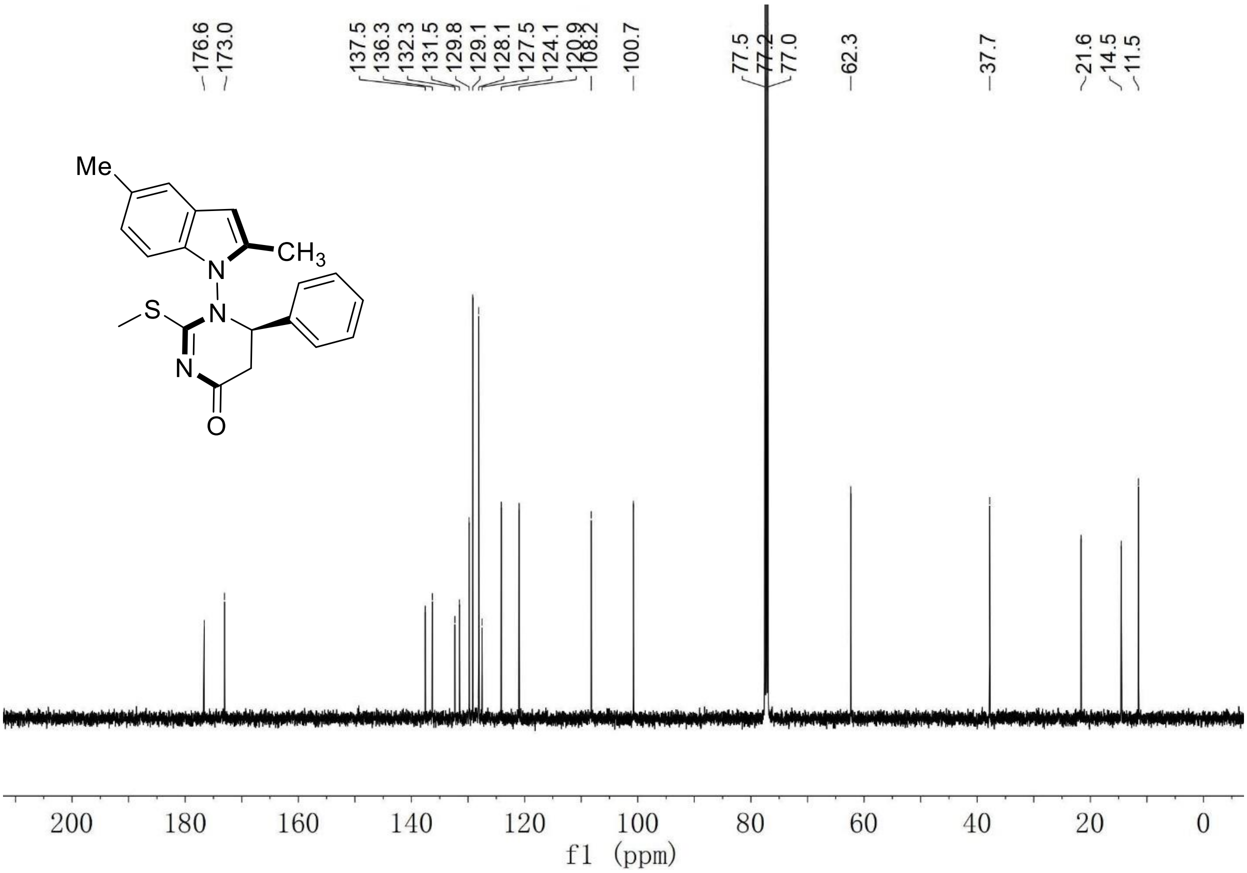

Supplementary Figure 109. ^{13}C NMR spectrum of compound 3p ($CDCl_3$, 126 MHz, 298 K)

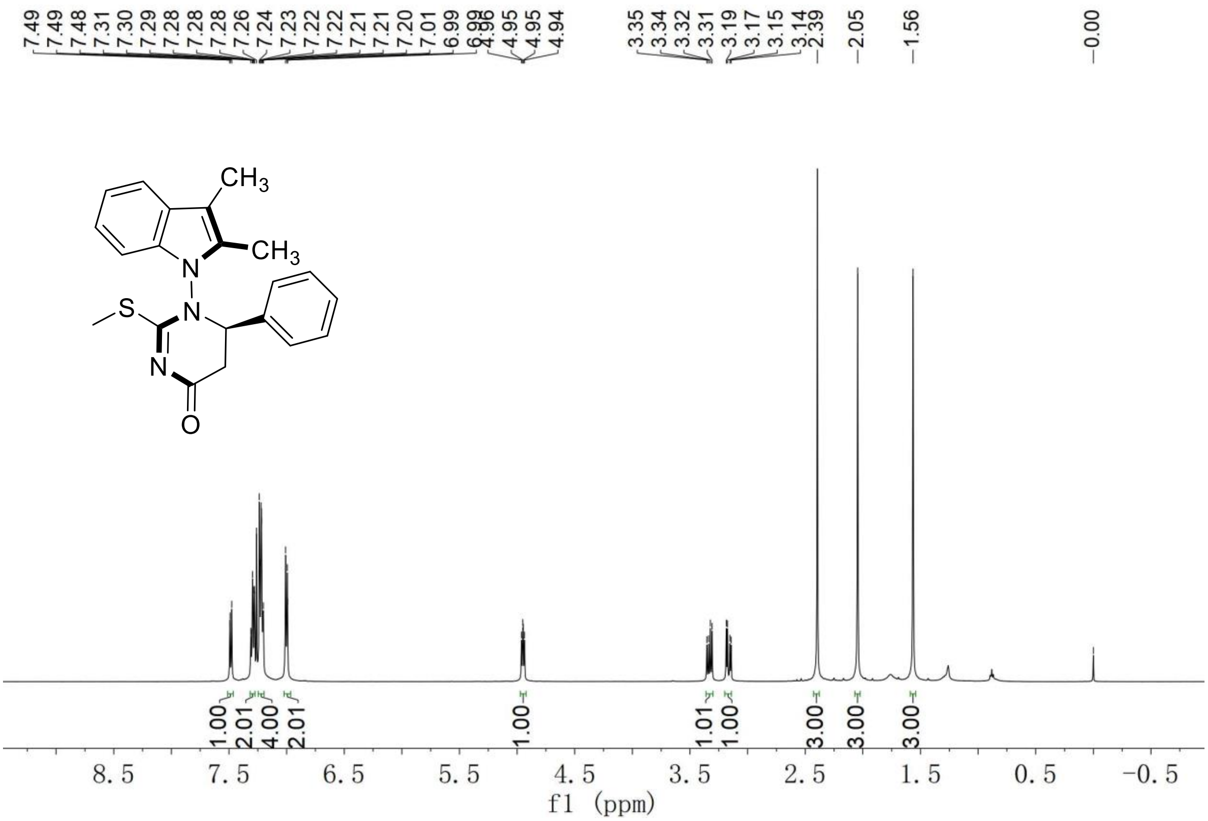

Supplementary Figure 110. ^{1}H NMR spectrum of compound 3q ($CDCl_3$, 500 MHz, 298 K)

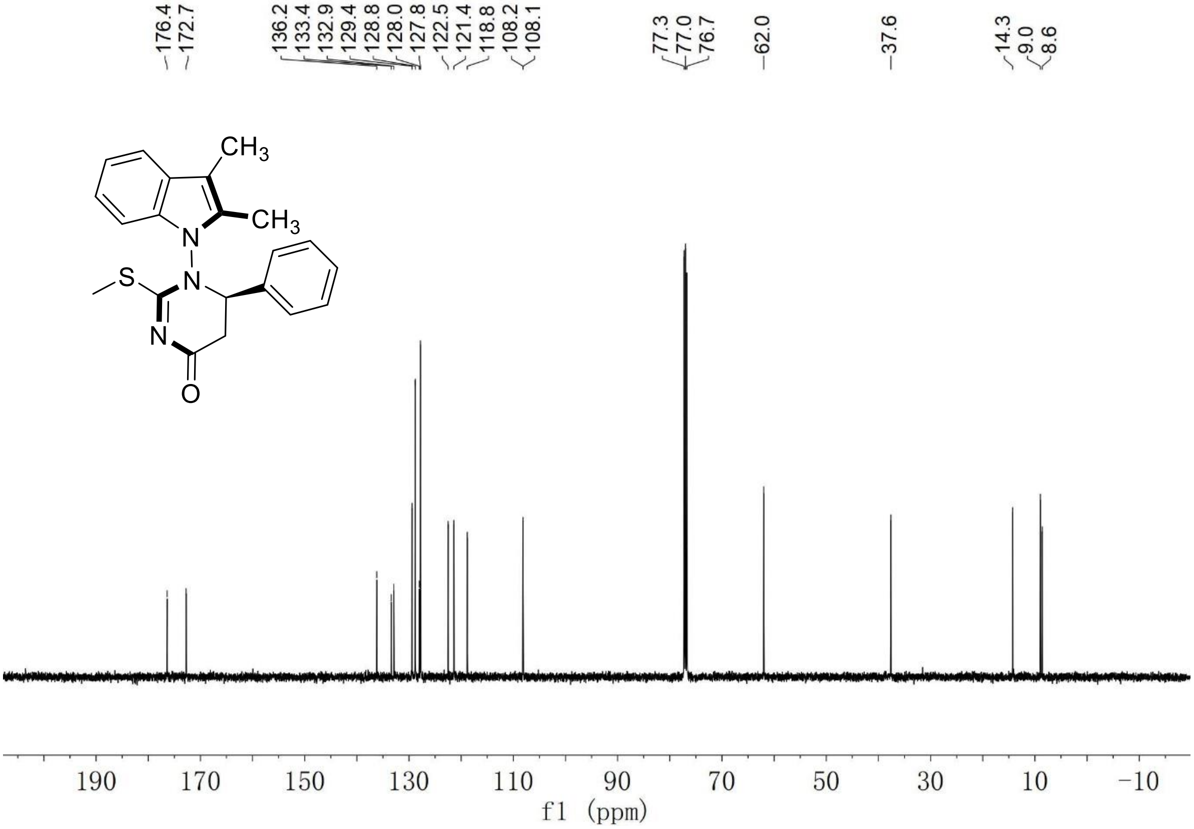

Supplementary Figure 111. ^{13}C NMR spectrum of compound 3q ($CDCl_3$, 126 MHz, 298 K)

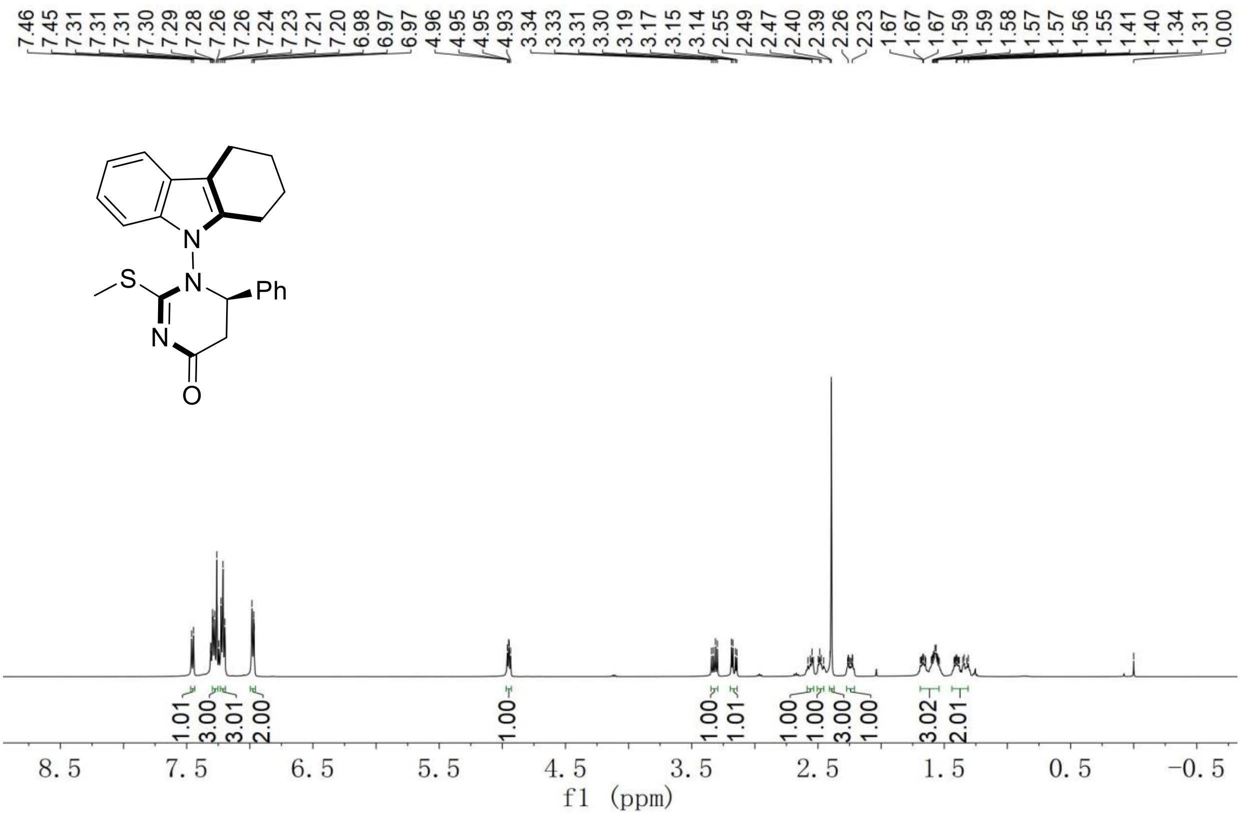

Supplementary Figure 112. ^{1}H NMR spectrum of compound 3r ($CDCl_3$, 500 MHz, 298 K)

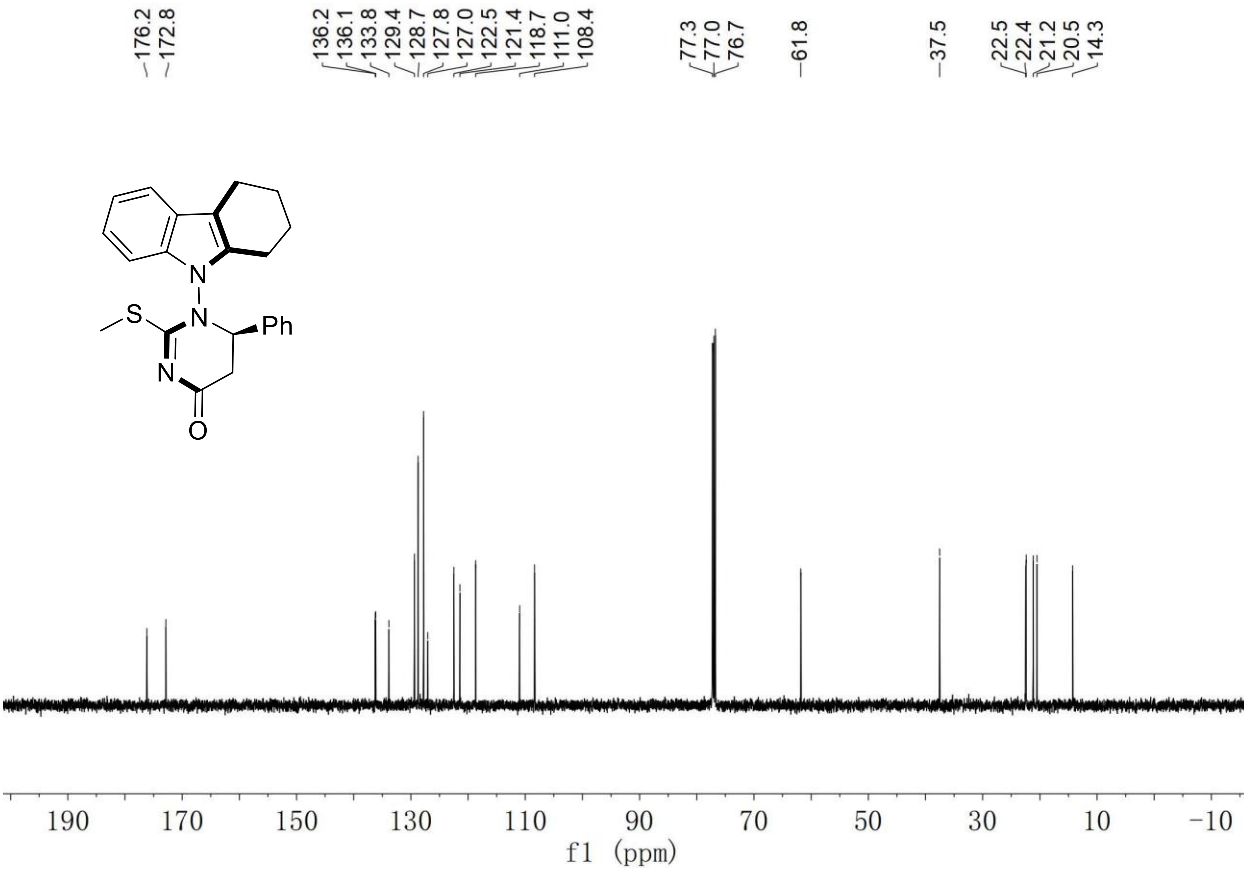

Supplementary Figure 113. ^{13}C NMR spectrum of compound 3r ($CDCl_3$, 126 MHz, 298 K)

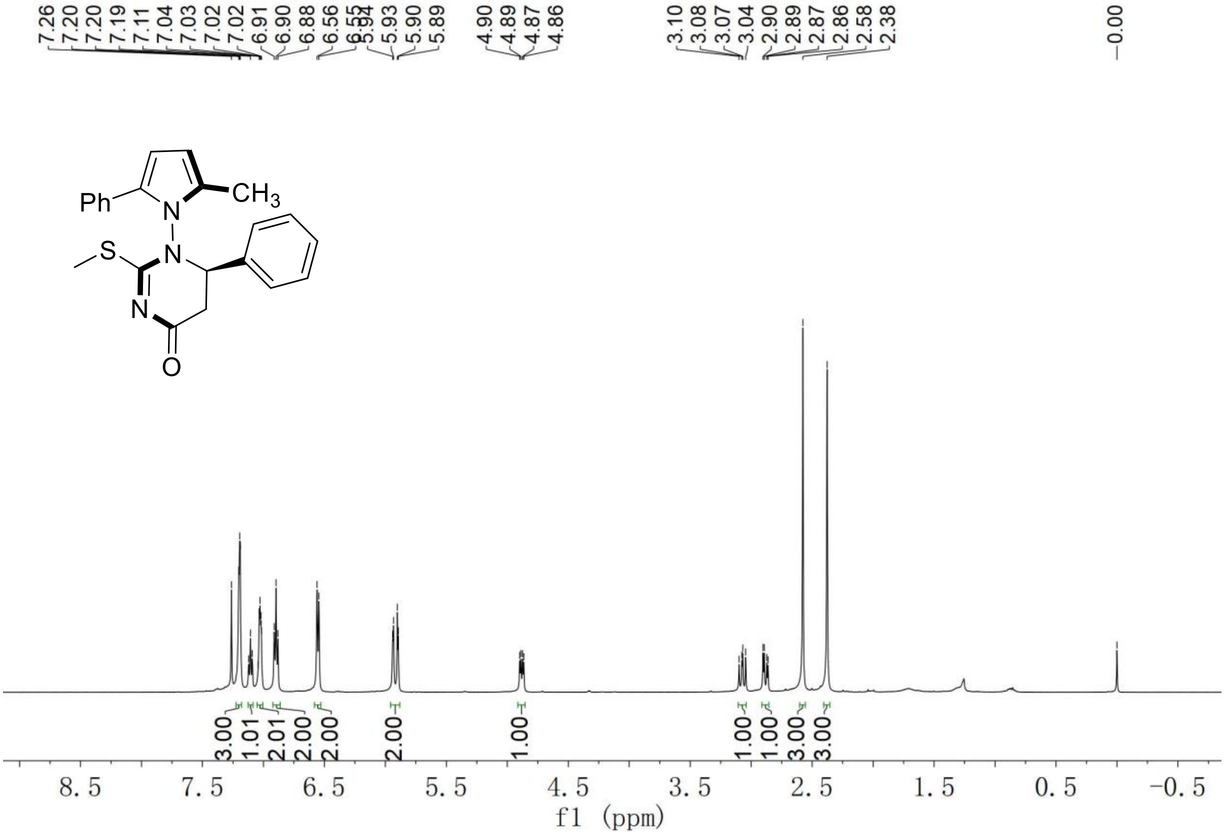

Supplementary Figure 114. ^{1}H NMR spectrum of compound 3s ($CDCl_3$, 500 MHz, 298 K)

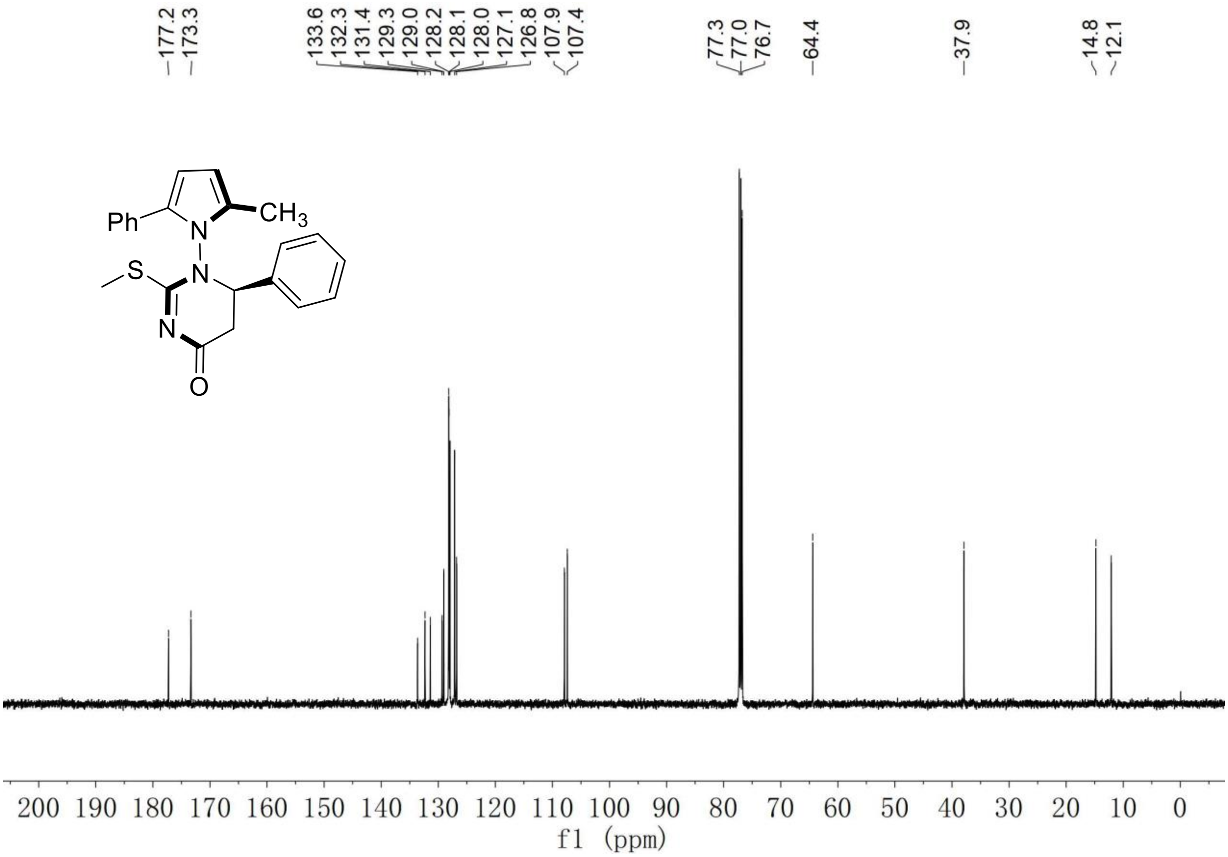

Supplementary Figure 115. ^{13}C NMR spectrum of compound 3s ($CDCl_3$, 126 MHz, 298 K)

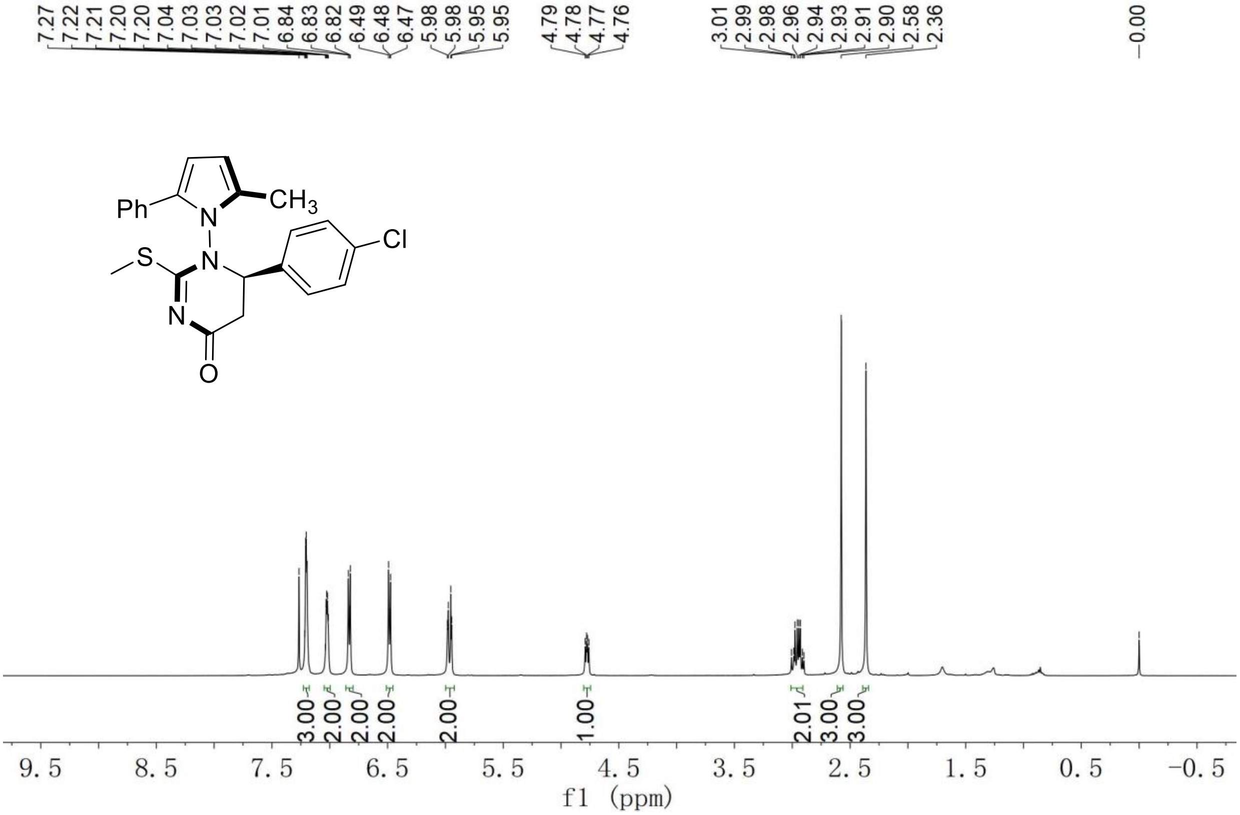

Supplementary Figure 116. ^{1}H NMR spectrum of compound 3t ($CDCl_3$, 500 MHz, 298 K)

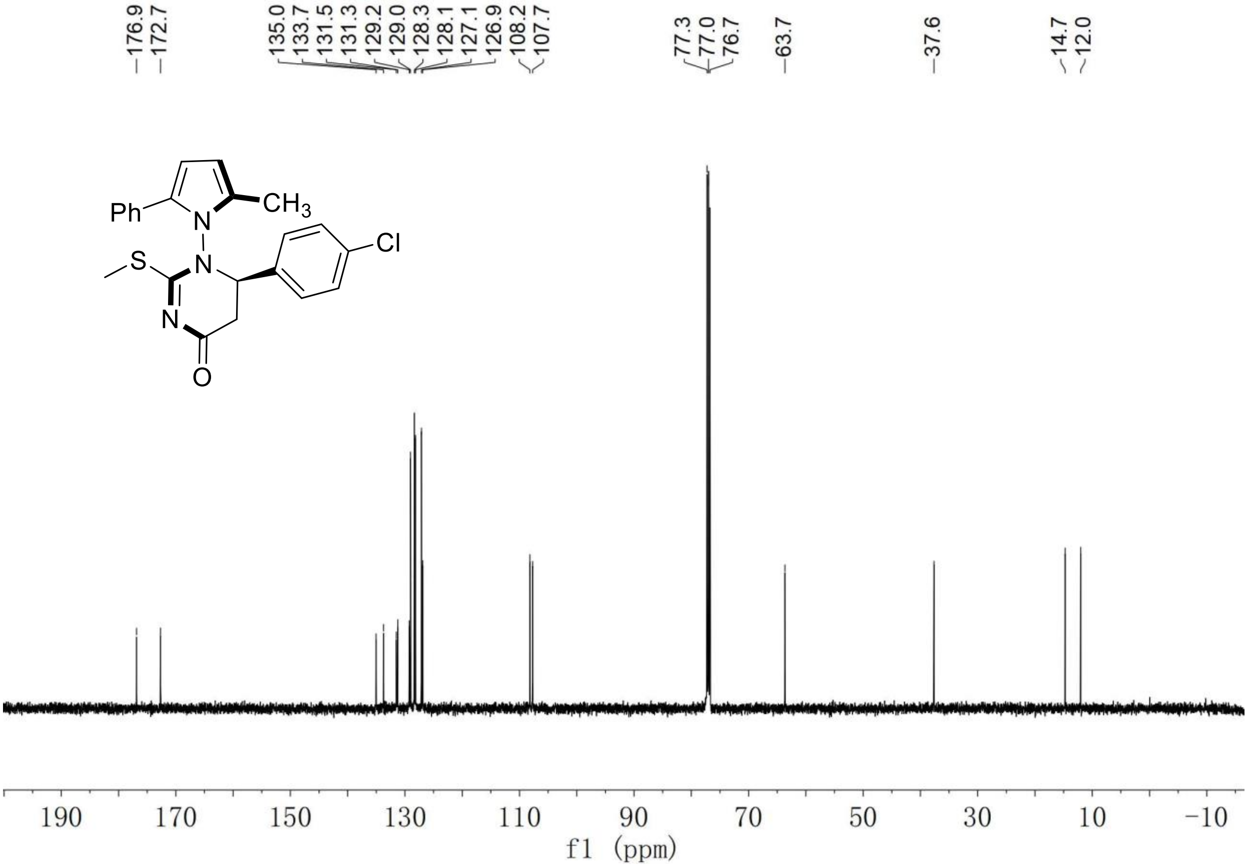

Supplementary Figure 117. ^{13}C NMR spectrum of compound 3t ($CDCl_3$, 126 MHz, 298 K)

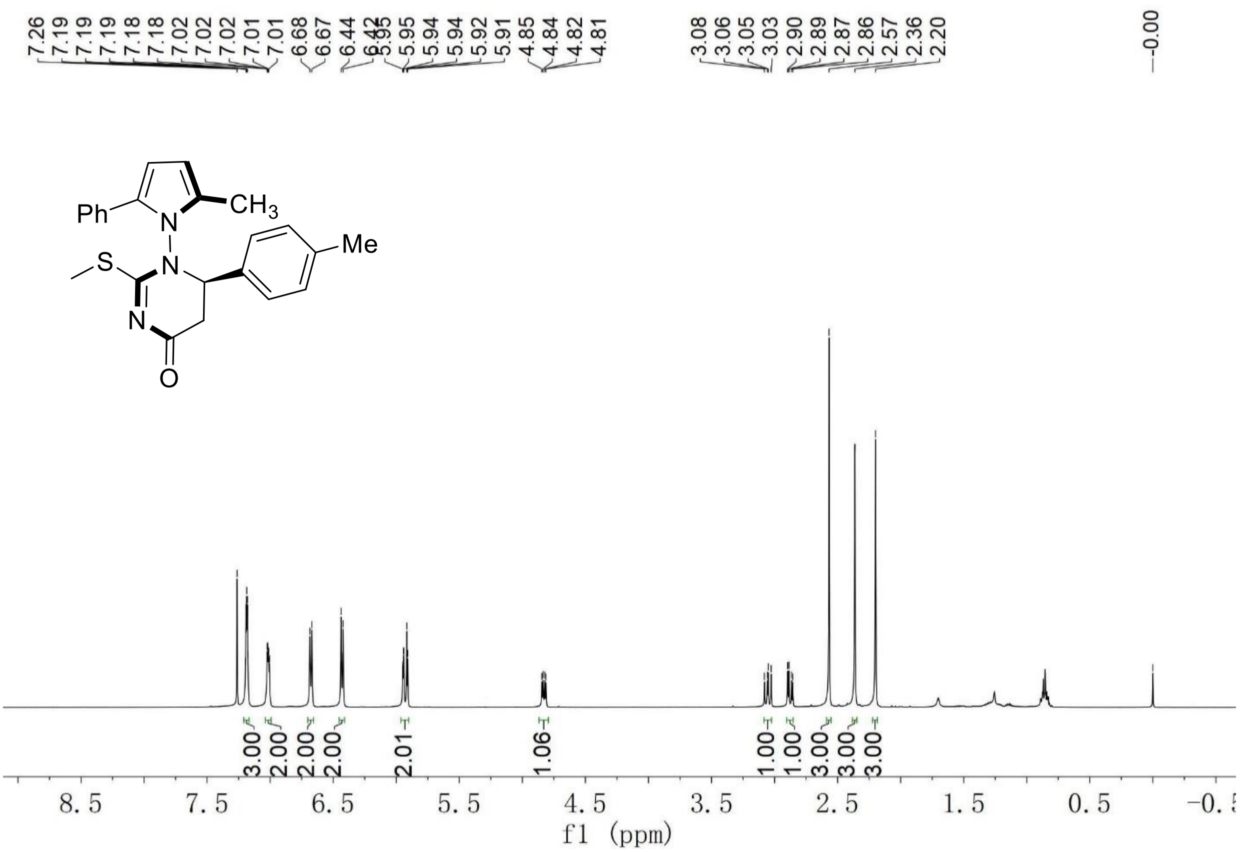

Supplementary Figure 118. ^{1}H **NMR spectrum of compound 3u ($CDCl_3$, 500 MHz, 298 K)**

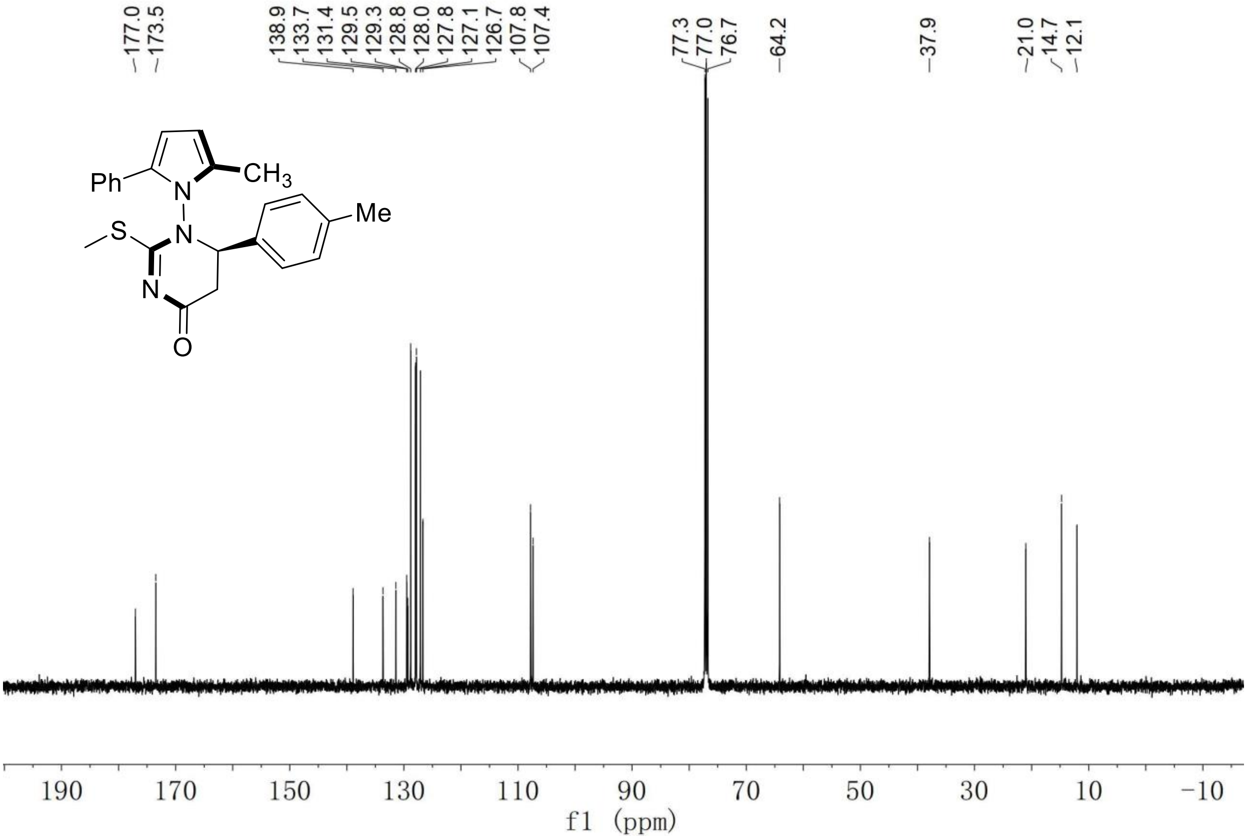

Supplementary Figure 119. ^{13}C **NMR spectrum of compound 3u ($CDCl_3$, 126 MHz, 298 K)**

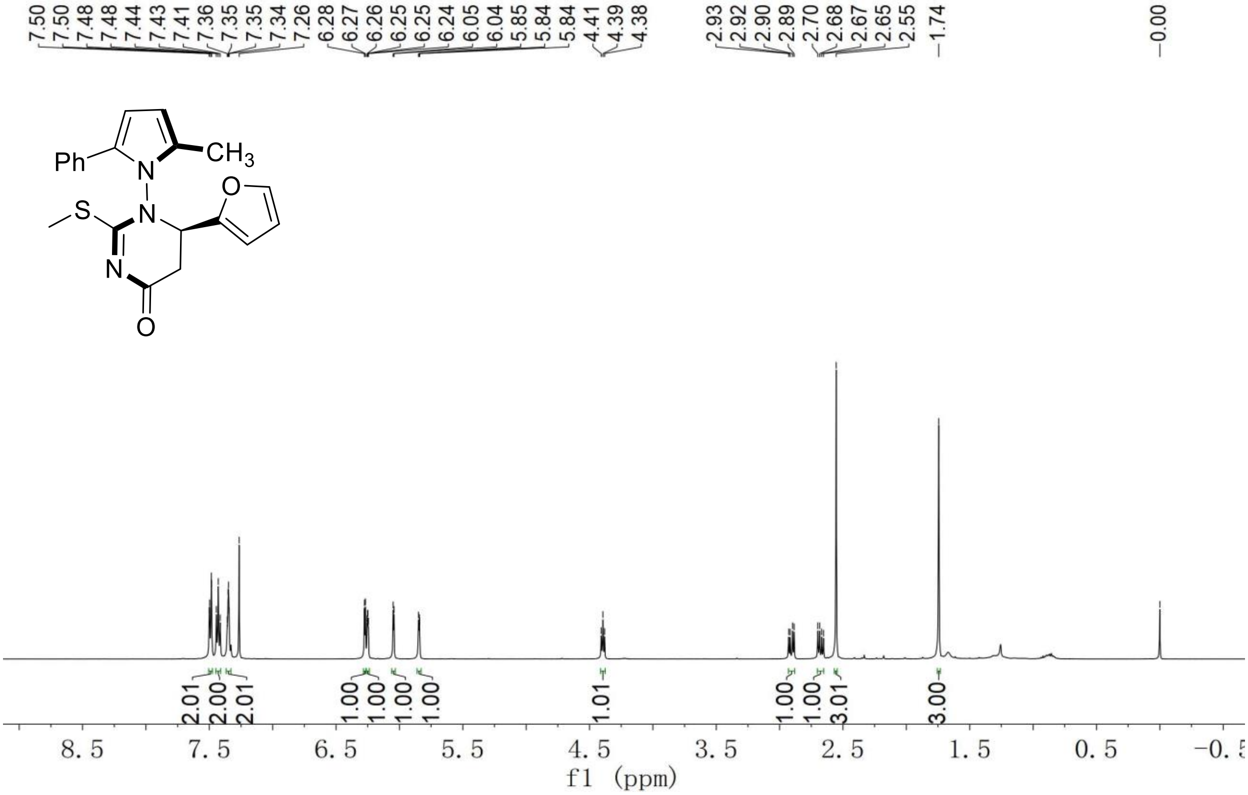

Supplementary Figure 120. ^{1}H **NMR spectrum of compound 3v ($CDCl_3$, 500 MHz, 298 K)**

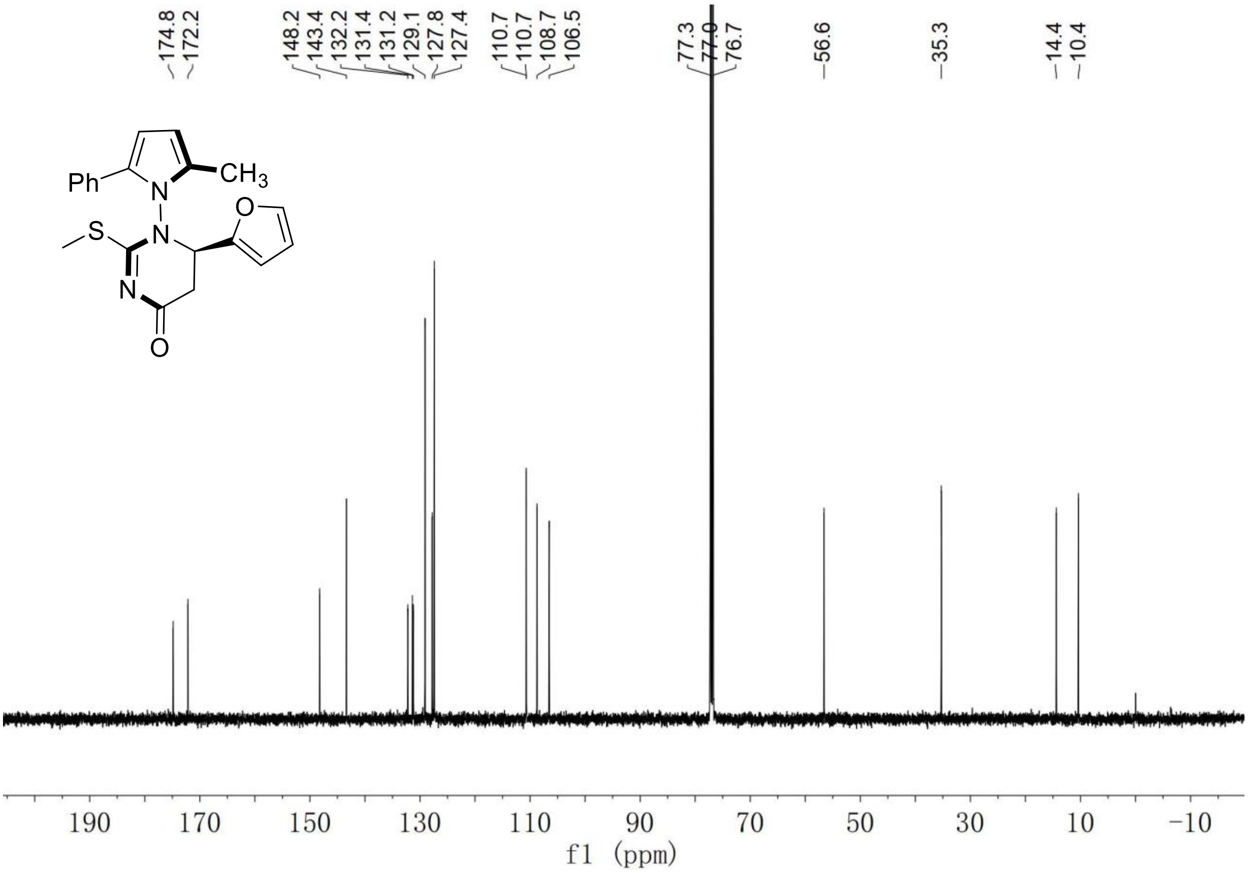

Supplementary Figure 121. ^{13}C **NMR spectrum of compound 3v ($CDCl_3$, 126 MHz, 298 K)**

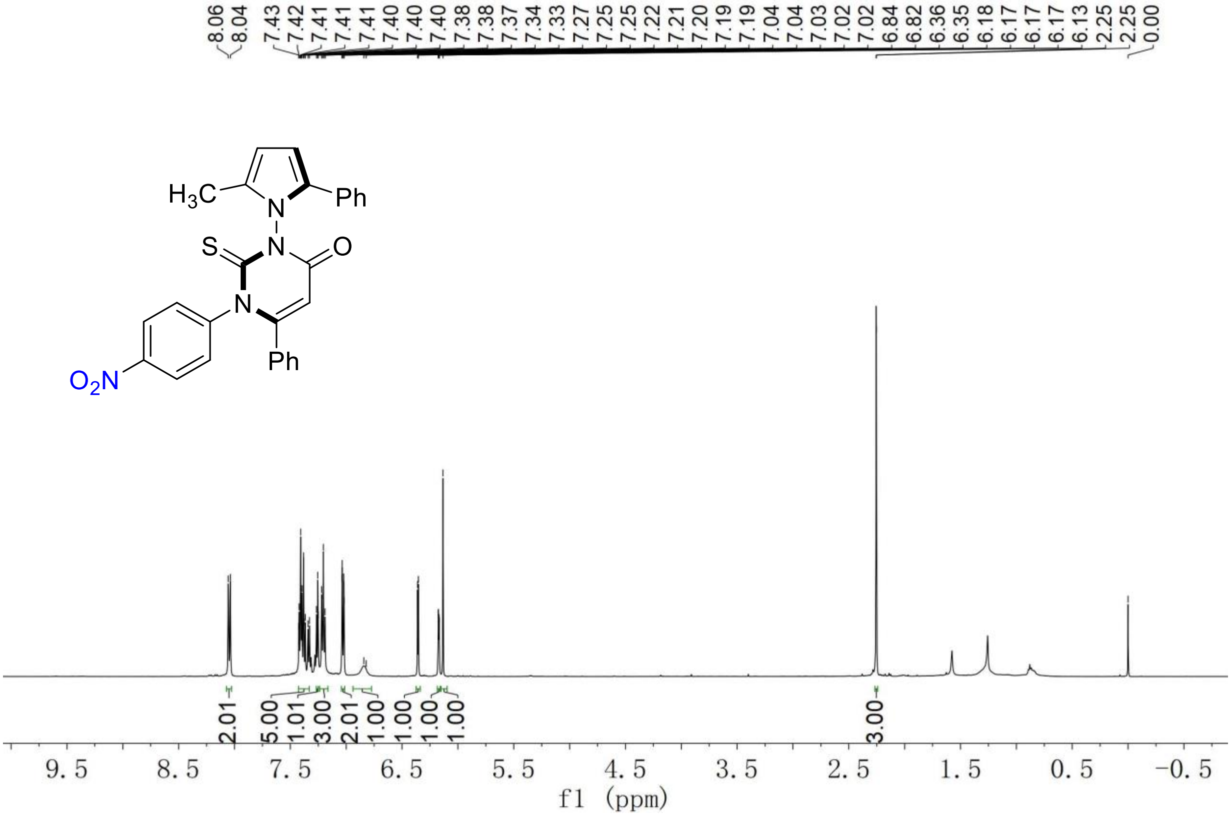

Supplementary Figure 122. **^{1}H NMR spectrum of compound 7a ($CDCl_3$, 500 MHz, 298 K)**

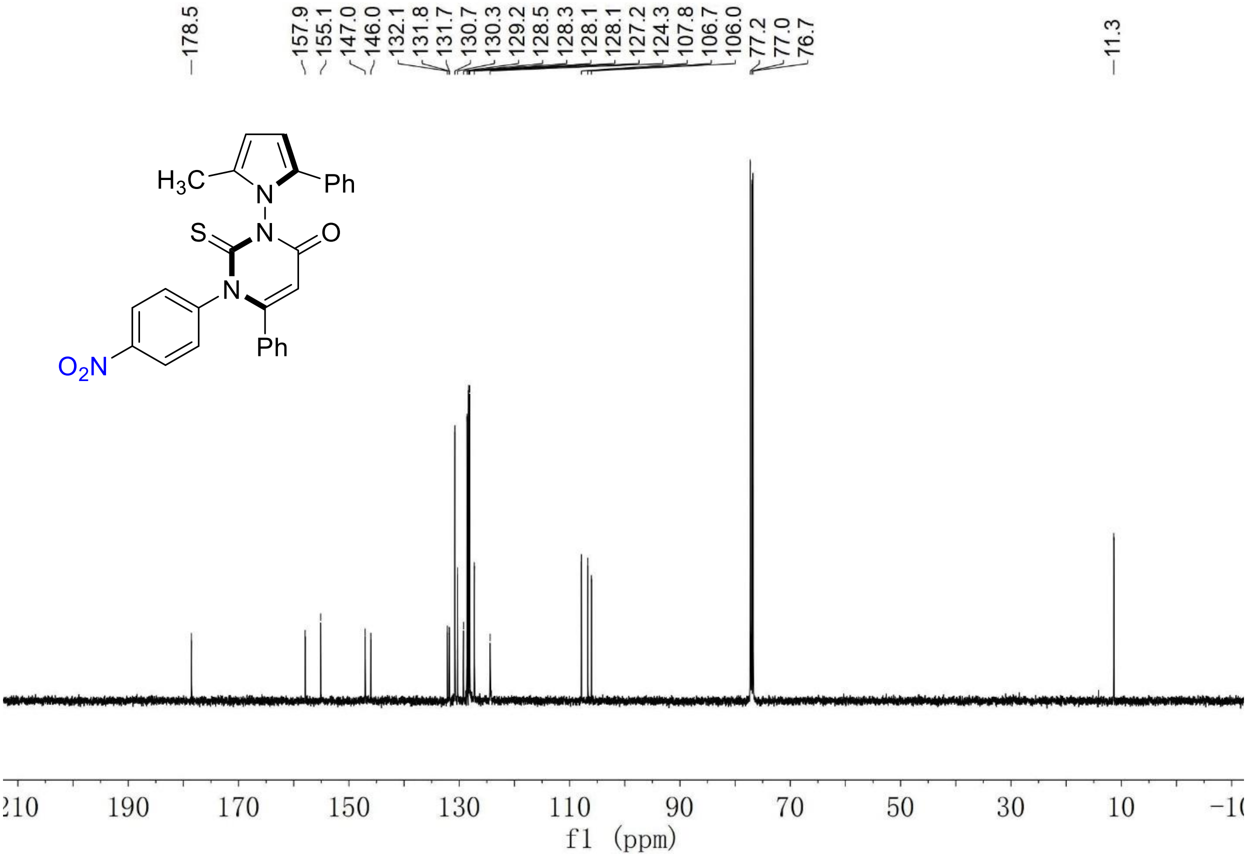

Supplementary Figure 123. **^{13}C NMR spectrum of compound 7a ($CDCl_3$, 126 MHz, 298 K)**

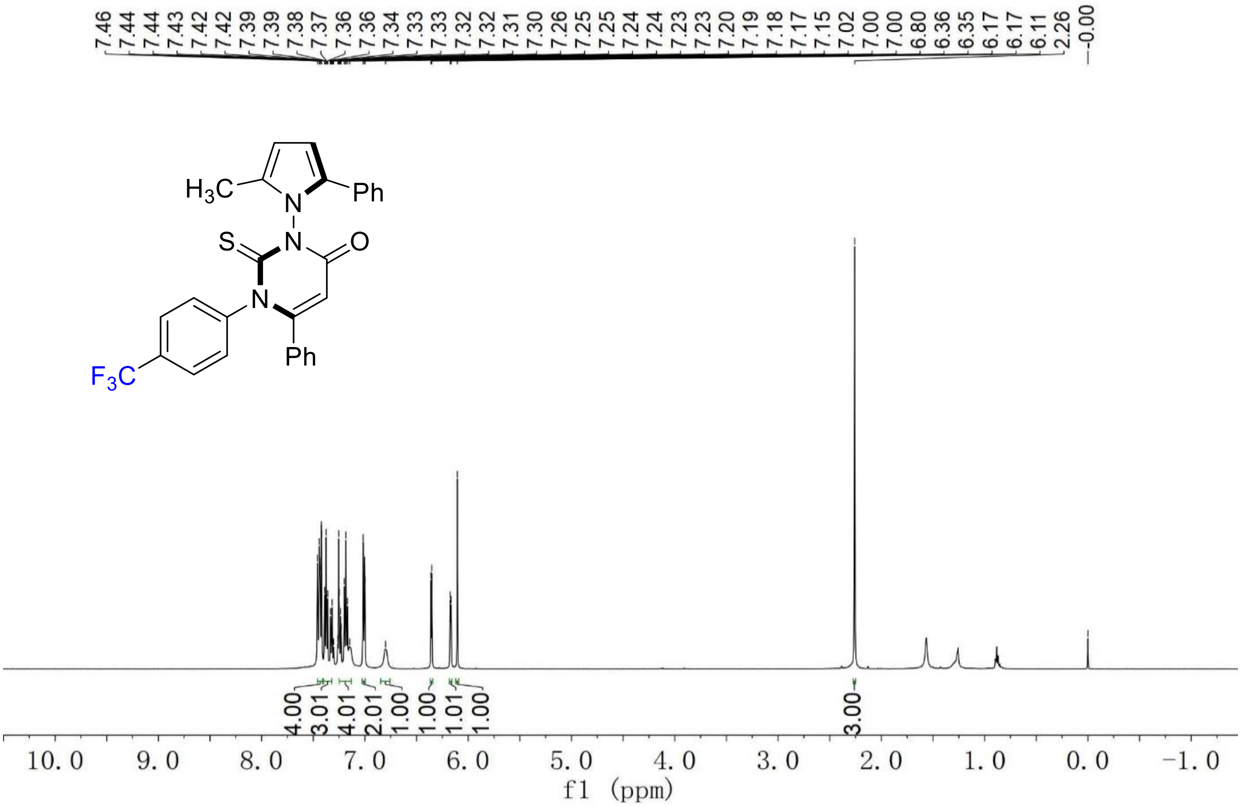

Supplementary Figure 124. ^{1}H NMR spectrum of compound 7b ($CDCl_3$, 500 MHz, 298 K)

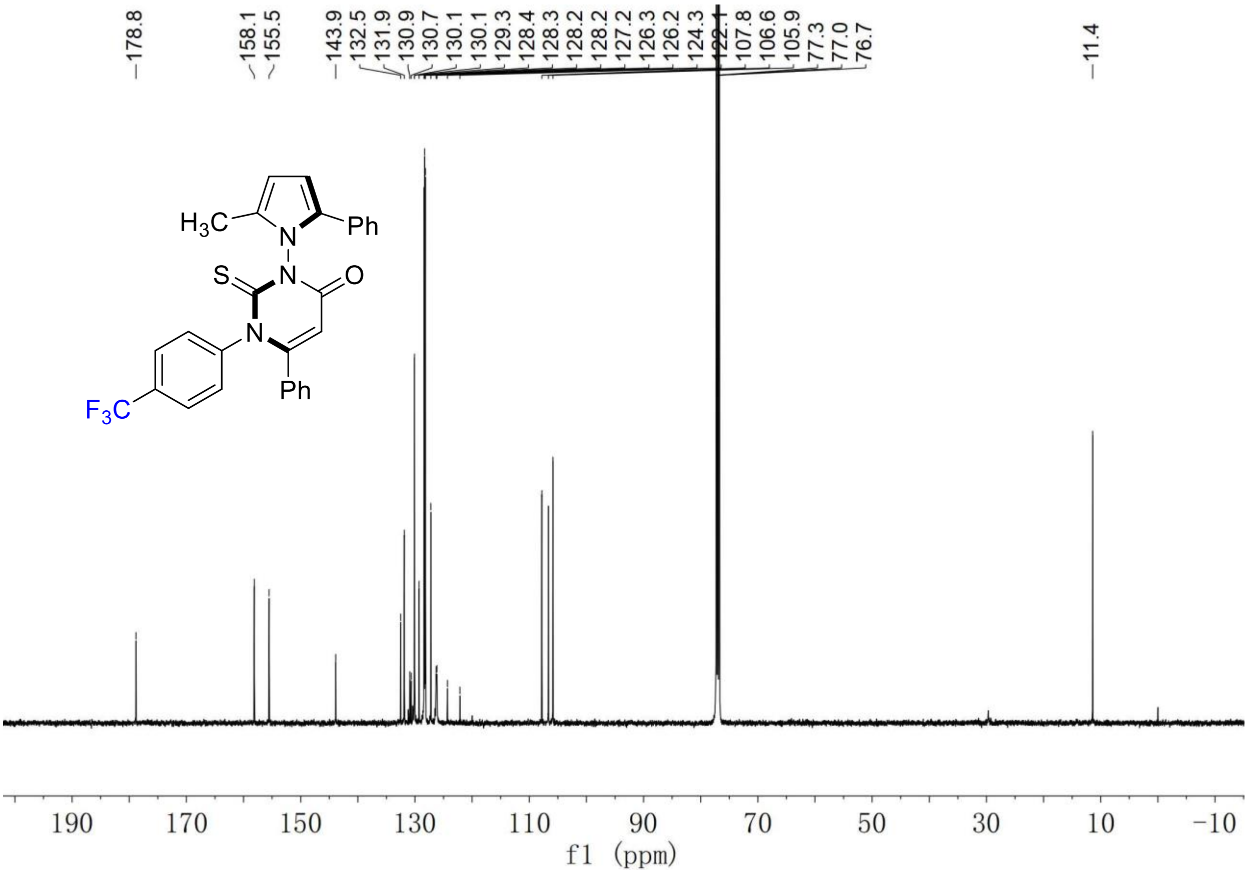

Supplementary Figure 125. ^{13}C NMR spectrum of compound 7b ($CDCl_3$, 126 MHz, 298 K)

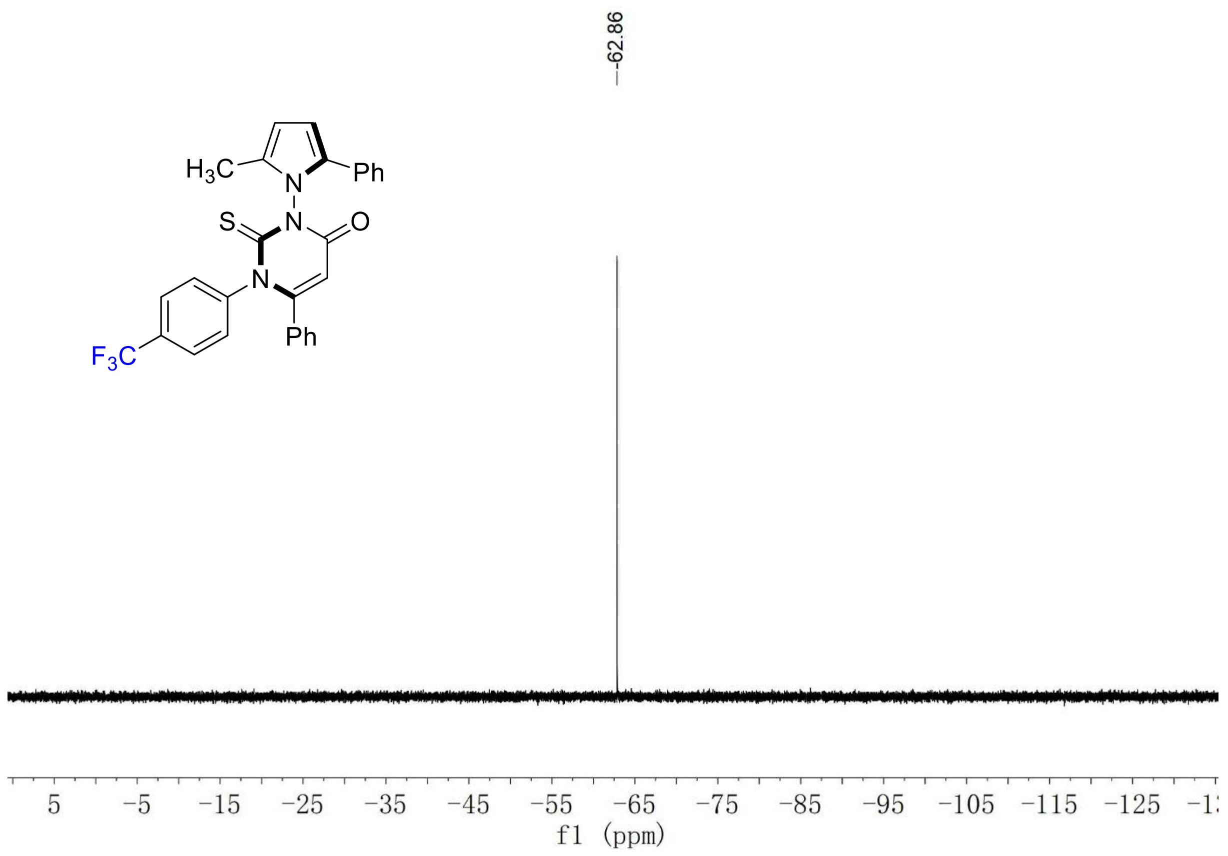

Supplementary Figure 126. ^{19}F NMR spectrum of compound 7b ($CDCl_3$, 471 MHz, 298 K)

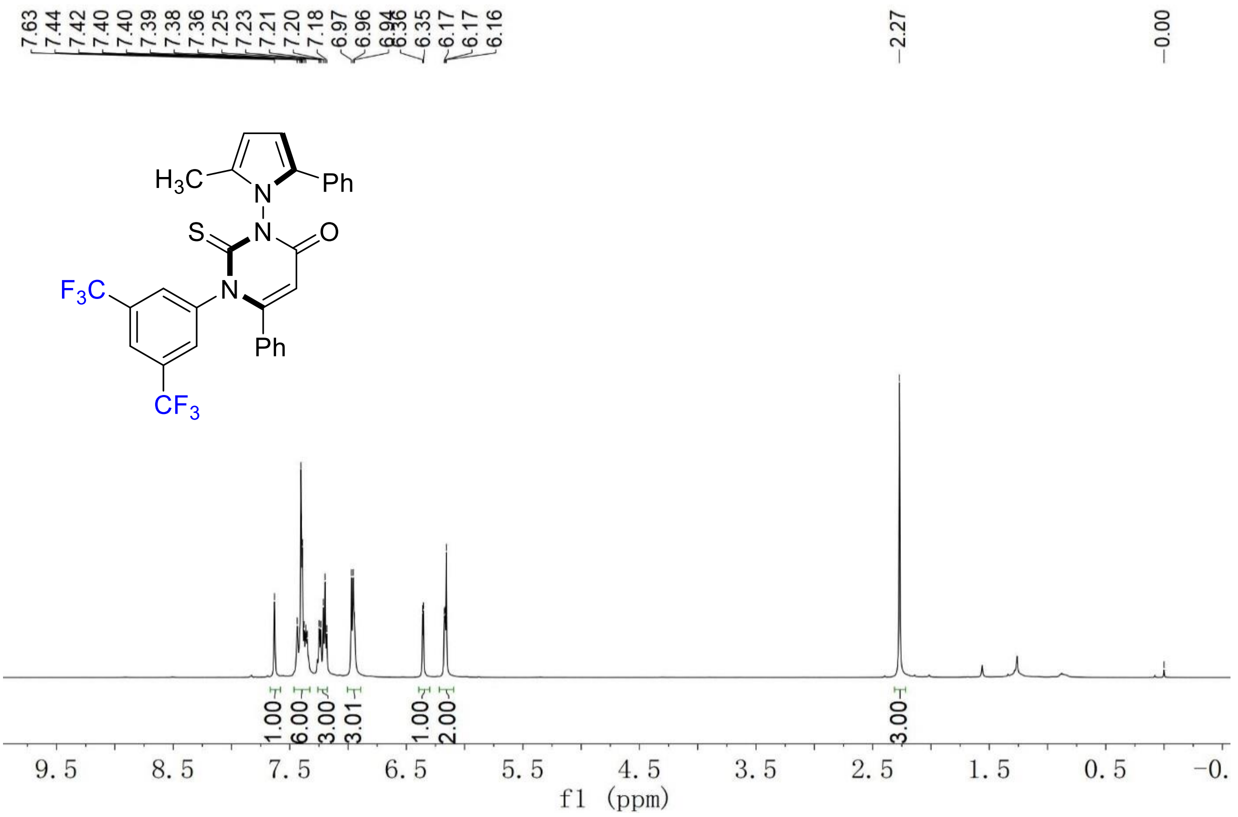

Supplementary Figure 127. ^{1}H NMR spectrum of compound 7c ($CDCl_3$, 500 MHz, 298 K)

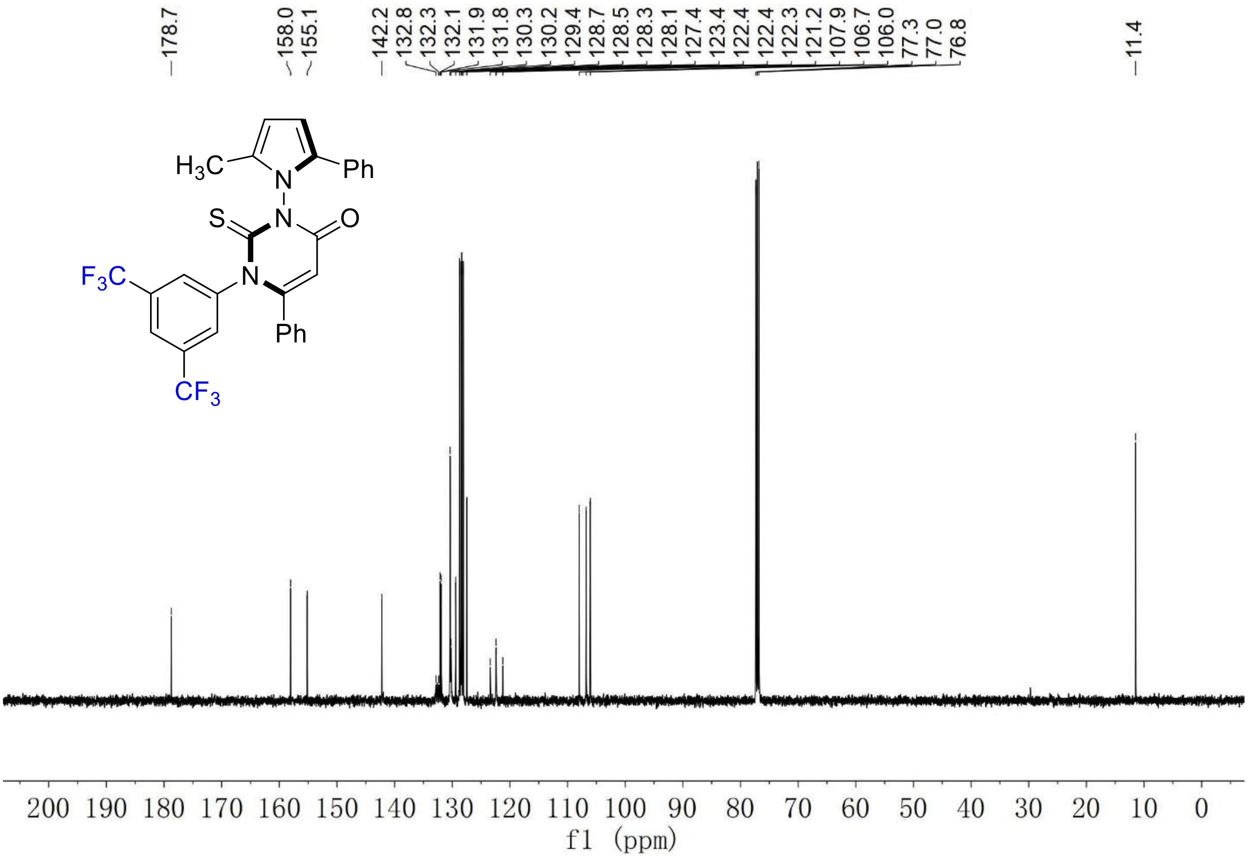

Supplementary Figure 128. ^{13}C NMR spectrum of compound 7c ($CDCl_3$, 126 MHz, 298 K)

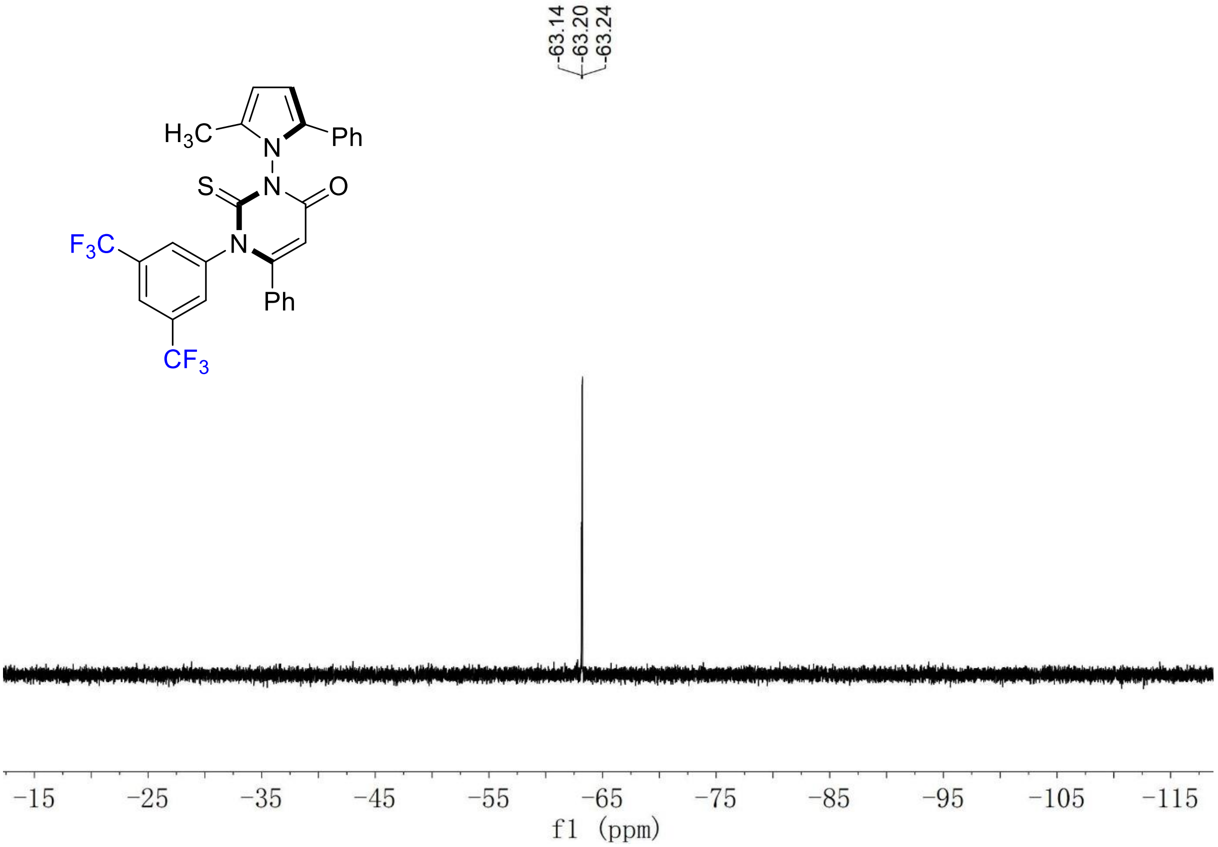

Supplementary Figure 129. ^{19}F NMR spectrum of compound 7c ($CDCl_3$, 126 MHz, 298 K)

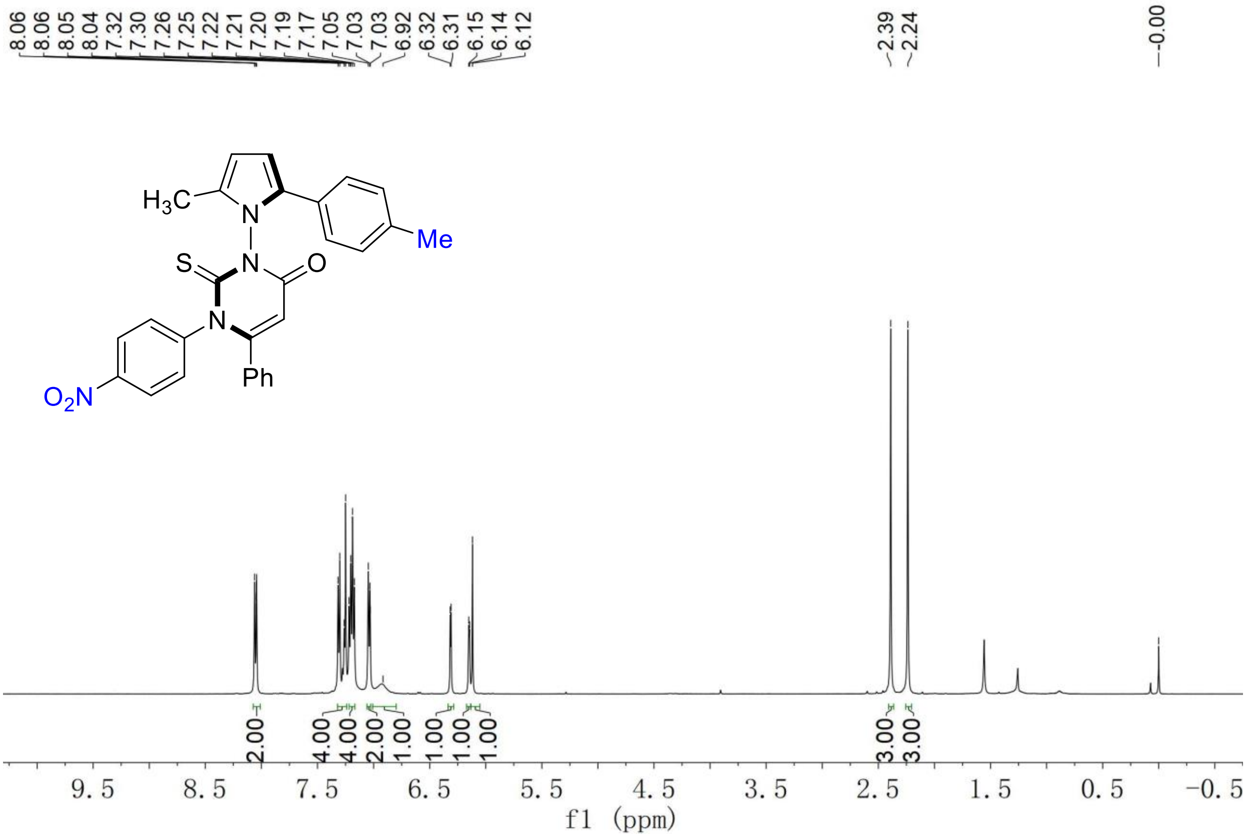

Supplementary Figure 130. ^{1}H NMR spectrum of compound 7d ($CDCl_3$, 500 MHz, 298 K)

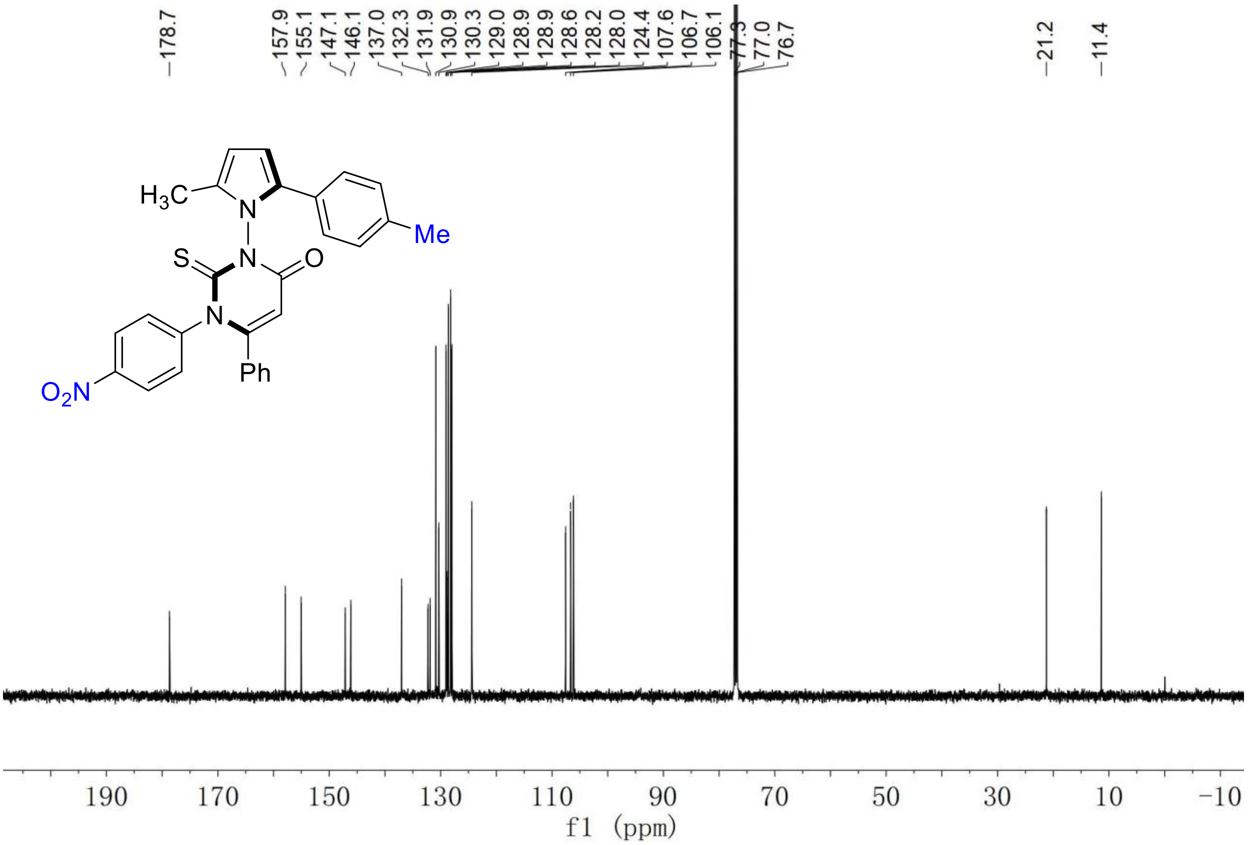

Supplementary Figure 131. ^{13}C NMR spectrum of compound 7d ($CDCl_3$, 126 MHz, 298 K)

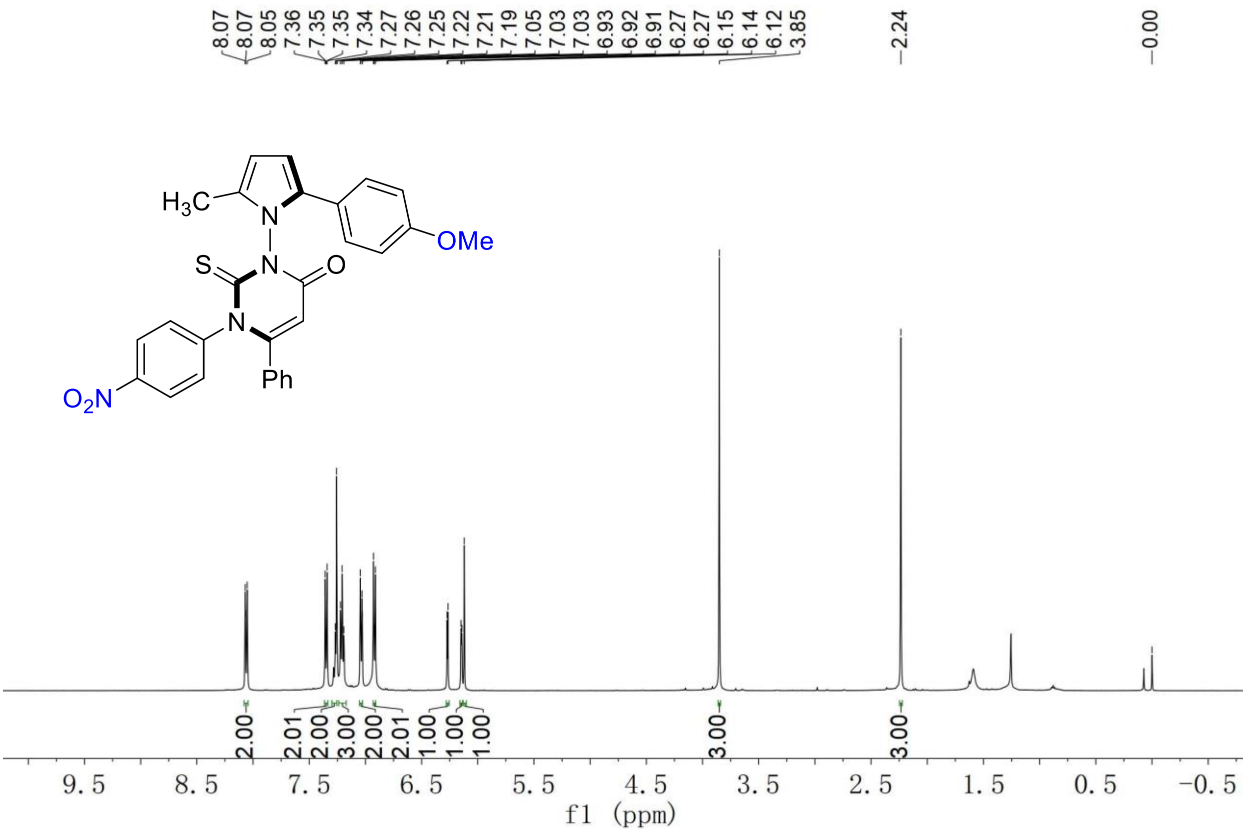

Supplementary Figure 132. ^{1}H NMR spectrum of compound 7e ($CDCl_3$, 500 MHz, 298 K)

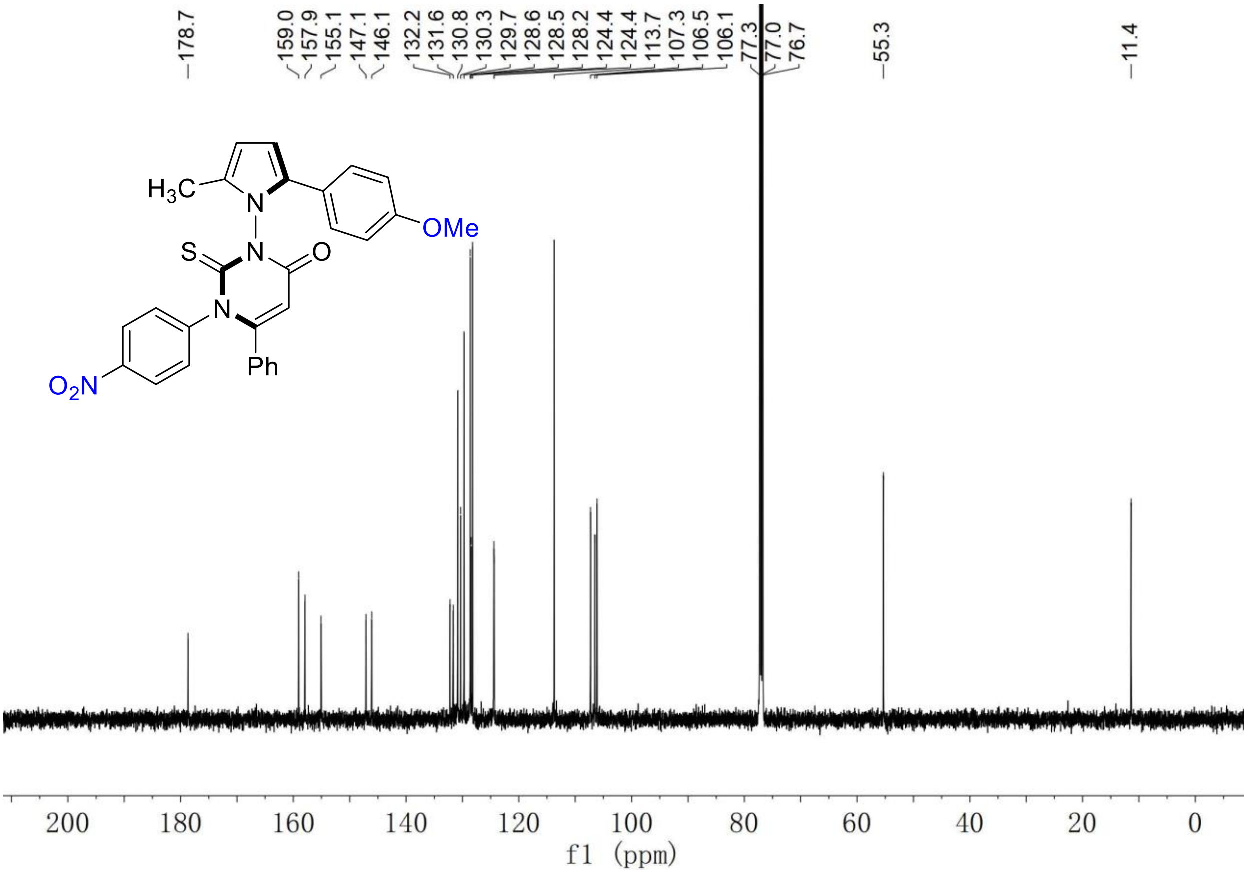

Supplementary Figure 133. ^{13}C NMR spectrum of compound 7e ($CDCl_3$, 126 MHz, 298 K)

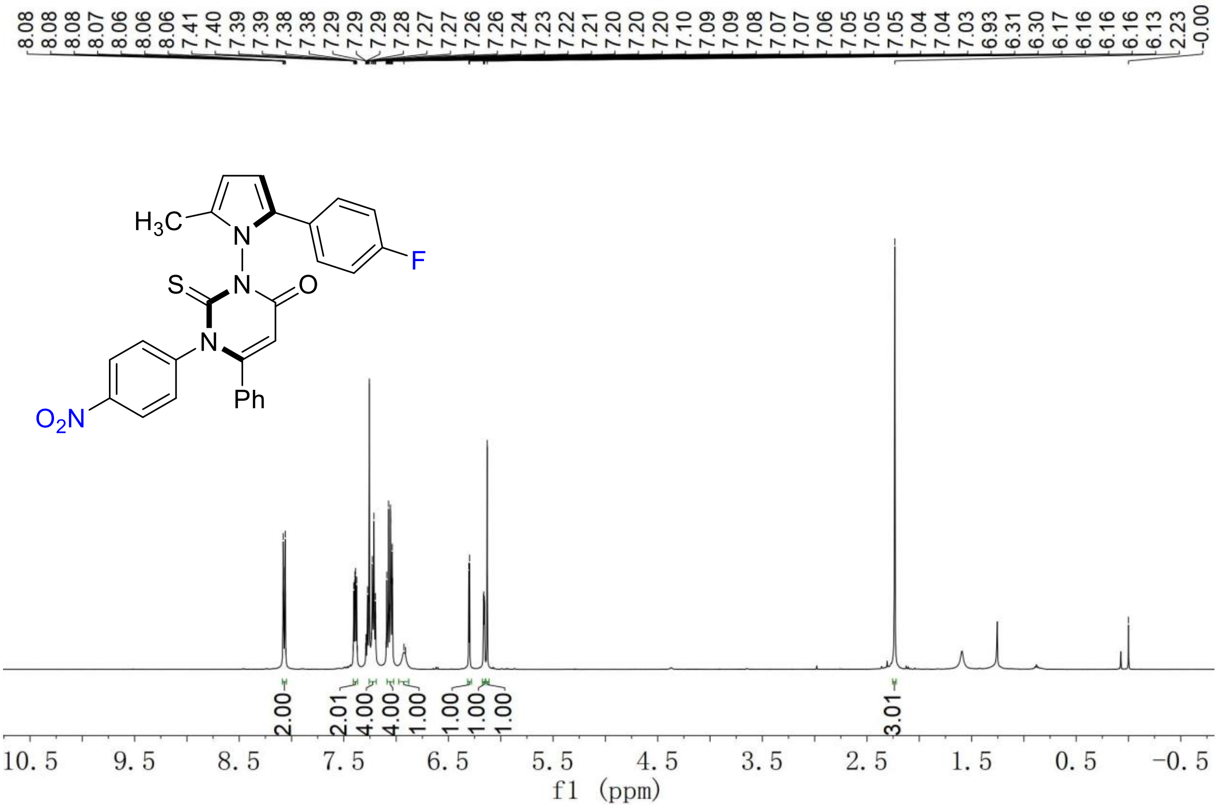

Supplementary Figure 134. ^{1}H NMR spectrum of compound 7f ($CDCl_3$, 500 MHz, 298 K)

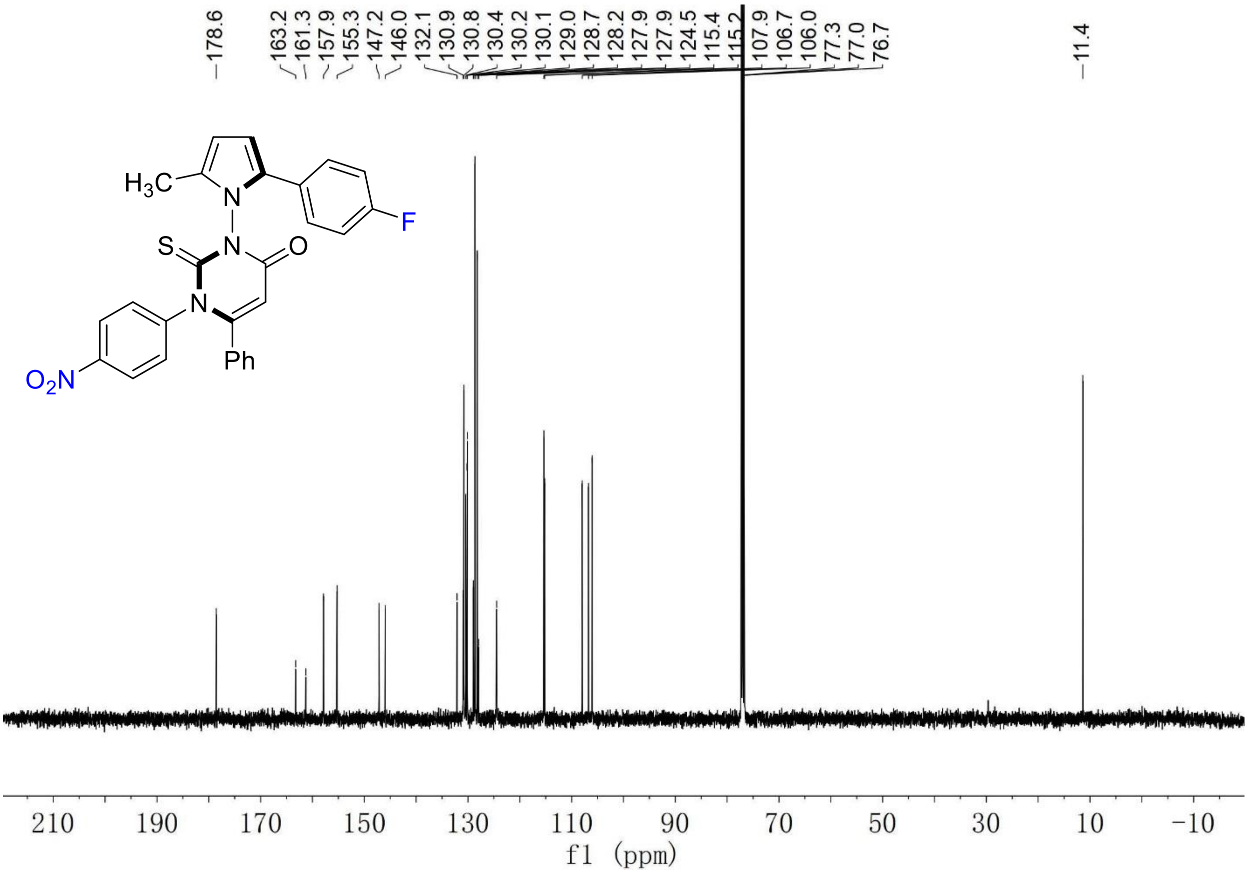

Supplementary Figure 135. ^{13}C NMR spectrum of compound 7f ($CDCl_3$, 126 MHz, 298 K)

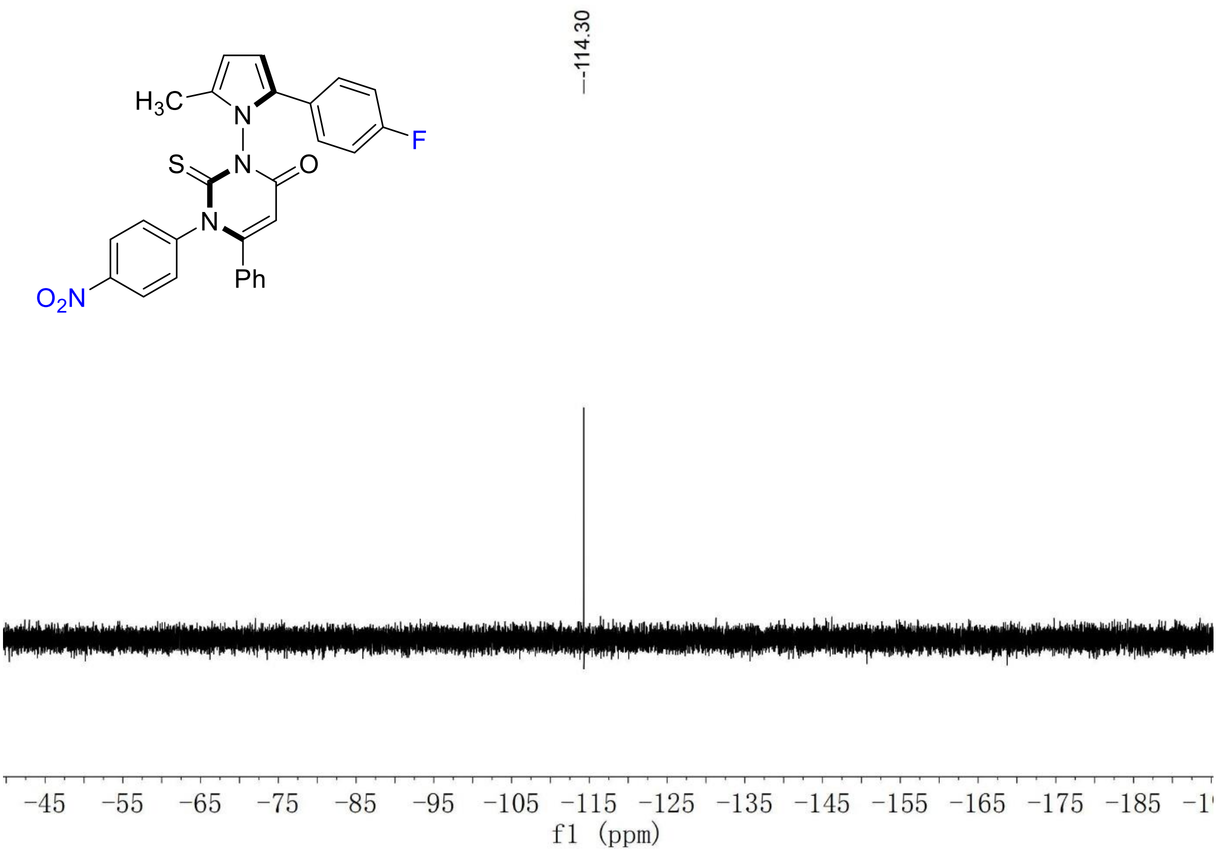

Supplementary Figure 136. ^{19}F NMR spectrum of compound 7f ($CDCl_3$, 471 MHz, 298 K)

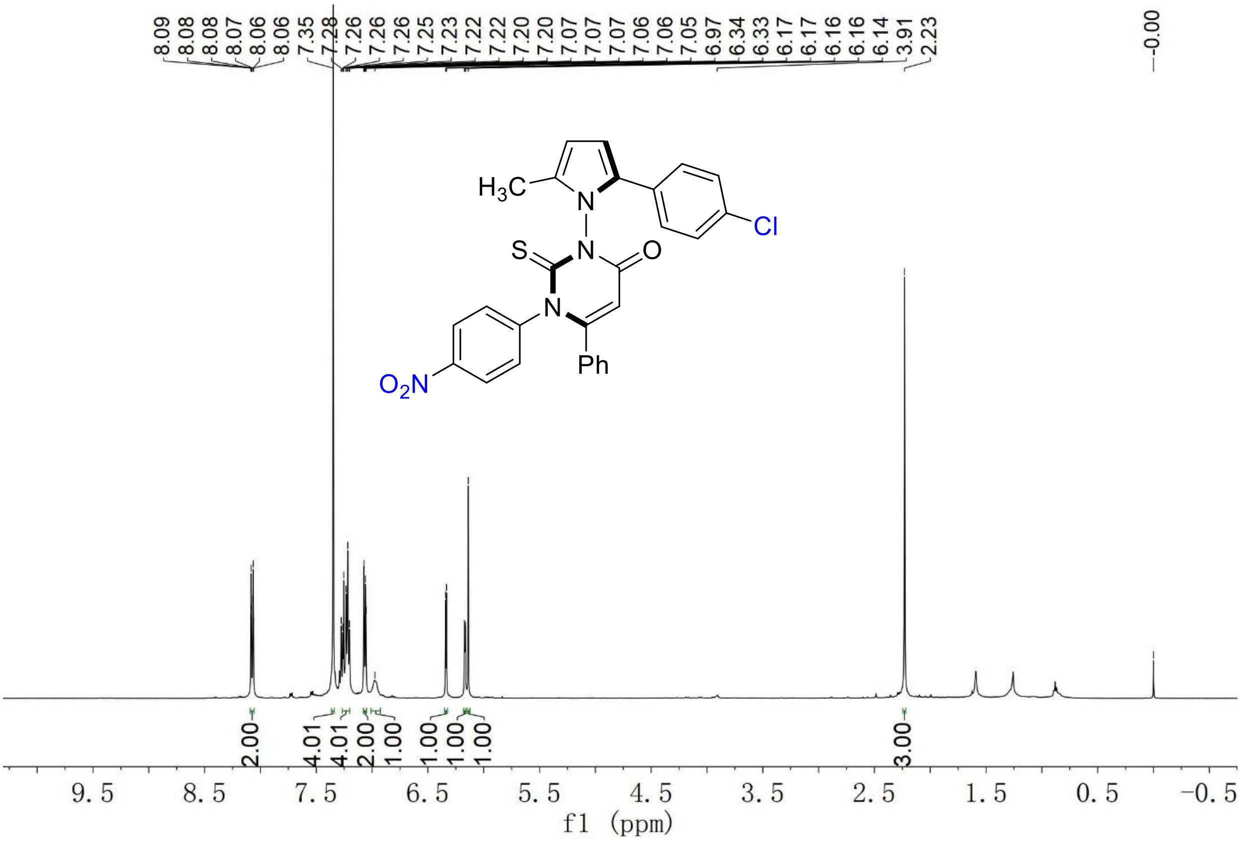

Supplementary Figure 137. ^{1}H NMR spectrum of compound 7g ($CDCl_3$, 500 MHz, 298 K)

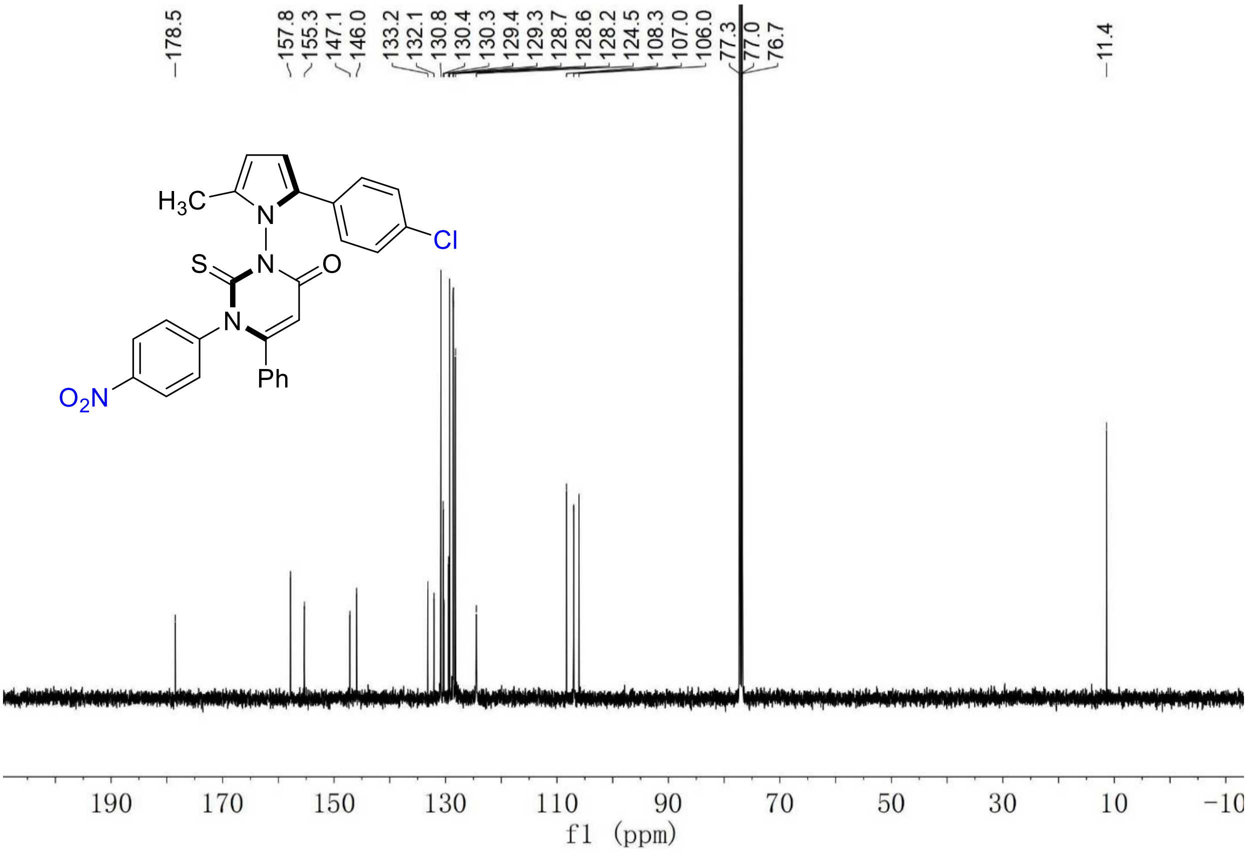

Supplementary Figure 138. ^{13}C NMR spectrum of compound 7g ($CDCl_3$, 126 MHz, 298 K)

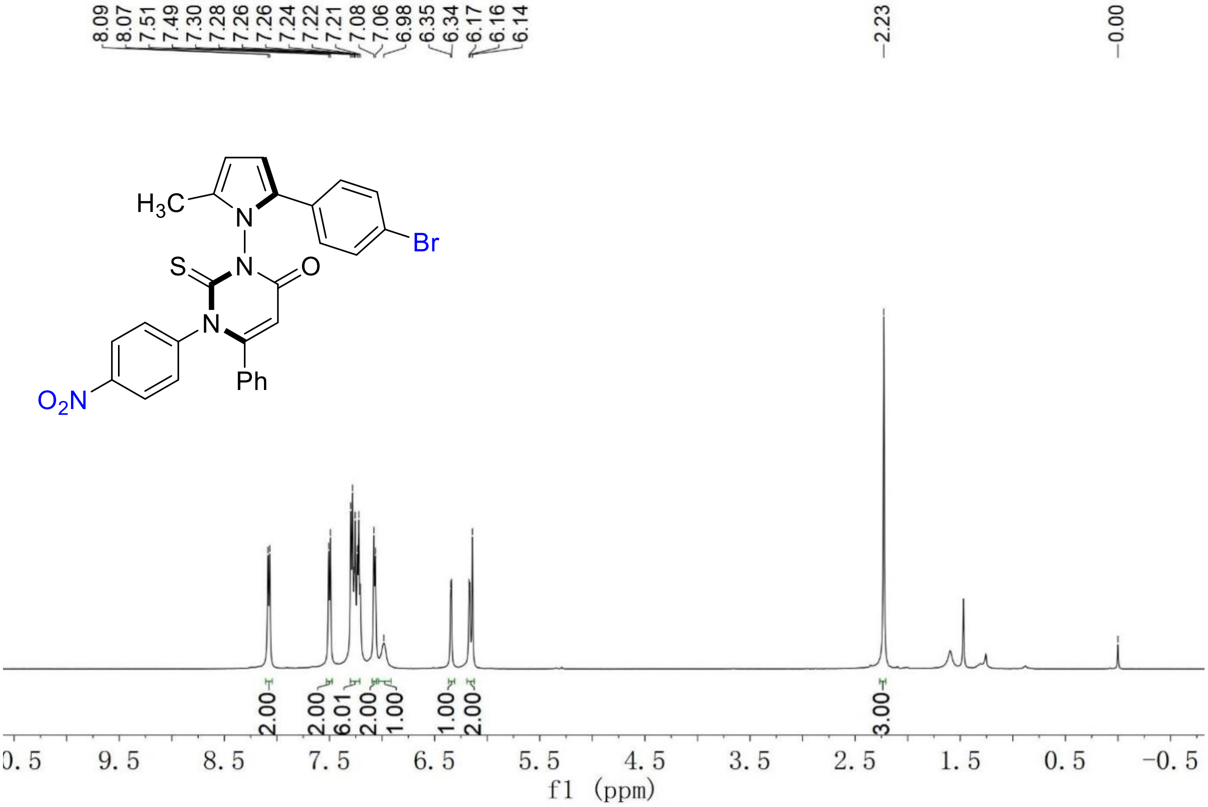

Supplementary Figure 139. **^{1}H NMR spectrum of compound 7h ($CDCl_3$, 500 MHz, 298 K)**

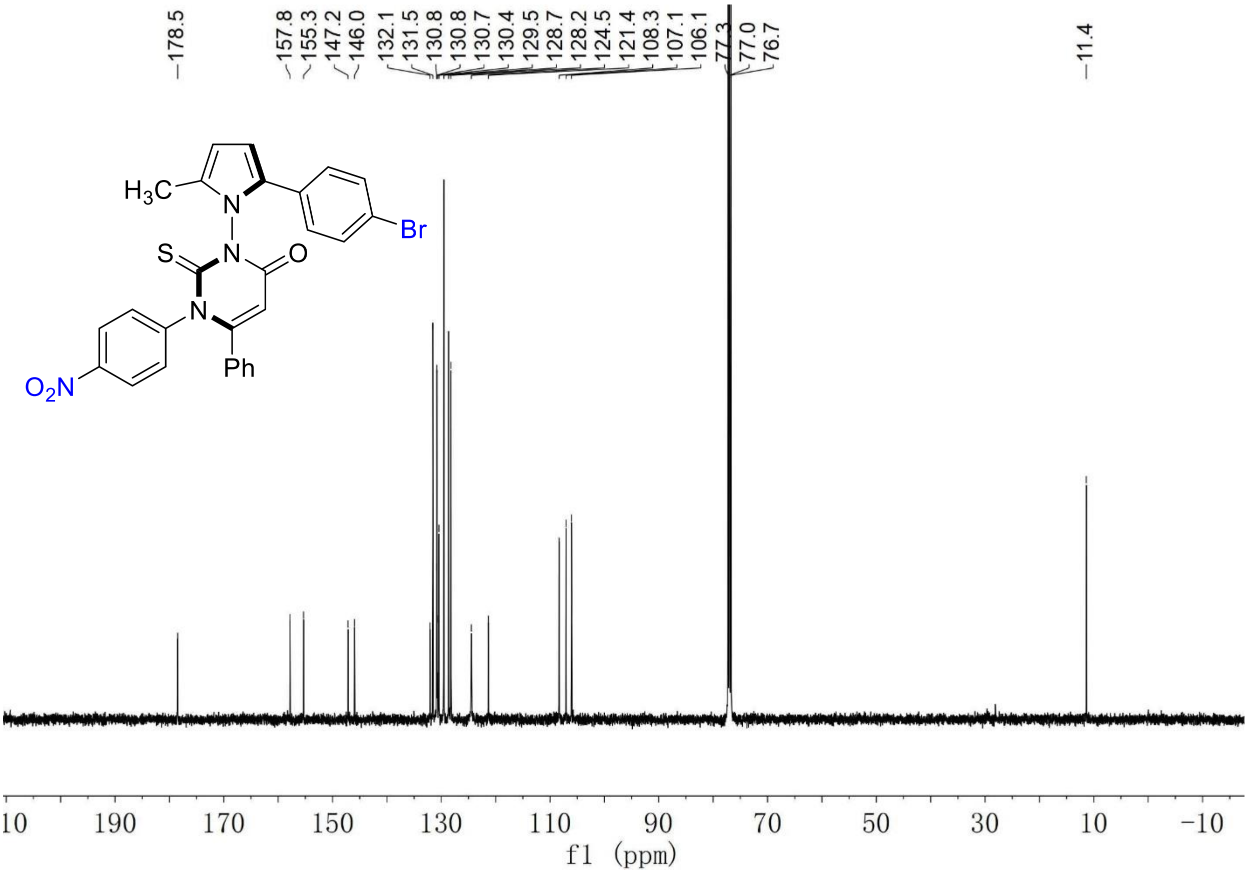

Supplementary Figure 140. **^{13}C NMR spectrum of compound 7h ($CDCl_3$, 126 MHz, 298 K)**

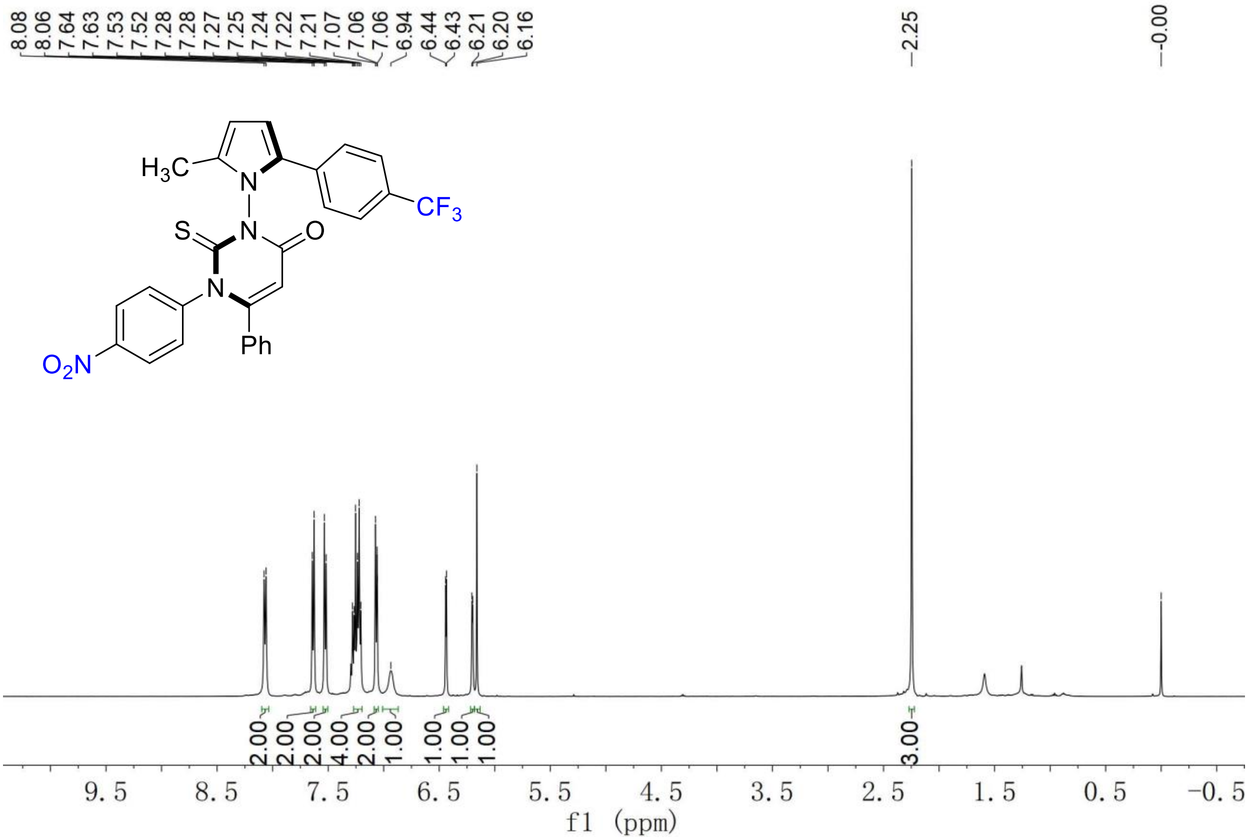

Supplementary Figure 141. ^{1}**H NMR spectrum of compound 7i (CDCl$_3$, 500 MHz, 298 K)**

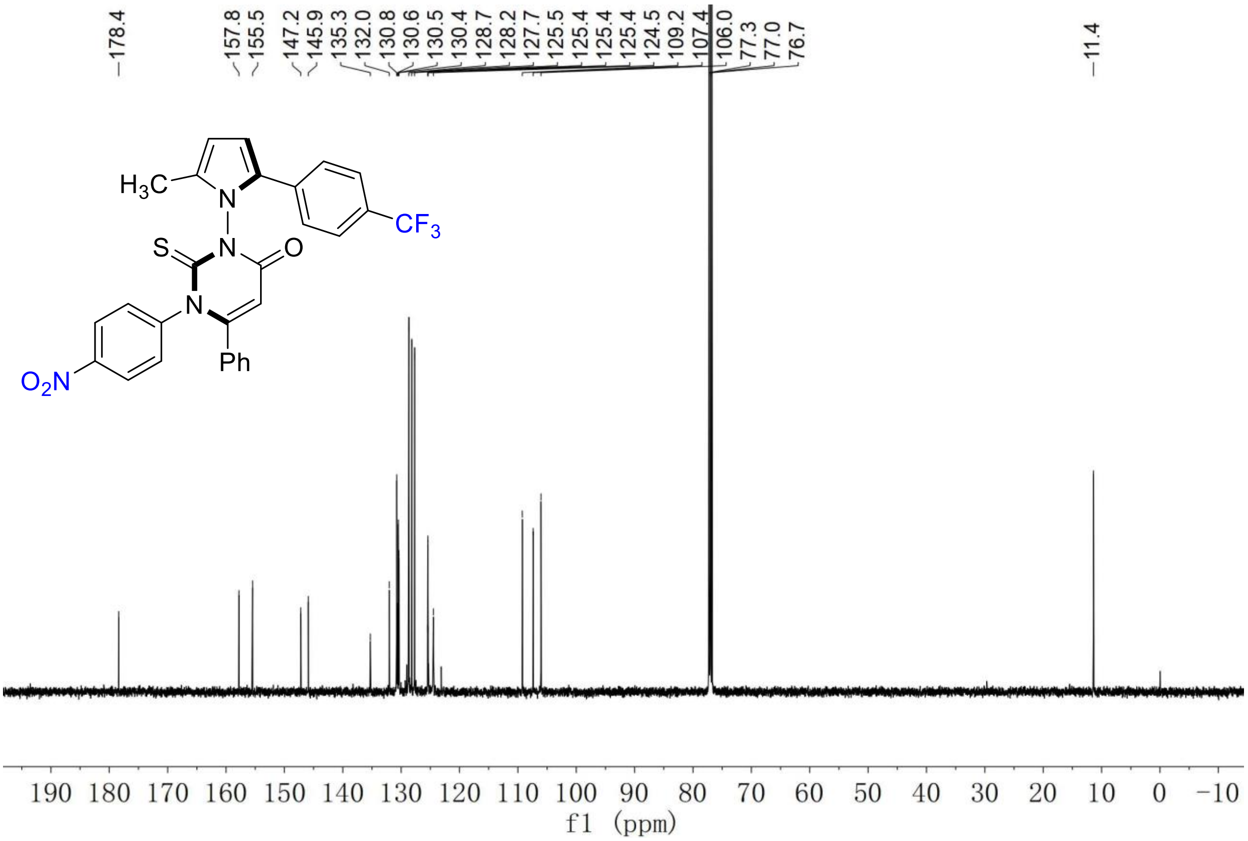

Supplementary Figure 142. ^{13}**C NMR spectrum of compound 7i (CDCl$_3$, 126 MHz, 298 K)**

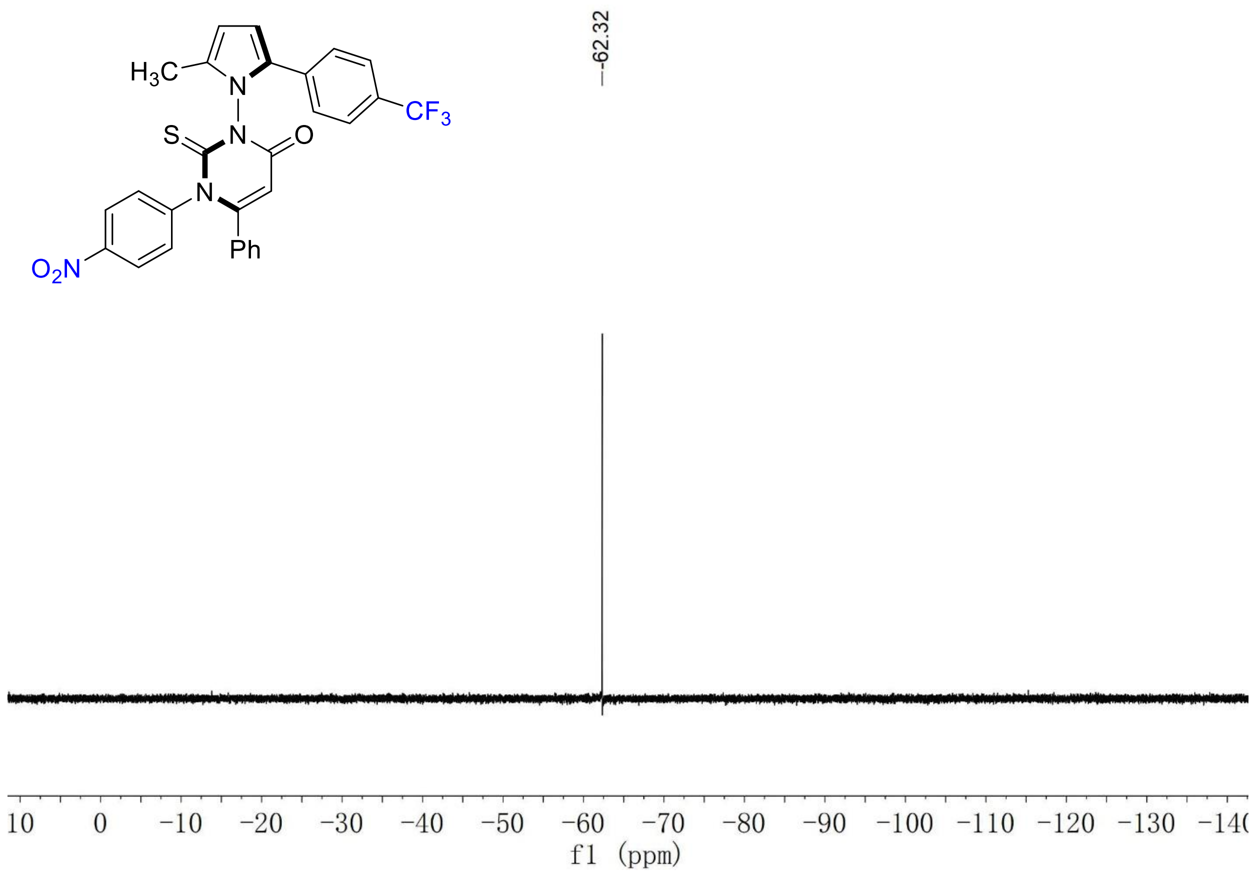

Supplementary Figure 143. ^{19}F NMR spectrum of compound 7i ($CDCl_3$, 471 MHz, 298 K)

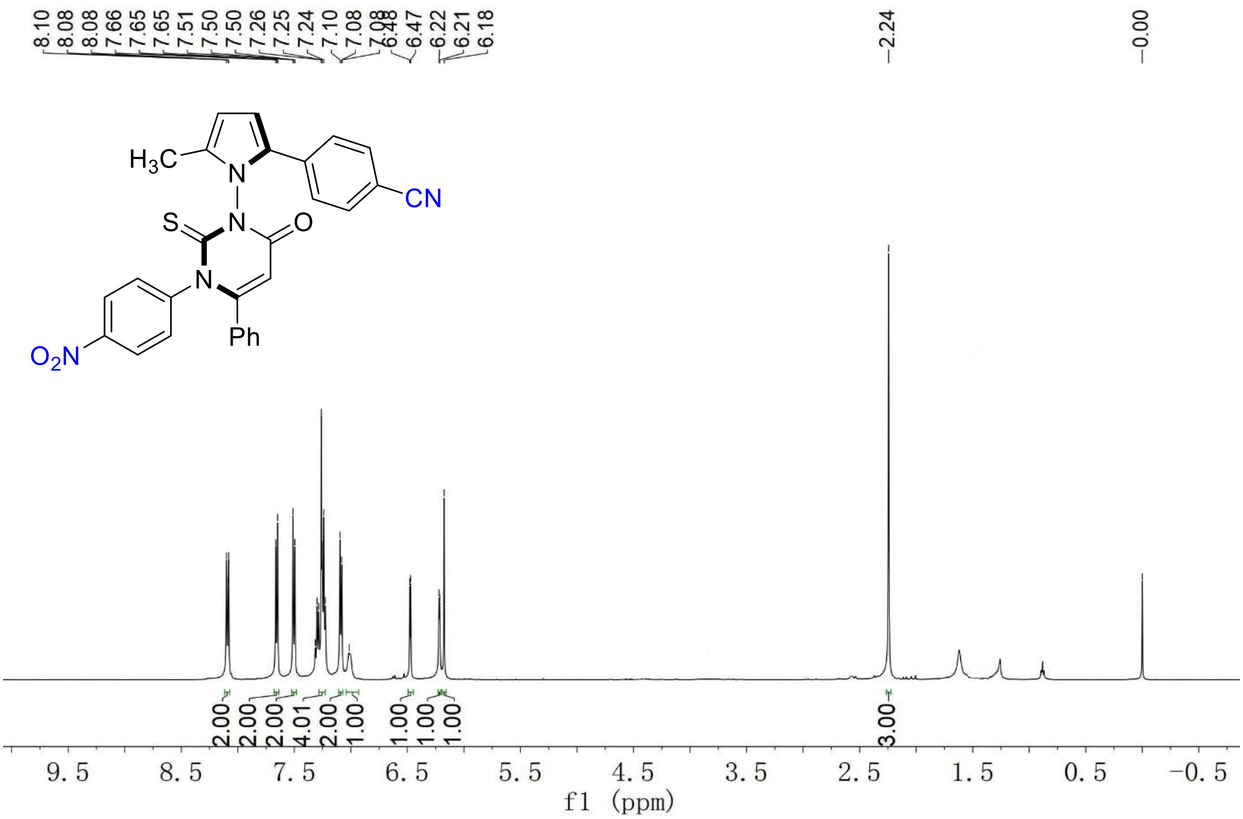

Supplementary Figure 144. ^{1}**H NMR spectrum of compound 7j (CDCl$_3$, 500 MHz, 298 K)**

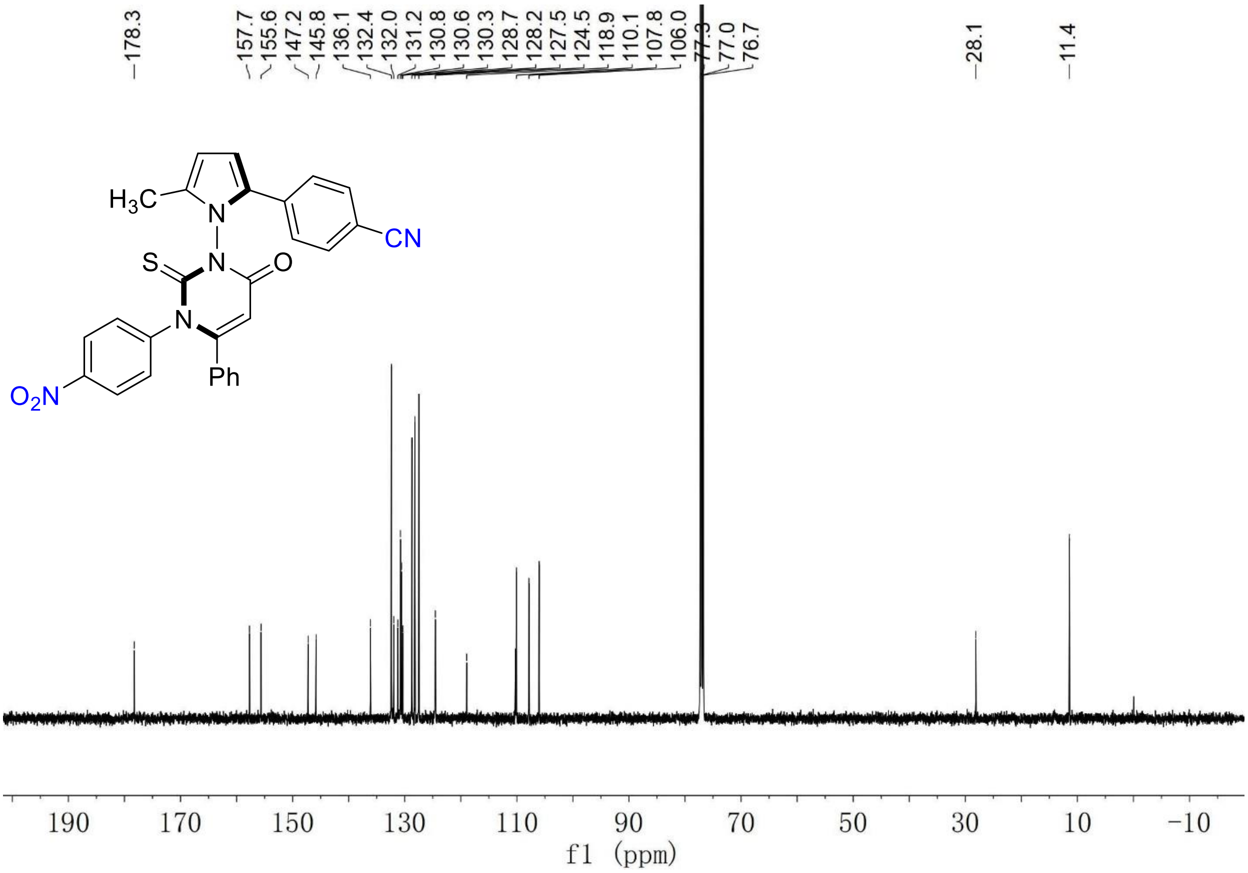

Supplementary Figure 145. ^{13}**C NMR spectrum of compound 7j (CDCl$_3$, 126 MHz, 298 K)**

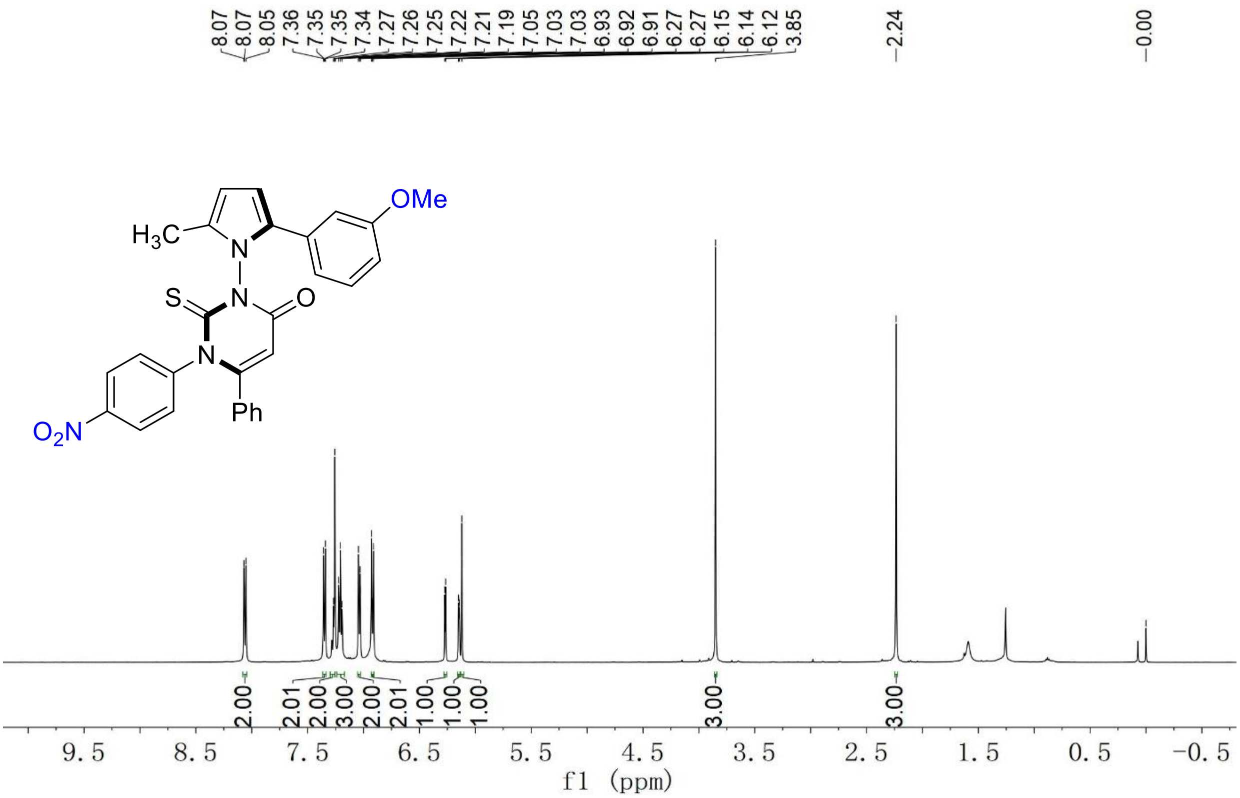

Supplementary Figure 146. ^{1}H NMR spectrum of compound 7k ($CDCl_3$, 500 MHz, 298 K)

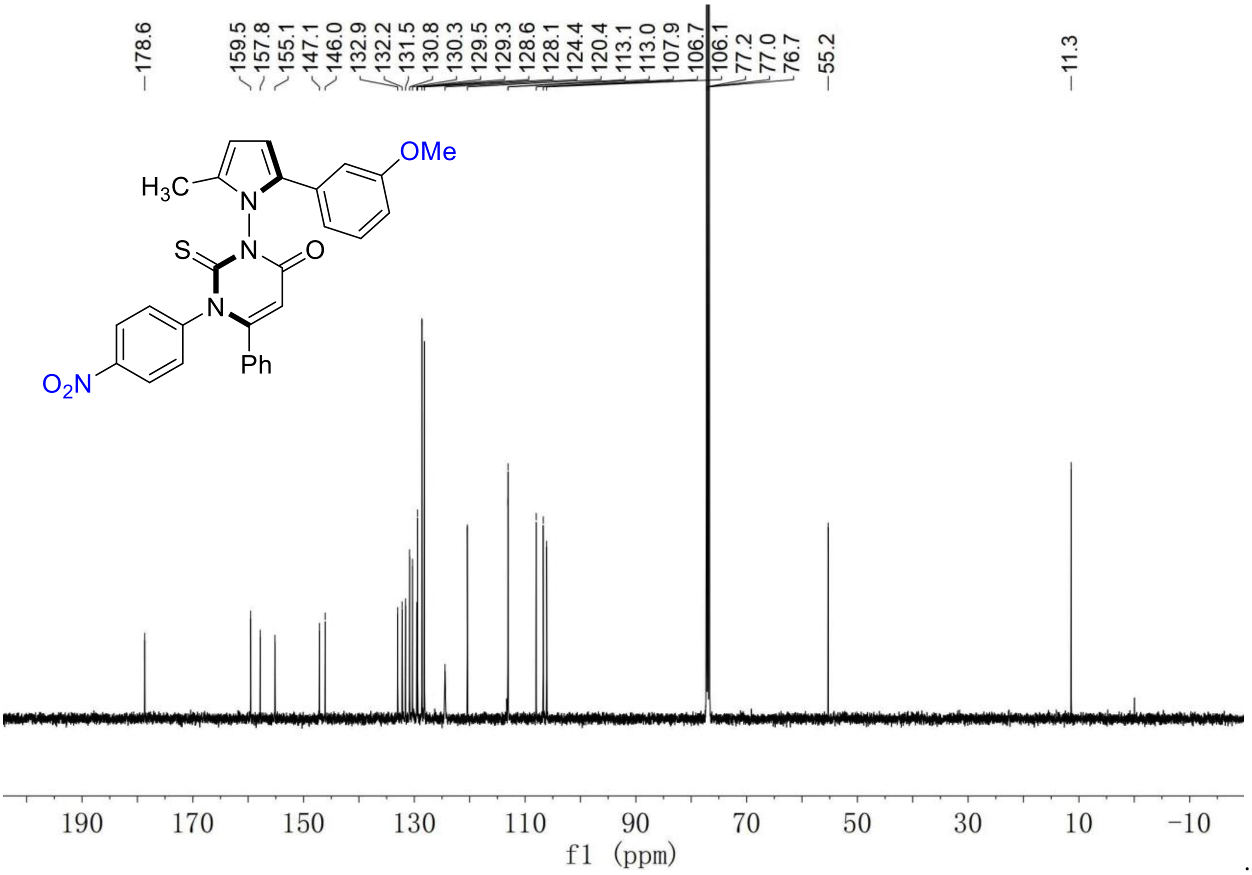

Supplementary Figure 147. ^{13}C NMR spectrum of compound 7k ($CDCl_3$, 126 MHz, 298 K)

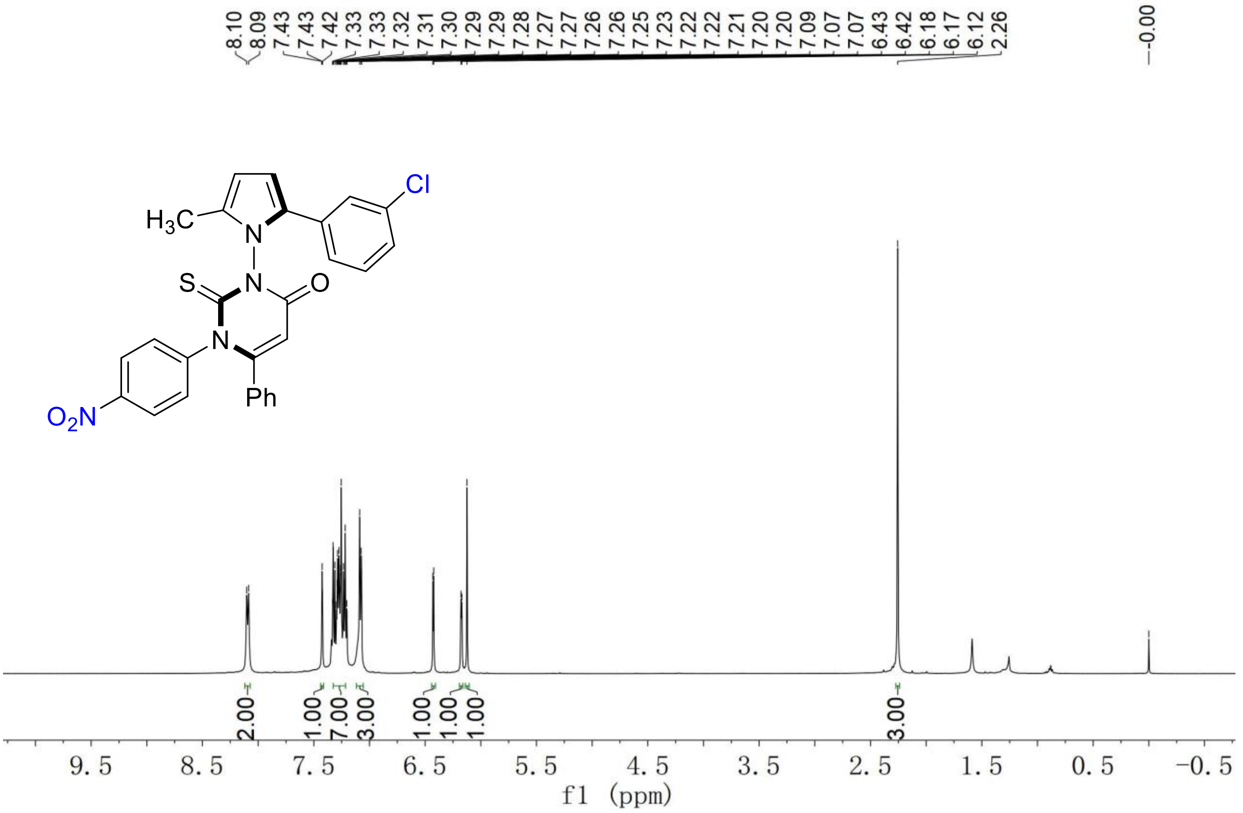

Supplementary Figure 148. ^{1}H NMR spectrum of compound 7l ($CDCl_3$, 500 MHz, 298 K)

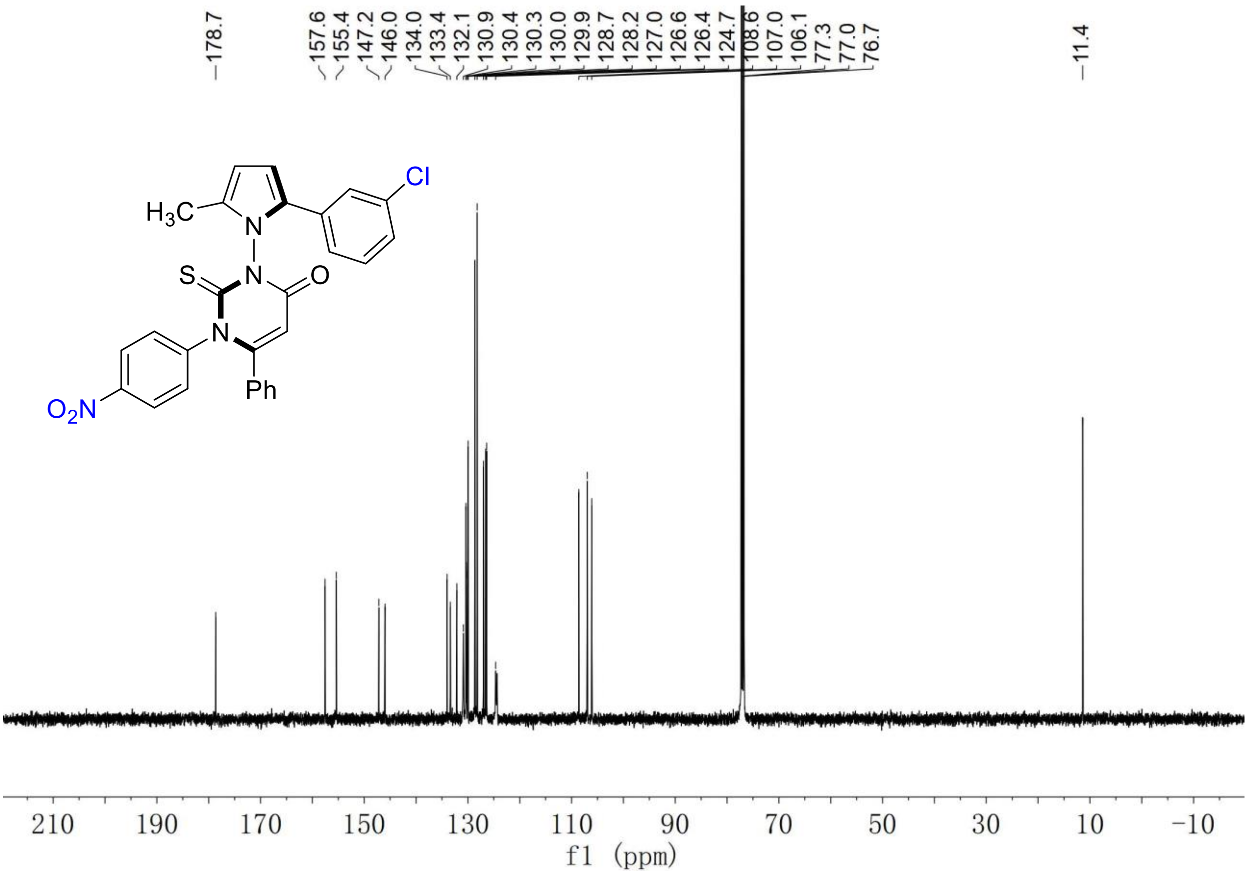

Supplementary Figure 149. ^{13}C NMR spectrum of compound 7l ($CDCl_3$, 126 MHz, 298 K)

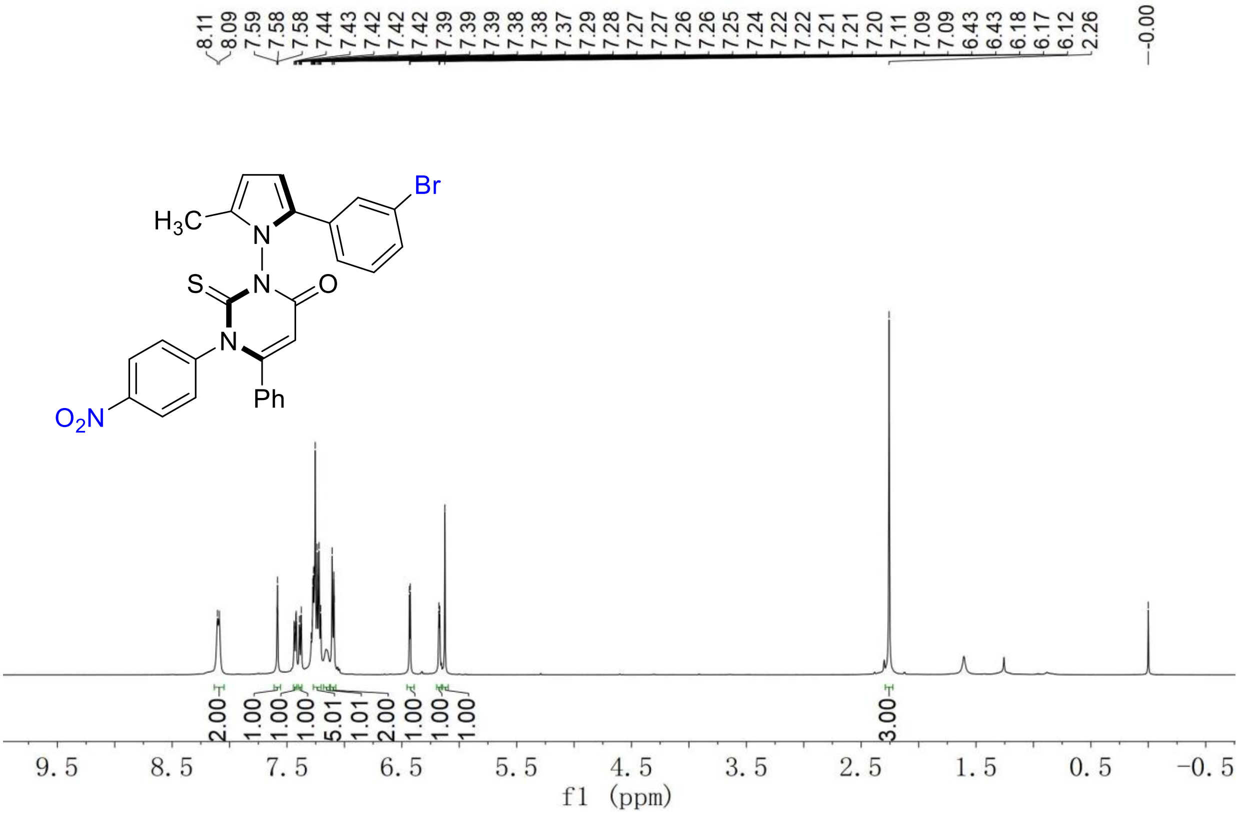

Supplementary Figure 150. ^{1}H NMR spectrum of compound 7m ($CDCl_3$, 500 MHz, 298 K)

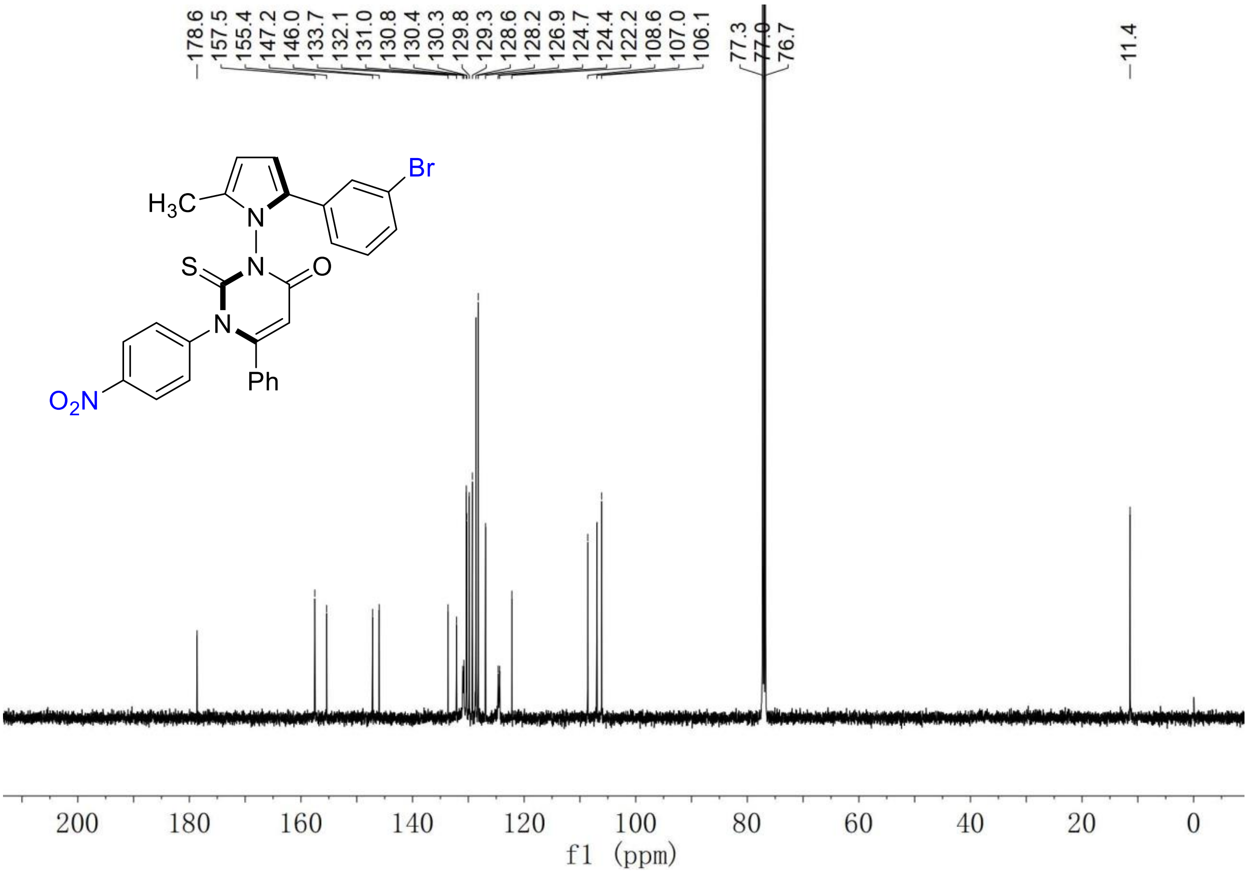

Supplementary Figure 151. ^{13}C NMR spectrum of compound 7m ($CDCl_3$, 126 MHz, 298 K)

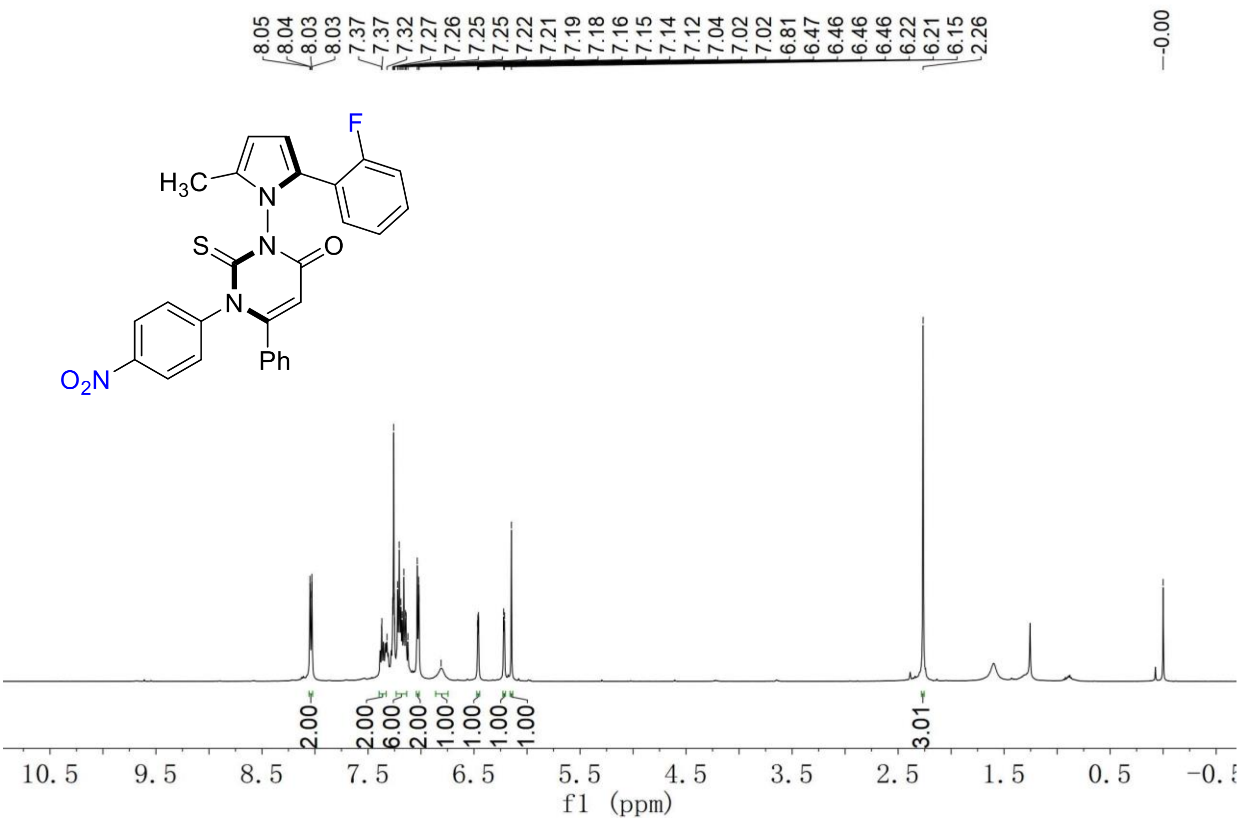

Supplementary Figure 152. ^{1}H NMR spectrum of compound 7n ($CDCl_3$, 500 MHz, 298 K)

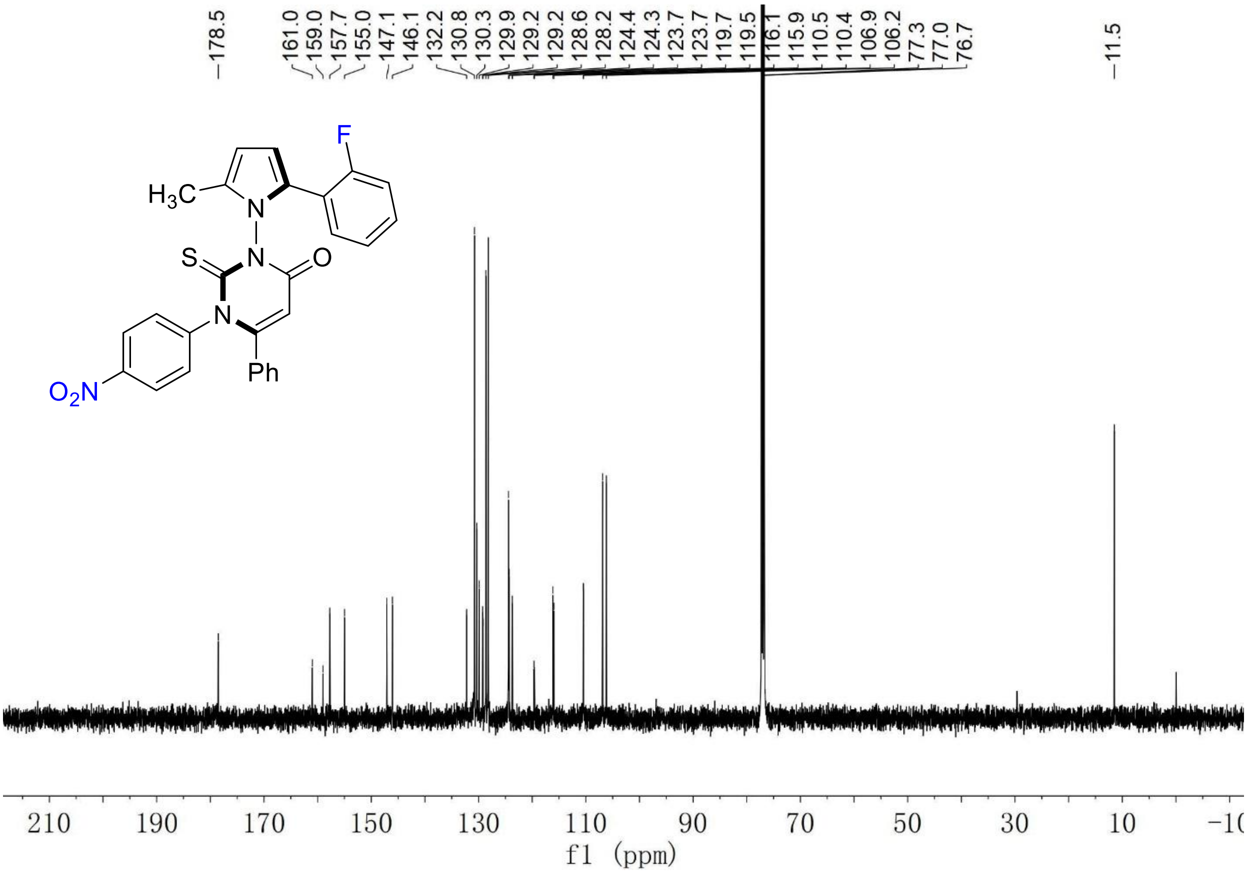

Supplementary Figure 153. ^{13}C NMR spectrum of compound 7n ($CDCl_3$, 126 MHz, 298 K)

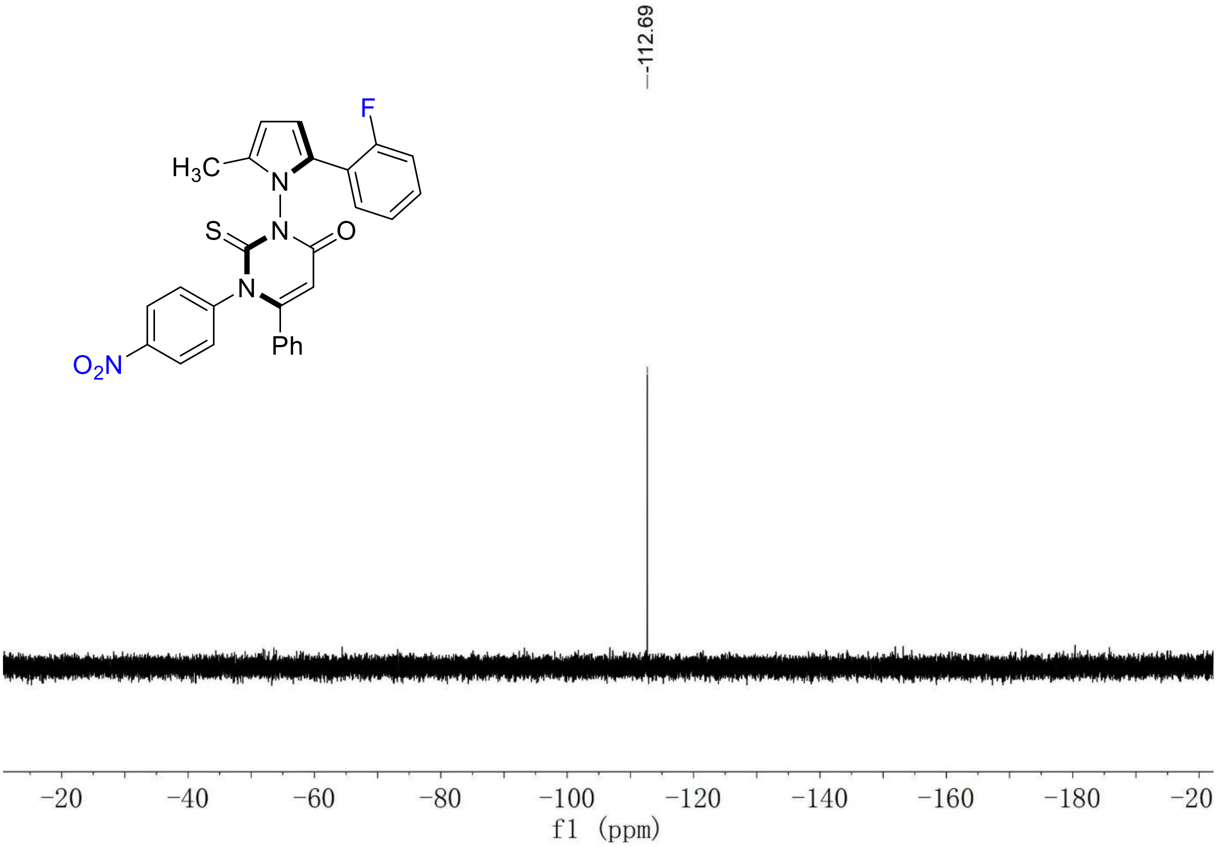

Supplementary Figure 154. ^{19}F NMR spectrum of compound 7n ($CDCl_3$, 471 MHz, 298 K)

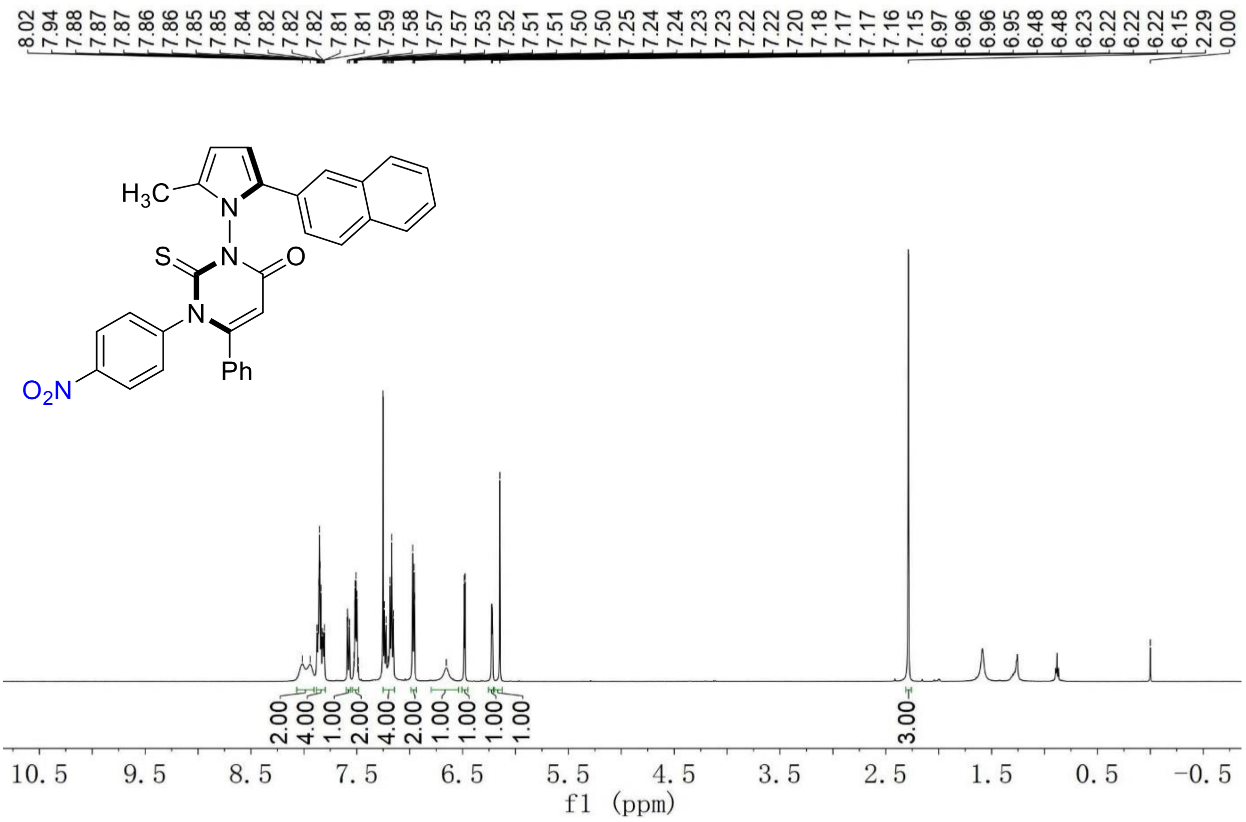

Supplementary Figure 155. ^{1}**H NMR spectrum of compound 7o (CDCl$_3$, 500 MHz, 298 K)**

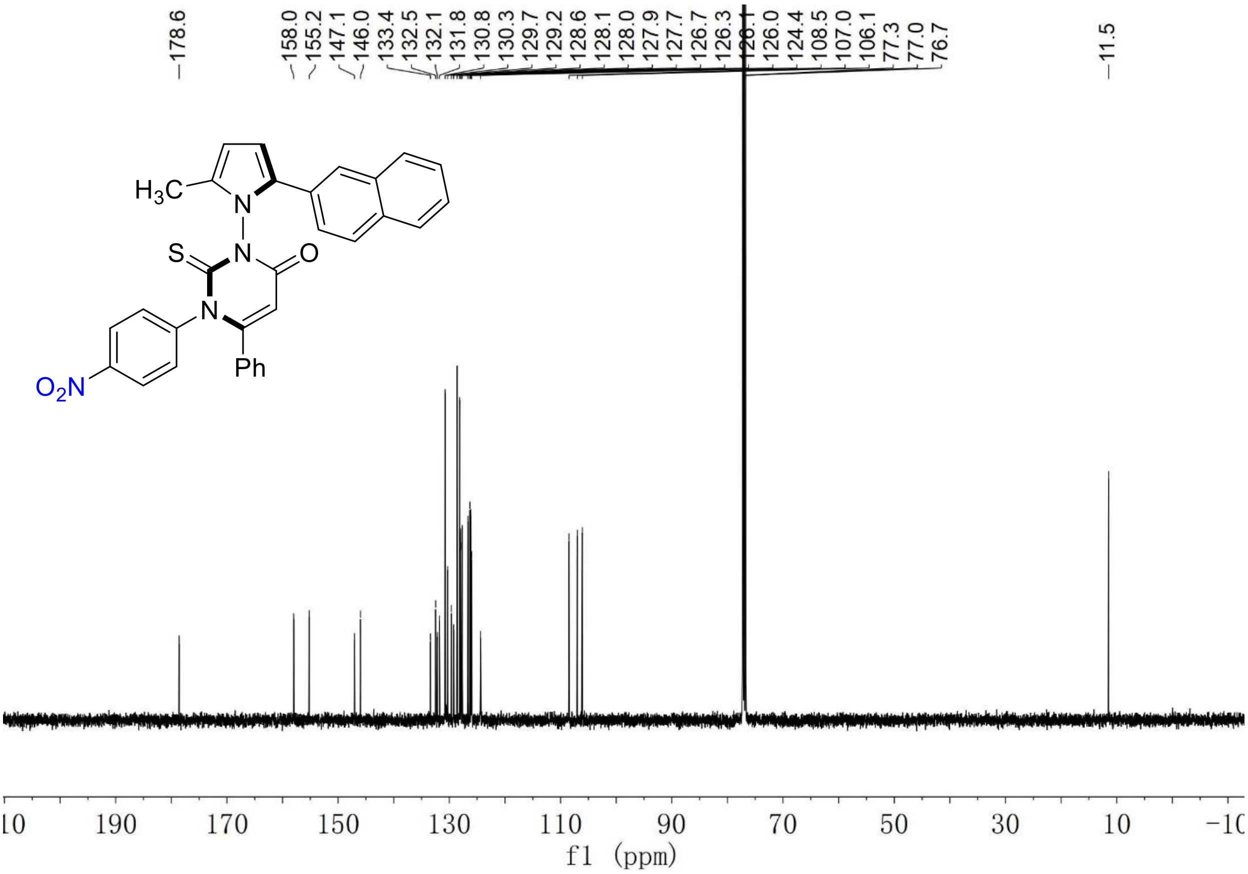

Supplementary Figure 156. ^{13}**C NMR spectrum of compound 7o (CDCl$_3$, 126 MHz, 298 K)**

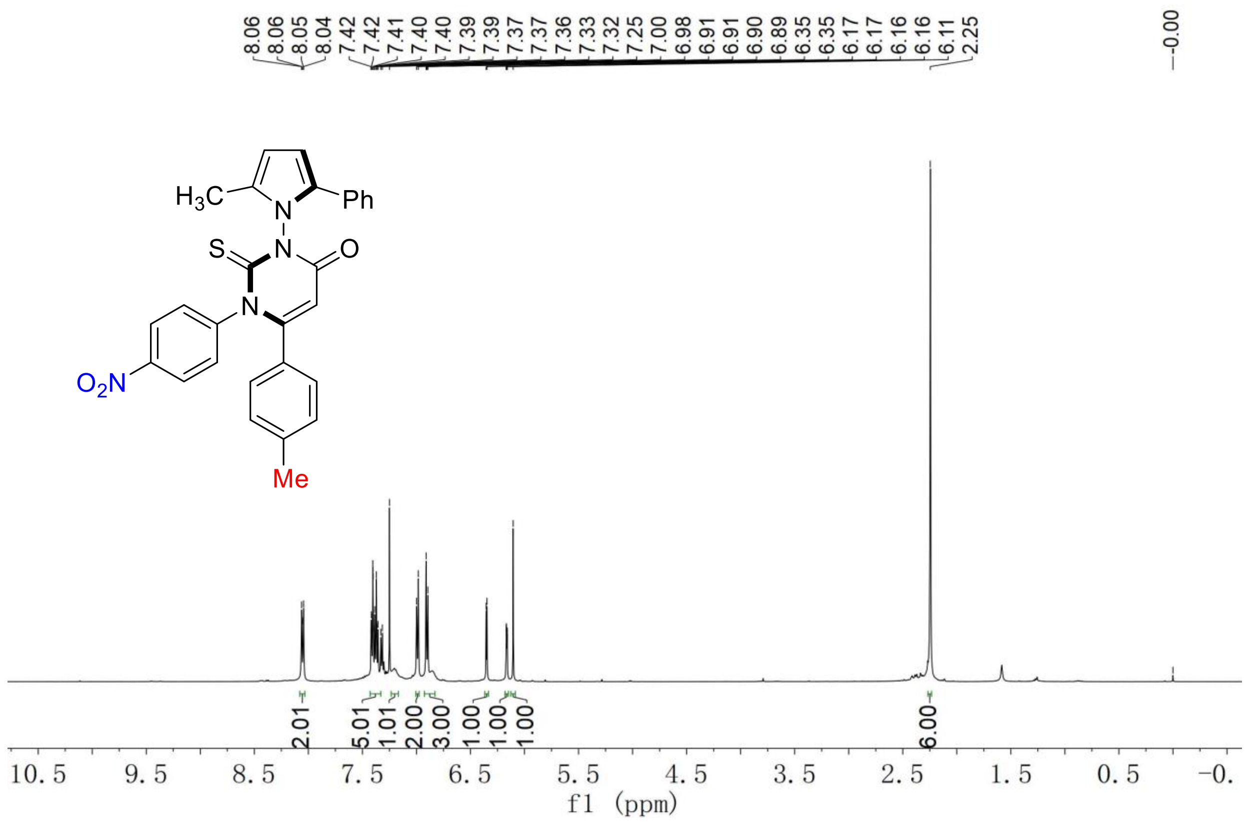

Supplementary Figure 157. ^{1}H NMR spectrum of compound 7p ($CDCl_3$, 500 MHz, 298 K)

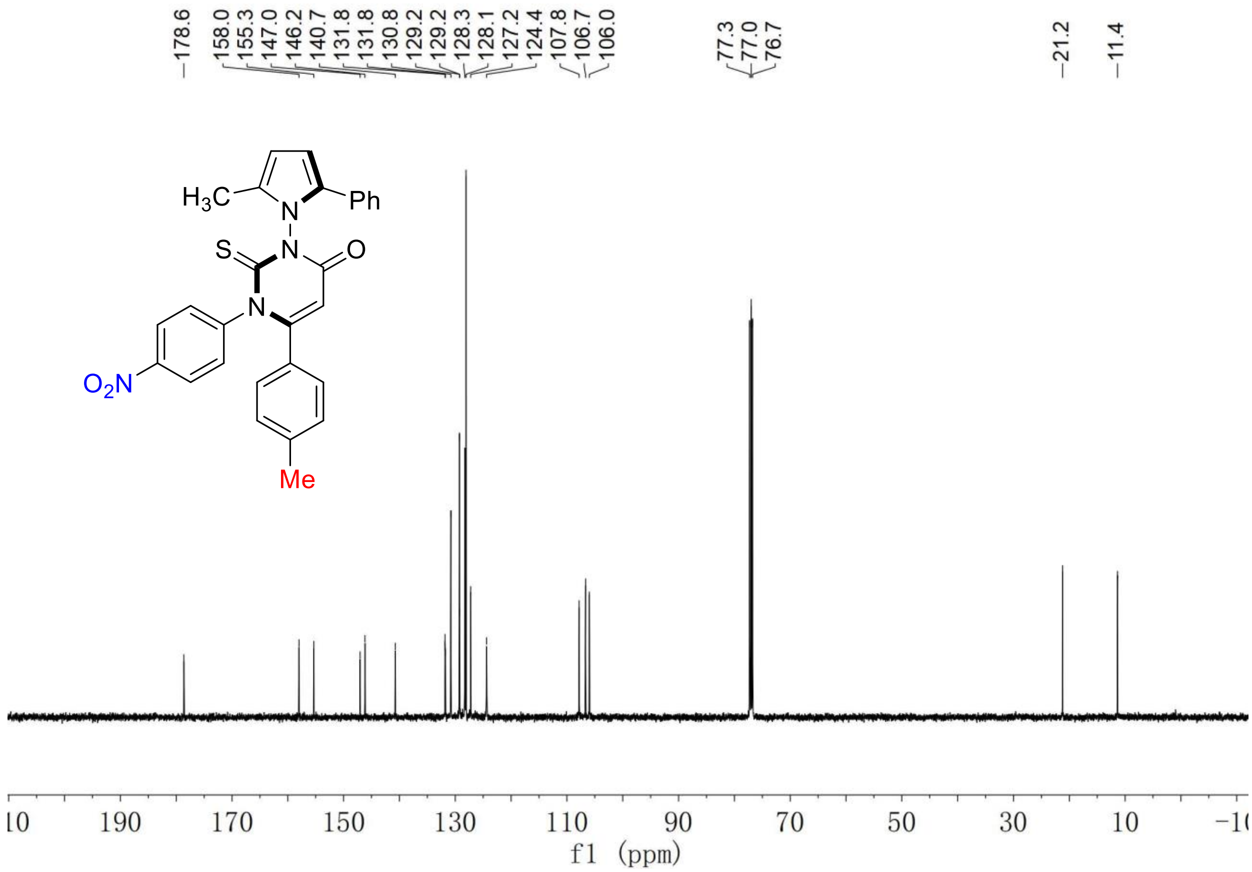

Supplementary Figure 158. ^{13}C NMR spectrum of compound 7p ($CDCl_3$, 126 MHz, 298 K)

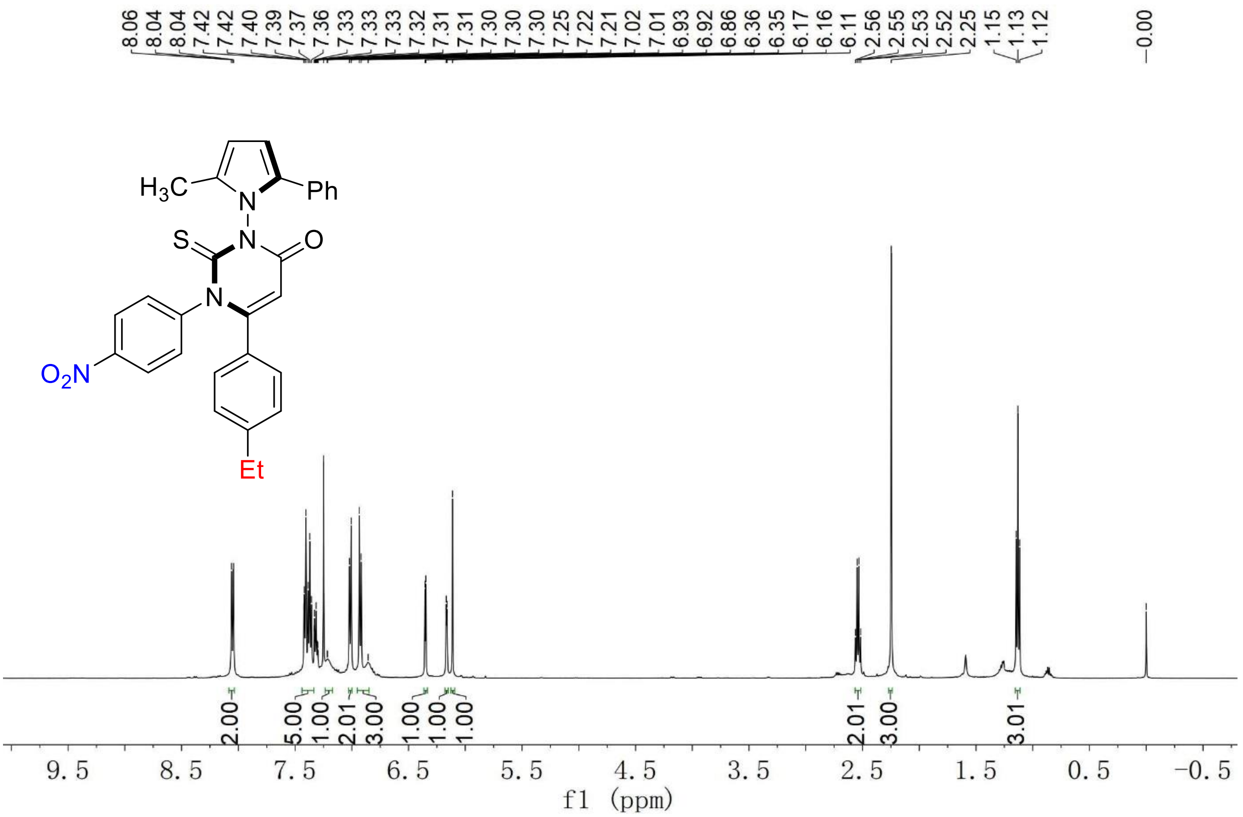

Supplementary Figure 159. ^{1}H NMR spectrum of compound 7q ($CDCl_3$, 500 MHz, 298 K)

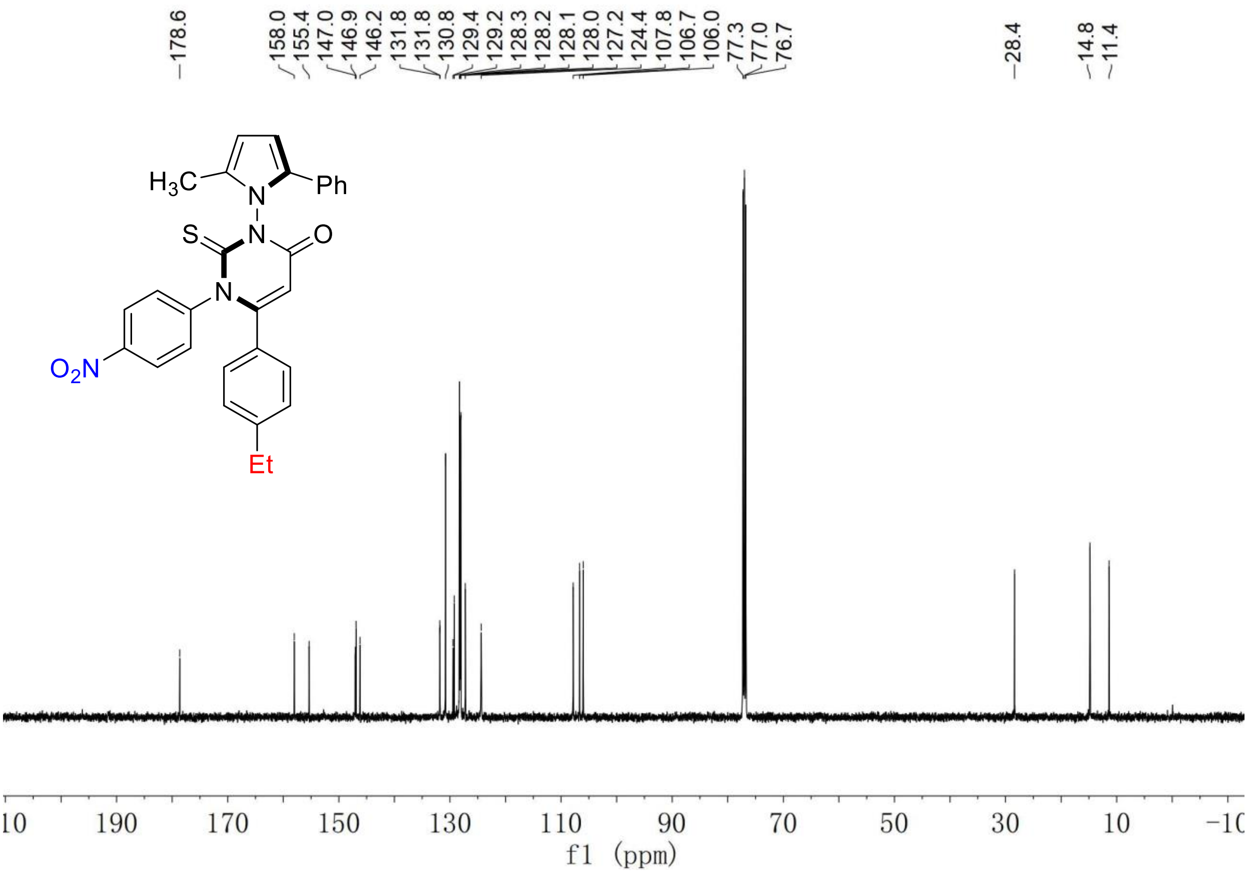

Supplementary Figure 160. ^{13}C NMR spectrum of compound 7q ($CDCl_3$, 126 MHz, 298 K)

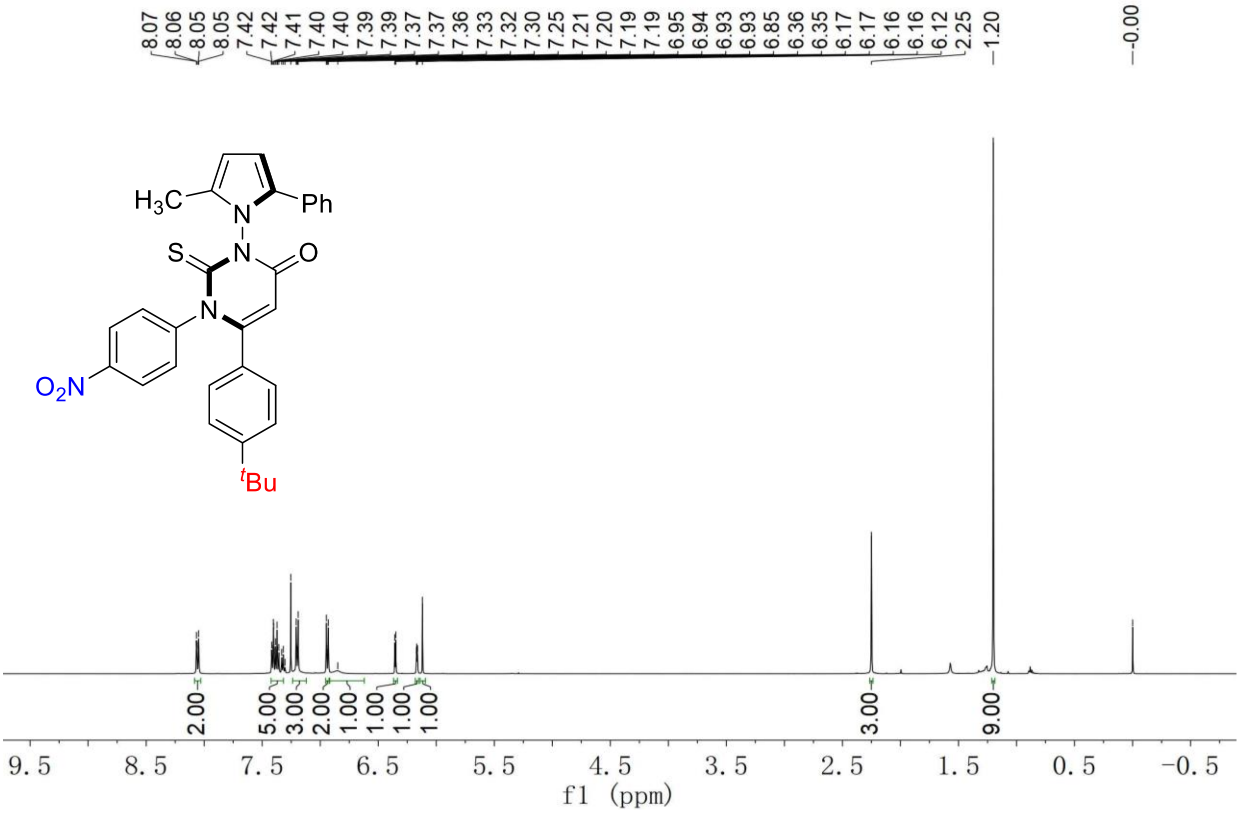

Supplementary Figure 161. ^{1}**H NMR spectrum of compound 7r (CDCl**$_3$**, 500 MHz, 298 K)**

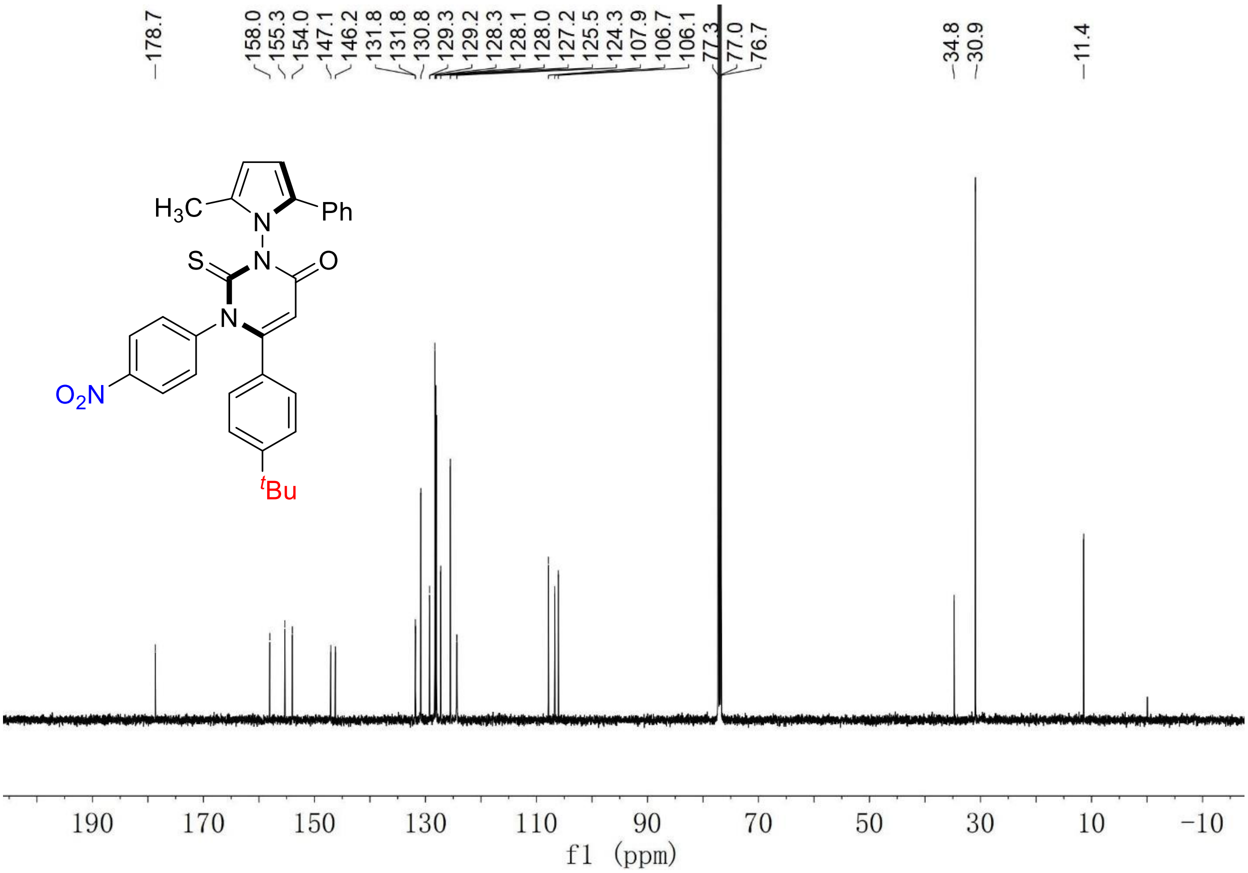

Supplementary Figure 162. ^{13}**C NMR spectrum of compound 7r (CDCl**$_3$**, 126 MHz, 298 K)**

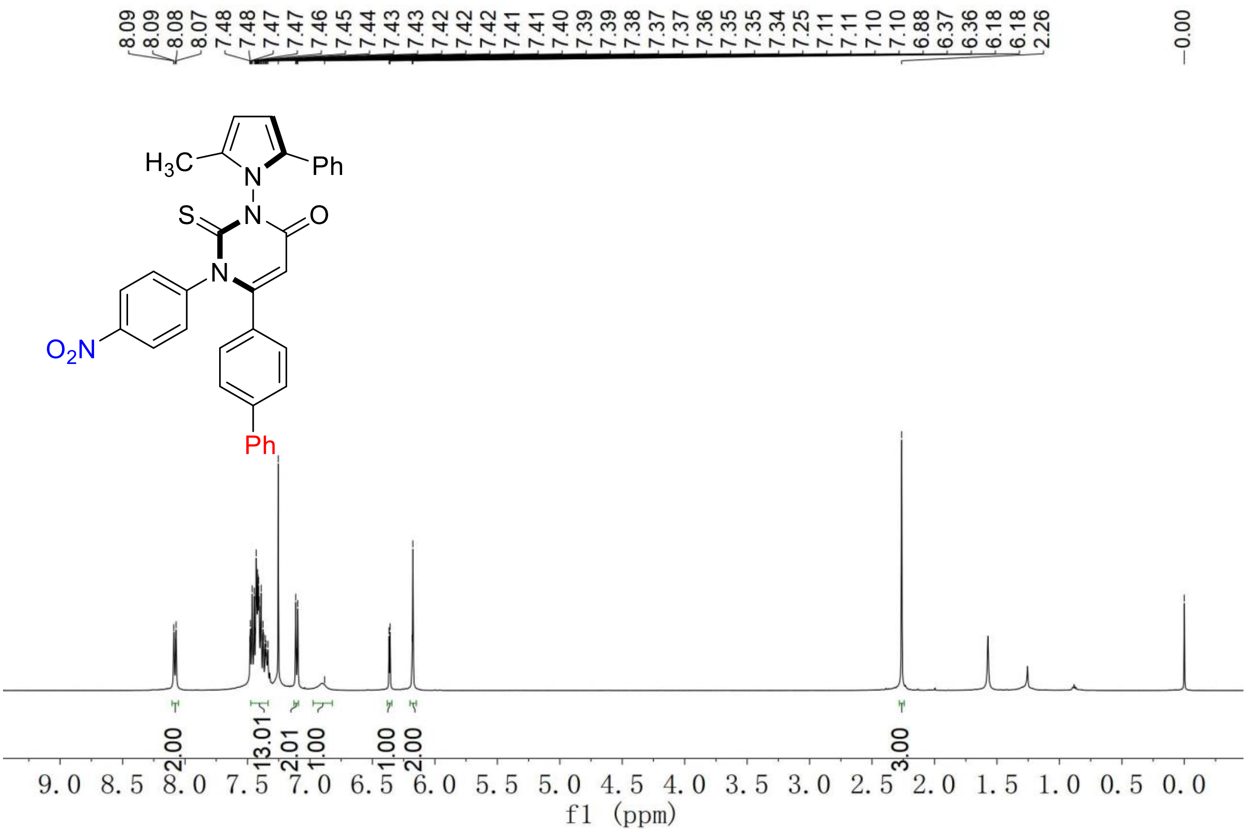

Supplementary Figure 163. ^{1}H NMR spectrum of compound 7s ($CDCl_3$, 500 MHz, 298 K)

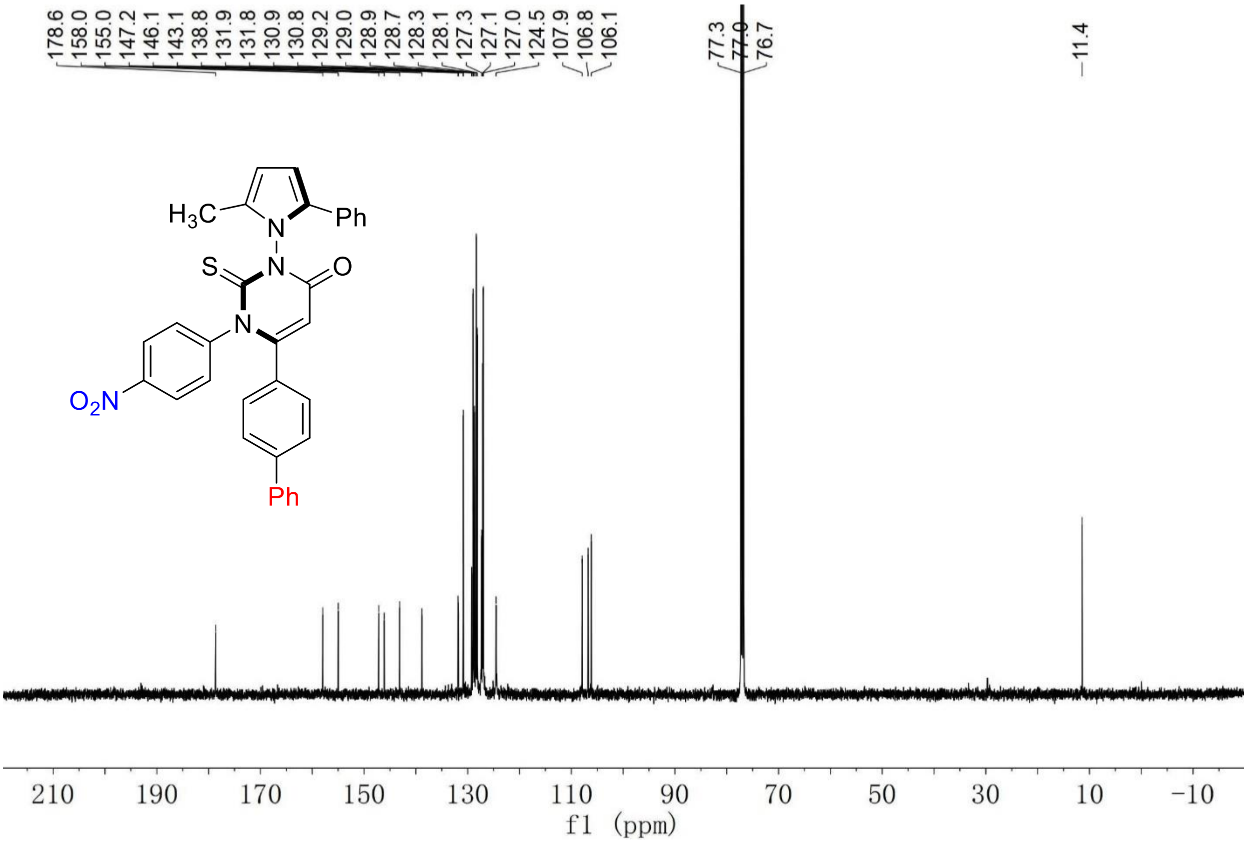

Supplementary Figure 164. ^{13}C NMR spectrum of compound 7s ($CDCl_3$, 126 MHz, 298 K)

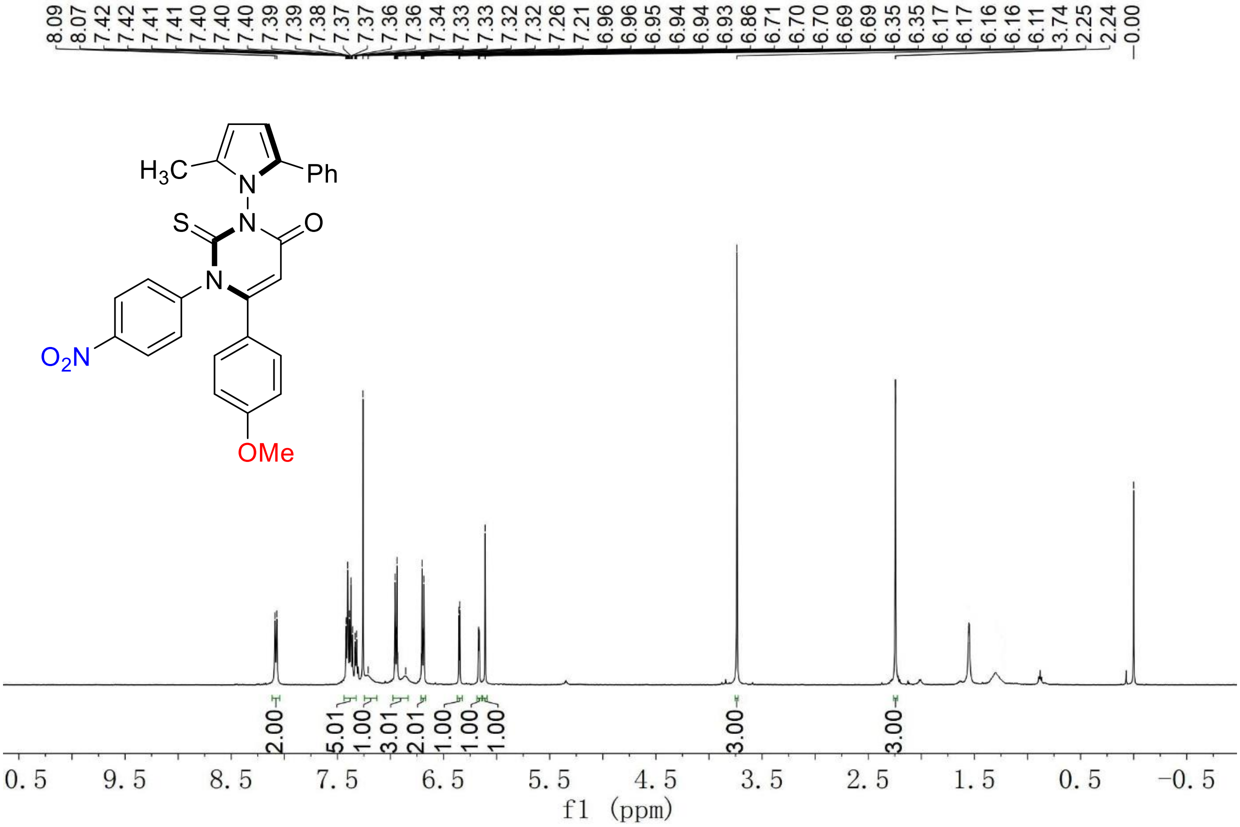

Supplementary Figure 165. ^{1}H NMR spectrum of compound 7t ($CDCl_3$, 500 MHz, 298 K)

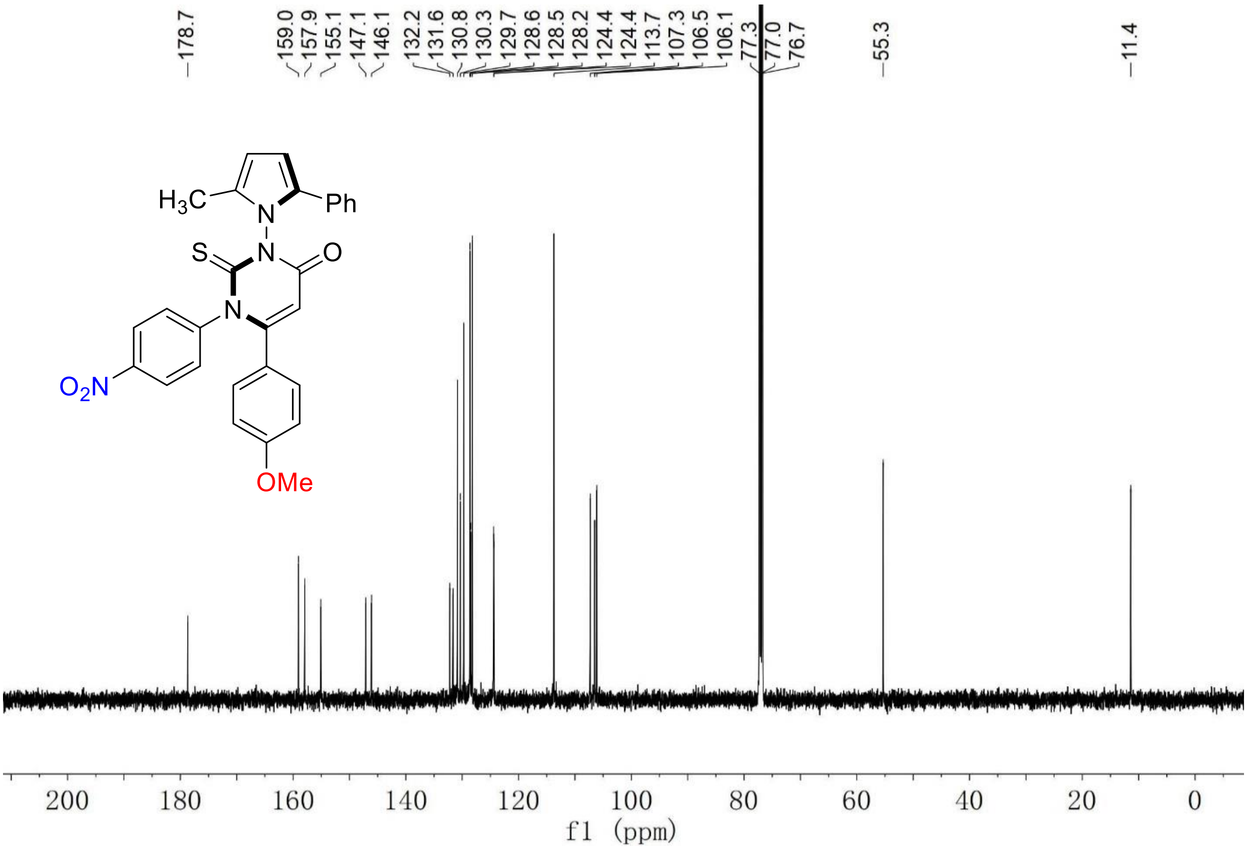

Supplementary Figure 166. ^{13}C NMR spectrum of compound 7t ($CDCl_3$, 126 MHz, 298 K)

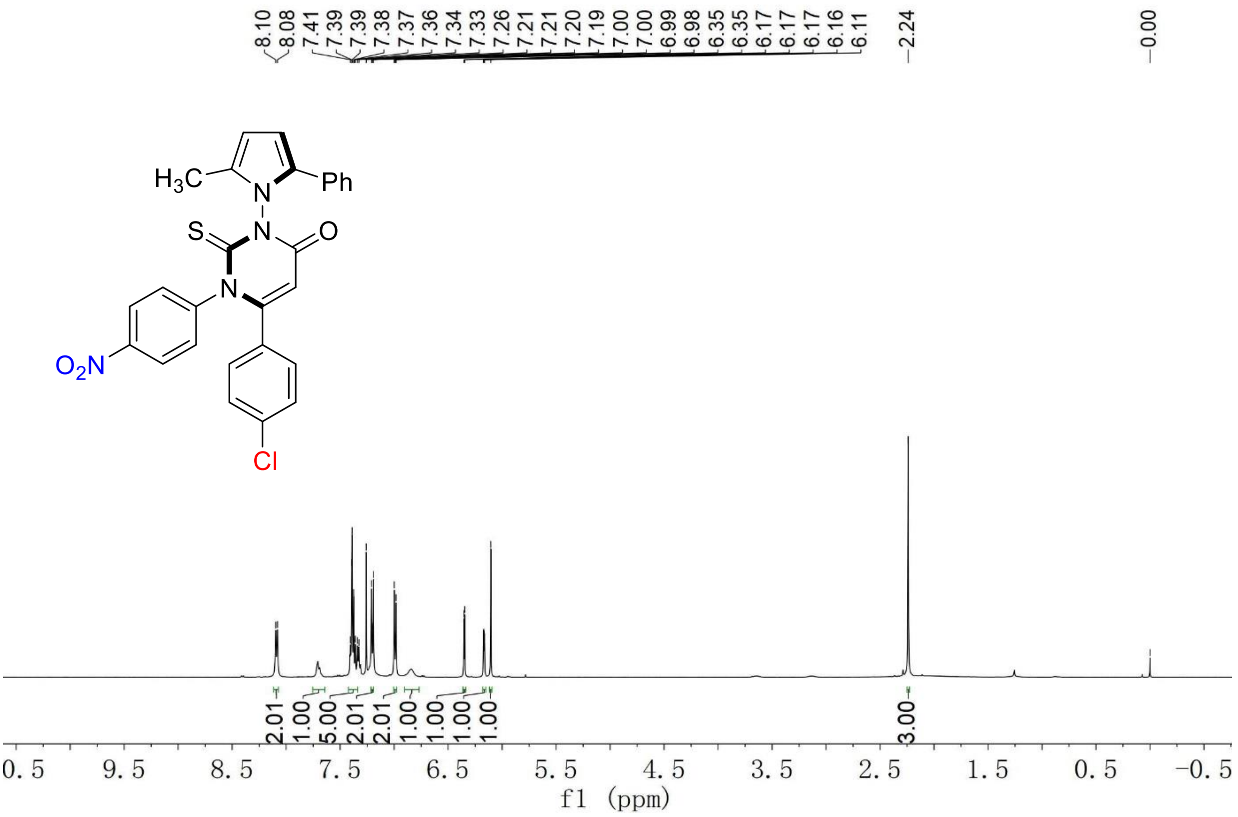

Supplementary Figure 167. ^{1}H NMR spectrum of compound 7u ($CDCl_3$, 500 MHz, 298 K)

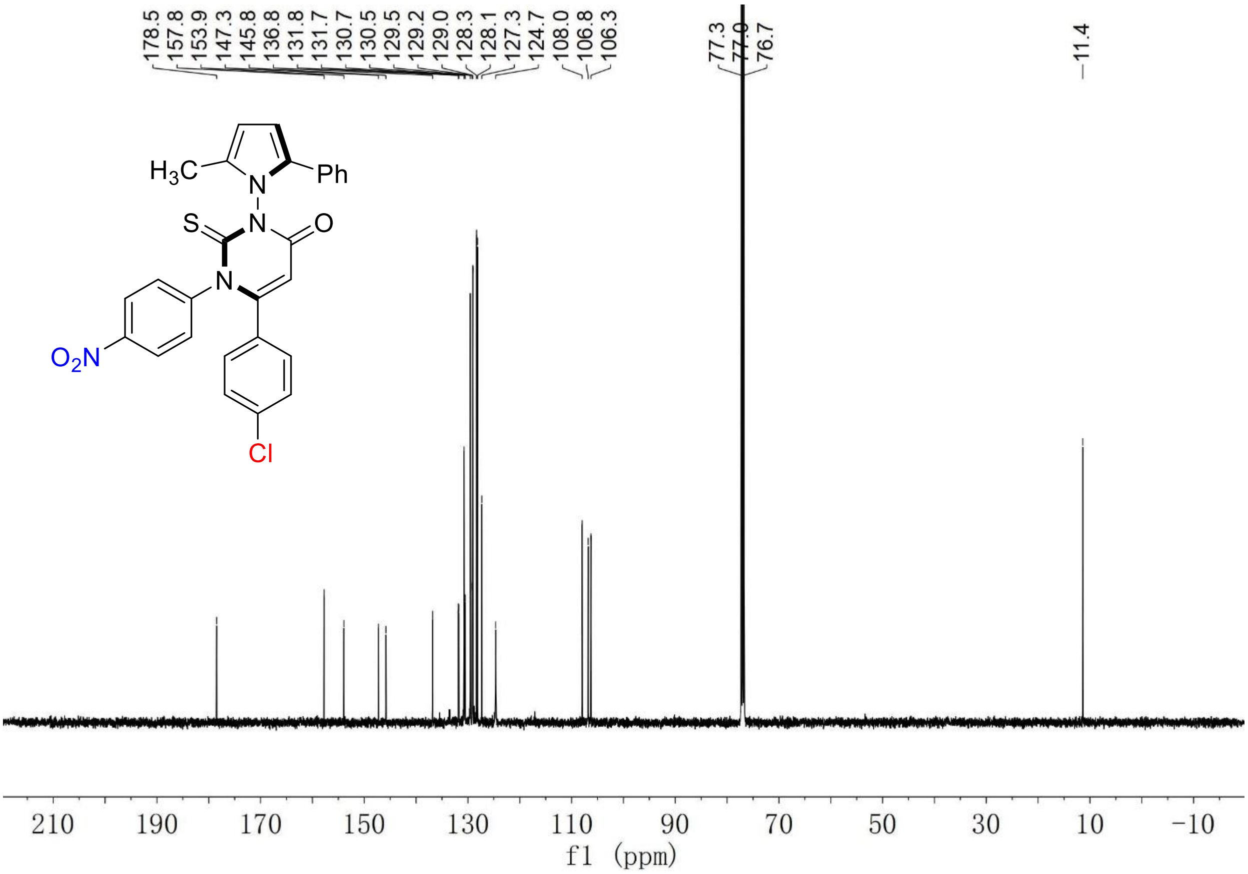

Supplementary Figure 168. ^{13}C NMR spectrum of compound 7u ($CDCl_3$, 126 MHz, 298 K)

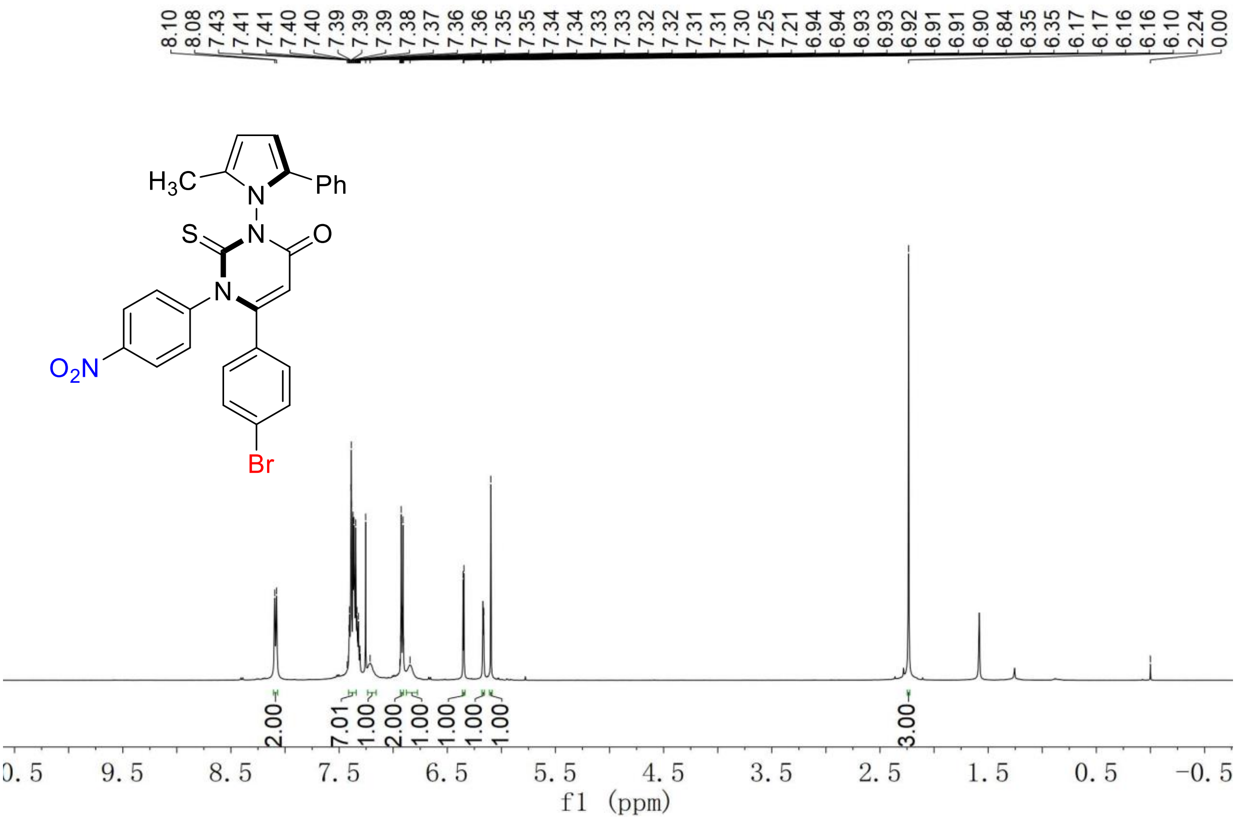

Supplementary Figure 169. ^{1}H NMR spectrum of compound **7v** ($CDCl_3$, 500 MHz, 298 K)

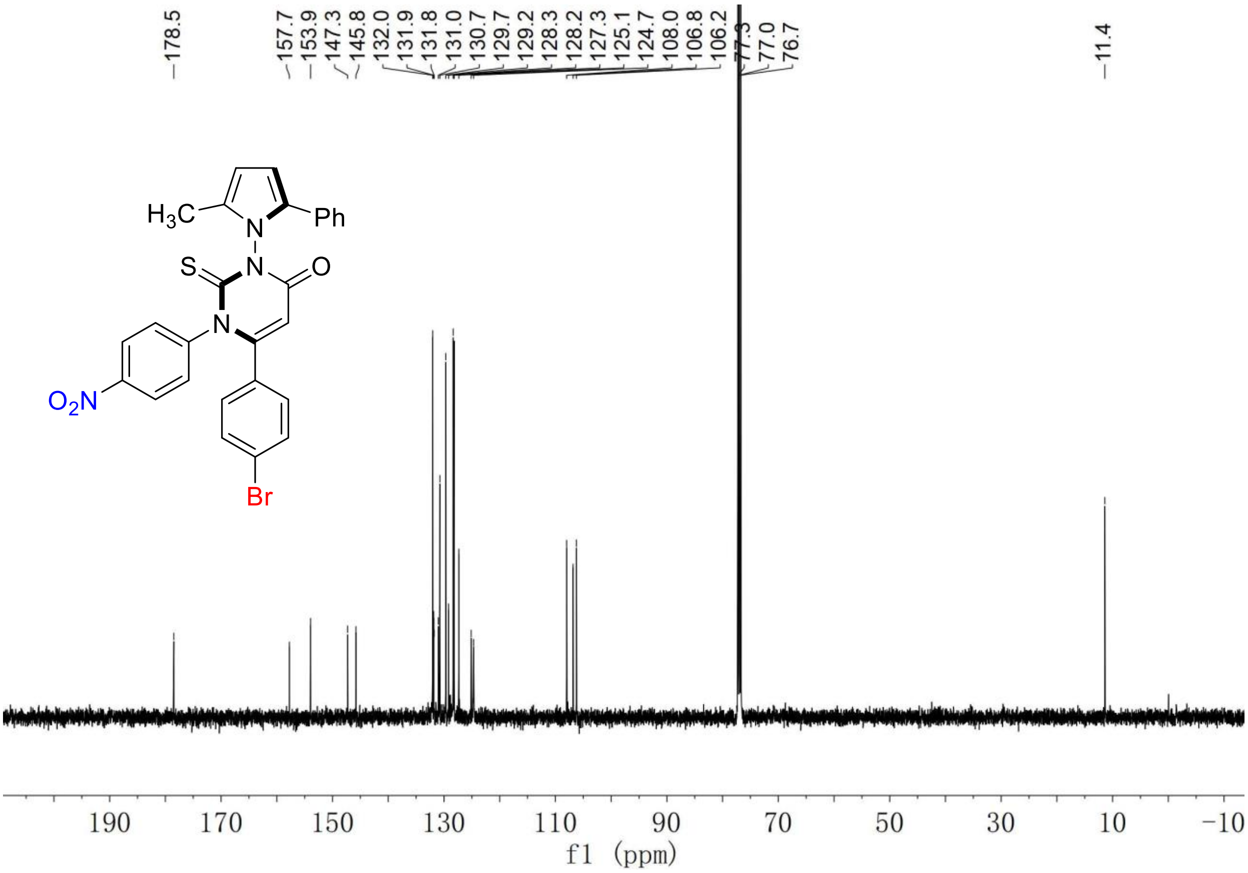

Supplementary Figure 170. ^{13}C NMR spectrum of compound **7v** ($CDCl_3$, 126 MHz, 298 K)

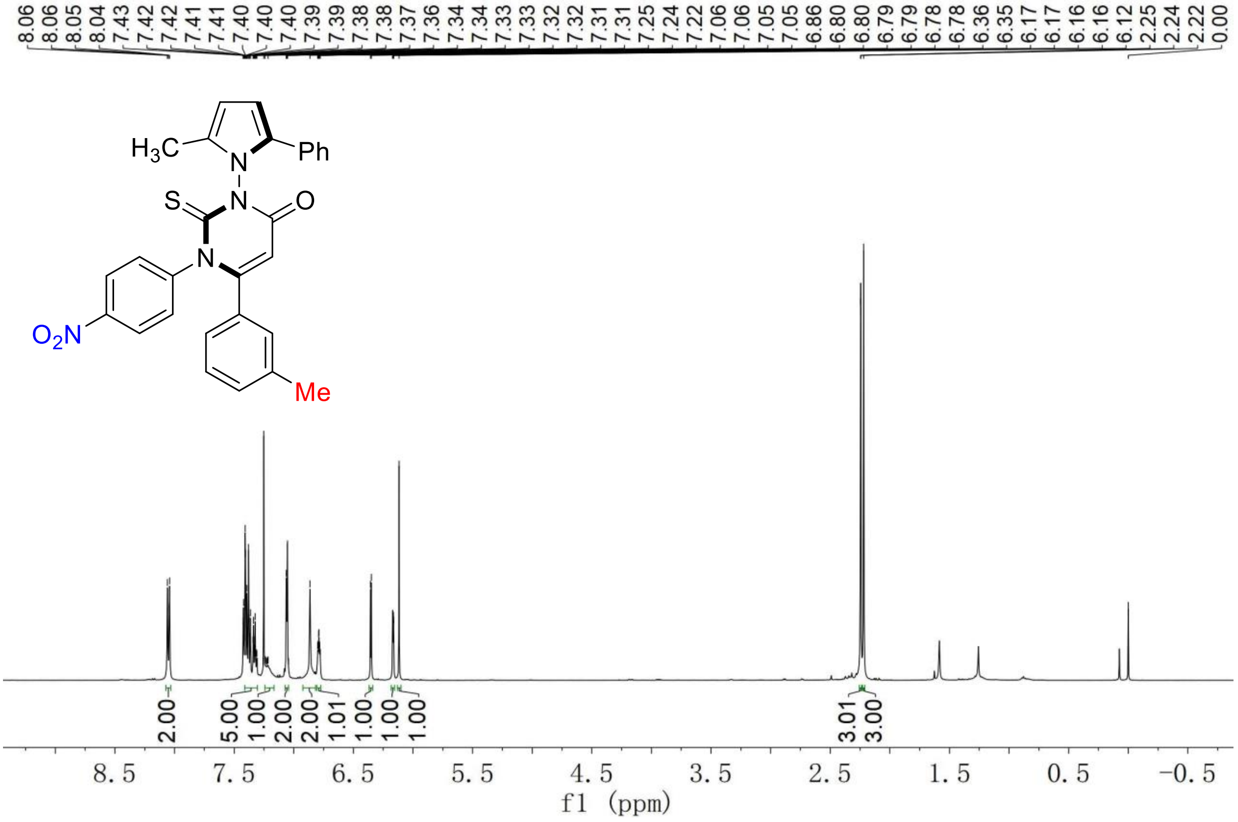

Supplementary Figure 171. ^{1}H NMR spectrum of compound 7w ($CDCl_3$, 500 MHz, 298 K)

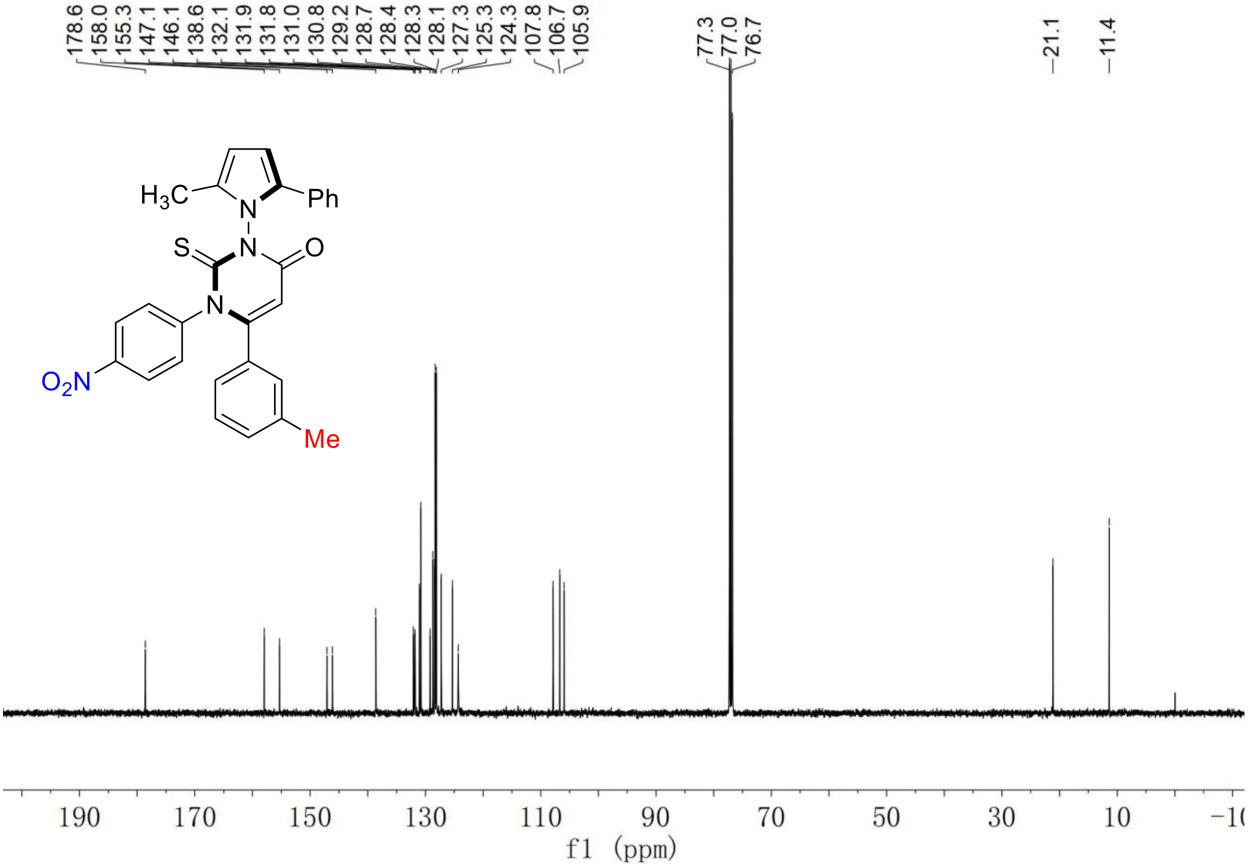

Supplementary Figure 172. ^{13}C NMR spectrum of compound 7w ($CDCl_3$, 126 MHz, 298 K)

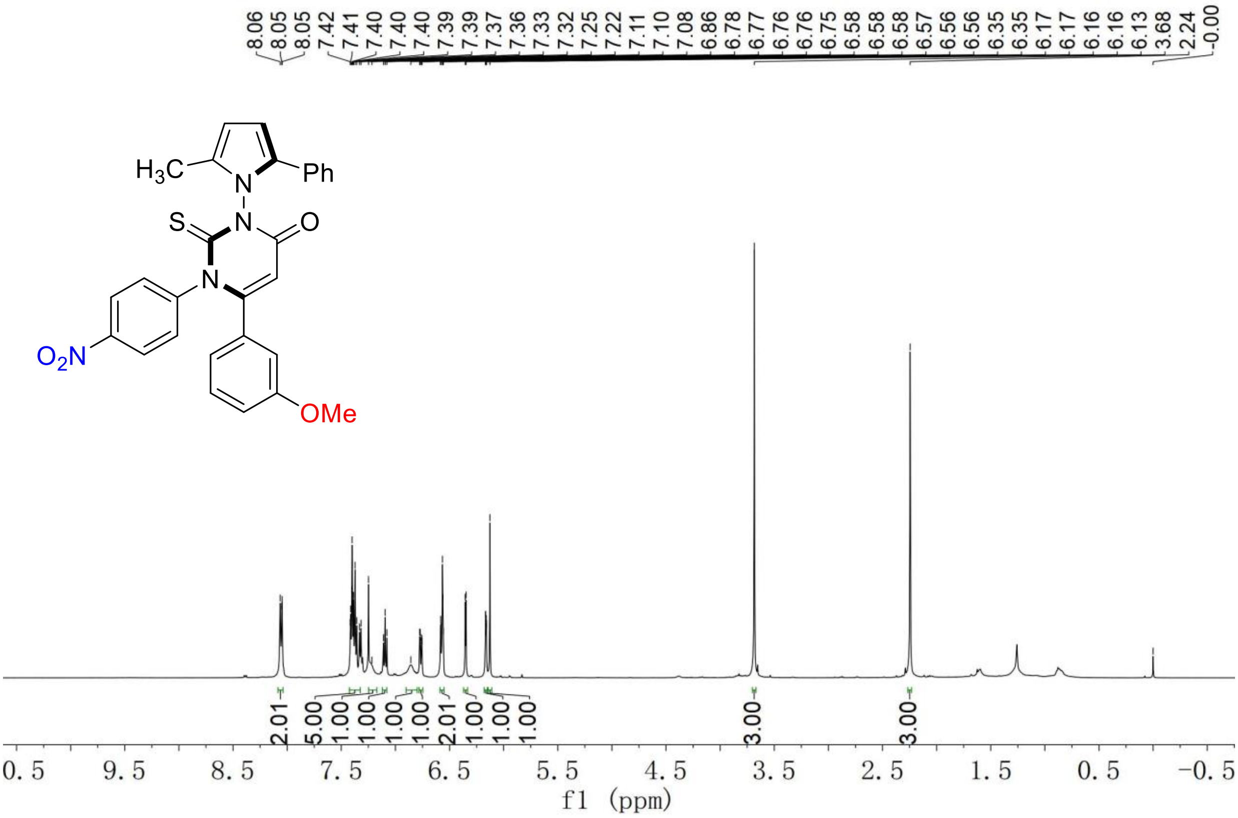

Supplementary Figure 173. ^{1}H NMR spectrum of compound 7x ($CDCl_3$, 500 MHz, 298 K)

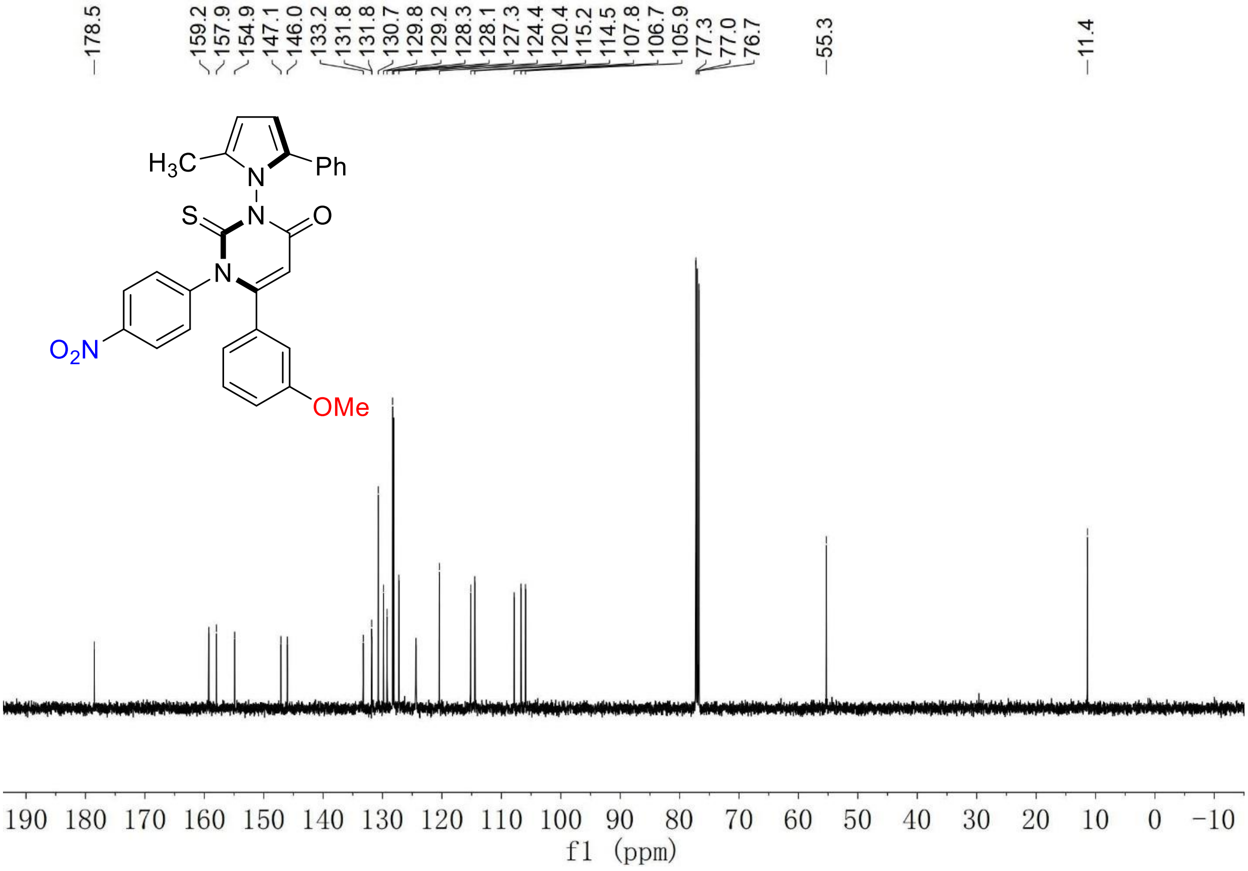

Supplementary Figure 174. ^{13}C NMR spectrum of compound 7x ($CDCl_3$, 126 MHz, 298 K)

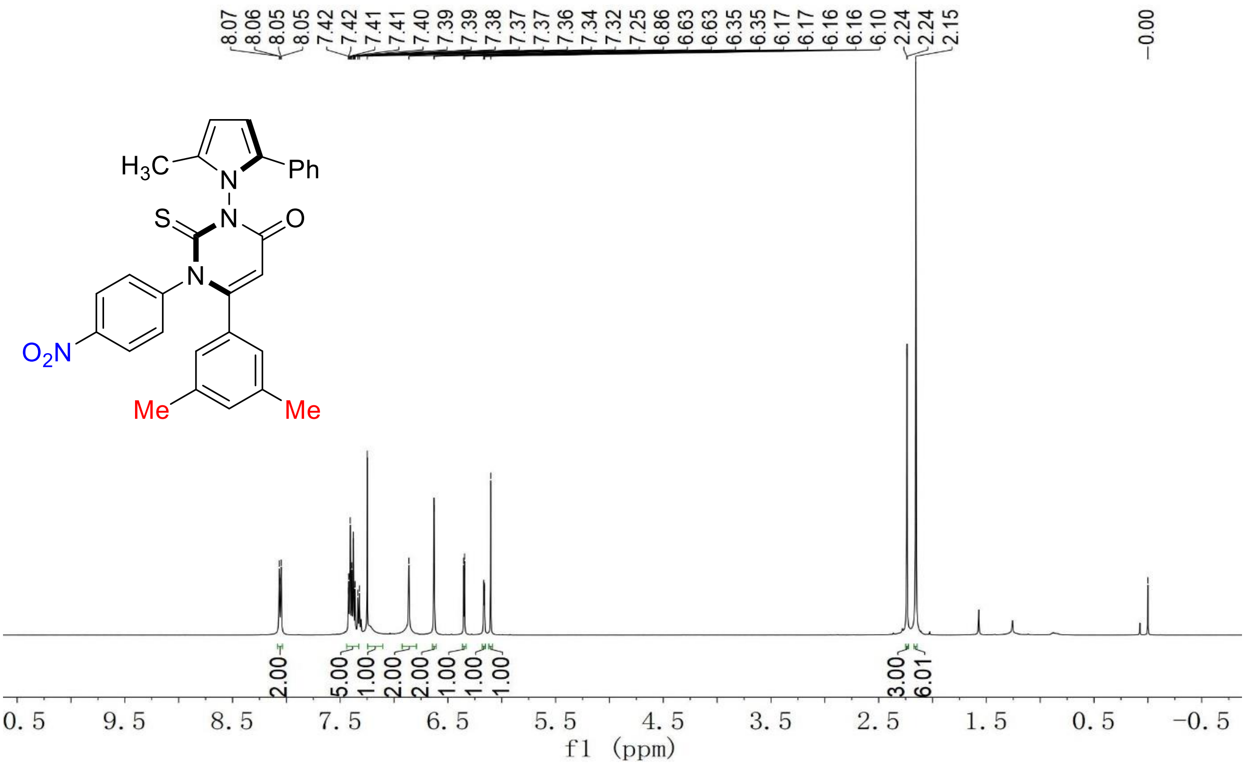

Supplementary Figure 175. ^{1}H NMR spectrum of compound 7y ($CDCl_3$, 500 MHz, 298 K)

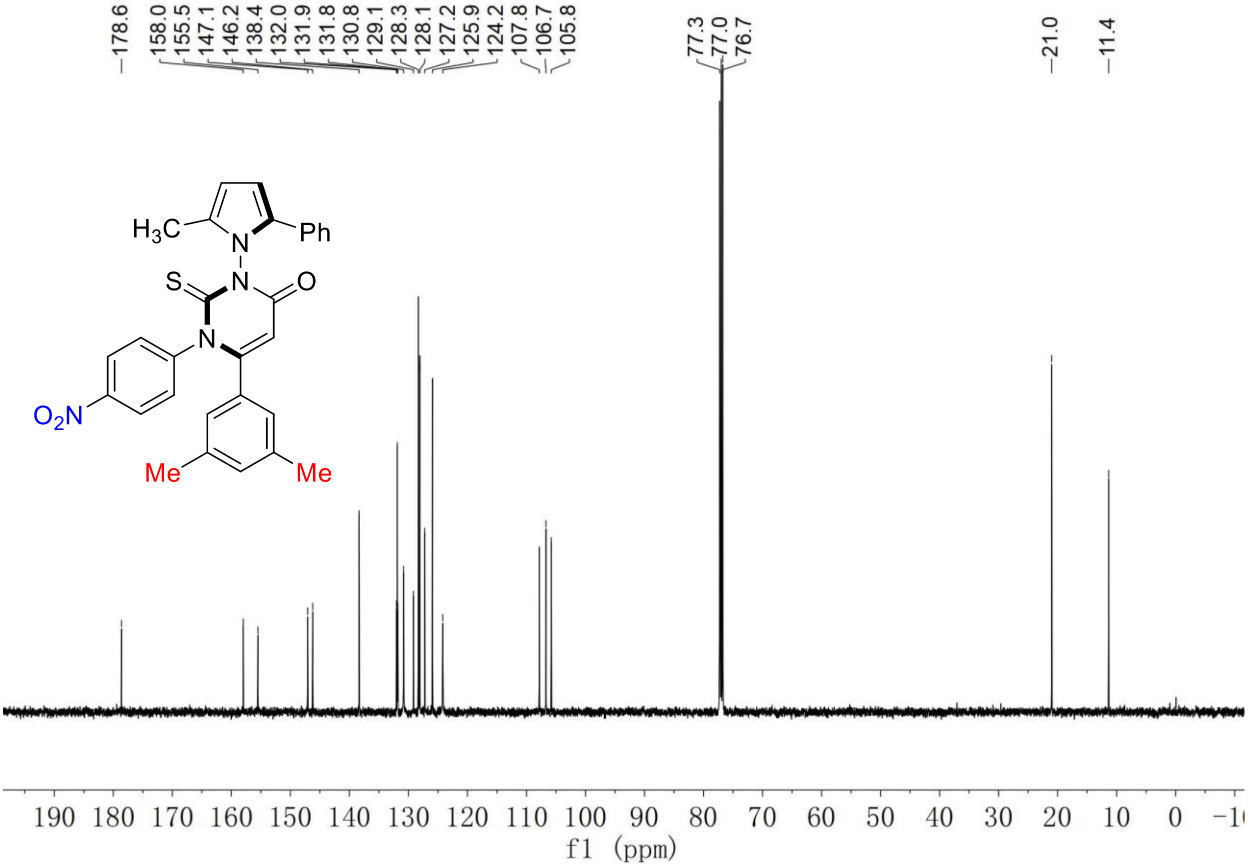

Supplementary Figure 176. ^{13}C NMR spectrum of compound 7y ($CDCl_3$, 126 MHz, 298 K)

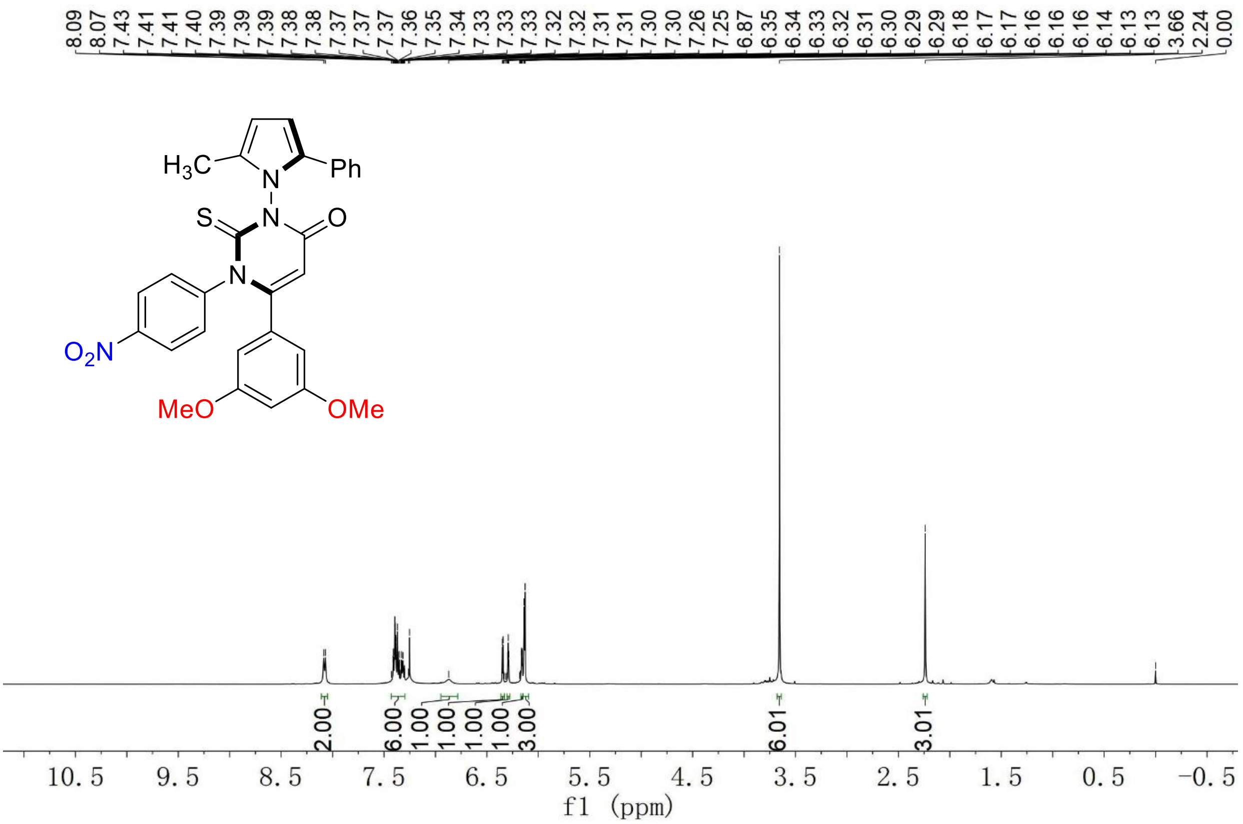

Supplementary Figure 177. **^{1}H NMR spectrum of compound 7z ($CDCl_3$, 500 MHz, 298 K)**

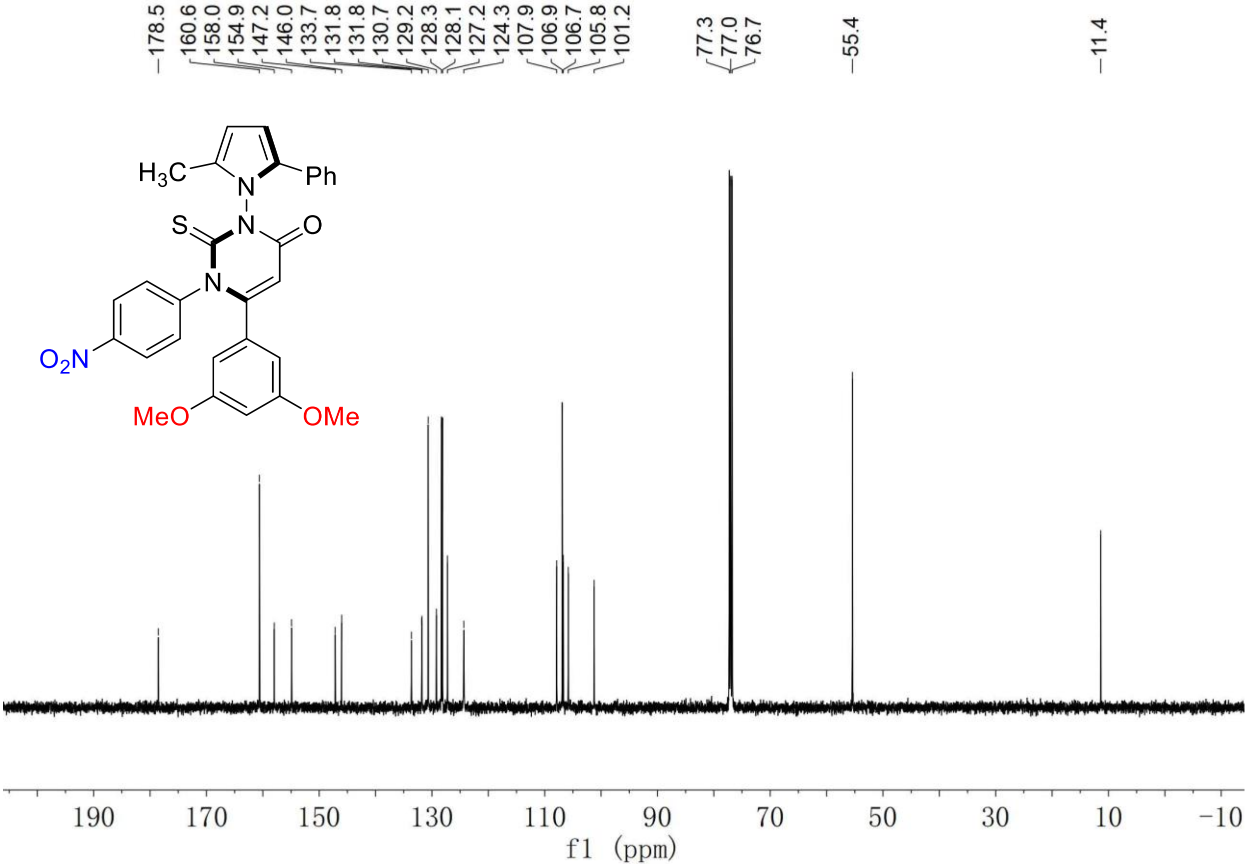

Supplementary Figure 178. **^{13}C NMR spectrum of compound 7z ($CDCl_3$, 126 MHz, 298 K)**

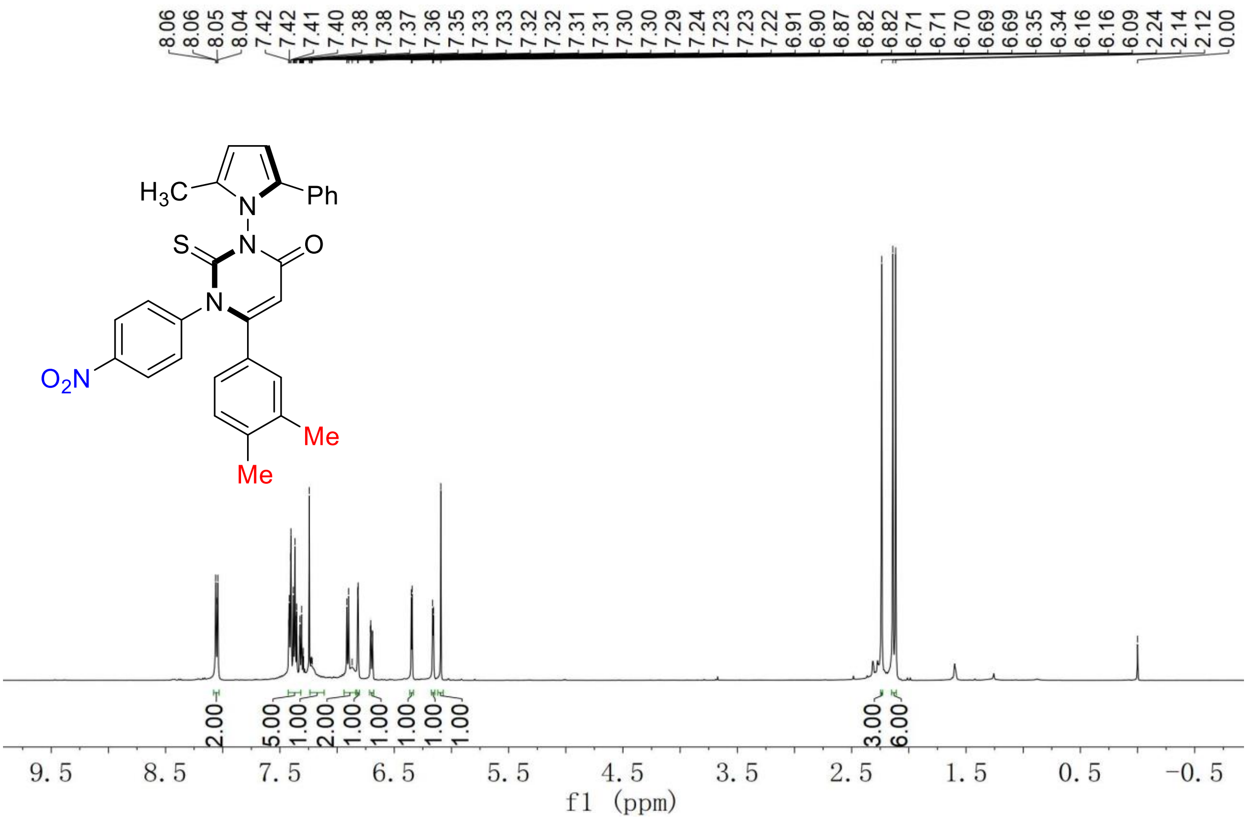

Supplementary Figure 179. ^{1}H NMR spectrum of compound 7aa ($CDCl_3$, 500 MHz, 298 K)

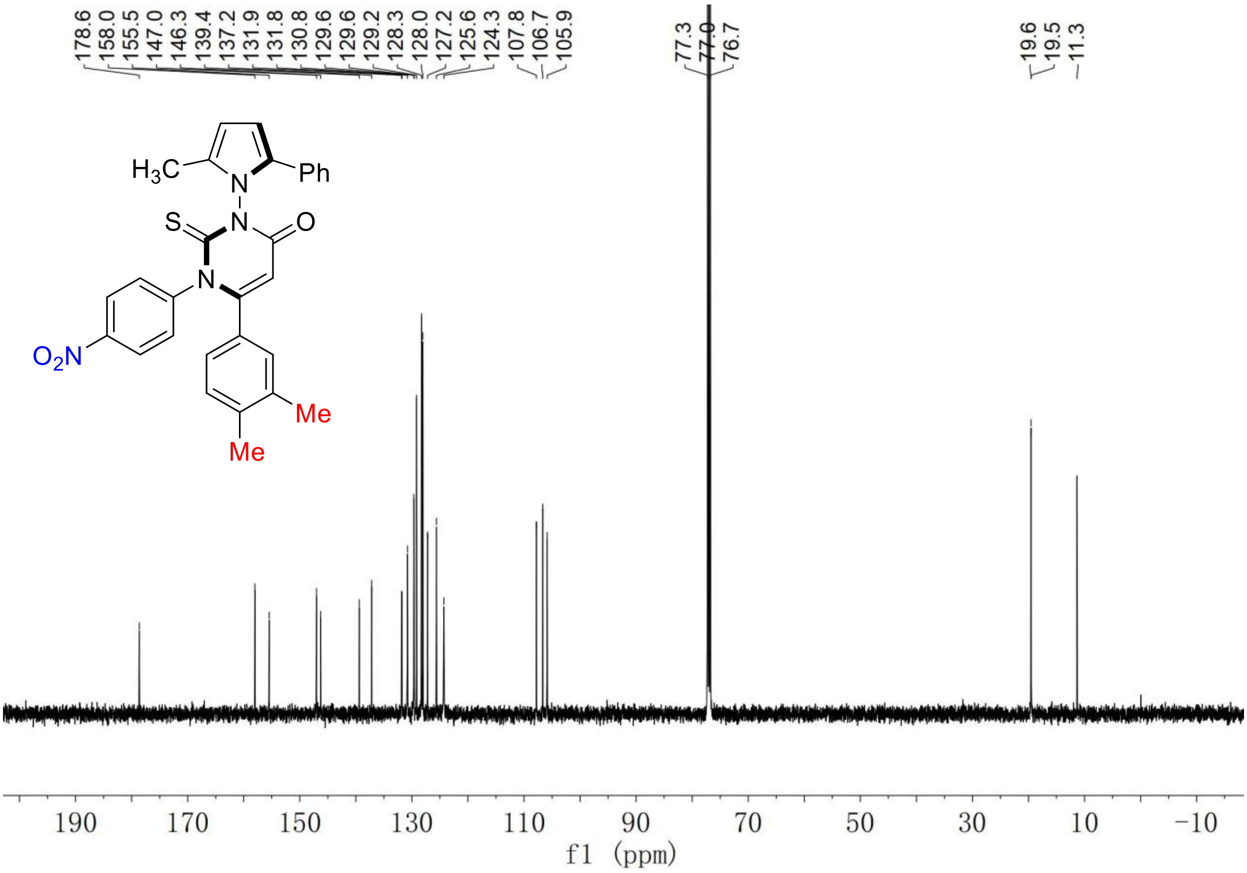

Supplementary Figure 180. ^{13}C NMR spectrum of compound 7aa ($CDCl_3$, 126 MHz, 298 K)

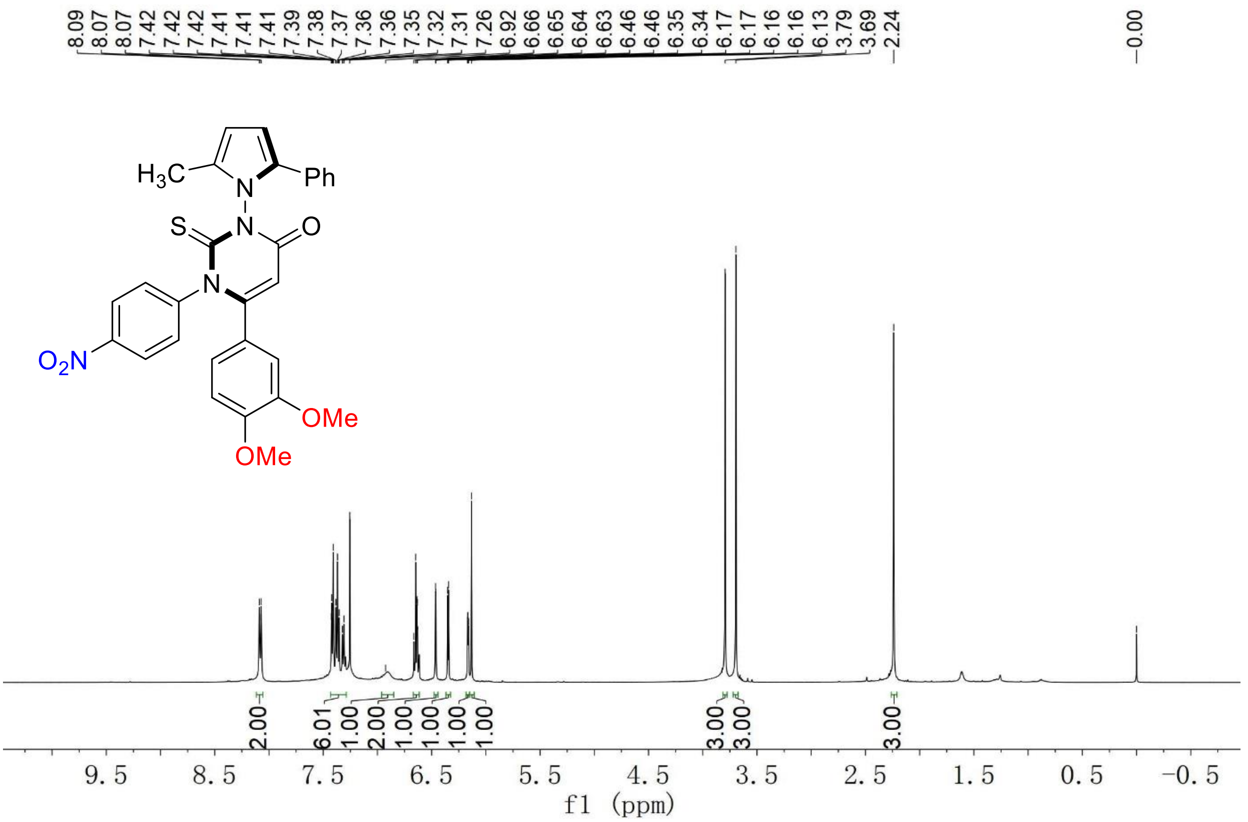

Supplementary Figure 181. ^{1}**H NMR spectrum of compound 7ab (CDCl$_3$, 500 MHz, 298 K)**

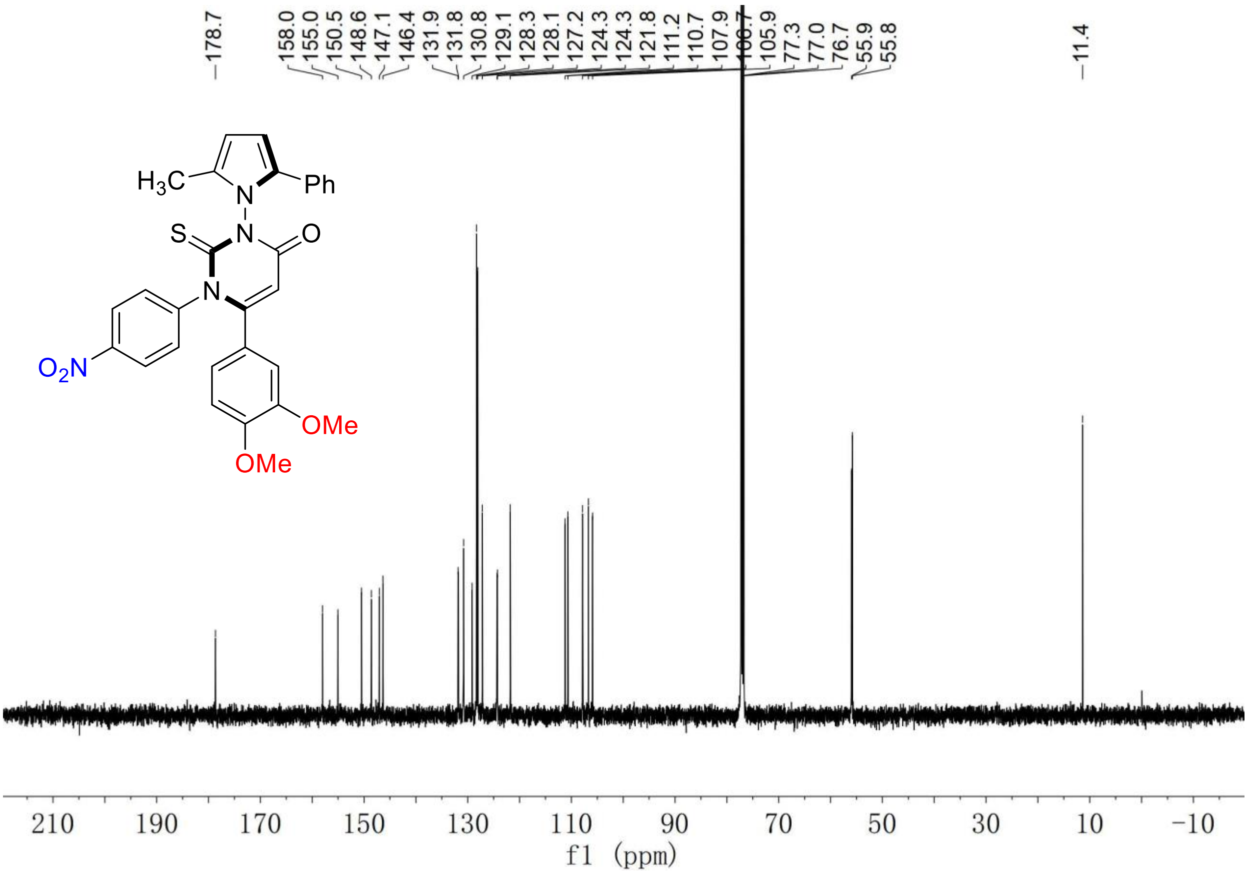

Supplementary Figure 182. ^{13}**C NMR spectrum of compound 7ab (CDCl$_3$, 126 MHz, 298 K)**

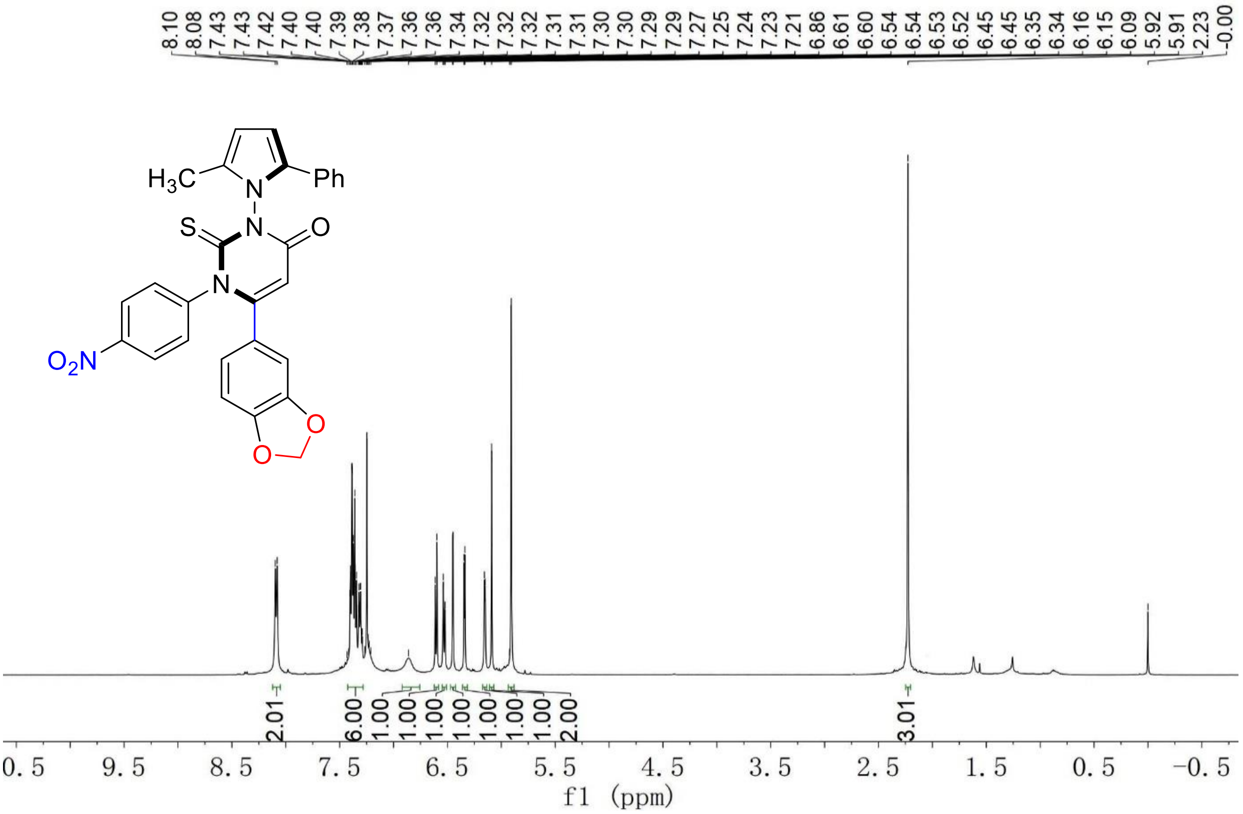

Supplementary Figure 183. ^{1}H NMR spectrum of compound 7ac ($CDCl_3$, 500 MHz, 298 K)

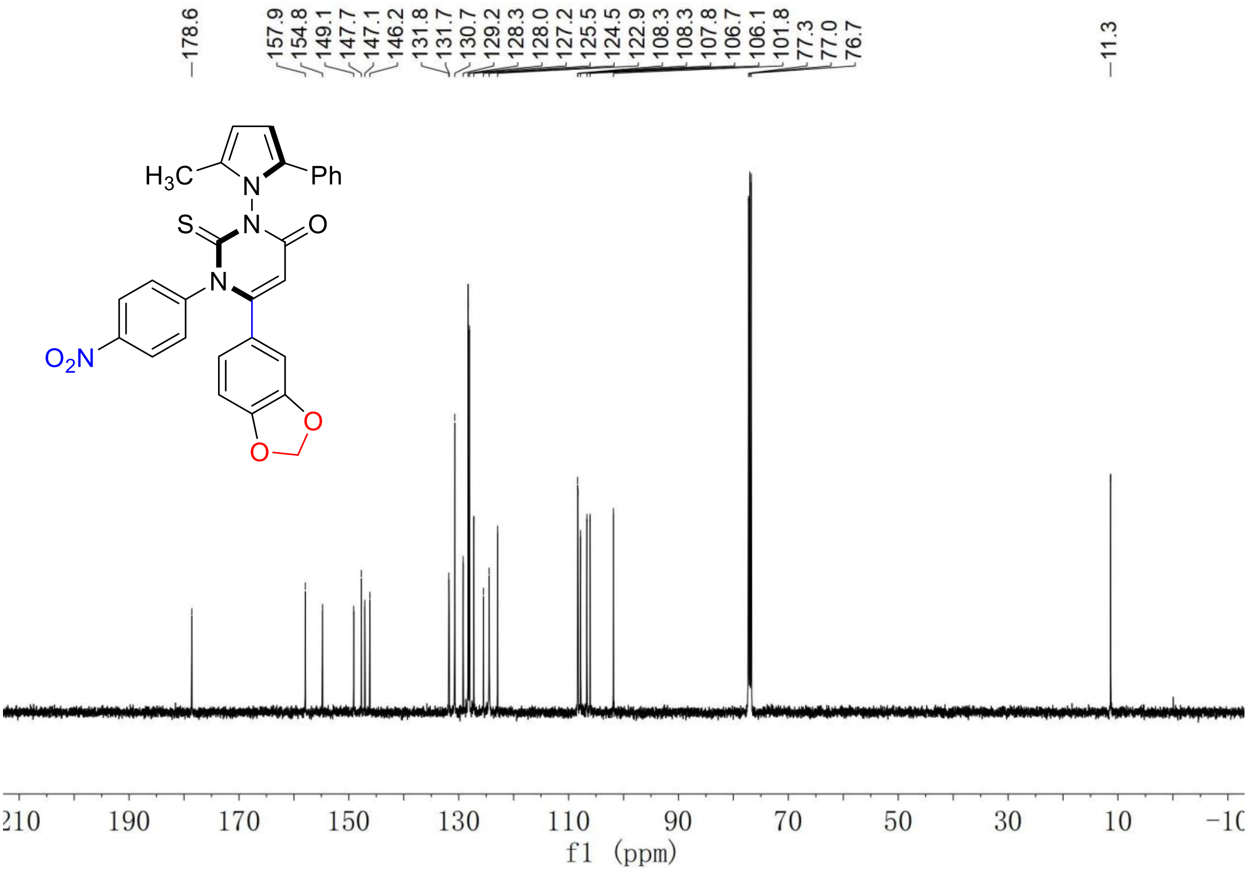

Supplementary Figure 184. ^{13}C NMR spectrum of compound 7ac ($CDCl_3$, 126 MHz, 298 K)

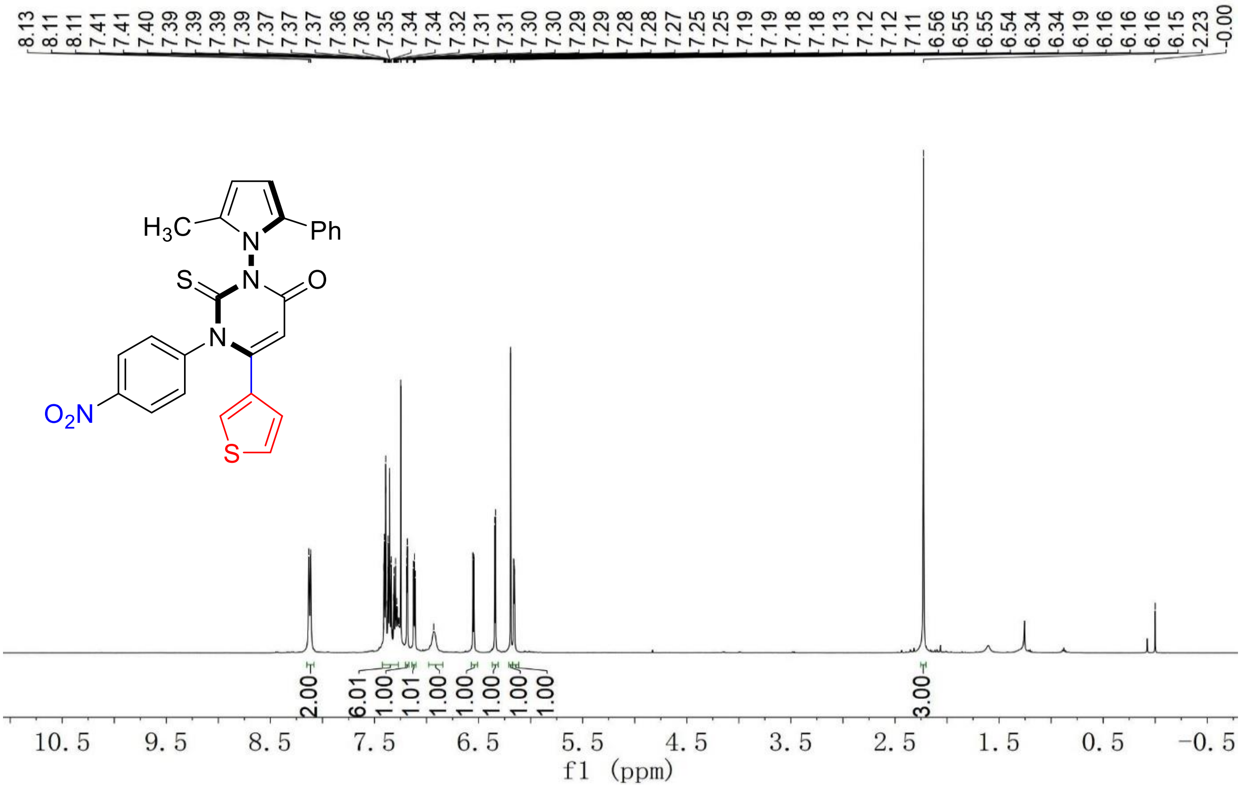

Supplementary Figure 185. ^{1}H NMR spectrum of compound 7ad ($CDCl_3$, 500 MHz, 298 K)

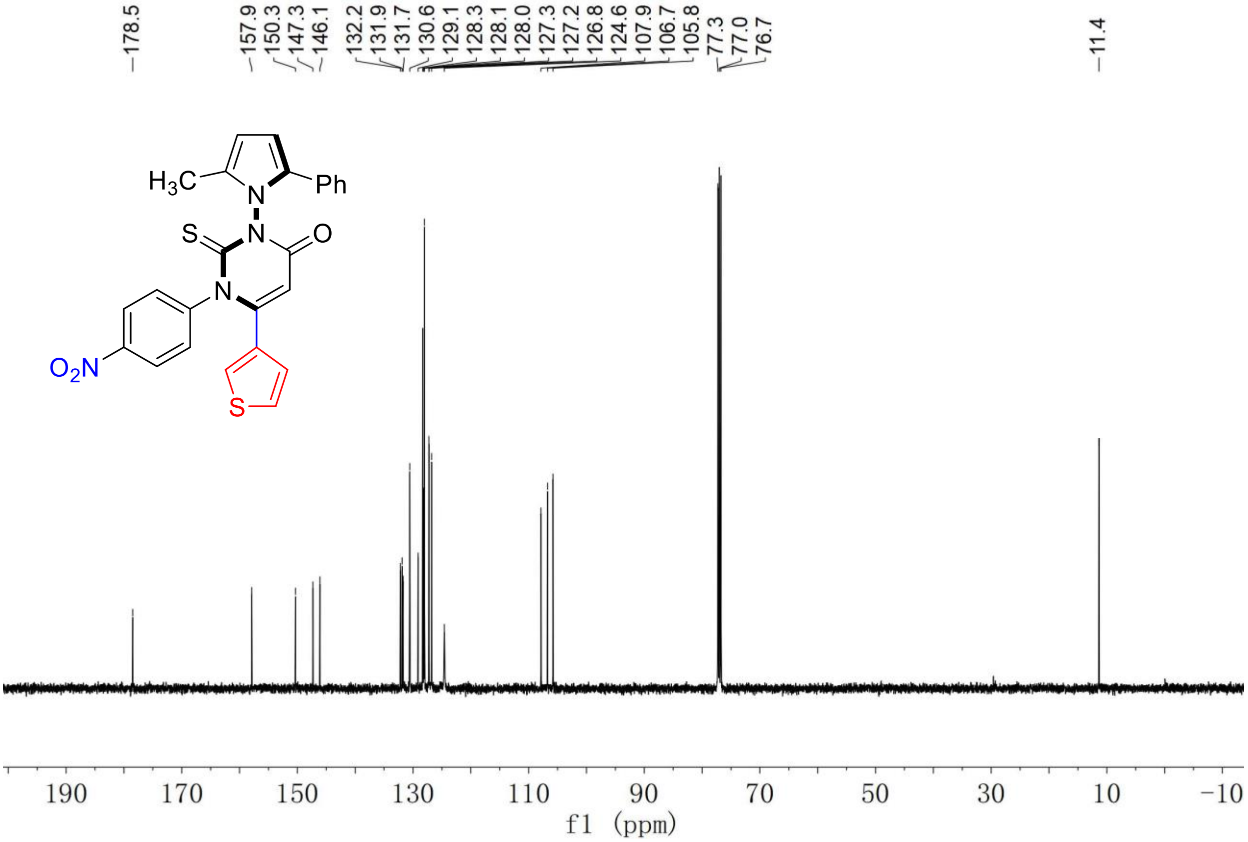

Supplementary Figure 186. ^{13}C NMR spectrum of compound 7ad ($CDCl_3$, 126 MHz, 298 K)

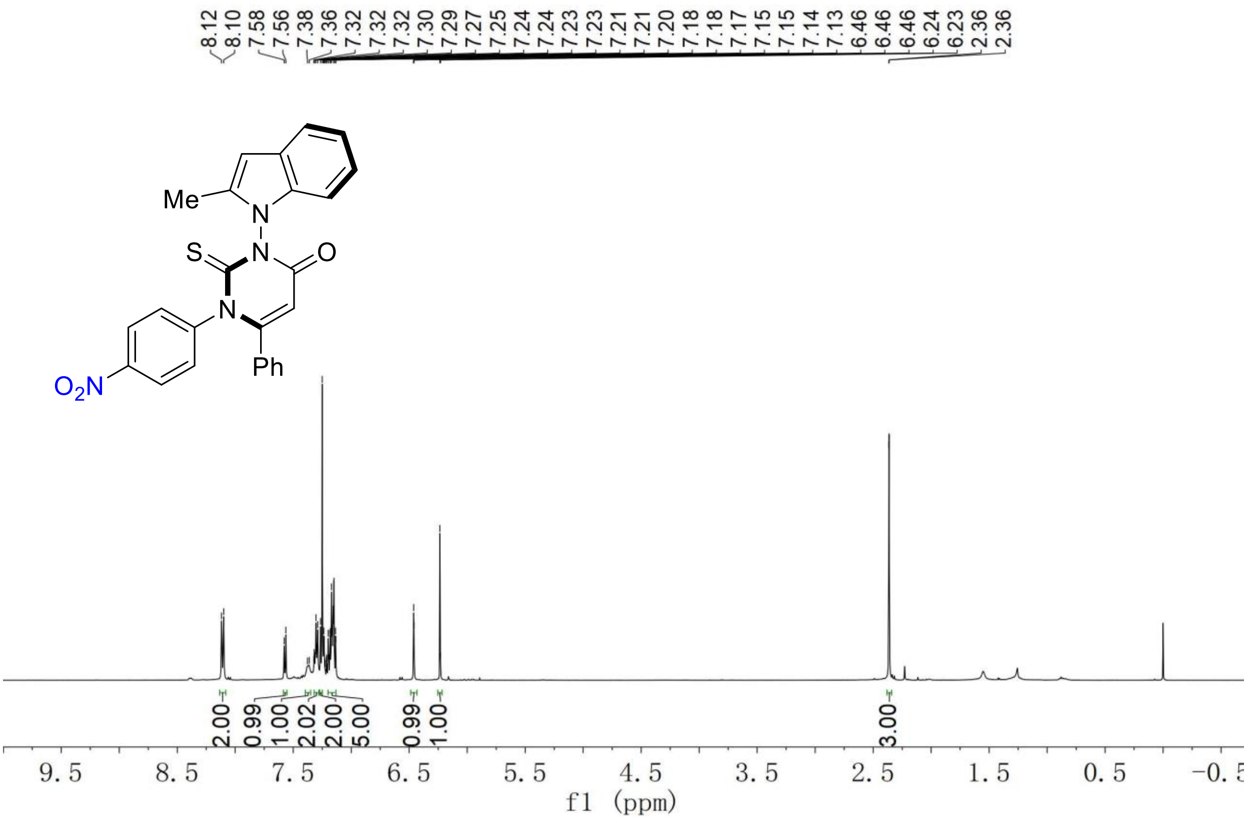

Supplementary Figure 187. ^{1}H NMR spectrum of compound 7ae ($CDCl_3$, 500 MHz, 298 K)

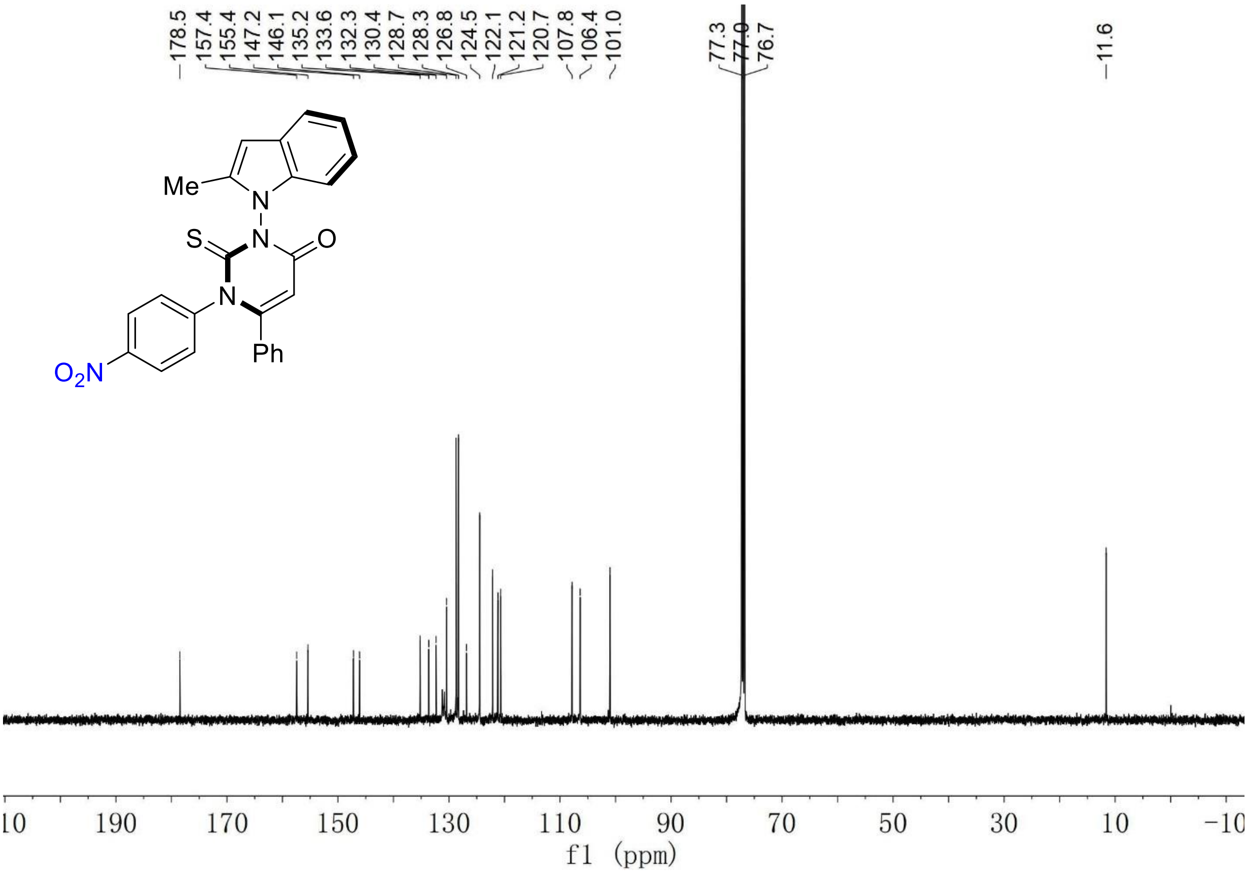

Supplementary Figure 188. ^{13}C NMR spectrum of compound 7ae ($CDCl_3$, 126 MHz, 298 K)

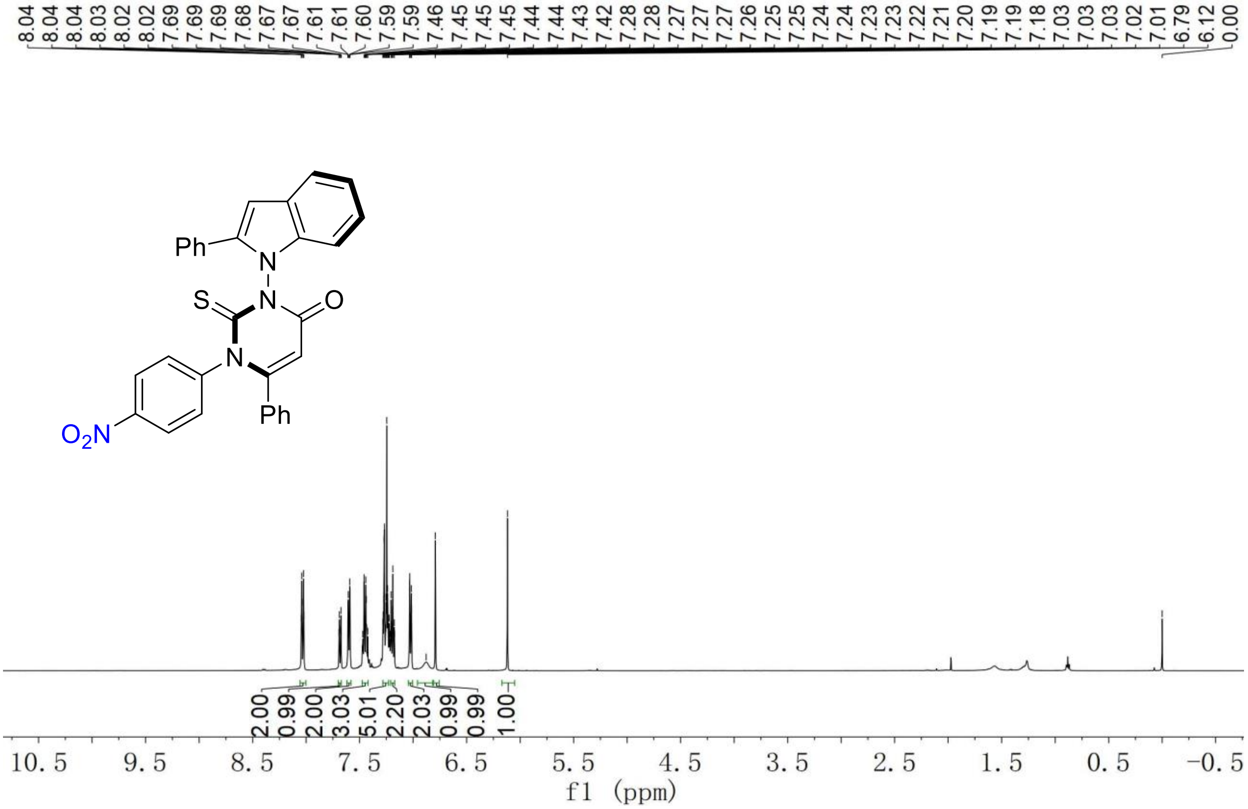

Supplementary Figure 189. ^{1}H NMR spectrum of compound 7af ($CDCl_3$, 500 MHz, 298 K)

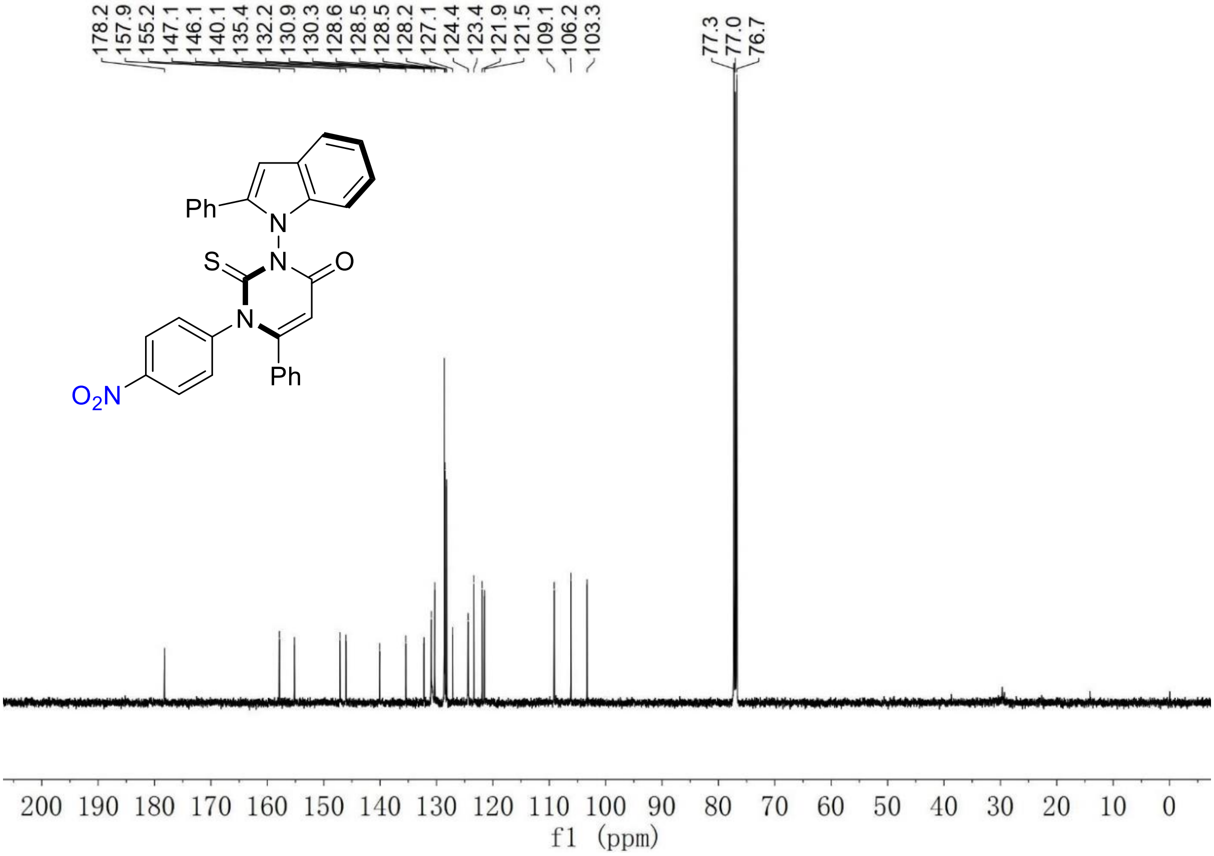

Supplementary Figure 190. ^{13}C NMR spectrum of compound 7af ($CDCl_3$, 126 MHz, 298 K)

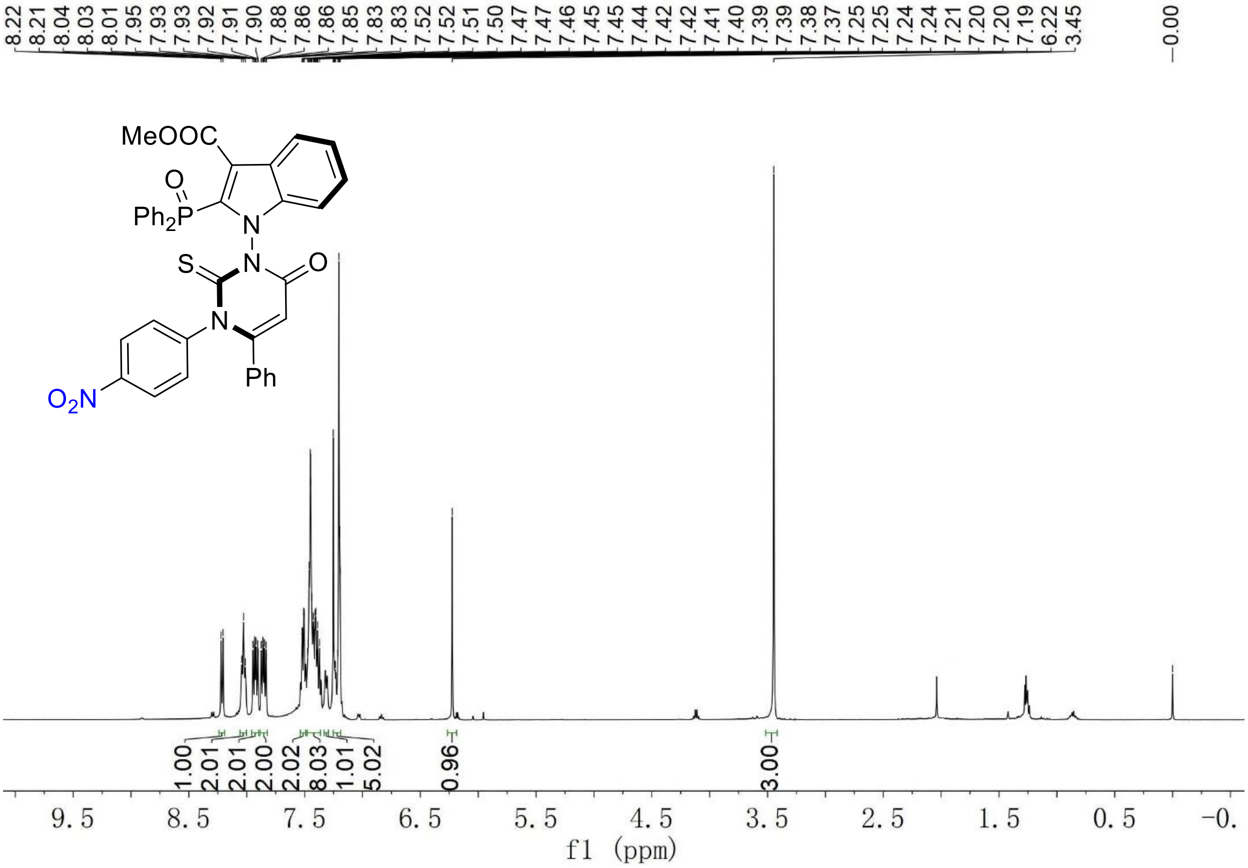

Supplementary Figure 191. ^{1}H NMR spectrum of compound 7ag ($CDCl_3$, 500 MHz, 298 K)

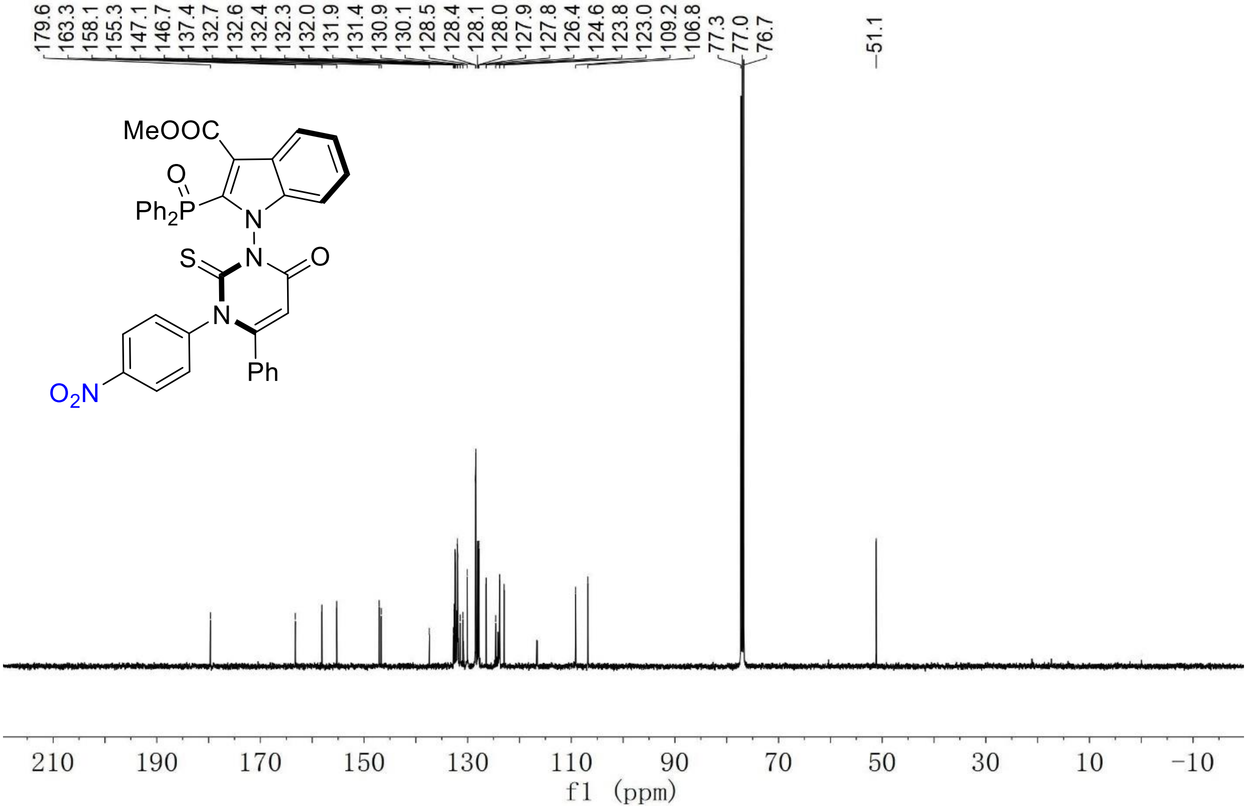

Supplementary Figure 192. ^{13}C NMR spectrum of compound 7ag ($CDCl_3$, 126 MHz, 298 K)

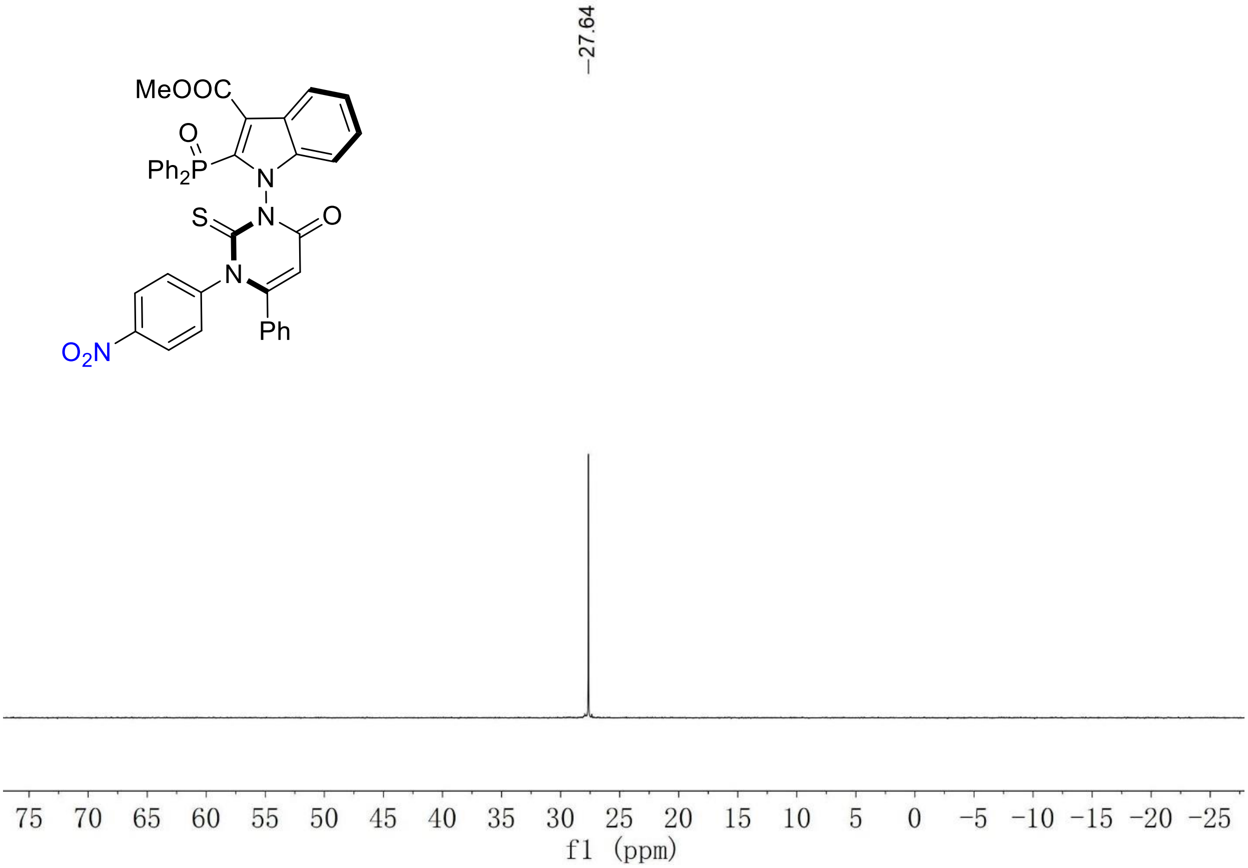

Supplementary Figure 193. ^{31}**P NMR spectrum of compound 7ag (CDCl$_3$, 202 MHz, 298 K)**

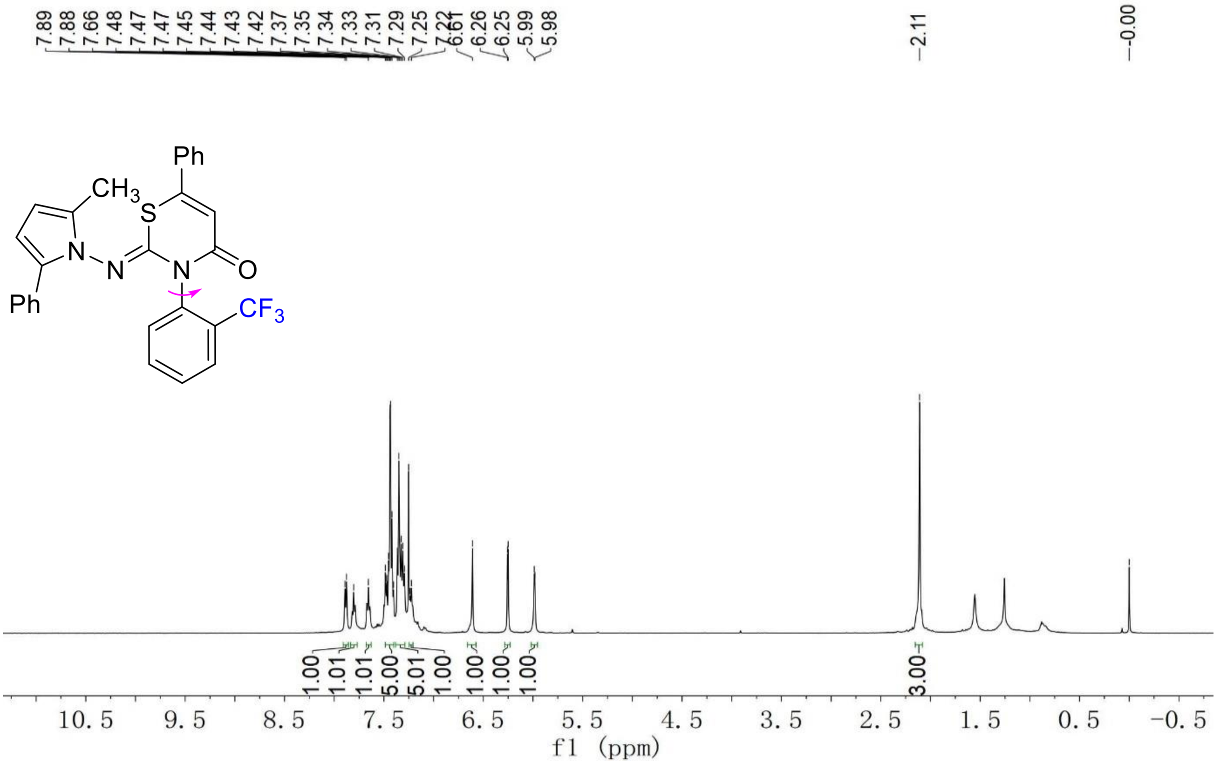

Supplementary Figure 194. **^{1}H NMR spectrum of compound 9a ($CDCl_3$, 500 MHz, 298 K)**

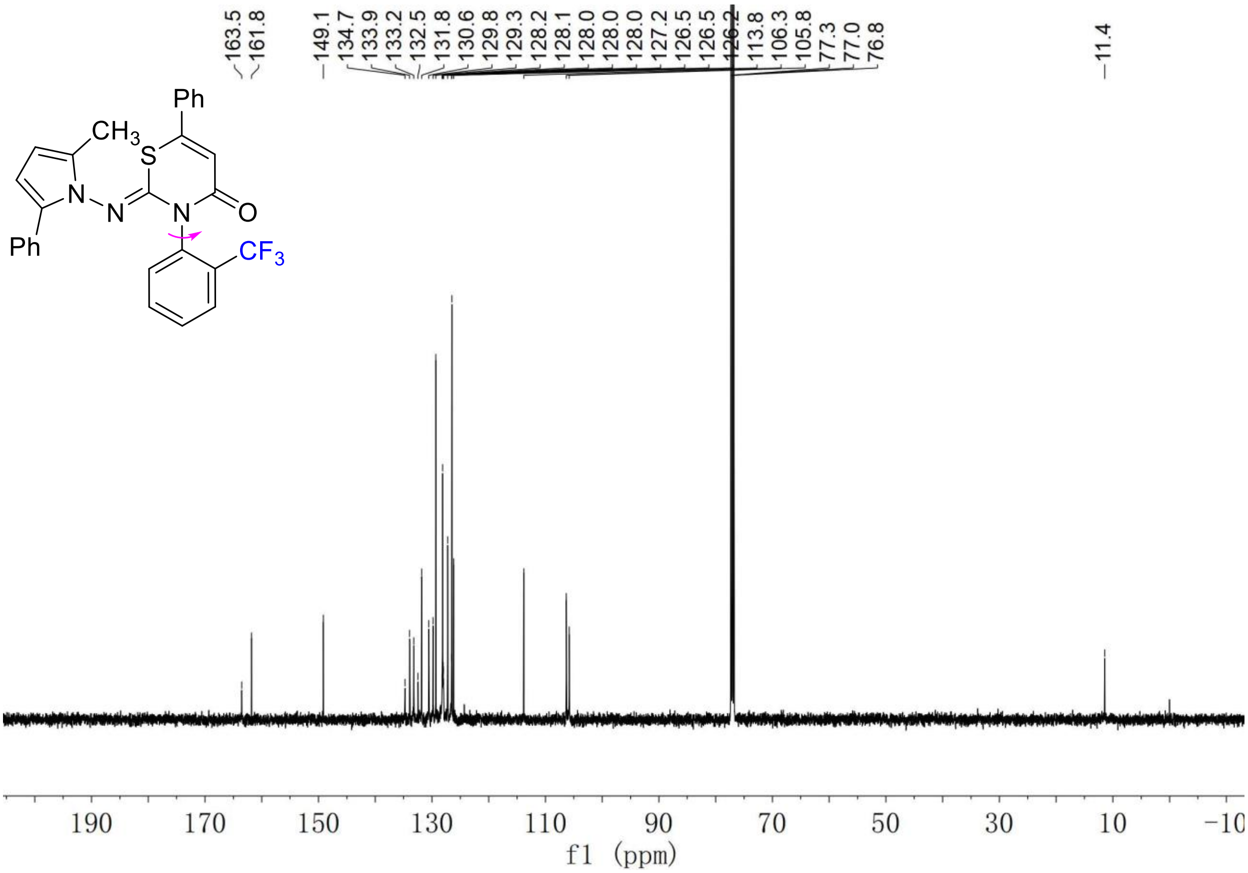

Supplementary Figure 195. **^{13}C NMR spectrum of compound 9a ($CDCl_3$, 126 MHz, 298 K)**

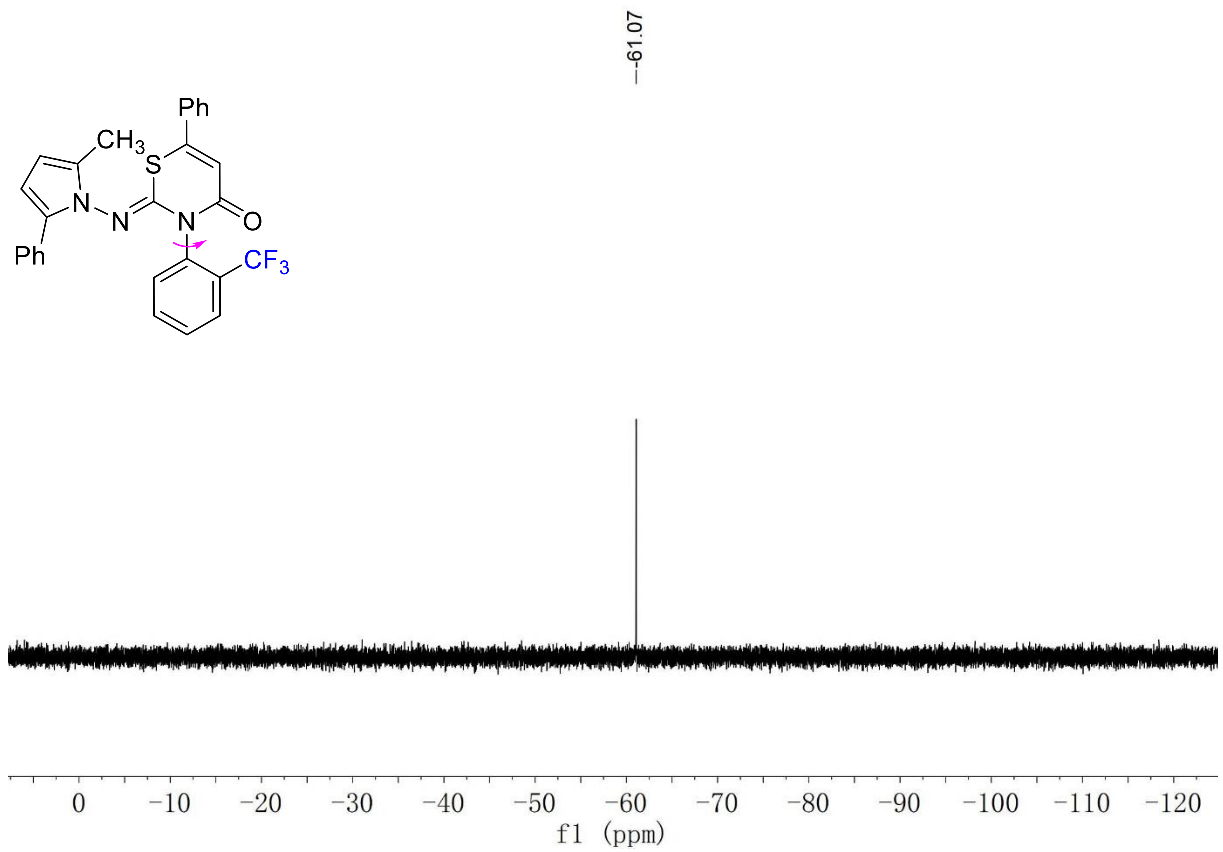

Supplementary Figure 196. ^{19}**F NMR spectrum of compound 9a (CDCl$_3$, 471 MHz, 298 K)**

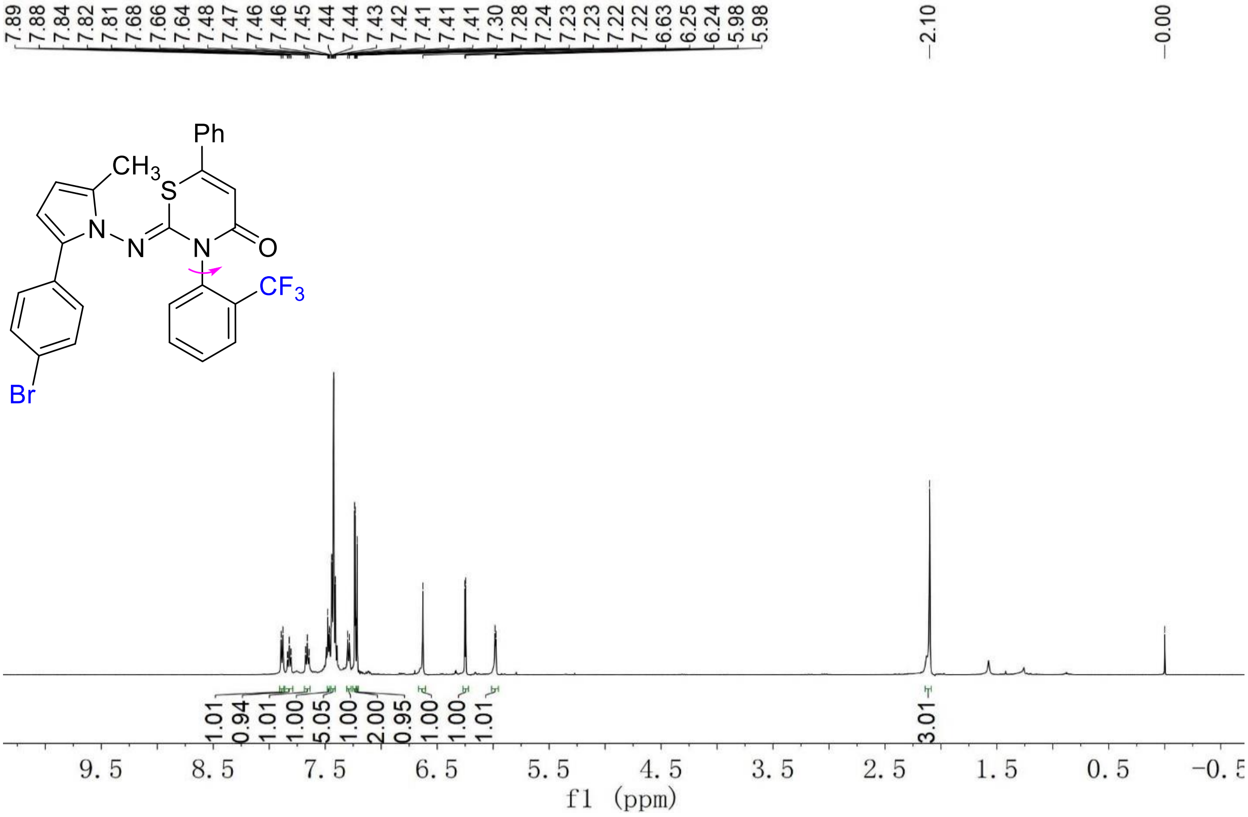

Supplementary Figure 197. ^{1}H NMR spectrum of compound 9b ($CDCl_3$, 500 MHz, 298 K)

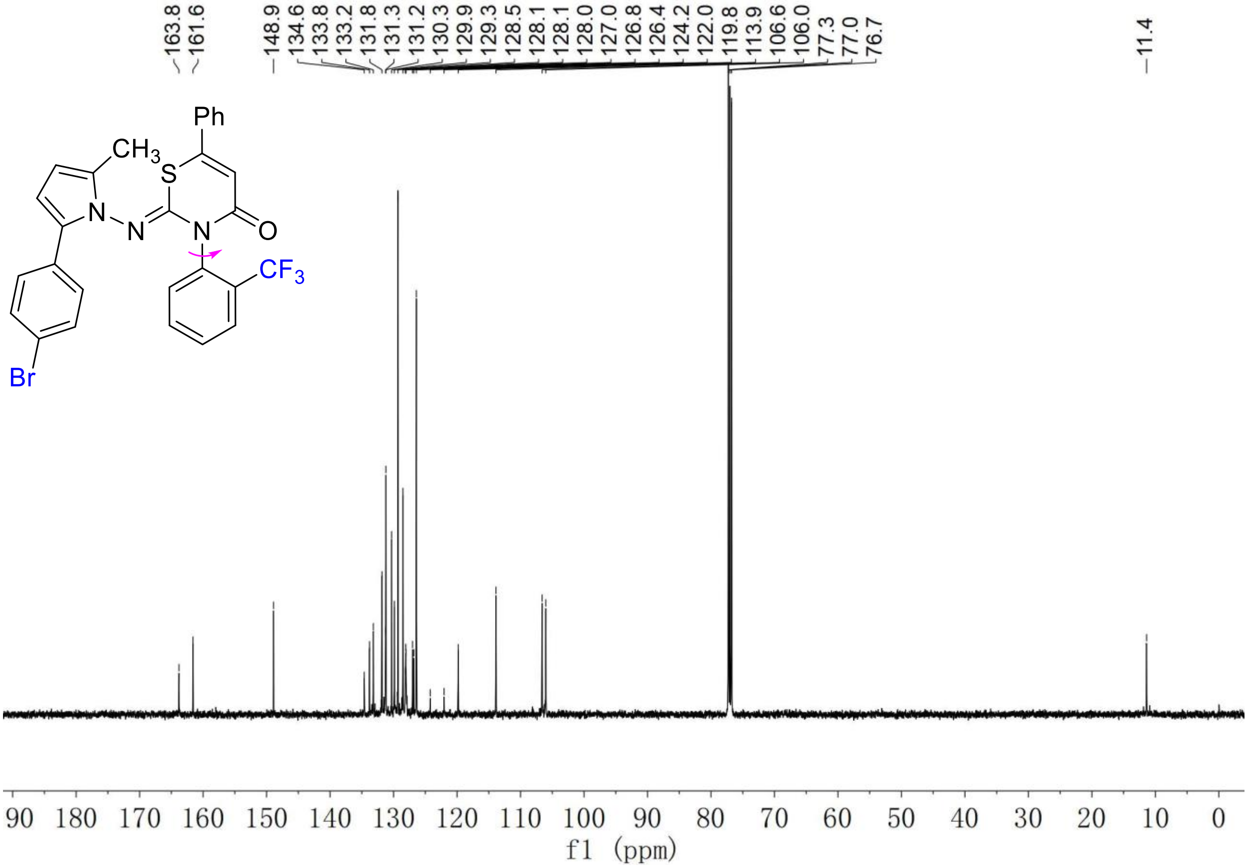

Supplementary Figure 198. ^{13}C NMR spectrum of compound 9b ($CDCl_3$, 126 MHz, 298 K)

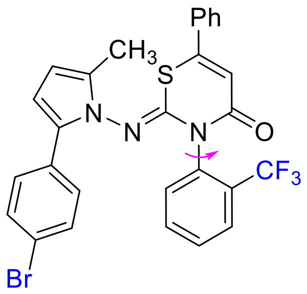

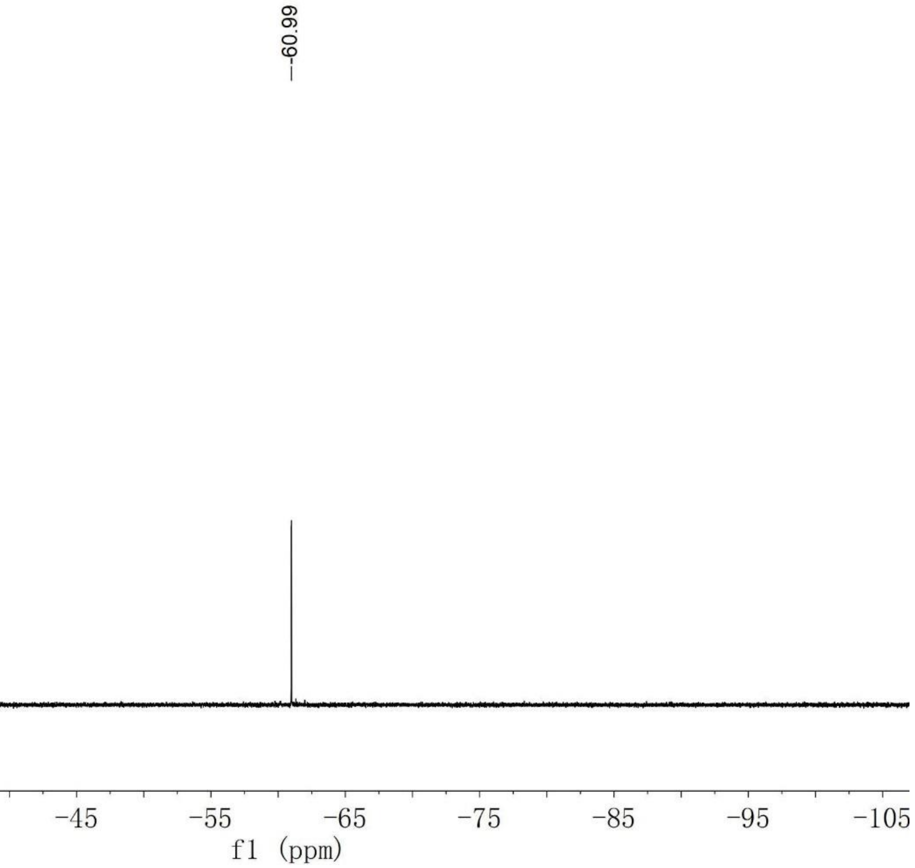

Supplementary Figure 199. ^{19}F NMR spectrum of compound 9b ($CDCl_3$, 471 MHz, 298 K)

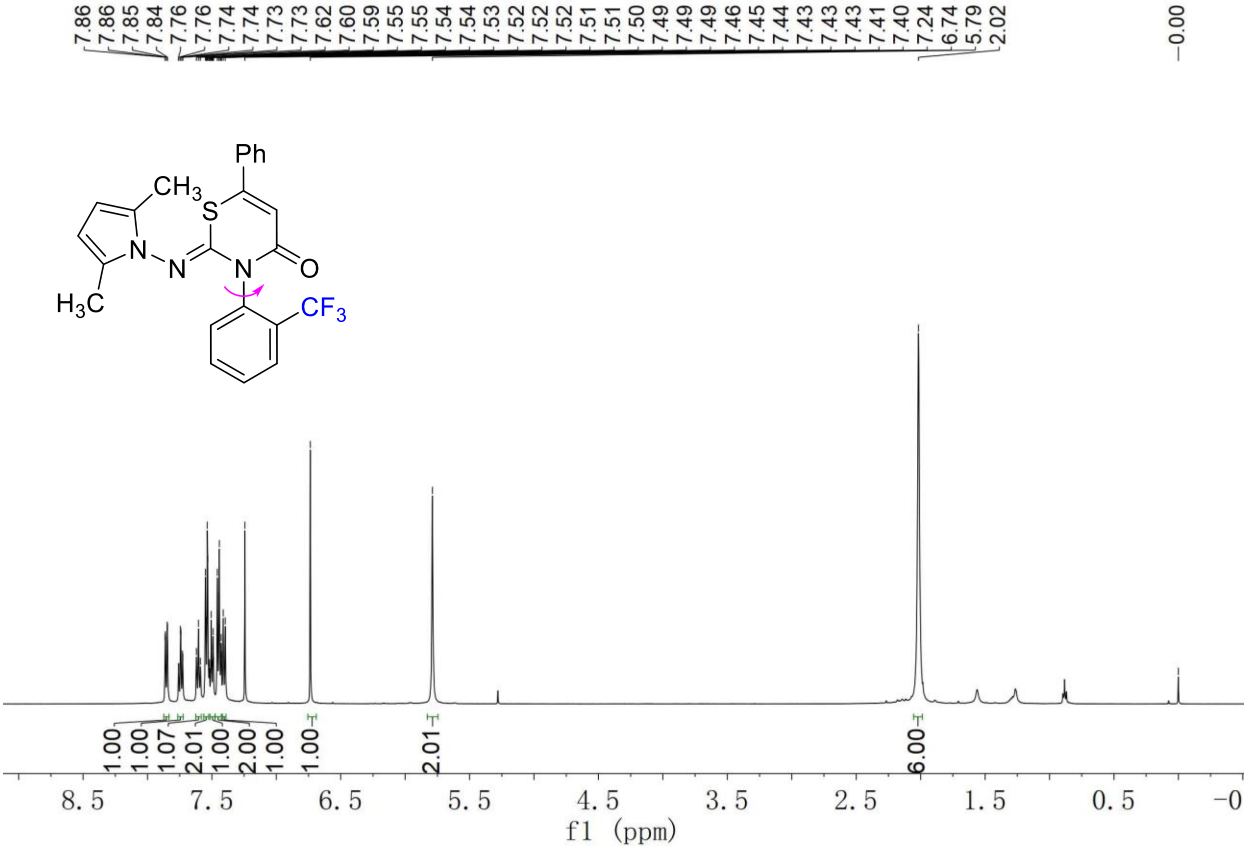

Supplementary Figure 200. **^{1}H NMR spectrum of compound 9c ($CDCl_3$, 500 MHz, 298 K)**

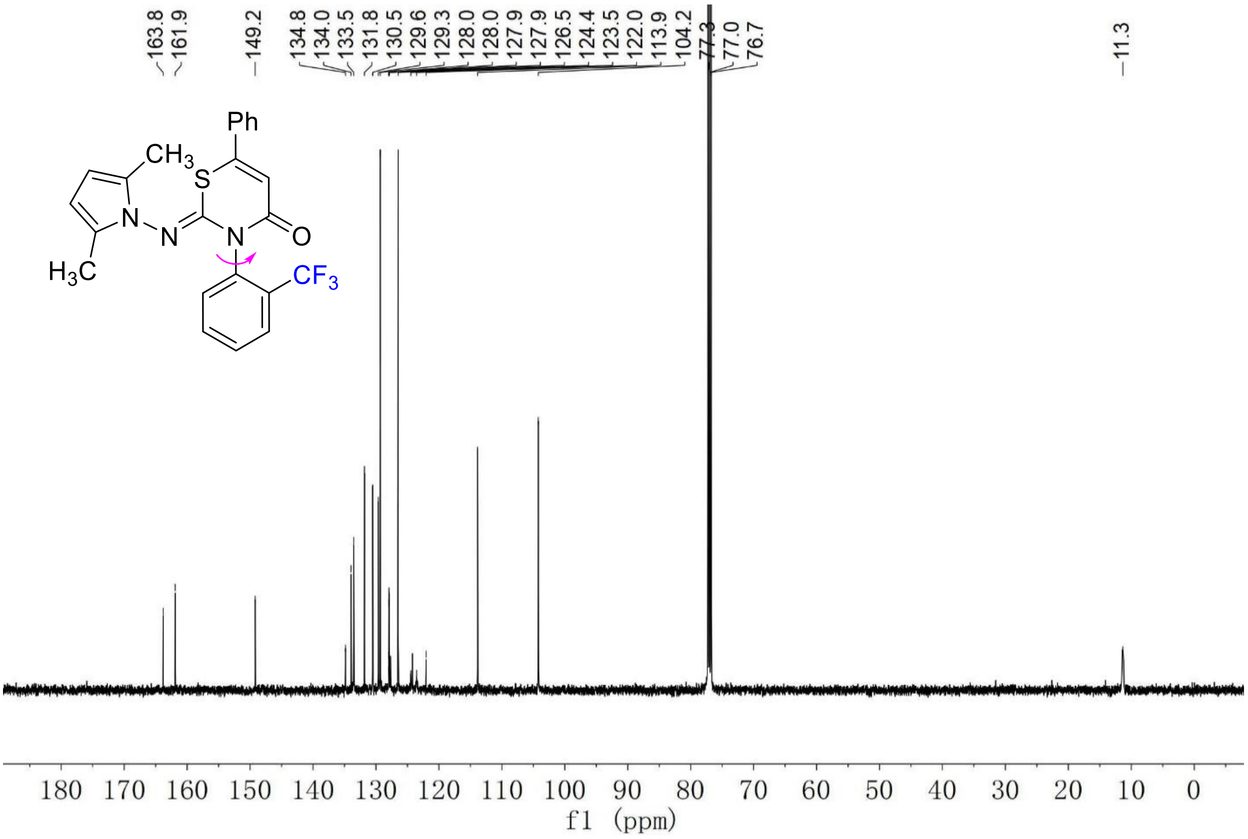

Supplementary Figure 201. **^{13}C NMR spectrum of compound 9c ($CDCl_3$, 126 MHz, 298 K)**

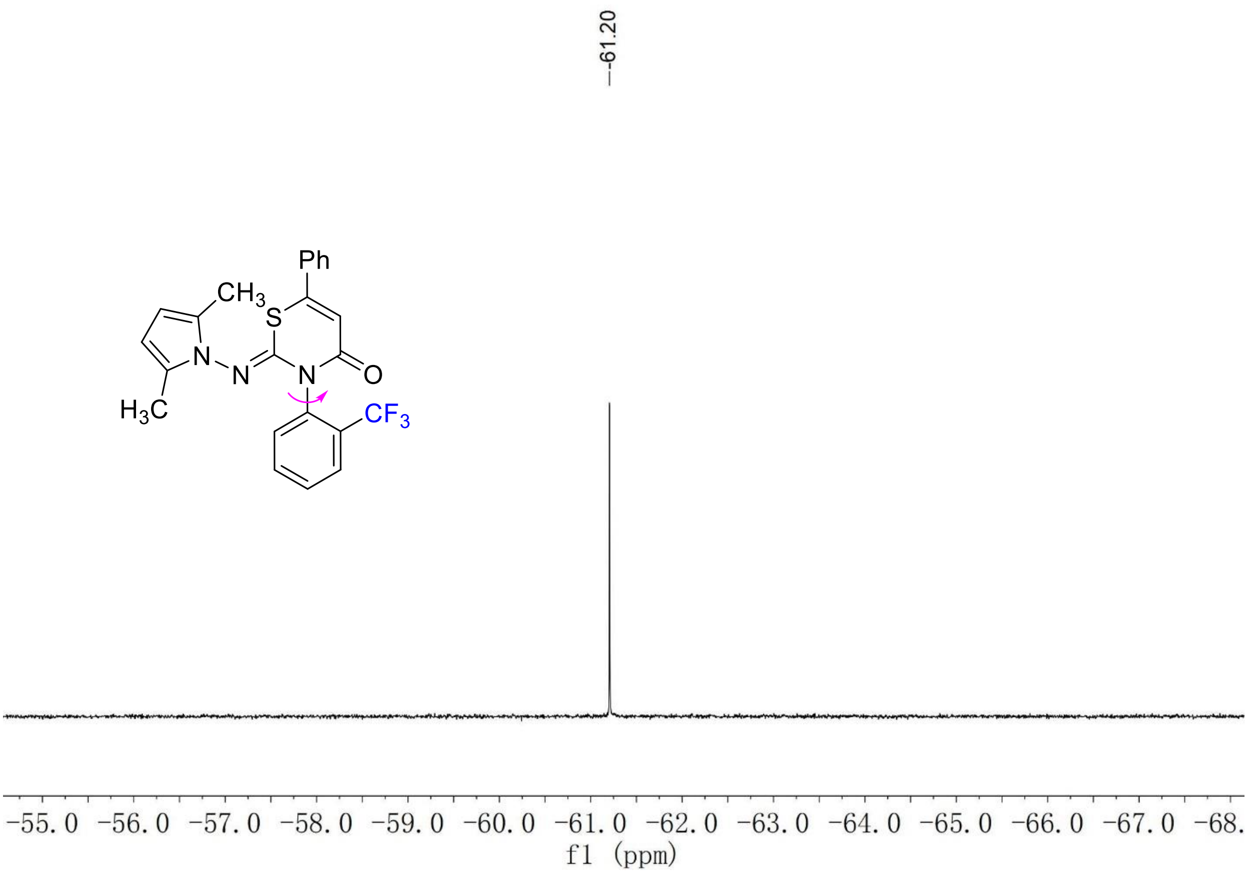

Supplementary Figure 202. ^{19}F NMR spectrum of compound 9c ($CDCl_3$, 471 MHz, 298 K)

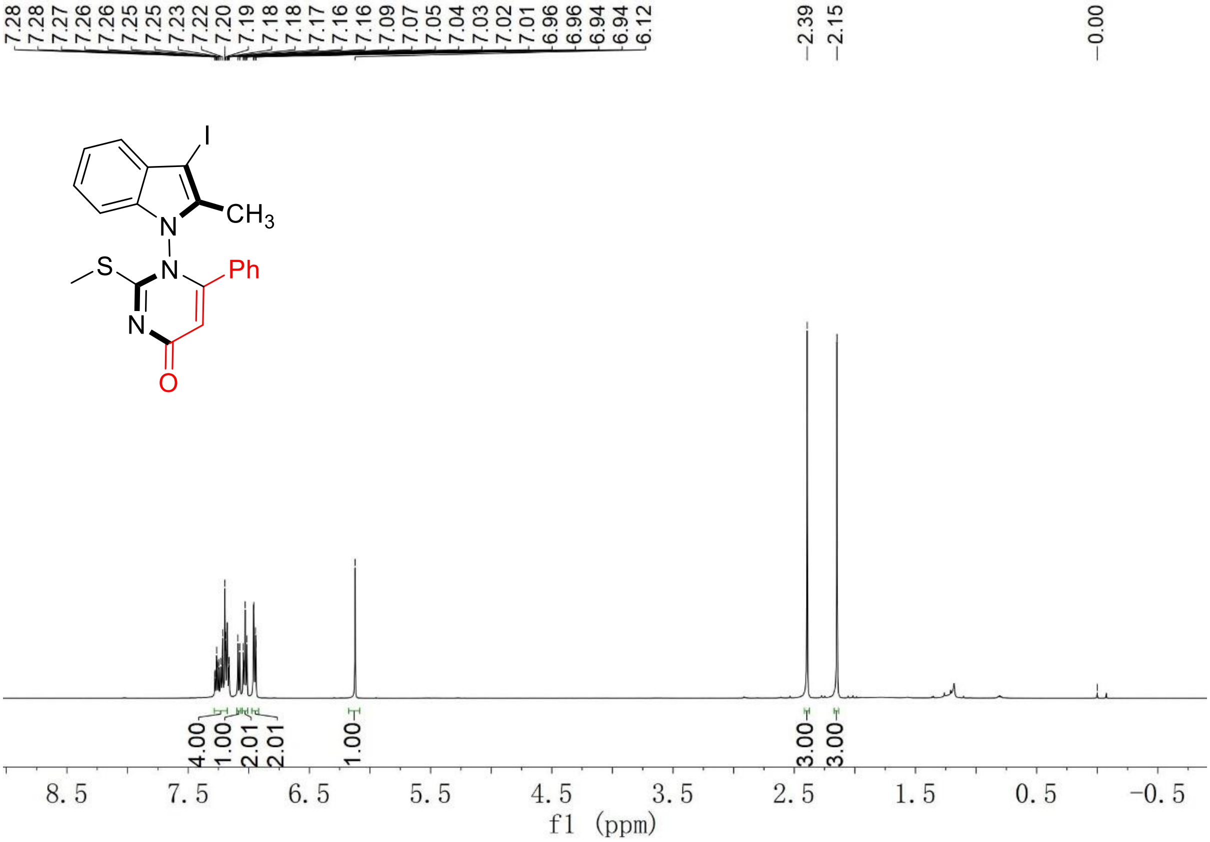

Supplementary Figure 203. **^{1}H NMR spectrum of compound 4 ($CDCl_3$, 500 MHz, 298 K)**

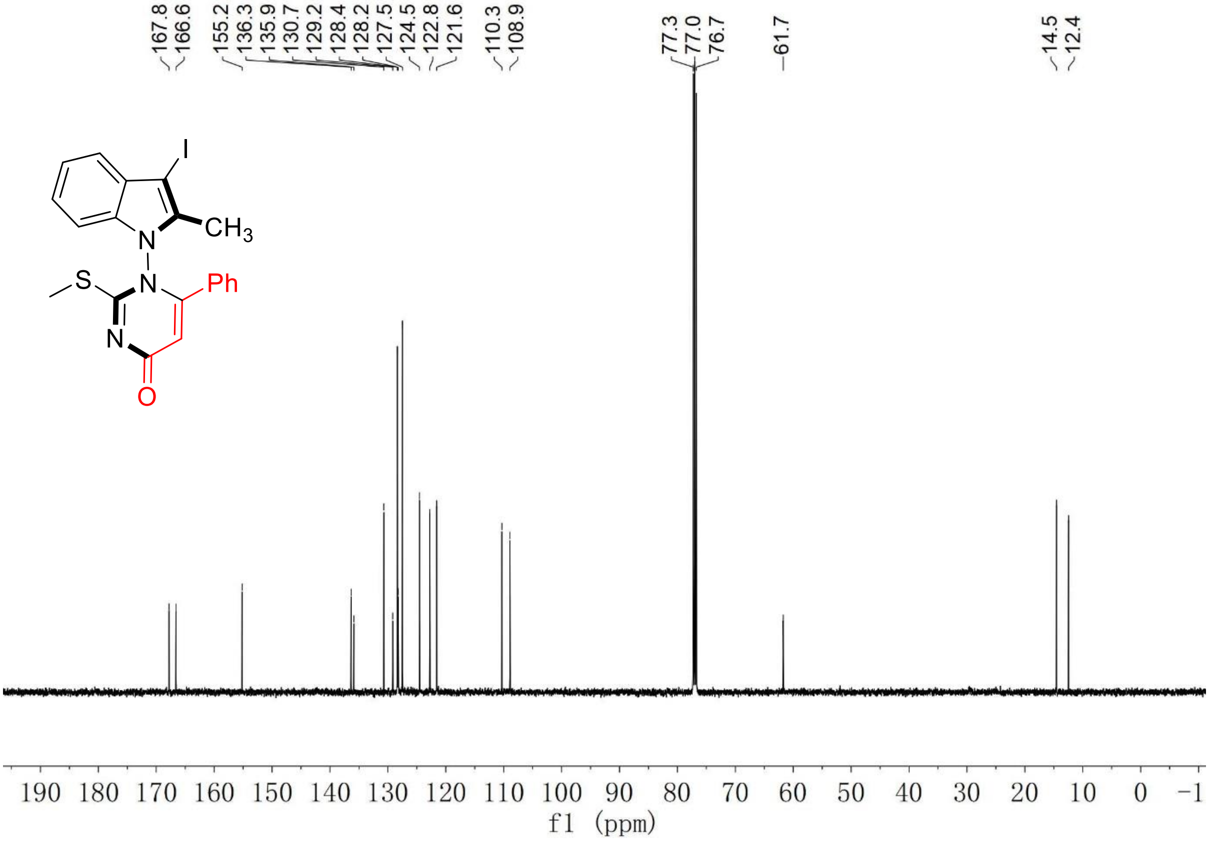

Supplementary Figure 204. **^{13}C NMR spectrum of compound 4 ($CDCl_3$, 126 MHz, 298 K)**

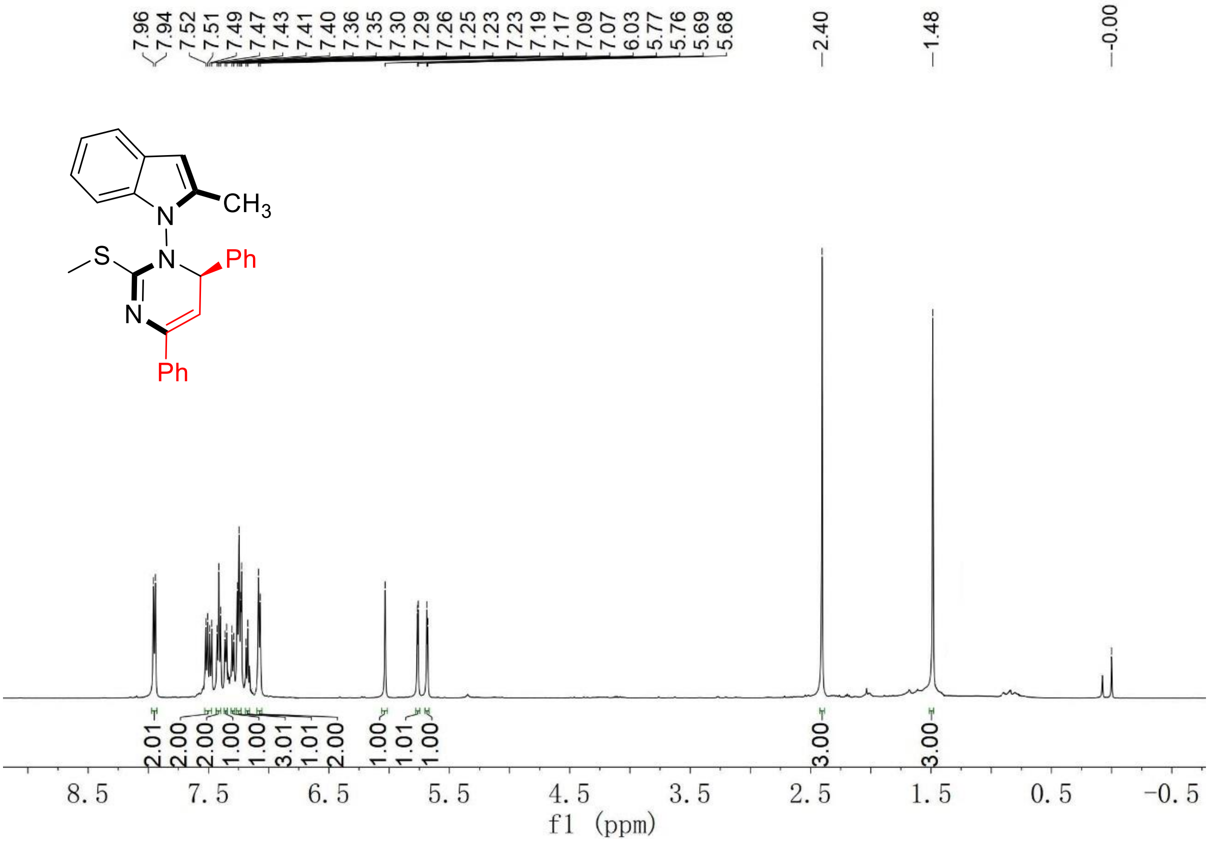

Supplementary Figure 205. ^{1}H NMR spectrum of compound 10 ($CDCl_3$, 500 MHz, 298 K)

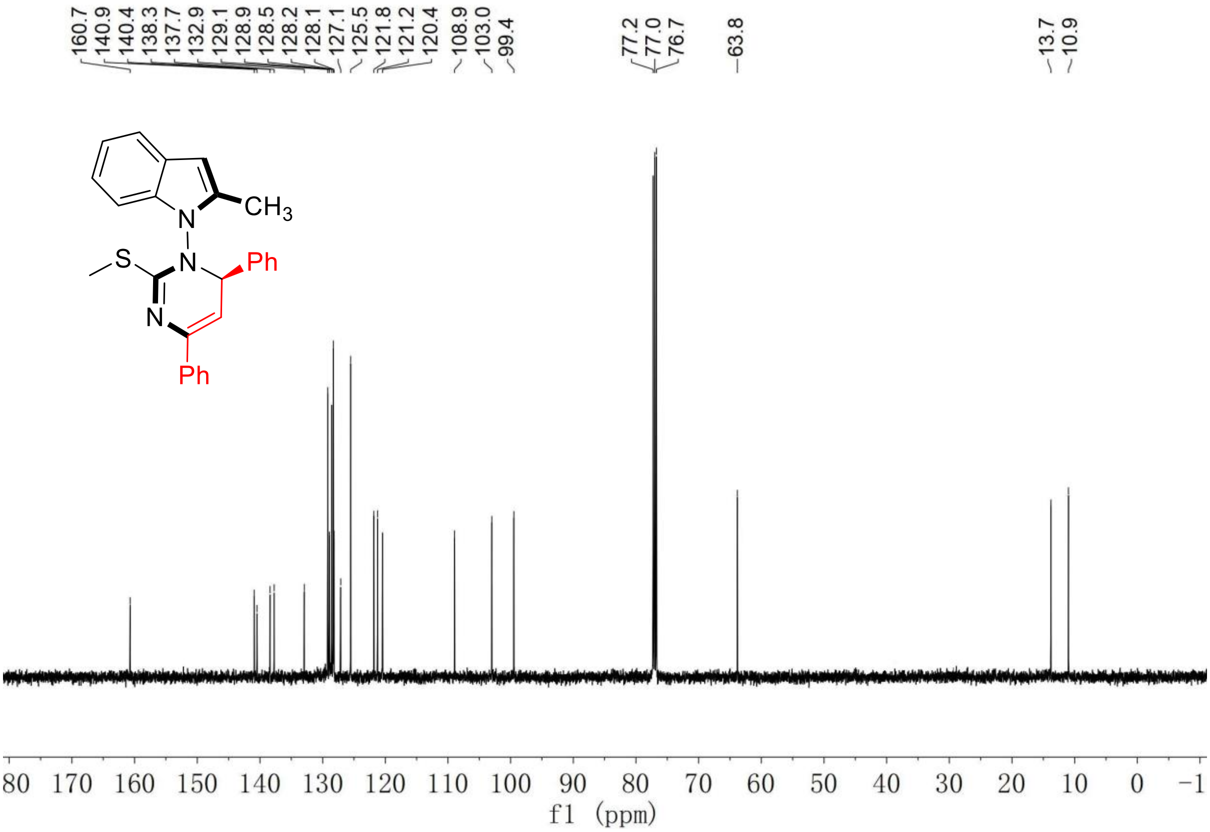

Supplementary Figure 206. ^{13}C NMR spectrum of compound 10 ($CDCl_3$, 126 MHz, 298 K)

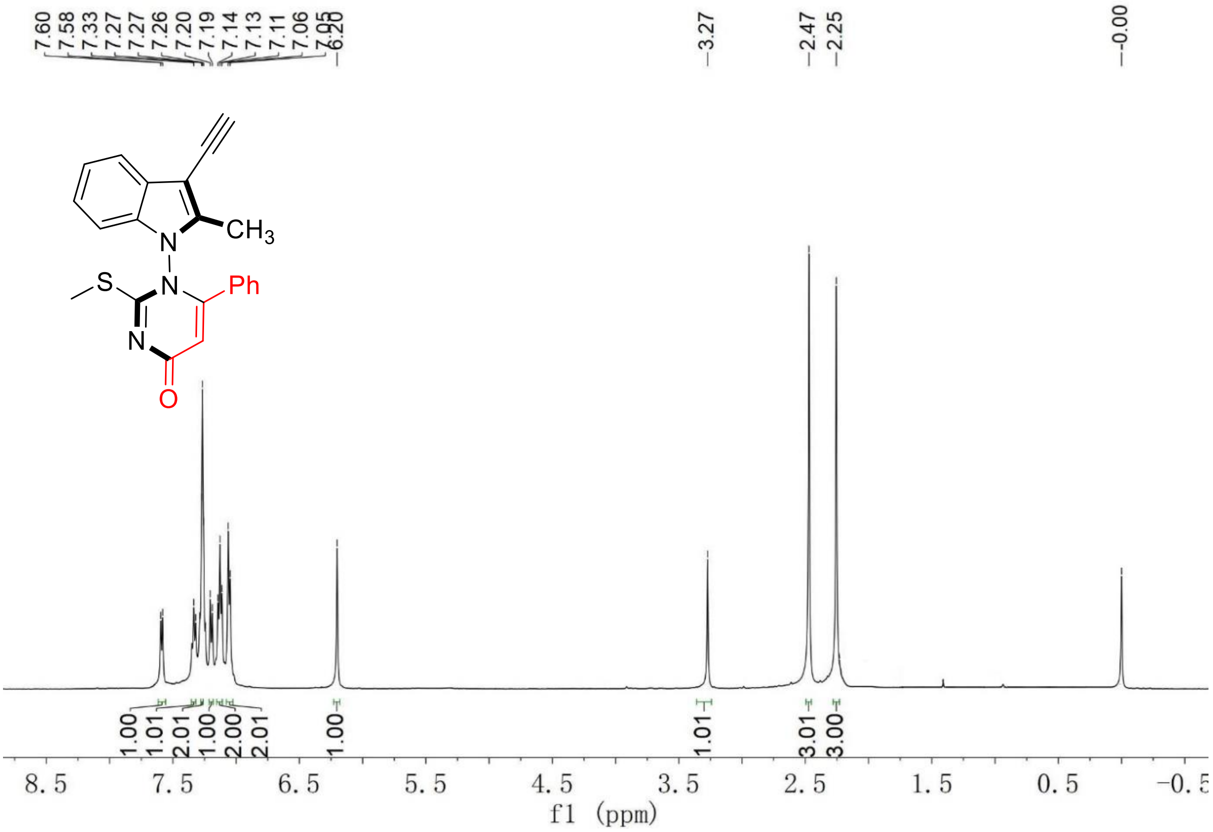

Supplementary Figure 207. ^{1}H **NMR spectrum of compound 11 ($CDCl_3$, 500 MHz, 298 K)**

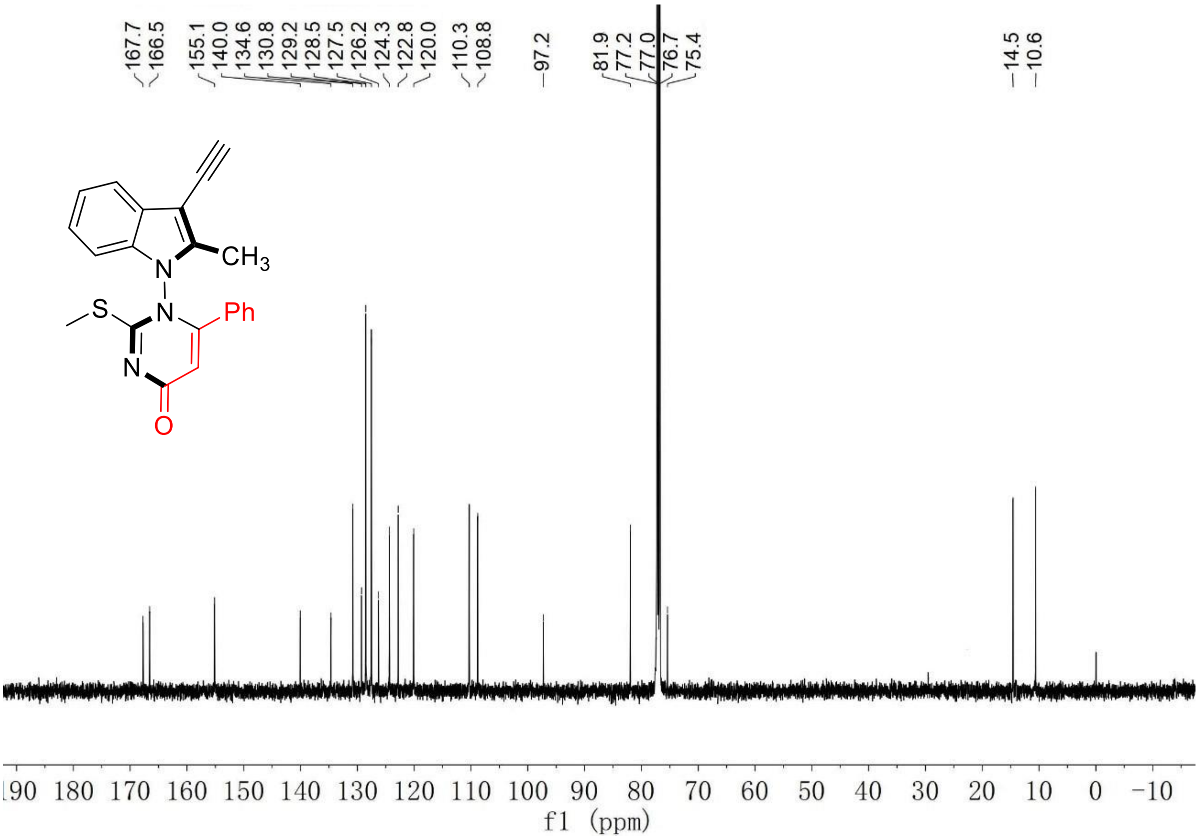

Supplementary Figure 208. ^{13}C **NMR spectrum of compound 11 ($CDCl_3$, 126 MHz, 298 K)**

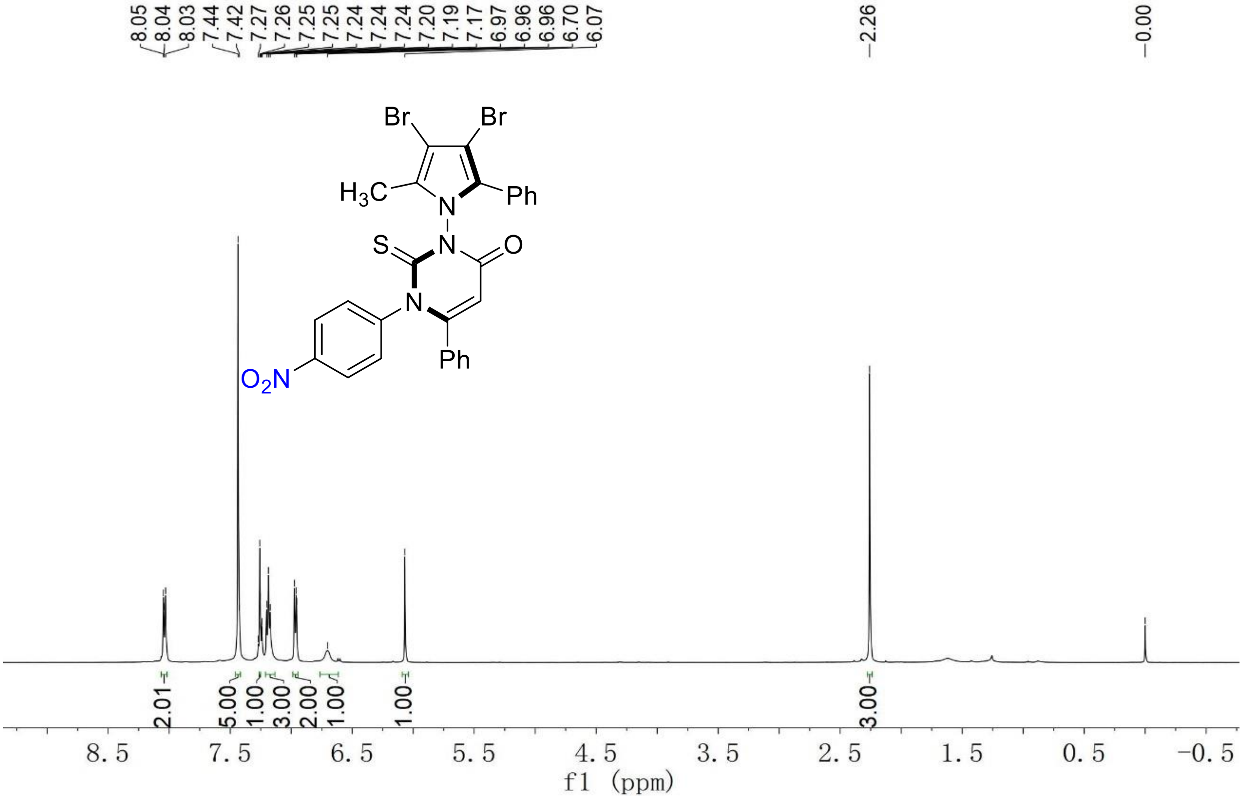

Supplementary Figure 209. ^{1}H NMR spectrum of compound 12 ($CDCl_3$, 500 MHz, 298 K)

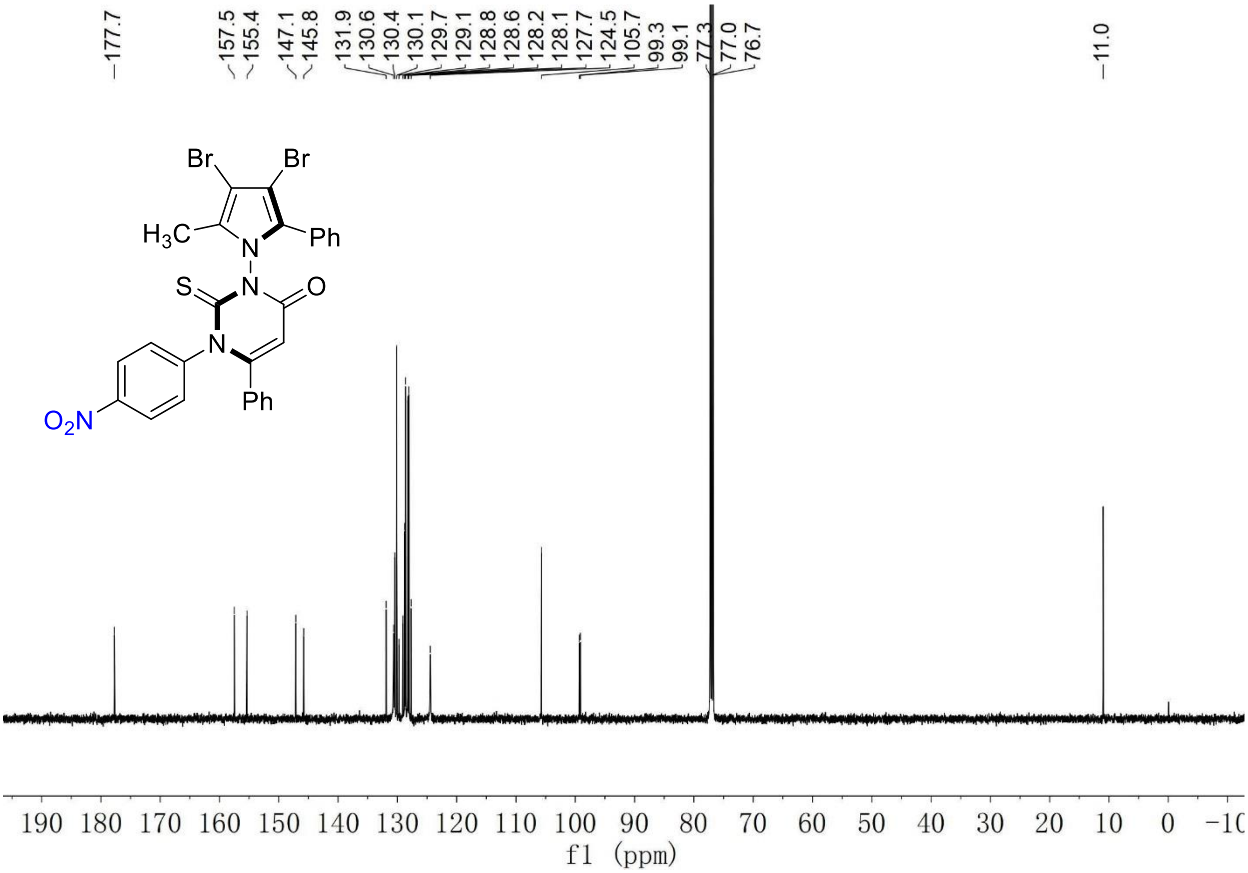

Supplementary Figure 210. ^{13}C NMR spectrum of compound 12 ($CDCl_3$, 126 MHz, 298 K)

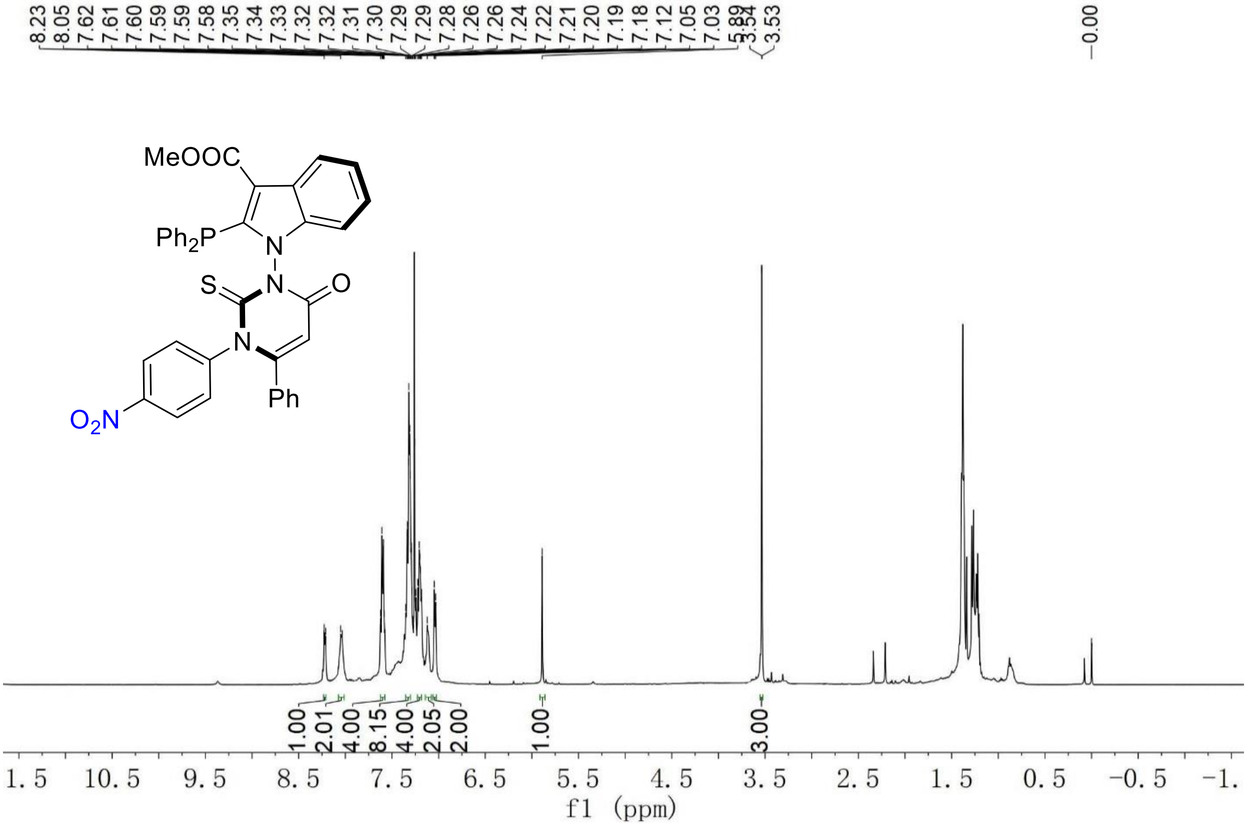

Supplementary Figure 211. ^{1}H NMR spectrum of compound 13 ($CDCl_3$, 500 MHz, 298 K)

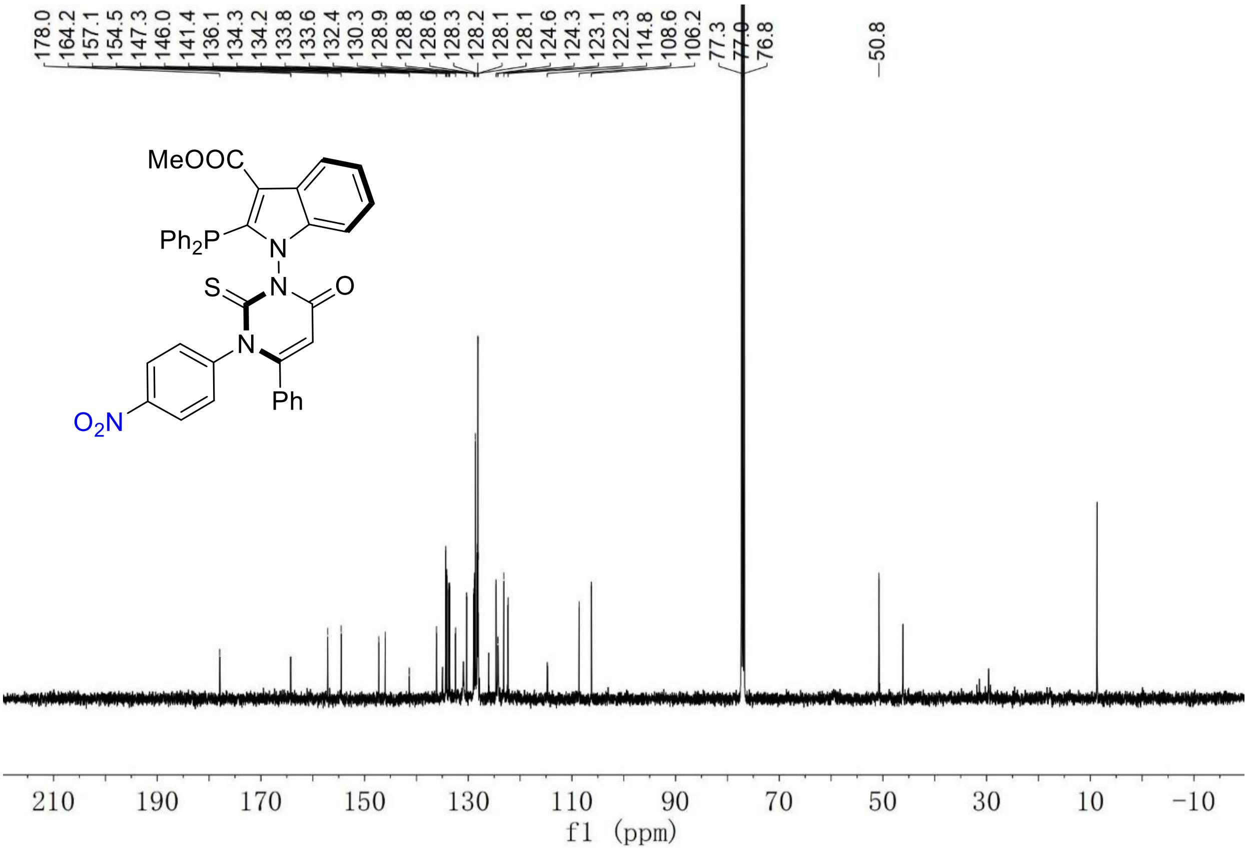

Supplementary Figure 212. ^{13}C NMR spectrum of compound 13 ($CDCl_3$, 126 MHz, 298 K)

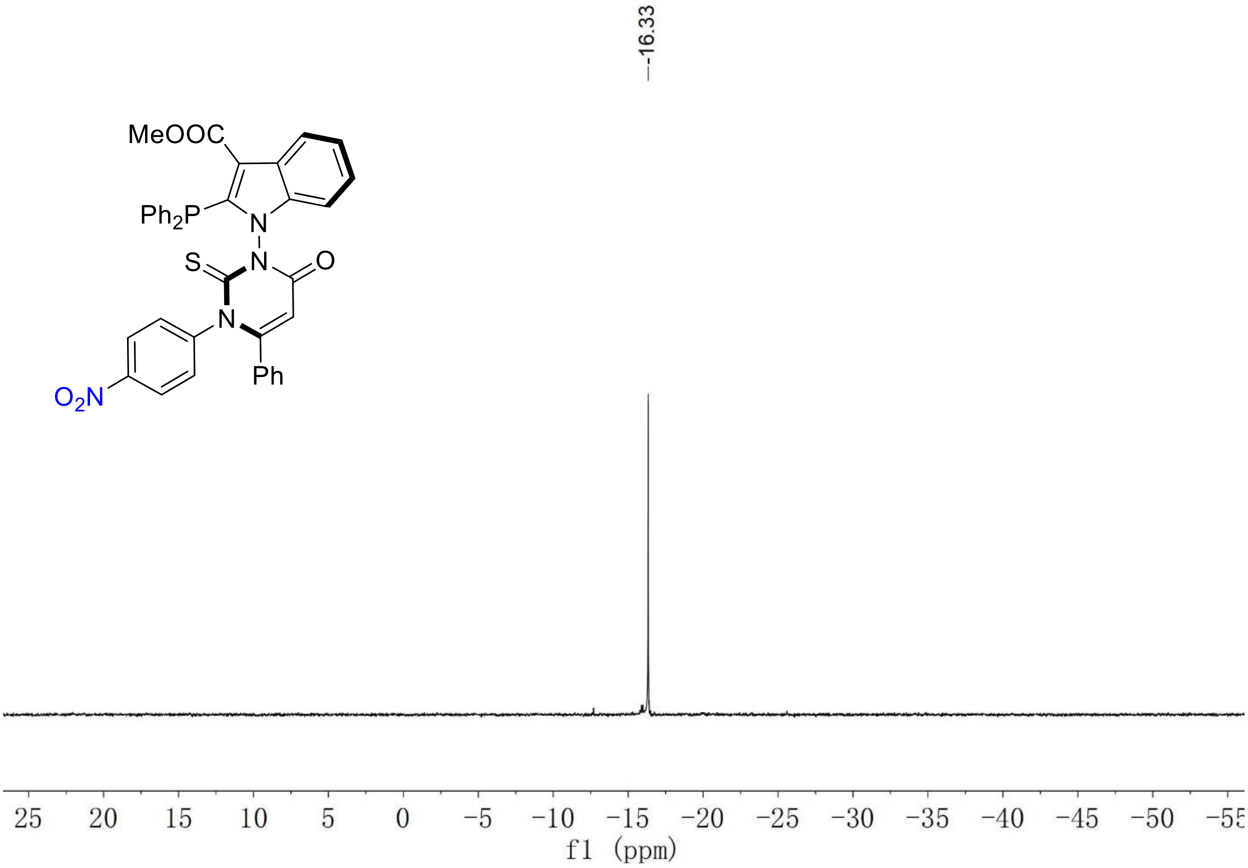

Supplementary Figure 213. ^{31}P NMR spectrum of compound 13 ($CDCl_3$, 202 MHz, 298 K)

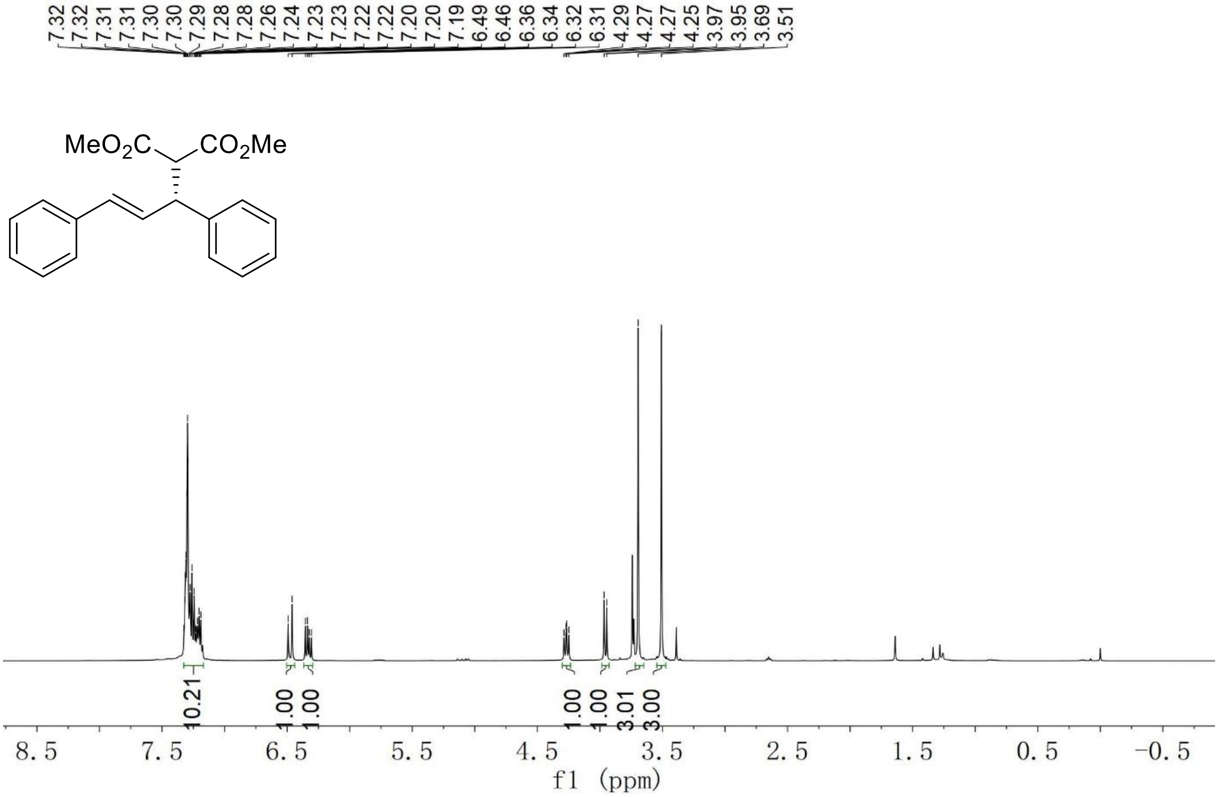

Supplementary Figure 214. ^{1}H NMR spectrum of compound 16 ($CDCl_3$, 500 MHz, 298 K)

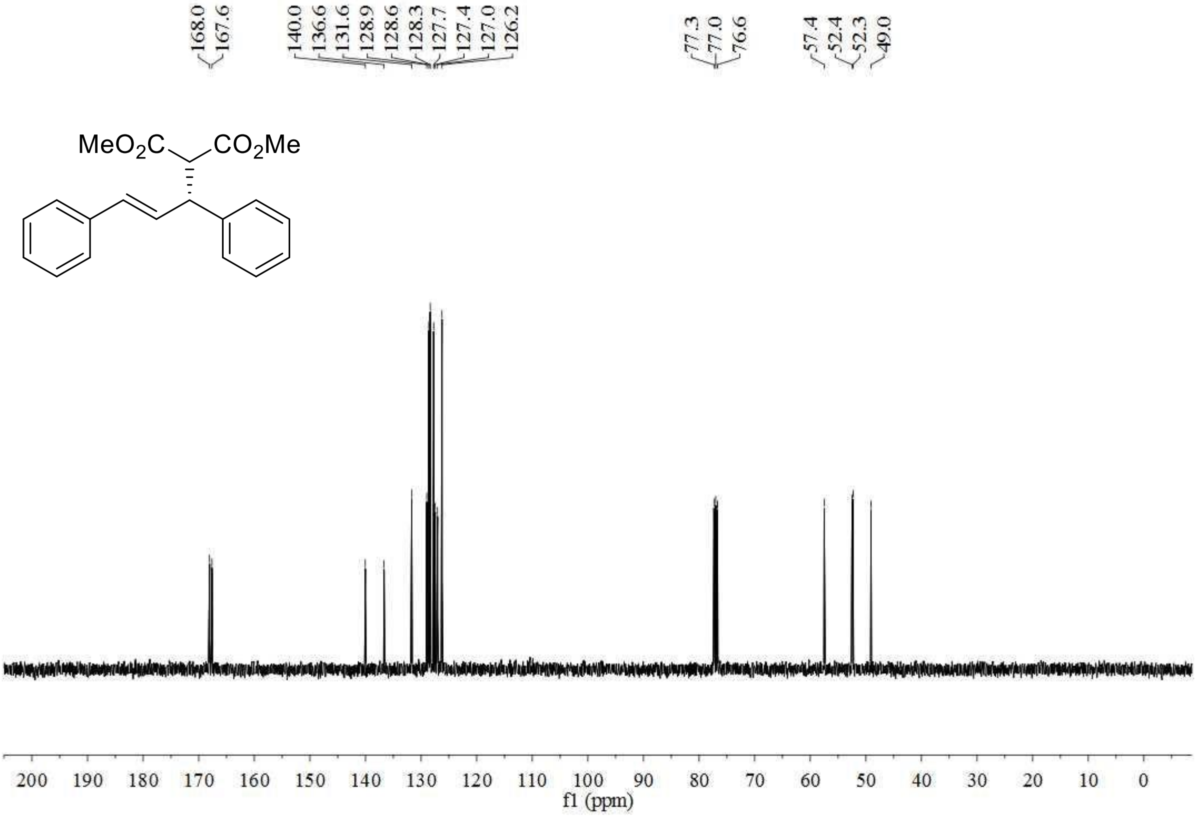

Supplementary Figure 215. ^{13}C NMR spectrum of compound 16 ($CDCl_3$, 126 MHz, 298 K)

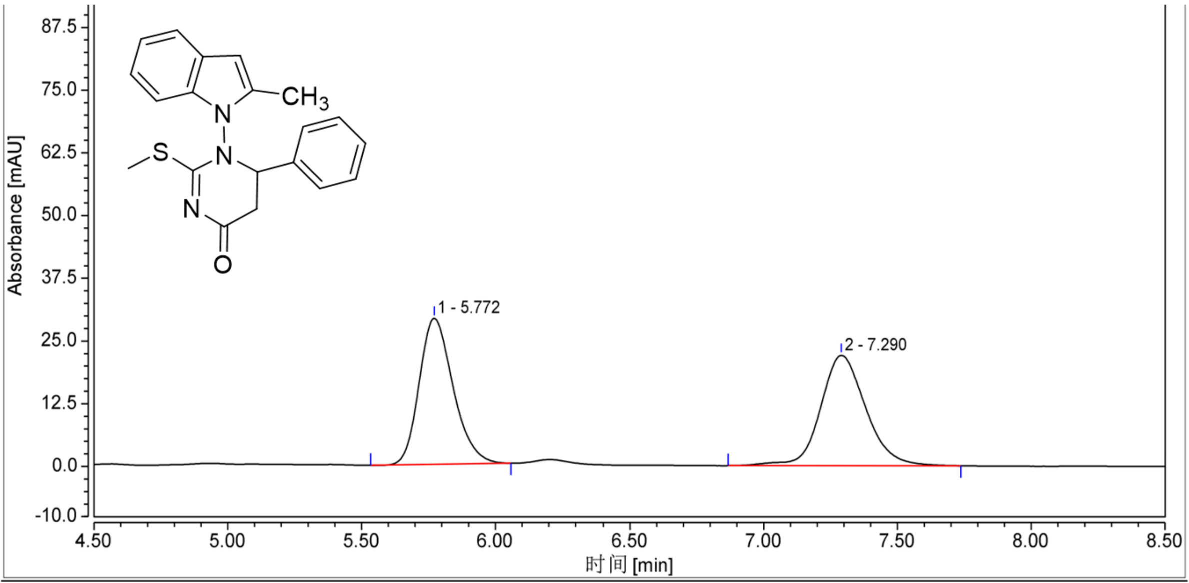

Result

Peak	RT<br>min	Area<br>mAU*min	Height<br>mAU	Area<br>%	Height<br>%
1	5.772	4.286	29.120	49.83	56.97
2	7.290	4.315	21.993	50.17	43.03

Supplementary Figure 216. HPLC chromatogram of racemic 3a

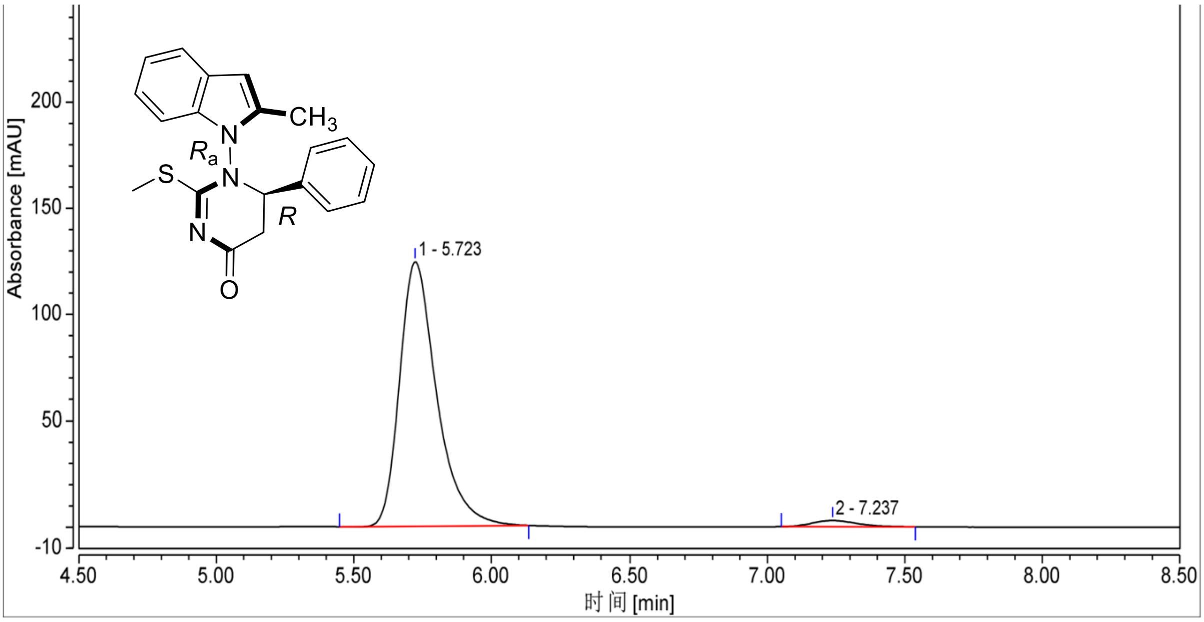

Result

Peak	RT<br>min	Area<br>mAU*min	Height<br>mAU	Area<br>%	Height<br>%
1	5.723	19.475	124.454	97.23	97.69
2	7.237	0.555	2.941	2.77	2.31

Supplementary Figure 217. HPLC chromatogram of enantiomerically enriched 3a

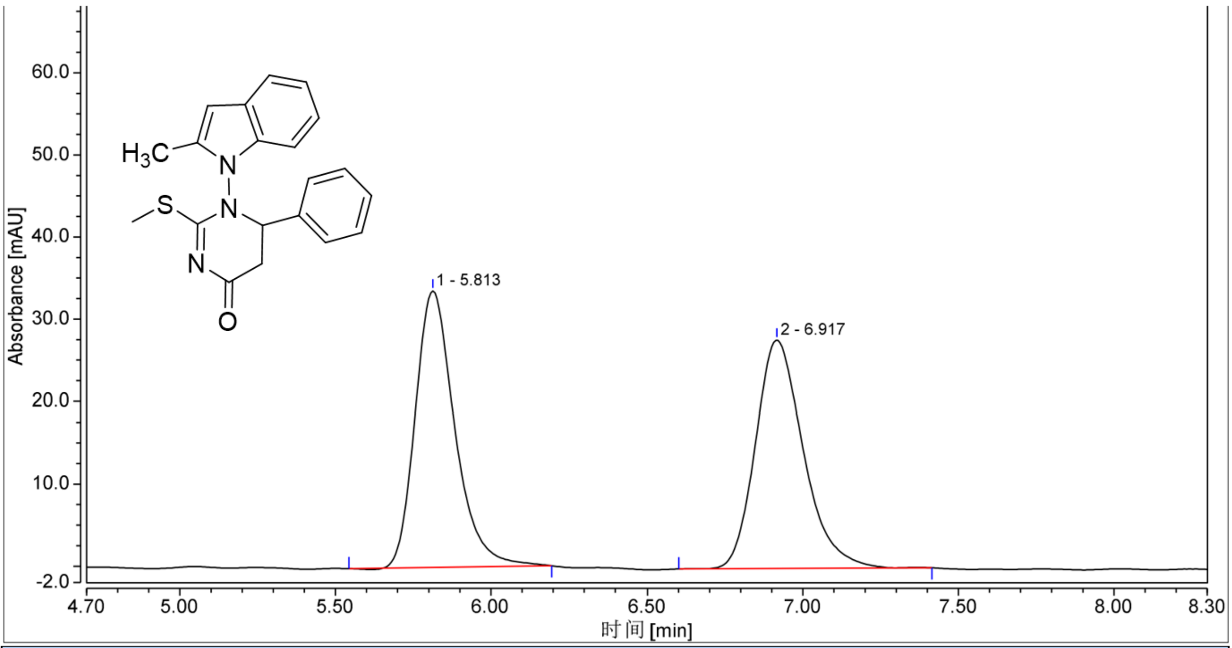

Result

Peak	RT min	Area mAU*min	Height mAU	Area %	Height %
1	5.813	4.941	33.590	49.97	54.78
2	6.917	4.947	27.730	50.03	45.22

Supplementary Figure 218. HPLC chromatogram of racemic 3a'

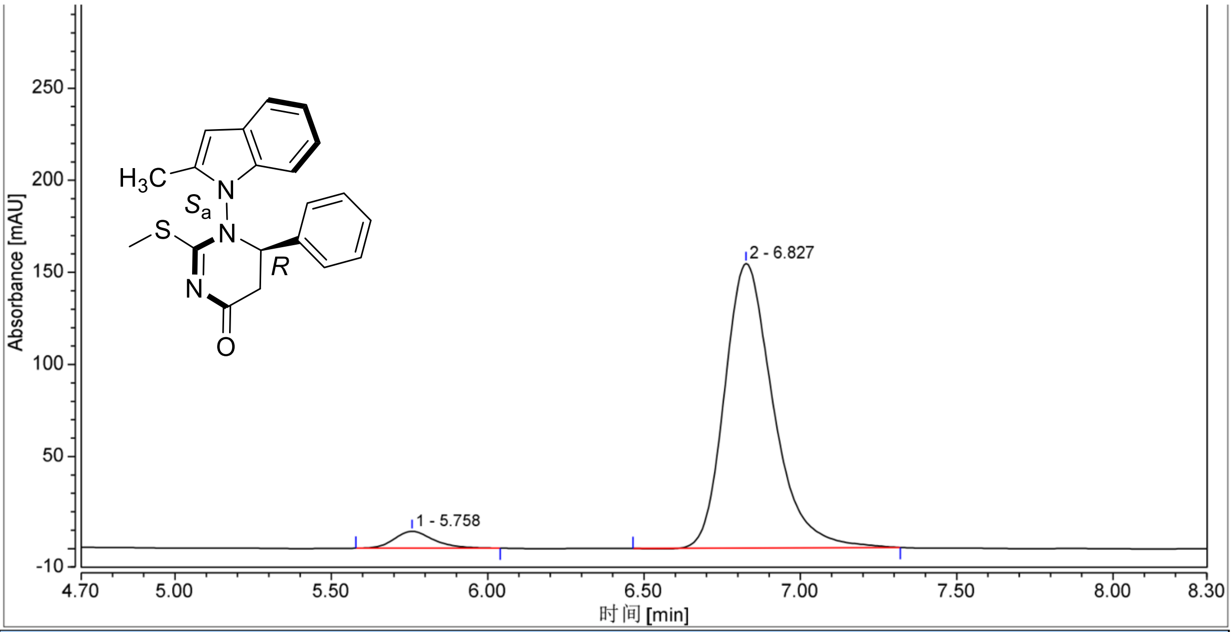

Result

Peak	RT min	Area mAU*min	Height mAU	Area %	Height %
1	5.758	1.336	9.199	4.59	5.62
2	6.827	27.797	154.554	95.41	94.38

Supplementary Figure 219. HPLC chromatogram of enantiomerically enriched 3a'

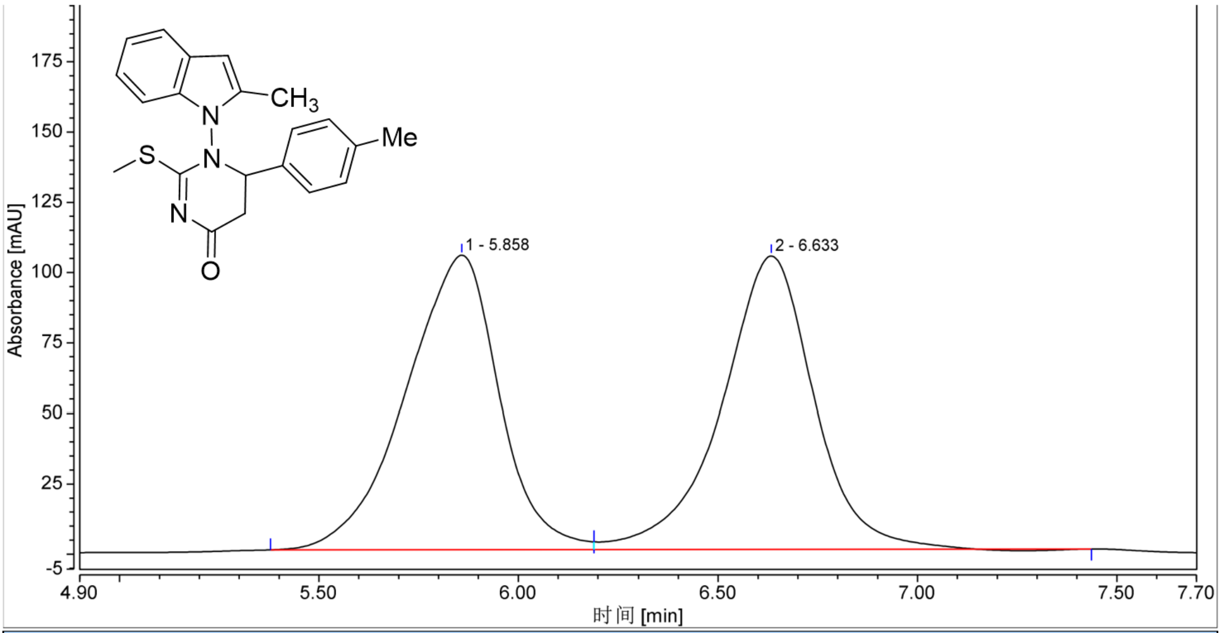

Result

Peak	RT<br>min	Area<br>mAU*min	Height<br>mAU	Area<br>%	Height<br>%
1	5.858	28.445	104.567	50.39	50.10
2	6.633	28.000	104.142	49.61	49.90

Supplementary Figure 220. HPLC chromatogram of racemic 3b

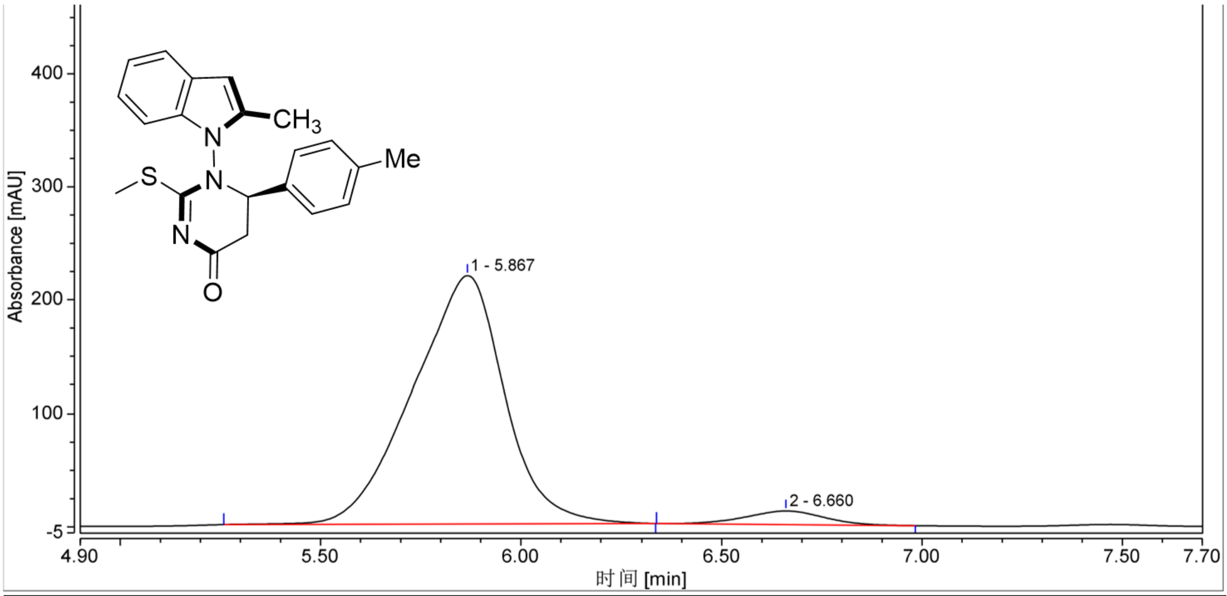

Result

Peak	RT<br>min	Area<br>mAU*min	Height<br>mAU	Area<br>%	Height<br>%
1	5.867	58.168	218.770	95.48	94.77
2	6.660	2.751	12.078	4.52	5.23

Supplementary Figure 221. HPLC chromatogram of enantiomerically enriched 3b

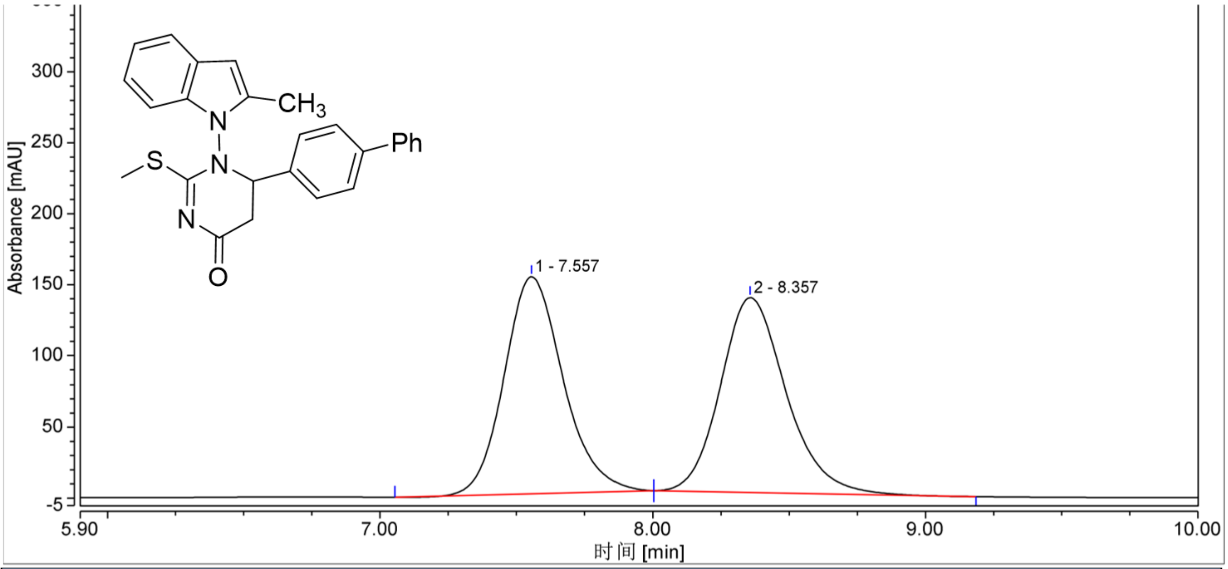

Result

Peak	RT<br>min	Area<br>mAU*min	Height<br>mAU	Area<br>%	Height<br>%
1	7.557	38.265	152.513	50.00	52.72
2	8.357	38.264	136.787	50.00	47.28

Supplementary Figure 222. HPLC chromatogram of racemic 3c

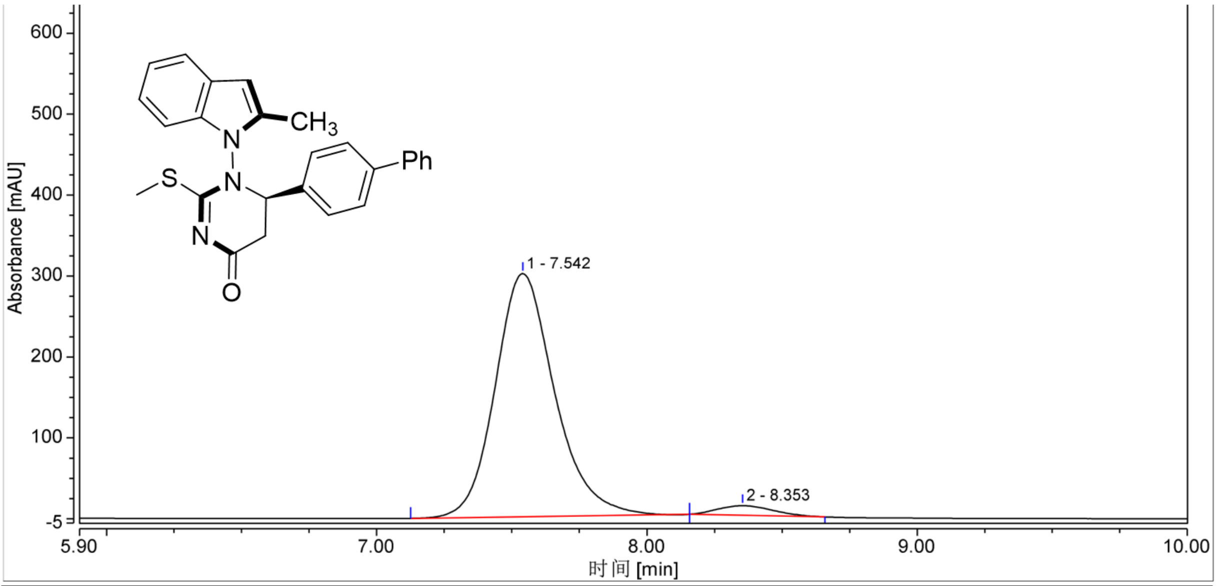

Result

Peak	RT<br>min	Area<br>mAU*min	Height<br>mAU	Area<br>%	Height<br>%
1	7.542	76.598	300.448	96.44	96.21
2	8.353	2.832	11.835	3.56	3.79

Supplementary Figure 223. HPLC chromatogram of enantiomerically enriched 3c

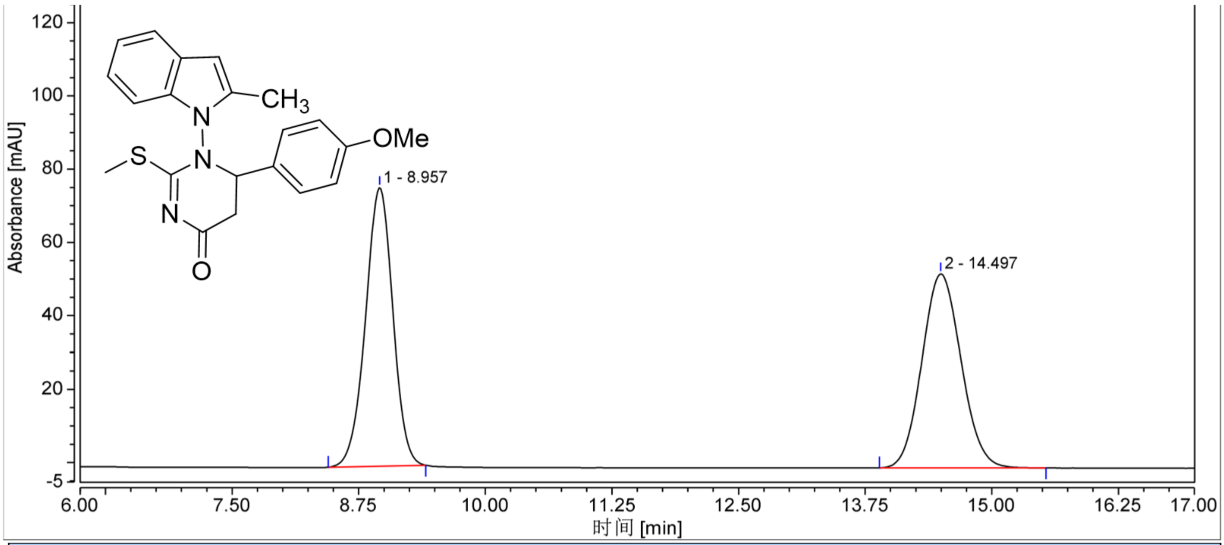

Result

Peak	RT<br>min	Area<br>mAU*min	Height<br>mAU	Area<br>%	Height<br>%
1	8.957	23.629	75.948	49.97	58.97
2	14.497	23.656	52.845	50.03	41.03

Supplementary Figure 224. HPLC chromatogram of racemic 3d

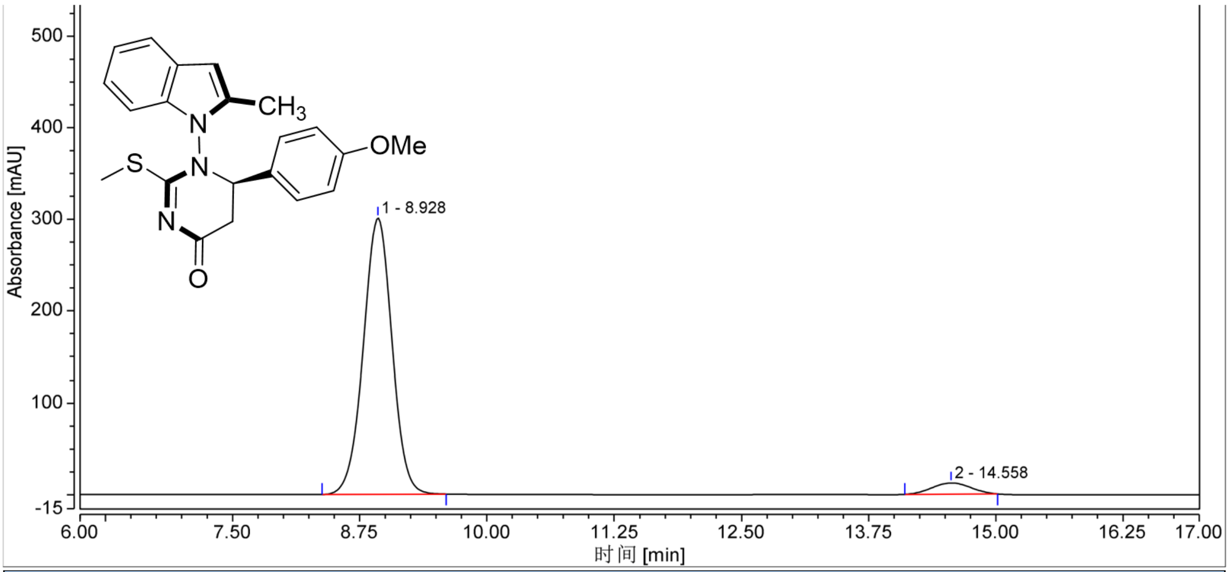

Result

Peak	RT<br>min	Area<br>mAU*min	Height<br>mAU	Area<br>%	Height<br>%
1	8.928	97.806	300.564	94.78	96.09
2	14.558	5.383	12.239	5.22	3.91

Supplementary Figure 225. HPLC chromatogram of enantiomerically enriched 3d

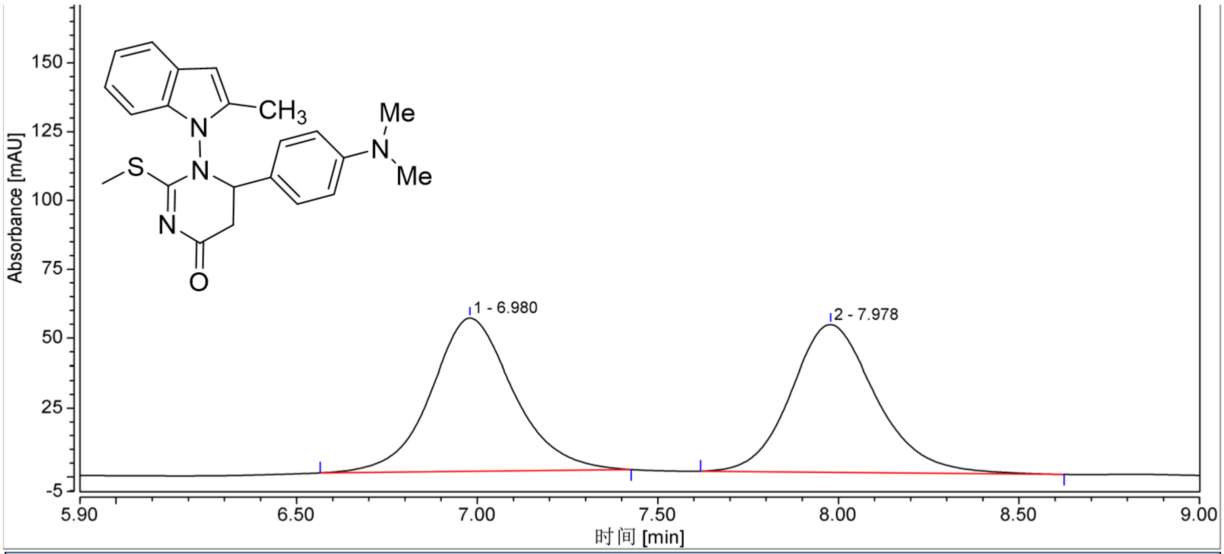

Result

Peak	RT<br>min	Area<br>mAU*min	Height<br>mAU	Area<br>%	Height<br>%
1	6.980	14.686	55.188	50.18	50.92
2	7.978	14.579	53.203	49.82	49.08

Supplementary Figure 226. HPLC chromatogram of racemic 3e

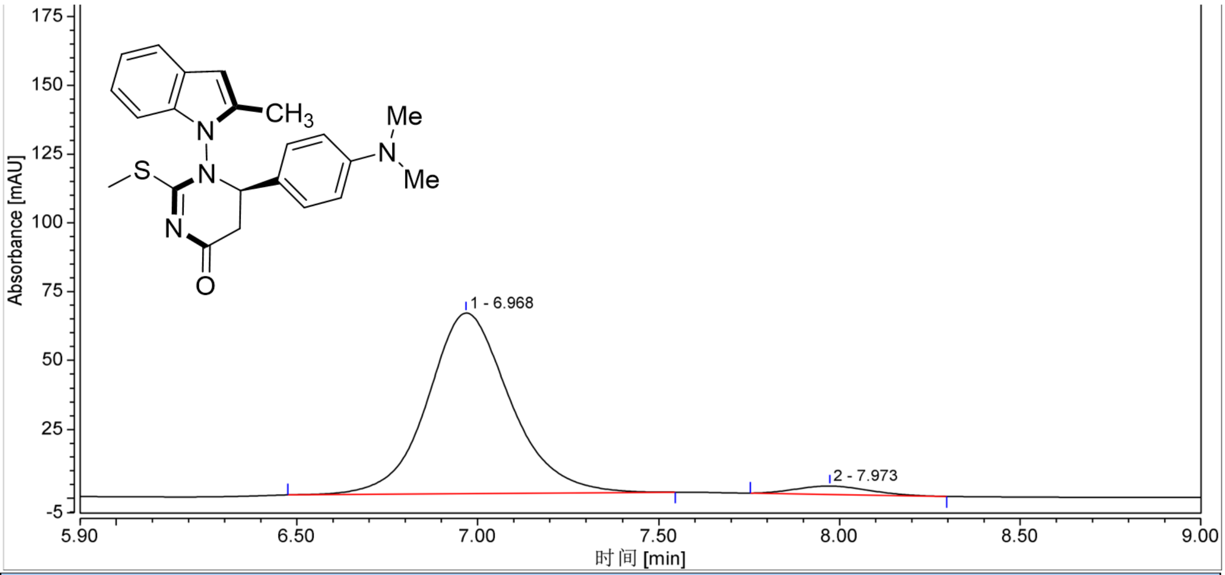

Result

Peak	RT<br>min	Area<br>mAU*min	Height<br>mAU	Area<br>%	Height<br>%
1	6.968	17.390	65.491	96.09	95.55
2	7.973	0.708	3.048	3.91	4.45

Supplementary Figure 227. HPLC chromatogram of enantiomerically enriched 3e

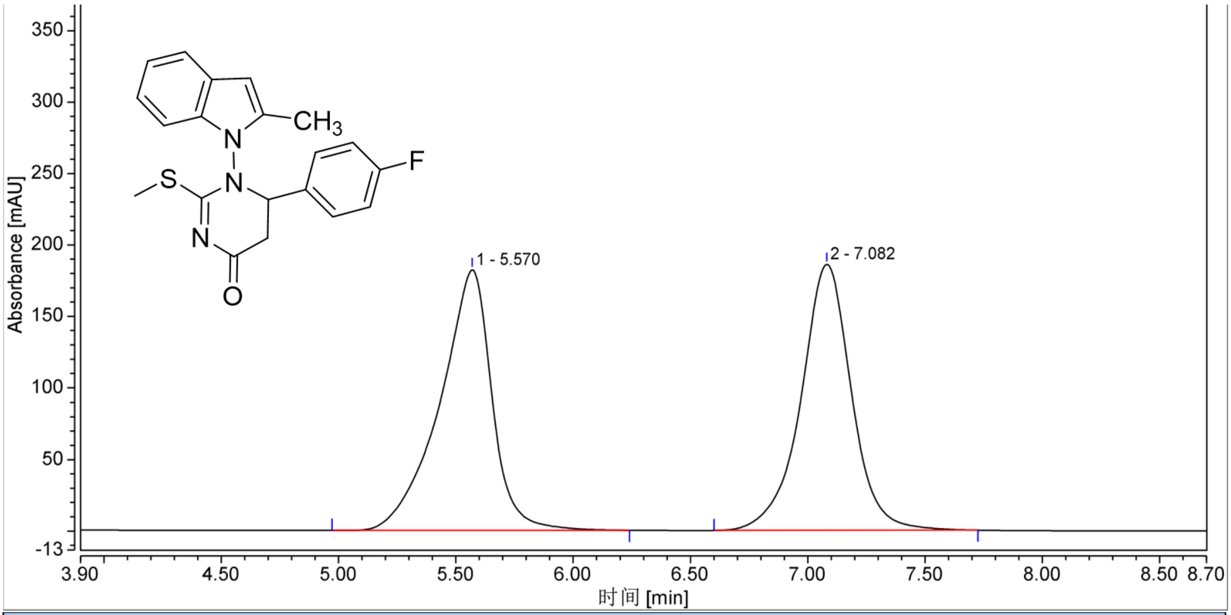

Result

Peak	RT<br>min	Area<br>mAU*min	Height<br>mAU	Area<br>%	Height<br>%
1	5.570	46.882	182.104	50.26	49.49
2	7.082	46.392	185.863	49.74	50.51

Supplementary Figure 228. HPLC chromatogram of racemic 3f

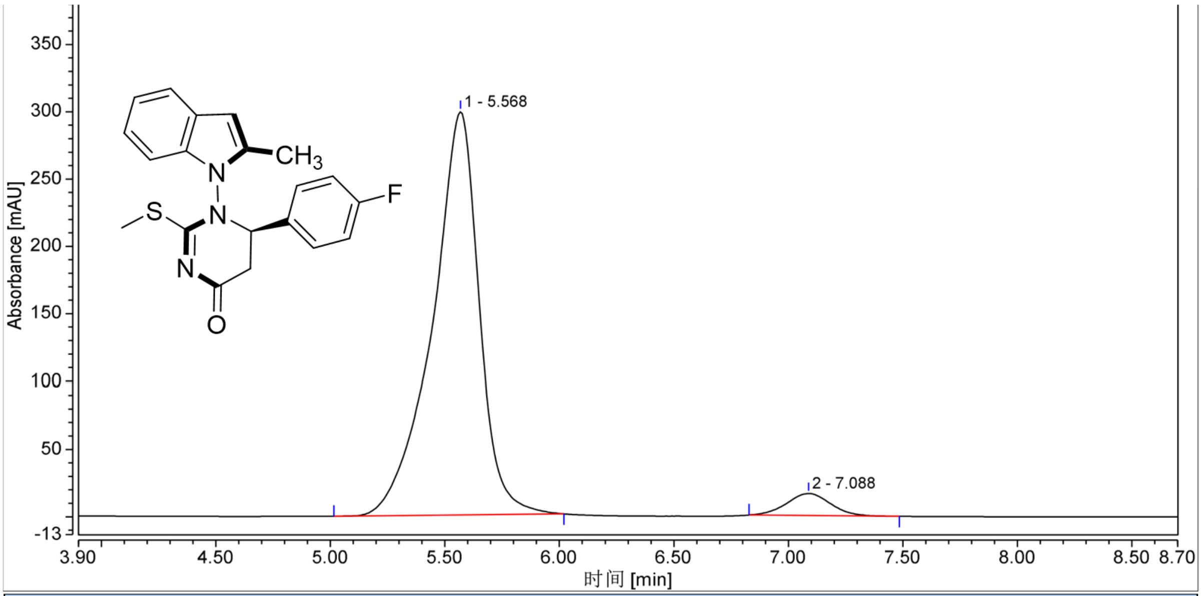

Result

Peak	RT<br>min	Area<br>mAU*min	Height<br>mAU	Area<br>%	Height<br>%
1	5.568	70.626	298.777	95.09	94.81
2	7.088	3.646	16.365	4.91	5.19

Supplementary Figure 229. HPLC chromatogram of enantiomerically enriched 3f

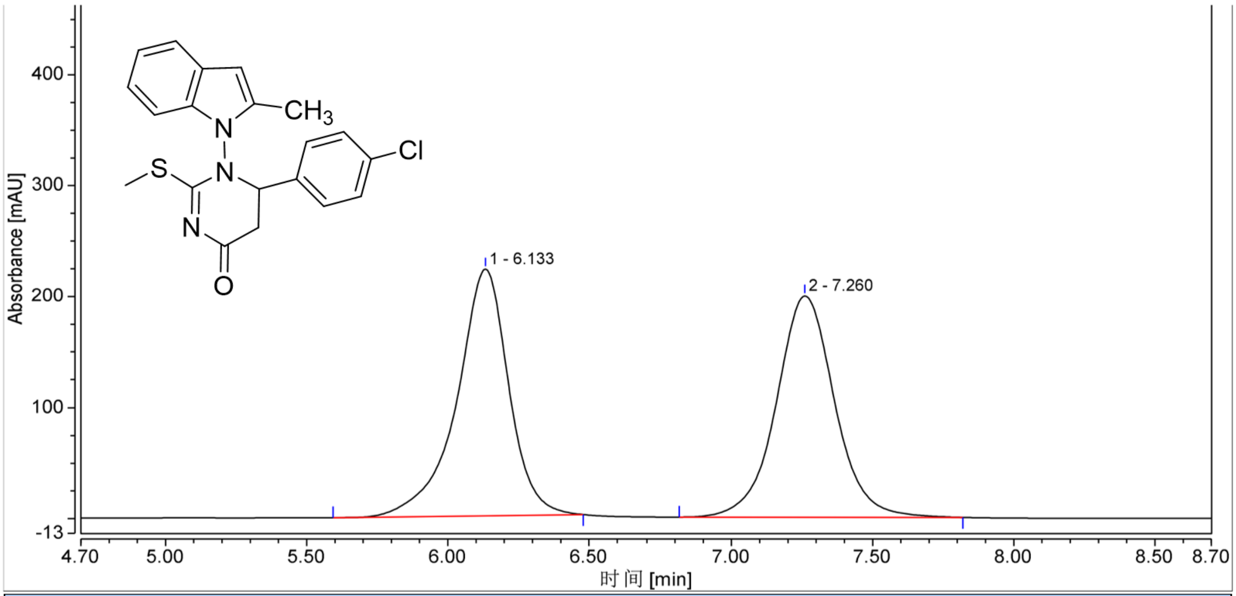

Result

Peak	RT<br>min	Area<br>mAU*min	Height<br>mAU	Area<br>%	Height<br>%
1	6.133	47.427	222.140	49.85	52.72
2	7.260	47.713	199.237	50.15	47.28

Supplementary Figure 230. HPLC chromatogram of racemic 3g

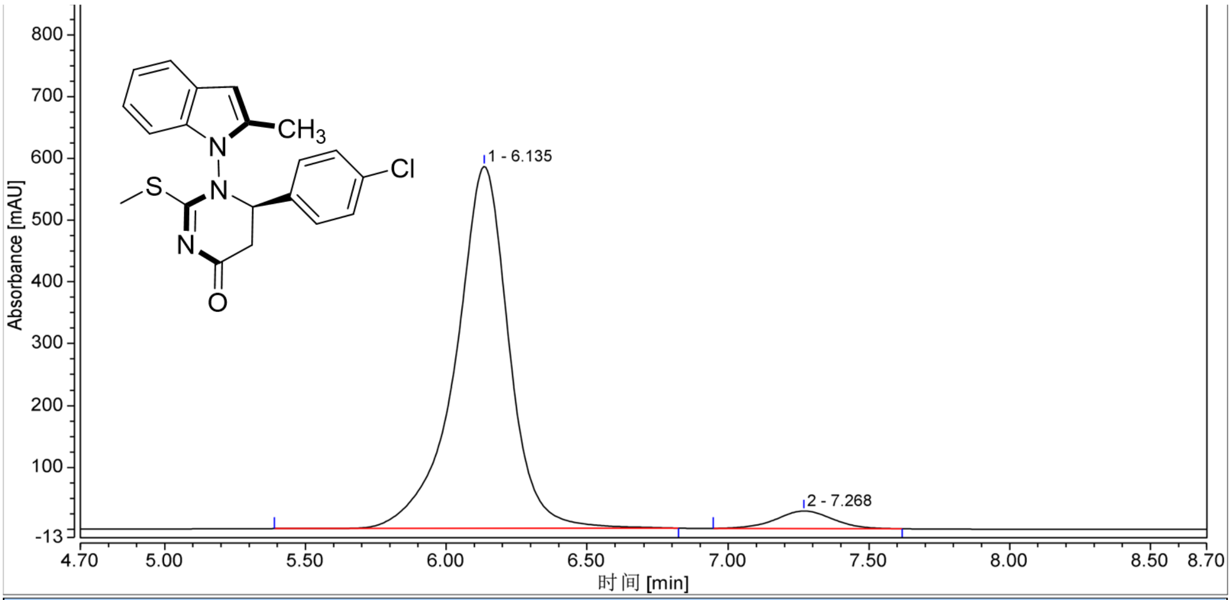

Result

Peak	RT<br>min	Area<br>mAU*min	Height<br>mAU	Area<br>%	Height<br>%
1	6.135	129.196	585.377	95.10	95.34
2	7.268	6.658	28.604	4.90	4.66

Supplementary Figure 231. HPLC chromatogram of enantiomerically enriched 3g

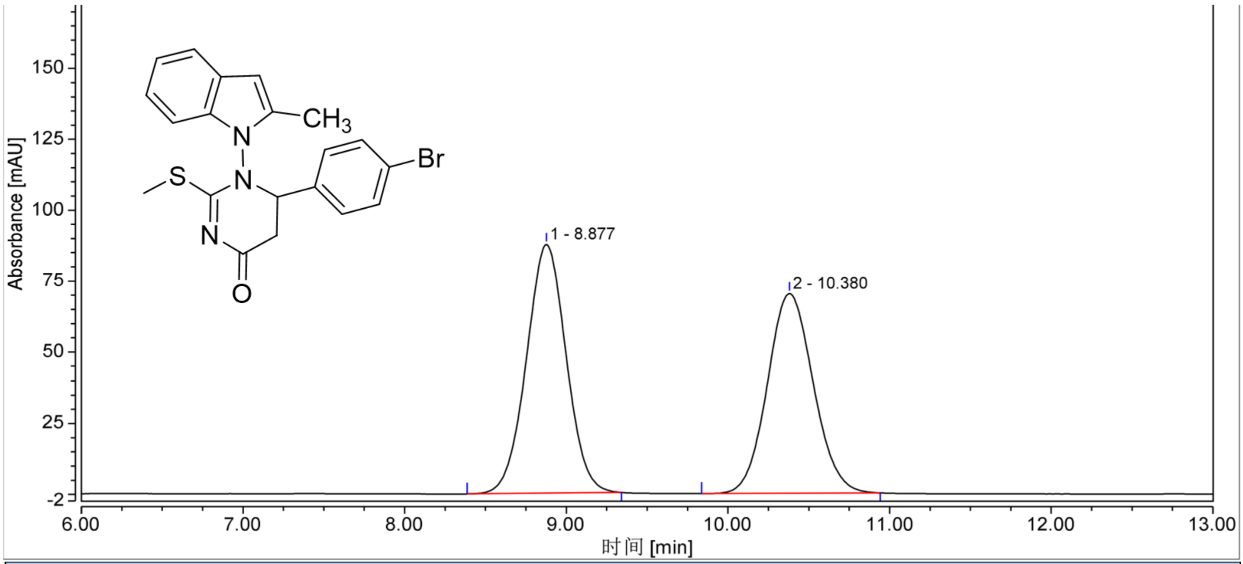

Result					
Peak	RT<br>min	Area<br>mAU*min	Height<br>mAU	Area<br>%	Height<br>%
1	8.877	24.968	87.520	51.97	55.50
2	10.380	23.076	70.186	48.03	44.50

Supplementary Figure 232. HPLC chromatogram of racemic 3h

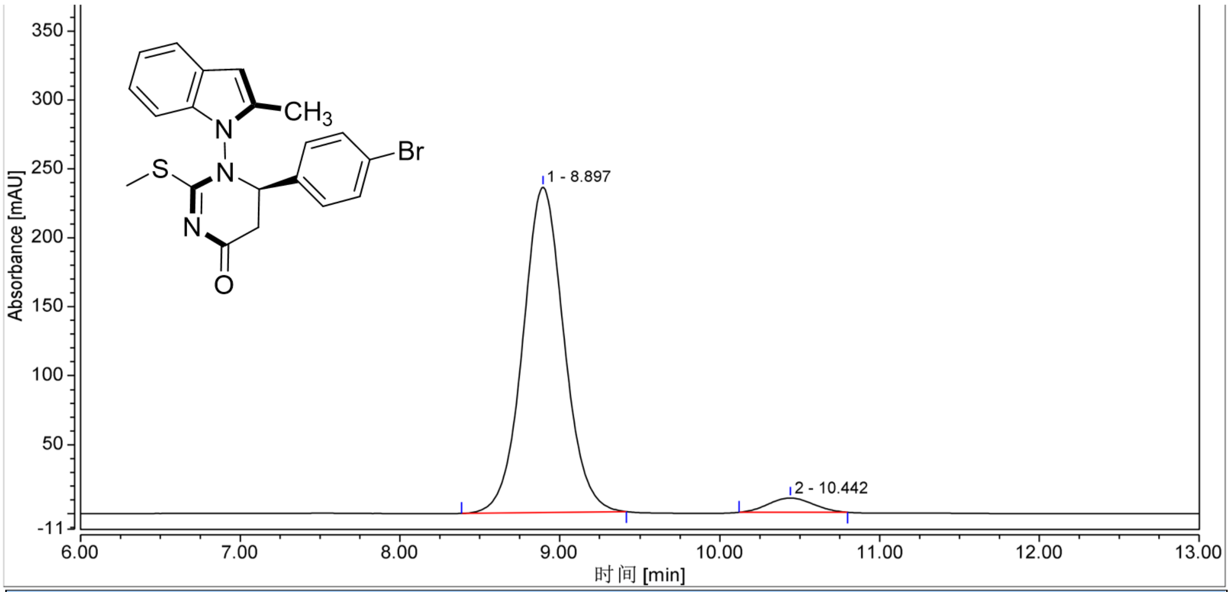

Result					
Peak	RT<br>min	Area<br>mAU*min	Height<br>mAU	Area<br>%	Height<br>%
1	8.897	70.505	235.952	95.45	95.78
2	10.442	3.362	10.400	4.55	4.22

Supplementary Figure 233. HPLC chromatogram of enantiomerically enriched 3h

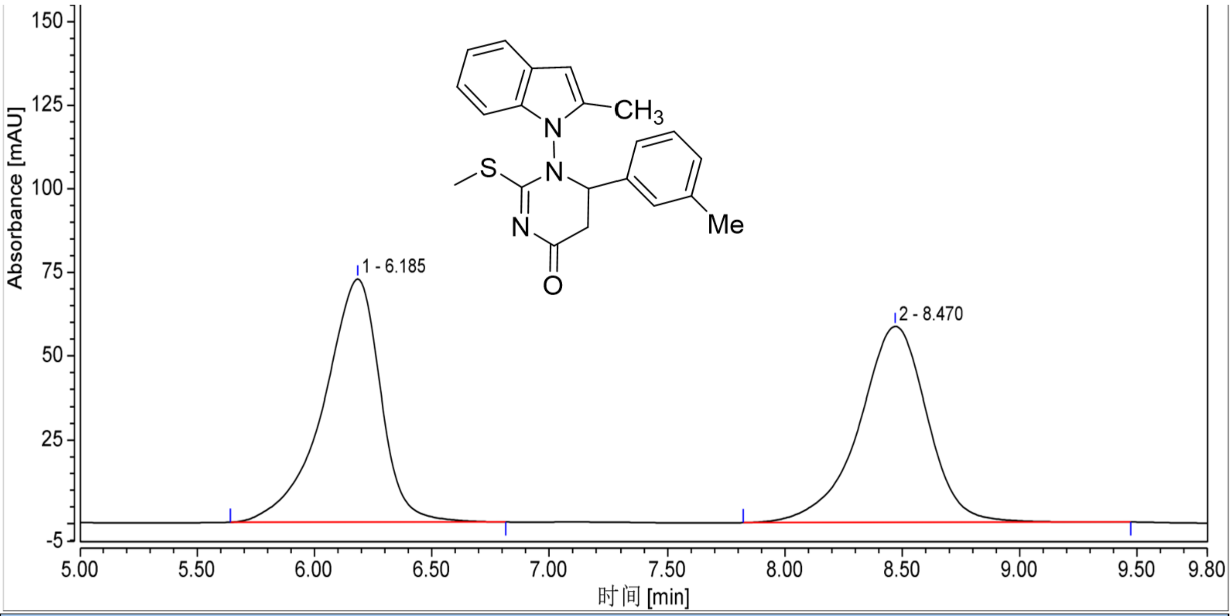

Result

Peak	RT min	Area mAU*min	Height mAU	Area %	Height %
1	6.185	20.441	72.573	50.89	55.38
2	8.470	19.726	58.470	49.11	44.62

Supplementary Figure 234. HPLC chromatogram of racemic 3i

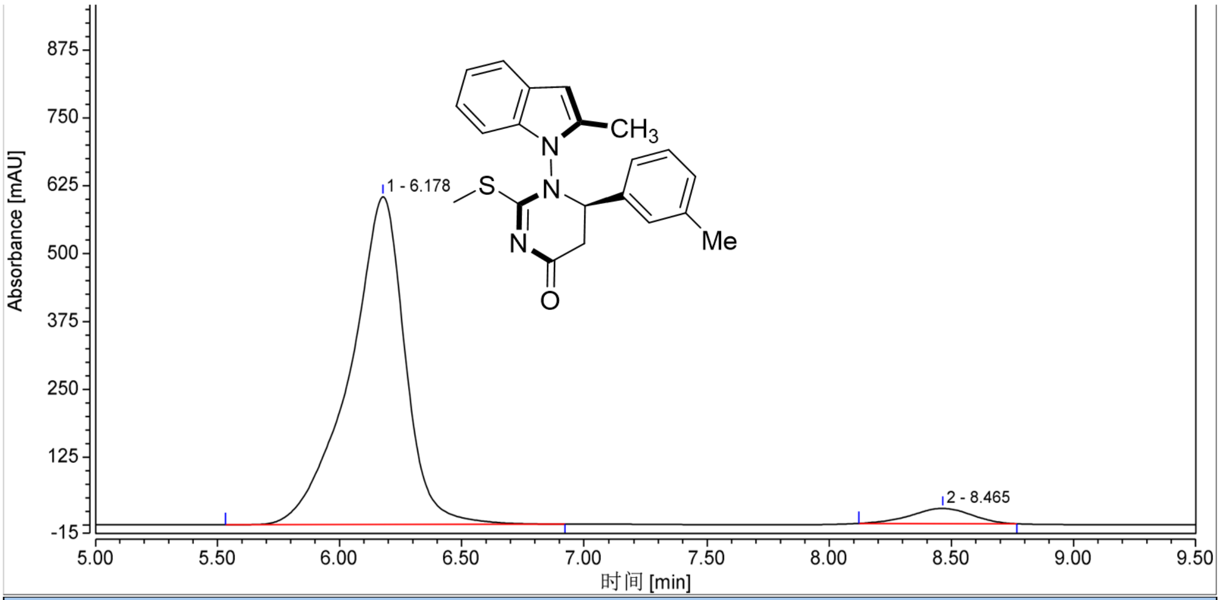

Result

Peak	RT min	Area mAU*min	Height mAU	Area %	Height %
1	6.178	157.987	604.317	95.18	95.58
2	8.465	8.000	27.928	4.82	4.42

Supplementary Figure 235. HPLC chromatogram of enantiomerically enriched 3i

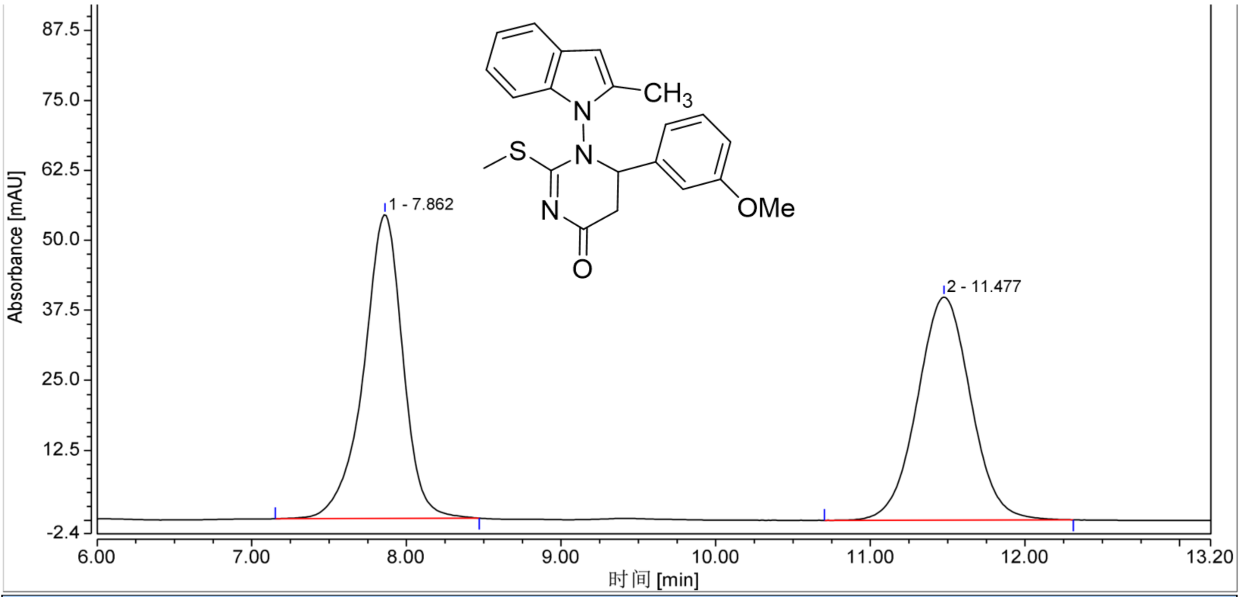

Result

Peak	RT<br>min	Area<br>mAU*min	Height<br>mAU	Area<br>%	Height<br>%
1	7.862	15.960	54.205	50.08	57.68
2	11.477	15.910	39.770	49.92	42.32

Supplementary Figure 236. HPLC chromatogram of racemic 3j

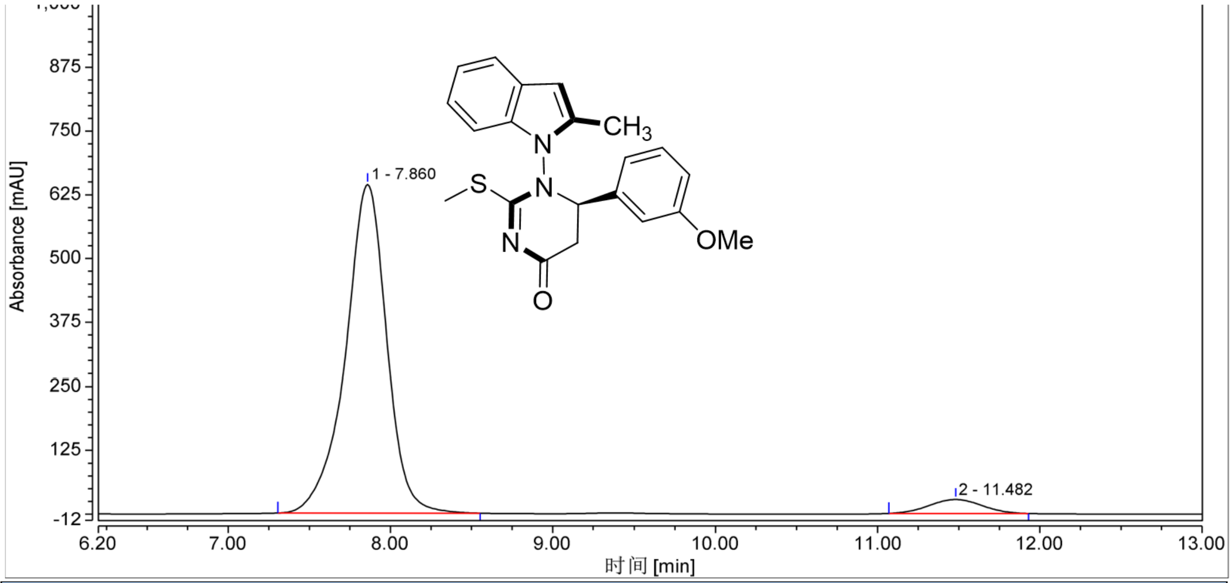

Result

Peak	RT<br>min	Area<br>mAU*min	Height<br>mAU	Area<br>%	Height<br>%
1	7.860	189.044	643.399	94.81	95.92
2	11.482	10.340	27.385	5.19	4.08

Supplementary Figure 237. HPLC chromatogram of enantiomerically enriched 3j

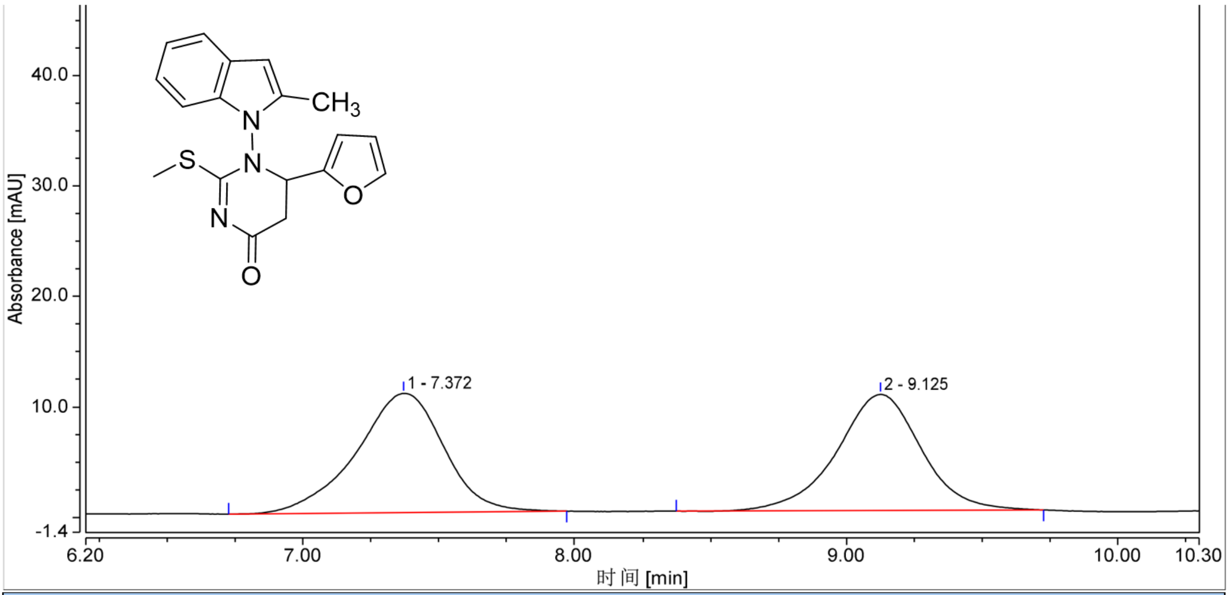

Result

Peak	RT min	Area mAU*min	Height mAU	Area %	Height %
1	7.372	4.046	10.810	51.60	50.68
2	9.125	3.794	10.520	48.40	49.32

Supplementary Figure 238. HPLC chromatogram of racemic 3k

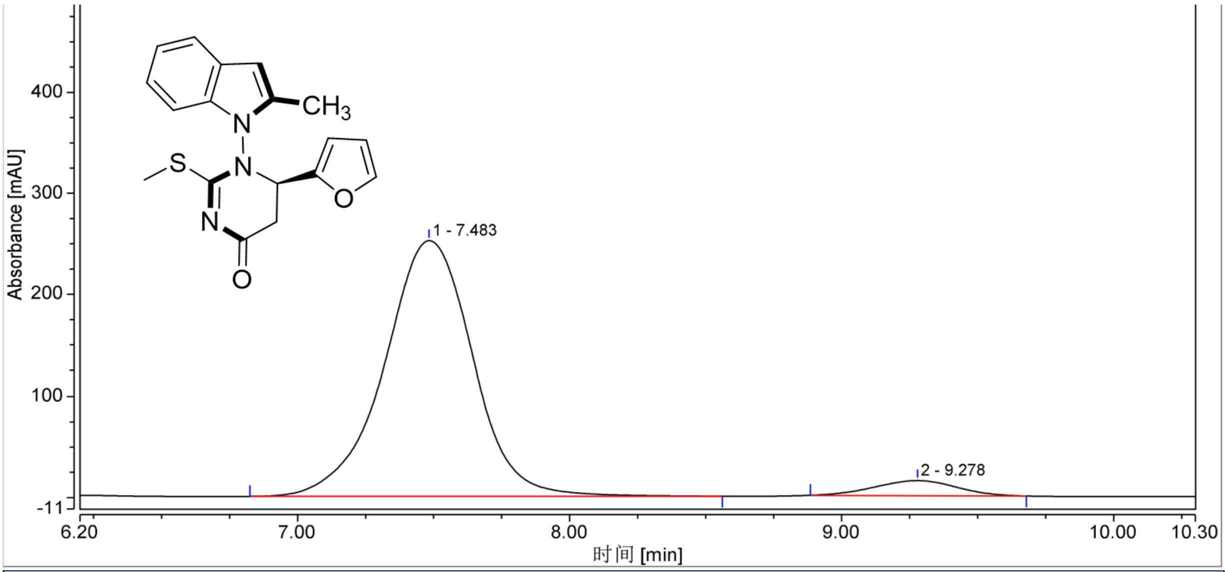

Result

Peak	RT min	Area mAU*min	Height mAU	Area %	Height %
1	7.483	94.916	251.907	94.81	94.42
2	9.278	5.196	14.895	5.19	5.58

Supplementary Figure 239. HPLC chromatogram of enantiomerically enriched 3k

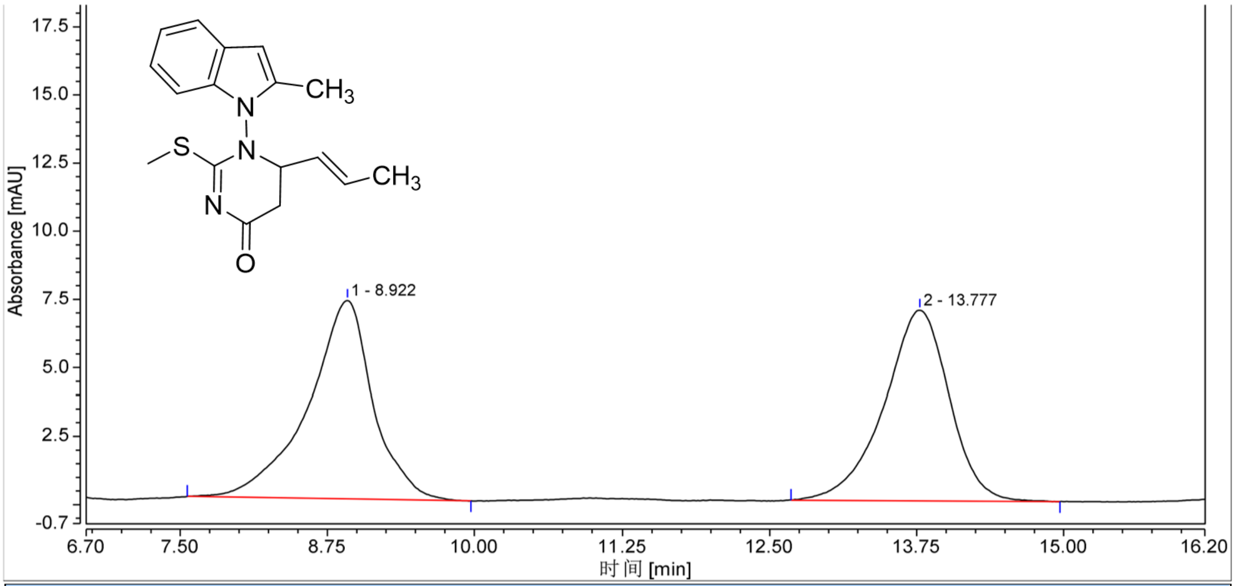

Result

Peak	RT<br>min	Area<br>mAU*min	Height<br>mAU	Area<br>%	Height<br>%
1	8.922	4.645	7.255	50.87	51.01
2	13.777	4.486	6.967	49.13	48.99

Supplementary Figure 240. HPLC chromatogram of racemic 3l

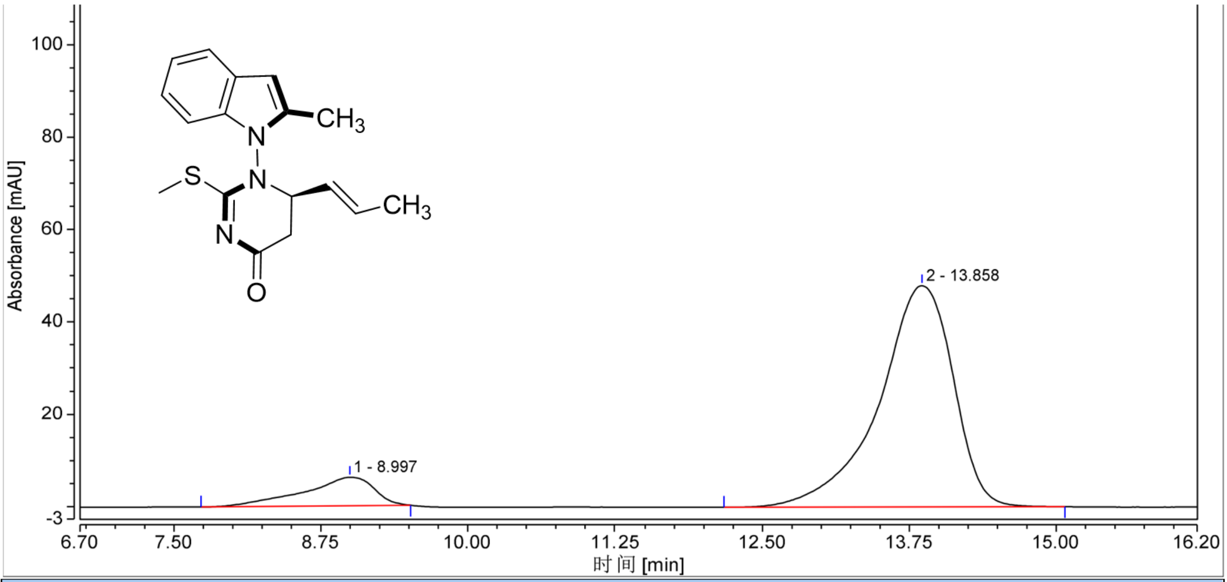

Result

Peak	RT<br>min	Area<br>mAU*min	Height<br>mAU	Area<br>%	Height<br>%
1	8.997	4.300	6.167	10.92	11.42
2	13.858	35.093	47.832	89.08	88.58

Supplementary Figure 241. HPLC chromatogram of enantiomerically enriched 3l

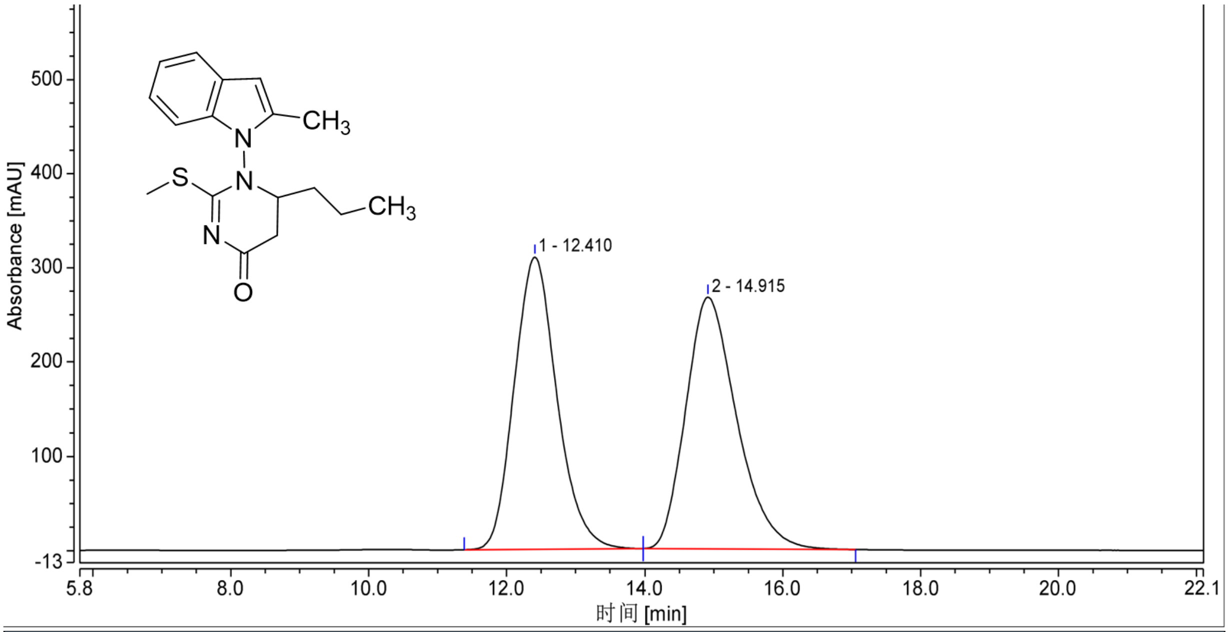

Result

Peak	RT<br>min	Area<br>mAU*min	Height<br>mAU	Area<br>%	Height<br>%
1	12.410	222.451	310.059	49.30	53.76
2	14.915	228.746	266.733	50.70	46.24

Supplementary Figure 242. HPLC chromatogram of racemic 3m

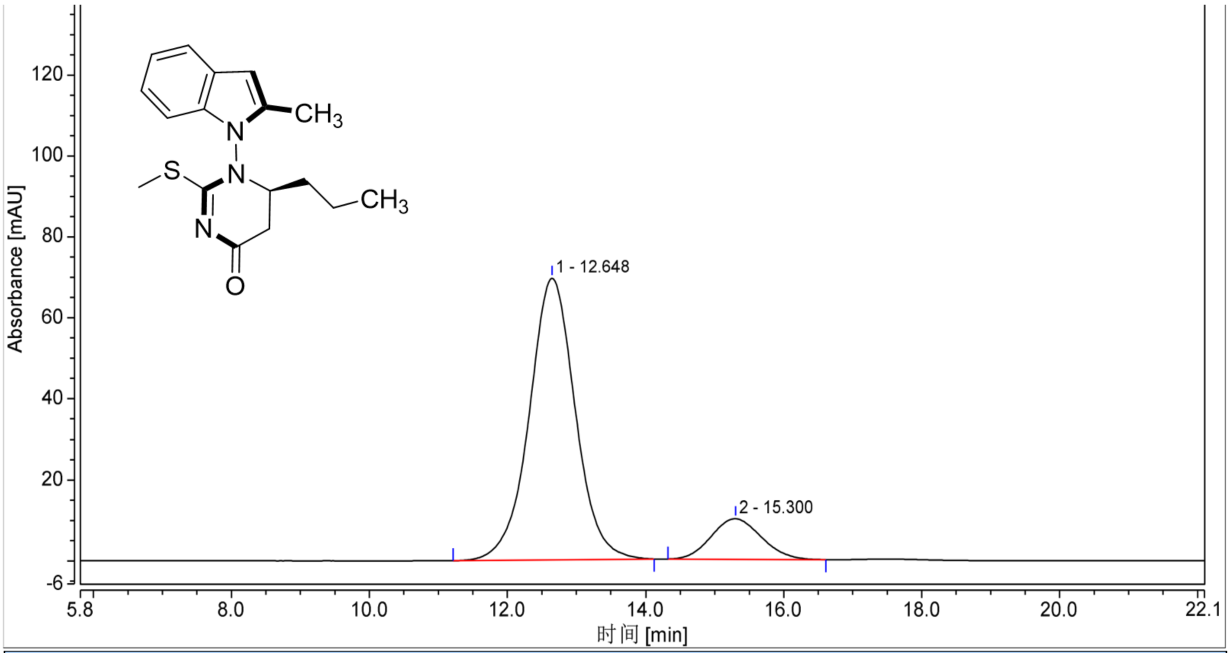

Result

Peak	RT<br>min	Area<br>mAU*min	Height<br>mAU	Area<br>%	Height<br>%
1	12.648	53.638	69.495	86.36	87.36
2	15.300	8.471	10.052	13.64	12.64

Supplementary Figure 243. HPLC chromatogram of enantiomerically enriched 3m

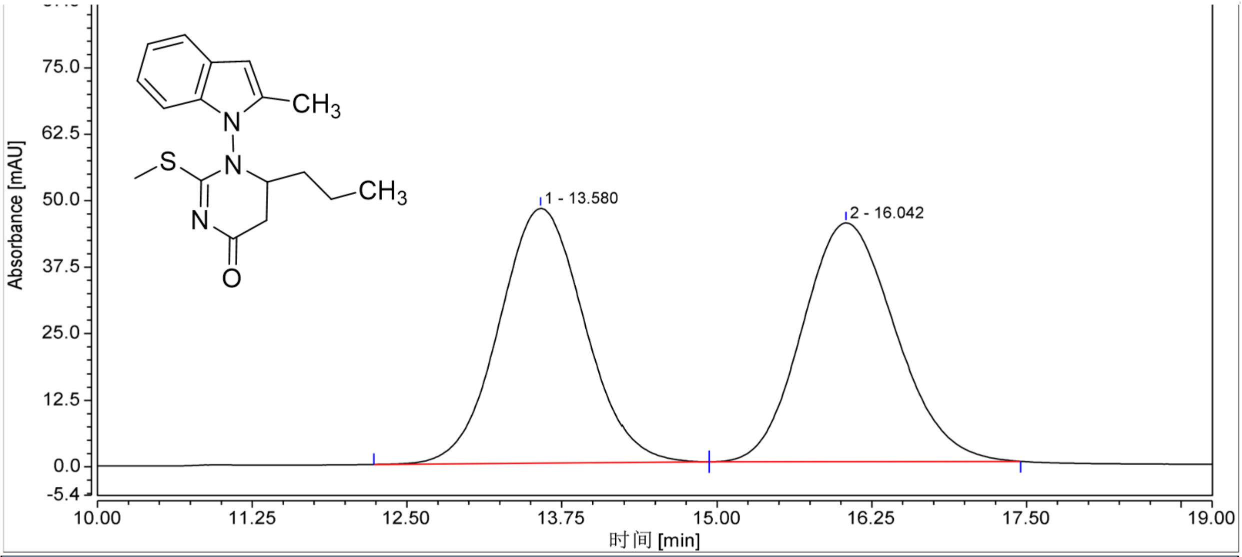

Result

Peak	RT min	Area mAU*min	Height mAU	Area %	Height %
1	13.580	38.246	47.825	49.43	51.62
2	16.042	39.120	44.819	50.57	48.38

Supplementary Figure 244. HPLC chromatogram of racemic 3m'

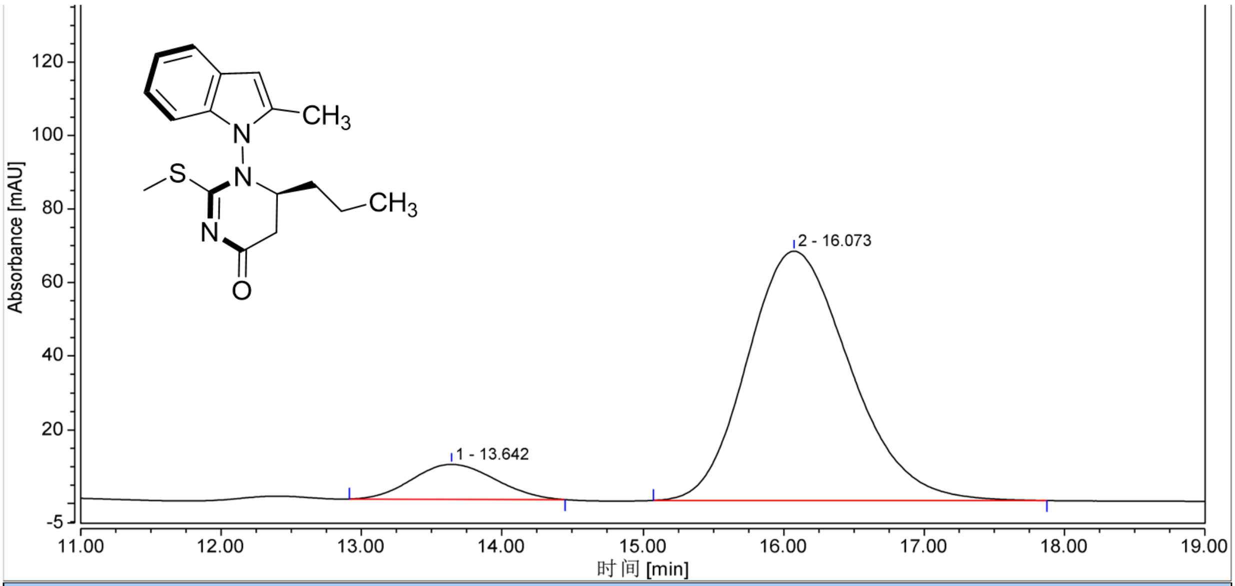

Result

Peak	RT min	Area mAU*min	Height mAU	Area %	Height %
1	13.642	6.519	9.506	10.19	12.31
2	16.073	57.480	67.701	89.81	87.69

Supplementary Figure 245. HPLC chromatogram of enantiomerically enriched 3m'

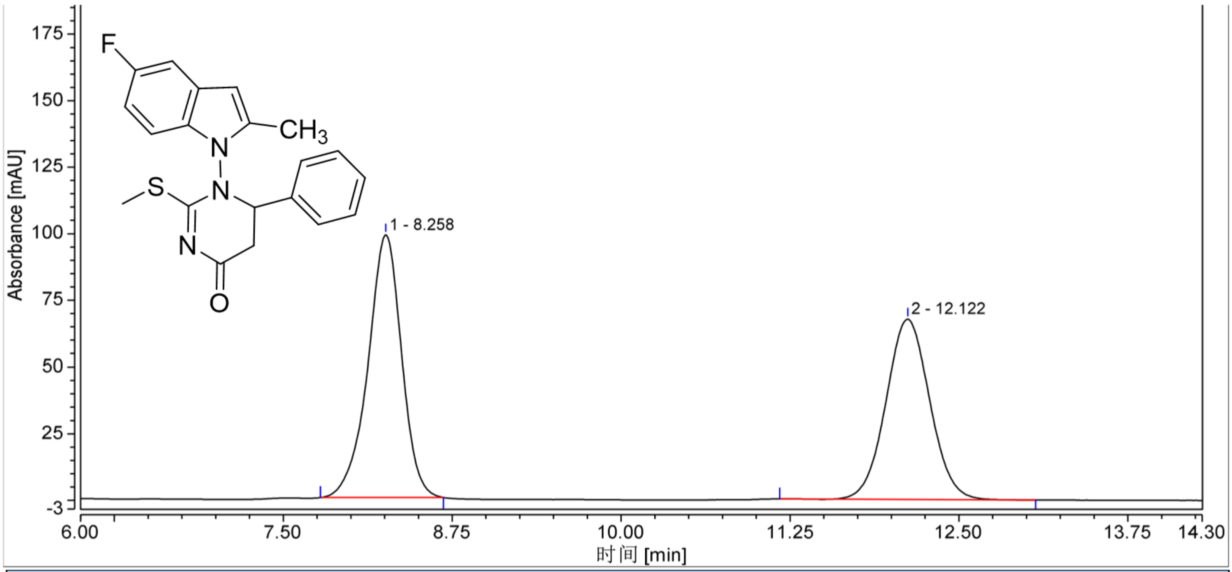

Result

Peak	RT<br>min	Area<br>mAU*min	Height<br>mAU	Area<br>%	Height<br>%
1	8.258	28.421	98.539	52.84	59.33
2	12.122	25.363	67.547	47.16	40.67

Supplementary Figure 246. HPLC chromatogram of racemic 3n

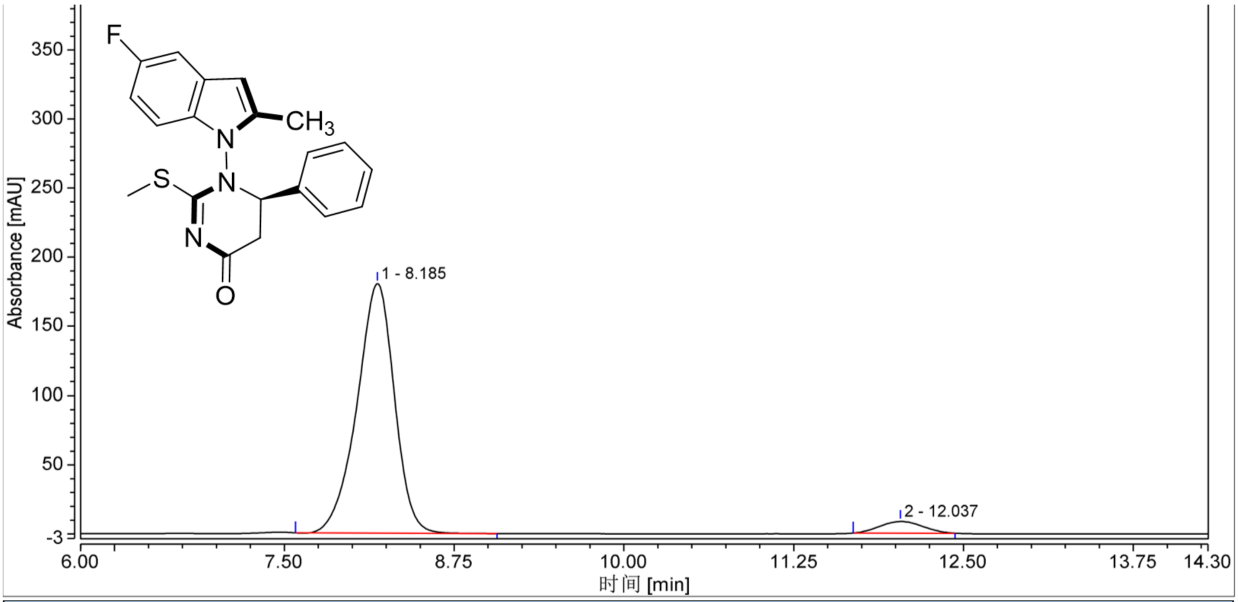

Result

Peak	RT<br>min	Area<br>mAU*min	Height<br>mAU	Area<br>%	Height<br>%
1	8.185	57.601	180.262	94.94	95.53
2	12.037	3.069	8.437	5.06	4.47

Supplementary Figure 247. HPLC chromatogram of enantiomerically enriched 3n

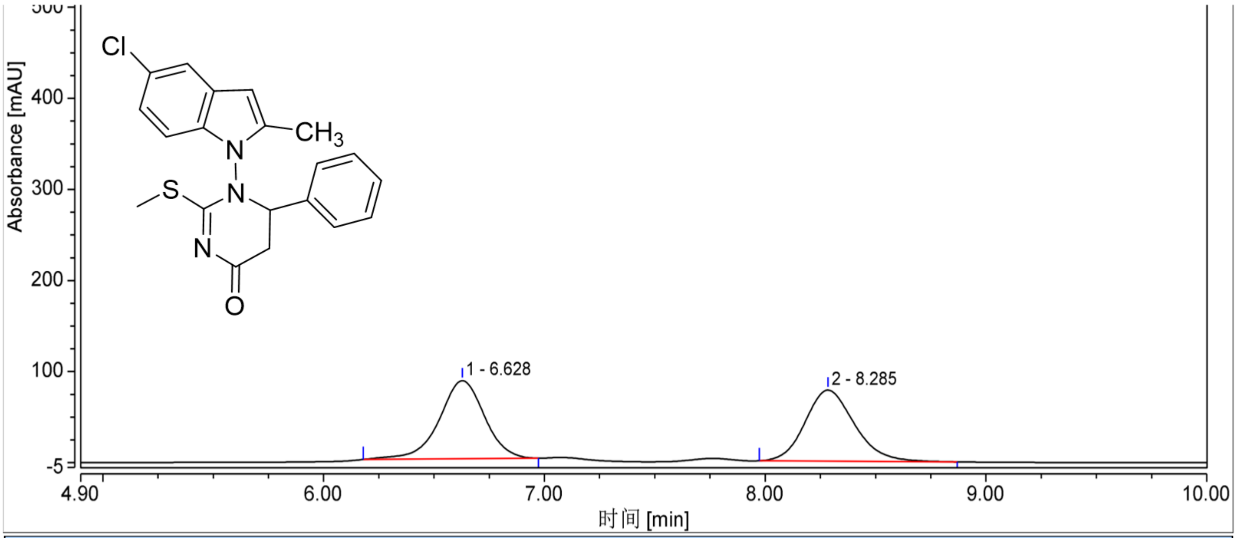

Result					
Peak	RT<br>min	Area<br>mAU*min	Height<br>mAU	Area<br>%	Height<br>%
1	6.628	20.422	85.797	50.28	52.38
2	8.285	20.193	77.992	49.72	47.62

Supplementary Figure 248. HPLC chromatogram of racemic 3o

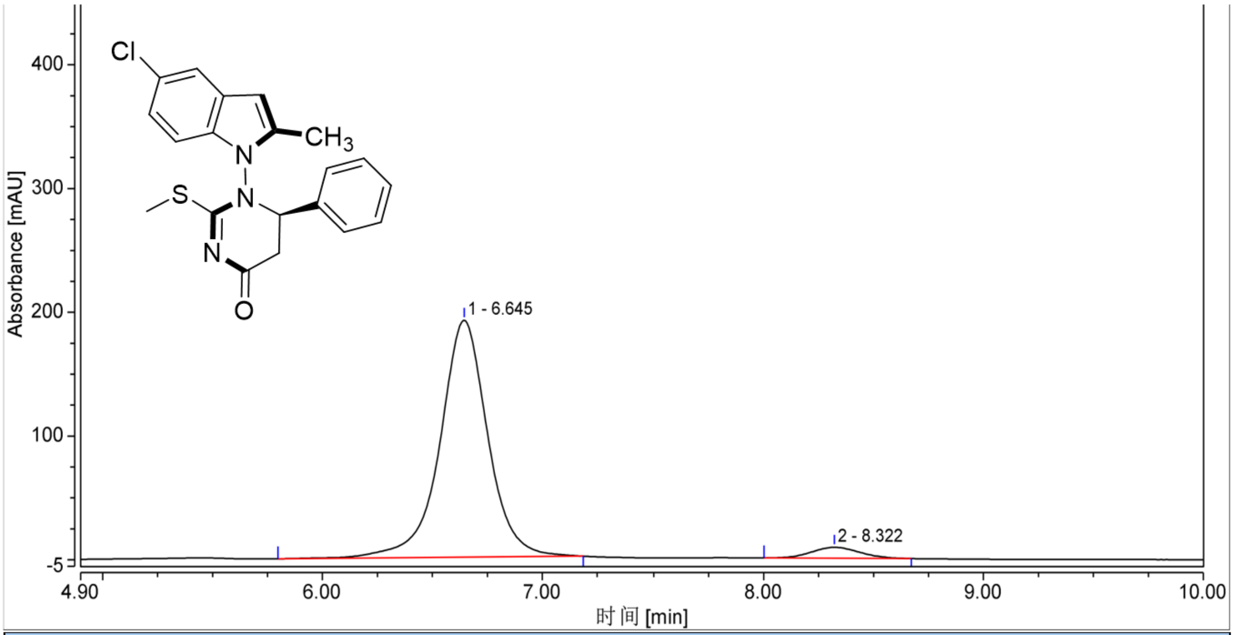

Result					
Peak	RT<br>min	Area<br>mAU*min	Height<br>mAU	Area<br>%	Height<br>%
1	6.645	48.339	191.490	95.51	95.57
2	8.322	2.274	8.875	4.49	4.43

Supplementary Figure 249. HPLC chromatogram of enantiomerically enriched 3o

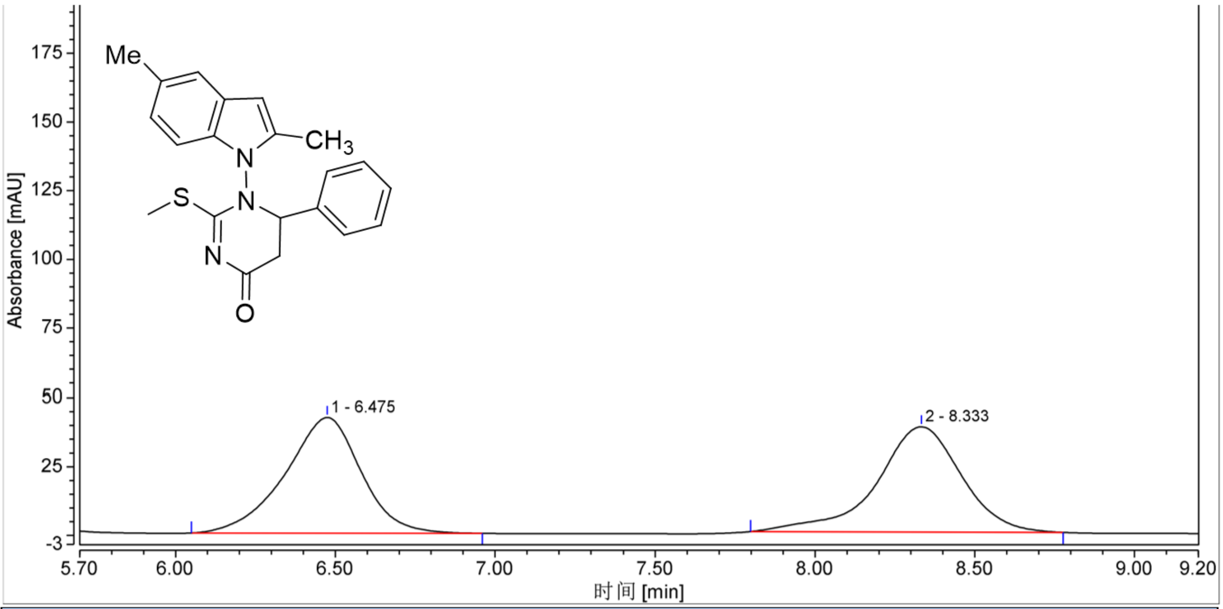

Result

Peak	RT min	Area mAU*min	Height mAU	Area %	Height %
1	6.475	11.617	42.114	49.54	52.35
2	8.333	11.832	38.332	50.46	47.65

Supplementary Figure 250. HPLC chromatogram of racemic 3p

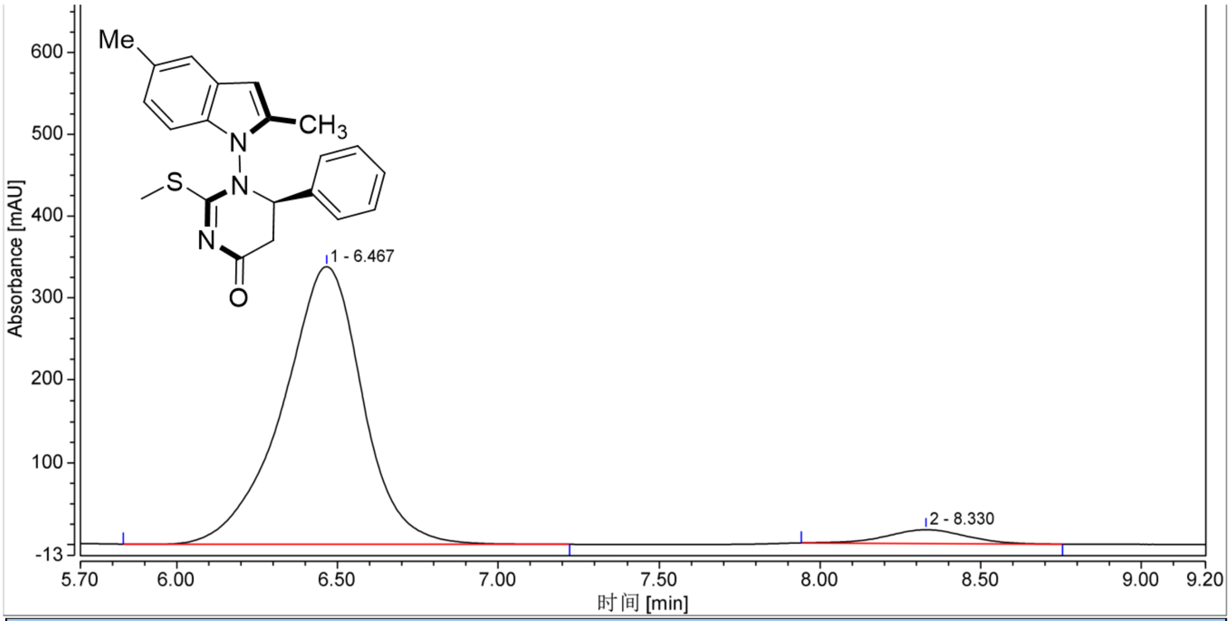

Result

Peak	RT min	Area mAU*min	Height mAU	Area %	Height %
1	6.467	97.612	337.644	95.29	95.24
2	8.330	4.828	16.863	4.71	4.76

Supplementary Figure 251. HPLC chromatogram of enantiomerically enriched 3p

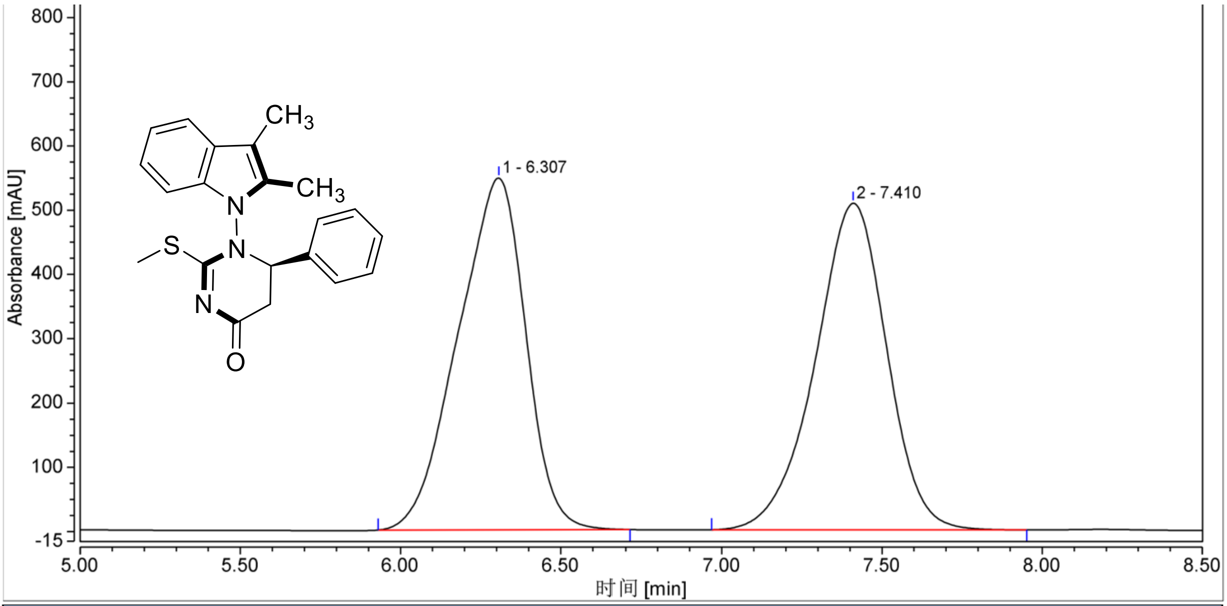

Peak	RT<br>min	Area<br>mAU*min	Height<br>mAU	Area<br>%	Height<br>%
1	6.307	133.832	547.520	50.42	51.87
2	7.410	131.618	508.109	49.58	48.13

Supplementary Figure 252. HPLC chromatogram of racemic 3q

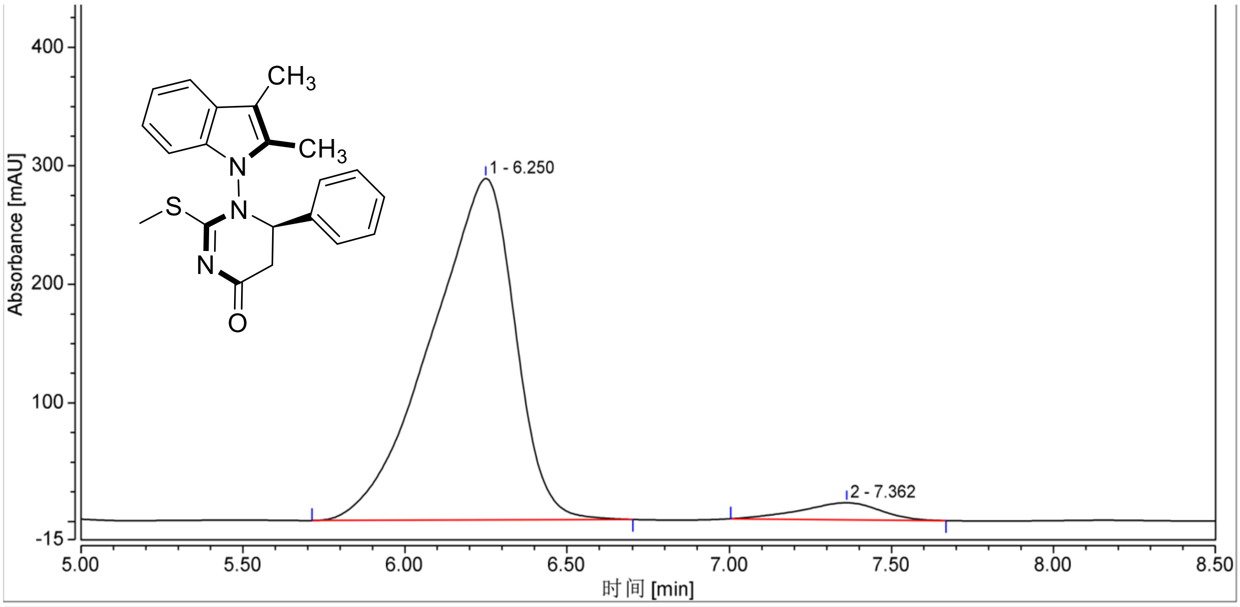

Peak	RT<br>min	Area<br>mAU*min	Height<br>mAU	Area<br>%	Height<br>%
1	6.250	86.704	287.912	95.52	95.26
2	7.362	4.063	14.315	4.48	4.74

Supplementary Figure 253. HPLC chromatogram of enantiomerically enriched 3q

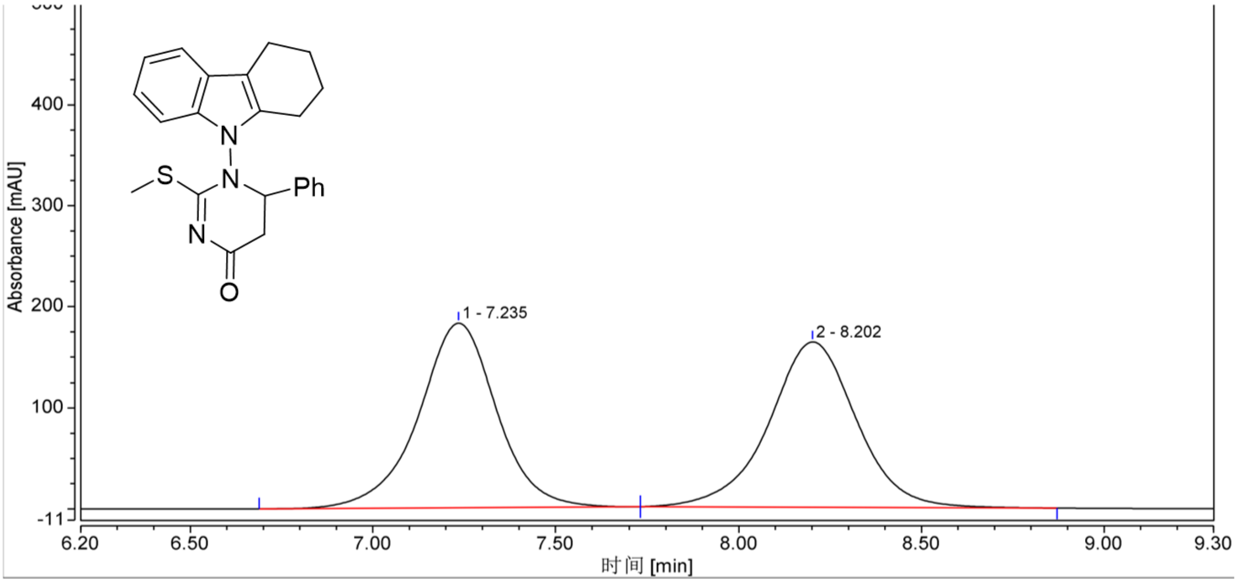

Result

Peak	RT<br>min	Area<br>mAU*min	Height<br>mAU	Area<br>%	Height<br>%
1	7.235	45.353	182.304	49.84	52.75
2	8.202	45.636	163.291	50.16	47.25

Supplementary Figure 254. HPLC chromatogram of racemic 3r

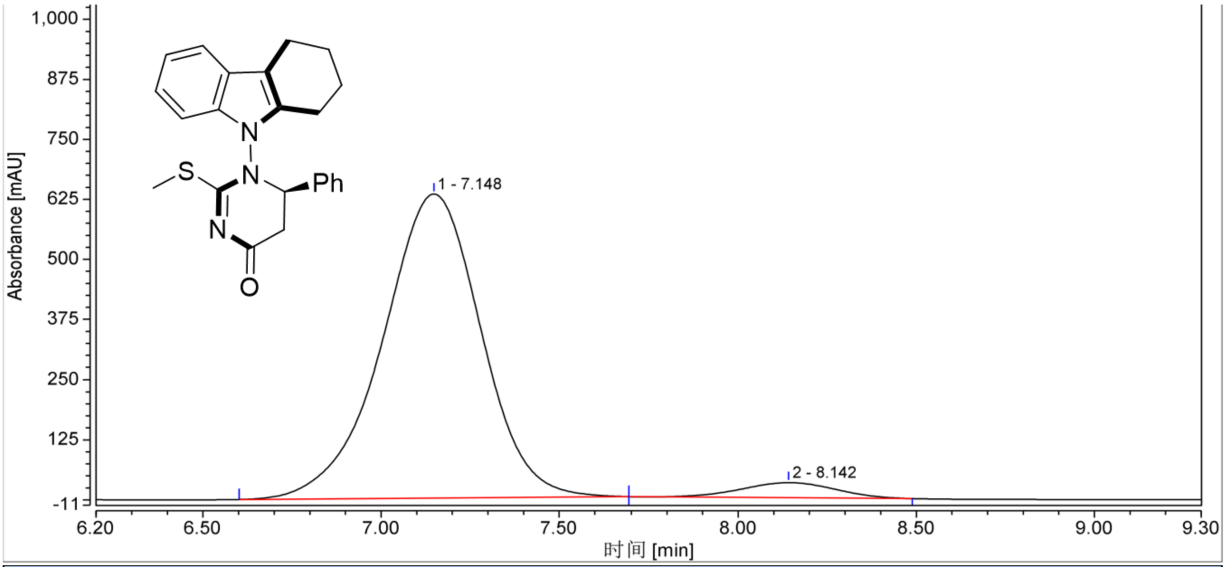

Result

Peak	RT<br>min	Area<br>mAU*min	Height<br>mAU	Area<br>%	Height<br>%
1	7.148	202.472	632.344	95.45	95.26
2	8.142	9.662	31.483	4.55	4.74

Supplementary Figure 255. HPLC chromatogram of enantiomerically enriched 3r

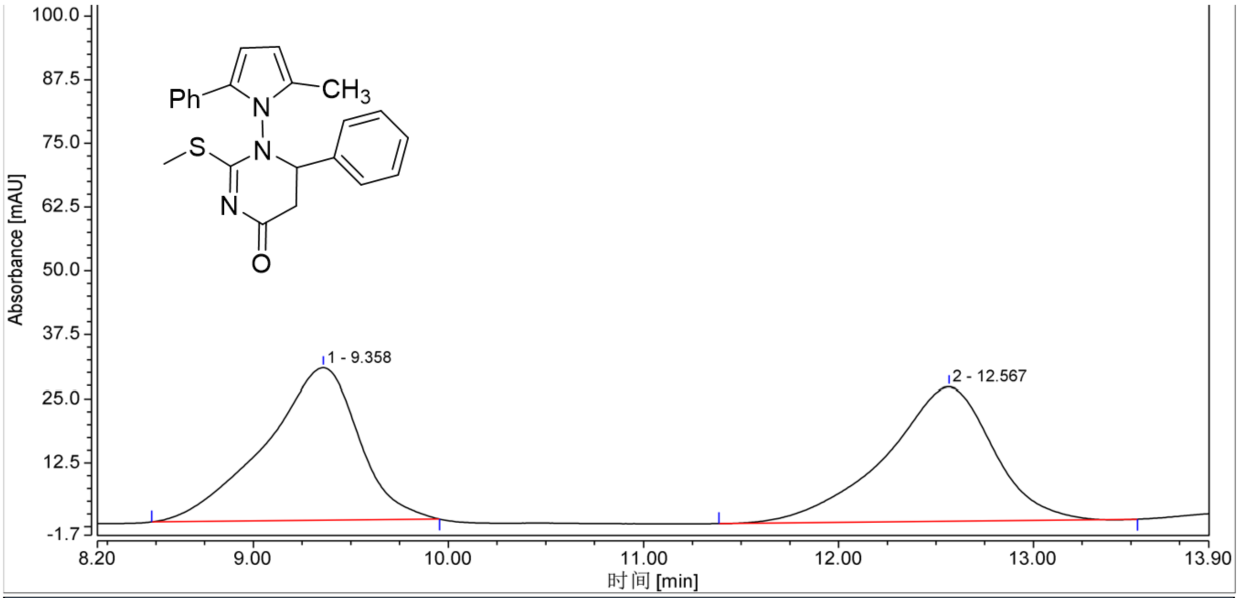

Result

Peak	RT min	Area mAU*min	Height mAU	Area %	Height %
1	9.358	16.630	29.689	49.79	53.12
2	12.567	16.772	26.200	50.21	46.88

Supplementary Figure 256. HPLC chromatogram of racemic 3s

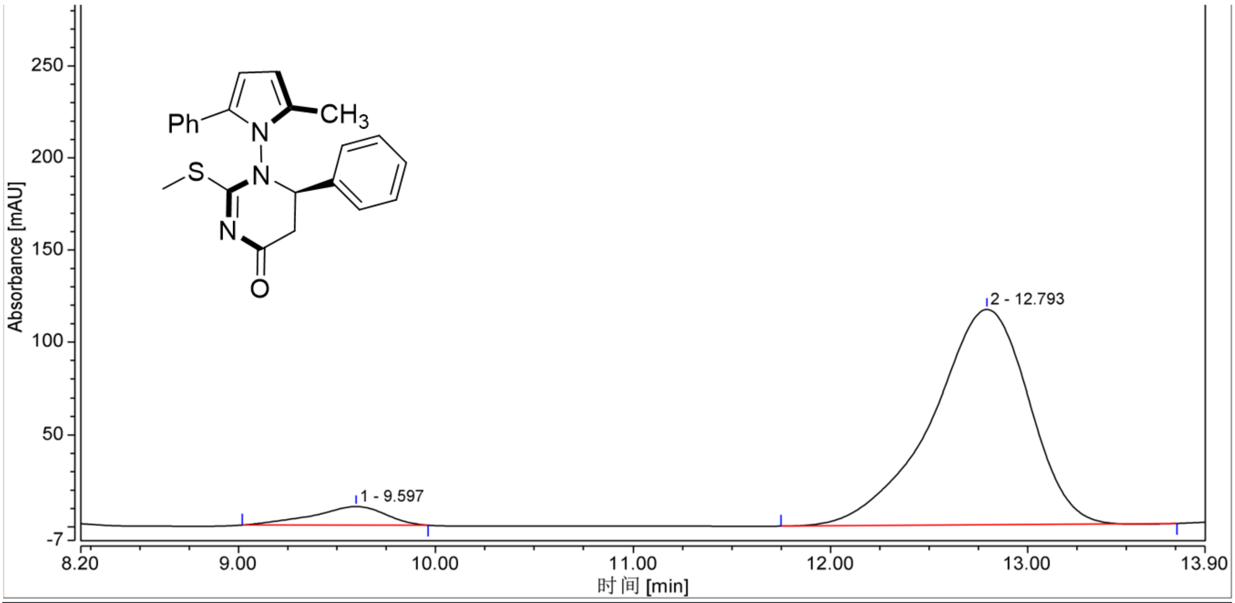

Result

Peak	RT min	Area mAU*min	Height mAU	Area %	Height %
1	9.597	4.305	10.118	6.08	7.99
2	12.793	66.457	116.481	93.92	92.01

Supplementary Figure 257. HPLC chromatogram of enantiomerically enriched 3s

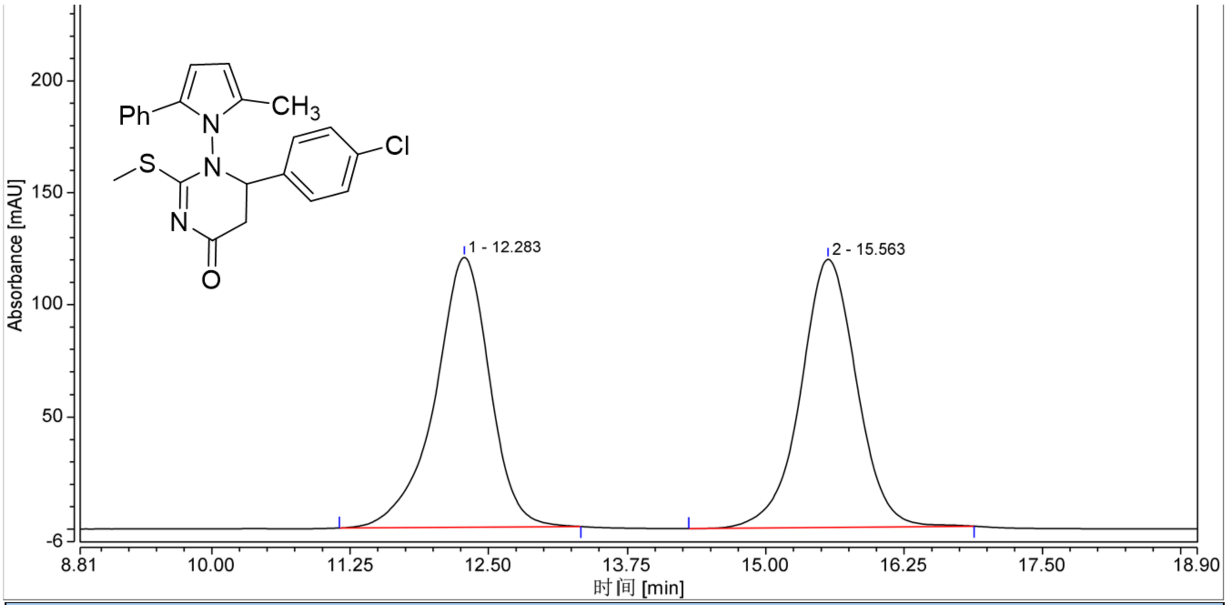

Result					
Peak	RT min	Area mAU*min	Height mAU	Area %	Height %
1	12.283	69.292	120.287	49.69	50.16
2	15.563	70.161	119.517	50.31	49.84

Supplementary Figure 258. HPLC chromatogram of racemic 3t

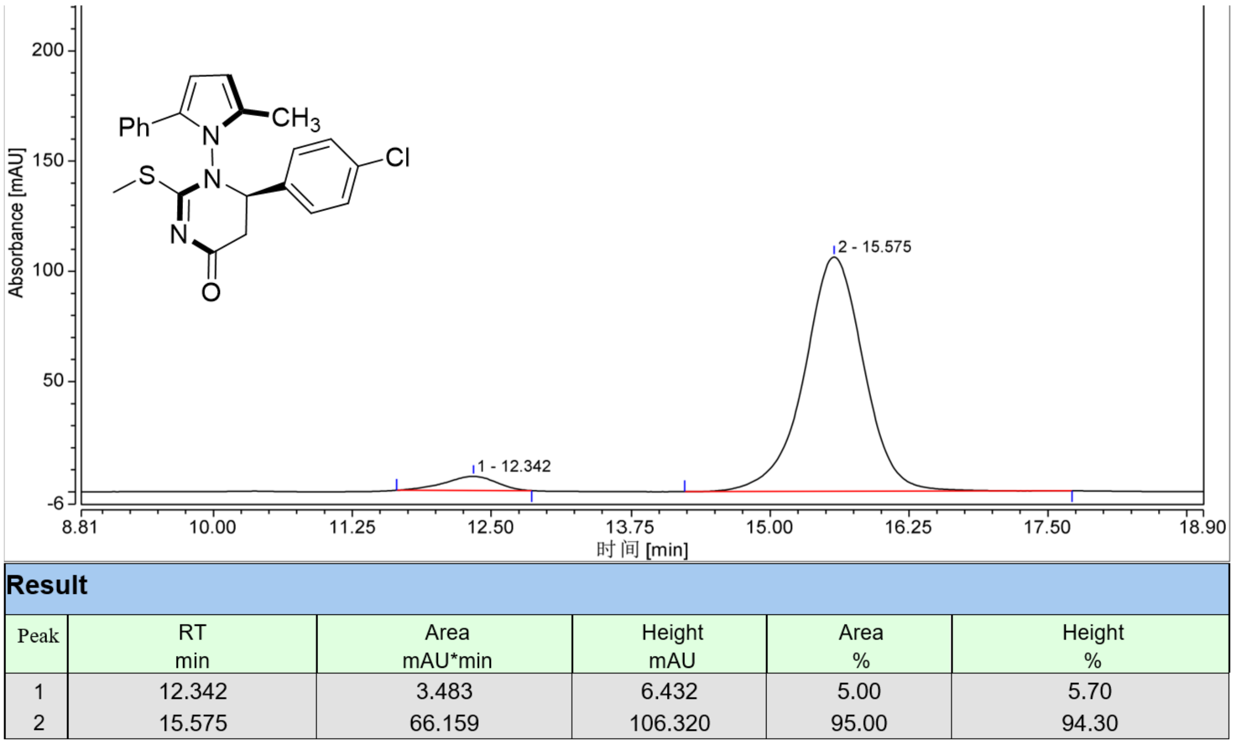

Result					
Peak	RT min	Area mAU*min	Height mAU	Area %	Height %
1	12.342	3.483	6.432	5.00	5.70
2	15.575	66.159	106.320	95.00	94.30

Supplementary Figure 259. HPLC chromatogram of enantiomerically enriched 3t

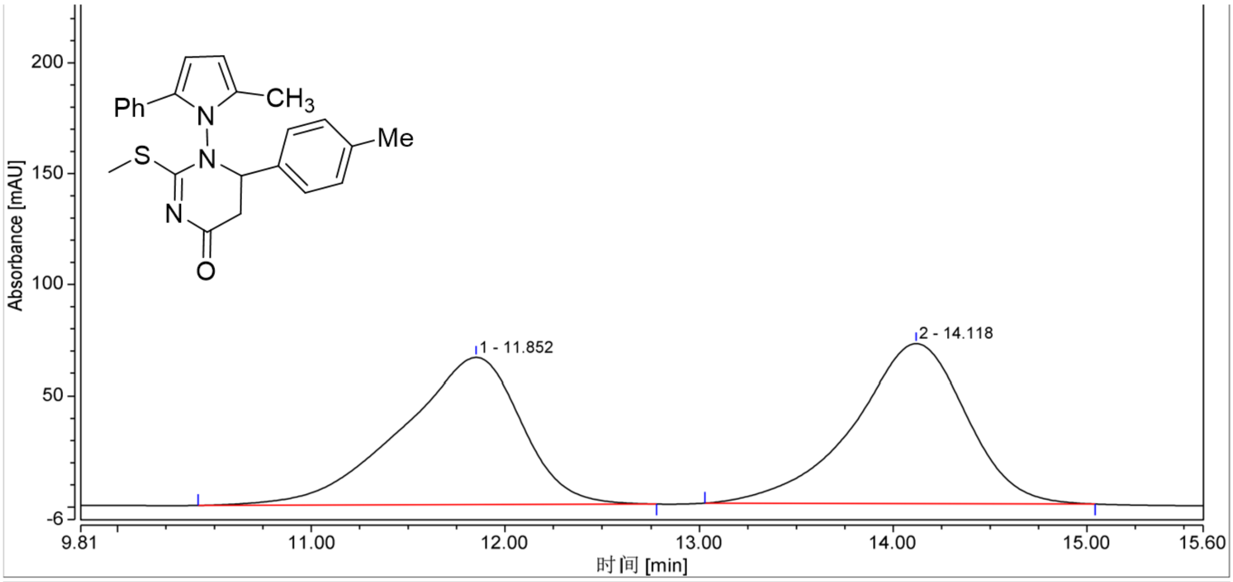

Result

Peak	RT<br>min	Area<br>mAU*min	Height<br>mAU	Area<br>%	Height<br>%
1	11.852	47.489	66.028	49.50	47.91
2	14.118	48.451	71.785	50.50	52.09

Supplementary Figure 260. HPLC chromatogram of racemic 3u

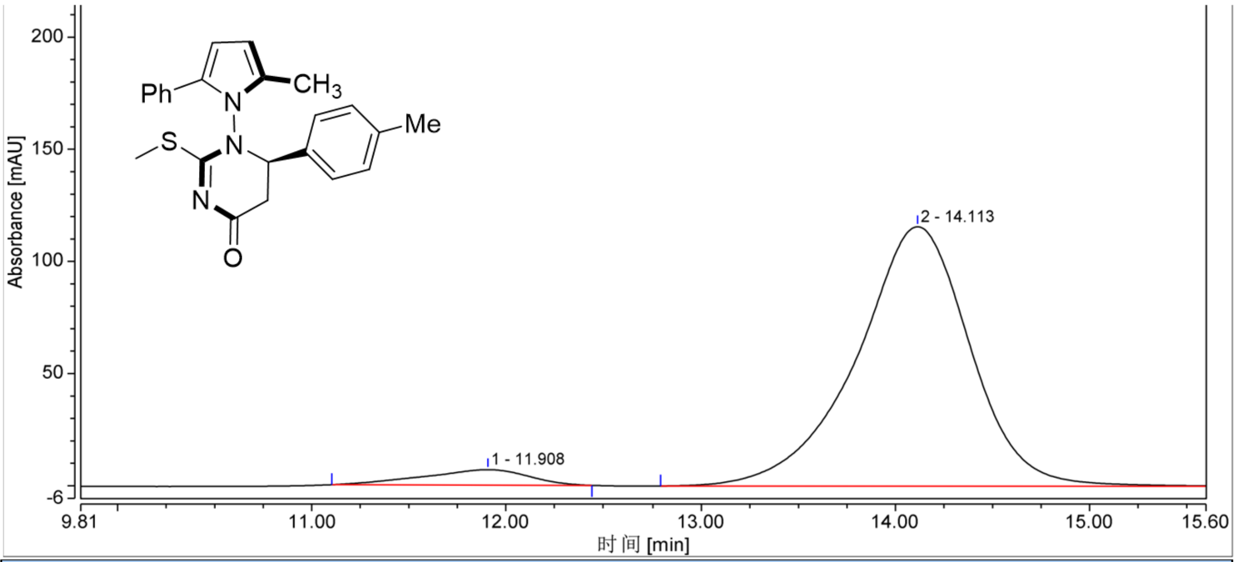

Result

Peak	RT<br>min	Area<br>mAU*min	Height<br>mAU	Area<br>%	Height<br>%
1	11.908	4.270	6.884	5.16	5.62
2	14.113	78.537	115.681	94.84	94.38

Supplementary Figure 261. HPLC chromatogram of enantiomerically enriched 3u

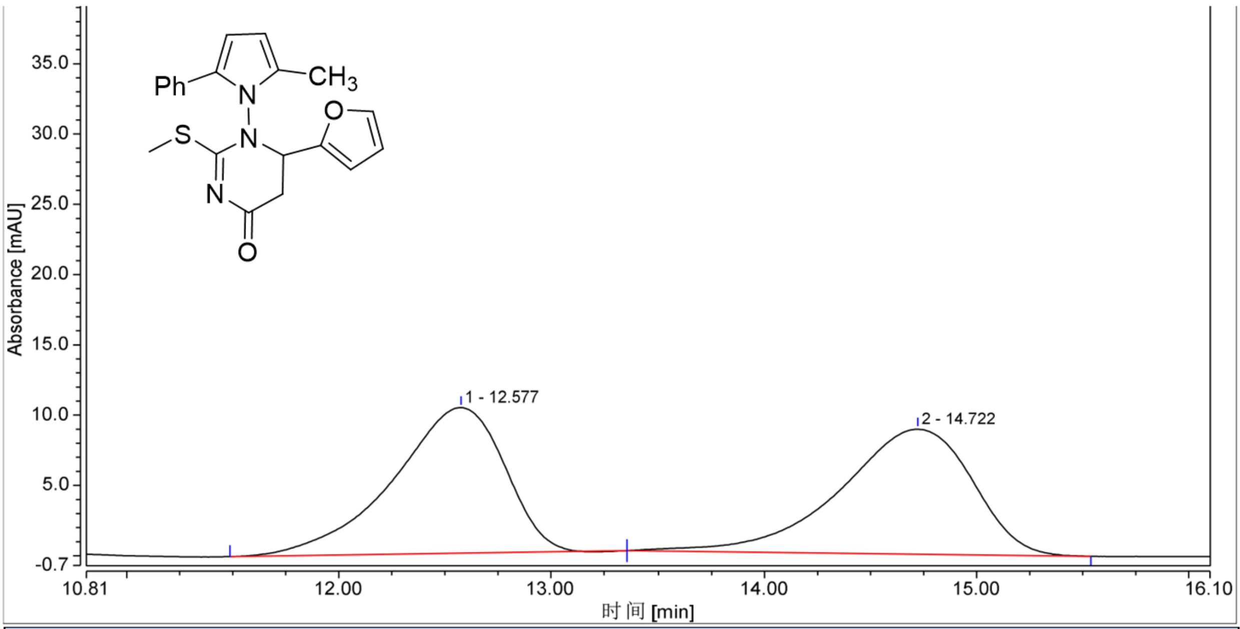

Result

Peak	RT<br>min	Area<br>mAU*min	Height<br>mAU	Area<br>%	Height<br>%
1	12.577	6.308	10.352	49.54	53.77
2	14.722	6.425	8.900	50.46	46.23

Supplementary Figure 262. HPLC chromatogram of racemic 3v

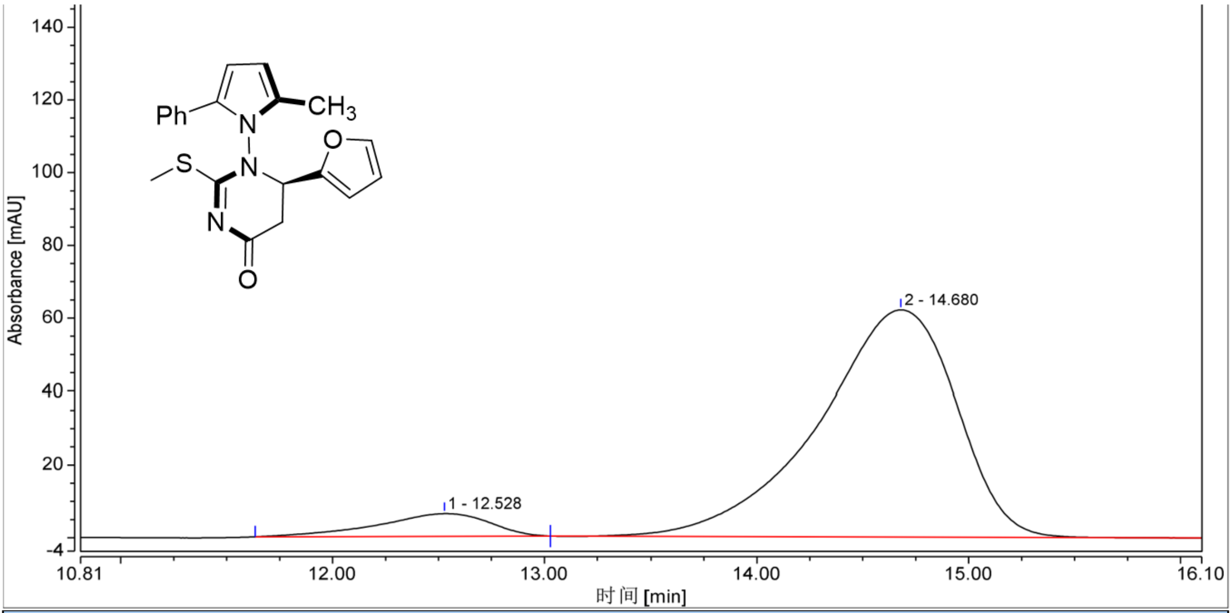

Result

Peak	RT<br>min	Area<br>mAU*min	Height<br>mAU	Area<br>%	Height<br>%
1	12.528	3.795	6.243	7.49	9.14
2	14.680	46.851	62.054	92.51	90.86

Supplementary Figure 263. HPLC chromatogram of enantiomerically enriched 3v

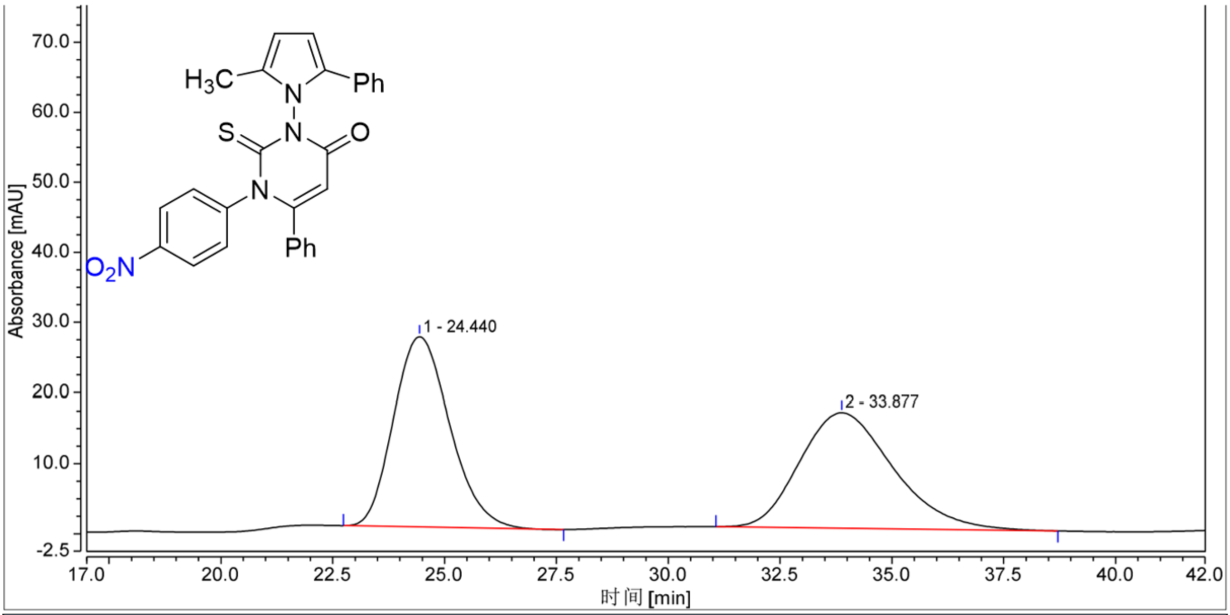

Result

Peak	RT<br>min	Area<br>mAU*min	Height<br>mAU	Area<br>%	Height<br>%
1	24.440	39.409	26.981	49.63	62.06
2	33.877	39.990	16.498	50.37	37.94

Supplementary Figure 264. HPLC chromatogram of racemic 7a

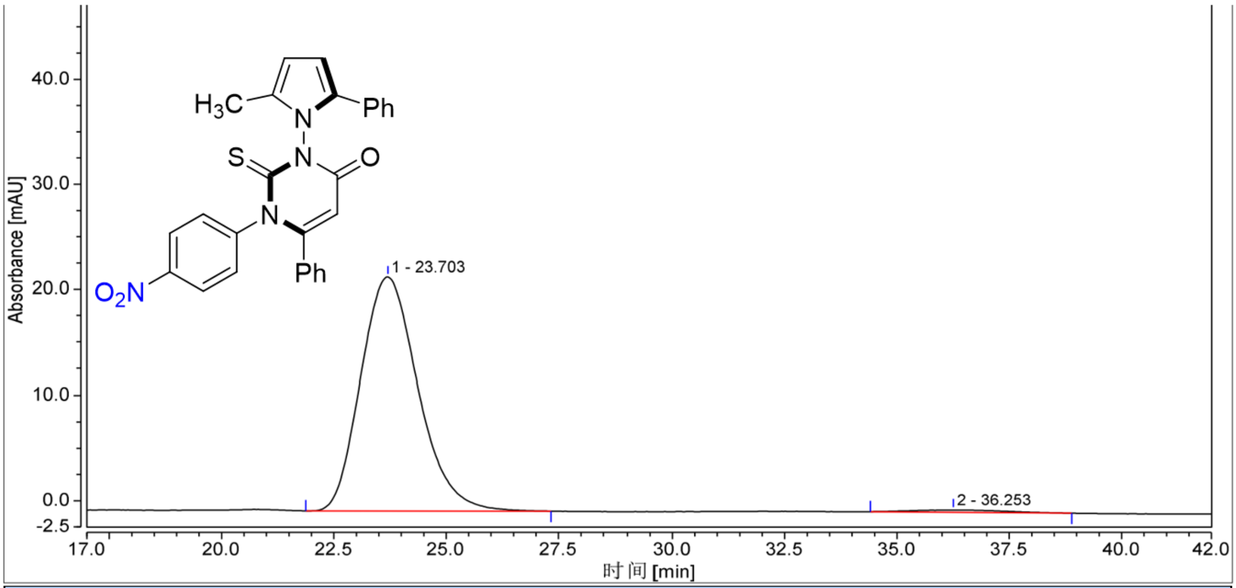

Result

Peak	RT<br>min	Area<br>mAU*min	Height<br>mAU	Area<br>%	Height<br>%
1	23.703	32.753	22.159	98.43	98.99
2	36.253	0.523	0.226	1.57	1.01

Supplementary Figure 265. HPLC chromatogram of enantiomerically enriched 7a

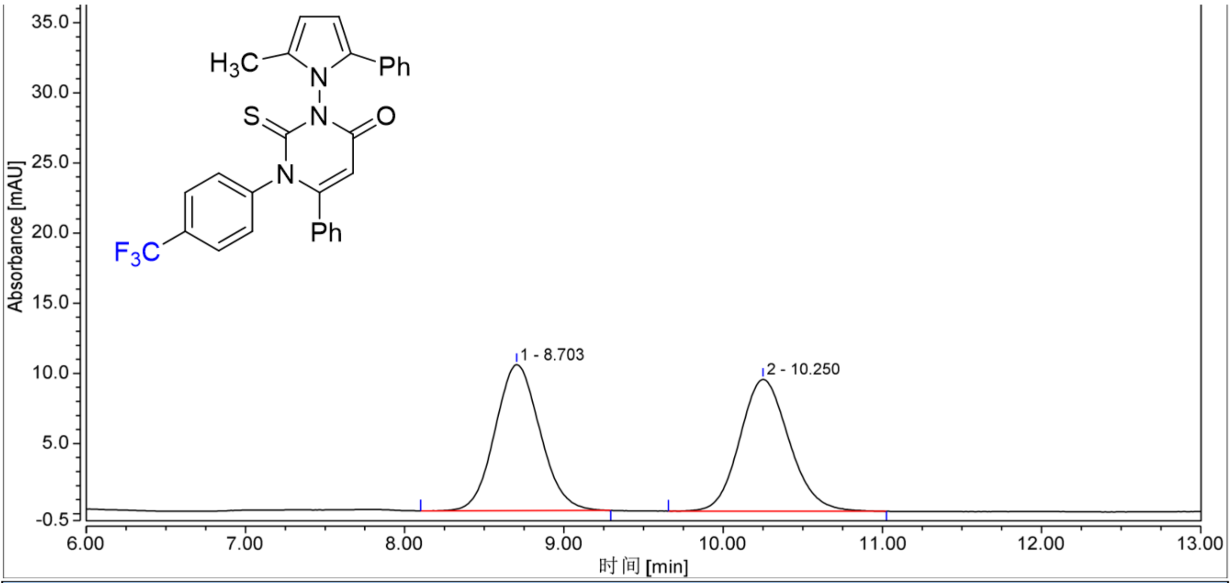

Result

Peak	RT min	Area mAU*min	Height mAU	Area %	Height %
1	8.703	3.408	10.402	50.21	52.56
2	10.250	3.379	9.391	49.79	47.44

Supplementary Figure 266. HPLC chromatogram of racemic 7b

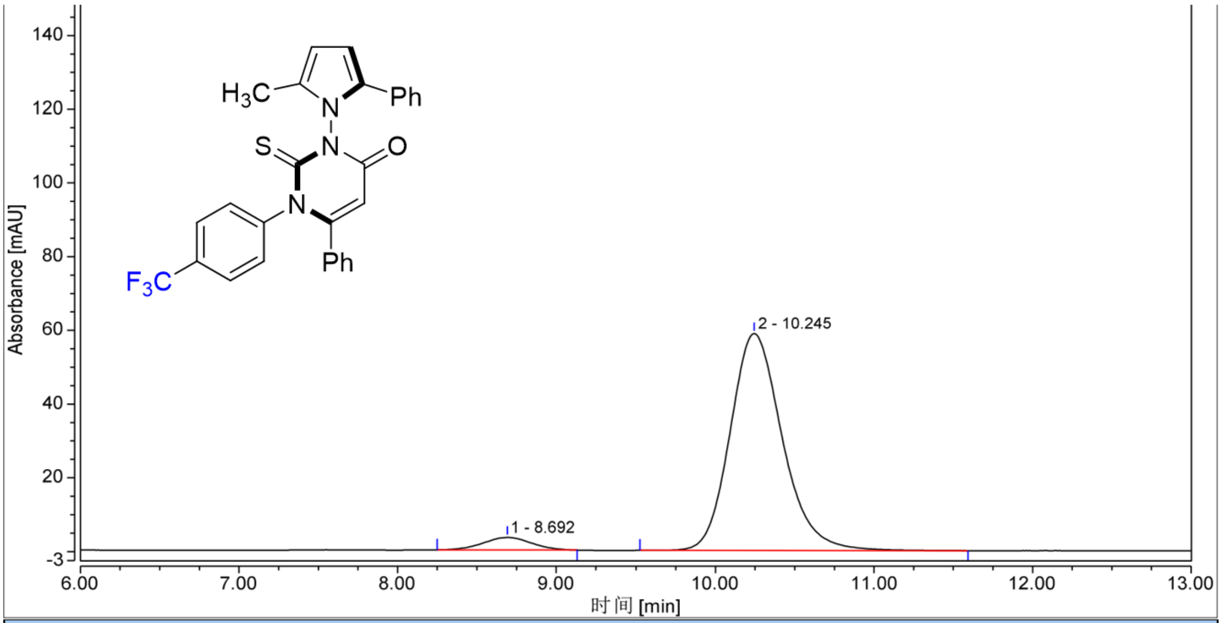

Result

Peak	RT min	Area mAU*min	Height mAU	Area %	Height %
1	8.692	1.196	3.393	5.06	5.45
2	10.245	22.447	58.861	94.94	94.55

Supplementary Figure 267. HPLC chromatogram of enantiomerically enriched 7b

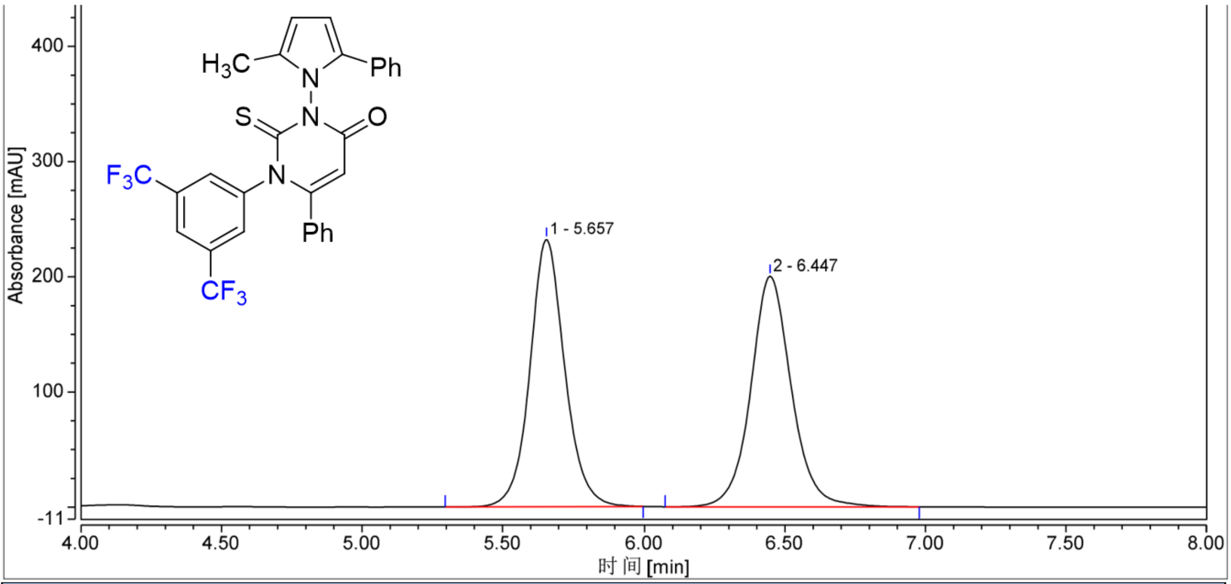

Result

Peak	RT min	Area mAU*min	Height mAU	Area %	Height %
1	5.657	33.361	232.125	49.86	53.69
2	6.447	33.554	200.236	50.14	46.31

Supplementary Figure 268. HPLC chromatogram of racemic 7c

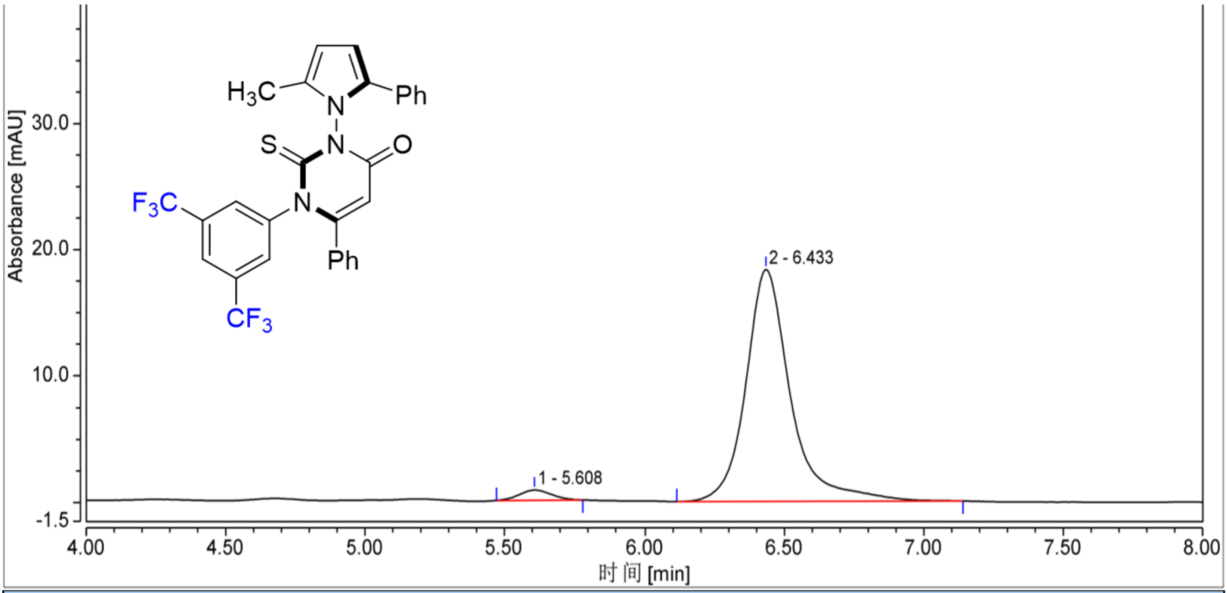

Result

Peak	RT min	Area mAU*min	Height mAU	Area %	Height %
1	5.608	0.108	0.813	3.07	4.24
2	6.433	3.417	18.353	96.93	95.76

Supplementary Figure 269. HPLC chromatogram of enantiomerically enriched 7c

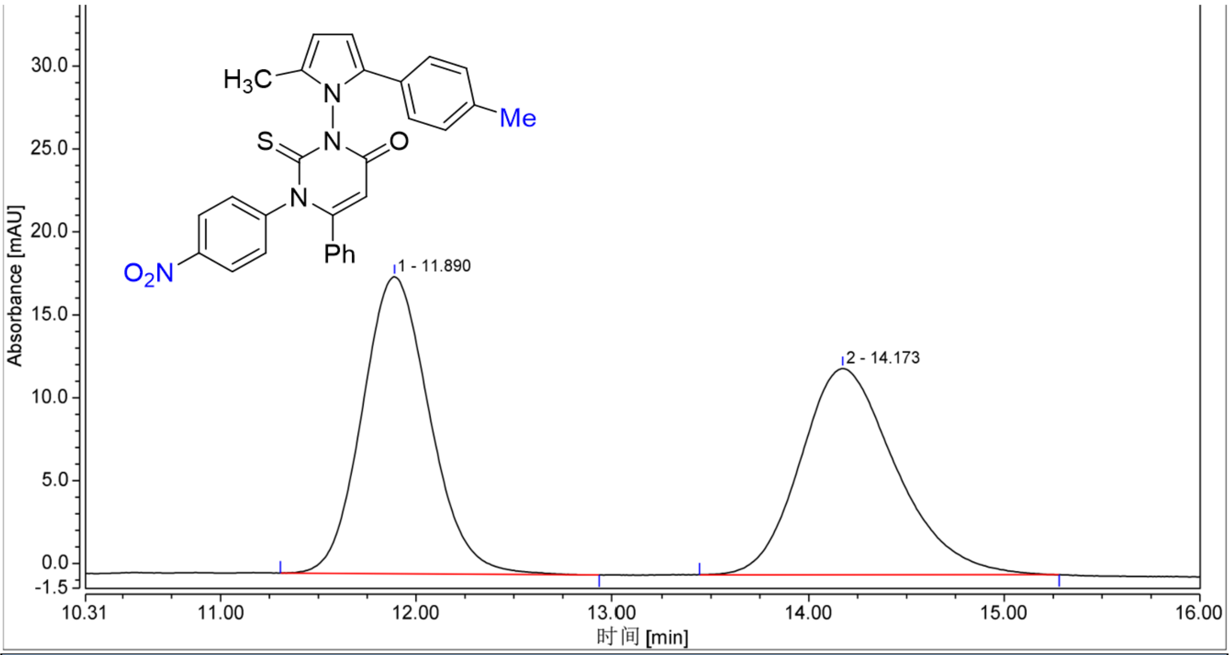

Result

Peak	RT<br>min	Area<br>mAU*min	Height<br>mAU	Area<br>%	Height<br>%
1	11.890	7.151	17.911	50.33	59.03
2	14.173	7.057	12.434	49.67	40.97

Supplementary Figure 270. HPLC chromatogram of racemic 7d

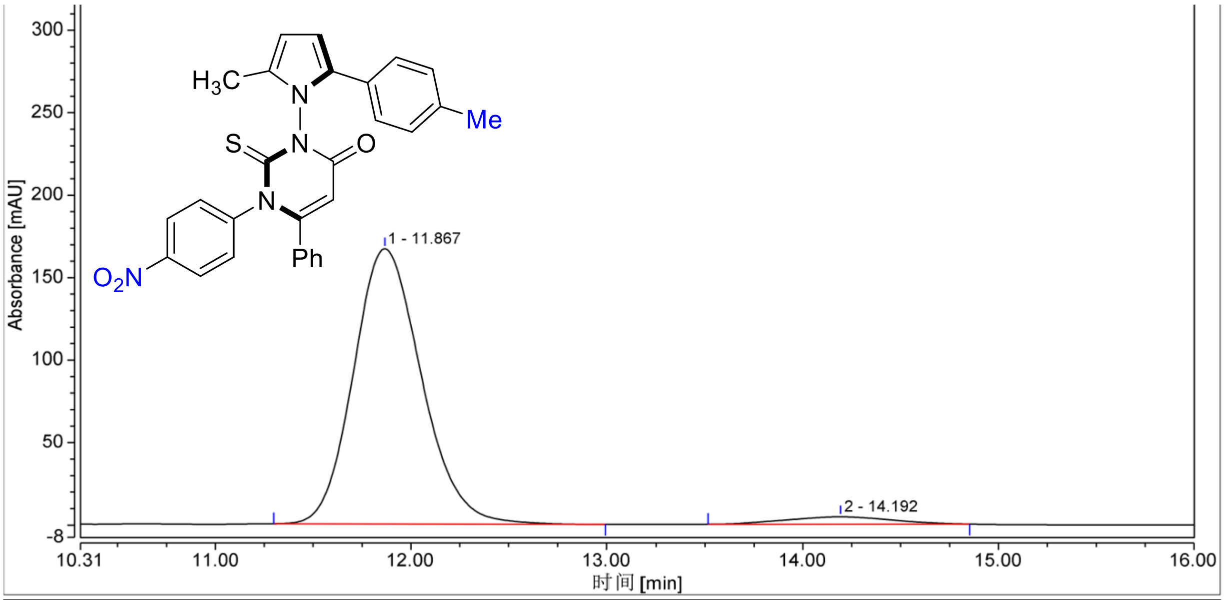

Result

Peak	RT<br>min	Area<br>mAU*min	Height<br>mAU	Area<br>%	Height<br>%
1	11.867	67.314	166.842	96.05	97.39
2	14.192	2.770	4.466	3.95	2.61

Supplementary Figure 271. HPLC chromatogram of enantiomerically enriched 7d

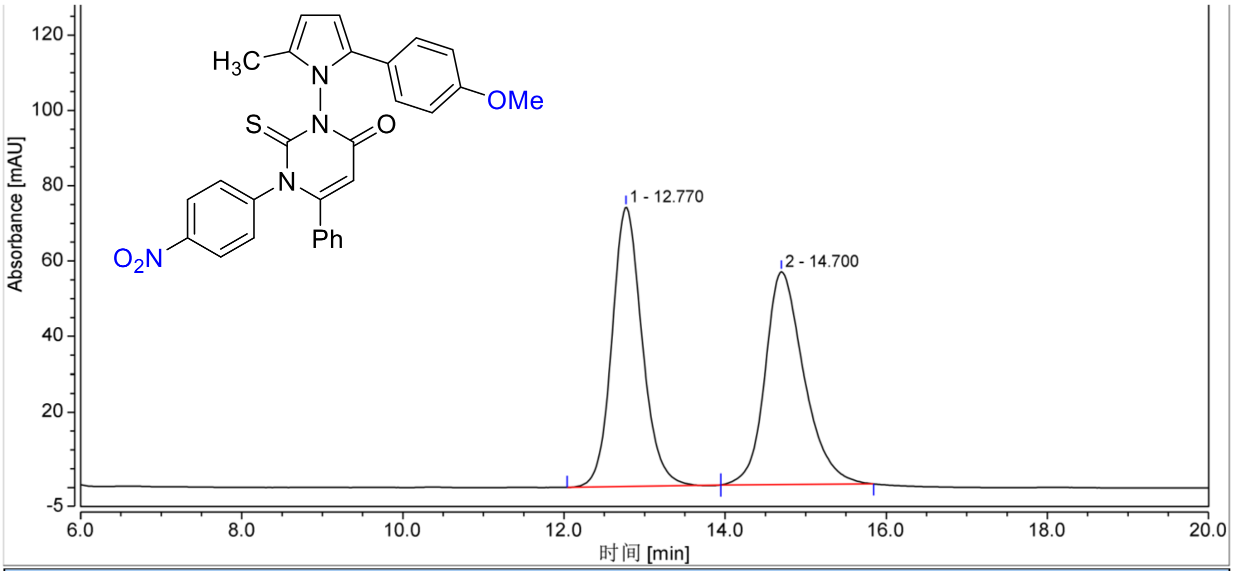

Result

Peak	RT min	Area mAU*min	Height mAU	Area %	Height %
1	12.770	32.052	74.025	50.39	56.79
2	14.700	31.555	56.328	49.61	43.21

Supplementary Figure 272. HPLC chromatogram of racemic 7e

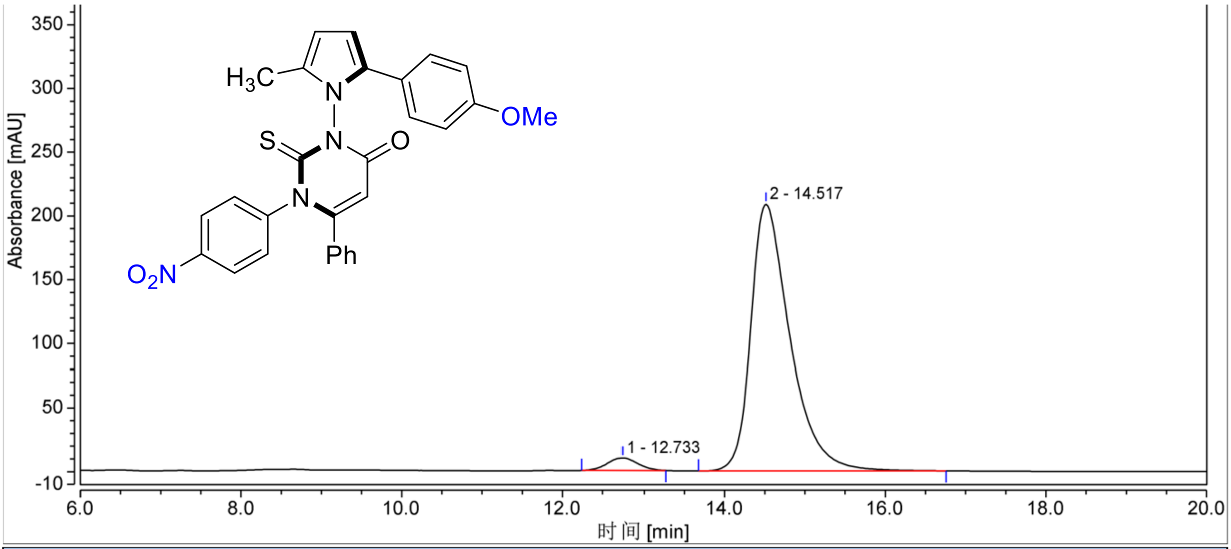

Result

Peak	RT min	Area mAU*min	Height mAU	Area %	Height %
1	12.733	4.107	9.788	3.36	4.48
2	14.517	117.986	208.608	96.64	95.52

Supplementary Figure 273. HPLC chromatogram of enantiomerically enriched 7e

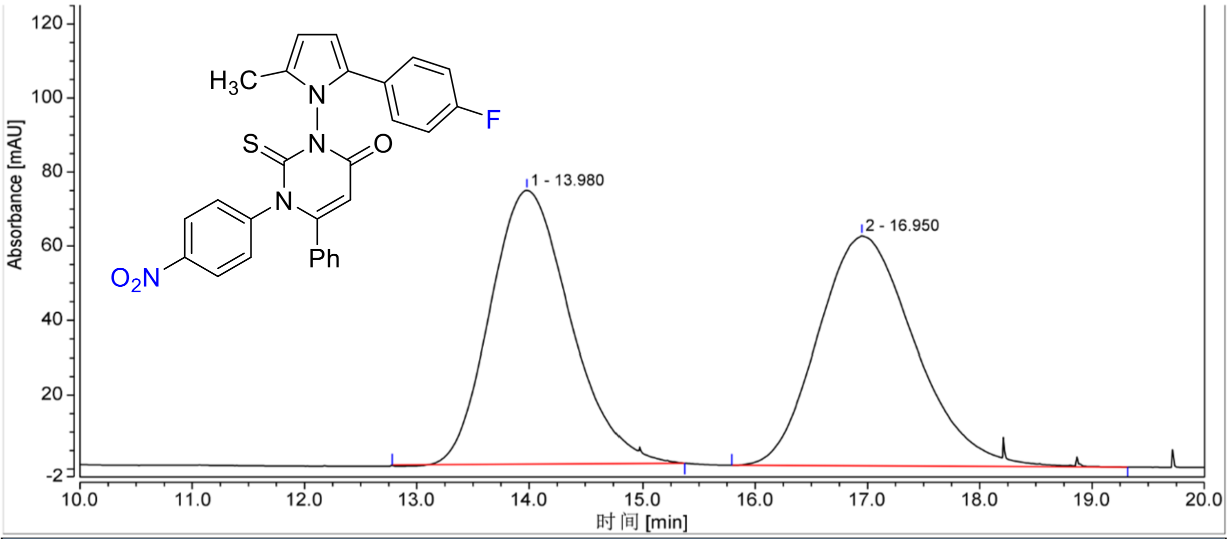

Result					
Peak	RT<br>min	Area<br>mAU*min	Height<br>mAU	Area<br>%	Height<br>%
1	13.980	60.575	73.737	49.44	54.33
2	16.950	61.938	61.976	50.56	45.67

Supplementary Figure 274. HPLC chromatogram of racemic 7f

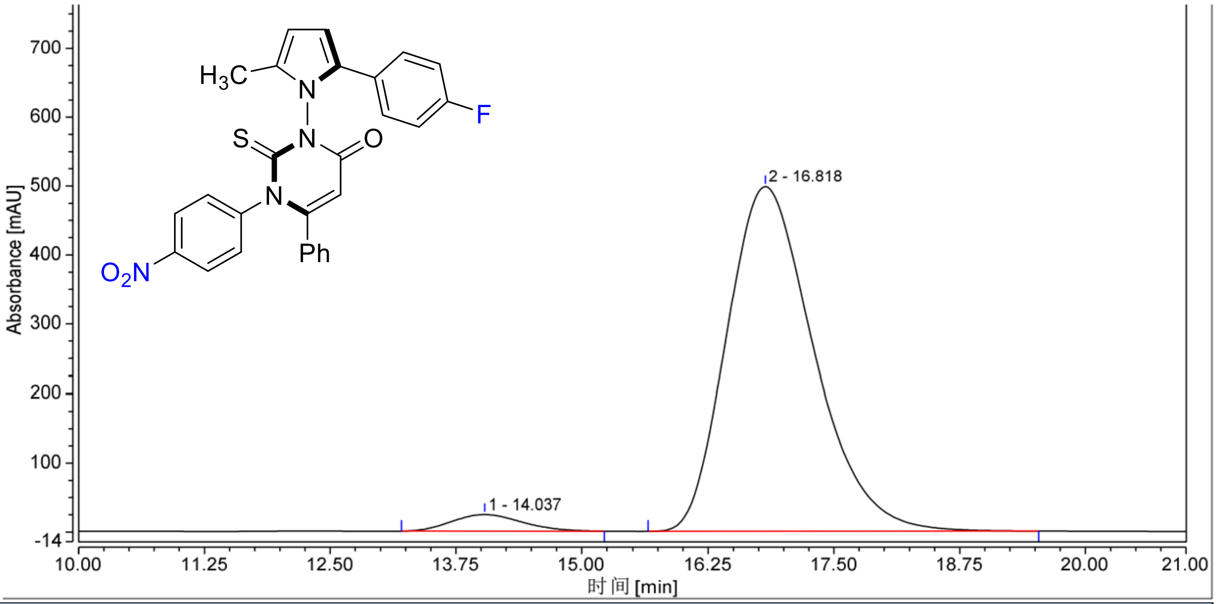

Result					
Peak	RT<br>min	Area<br>mAU*min	Height<br>mAU	Area<br>%	Height<br>%
1	14.037	19.499	24.000	3.76	4.60
2	16.818	498.855	497.969	96.24	95.40

Supplementary Figure 275. HPLC chromatogram of enantiomerically enriched 7f

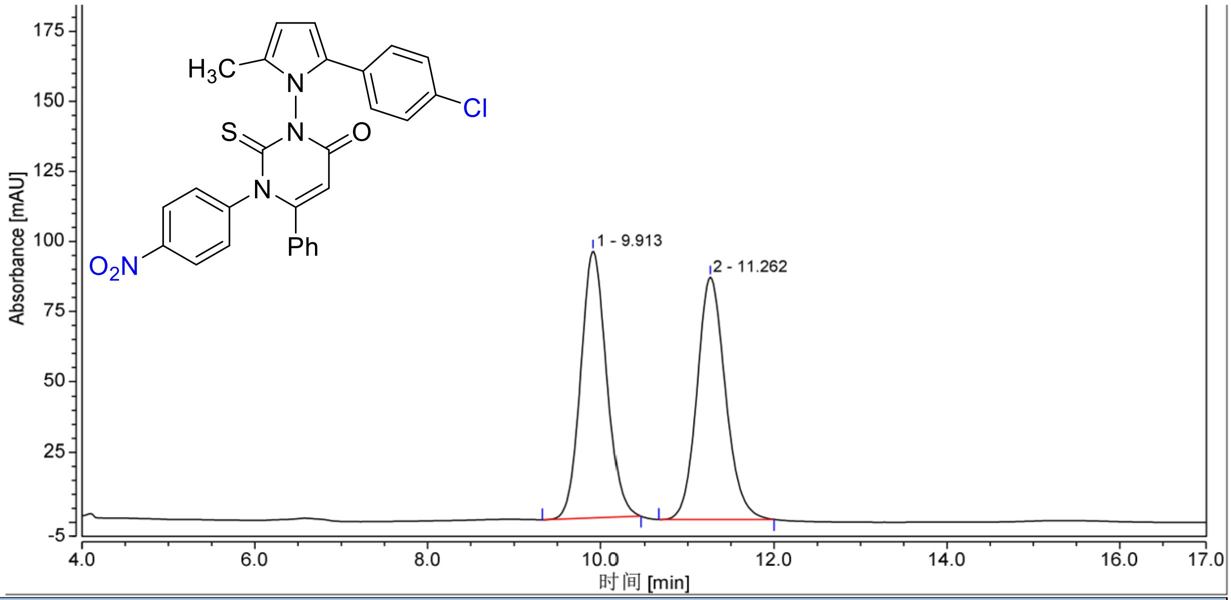

Result

Peak	RT<br>min	Area<br>mAU*min	Height<br>mAU	Area<br>%	Height<br>%
1	9.913	32.683	95.006	49.78	52.42
2	11.262	32.971	86.240	50.22	47.58

Supplementary Figure 276. HPLC chromatogram of racemic 7g

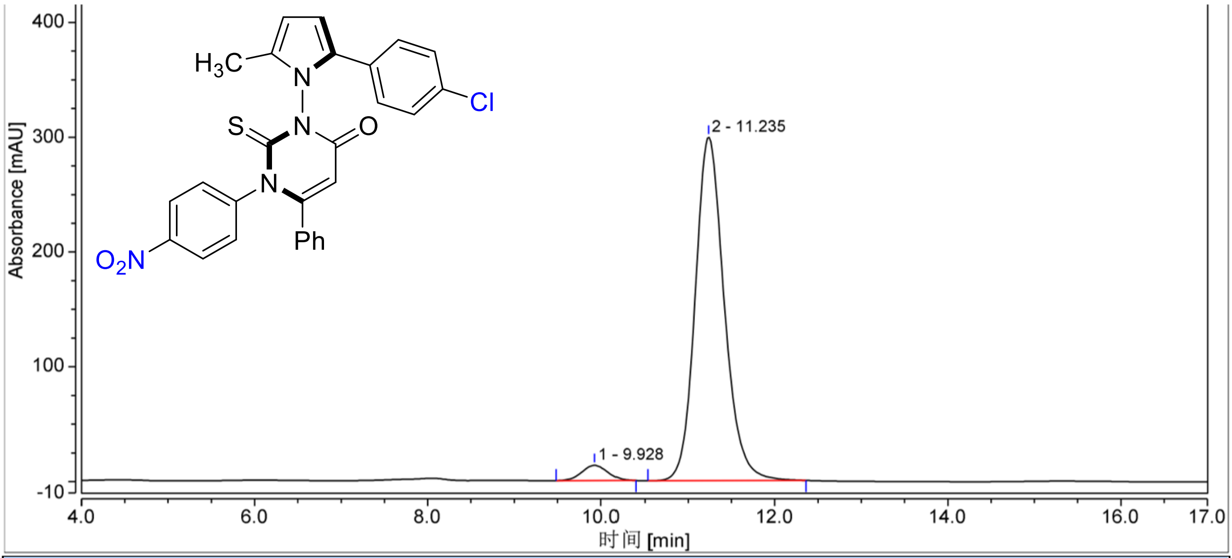

Result

Peak	RT<br>min	Area<br>mAU*min	Height<br>mAU	Area<br>%	Height<br>%
1	9.928	4.589	13.319	3.80	4.26
2	11.235	116.297	299.385	96.20	95.74

Supplementary Figure 277. HPLC chromatogram of enantiomerically enriched 7g

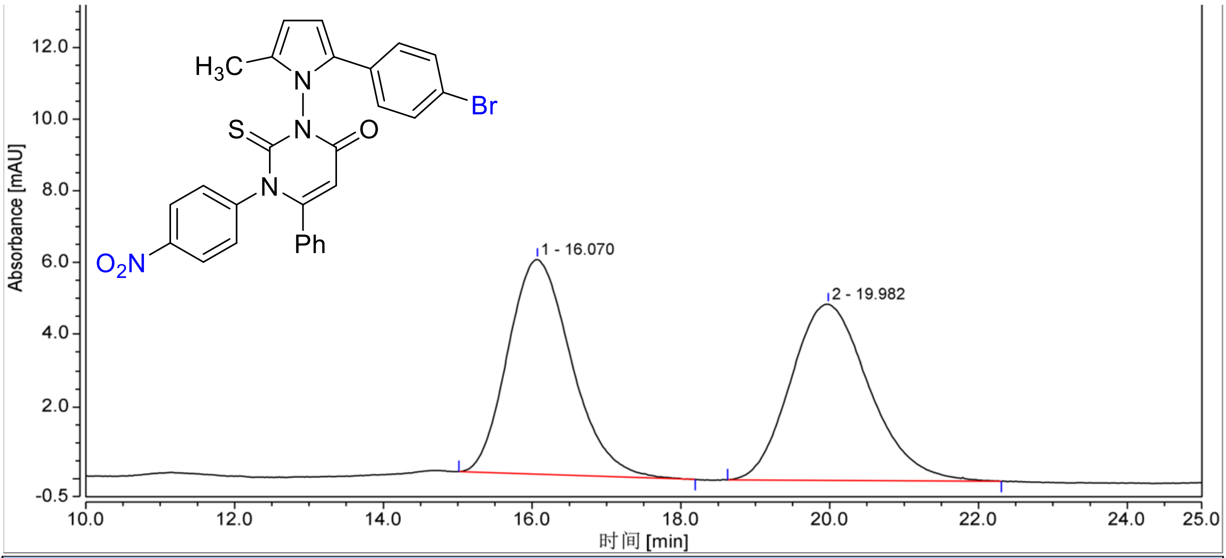

Result

Peak	RT<br>min	Area<br>mAU*min	Height<br>mAU	Area<br>%	Height<br>%
1	16.070	5.878	5.955	49.57	54.95
2	19.982	5.979	4.882	50.43	45.05

Supplementary Figure 278. HPLC chromatogram of racemic 7h

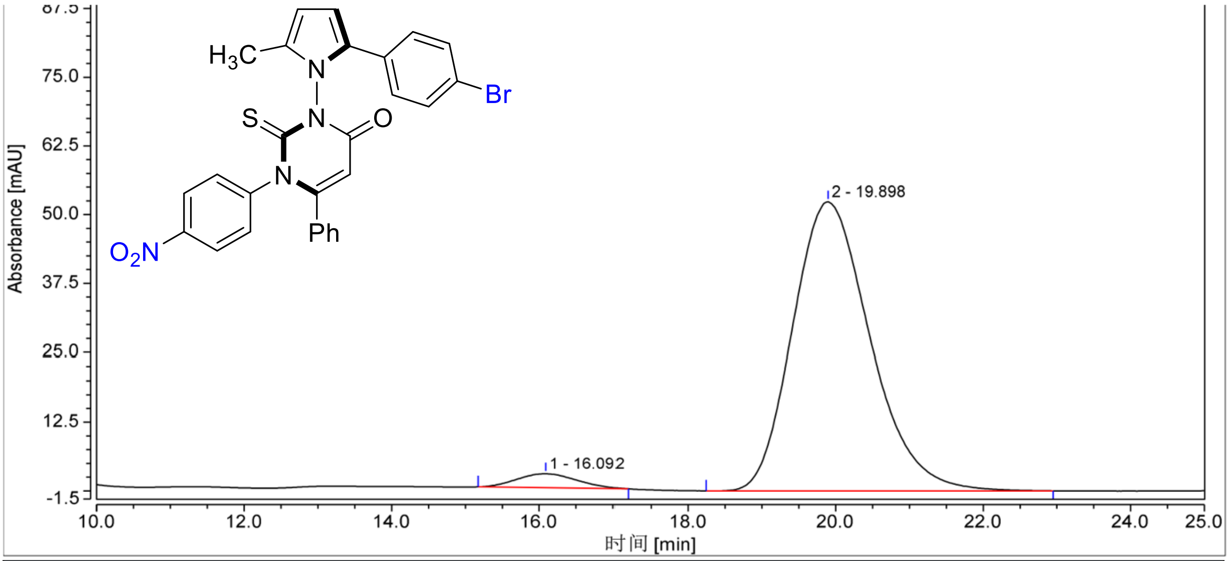

Result

Peak	RT<br>min	Area<br>mAU*min	Height<br>mAU	Area<br>%	Height<br>%
1	16.092	2.390	2.553	3.62	4.65
2	19.898	63.668	52.327	96.38	95.35

Supplementary Figure 279. HPLC chromatogram of enantiomerically enriched 7h

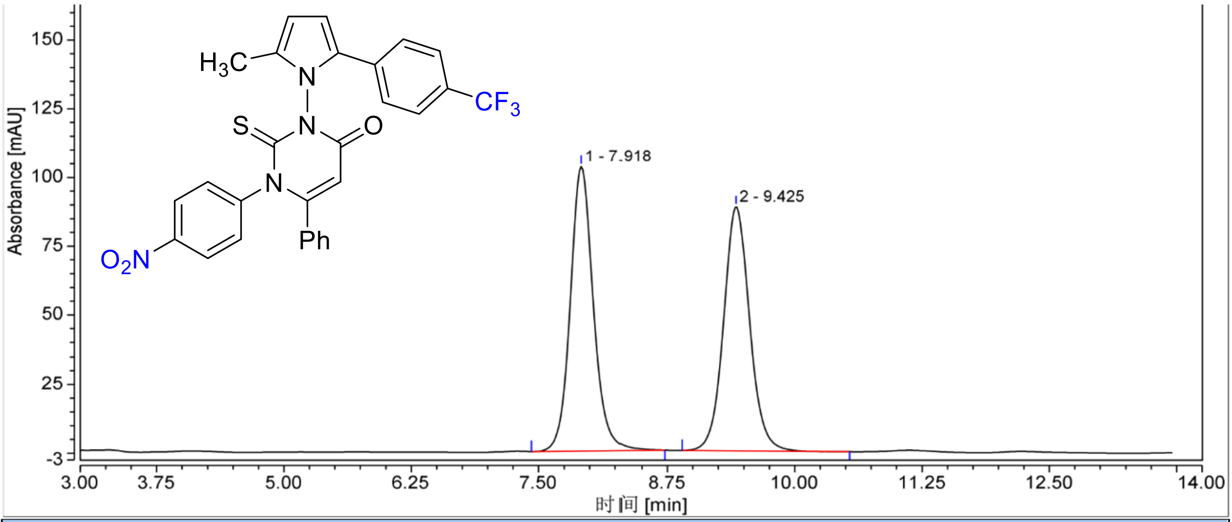

Result

Peak	RT min	Area mAU*min	Height mAU	Area %	Height %
1	7.918	27.134	103.242	50.94	53.84
2	9.425	26.130	88.521	49.06	46.16

Supplementary Figure 280. HPLC chromatogram of racemic 7i

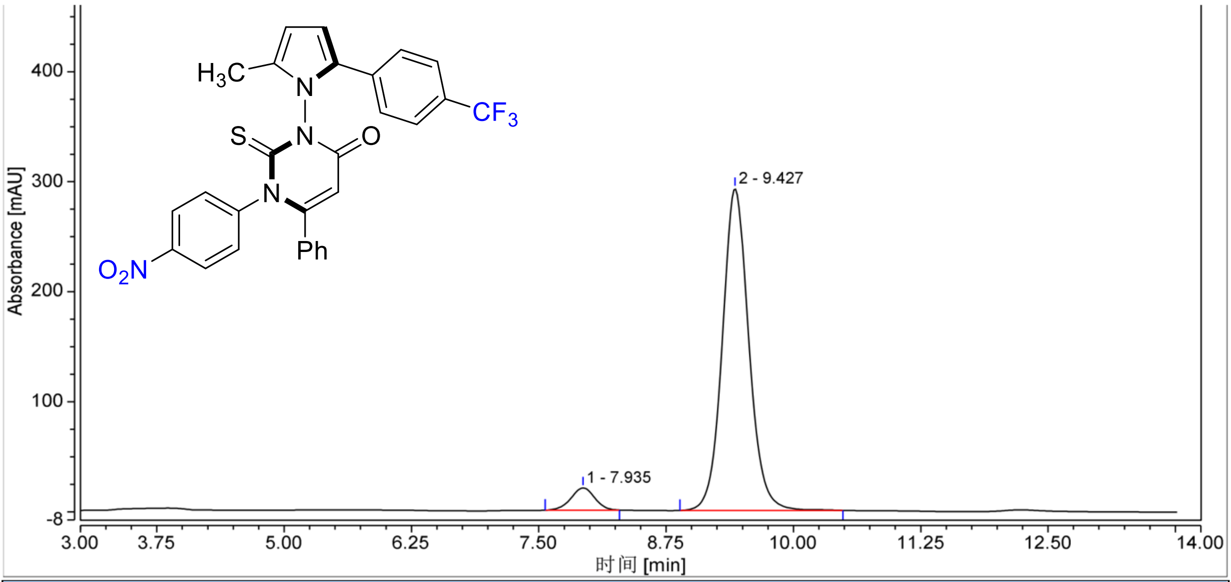

Result

Peak	RT min	Area mAU*min	Height mAU	Area %	Height %
1	7.935	5.477	20.227	5.87	6.47
2	9.427	87.801	292.624	94.13	93.53

Supplementary Figure 281. HPLC chromatogram of enantiomerically enriched 7i

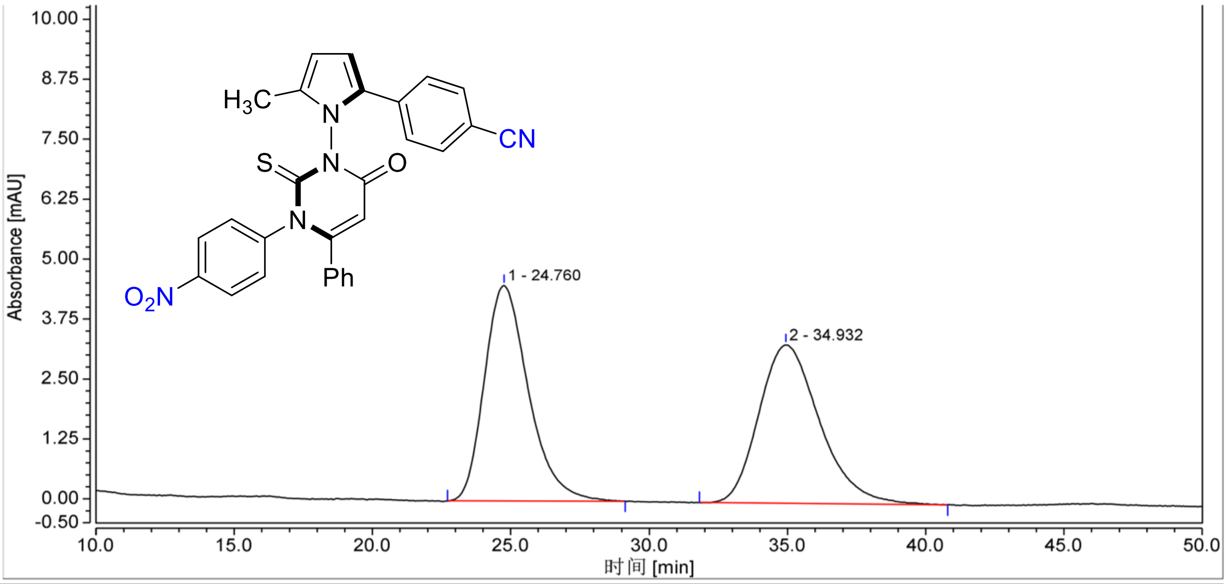

Result

Peak	RT<br>min	Area<br>mAU*min	Height<br>mAU	Area<br>%	Height<br>%
1	24.760	8.297	4.493	49.66	57.63
2	34.932	8.409	3.303	50.34	42.37

Supplementary Figure 282. HPLC chromatogram of racemic 7j

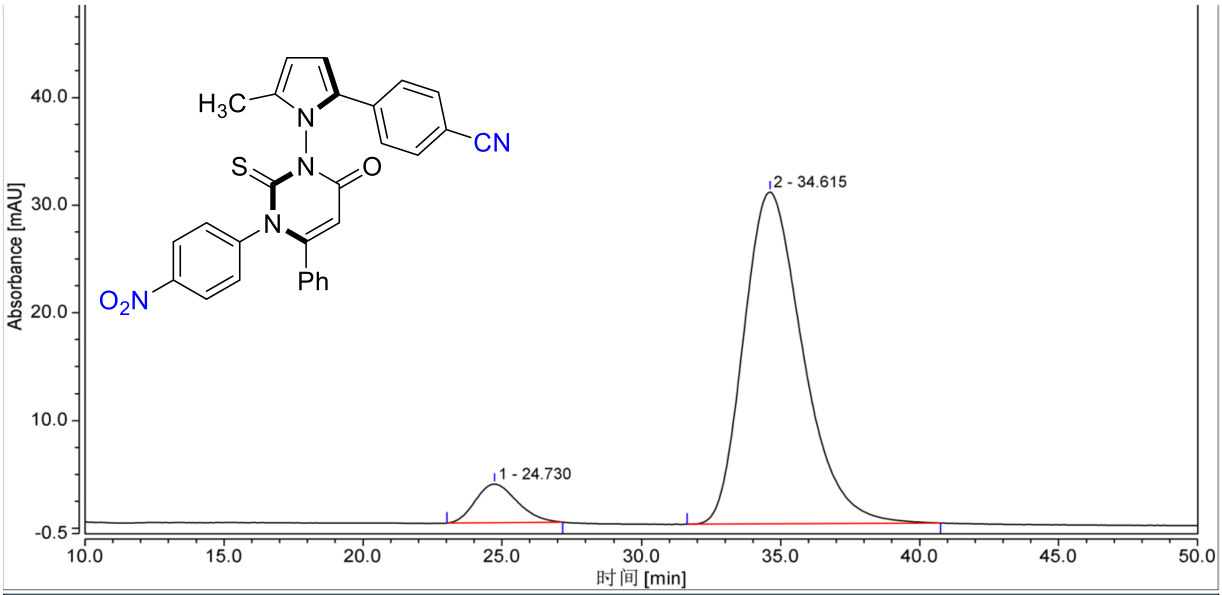

Result

Peak	RT<br>min	Area<br>mAU*min	Height<br>mAU	Area<br>%	Height<br>%
1	24.730	6.248	3.590	7.59	10.43
2	34.615	76.113	30.811	92.41	89.57

Supplementary Figure 283. HPLC chromatogram of enantiomerically enriched 7j

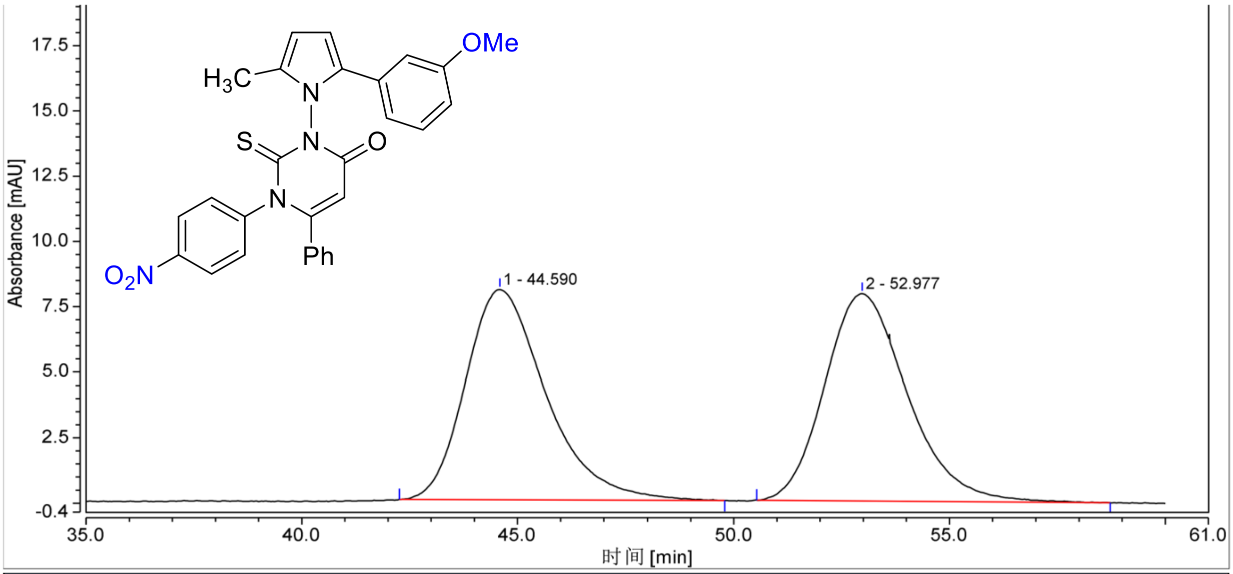

Result

Peak	RT<br>min	Area<br>mAU*min	Height<br>mAU	Area<br>%	Height<br>%
1	44.590	17.364	8.028	49.65	50.33
2	52.977	17.610	7.922	50.35	49.67

Supplementary Figure 284. HPLC chromatogram of racemic 7k

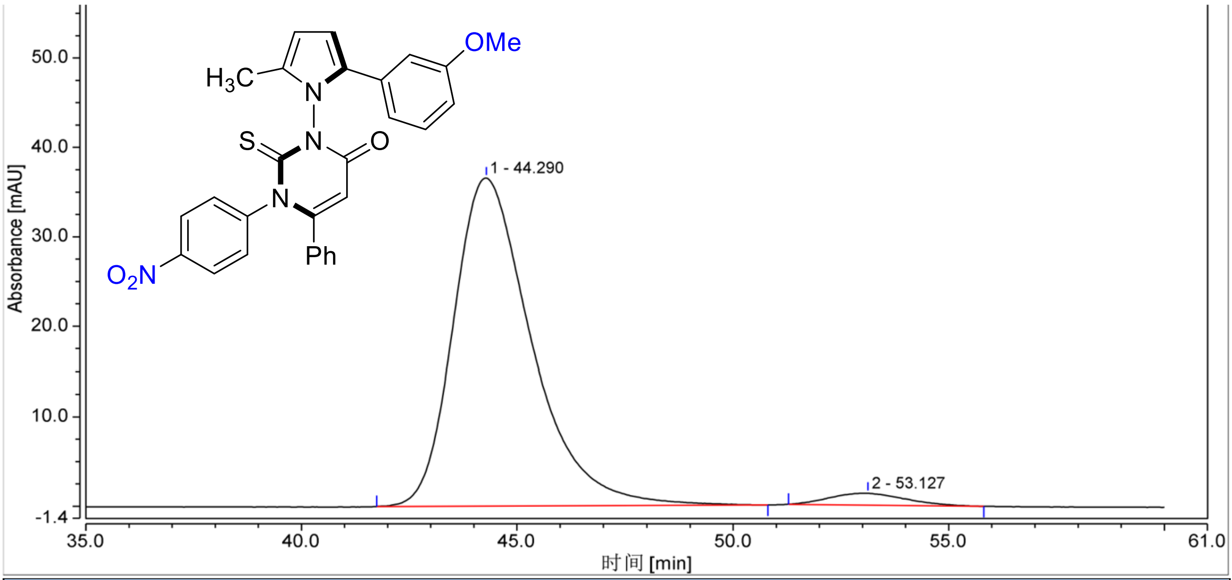

Result

Peak	RT<br>min	Area<br>mAU*min	Height<br>mAU	Area<br>%	Height<br>%
1	44.290	76.857	36.561	96.67	96.51
2	53.127	2.649	1.320	3.33	3.49

Supplementary Figure 285. HPLC chromatogram of enantiomerically enriched 7k

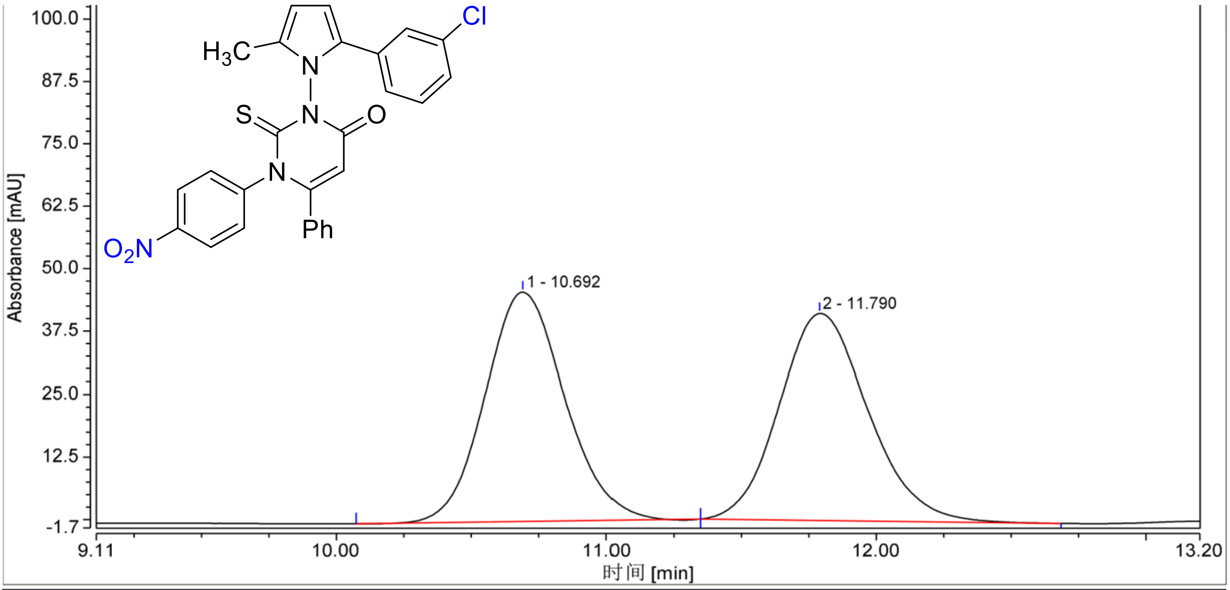

Result					
Peak	RT<br>min	Area<br>mAU*min	Height<br>mAU	Area<br>%	Height<br>%
1	10.692	15.643	45.642	49.75	52.58
2	11.790	15.798	41.166	50.25	47.42

Supplementary Figure 286. HPLC chromatogram of racemic 7l

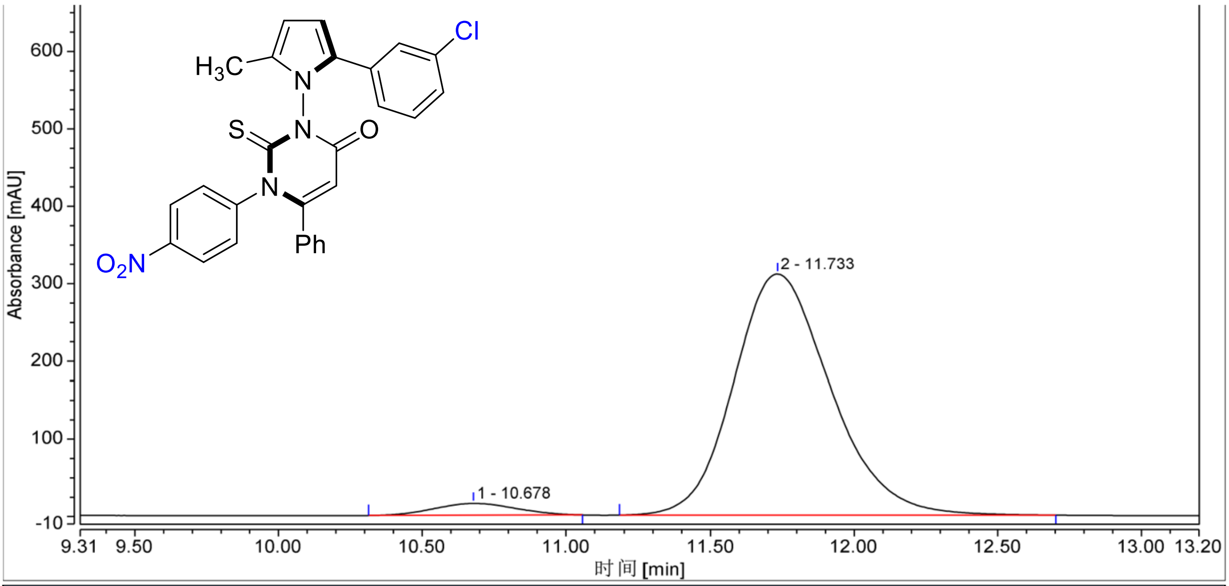

Result					
Peak	RT<br>min	Area<br>mAU*min	Height<br>mAU	Area<br>%	Height<br>%
1	10.678	5.004	15.242	3.95	4.68
2	11.733	121.573	310.605	96.05	95.32

Supplementary Figure 287. HPLC chromatogram of enantiomerically enriched 7l

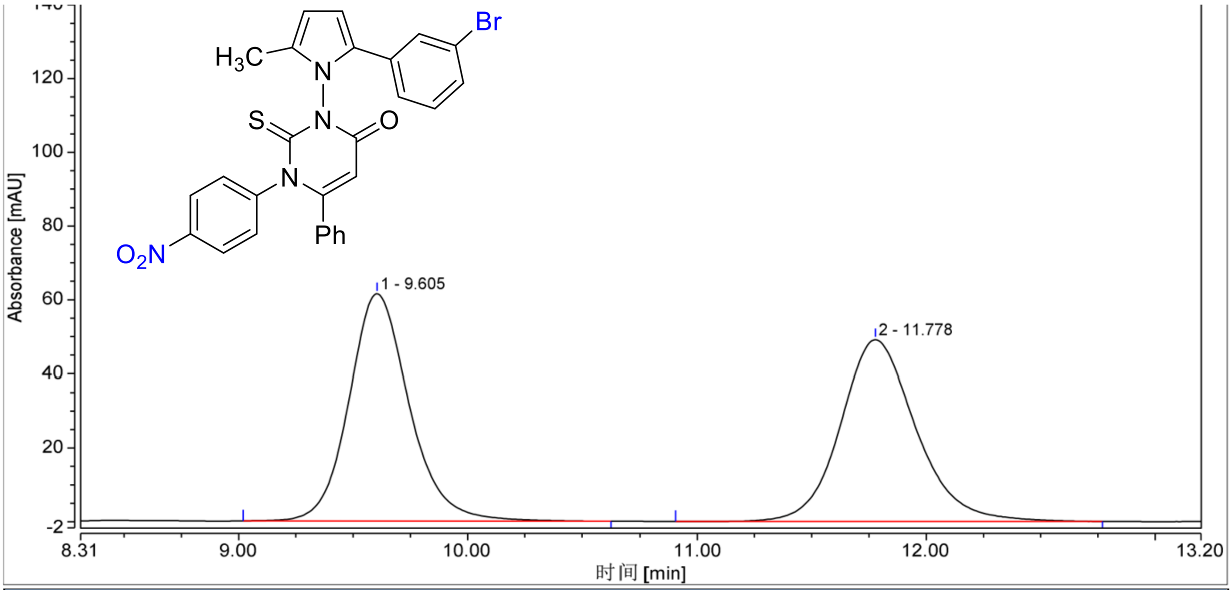

Result

Peak	RT min	Area mAU*min	Height mAU	Area %	Height %
1	9.605	18.807	61.423	50.02	55.58
2	11.778	18.793	49.081	49.98	44.42

Supplementary Figure 288. HPLC chromatogram of racemic 7m

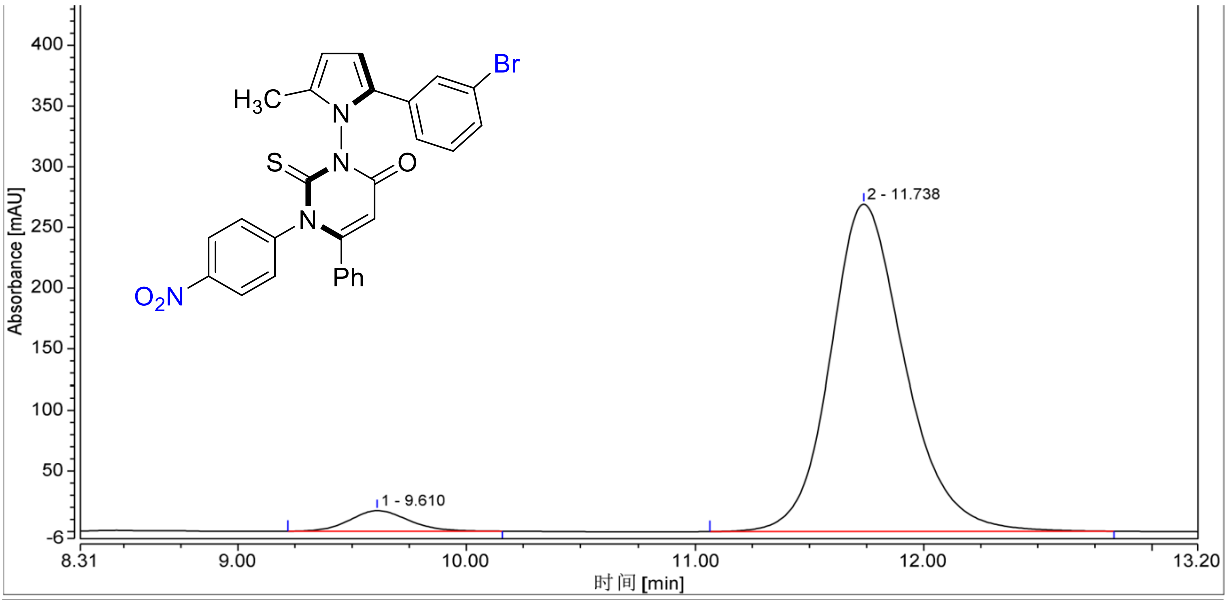

Result

Peak	RT min	Area mAU*min	Height mAU	Area %	Height %
1	9.610	5.095	16.973	4.75	5.94
2	11.738	102.167	268.978	95.25	94.06

Supplementary Figure 289. HPLC chromatogram of enantiomerically enriched 7m

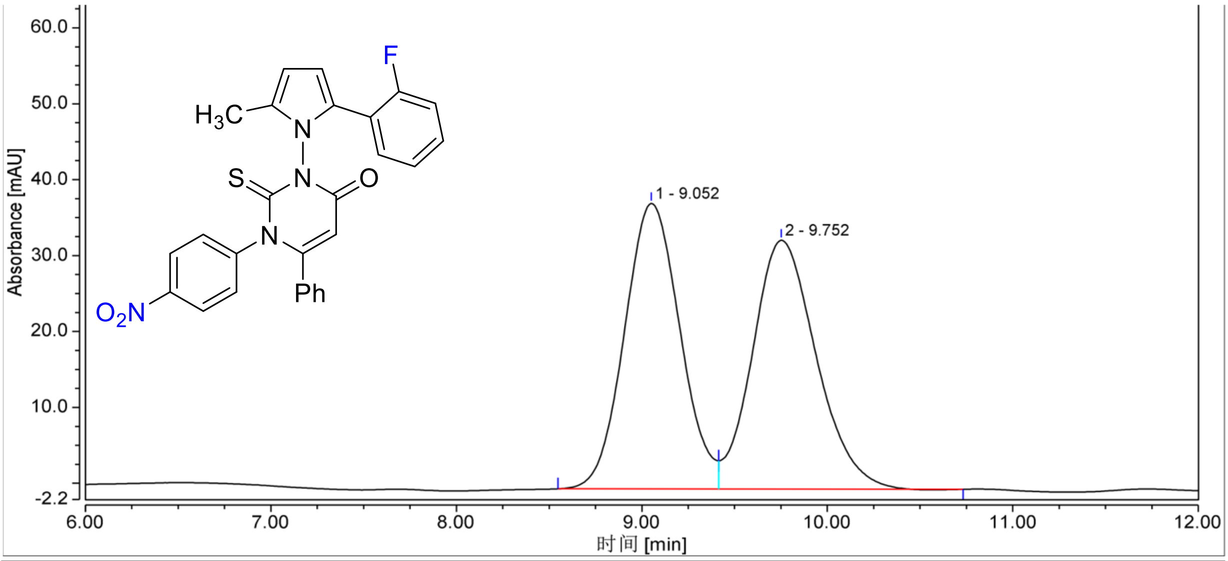

Result

Peak	RT min	Area mAU*min	Height mAU	Area %	Height %
1	9.052	13.043	37.603	49.99	53.42
2	9.752	13.050	32.785	50.01	46.58

Supplementary Figure 290. HPLC chromatogram of racemic 7n

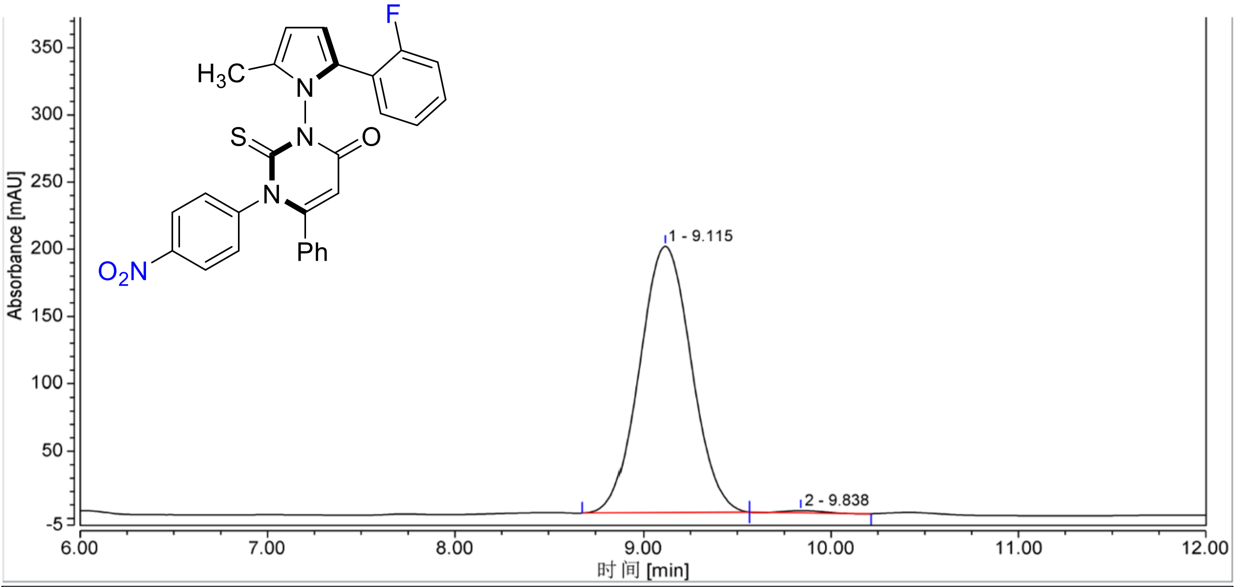

Result

Peak	RT min	Area mAU*min	Height mAU	Area %	Height %
1	9.115	63.139	197.960	99.46	99.20
2	9.838	0.343	1.600	0.54	0.80

Supplementary Figure 291. HPLC chromatogram of enantiomerically enriched 7n

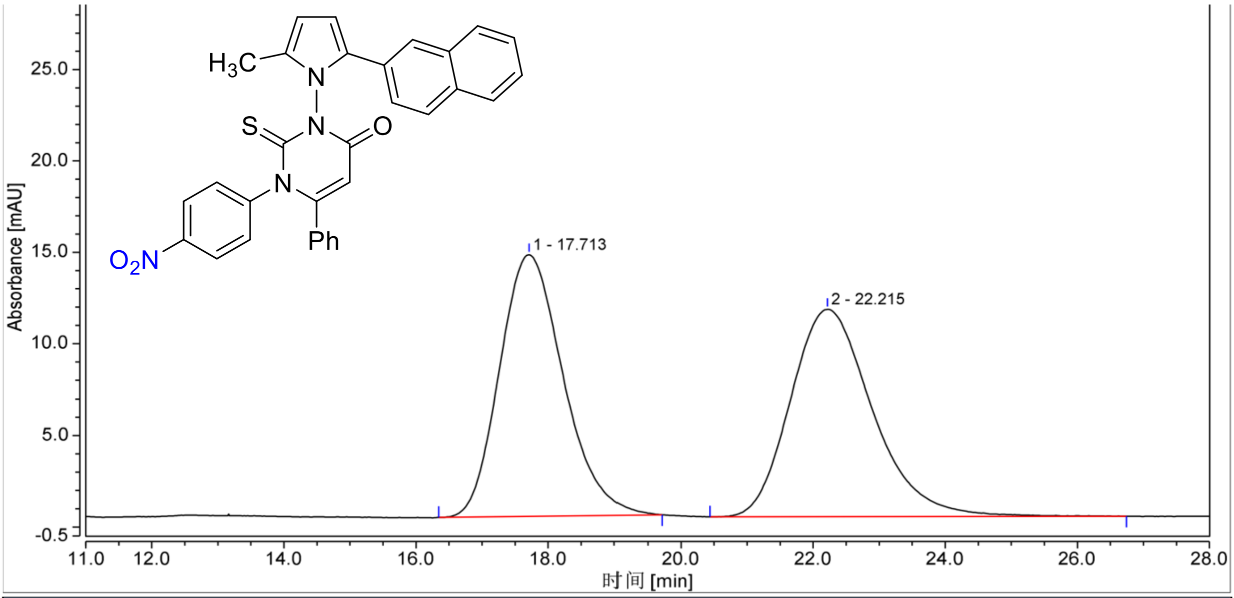

Result

Peak	RT<br>min	Area<br>mAU*min	Height<br>mAU	Area<br>%	Height<br>%
1	17.713	15.954	14.291	49.50	55.79
2	22.215	16.278	11.325	50.50	44.21

Supplementary Figure 292. HPLC chromatogram of racemic 7o

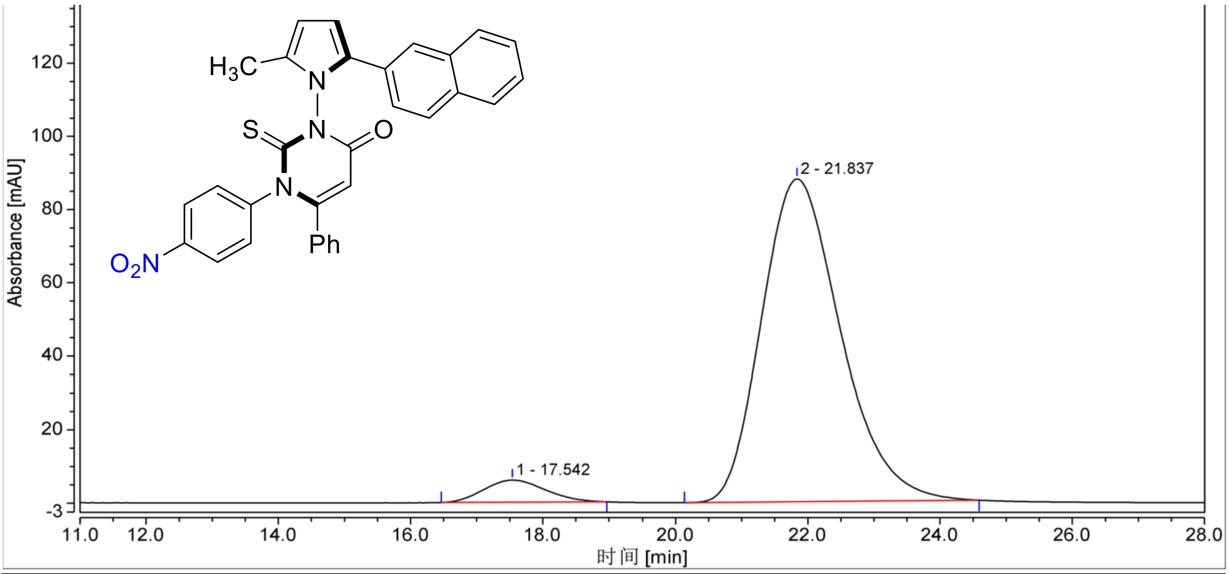

Result

Peak	RT<br>min	Area<br>mAU*min	Height<br>mAU	Area<br>%	Height<br>%
1	17.542	6.506	6.044	5.08	6.43
2	21.837	121.680	88.018	94.92	93.57

Supplementary Figure 293. HPLC chromatogram of enantiomerically enriched 7o

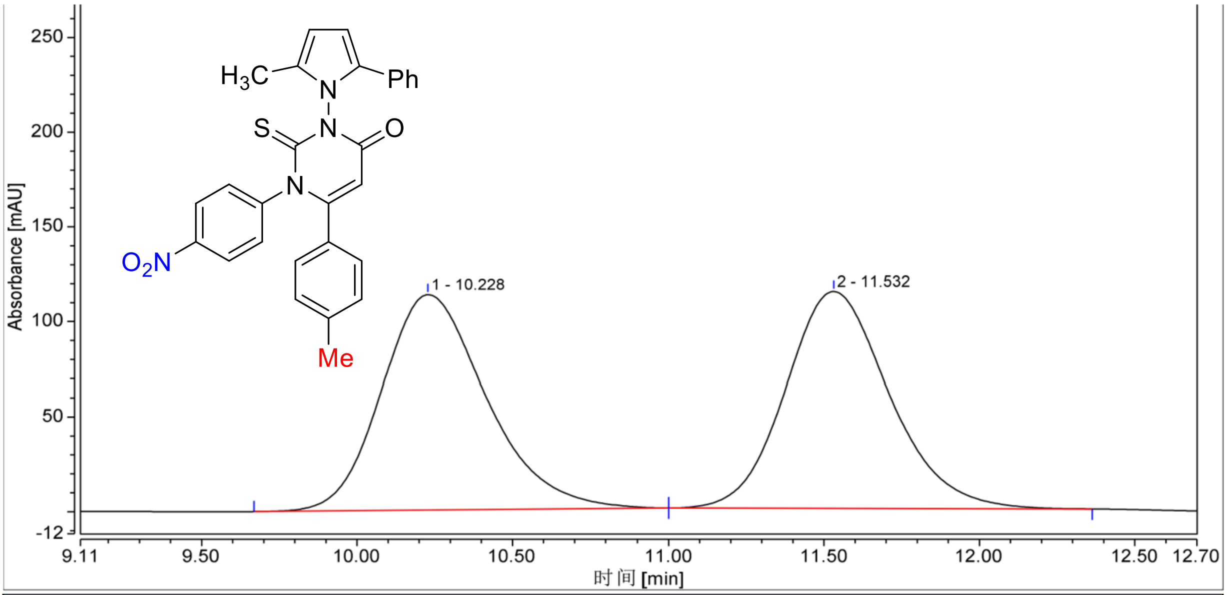

Result

Peak	RT<br>min	Area<br>mAU*min	Height<br>mAU	Area<br>%	Height<br>%
1	10.228	44.401	113.312	50.09	49.83
2	11.532	44.246	114.067	49.91	50.17

Supplementary Figure 294. HPLC chromatogram of racemic 7p

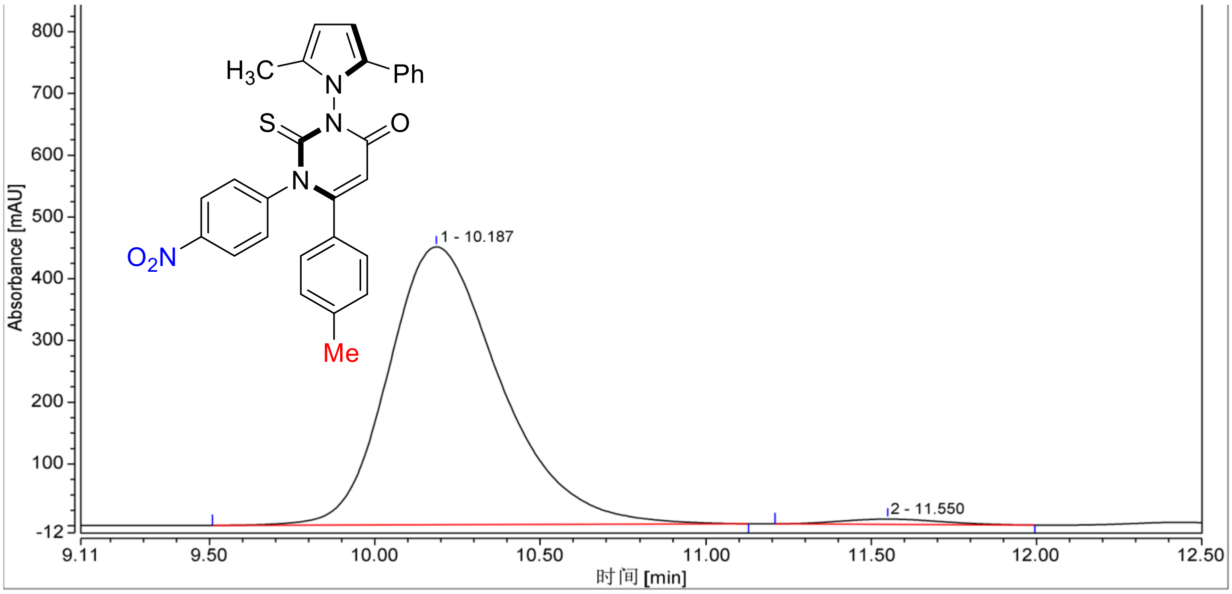

Result

Peak	RT<br>min	Area<br>mAU*min	Height<br>mAU	Area<br>%	Height<br>%
1	10.187	177.613	450.044	98.34	98.15
2	11.550	2.991	8.499	1.66	1.85

Supplementary Figure 295. HPLC chromatogram of enantiomerically enriched 7p

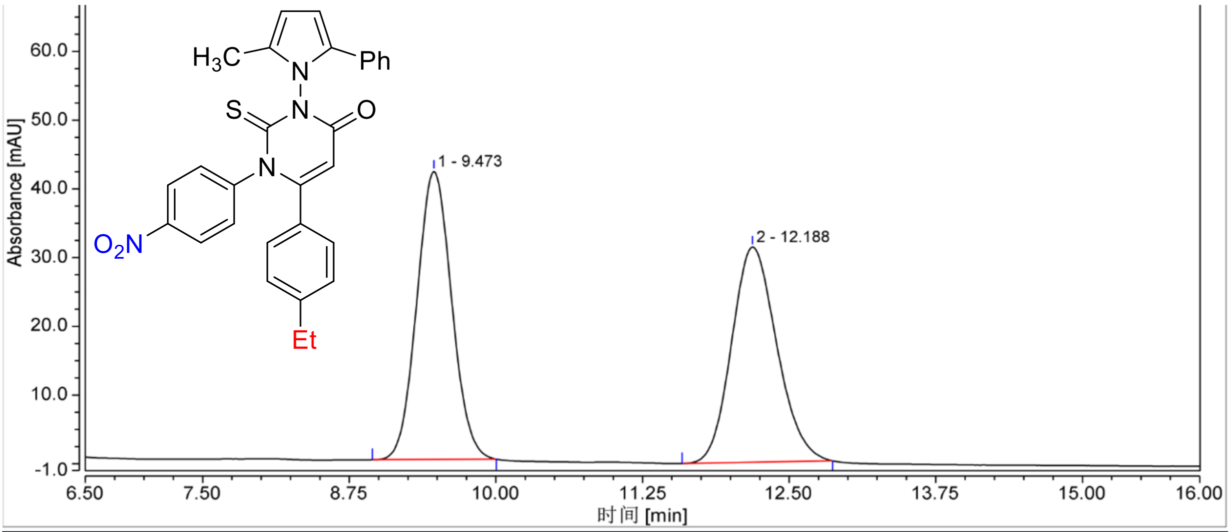

Result

Peak	RT<br>min	Area<br>mAU*min	Height<br>mAU	Area<br>%	Height<br>%
1	9.473	14.420	41.944	50.76	57.24
2	12.188	13.987	31.329	49.24	42.76

Supplementary Figure 296. HPLC chromatogram of racemic 7q

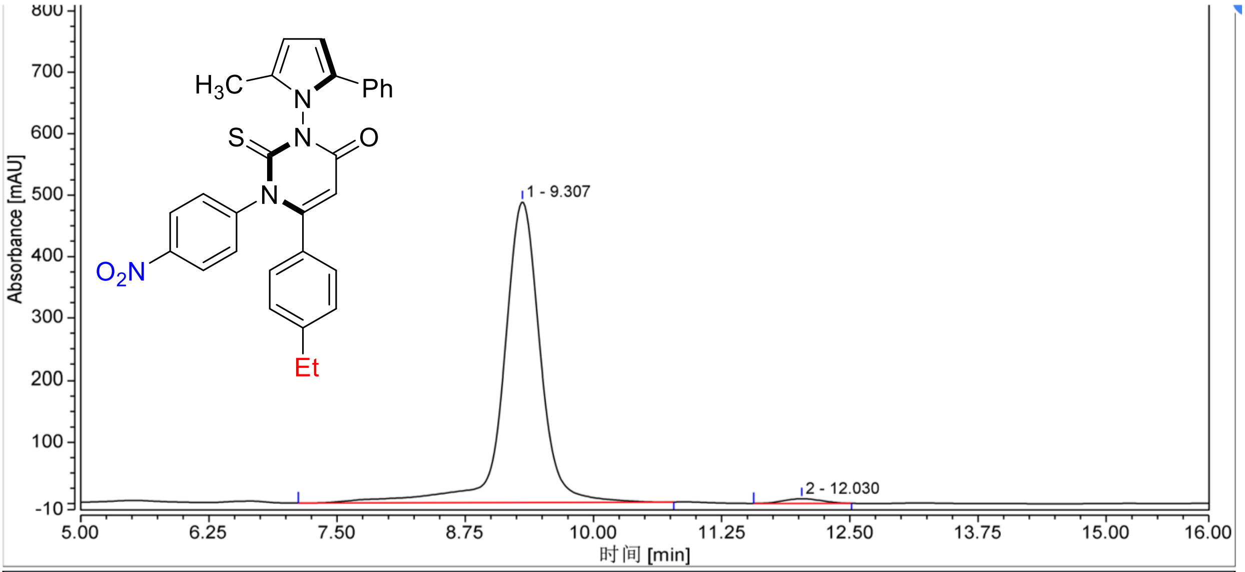

Result

Peak	RT<br>min	Area<br>mAU*min	Height<br>mAU	Area<br>%	Height<br>%
1	9.307	202.744	486.946	98.44	98.47
2	12.030	3.205	7.577	1.56	1.53

Supplementary Figure 297. HPLC chromatogram of enantiomerically enriched 7q

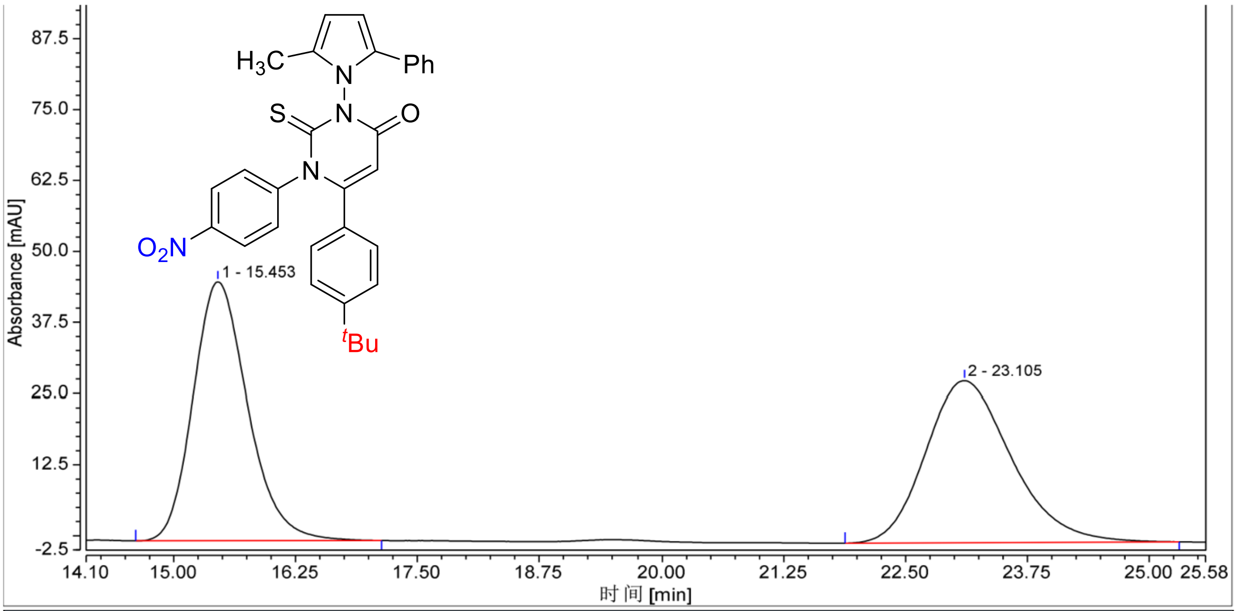

Result

Peak	RT min	Area mAU*min	Height mAU	Area %	Height %
1	15.453	29.607	45.501	50.40	61.55
2	23.105	29.135	28.423	49.60	38.45

Supplementary Figure 298. HPLC chromatogram of racemic 7r

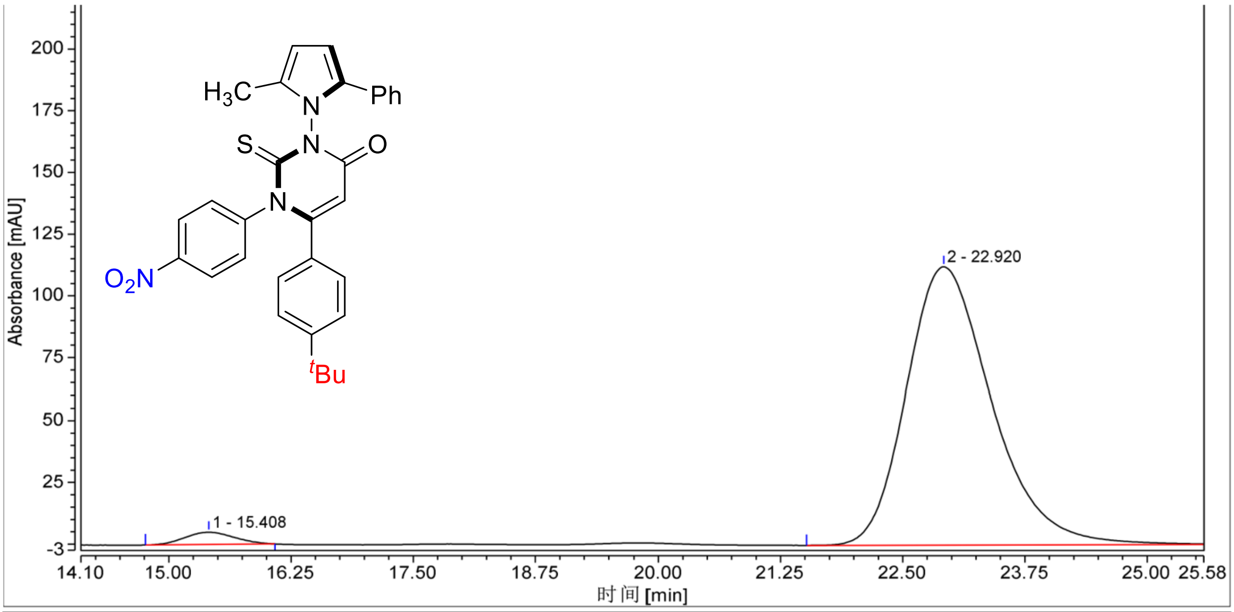

Result

Peak	RT min	Area mAU*min	Height mAU	Area %	Height %
1	15.408	2.921	4.948	2.51	4.23
2	22.920	113.556	112.149	97.49	95.77

Supplementary Figure 299. HPLC chromatogram of enantiomerically enriched 7r

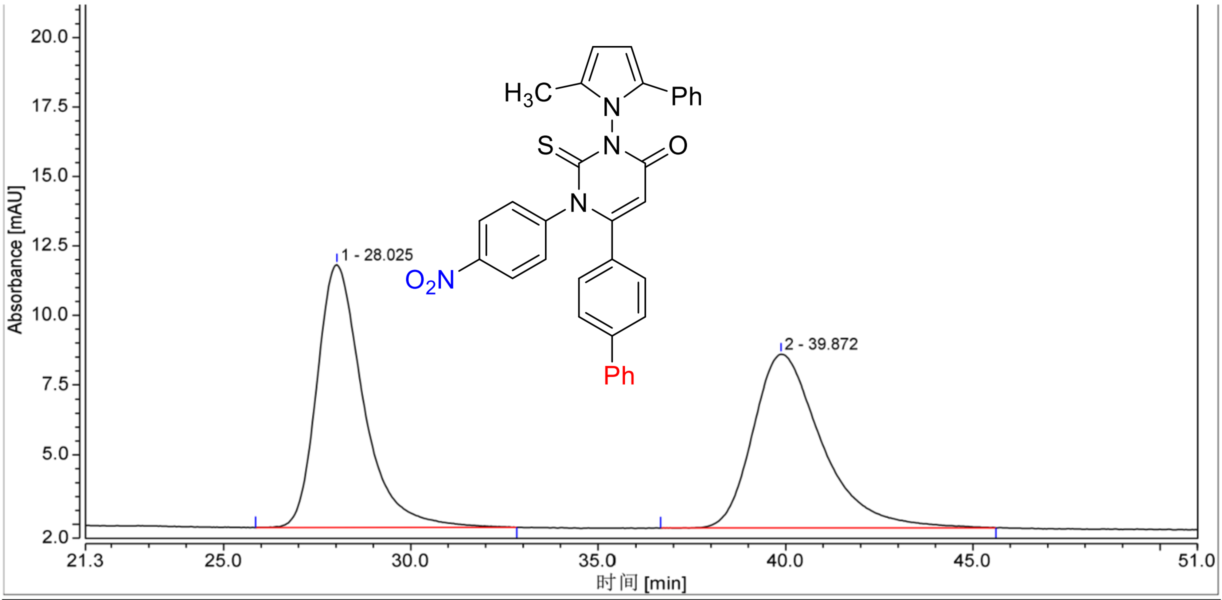

Result

Peak	RT min	Area mAU*min	Height mAU	Area %	Height %
1	28.025	13.547	9.430	50.45	60.22
2	39.872	13.304	6.230	49.55	39.78

Supplementary Figure 300. HPLC chromatogram of racemic 7s

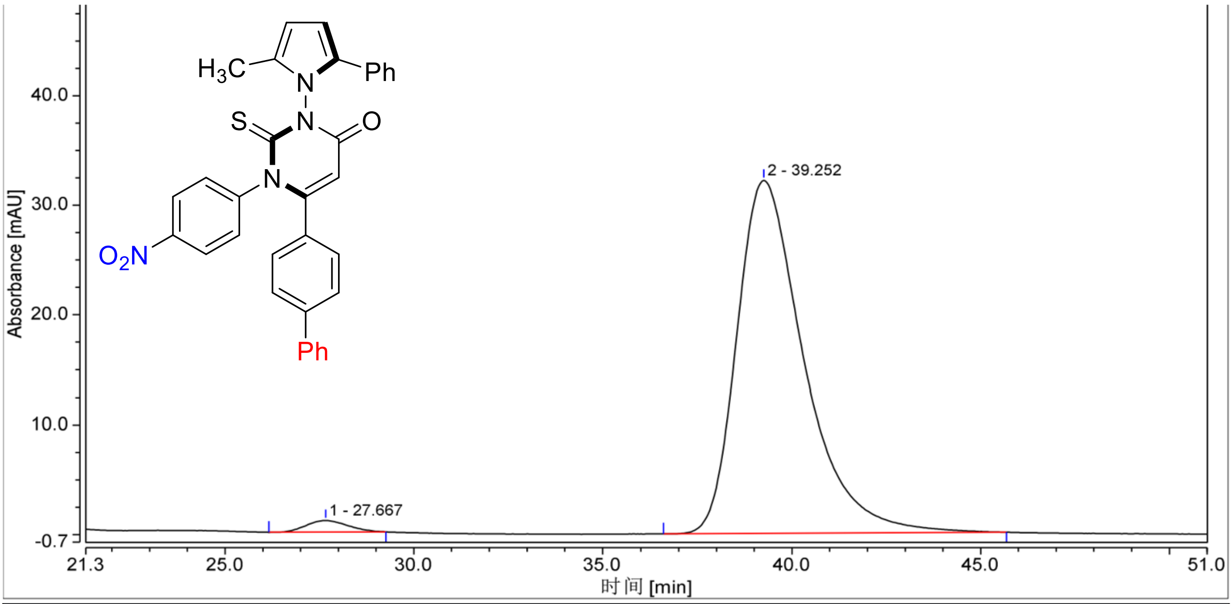

Result

Peak	RT min	Area mAU*min	Height mAU	Area %	Height %
1	27.667	1.364	1.062	2.02	3.19
2	39.252	66.244	32.174	97.98	96.81

Supplementary Figure 301. HPLC chromatogram of enantiomerically enriched 7s

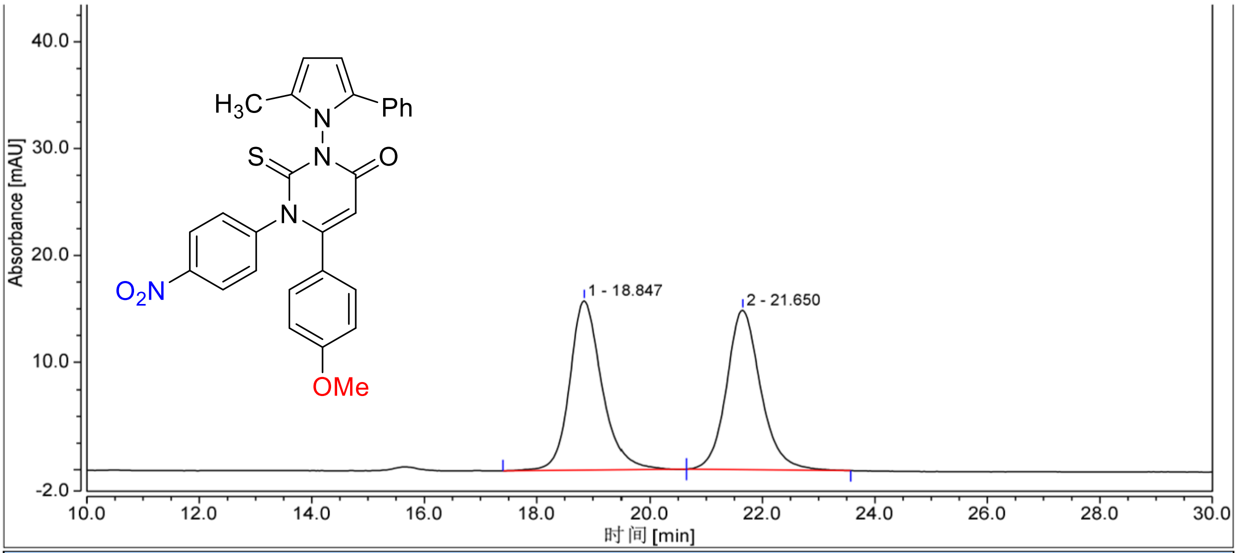

Result

Peak	RT min	Area mAU*min	Height mAU	Area %	Height %
1	18.847	10.470	15.782	50.01	51.50
2	21.650	10.466	14.862	49.99	48.50

Supplementary Figure 302. HPLC chromatogram of racemic 7t

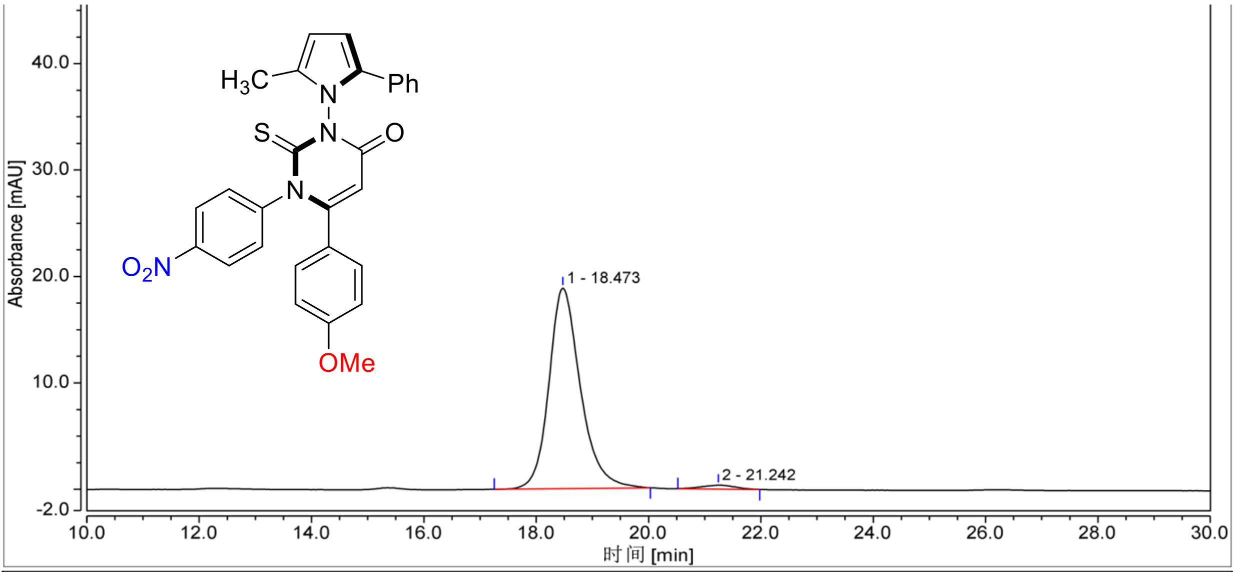

Result

Peak	RT min	Area mAU*min	Height mAU	Area %	Height %
1	18.473	12.252	18.855	98.00	98.09
2	21.242	0.250	0.367	2.00	1.91

Supplementary Figure 303. HPLC chromatogram of enantiomerically enriched 7t

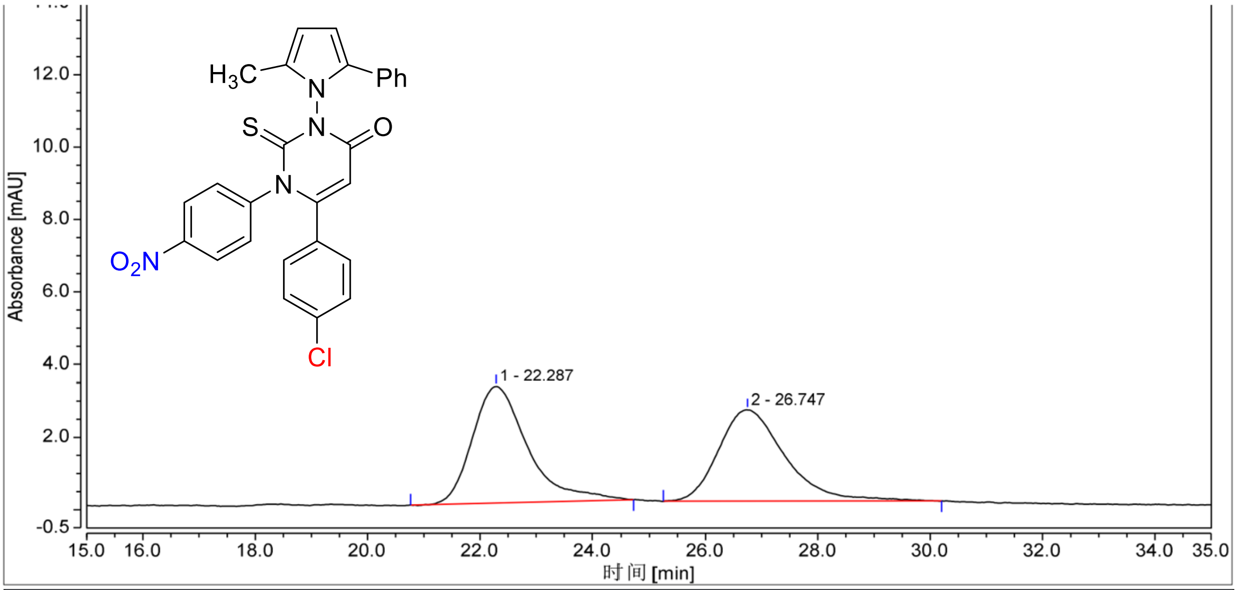

Result

Peak	RT<br>min	Area<br>mAU*min	Height<br>mAU	Area<br>%	Height<br>%
1	22.287	3.656	3.220	51.44	56.03
2	26.747	3.452	2.527	48.56	43.97

Supplementary Figure 304. HPLC chromatogram of racemic 7u

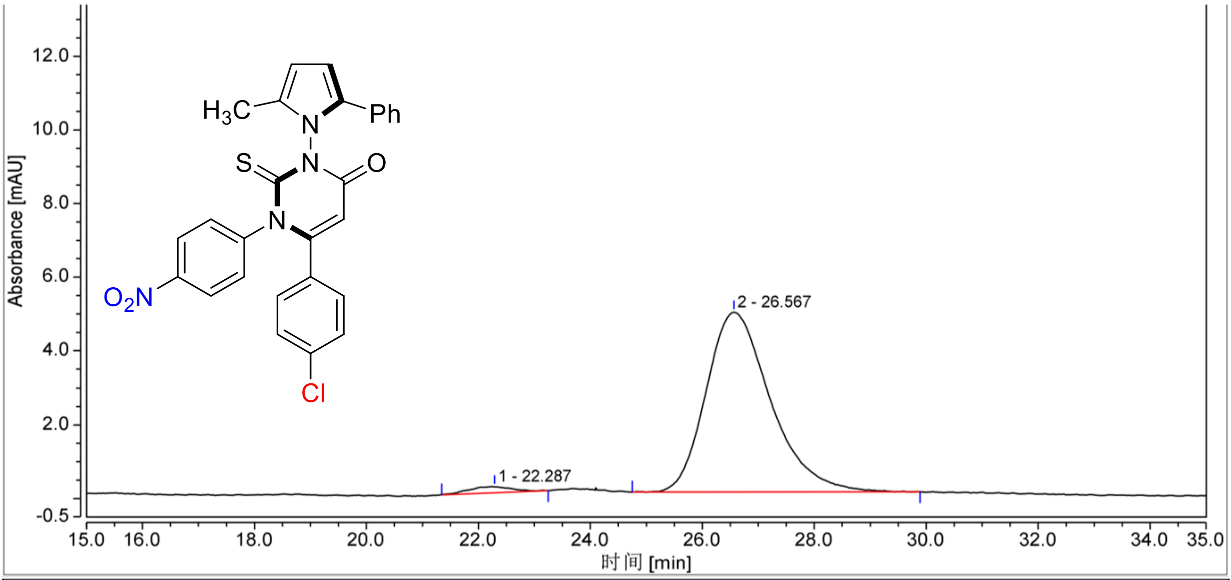

Result

Peak	RT<br>min	Area<br>mAU*min	Height<br>mAU	Area<br>%	Height<br>%
1	22.287	0.148	0.167	2.22	3.31
2	26.567	6.498	4.871	97.78	96.69

Supplementary Figure 305. HPLC chromatogram of enantiomerically enriched 7u

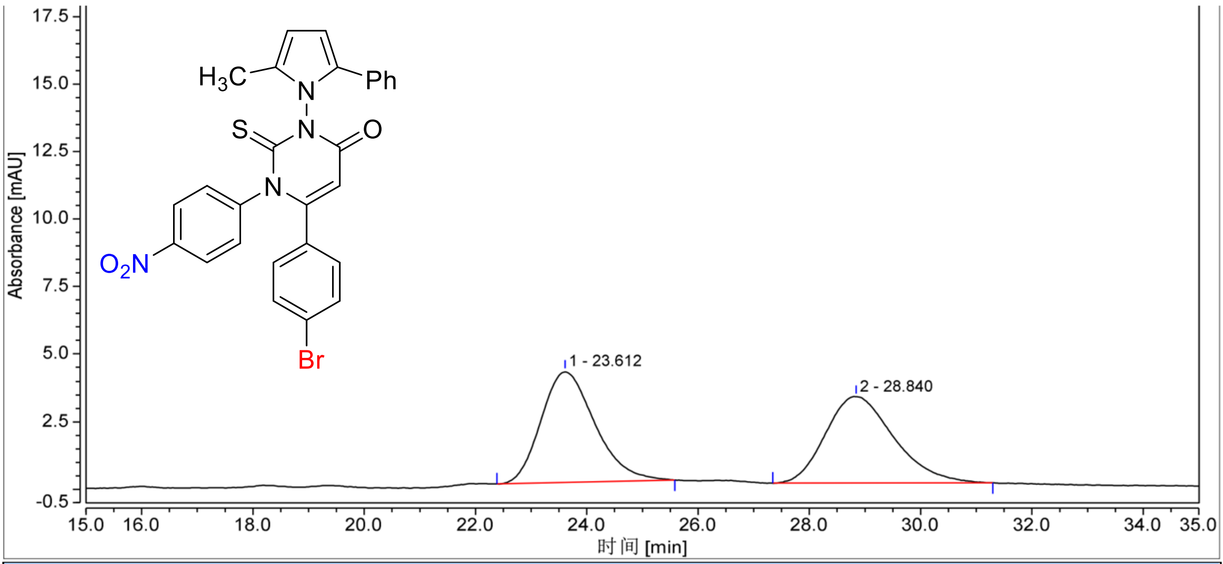

Result

Peak	RT<br>min	Area<br>mAU*min	Height<br>mAU	Area<br>%	Height<br>%
1	23.612	4.605	4.103	50.15	56.05
2	28.840	4.576	3.217	49.85	43.95

Supplementary Figure 306. HPLC chromatogram of racemic 7v

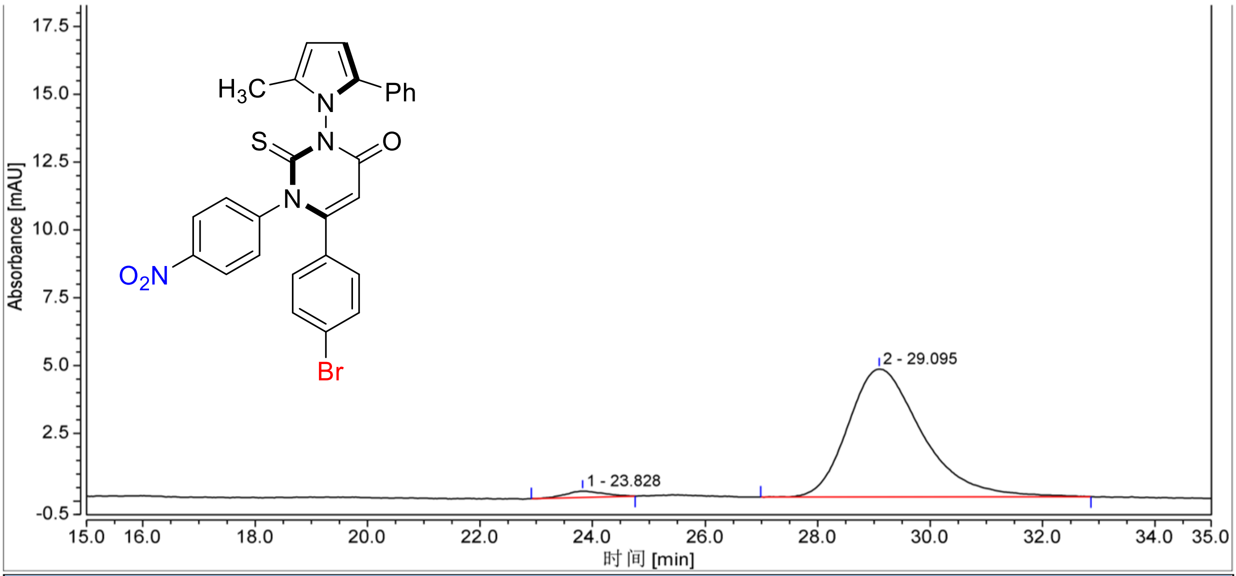

Result

Peak	RT<br>min	Area<br>mAU*min	Height<br>mAU	Area<br>%	Height<br>%
1	23.828	0.179	0.232	2.43	4.68
2	29.095	7.210	4.723	97.57	95.32

Supplementary Figure 307. HPLC chromatogram of enantiomerically enriched 7v

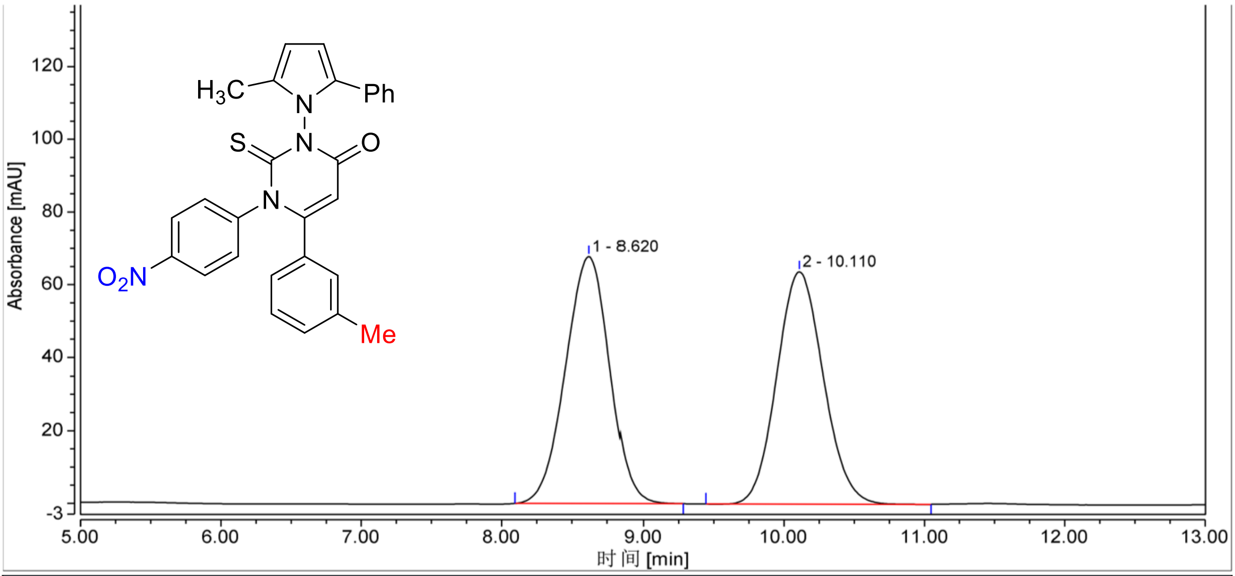

Result

Peak	RT<br>min	Area<br>mAU*min	Height<br>mAU	Area<br>%	Height<br>%
1	8.620	24.519	67.788	50.02	51.53
2	10.110	24.499	63.771	49.98	48.47

Supplementary Figure 308. HPLC chromatogram of racemic 7w

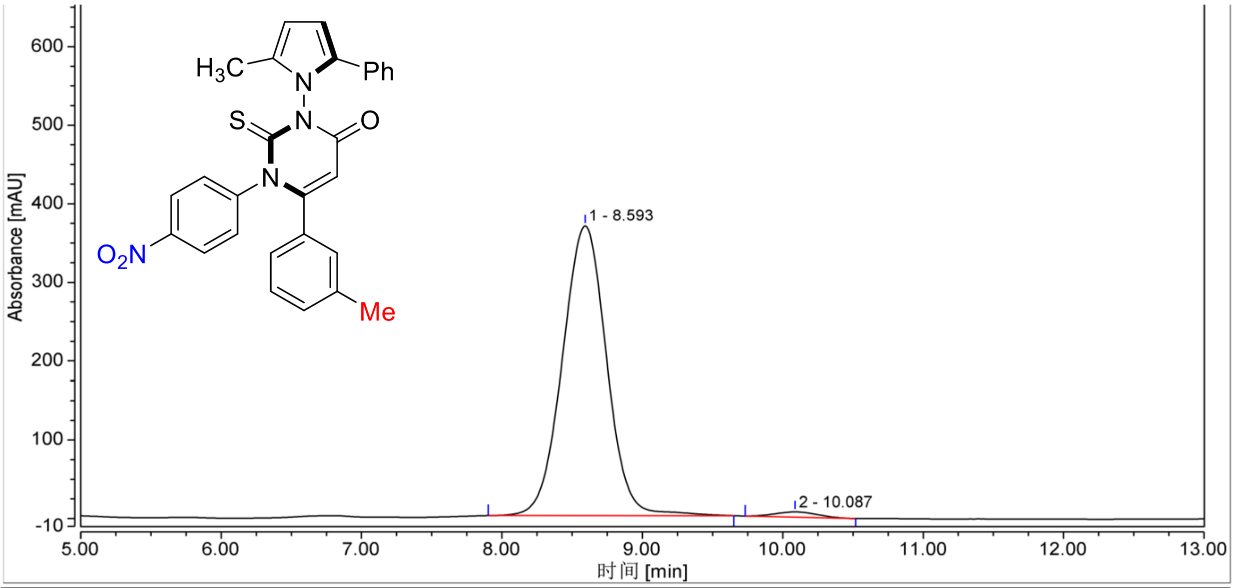

Result

Peak	RT<br>min	Area<br>mAU*min	Height<br>mAU	Area<br>%	Height<br>%
1	8.593	134.208	368.242	98.30	98.23
2	10.087	2.317	6.620	1.70	1.77

Supplementary Figure 309. HPLC chromatogram of enantiomerically enriched 7w

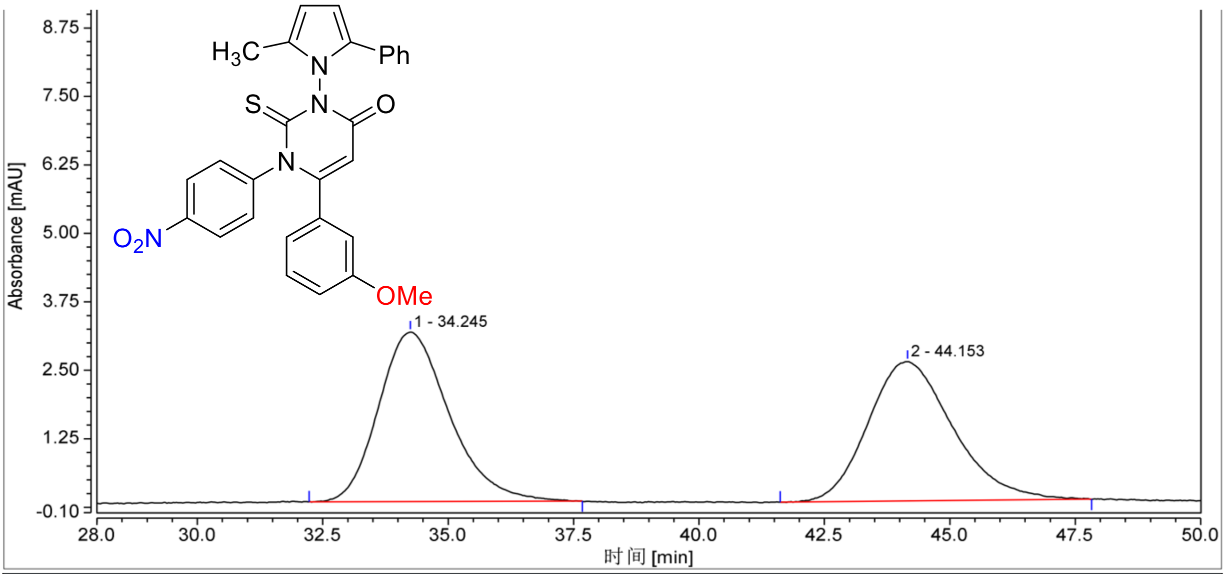

Result

Peak	RT<br>min	Area<br>mAU*min	Height<br>mAU	Area<br>%	Height<br>%
1	34.245	5.053	3.098	50.19	54.85
2	44.153	5.013	2.550	49.81	45.15

Supplementary Figure 310. HPLC chromatogram of racemic 7x

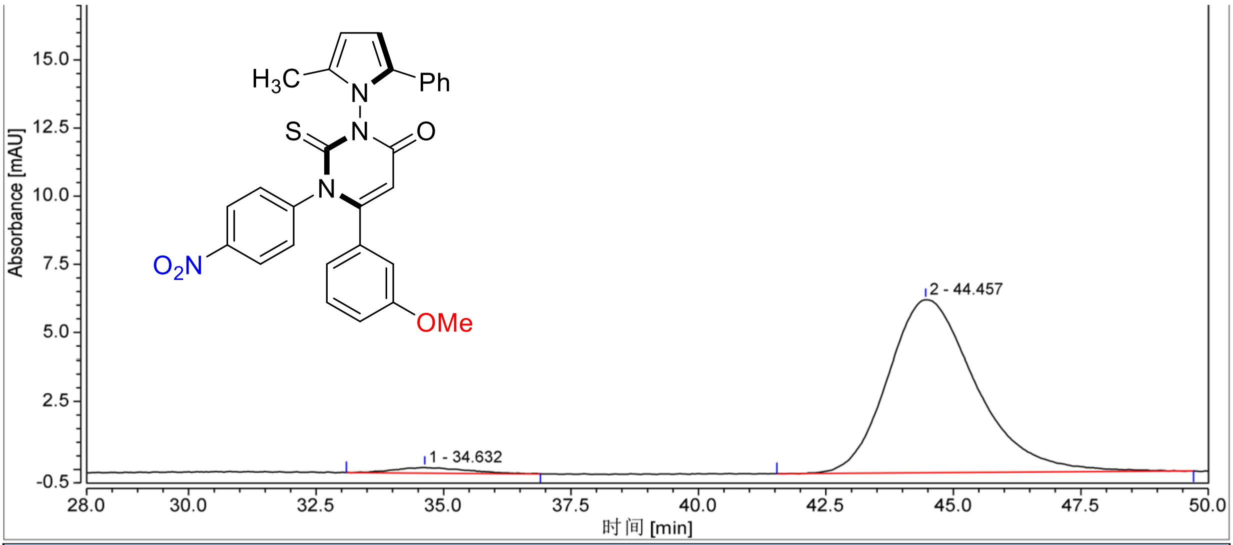

Result

Peak	RT<br>min	Area<br>mAU*min	Height<br>mAU	Area<br>%	Height<br>%
1	34.632	0.329	0.211	2.53	3.23
2	44.457	12.680	6.320	97.47	96.77

Supplementary Figure 311. HPLC chromatogram of enantiomerically enriched 7x

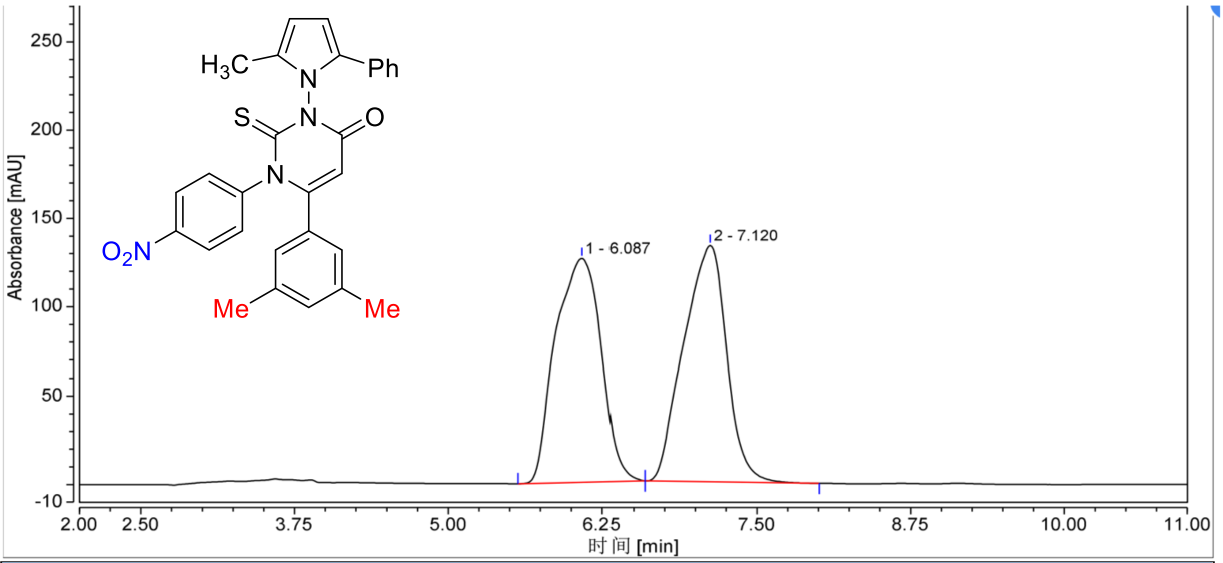

Result					
Peak	RT min	Area mAU*min	Height mAU	Area %	Height %
1	6.087	53.867	126.050	49.97	48.65
2	7.120	53.923	133.041	50.03	51.35

Supplementary Figure 312. HPLC chromatogram of racemic 7y

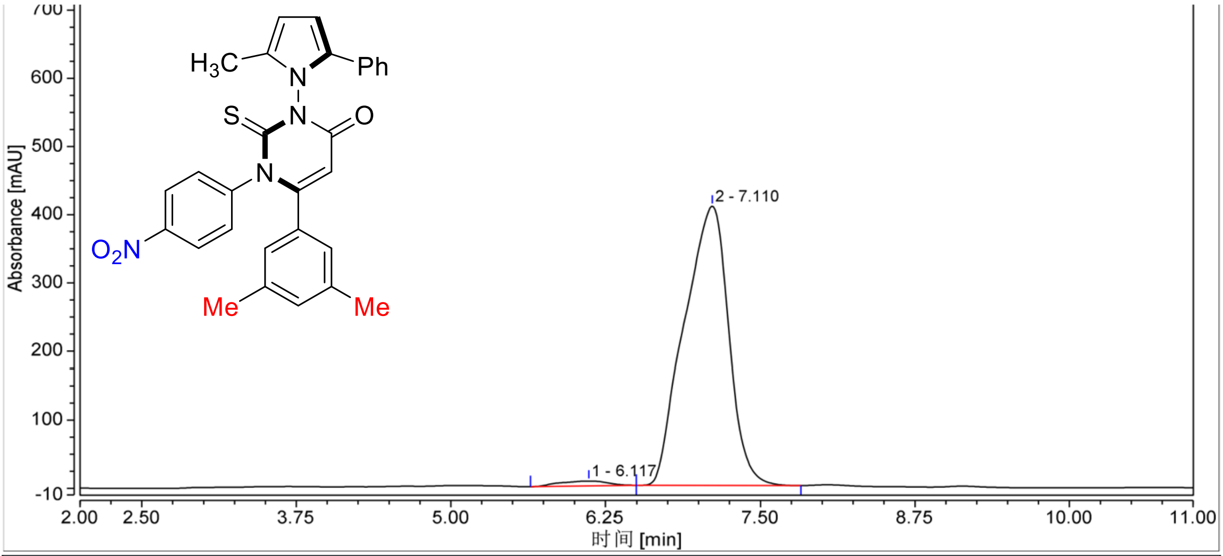

Result					
Peak	RT min	Area mAU*min	Height mAU	Area %	Height %
1	6.117	3.167	6.973	1.83	1.68
2	7.110	169.929	408.471	98.17	98.32

Supplementary Figure 313. HPLC chromatogram of enantiomerically enriched 7y

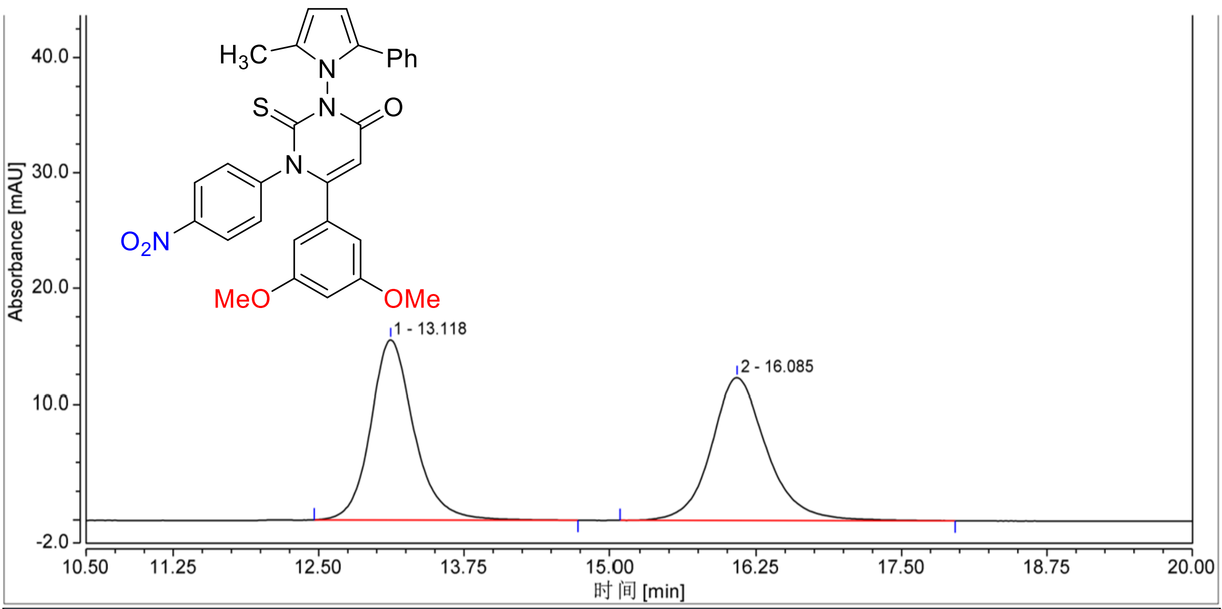

Result

Peak	RT<br>min	Area<br>mAU*min	Height<br>mAU	Area<br>%	Height<br>%
1	13.118	6.730	15.519	49.65	55.85
2	16.085	6.824	12.269	50.35	44.15

Supplementary Figure 314. HPLC chromatogram of racemic 7z

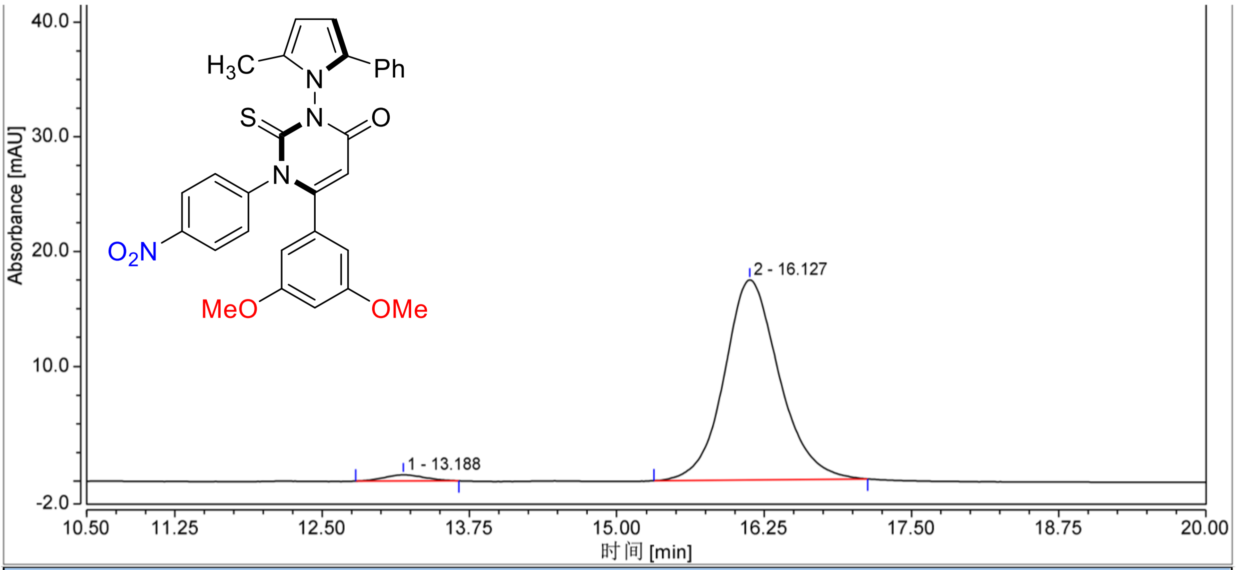

Result

Peak	RT<br>min	Area<br>mAU*min	Height<br>mAU	Area<br>%	Height<br>%
1	13.188	0.211	0.529	2.18	2.95
2	16.127	9.466	17.417	97.82	97.05

Supplementary Figure 315. HPLC chromatogram of enantiomerically enriched 7z

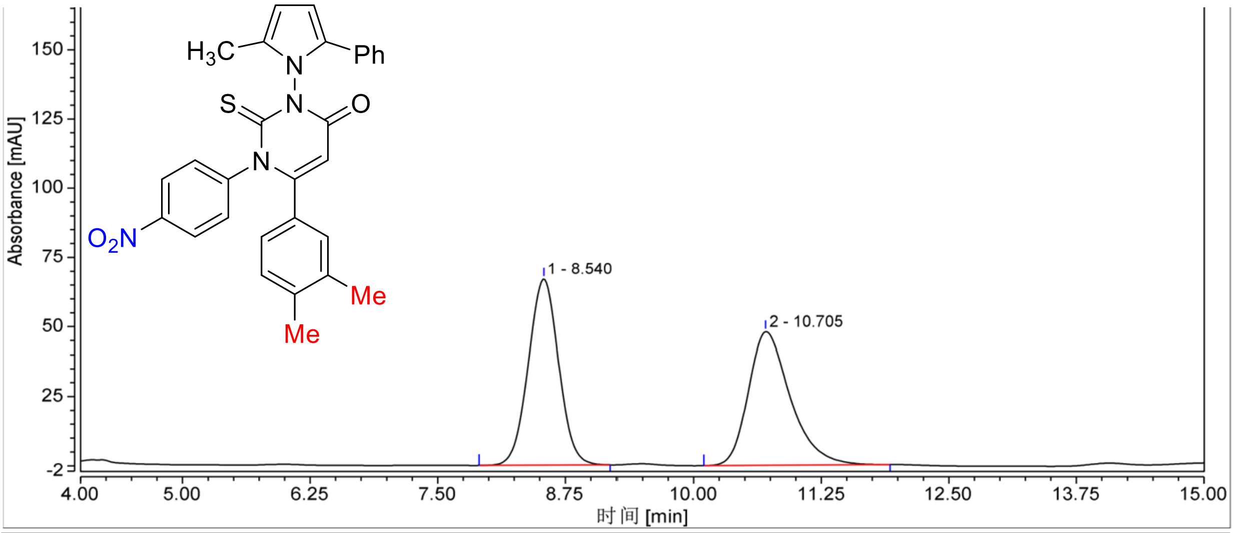

Result					
Peak	RT min	Area mAU*min	Height mAU	Area %	Height %
1	8.540	23.063	67.011	50.12	58.27
2	10.705	22.956	47.999	49.88	41.73

Supplementary Figure 316. HPLC chromatogram of racemic 7aa

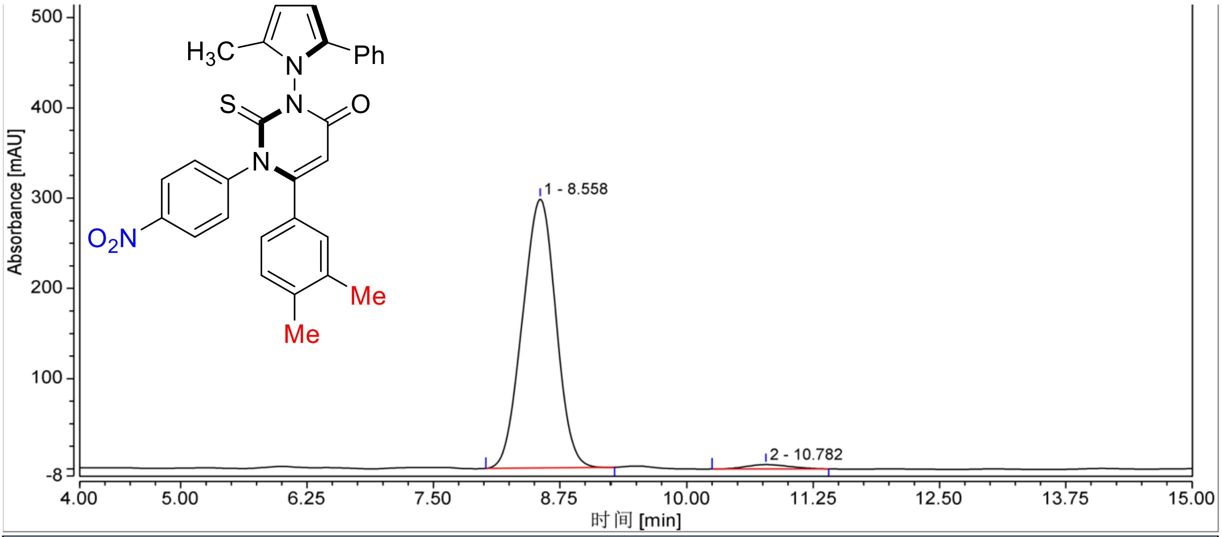

Result					
Peak	RT min	Area mAU*min	Height mAU	Area %	Height %
1	8.558	114.015	297.885	97.93	98.42
2	10.782	2.407	4.783	2.07	1.58

Supplementary Figure 317. HPLC chromatogram of enantiomerically enriched 7aa

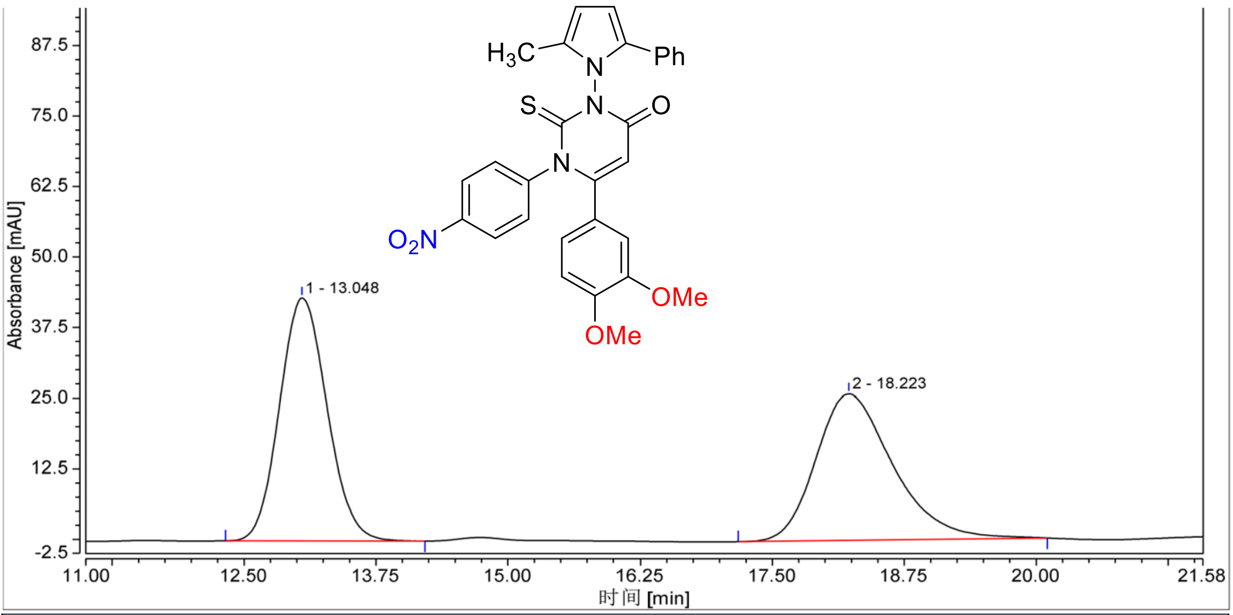

Result

Peak	RT<br>min	Area<br>mAU*min	Height<br>mAU	Area<br>%	Height<br>%
1	13.048	22.466	43.007	50.57	62.40
2	18.223	21.958	25.914	49.43	37.60

Supplementary Figure 318. HPLC chromatogram of racemic 7ab

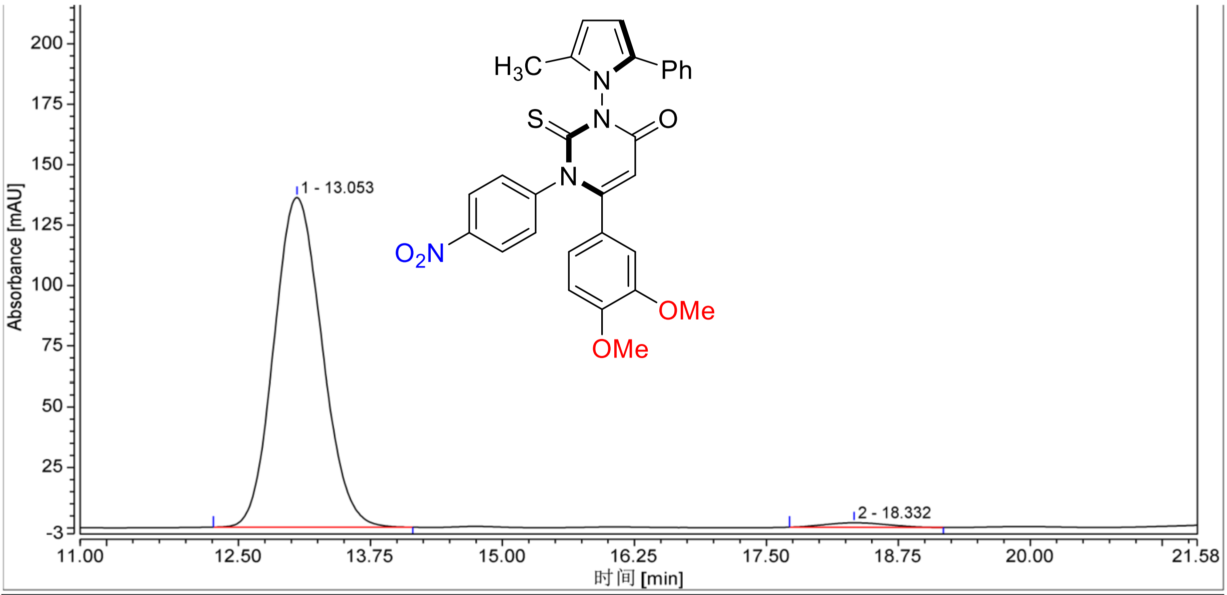

Result

Peak	RT<br>min	Area<br>mAU*min	Height<br>mAU	Area<br>%	Height<br>%
1	13.053	71.906	136.160	98.23	98.63
2	18.332	1.297	1.896	1.77	1.37

Supplementary Figure 319. HPLC chromatogram of enantiomerically enriched 7ab

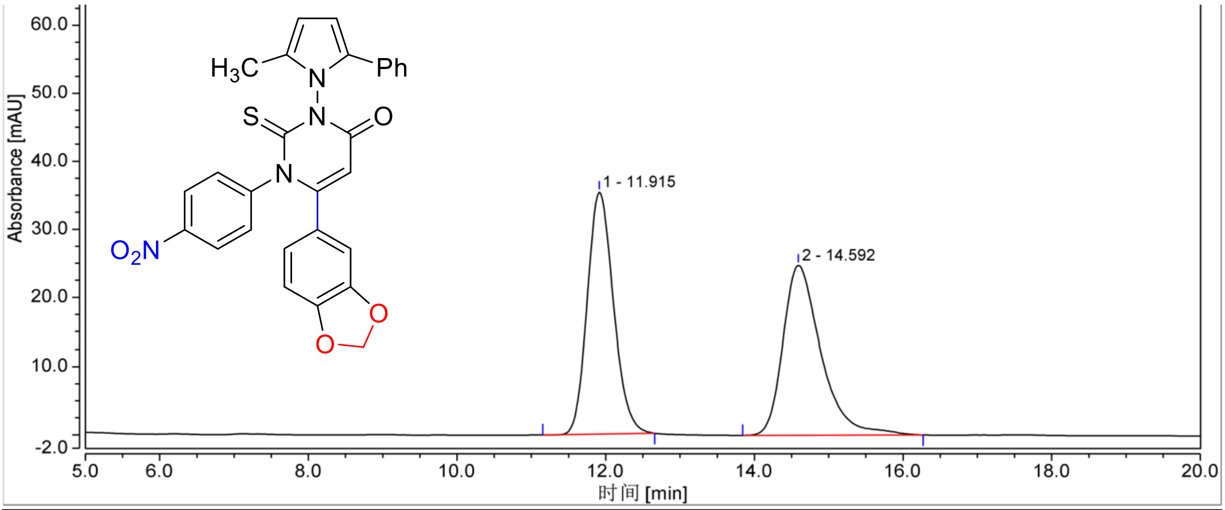

Result

Peak	RT min	Area mAU*min	Height mAU	Area %	Height %
1	11.915	14.437	35.366	49.62	58.81
2	14.592	14.657	24.771	50.38	41.19

Supplementary Figure 320. HPLC chromatogram of racemic 7ac

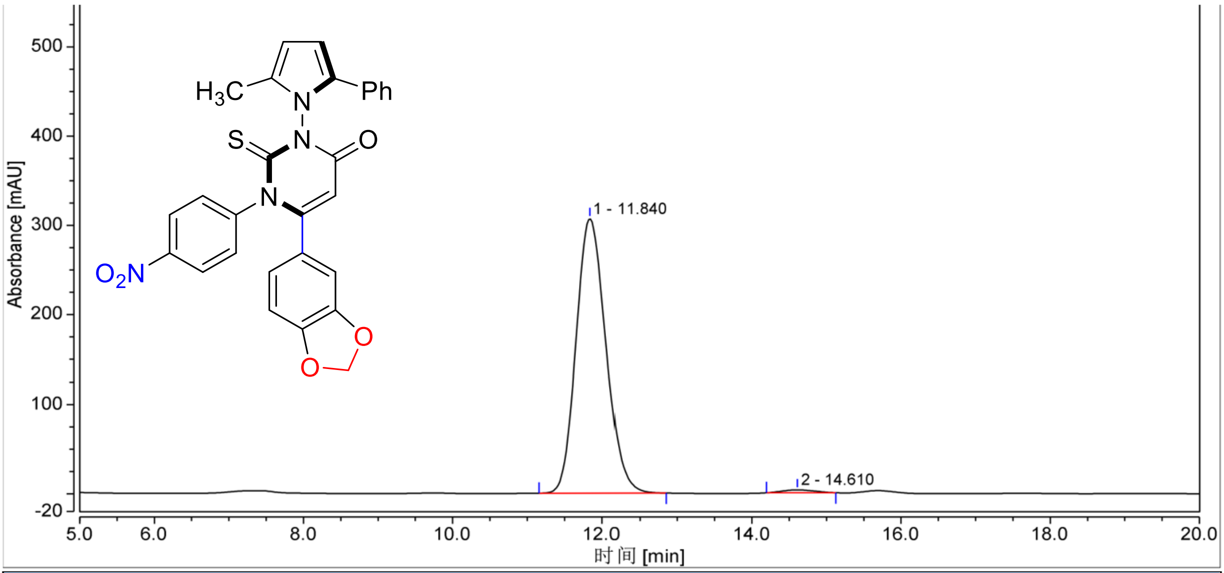

Result

Peak	RT min	Area mAU*min	Height mAU	Area %	Height %
1	11.840	141.044	306.706	98.87	98.97
2	14.610	1.619	3.199	1.13	1.03

Supplementary Figure 321. HPLC chromatogram of enantiomerically enriched 7ac

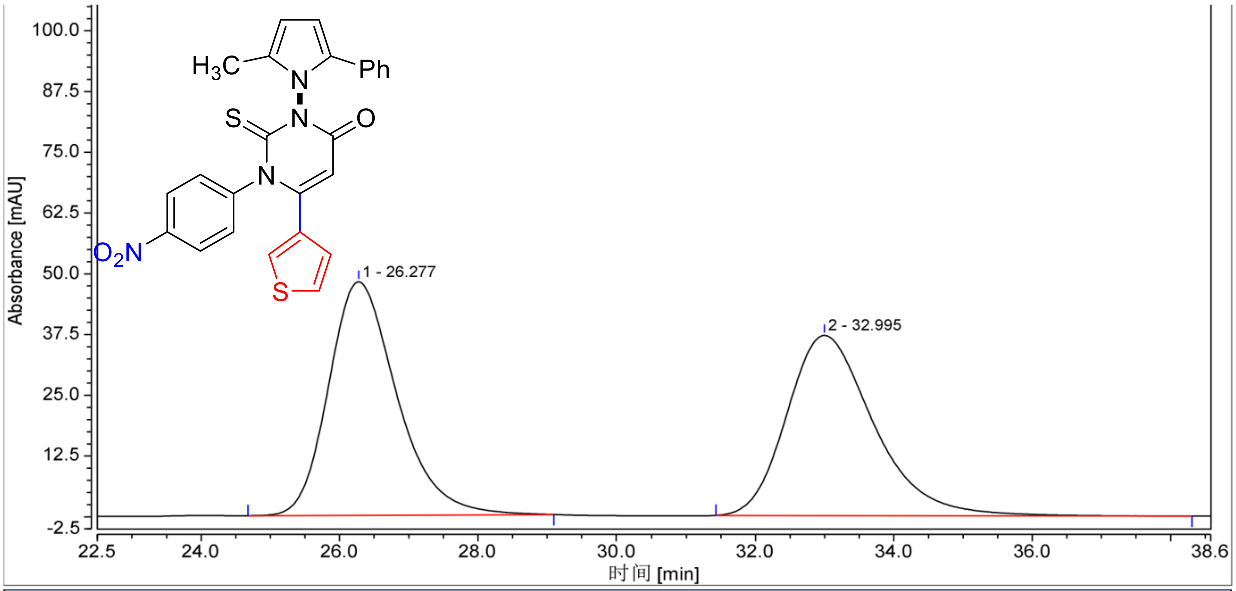

Result					
Peak	RT<br>min	Area<br>mAU*min	Height<br>mAU	Area<br>%	Height<br>%
1	26.277	55.054	48.045	50.45	56.44
2	32.995	54.079	37.074	49.55	43.56

Supplementary Figure 322. HPLC chromatogram of racemic 7ad

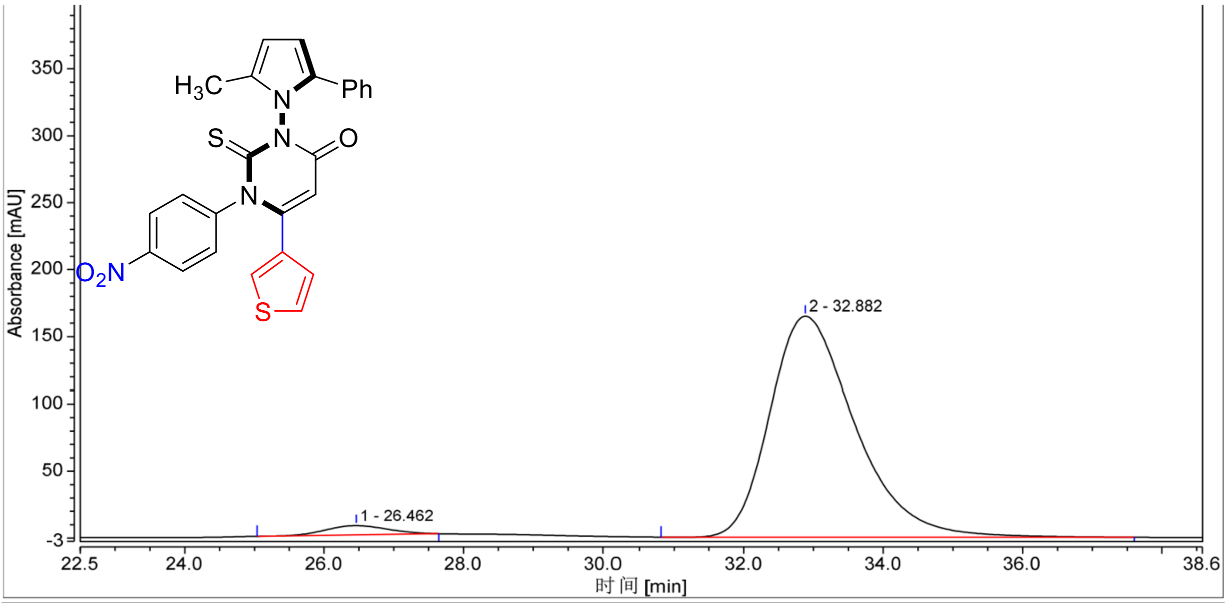

Result					
Peak	RT<br>min	Area<br>mAU*min	Height<br>mAU	Area<br>%	Height<br>%
1	26.462	7.181	6.896	2.99	4.02
2	32.882	233.245	164.477	97.01	95.98

Supplementary Figure 323. HPLC chromatogram of enantiomerically enriched 7ad

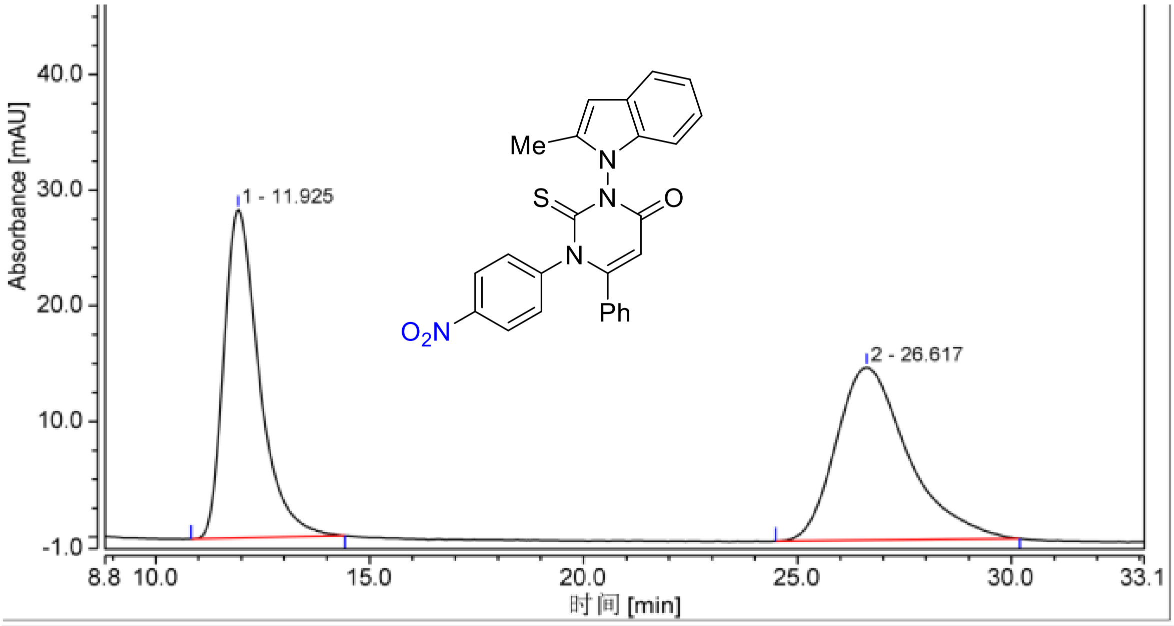

Result					
Peak	RT min	Area mAU*min	Height mAU	Area %	Height %
1	11.925	27.292	28.390	48.31	65.53
2	26.617	29.199	14.934	51.69	34.47

Supplementary Figure 324. HPLC chromatogram of racemic 7ae

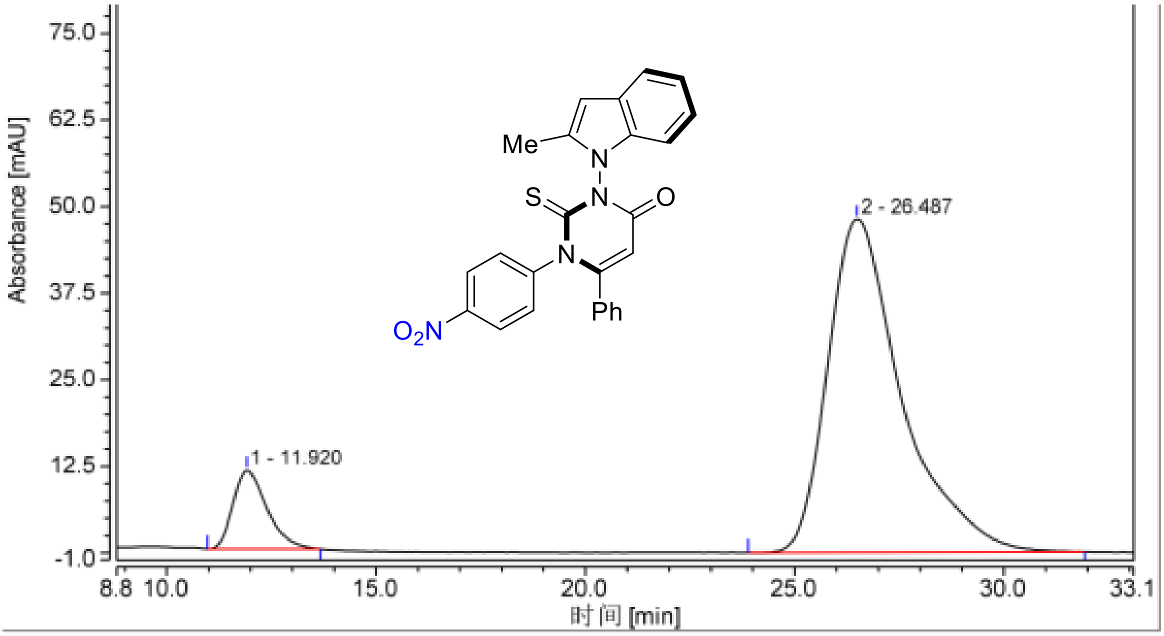

Result					
Peak	RT min	Area mAU*min	Height mAU	Area %	Height %
1	11.920	10.683	11.316	9.79	19.05
2	26.487	98.401	48.096	90.21	80.95

Supplementary Figure 325. HPLC chromatogram of enantiomerically enriched 7ae

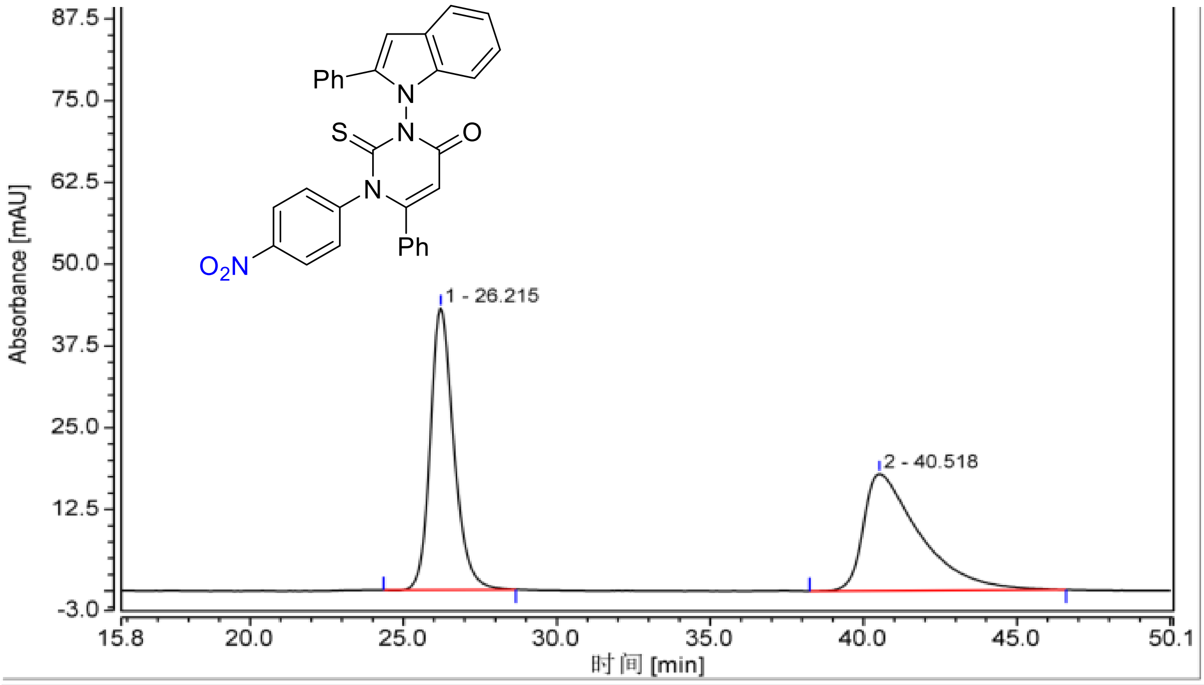

Result					
Peak	RT<br>min	Area<br>mAU*min	Height<br>mAU	Area<br>%	Height<br>%
1	26.215	38.289	43.065	50.20	70.68
2	40.518	37.991	17.862	49.80	29.32

Supplementary Figure 326. HPLC chromatogram of racemic 7af

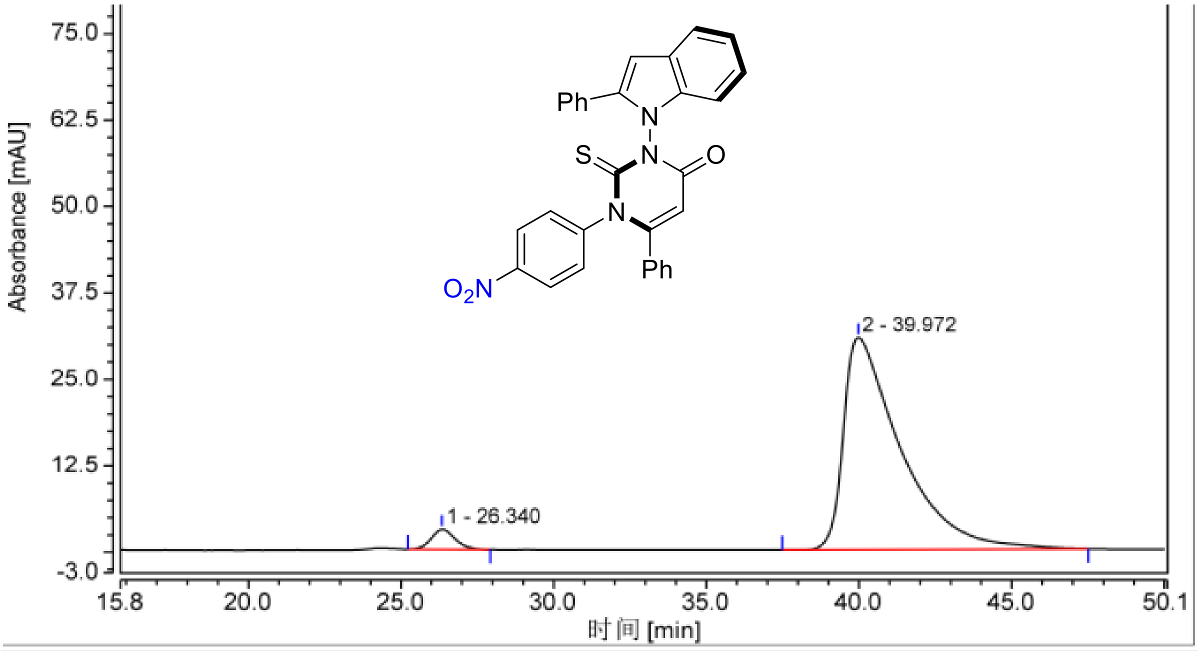

Result					
Peak	RT<br>min	Area<br>mAU*min	Height<br>mAU	Area<br>%	Height<br>%
1	26.340	2.595	2.860	3.77	8.54
2	39.972	66.292	30.646	96.23	91.46

Supplementary Figure 327. HPLC chromatogram of enantiomerically enriched 7af

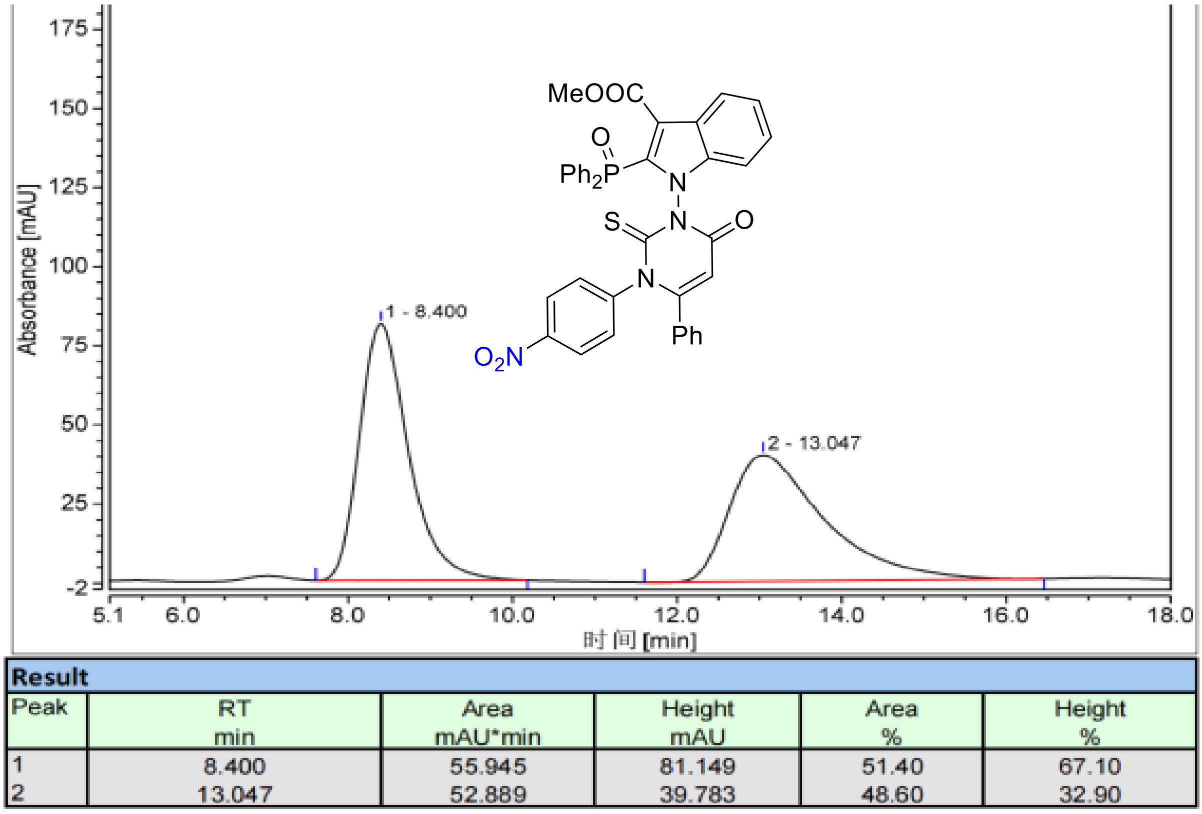

Result					
Peak	RT<br>min	Area<br>mAU*min	Height<br>mAU	Area<br>%	Height<br>%
1	8.400	55.945	81.149	51.40	67.10
2	13.047	52.889	39.783	48.60	32.90

Supplementary Figure 328. HPLC chromatogram of racemic 7ag

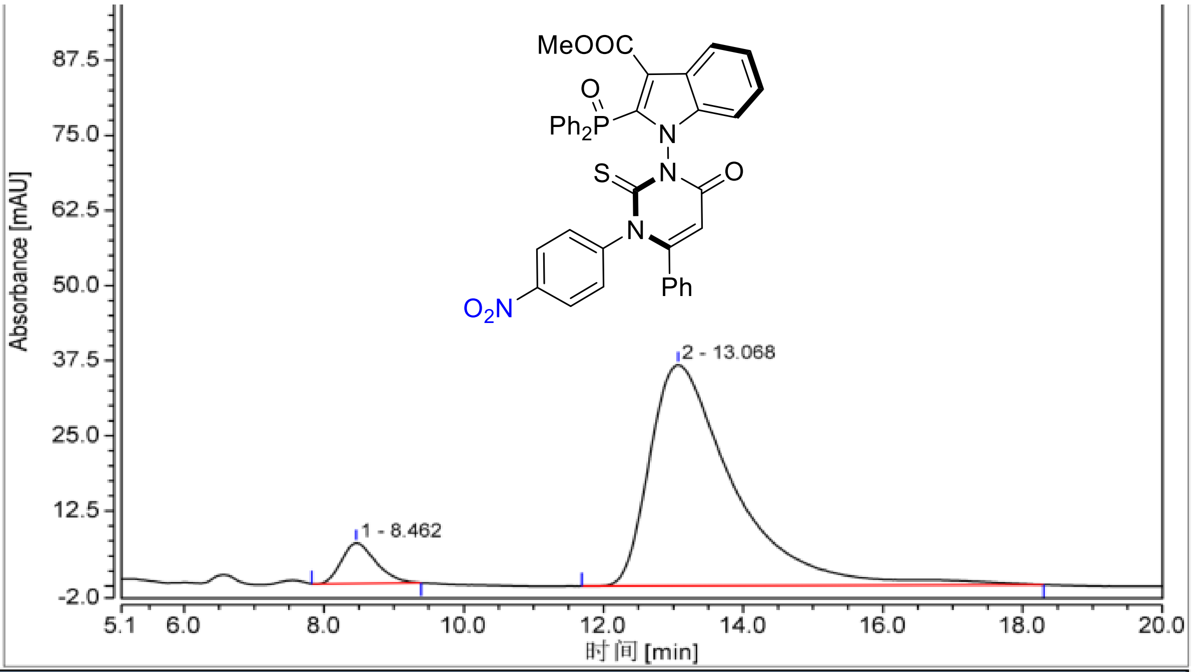

Result					
Peak	RT<br>min	Area<br>mAU*min	Height<br>mAU	Area<br>%	Height<br>%
1	8.462	3.831	6.700	6.96	15.42
2	13.068	51.189	36.744	93.04	84.58

Supplementary Figure 329. HPLC chromatogram of enantiomerically enriched 7ag

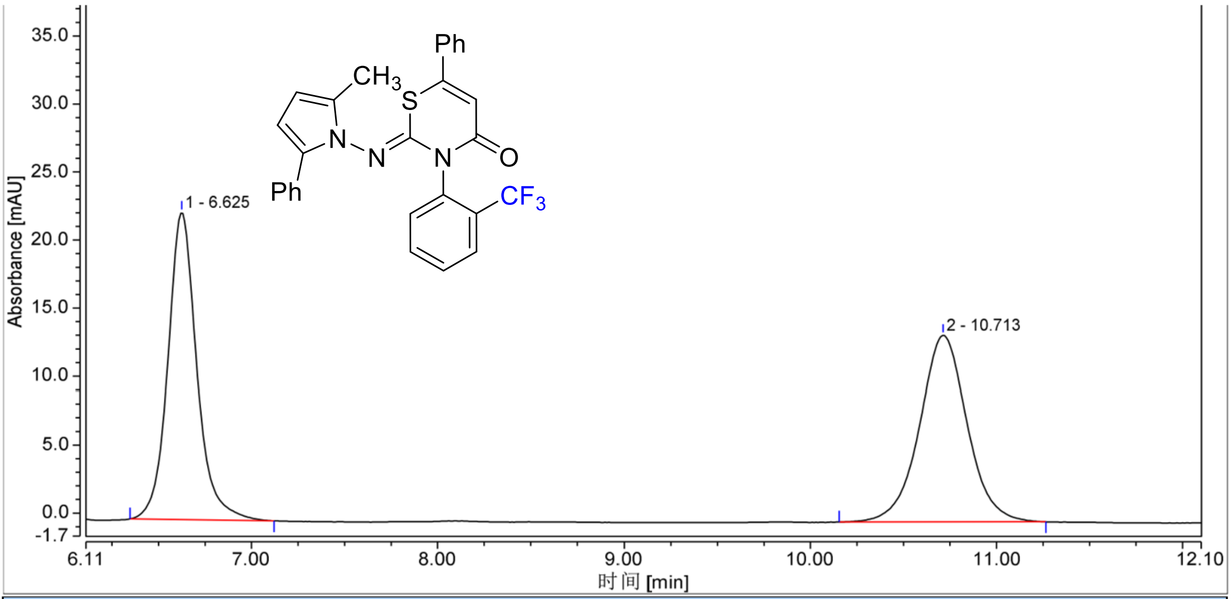

Result

Peak	RT min	Area mAU*min	Height mAU	Area %	Height %
1	6.625	4.055	22.511	50.04	62.26
2	10.713	4.048	13.648	49.96	37.74

Supplementary Figure 330. HPLC chromatogram of racemic 9a

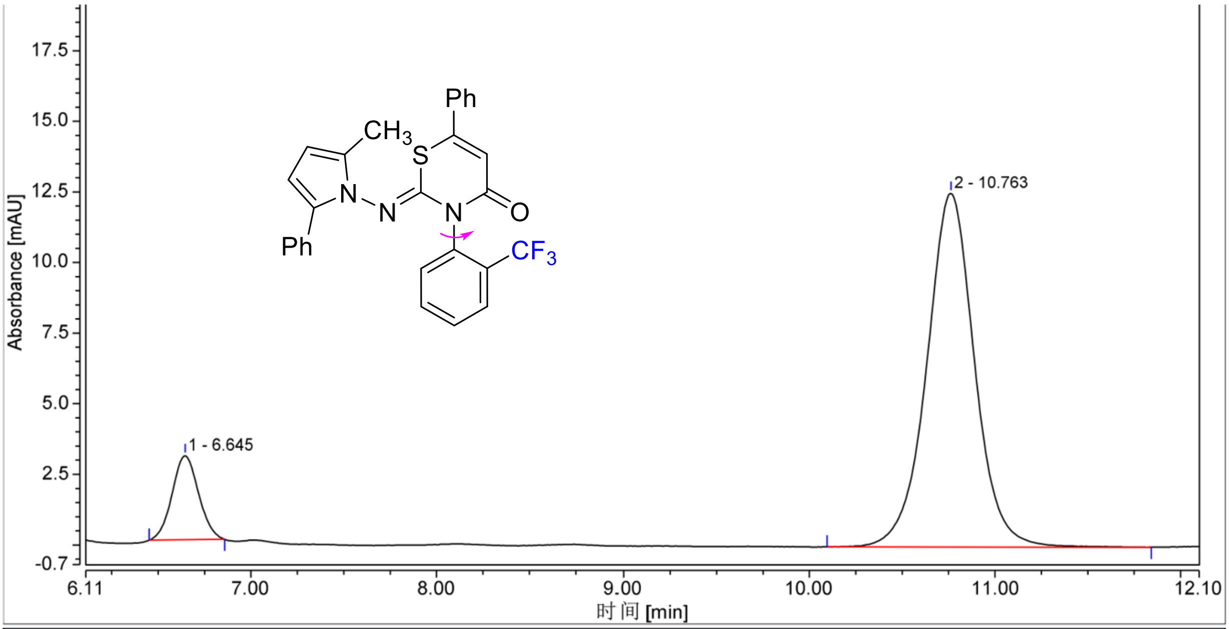

Result

Peak	RT min	Area mAU*min	Height mAU	Area %	Height %
1	6.645	0.493	2.983	11.66	19.24
2	10.763	3.734	12.525	88.34	80.76

Supplementary Figure 331. HPLC chromatogram of enantiomerically enriched 9a

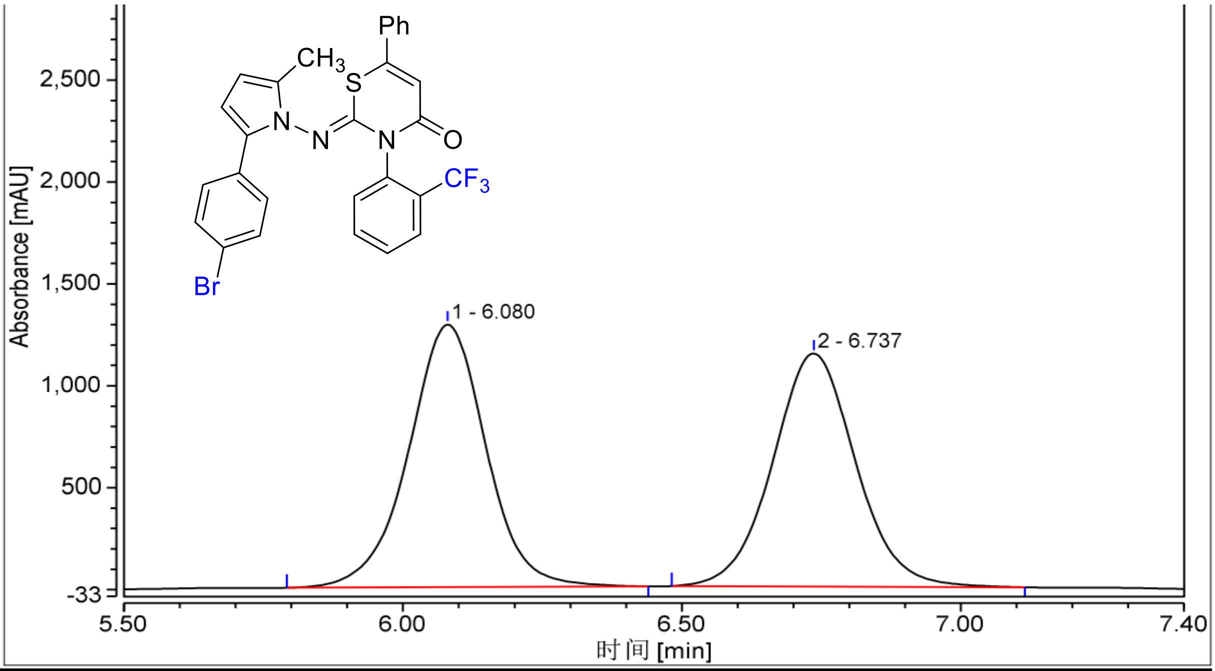

Result					
Peak	RT<br>min	Area<br>mAU*min	Height<br>mAU	Area<br>%	Height<br>%
1	6.080	207.717	1288.704	51.26	52.99
2	6.737	197.494	1143.074	48.74	47.01

Supplementary Figure 332. HPLC chromatogram of racemic 9b

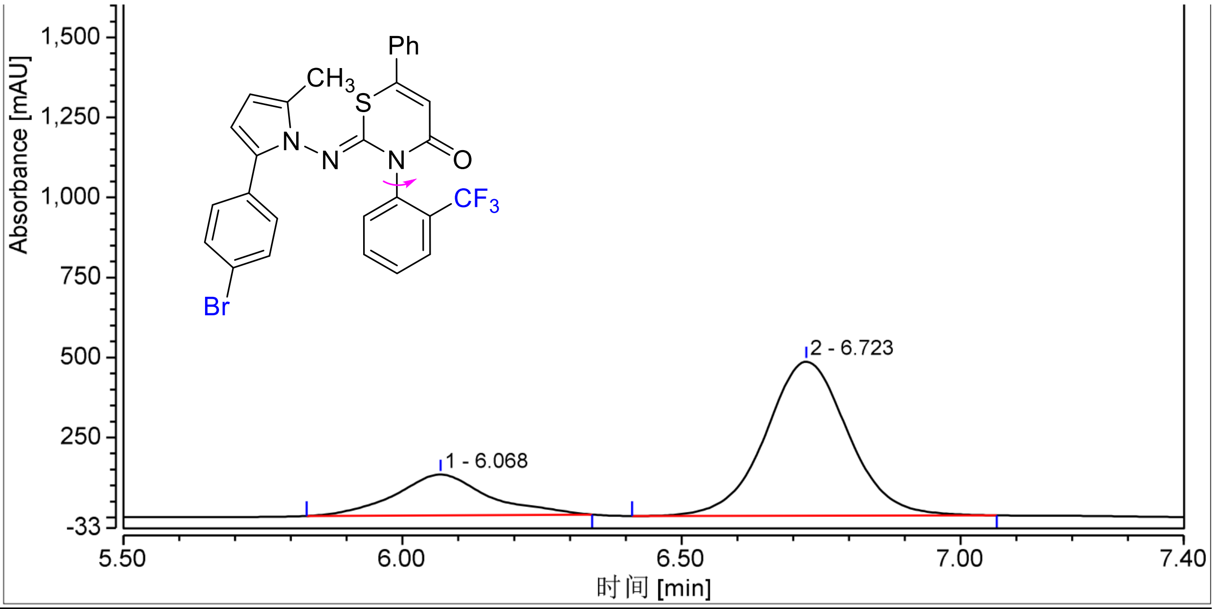

Result					
Peak	RT<br>min	Area<br>mAU*min	Height<br>mAU	Area<br>%	Height<br>%
1	6.068	24.730	127.337	22.98	20.92
2	6.723	82.880	481.319	77.02	79.08

Supplementary Figure 333. HPLC chromatogram of enantiomerically enriched 9b

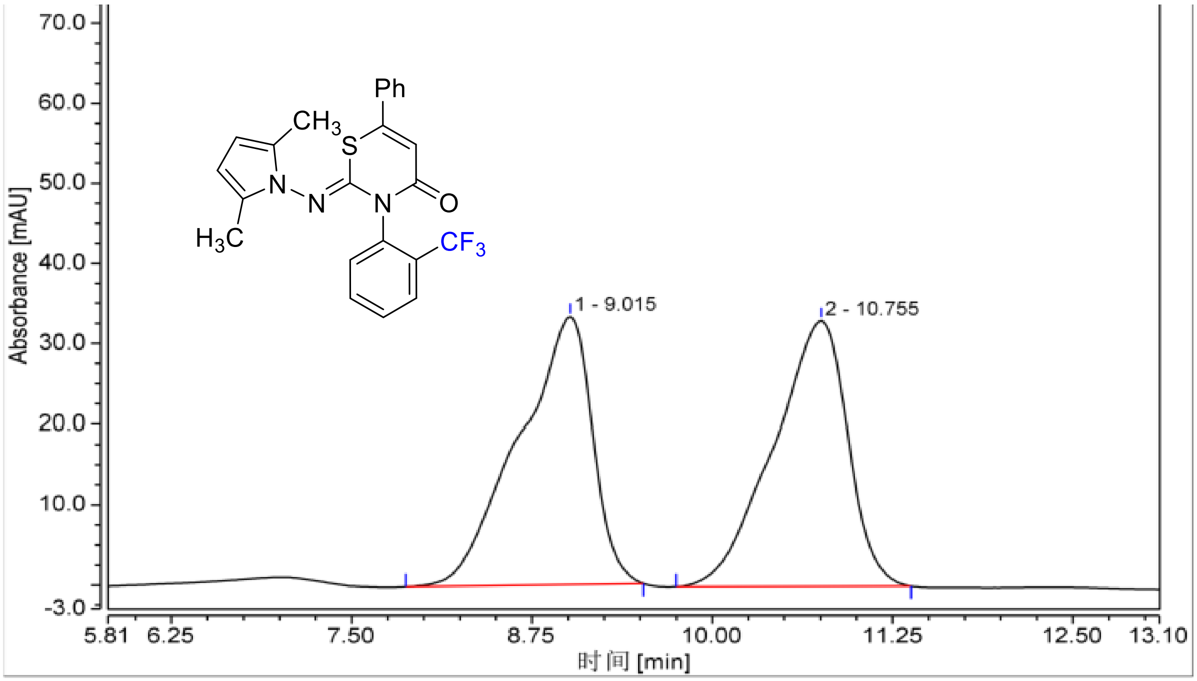

Result					
Peak	RT<br>min	Area<br>mAU*min	Height<br>mAU	Area<br>%	Height<br>%
1	9.015	19.740	33.267	50.05	50.20
2	10.755	19.702	32.998	49.95	49.80

Supplementary Figure 334. HPLC chromatogram of racemic 9c

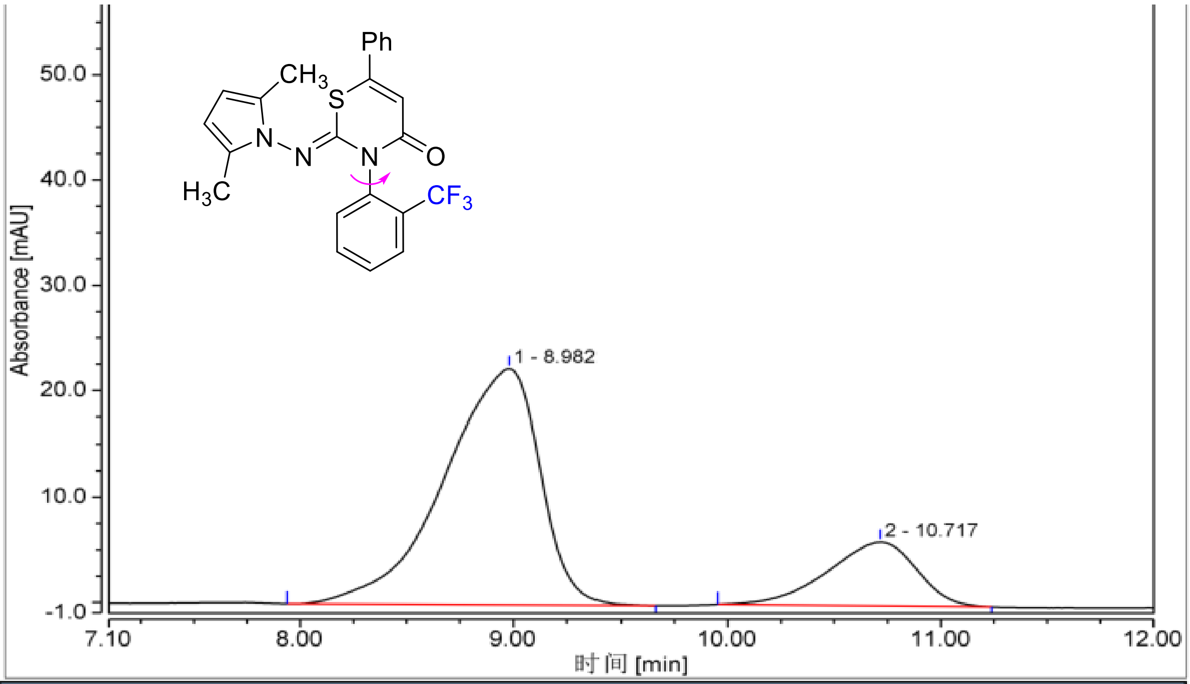

Result					
Peak	RT<br>min	Area<br>mAU*min	Height<br>mAU	Area<br>%	Height<br>%
1	8.982	11.699	22.309	79.96	78.62
2	10.717	2.933	6.066	20.04	21.38

Supplementary Figure 335.HPLC chromatogram of enantiomerically enriched 9c

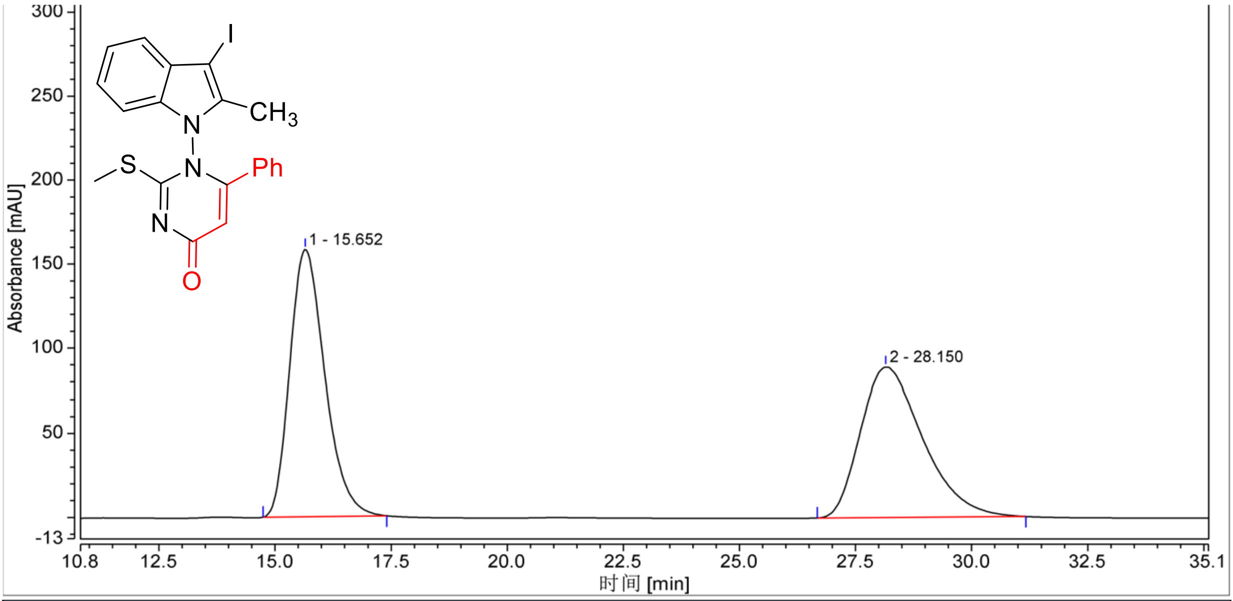

Result					
Peak	RT<br>min	Area<br>mAU*min	Height<br>mAU	Area<br>%	Height<br>%
1	15.652	138.551	158.239	50.01	64.07
2	28.150	138.516	88.725	49.99	35.93

Supplementary Figure 336. HPLC chromatogram of racemic 4

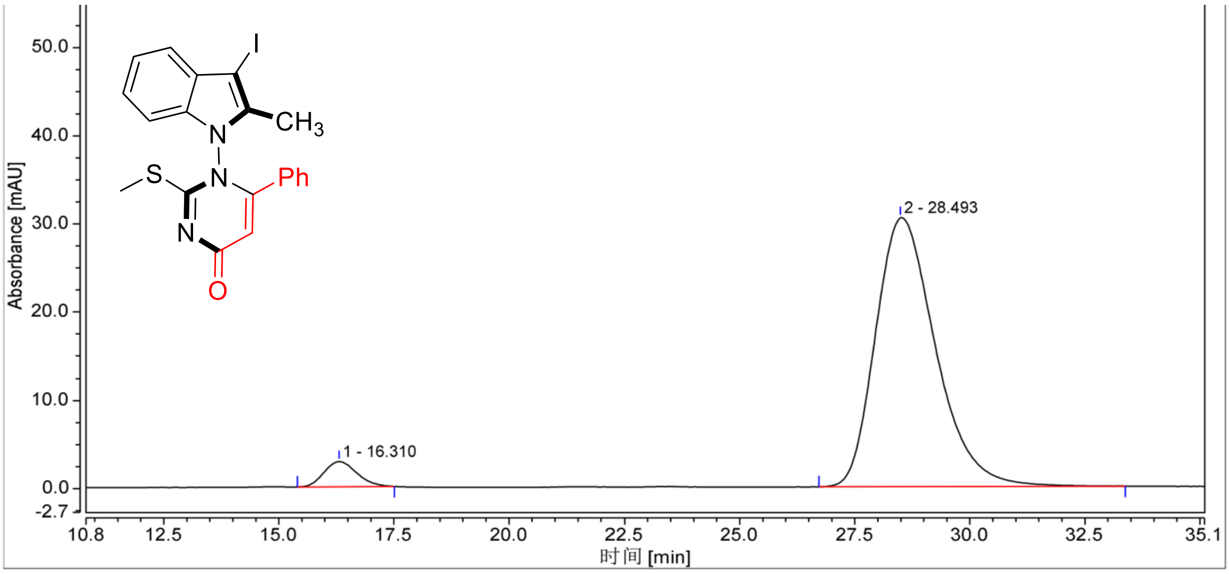

Result					
Peak	RT<br>min	Area<br>mAU*min	Height<br>mAU	Area<br>%	Height<br>%
1	16.310	2.377	2.869	4.91	8.60
2	28.493	46.016	30.485	95.09	91.40

Supplementary Figure 337. HPLC chromatogram of enantiomerically enriched 4

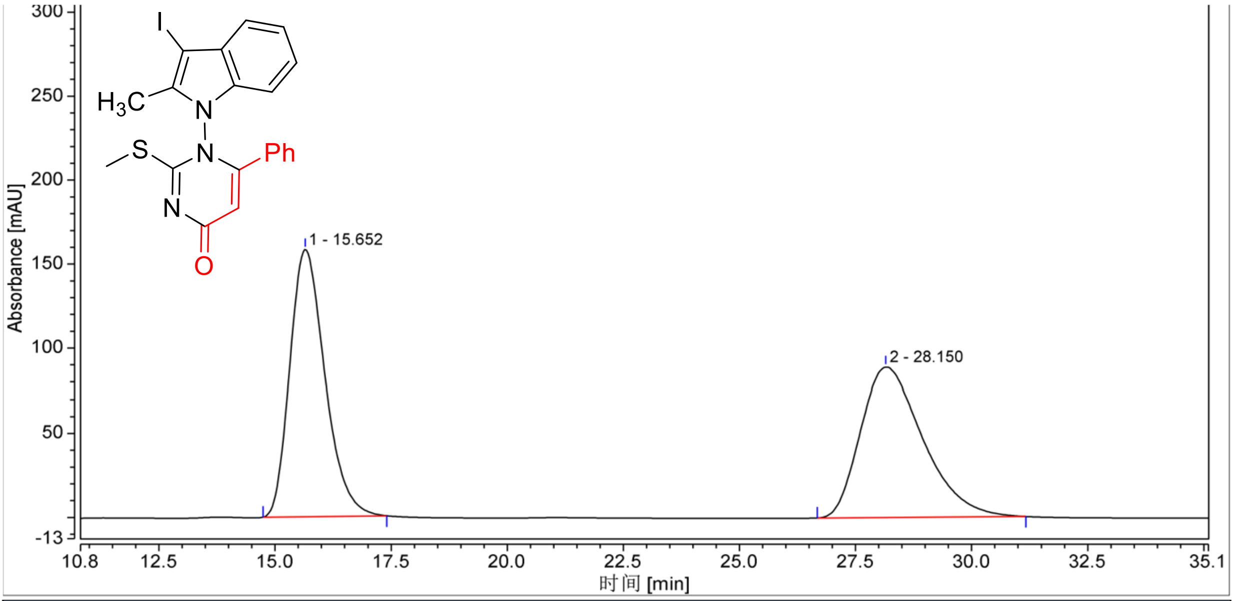

Result

Peak	RT<br>min	Area<br>mAU*min	Height<br>mAU	Area<br>%	Height<br>%
1	15.652	138.551	158.239	50.01	64.07
2	28.150	138.516	88.725	49.99	35.93

Supplementary Figure 338. HPLC chromatogram of racemic 4

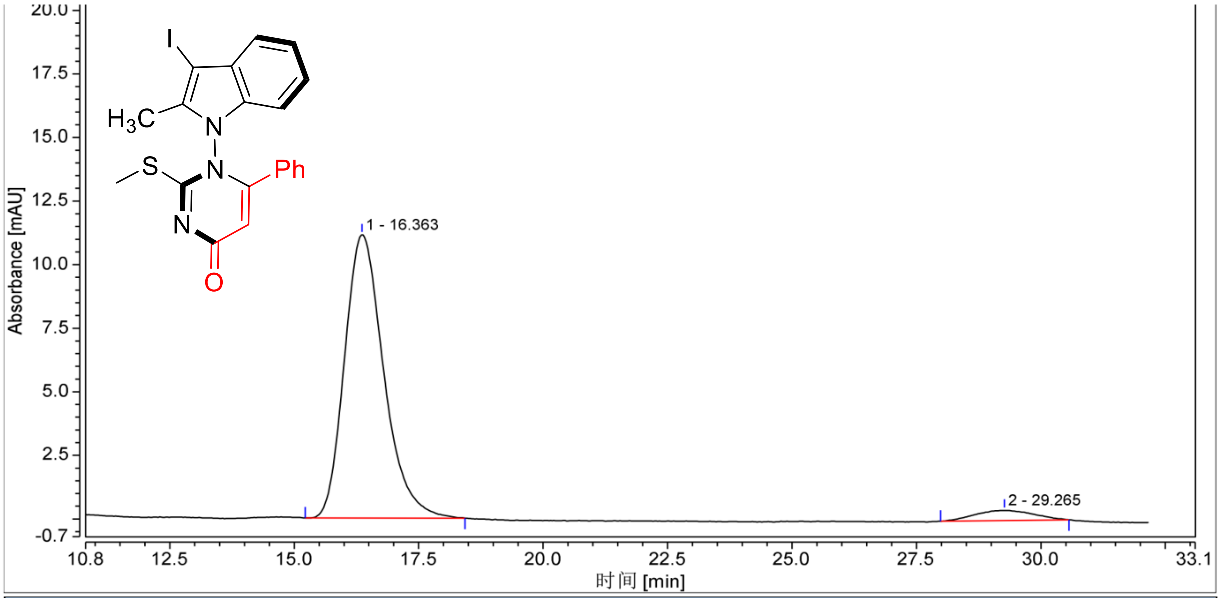

Result

Peak	RT<br>min	Area<br>mAU*min	Height<br>mAU	Area<br>%	Height<br>%
1	16.363	10.168	11.137	94.99	96.55
2	29.265	0.537	0.398	5.01	3.45

Supplementary Figure 339. HPLC chromatogram of enantiomerically enriched *ent*-4

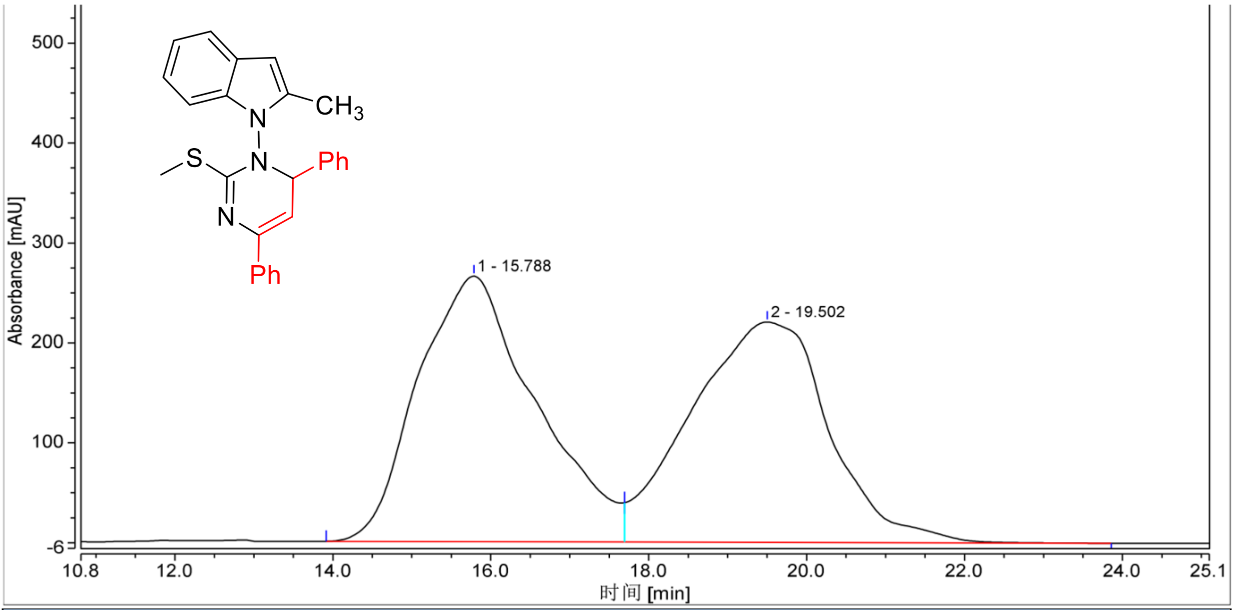

Result

Peak	RT min	Area mAU*min	Height mAU	Area %	Height %
1	15.788	459.764	265.733	50.70	54.65
2	19.502	447.069	220.490	49.30	45.35

Supplementary Figure 340. HPLC chromatogram of racemic 10

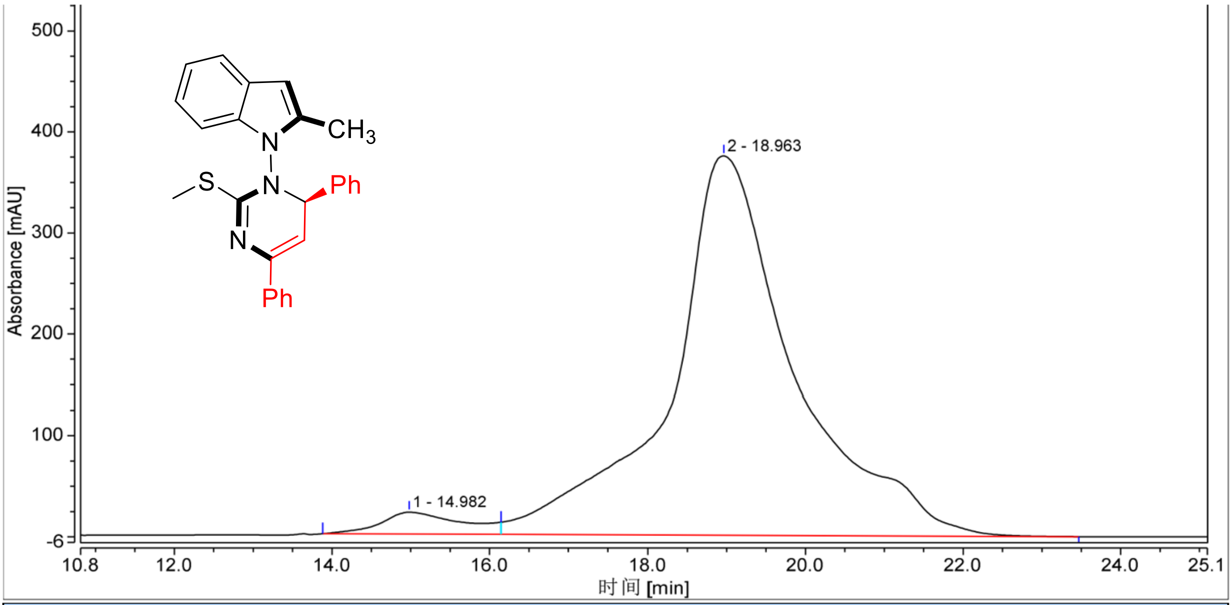

Result

Peak	RT min	Area mAU*min	Height mAU	Area %	Height %
1	14.982	25.971	21.631	3.65	5.46
2	18.963	685.121	374.781	96.35	94.54

Supplementary Figure 341. HPLC chromatogram of enantiomerically enriched 10

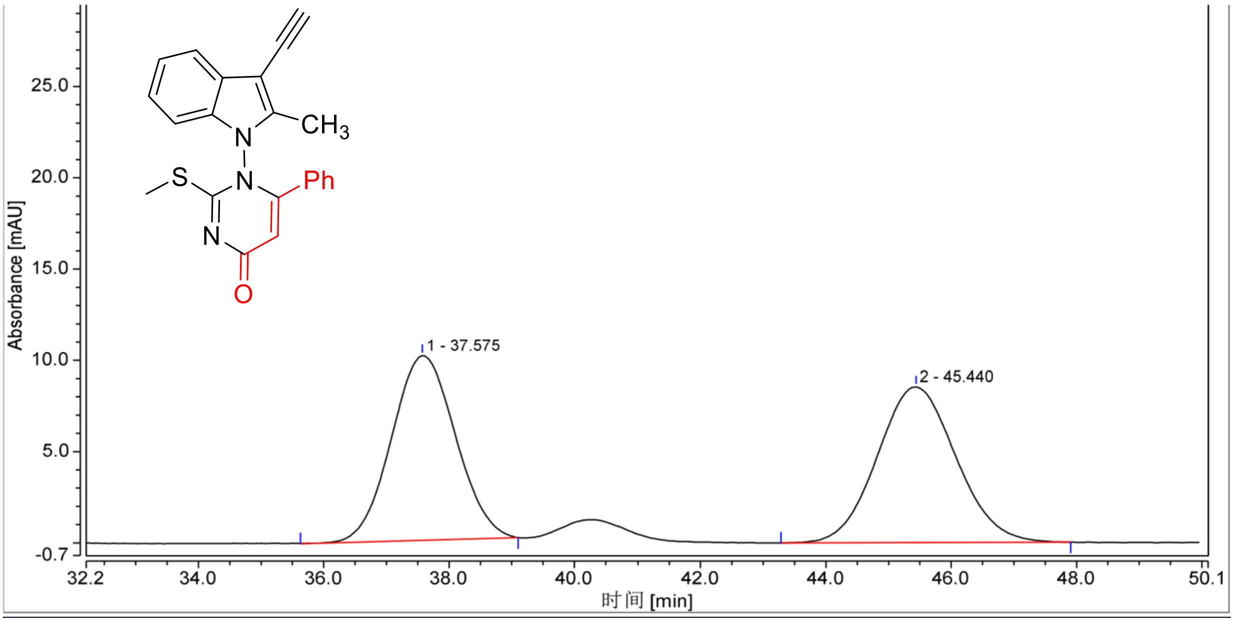

Result

Peak	RT<br>min	Area<br>mAU*min	Height<br>mAU	Area<br>%	Height<br>%
1	37.575	11.985	10.125	49.16	54.28
2	45.440	12.397	8.528	50.84	45.72

Supplementary Figure 342. HPLC chromatogram of racemic 11

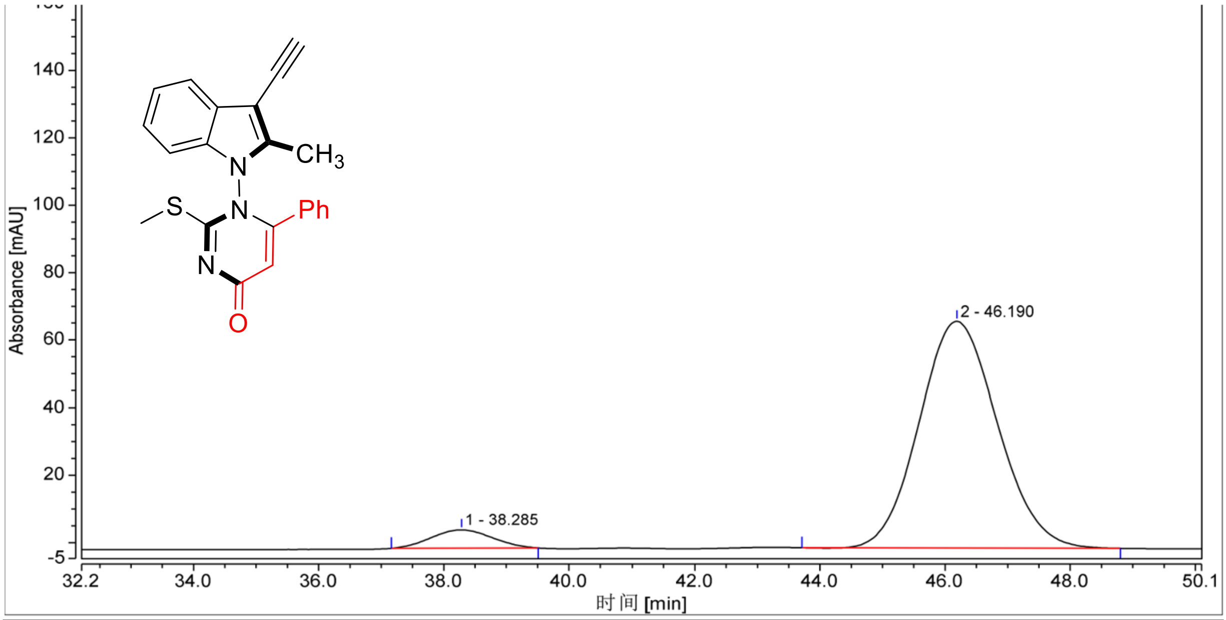

Result

Peak	RT<br>min	Area<br>mAU*min	Height<br>mAU	Area<br>%	Height<br>%
1	38.285	6.111	5.440	5.91	7.50
2	46.190	97.289	67.063	94.09	92.50

Supplementary Figure 343. HPLC chromatogram of enantiomerically enriched 11

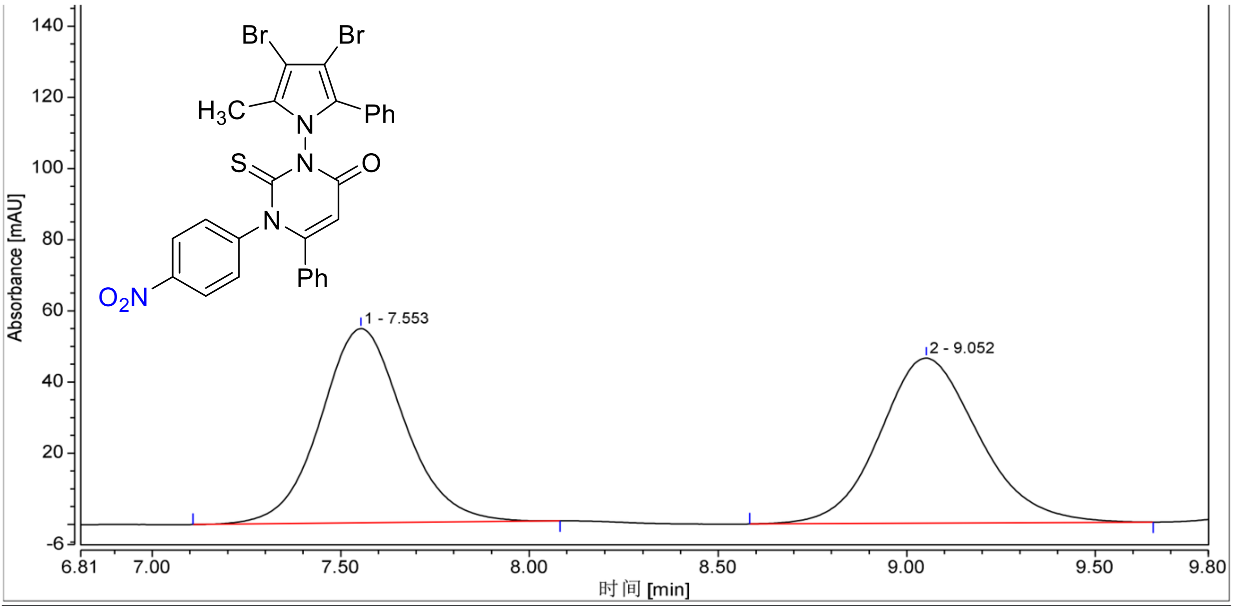

Result

Peak	RT<br>min	Area<br>mAU*min	Height<br>mAU	Area<br>%	Height<br>%
1	7.553	14.064	54.601	49.83	54.07
2	9.052	14.161	46.383	50.17	45.93

Supplementary Figure 344. HPLC chromatogram of racemic 12

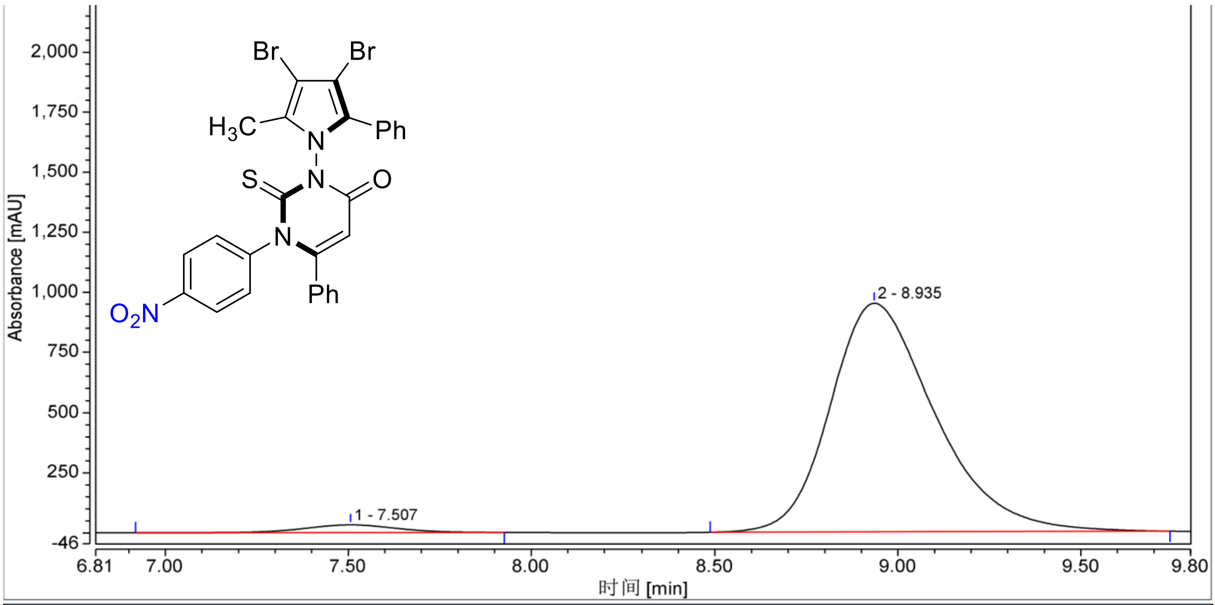

Result

Peak	RT<br>min	Area<br>mAU*min	Height<br>mAU	Area<br>%	Height<br>%
1	7.507	9.411	32.166	2.89	3.27
2	8.935	316.476	950.049	97.11	96.73

Supplementary Figure 345. HPLC chromatogram of enantiomerically enriched 12

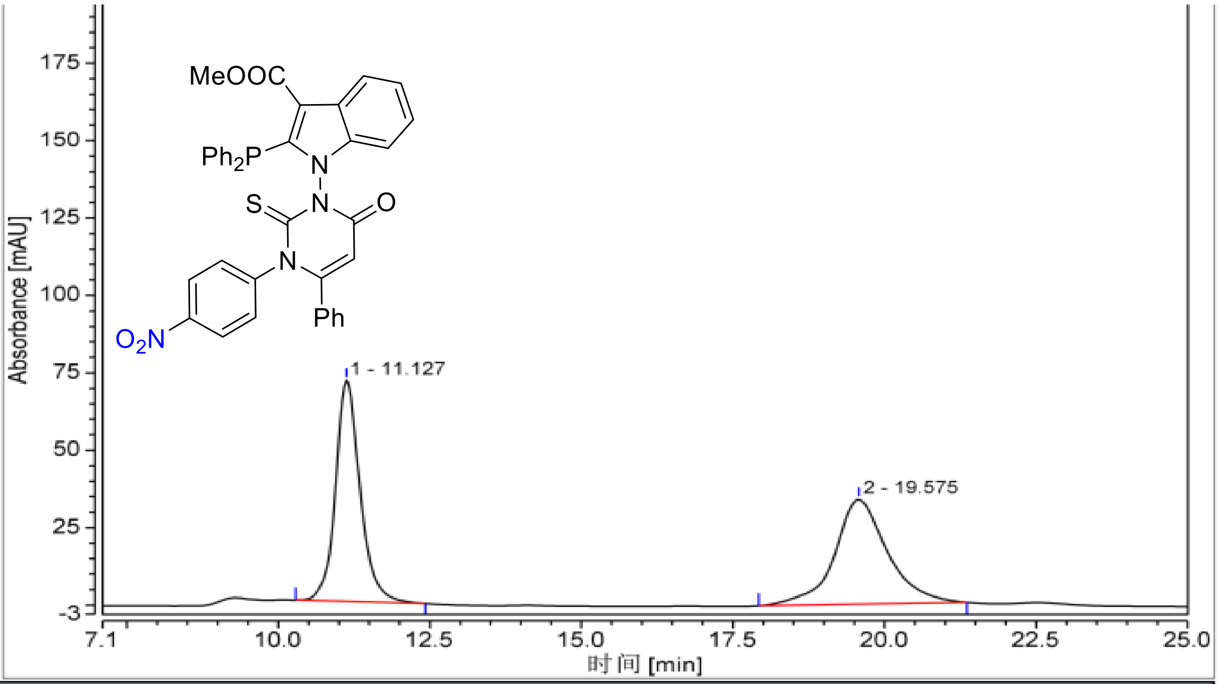

Peak	RT min	Area mAU*min	Height mAU	Area %	Height %
1	11.127	33.628	71.353	49.63	67.88
2	19.575	34.134	33.762	50.37	32.12

Supplementary Figure 346. HPLC chromatogram of racemic 13

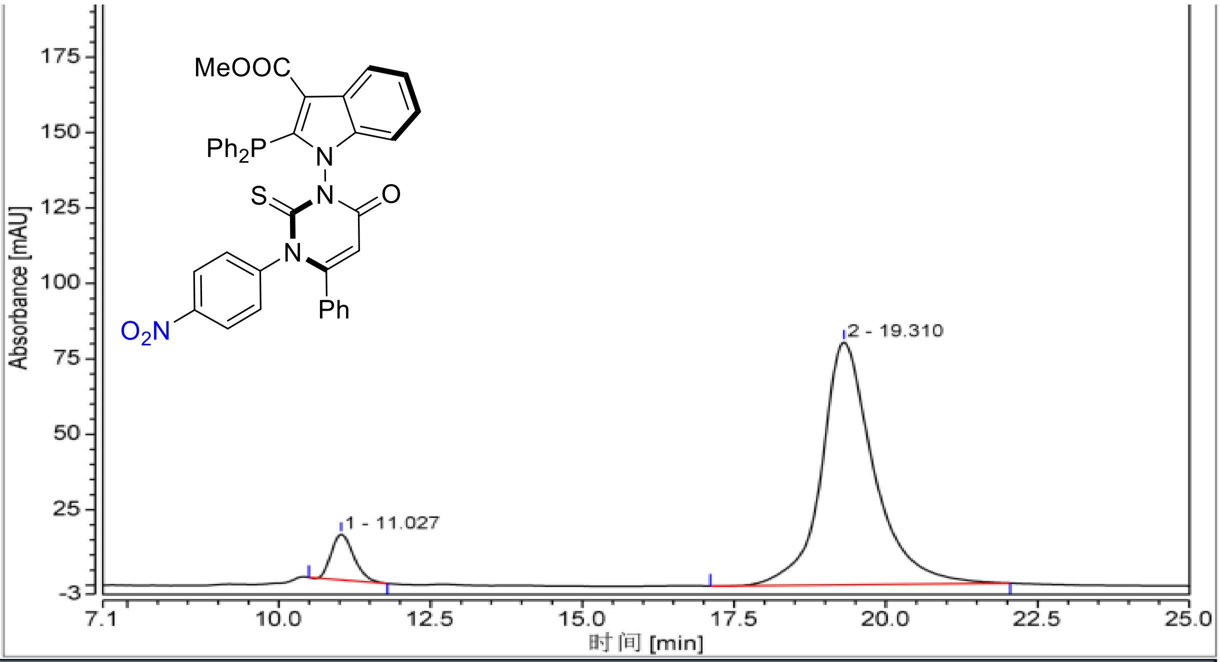

Peak	RT min	Area mAU*min	Height mAU	Area %	Height %
1	11.027	6.477	15.095	7.47	15.80
2	19.310	80.247	80.418	92.53	84.20

Supplementary Figure 347. HPLC chromatogram of enantiomerically enriched 13

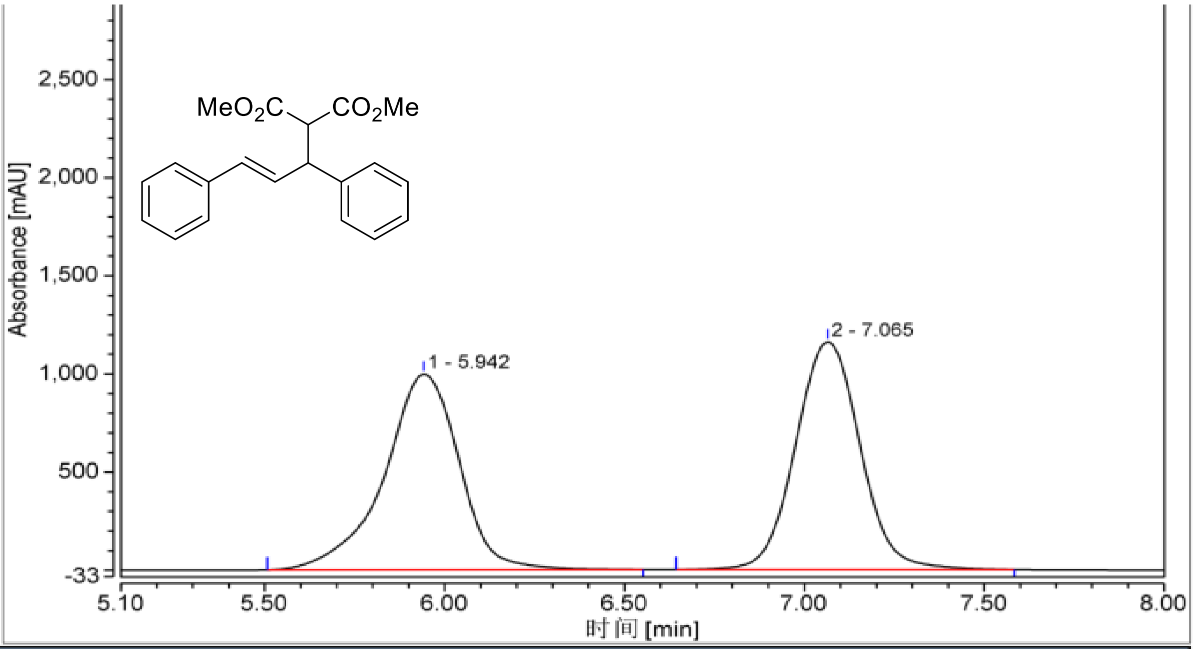

Result					
Peak	RT<br>min	Area<br>mAU*min	Height<br>mAU	Area<br>%	Height<br>%
1	5.942	241.492	996.284	49.95	46.21
2	7.065	242.007	1159.937	50.05	53.79

Supplementary Figure 348. HPLC chromatogram of racemic 16

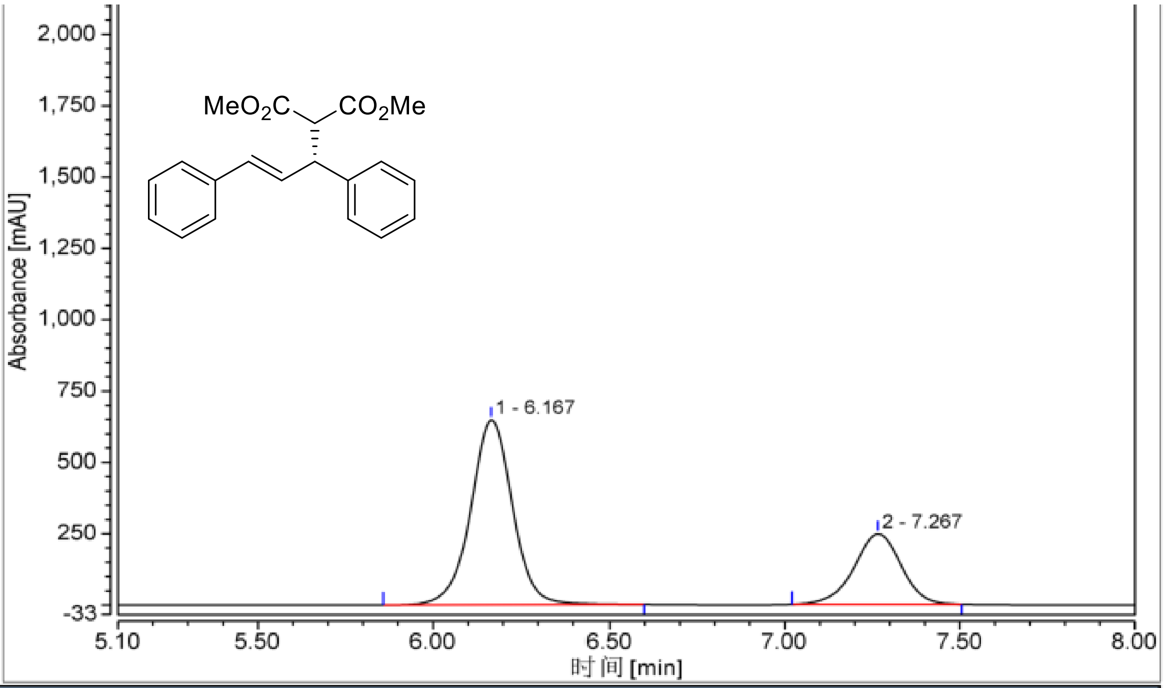

Result					
Peak	RT<br>min	Area<br>mAU*min	Height<br>mAU	Area<br>%	Height<br>%
1	6.167	89.959	647.589	69.95	72.40
2	7.267	38.639	246.870	30.05	27.60

Supplementary Figure 349. HPLC chromatogram of enantiomerically enriched 16

12. Computational Methods

Geometry optimizations and frequency calculations were performed at M06-2X/6-31G(d) level. To verify the nature of the located transition state structures, IRC were performed at the same level of theory for selected transition states only. For N-N rotational transition states, the imaginary frequencies were too low. Thus, IRC calculations became very challenging to carry out. Instead, visual inspections were used to check these translational transition states being the correct ones. Higher level single-point energy calculations were performed at M06-2X/6-311+G(2d,p) level. Solvent effect was incorporated using the SMD[3] implicit reaction field model and toluene as the solvent. Relative free energies were computed at the temperature of 298 K, except for the rotational barrier calculations, which were performed at 423 K. An entropy correction term to free energy values, of the magnitude of 7.06 kJ/mol at 298 K, was included to account for the change of state from gaseous to standard state of 1 M.[4] It does not affect the barrier of unimolecular reactions, so was not included in the free energy values at 423 K. For transition states and intermediates, it is important to explore the conformational space and locate the lowest-energy conformers. Due to the rigidity of NHC and substrates, our conformational search yielded only a few distinct conformers. Higher-energy conformers were reported alongside the lowest-energy ones whenever possible. Graphics of optimized structures were rendered using the CYLview program.[5] All DFT calculations were carried out with the Gaussian 16 suite of programs.[6]

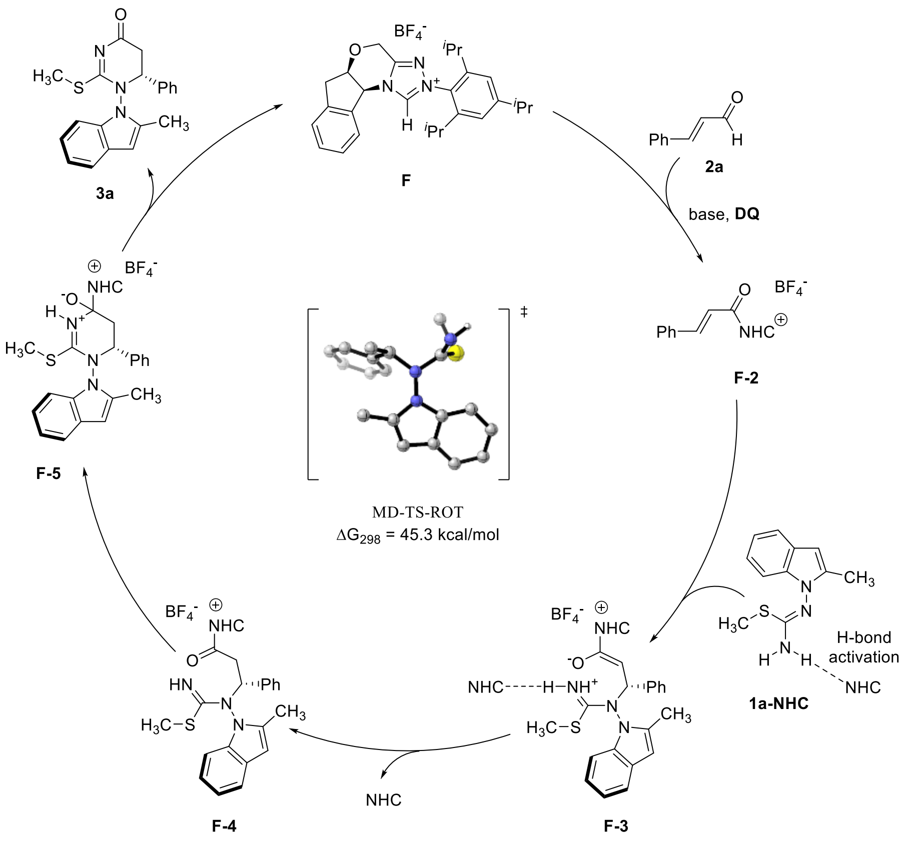

Supplementary Figure 350. Proposed catalytic cycle. C-H hydrogens are hidden for clarity.

Reaction Stereoselectivity

We calculated the four diastereomeric transition states of **F-TS2**, and the result was shown in **Supplementary Table 8**. Compared to experimental diastereoselectivity of 3:1, our predicted dr values, based on relative energies and enthalpies of F-TS2-RS_a with respect to F-TS2-RR_a, were in excellent agreement. Reversed diastereoselectivity of 1:9.6 was obtained when relative Gibbs free energies were computed. It has been known that accurate entropy calculations of such systems as contain many low-lying frequencies are very challenging. In the current case, the second carbene catalyst only forms a weak N-H···C hydrogen bond with the nucleophile **1a** in **F-TS2**. Although it helps to activate **1a** and lowers the reaction barrier, it also gives rise to large amount of errors in the calculated Gibbs free energies.

The predicted ee values, irrespective of the type of energy used, are all in excess of 99%, despite of our extensive conformational study of SS_a transition states. This is only in qualitative agreement with the general high levels of enantioselectivities observed for NHC catalysts. The discrepancy of the predicted ee value may be attributed to two possible reasons. Firstly, calculated entropy values typically contain a large amount of uncertainty, especially in the current case where the second NHC catalyst binds to the rest of the TS moiety through a non-covalent interaction. Secondly, there lacks a systematic approach to the conformational search of **TS2-*SS*a**, with respect to the location and orientation of the second NHC catalyst. Nevertheless, it is likely that the origin of enantioselectivity can still be attributed to transition state models reported in our earlier works.[7]

Supplementary Table 8. Relative energies, enthalpies and free energies of all four diastereomeric transition states of **F**-catalyzed reactions, with respect to RR_a, calculated at M06-2X/6-311+G(2d,p)-SMD(toluene)//M06-2X/6-31G(d)-SMD(toluene) level and 298 K. Energies are reported in kJ/mol.

Energy	RR_a	RS_a	SS_a	SR_a	ee^{pred}	dr^{pred}
**ΔE**	0.0	3.0	20.2	33.1	> 99%	3.4:1
**ΔH**	0.0	2.0	20.1	33.8	> 99%	2.2:1
**ΔG**	0.0	-5.6	18.6	30.5	> 99%	1:9.6

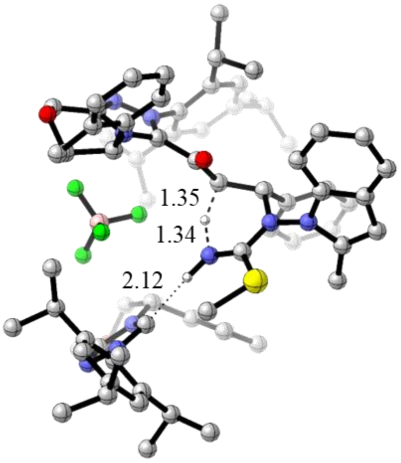

F-TS2-*RR*$_a$

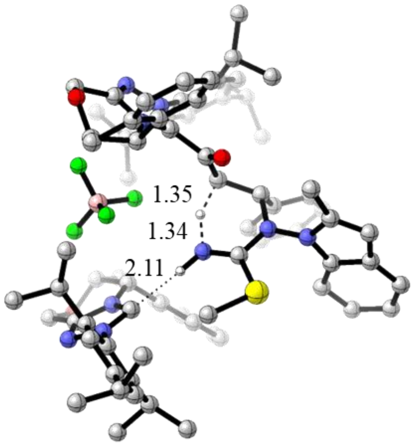

F-TS2-*RS*$_a$

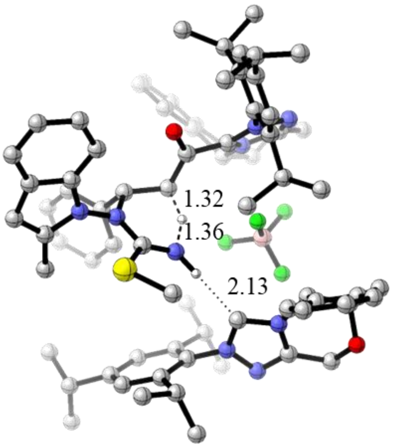

F-TS2-*SS*$_a$

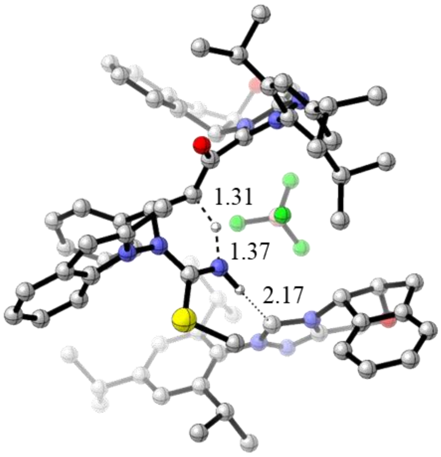

F-TS2-*SR*$_a$

Supplementary Figure 351. Optimized structures of the two enantioselectivity-determining transition states for the reaction catalyzed by **F**. Distances are reported in Å.

13. References

1. Huang, X.-L., He, L., Shao, P.-L. & Ye, S. [4+2] Cycloaddition of Ketenes with N-Benzoyldiazenes Catalyzed by N-Heterocyclic Carbenes. *Angew. Chem.Int. Ed.* **48**, 192-195 (2009).

2. Zhao, C., Li, F. & Wang, J. N-Heterocyclic Carbene Catalyzed Dynamic Kinetic Resolution of Pyranones. *Angew. Chem.Int. Ed.* **55**, 1820-1824 (2016).

3. Marenich, A. V., Cramer, C. J. & Truhlar, D. G. Universal Solvation Model Based on Solute Electron Density and on a Continuum Model of the Solvent Defined by the Bulk Dielectric Constant and Atomic Surface Tensions. *J. Phys. Chem. B* **113**, 6378-6396 (2019).

4. Plata, R. E. & Singleton, D. A., A Case Study of the Mechanism of Alcohol-Mediated Morita Baylis–Hillman Reactions. The Importance of Experimental Observations. *J. Am. Chem. Soc.* **137**, 3811-3826 (2015).

5. Legault, C. Y. *CYLview, 1.0b*, Université de Sherbrooke, 2009 (http://www.cylview.org).

6. Frisch, M. J., Trucks, G. W., Schlegel, H. B., Scuseria, G. E., Robb, M. A., Cheeseman, J. R., Scalmani, G., Barone, V., Petersson, G. A., Nakatsuji, H., et al *Gaussian 16 Rev. C.01*, Wallingford, CT, 2016.

7. Lu, S., Song, X., Poh, S. B., Yang, H., Wong, M. W. & Zhao, Y. Access to Enantiopure Triarylmethanes and 1,1-Diarylalkanes by NHC-Catalyzed Acylative Desymmetrization. *Chem. Eur. J.* **23**, 2275-2281 (2017).
